# Data Mining Strategies to Improve Multiplex Microbead Immunoassay Tolerance in a Mouse Model of Infectious Diseases

**DOI:** 10.1371/journal.pone.0116262

**Published:** 2015-01-23

**Authors:** Akshay Mani, Resmi Ravindran, Soujanya Mannepalli, Daniel Vang, Paul A. Luciw, Michael Hogarth, Imran H. Khan, Viswanathan V. Krishnan

**Affiliations:** 1 Center for Comparative Medicine, University of California Davis, Davis, California, United States of America; 2 Department of Chemistry, California State University, Fresno, California, United States of America; 3 Department of Pathology and Laboratory Medicine, University of California School of Medicine, Davis, California, United States of America; Technische Universität Dresden, Medical Faculty, GERMANY

## Abstract

Multiplex methodologies, especially those with high-throughput capabilities generate large volumes of data. Accumulation of such data (e.g., genomics, proteomics, metabolomics etc.) is fast becoming more common and thus requires the development and implementation of effective data mining strategies designed for biological and clinical applications. Multiplex microbead immunoassay (MMIA), on xMAP or MagPix platform (Luminex), which is amenable to automation, offers a major advantage over conventional methods such as Western blot or ELISA, for increasing the efficiencies in serodiagnosis of infectious diseases. MMIA allows detection of antibodies and/or antigens efficiently for a wide range of infectious agents simultaneously in host blood samples, in one reaction vessel. In the process, MMIA generates large volumes of data. In this report we demonstrate the application of data mining tools on how the inherent large volume data can improve the assay tolerance (measured in terms of sensitivity and specificity) by analysis of experimental data accumulated over a span of two years. The combination of prior knowledge with machine learning tools provides an efficient approach to improve the diagnostic power of the assay in a continuous basis. Furthermore, this study provides an in-depth knowledge base to study pathological trends of infectious agents in mouse colonies on a multivariate scale. Data mining techniques using serodetection of infections in mice, developed in this study, can be used as a general model for more complex applications in epidemiology and clinical translational research.

## Introduction

In biomedical research, many *in vivo* models (e.g., yeast, worms, flies, fish, mouse, rat, monkey etc.) are used, however, the mouse model remains the most useful, widespread and important for biomedical research and clinical relevance [1–5]. Approximately 40 million mice are used in a variety of biomedical/biological research projects, carried out in many academic and industry settings, each year in the US [6]. It is therefore, critically important that the quality of these research animals be carefully maintained. In particular, infectious agents that are common in mouse research colonies must be diligently monitored. Considering the overwhelmingly large numbers of research animals in use, it is imperative that the detection methods be accurate, highly efficient (have a high-throughput), and preferably automated. We have published on the development, validation and clinical implementation of multiplex microbead immunoassays (MMIA) to meet the above objectives by using serological based, routine screening of mouse and nonhuman primate colonies for the specific infectious pathogens to aid in establishment and maintenance of specific pathogen free (SPF) status [7, 8]. In this report, we describe the use of algorithm driven computational approaches for the analysis and continuous interpretation of moderately large volumes of complex sets of data that are obtained in the process of characterizing the status of infectious pathogens in the laboratory mouse. In addition, these studies may provide a system for handling such data in biomedical research, in general (e.g., genomics, proteomics, metabolomics etc.) [9, 10].

As described above, maintenance of SPF mouse colonies is critical for biomedical research. Experimental animals exposed to, or infected with infectious agents may yield questionable data, thereby confounding the findings of a given study. Due to the manipulations of the laboratory mouse genome, an underlying disease, or even an underlying infection without signs of disease, can alter the genotype and phenotype leading to problematic or misleading results [11]. Laboratory mouse strains may be screened for several important infectious pathogens [12–19], as a part of routine colony management practice, in order to maintain well-characterized and reliable experimental systems [20]. Therefore, it is vital that mouse colonies be maintained in a pathogen free environment minimizing the possibilities of disease outbreaks which can not only wipe out a colony but also lead to questionable experimental results [21].

Sero-surveillance is a critical component of maintaining healthy mouse colonies. Monitoring of animals for accurate knowledge of common pathogens is not only desirable for colony maintenance but critical to preserve special mouse strains (e.g., genetically modified or engineered strains). Sera from sentinel mice can be tested with conventional immunoassays via enzyme-linked immunosorbent assay (ELISA) or indirect fluorescent antibody assay (IFA). A critical limitation of conventional immunoassays is that they can detect only a single infectious agent in each serum sample resulting in an inefficient testing system. To circumvent this limitation, it is more efficient to implement a multiplex microbead immunoassay (MMIA) technology. MMIA can measure up to 100 analytes in a single reaction in a high throughput manner [22]. This method has been implemented for a variety of biomedical research and clinical applications (e.g., immunology/transplantation, infectious diseases, cancer, neurological diseases, pediatric medicine etc.). For details the reader is directed to extensive review articles in this area [23–25]. We have successfully implemented MMIA for routine sero-surveillance of mouse colonies at the Comparative Pathology Laboratory (University of California Davis) and Jackson Laboratories (Bar Harbor, Maine, USA). Use of computational methods for data analysis and interpretation served as an integral component of the previous studies. In a proof-of-concept study, Khan and co-workers developed MMIA protocol for serodetection of multiple infectious pathogens [7].

In this study, we provide models of data mining tools to assess large quantities of data generated in the multiplex immunoassay system. We defined a *training set* based on the validated experimental data (simultaneous detection of antibodies to 9 different infectious agents) from 1,161 serum samples [7]. The *testing* data consisted of more than 15,000 animals that were routinely tested at two different animal facilities over two year span. These mice were surveyed for the same set of nine infectious agents. A cumulative validation of these results using data mining tools allows efficient monitoring of the test performance at mouse colonies, possible quality control of the data, as well as evaluation of the inherent tolerance to assay performance as a large number of samples are tested routinely.

Prior to field implementation of an assay, quality of the assay performance is established during the production process. Once the surveys are completed, the information on the performed assays is seldom used for feedback, unless there is a suspected problem with the results. In the era of data driven approaches, knowledge is information and both positive as well as negative feedback on performance is important. The completed multiplex immunoassay assay results on the mice colonies might have several levels of valuable information that could be utilized for improvement at all stages of the pipeline: assay optimization, definition of cutoff to differentiate between true positives vs. negatives, identification of outliers, effect on variation in the quality of consumables, biological variation on the mice colonies, mutational changes of the tested infectious agents and other mouse colony dependent variables. The large amount of previously collected multiplex immunoassay over a two year study period provide model data to explore the utility of the feedback driven optimization of all aspects of assay production, validation, implementation and deployment. This model for analyzing field data will add value in a continuous manner to significantly improving the accuracy of many other biological and clinical assay formats.

## Materials and Methods

*Ethics Statements:(1) Mice were housed at The Jackson Laboratory in AAALAC-approved animal facilities using animal care programs in compliance with the Public Health Service Guide for the Care and Use of Laboratory Animals. This study was reviewed and approved by the institutional animal care and use committee. The name of the committee is The Jackson Laboratory Institutional Animal Care and Use Committee. (2) Regarding amelioration of suffering, we receive mice for health monitoring from all mouse rooms. Mice are bled for serologic testing, the mice are euthanized by cervical dislocation. The mice are then necropsied and samples collected for diagnostic testing*.

### Multiplex microbead immune assay (MMIA)

Viruses purified by sucrose-density gradient were purchased from Advanced Biotechnologies Inc., (Columbia, MD):, Nebraska calf diarrhea virus (NCDV) for epizootic diarrhea virus of infant mice (EDIM), Theiler’s mouse encephalomyelitis virus/GDVII strain(GD7), mouse hepatitis virus (MHV), mouse minute virus (MMV), pneumonia virus (PVM), Sendai virus, and vaccinia virus for ectromelia virus (ECTRO). Respiratory enteric orphan virus (Reo-3 virus) and *M. pulmonis* (MYC) was cultured in our laboratory. Cell lysates were prepared for coating microbead as previously described [7, 8].

All antigens were prepared for bead coupling as previously detailed [7, 8]. The optimal concentration for each antigen was determined by coupling different microbead sets (2.5 × 10^6^ beads/coupling) with a range of protein concentrations for each antigen [7, 8]. Coated microbeads for each antigen were tested with sera from infected mice to select the antigen concentration that displayed the strongest positive signal and lowest background (against normal sera). Optimal concentrations of antigens were covalently conjugated to carboxylated microbead (Luminex Corp., Austin, TX) for large scale coupling (2.5 × 10^7^ beads/coupling) per manufacturer’s instructions (http://www.luminexcorp.com/uploads/data/Technology%20Tips%20FAQs/Recommendations%20for%20Scaling%20Up%20Antibody%20Coupling%20Reactions%200407%2010242.pdf). Three bead sets were coupled as internal controls for the assay: 1) biotin-conjugated goat immunoglobulin G (IgG) (Rockland Immunochemicals, Gilbertsville, PA) at 100 µg/ml as a positive control protein for reaction with streptavidin-R-phycoerythrin, 2) rabbit anti-mouse IgG (Bethyl Laboratories, Montgomery, TX) 1 µg/ml as a positive control for sample addition and 3) bovine serum albumin (BSA;100 µg/ml) (Pierce, Rockford, IL) as a negative control protein.

Multiplex assay was essentially performed and data were collected as previously described [7, 8]. Though the beads generally manufactured from multiple lots over the period of the experiments (approximately two years), antigen preparation and coupling to the beads were performed in a consistent manner as per standard operating procedures. Additional quality control of the various preparation steps reduces any other experimental variations between the various batches and thus increasing the robustness of the data.

### Experimental data for “Training Set”

Two types of plate-control samples were used in each multiplex immunoassay experiment: positive (high and low titer) and negative for antibodies against all nine pathogens. Two positive control samples, one with high and the other with low antibody titer representing each pathogen, were generated [8]. To generate these positive control samples, single positive sera (one high and one low titer) per pathogen, from mice infected with each of the pathogens were selected. Such individually selected sera for each of the nine pathogens were then mixed in equal proportion to obtain two separate pools (one high and one low titer) of positive control sera. For negative plate-control, samples from uninfected mice, confirmed to be negative for antibodies against all 9 pathogens by ELISA were pooled. Aliquots of the positive and negative plate-controls were prepared and stored frozen at -80°C until used.

Assay reproducibility was determined as percent variation of multiplex assay results for the positive control samples with data were obtained from twelve independent experiments (performed at CCM). Plate-to-plate variation was estimated to be 2 to 6% [8]. The overall percent variation among other sites ranged from 13 to 15% with the exception of one infectious agent, Mycoplasma, which was 20%.

### Experimental data for the “Test Set”

Data from the routine field testing were generated at Jackson Laboratory (Jax) and Comparative Pathology Laboratory (CPL), University of California, Davis over a period of two years. These data are termed as “Test Data Set”. Both the plate-control and “Test Data Set” are not specific to a particular mouse strain. Once the assay has been performed at the respective laboratories, the results were sent back to CCM via email (MS Excel format) periodically. Each file was labeled with the date on which the experiments were performed. All the Excel sheets were combined using ‘awk’ scripts which were written in-house. During the routine field testing, assays were performed on single sample basis (not in duplicate). Assay reproducibility at the implementation site was measured independently and the percent variations among different microbead sets were similar between Jax and CPL but higher than those at CCM (approximately 6 to 10%) [8].

### Study design for data mining

**Figure 1:**
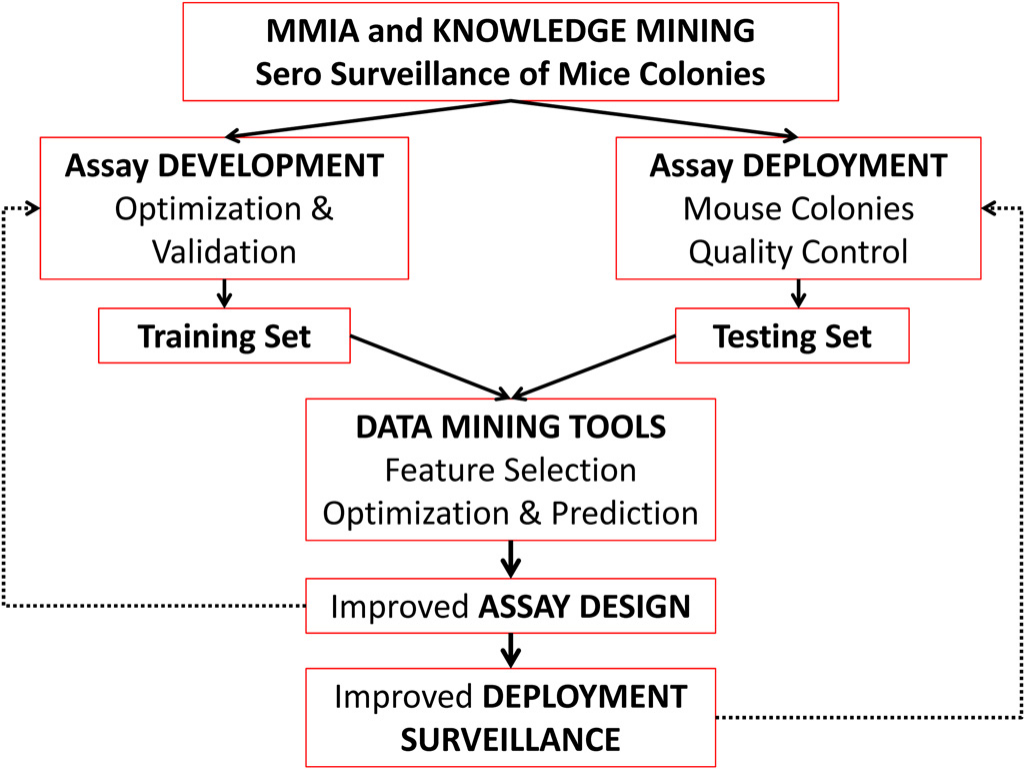
Flow chart of the data mining approach sero-surveillance of mice colonies. Multiplex microbead immunoassays (MMIA) results developed to detect multiple viruses are optimized and validated to develop the training set. Assays deployed in mice colonies were then used as the test set using a variety of data mining algorithms. This process leads to a better detection, optimization, and deployment of sero-detection of infectious diseases in mouse colonies. The proposed approach can be dynamically implemented for any large scale diagnostics for clinical and translational research applications.

Fig. 1 shows the flowchart of the overview of the workflow. “Training Data Set” was developed using the extensively validated data from experimentally infected animals [7, 8]. The data were screened to ensure for the quality, reproducibility and assay variability within and between established CV of the experiments [8]. Baseline values of each microbead set for reactivity against samples from normal (healthy/uninfected) mice were first established in terms of a raw median fluorescent intensity (MFI). Using a combination of multivariate statistics that accounts for assay interference, *cutoff* value for each microbead set was defined (see below).

### Construction of “Training Data Set”

Data from previously published validation study were used to construct “Training Data Set”. These data consist of 1,632 serum samples for all the 9 pathogens collected in duplicate. MFI values from BSA coated microbeads (see antigen preparation and coupling to microbead) in the multiplex assays indicated non-specific sample reactivity [8]. Typically a MFI value > 100 in a sample for BSA microbead suggested a non-specific cross reactivity. Such non-specific cross reactivity is a significant source for misclassification (Fig. 1 and Table 2 of Ravindran et al. [8]). Therefore, all samples with MFI values for BSA microbead > 100 were removed, leaving a total of *1,161* samples.

### Determination of assay cutoff value

Raw MFI values from the “Training Data Set” were used without further modification. Robust linear regression was performed on the observed versus theoretical quantiles to determine which linear transformation of the *t* statistics would confer a normal distribution and then scaled accordingly by using a quantile normalization procedure. Data were visualized with box-and-whisker plots and scatter plots prior to analysis. Measured MFI levels were adjusted to the same interquartile range. A linear model fit was determined for each pathogen by using the LIMMA package in R. Three different groups were defined for the linear model: (a) normal samples, (b) background values from single-positive sera that were positive for one pathogen and negative for other eight non-specific pathogens and (c) signals from positive control samples. Mean values were estimated using linear modeling for each of the groups (each measurement was performed in duplicates) according to a previously described procedure [24, 26]. Briefly, changes were calculated across the three (a, b, and c) conditions by fitting a linear model to the data, and the statistical significance was estimated by a two-step process. First, an analytical data set that encompassed each group for which a significant signal was detected in at least one comparison between normal (or non-specific background from single positive sera) was obtained. This was followed by differential expression across the multiple comparisons detected by an *F test* along with separate *F tests* for each pathogen. Fold changes were estimated and *p values* were adjusted by using the Benjamini-Hochberg procedure (data not shown). Estimated mean values of each group are listed in **Table A in** S1 File (supporting information).

Cutoff values were calculated as described earlier from two background sources [8]: (a) background from normal samples (Bkgrd) is defined as the mean MFI of each microbead against the normal samples, (b) Single-positive sera are positive for antibodies to one but are negative for antibodies to eight, non-specific infectious agents giving rise to background (mean MFI) against each of the nine non-specific microbeads sets (S-P Bkgrd). Using background from these two sources, the cutoff (CO) value for each microbead set is calculated as: “Normal and S-P Bkgrd + (3 × SD)”, where SD is the standard deviation of the respective backgrounds. This definition of cutoff assumes that the data in each sub-group is normally distributed. For example, for the GD7 microbead, cutoff value is MFI of 90; reactivity of any sample against GD7 microbead >90 is considered positive for infection by GD7. The cutoff for each infectious agent is different and ranges from 72 to 246 (Table 1).

**Table 1:** Experimentally validated cutoff values of serodetection by MMIA.

**No**	**Virus**	**Microbead**	**MFI-cutoff**
1	Epizootic diarrhea virus of infant mice	EDIM	141
2	Theiler’s mouse encephalomyelitis virus/GDVII strain	GD7	90
3	Mouse hepatitis virus	MHV	177
4	Mouse minute virus	MMV	246
5	Mycoplasma pulmonis	MYC	217
6	Pneumovirus of mouse	PVM	92
7	Respiratory enteric orphan virus (Reo-3 virus)	REO	125
8	Sendai virus	SEN	233
9	Ectromelia virus	ECTRO	217

### Construction of “Testing Data Set”

Experimental data from two sites (Jax and CPL) where the multiplex microbead immunoassay has been implemented for the routine testing of mice for infectious agents were used to construct the “Testing Data Set” as follows: CPL, UC Davis (n = 3850) and Jax (n = 15350) to assess the 9 classes (sensitivity and specificity of the nine microbead sets). All the MMIAs were performed with an internal control of BSA coated microbead to determine the background reactivity of each mouse sample [7]. As in the case of the “Training Data Set” design, all samples with BSA levels > 100 (MFI) were removed to produce a final testing set of 15,403 samples for all 9 microbeads. Data reduction (removal of serum samples due to non-specific interactions or cross reactivity) in both the “Training Data Set” and “Testing Data Set” are not location dependent. Approximately same percentage of data were eliminated from both the locations (due to non-specific reactivity of samples against BSA microbead) during data filtering process in the construction of the “Training Data Set” and “Testing Data Set”, 20.3% and 19.7%, respectively.

### Data mining

Several classification schemes following the flow chart (Fig. 1) were implemented. Identification of 9 different infectious agents was represented with their respective classification labels and each mouse was matched with their attribute vector. The final dimensions of the testing and training sets were *15,403 and 1,161*, respectively. (The “Training Data Set” and “Test Data Set” are included in the supporting information Tables B and C in S1 File). Weka version 3.5.7 (http://www.cs.waikato.ac.nz/ml/weka/), developed by the University of Waikato in New Zealand, is a software collecting a variety of state-of-art machine learning algorithms was employed [27–29].

### Performance of the “Training Data Set”

The quality of the “Training Data Set” was evaluated by three different classification schemes (J48, Bayes-net and Random Forest) using Weka. J48 algorithm based on the statistical classification system of C4.5 which is known to perform well against a sample distribution [30], Bayes-net utilizes a probability based evaluation [31] while random forest [32] allows multiple models, a popular classification scheme. For each algorithm the training set was cross validated with various ‘k’ values (k = 5, 10, 15 and 20) and the corresponding average of the true positive rate was measured. The dataset was split into ‘k’ equal size partitions at random. Each partition is used for testing in turn and the rest is used for training, i.e., each time 1/k^th^ of the dataset is used for testing and the rest for training, and the procedure is repeated ‘k’ times so that each data is used for training and testing exactly once. As no significant changes in the true positive rate were measured for k > 10, results of the ten times cross validated “Training Data Set” were used. No additional modifications were done to the data. The three algorithms used for validating the training set are part of the top ten classifications schemes often used in the literature [33] and the details of implementing the schemes are provided by Witten et al. [29].

### Application to the “Testing Data Set”

The “Training Data Set” developed above was used for the performance evaluation of the “Testing Data Set”. We employed 26 different algorithms (Meta Class Classifier, J48, SMO, Simple Logistic, Multilayer Perception, Lazy ibk, LMT, Rules Decision table, Meta Bagging Meta Logi Boost, Kstar, Rules PART, REP Tree, Meta Random Committee, Random Forest, Random forest subspace, Classification via Regression, Logistic, Rules One R, Rules Zero R, Bayesnet, Random tree, Naives Bayes, Meta Filtered Classifier, NaiveBayes Updateable and Attribute Selection Classifier) using the “Training Data Set” (1,161 samples) on the “Test Data Set” (15,403 samples). The quality of the “Training Data Set” is well characterized by the experimental validation [8] and therefore three well-established classification schemes were sufficient. As we wanted to establish a model that predicts the best outcome from the “Testing Data Set”, we have implemented a range of algorithms (26 in total, Supporting information).

### Performance Measures

True positive (TP) provides the measure of number of positive events positive for a virus infection and true negative (TN) provides the number of negative occurrences predicted correctly under a given classification scheme. False positive (FP) gives an estimate of negative events that are incorrectly predicted to be positive, while the false negative (FN) estimated the number of mice that were predicted negative but were positive [34].

For multi-class classification schemes and the sum over rows (i) or columns (j) of the confusion matrix (*M*) should be considered. For a confusion matrix of dimensionk×k, the TP, TN, FP and FN for the measure (class) ‘n’ could be defined as follows:

$$TP={M_{ii}|}_{i=n};\;TN=\sum_{i=1}^{k}{M_{ii}|}_{i\neq n};\;FP=\sum_{i=1}^{k}{M_{ij}|}_{i\neq n};\;FN=\sum_{j=1}^{k}{M_{ij}|}_{j\neq n}$$ [1]

These terms were combined to determine the performance of our testing *via* quantifiable categories such as sensitivity (SN), specificity (SP), positive predictive value (PPV), negative predictive value (NPV), test efficiency/accuracy (TE) and Matthew correlation coefficient (MCC). These quantifiers are defined as follows:

Sensitivity (SN) gives an estimate of the percentage of actual positives identified, while specificity (SP) gives an estimate of the percentage of negatives identified.

$$SN\left(\%\right)=\frac{TP\times 100}{TP+FN}$$ [2]

$$SP\left(\%\right)=\frac{TN\times 100}{FP+TN}$$ [3]

The effectiveness of a test is evaluated based on two measures namely, positive predictive value (PPV) and negative predictive value (NPV). PPV gives an estimate of the percentage of positive samples that were correctly predicted and NPV gives the percentage of negative samples that were correctly predicted [35, 36].

$$PPV\left(\%\right)=\frac{TP\times 100}{TP+FP}$$ [4]

$$NPV\left(\%\right)=\frac{TN\times 100}{FN+TN}$$ [5]

The prediction power of a model can be evaluated either by test efficiency (TE) or Mathew correlation coefficient (MCC) [37]. Test efficiency is also referred as *test accuracy*. The MCC is in essence a correlation coefficient between the observed and predicted classifications; it returns a value between −100% and +100%. A coefficient of +100% represents a perfect prediction, 0% no better than random prediction and −100% indicates total disagreement between prediction and observation [38]. TE and MCC are defined as follows.

$$TE\left(\%\right)=\frac{\left(TP+TN\right)\times 100}{TP+TN+FP+FN}$$ [6]

$$MCC\left(\%\right)=\frac{\left(TP\times TN-FP\times FN\right)\times 100}{\sqrt{\left(TP+FP\right)\left(TP+FN\right)\left(TN+FN\right)\left(TN+FP\right)}}$$ [7]

### Assay tolerance and effect of cutoff variation

As mentioned above (Table 1) the experimentally validated assay values [8] were used to define the various sub classes (specific reactivity of each sample against the nine microbeads, representing nine pathogens) within the “Testing Data Set”. In order to determine the tolerance (sensitivity and specificity) of our assay, we systematically and incrementally reduced the cutoff values. The cutoff values were categorized by increments of 5%, 15%, 25%, 40%, 55%, 75% and 90% of the experimental values (represented by 0%). The class definitions were reevaluated in each case and a ‘new’ “Test data Set” is redefined for evaluation. The top three performers of the 26 classification schemes applied earlier (see above) (Meta Class Classifier, J48 and Simple Logic) were used to for performance evaluation whenever the test set is redefined with a varying cutoff value. In addition to using Weka for classification, rest of the data analysis was performed using a combination of Excel macros, awk and Perl scripts or R statistical environment [39].

## Results

### Quality of training data set

**Figure 2:**
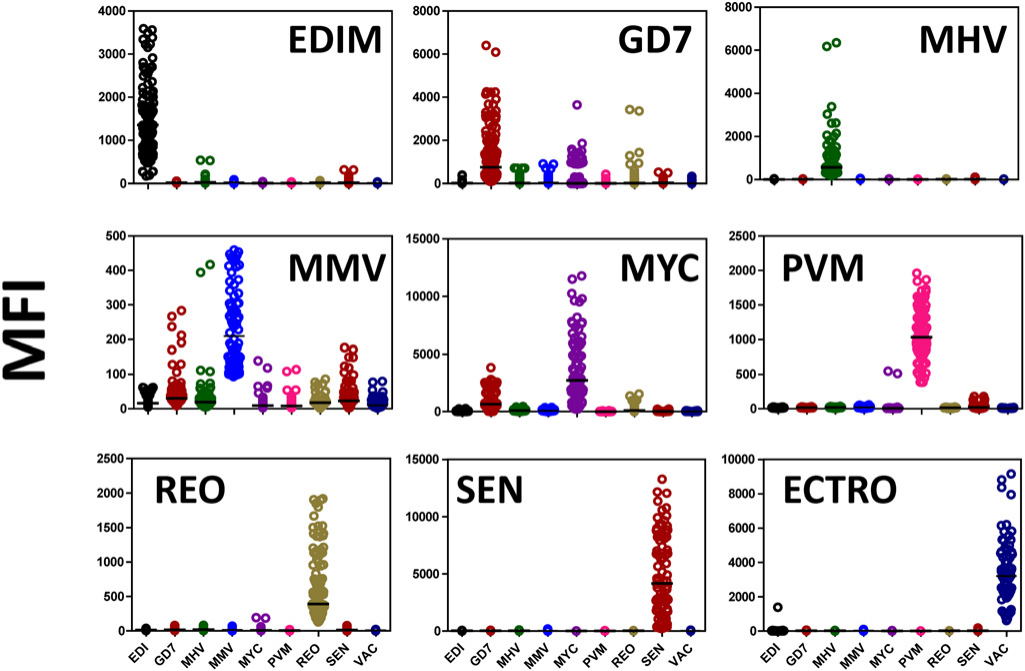
Distribution of median fluorescent intensities (MFI) within the training data for all the nine viruses. Each panel identifies the simultaneous detection of all the nine viruses with the highest value representing the mice that are positive for particular virus that is identified on the top. The abbreviations of the viruses are as follows: Epizootic diarrhea virus of infant mice (EDIM), Theiler’s mouse encephalomyelitis virus/GDVII strain (GD7), Mouse hepatitis virus (MHV), mouse minute virus (MMV), Mycoplasma pulmonis (MYC), Pneumovirus of mouse (PVM), Respiratory enteric orphan virus (Reo-3 virus) (REO), Sendai virus (SEN) and Ectromelia virus (ECTRO).

**Figure 3:**
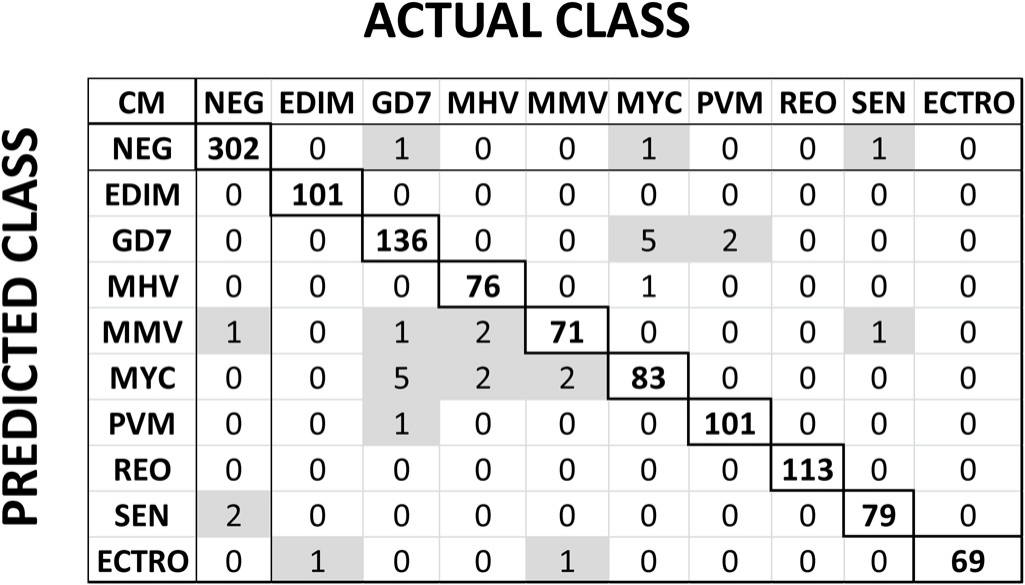
Confusion matrix (CM) of the training data set. Experimentally validated assay results were first pruned for inconsistencies via the three different standard classification algorithms (results from J48 is presented here). Numbers in the diagonal identifies the number of mice correctly identified (true positives) by the algorithm with upper and lower diagonal numbers are the corresponding false positive and false negative occurrences, respectively. Of the 305 total samples identified to be negative for any virus (NEG), three mice show false negative (one MMV and two SEN). The abbreviations of the viruses are as follows: Epizootic diarrhea virus of infant mice (EDIM), Theiler’s mouse encephalomyelitis virus/GDVII strain (GD7), Mouse hepatitis virus (MHV), mouse minute virus (MMV), Mycoplasma pulmonis (MYC), Pneumovirus of mouse (PVM), Respiratory enteric orphan virus (Reo-3 virus) (REO), Sendai virus (SEN), Ectromelia virus (ECTRO) with NEG stands for mice that are negative for any infection from these viruses.

Two key sets of data were used in this study. One data set was obtained using groups of experimentally infected animals, positive for antibodies against one infectious pathogen per group [7, 8]. This data set was termed “Training Data Set”. Distribution of this data set is shown in Fig. 2. MFI values for the detection of antibodies against each infectious agent (including negative controls) were plotted. These MFI values range between 0–500 (MFI units) for MMV to 0–15,000 (MFI units) for SEN. Thus, the training data set displayed a large dynamic range. Confusion matrix derived from the above training set was classified using the J48 algorithm (10 fold cross validation) is shown in Fig. 3. Cross validation of the models is critical in assessing how well the sub-groups could be considered independently within the statistical analysis. This reflects the accuracy of the predictive model in practice and provides a meaningful assessment tool. The confusion matrix correctly projected 97% of classification samples from animals with antibodies against different pathogens (negative samples were predicted correctly in 302 out of 305 samples). Performance measures of the “Training Data Set” were predicted using J48 algorithm as listed in Table 2. The Mathew Correlation Coefficient (MCC) for each feature was above 77% (except for MMV) and provides us with the necessary confidence in using this “Training Data Set”. The performance measures were similar for the other two classification schemes, Bayes-net and Random Forest (Supporting information Table D in S1 File).

**Table 2:** Performance of the “Training Data Set” using the classification algorithm J48.

**No**	**CLASS**	**SP (%)**	**SN (%)**	**PPV (%)**	**NPV (%)**	**TE (%)**	**MCC (%)**
0	**NEG**	98.6	99.7	99.8	98.6	99.6	98.3
1	**EDIM**	99.8	86.5	88.6	99.8	99.5	87.3
2	**GD7**	99.8	84.5	80.6	99.8	99.7	82.3
3	**MHV**	99.9	85.7	88.2	99.9	99.9	86.9
4	**MMV**	99.7	77.9	70.7	99.7	99.5	73.9
5	**MYC**	100.0	96.1	100.0	100.0	100.0	98.0
6	**PVM**	99.8	96.2	97.8	99.8	99.5	96.8
7	**REO**	99.9	85.4	72.9	99.9	99.9	78.8
8	**SEN**	99.9	93.3	63.6	99.9	99.9	77.0
9	**ECTRO**	99.9	82.4	74.7	99.9	99.8	78.3

### Predictive values of different classification algorithms

Predication of assay performance using the training set on the testing set is sensitive to the choice of the classifier (algorithm) for a given distribution of data. Therefore, it is important to evaluate the predictive results using many algorithms [40]. Our analysis utilized several predictive algorithms and displayed the relationship of the data distribution within a confusion matrix in order to determine the threshold dependent parameters for the various classifiers. Twenty six algorithms were used in this study for their predictive value to better understand the performance of the “Test Data Set” (positives defined by experimental cutoff values) measured in terms of testing efficiency (TE) and Mathew correlation coefficient (MCC), against the “Training Data Set”. The results from the top three algorithms are presented in Table 3. (Predictive measures under all the algorithms are given in Table E in S1 File). The TE values of the negative samples for the “Training Data Set” were used to identify the best performing algorithms: Meta class classified, J48 and Simple Logic provide a TE values of 90.8%, 88.4% and 85.7%, respectively (Table 3). For the “Test Data Set”, overall, these algorithms predicted a high TE scores for the majority of the infectious agents. Meta class classifier predicted a higher MCC (59.2%) for the negative samples (Table 3). Contrasting the testing efficiencies and MCC, one can assign a priority in the importance of algorithms with meta-class classifier at the top, followed by J48 and Simple Logic, relevant to all nine infectious agents as well as the negative samples. The other measures of performance (sensitivity, specificity, PPV and NPV) also follow the same trend as TE. The sensitivity and specificity value via MCC for meta-class classifier are high (TE > 86%) which shows its accuracy in predicting the relationship between the variables (Tables 2 and 3).

**Table 3:** Performance evaluations of the test set using the training set.

**Test Efficiency (TE) %**											
No	Algorithms	NEG	EDIM	GD7	MHV	MMV	MYC	PVM	REO	ECTRO	SEN
1	Meta Class Classifier	90.8	97.3	98.8	99.8	95.1	99.7	95.8	99.9	99.5	99.0
2	J48	88.4	97.9	98.4	99.8	89.4	99.1	91.1	99.7	99.4	99.9
3	Simple Logistic	85.7	97.0	94.6	99.2	92.6	99.6	90.5	99.7	99.5	99.8
**Mathew Correlation Coefficient (MCC) %**											
No	Algorithms	NEG	EDIM	GD7	MHV	MMV	MYC	PVM	REO	ECTRO	SEN
1	Meta Class Classifier	59.2	58.2	6.7	79.9	2.7	83.4	70.9	79.3	39.9	16.6
2	J48	16.5	32.0	5.2	77.6	18.8	25.3	11.0	38.4	16.3	40.2
3	Simple Logistic	37.9	35.1	27.5	62.2	2.9	80.1	8.4	55.8	46.1	45.0

### Tolerance limit for the detection of antibodies against each infectious agent in the multiplex assay

**Figure 4:**
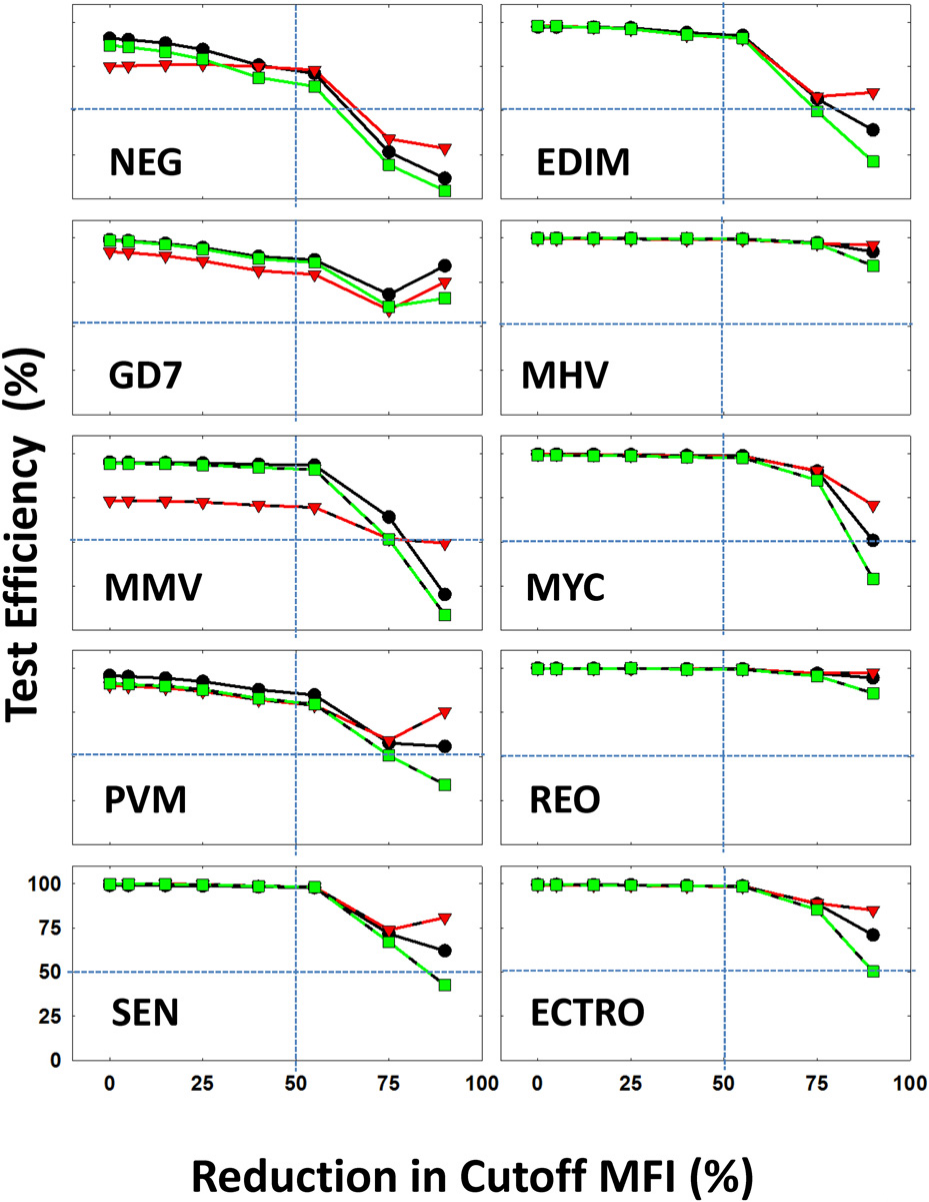
Assay tolerance performance in terms of test efficiency: The variation in the test efficiency (%) is plotted as a function of reduction in the experimental cutoff that is used to define the mice that are positive for each virus (marked) in each panel. The variation is calculated for three of the top performing algorithms: Meta Class Classifier (black symbols/lines), J48 (red symbols/lines) and Simple Logic (green symbols/lines). The experimental cutoff value is plotted at 0%. The 50% values along the X- and Y-axes are marked by vertical dotted lines. The abbreviations of the viruses are as follows: Epizootic diarrhea virus of infant mice (EDIM), Theiler’s mouse encephalomyelitis virus/GDVII strain (GD7), Mouse hepatitis virus (MHV), mouse minute virus (MMV), Mycoplasma pulmonis (MYC), Pneumovirus of mouse (PVM), Respiratory enteric orphan virus (Reo-3 virus) (REO), Sendai virus (SEN) and Ectromelia virus (ECTRO).

**Figure 5:**
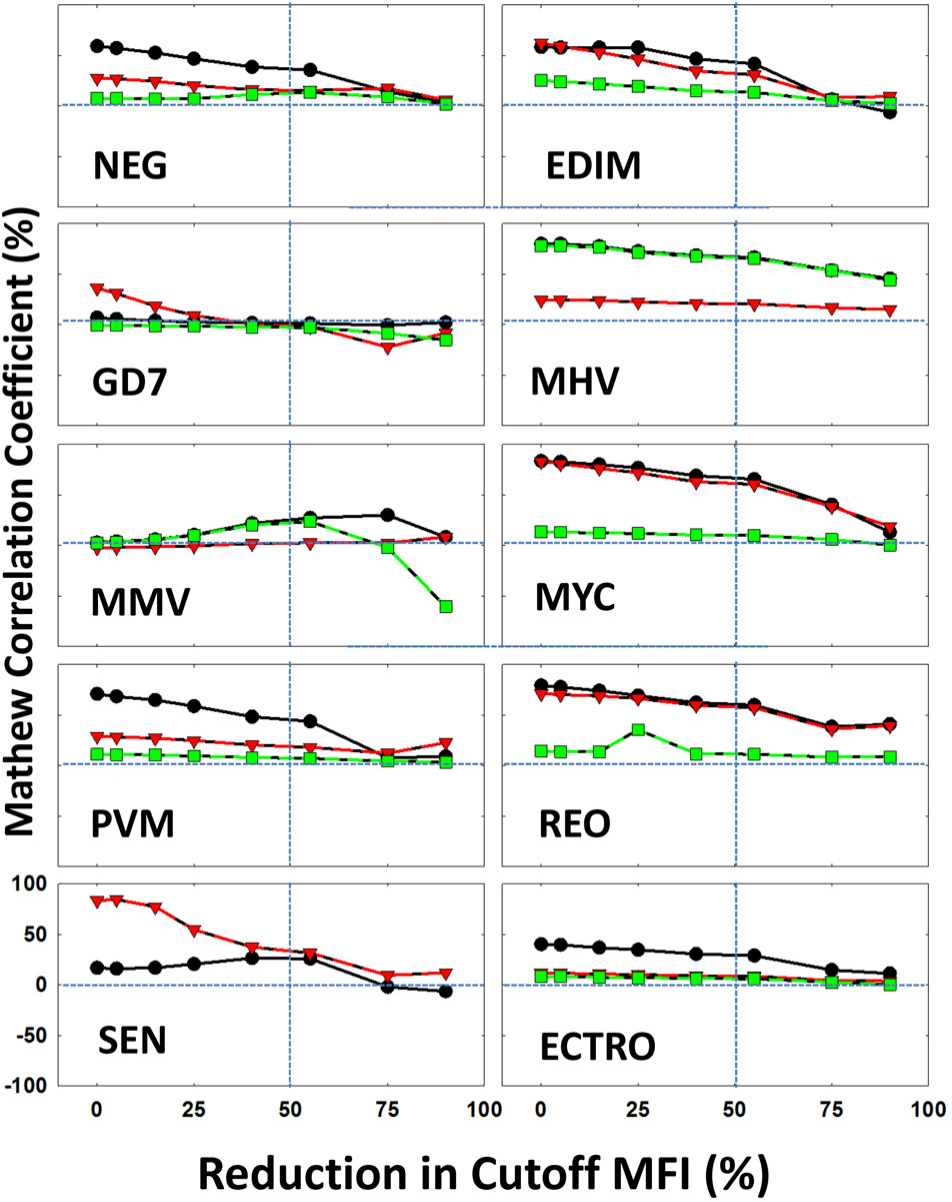
Assay tolerance performance in terms of Mathew Correlation Coefficient (MCC): The variation in the MCC (%) is plotted as a function of reduction in the experimental cutoff that is used to define the mice that are positive for each virus (marked) in each panel. The variation is calculated for three of the top performing algorithms: Meta Class Classifier (black symbols/lines), J48 (red symbols/lines) and Simple Logic (green symbols/lines). The experimental cutoff value is plotted at 0%. The 50% values along the X-axis and 0% along the Y-axis are marked by vertical dotted lines. The abbreviations of the viruses are as follows: Epizootic diarrhea virus of infant mice (EDIM), Theiler’s mouse encephalomyelitis virus/GDVII strain (GD7), Mouse hepatitis virus (MHV), mouse minute virus (MMV), Mycoplasma pulmonis (MYC), Pneumovirus of mouse (PVM), Respiratory enteric orphan virus (Reo-3 virus) (REO), Sendai virus (SEN) and Ectromelia virus (ECTRO).

Within the classifiers that are mentioned above, we were able to characterize ~ 85% of the data with reliable test efficiency (Table 3). In a multiplex format, the assay sensitivity for the detection of antibodies against a particular pathogen depends on the quality and performance of the assay for the detection of the other pathogens where interactions among various microbead sets as well as antibodies in the sample may affect the accuracy of detection of some or all infectious agents. The cutoff values for all the microbead sets in the multiplex assay were defined for each pathogen (Table 1) [8]. We employed the data mining tools to evaluate the tolerance of the overall assay, as it accounts for individual sensitivity of each microbead, its interactions with other microbeads within the multiplex assay as well as the sample, and takes into account the assay background variation. To test the limit of tolerance to accuracy of each microbead set we systematically altered the cutoff values of each microbead and evaluated the overall performance (Figs. 4 and 5). This was conducted by reducing the cutoff value for each class by a predefined percentage (Materials and Methods). For example in the case of EDIM, the defined cutoff value was 141; we reduced this value by 5% which gave us 134. With the reduced cutoff, MCC was calculated for the three algorithms.

By reducing the cutoff values of detection for each pathogen, the accuracy was reduced based on individual classifiers altering the relative rate of reduction for each bead set (Figs. 4 and 5). Furthermore, it was observed using the same “Training Data Set” and “Test Data Set” with different classifiers that the prediction profile varies from one classifier to another (Figs. 4 and 5). In the case of EDIM, ECTRO, NEG, MHV, PVM and MYC it was observed that performance of all the three algorithms reduced consistently (75% performance reduction). When examining GD7, the performance of Meta-class classifier and Simple Logic are reduced after 75% reduction even with J48 performance being increased. This could be the result of noise in the dataset. In the case of REO, the performance of Simple Logic was different when compared to the other two algorithms at a 55% reduction. One such example is seen in the case of SEN, where it was observed that the performance of the classifiers was reduced after 55% based off the cutoff value. The reason for the inconsistent performance at certain stages by some algorithms is due to the presence of points/data (noise) on the dataset that cannot be classified by the algorithm of choice.

## Discussion

Data mining techniques provide efficient and effective tools to observe and provide plausibility to compare large volumes of data by enabling elucidation of important patterns and correlations. The power of pattern recognition (data mining) to a large extent depends of the quality of the training data set [27, 29, 33, 41, 42]. In this study, the “Training Data Set” was defined by the experimental assay optimization [7, 8]. In order to verify the classification power of the “Training Data Set”, we employed three most commonly used classification schemes (J48, Bayes net and Random forest) with 10-fold cross validation (Figs. 2, 3 and Table 2). These training data (1,161 × 9 infectious agents+ normals) is then used on the field employed “Test Data Set” (Table 3). We have evaluated the performance of 26 different algorithms in terms of performance measures (and supporting information Table E in S1 File). More importantly, we have changed the assay cutoff value (derived from training set) systematically to test the tolerance of the field implemented test data (Figs. 4 & 5).

According to Fayyad et al some of the common critical factors in data mining protocols include: selection, pre-processing, data mining, and interpretation [43]. These steps are essential for reducing large and complex datasets in order to identify and quantify patterns for research analysis. The purpose of our study was to decipher and develop a model to simultaneously detect various pathogens within mouse populations by a multiplex immunoassay. During the initial stages of data collection, a series of reductions were done by removing outliers and missing data points. As a consequence of measuring all the antibodies in the same vessel (multiplexing), the overlap between different groups in the data becomes inevitable, in particular due to lack of specificity in antibody-antigen interactions in an immunoassay format. In other words, an object in a class has similar properties as those in other classes [44–46]. Even when the features are well-chosen and the data have good quality (well sampled data with less noise) the results of the classification will frequently vary with the choice of different algorithms and respective parameters [44–46]. Despite the long tradition of data mining research, there are no definite guidelines for choosing classifiers, and one is faced with the need to choose a method that best suits the given problem at hand.

A particular work of interest is the comprehensive study presenting the top 10 data mining algorithms identified by the IEEE International Conference on Data Mining by Wu et al [33, 47]. One of our top three performers in this study (J48) which was based on C.4.5 scheme (generates classifiers expressed as decision trees) [30], is the top performer listed by the above reference. Many comparative studies, similar to the present one, are specific to a given problem or task. Perhaps this is a consequence of the “No Free Lunch theorem”, which states that, without any prior knowledge, no single method can be preferred [48–51]. The appropriate choice of parameters requires certain knowledge of the underlying mechanisms behind both the biological nature of the assay and how algorithms treat the data with their default configuration settings for each algorithm. In a recent comparison of supervised classifiers using an artificial database, Amancio et al., [48] noted that the default parameters of Weka provides reliable and consistent results. Implementing Weka software, we evaluated the performance of several classifiers using default parameters in order to determine the feasibility to improve overall assay tolerance of multiplexed and multivariate data. We should emphasize that the choice of classifier is specific to the current data and may not be considered universal.

We obtained a “Test Data Set” from routine testing of nine infectious agents over a period of two years utilizing the implementation of multiplex serodetection of pathogens in multiple mouse colonies. A “Training Data Set” was obtained from experimentally inoculated groups of mice; one infectious agent per group. By creating a “Test Data Set” and a “Training Data Set”, we were able to optimize the Weka functionality via machining learning to better classify our data by mining the data which enables us to quantify our performance that was evaluated on the test set. This process was carried out multiple ways and was schematically tested through Weka’s data mining functionality.

A traditional statistical approach is highly essential in analyzing the experimental data at the time of validating the assay in order to establish the baseline for the cutoff values to distinguish true positive and false negative rates. Nevertheless it would be difficult to predict any implementation-dependent variations that might affect the assay performance over a longer period of time, since the assay is performed several times with new batch turnover. Several reasons contribute to any possible variations, both at the production of the assays and at their implementation: Production variations include the quality of the materials used in the reactions, antibodies produced, and technical variation, whereas at the at the implementation end the variations may be from evolutionary sequence changes in the viruses and their interaction with the mice as well as technical variations such as day-to-day logistics of different personnel doing the tests. Implementing a systematic data feedback routine, in addition to improving the tolerance of the assay performance as demonstrated here are valuable tools that can be implemented routinely in any biological or clinical assay setting. This approach is also capable of identifying other quality control issues at the production side to prompt trends in increase or decrease of specific infections in the mouse population via implementation design.

This study demonstrates that large volume, multiplex data can be efficiently and effectively mined using mining techniques. This approach may also enhance the understanding of disease detection by revealing patterns of blood based biomarkers that are otherwise obscured by the shear enormity of large data volumes. Our aim was to use a data mining approach to convert information into knowledge. Accordingly, the information generated from multiplex immunoassays is utilized to develop a predictive model for improving the accuracy of serodetection of pathogens within laboratory mouse populations. The prediction protocol is promising but the amount of information included may not be sufficient enough to fully exploit the prediction strategy. Our results have shown that computational techniques can be used optimally for the detection of pathogens with a high level of accuracy. In our studies, the accuracy and robustness of classification patterns that the Weka’s algorithms provided significantly improved and added value to the previous standard experimental testing [8]. The predictor was found to perform appropriately when applied to a test set that follows the same prospective distribution of the training set. If a change is introduced into the arrangement of classes within the test set, then the performance of the model will decline [52]. This often results in slow training time and difficulty in data interpretation. The disadvantages can be overcome if the tools are upgraded in a timely fashion by implementing more advanced features. The benefit of this inexpensive approach is that it contains a range of features that can help in construction of analytical models [53].

In conclusion, this study showed the usefulness of computational tools for data management, data classifying, and data assessment for understanding the multiplex immunoassays during intermediate stages, thus enabling improvements in the assay. As the size of the data increased, these techniques have helped in the improvement of predictive models. The model can be improved by using more information and classification vectors offered by data mining techniques. Although we focused on multiplex antibody detection of infectious agents in mice, the tools and techniques developed in our project have implications for the implementation of computational analysis of large volumes of data obtained in clinical translational research including proteomics, metabolomics or functional genomics.

## Supporting Information

S1 File Contains Tables A-E. **Table A.** Training Data Set: Mean values of MFI calculated using multivariate statistics. **Table B.** Training Data Set: Experimentally optimized testing data set for 10 attributes (nine infectious agents and normal). **Table C.** Testing Data Set: Sero-detection of infectious agents from data deployed two different animal facilities. **Table D.** Confusion matrices of the training set for Bayesnet and Random Forest algorithms. **Table E.** (a) Performance of the testing set with various classification algorithms—Test Efficiency and (b) Performance of the testing set with various classification algorithms—Mathew Correlation Coefficient (MCC).
(PDF)
