# Supplementary material for: Data Mining Strategies to Improve Multiplex Microbead Immunoassay Tolerance in a Mouse Model of Infectious Diseases
Source: PLoS One. 2015 Jan 23;10(1):e0116262. doi: 10.1371/journal.pone.0116262 (PMC4304816; doi:10.1371/journal.pone.0116262)
Supplement: S1 File — Table A. Training Data Set: Mean values of MFI calculated using multivariate statistics. Table B. Training Data Set: Experimentally optimized testing data set for 10 attributes (nine infectious agents and normal). Table C. Testing Data Set: Sero-detection of infectious agents from data deployed two different animal facilities. Table D. Confusion matrices of the training set for Bayesnet and Random Forest algorithms. Table E. (a) Performance of the testing set with various classification algorithms—Test Efficiency and (b) Performance of the testing set with various classification algorithms—Mathew Correlation Coefficient (MCC). (PDF) [file pone.0116262.s001.pdf]

Table S1: Training Data Set: Mean values of MFI calculated using multivariate statistics.

No	Virus	Microbead	Number	(a) Normal Samples (n=305)		(b) Background values from single-positive sera that positive for to one etiology but are negative for other eight non-specific infectious agents		(c) Positive Samples	
				Mean	STD	Mean	STD	Mean	STD
1	Epizootic diarrhea virus of infant mice	EDIM	101	22	12	21	20	1545	28
2	Theiler's mouse encephalomyelitis virus/GDVII strain	GD7	143	12	4	14	17	975	28
3	Mouse hepatitis virus	MHV	77	12	3	15	47	1362	75
4	Mouse minute virus	MMV	76	24	25	16	44	1427	74
5	Mycoplasma pulmonis	MYC	92	13	8	34	48	6147	120
6	Pneumovirus of mouse	PVM	102	16	13	12	8	1162	11
7	Respiratory enteric orphan virus (Reo-3 virus)	REO	113	14	13	17	18	1120	28
8	Sendai virus	SEN	81	13	8	49	96	6181	133
9	Ectromelia virus	ECTRO	71	15	5	16	62	3447	85

Table S2: Training Data Set

Train	EDI	GD7	MHV	MVM	MYC	PVM	REO	SEN	VAC	class
1	1038.5	13	15	16	16.5	8	8	69	9	EDI
2	1119	13.5	15	16	19	7	9	78	8	EDI
3	1796	15	14	8	7	7	12	8	9	EDI
4	1673	14	15	8.5	9	7	13	9	9	EDI
5	1774	13	14	13	9	9	16.5	19	9	EDI
6	1279	12	12	10	7	6	11	15	8	EDI
7	2221	10	10	6	21	5	5	8	8	EDI
8	1390.5	9	12.5	19	10	3	4	17	1978	VAC
9	1717	12	21	10	7	9	26	8.5	7	EDI
10	2134	13	21	11	8	9	31	8.5	7	EDI
11	1151	14	15	10.5	9	8	15.5	11.5	10	EDI
12	974	11	14	10	7.5	7	12	9	9	EDI
13	2689	15.5	41	14	22	9	62	6	13	EDI
14	2911	16	39	13	21	9	65	6	11	EDI
15	2797	15	20	10	9	7	7	8	7	EDI
16	2625	14	17	8	8	8	7	7	6.5	EDI
17	849	11	8.5	10	16	6	7	5	7	EDI
18	934	11	8	11	18	6	5	5	7	EDI
19	1106	11	11	11	6	6	10	3	6	EDI
20	1146.5	12	9	9	6	5	10	3	7	EDI
21	13	1288.5	11	10	15	10	6	11	16	GD7
22	12	1467	10	12	17	12	8	12	17.5	GD7
23	12	1348	13	17.5	15.5	6	7	18	9	GD7
24	13	1443	11	18	16	6	7	19.5	9	GD7
25	11	1259	13	19	11	8	8	17	10	GD7
26	11	1274.5	12	18	13	7	7.5	18.5	8	GD7
27	9	1101.5	7	12.5	9	5.5	5	18	7	GD7
28	10	1152	9	14	10	4	7	20	7	GD7
29	13	253.5	11	15	17.5	10	7	10	11	GD7
30	12	310	13	17.5	17	10	8	13	13	GD7
31	7	1821.5	8	7	52	4	3	4	6	GD7
32	9	1931	8	7	57	5	3	4	7	GD7
33	17	514	9	13.5	26	8	6	8.5	13	GD7
34	17	490	10	15	27.5	9	6	8	12	GD7
35	10	427.5	8	17	12	6	6	9	7	GD7
36	9	294.5	8	15	11	4	4	7	7	GD7
37	10	1177	9.5	10	5	6	4	4	10	GD7
38	9	961	7	8	4	4	2	3	6	GD7
39	7	816	7	7	6	4	4	3	6	GD7
40	7	895	6	7	5	4	5	3	6	GD7
41	7	11	1739	6	6	5	5	6	4	MHV
42	10	11	1813.5	6	5	6	5	8	7	MHV
43	9	10	1130	7	8	5	5	3	6	MHV
44	10	10	1013.5	5	7	6	5	3	5	MHV
45	9	12	6176	13	12	10	7.5	15	11.5	MHV

46	11	13	6352	12	11	8.5	9	12	9	MHV
47	10	10	150	10	11	5	5	11	5	NEG
48	10	11	159.5	11	12	4.5	5	11	6	NEG
49	9	11	665	7	6	6	20	6	6	MHV
50	7	10	642	8	6	4.5	18	6	5	MHV
51	10	10	568.5	6	7	6	4	5	5.5	MHV
52	13	11	620	8	7	8	4.5	6	5.5	MHV
53	9	11	983.5	12	8	6	6	45	6	MHV
54	11	11	1104	11	10	7	8	47	8.5	MHV
55	11	10	403	13	509.5	6.5	7	17	7	MYC
56	12	11	444	16	500	6.5	8	19	7	MYC
57	10	10.5	1008	11	11	6	6	9	7	MHV
58	8	11	982	10.5	9	7	7	8	6	MHV
59	11	12	2627	13	11	6	14	19	9	MHV
60	9.5	14	2610	13	11	7	14	22	8	MHV
61	17	20	3385	30	31	17	13	25	17.5	MHV
62	15	19	3038	26	22	15	14	22	18	MHV
63	7	9	745	6	5	6	4	4	5	MHV
64	6.5	10	795.5	6	4	5	5	5	6	MHV
65	15	13	1118	9	18	6	6	5	7	MHV
66	18	13	1154	8	22	7	5	5	7	MHV
67	9	10	194.5	8.5	6	5	4	5	8	MHV
68	9	8	161	11	7	6	5	5	8	NEG
69	8	8	1516.5	7	6	4.5	5	6	7	MHV
70	8	10	1226	6	5.5	5	5	5	7	MHV
71	7	8	1378	6	5	5	3	3	5	MHV
72	8	9	1371	6	4	5	4	3	4	MHV
73	8	8	494	5	4	3	4	4	4	MHV
74	8	9	546	6	5	5	5	5	5	MHV
75	6	6	342	4	4	4	3	2	3	MHV
76	7	7	367	4	5	5	5.5	4	6	MHV
77	7	9	834	6	10	5	5	3	5	MHV
78	7	9	893	6	11	4	4	4	6	MHV
79	8	10	2146	16	7	4	4	4	7	MHV
80	7	8	1599	13	5	4	3	3	5	MHV
81	7	20	13	359	7	4	11	18	5	MVM
82	7	22	14	393.5	6	5	14	23	5	MVM
83	35	283	13	367.5	139	6	77	4	4	MVM
84	33	266.5	11	326	118	5	73	4	4	MVM
85	13	170	11	276	8	6	48	20.5	5	MVM
86	16	237	15	372.5	11	9	63	31	7	MVM
87	16	57	11	262	14	6	9	24	6	MVM
88	16	65	14	296.5	16	9	11	24.5	6	MVM
89	7	10	10	110	13	4	5	45	5	MVM
90	8	10	9	112	11	3	4	40	4	MVM
91	8	10	9	149	5.5	4	19.5	9	4	MVM
92	10	9	9	177	5	5	23	11	5	MVM

93	11	16	7	200	6	4	8	6	5	MVM
94	10	14	7	219	4	3	7	7	5	MVM
95	10	43	8	341	9	5	8	6	4	MVM
96	8	33.5	7	267.5	8	5.5	7.5	4	3	MVM
97	39	15	6	433	8	5	7	17	16	MVM
98	32	13	5	410.5	6	6	6	12	15	MVM
99	8	44	7	151	4	4	9	77	5	MVM
100	7	41	7.5	136	4	4	7	65	5	MVM
101	11	132	10	28	27	6	30	17	7	GD7
102	11	144	11	28	28	6	32	18	7	GD7
103	12	75.5	9	333	12	7	15	19	7	MVM
104	10.5	72	7	305	9	5	21.5	21	6	MVM
105	10	17	12	85.5	11	6	6	26	7	NEG
106	11	18	13	106.5	15	6	7	31	7	MVM
107	18	22	10	89	8	4	7	4	8	NEG
108	19	24	9	99	9	6	7	3	7	MVM
109	13	206	21.5	191	13	7	63	35	8	GD7
110	12	233	21	190.5	12	8	66.5	39.5	8.5	GD7
111	11	80	11	303.5	15.5	5	13	18	4	MVM
112	12	106	9	438	66.5	8.5	14	17.5	7	MVM
113	13	123	12	31	17	8	29.5	18	8	GD7
114	12	119	13	33	17	8	31.5	17	10	GD7
115	7	12	7	415	7	5	4	3	5	MVM
116	9	13	7	412.5	7	5	5	3	5	MVM
117	7	17	8	228	3.5	5	5.5	23	3	MVM
118	5	14	5	189	3	7	2	21.5	2	MVM
119	9	23	7	444	30.5	3.5	7	7	11	MVM
120	9	15	5	459	46	7	10	9	14	MVM
121	11	716	137	69.5	6057	7	300	163	20	MYC
122	12	748	133	73	5906.5	8	306	159	19	MYC
123	12	1195	126	98.5	4219	8	512	24	12	MYC
124	16	1688	145	126	6088	14	679	30	20	MYC
125	17	342	25	27	4809	10	93.5	6	15	MYC
126	18	373.5	28.5	34	5364	7	109	6	15	MYC
127	11	1958	284	178	5075.5	11	833	8	20	MYC
128	13	2441.5	330	202.5	5678	12	976	9	23.5	MYC
129	34.5	852	119	82	4781	20	239.5	37	22.5	MYC
130	35	834	115	80	4860.5	22	240	35	23	MYC
131	13	1466	154	100.5	7148.5	9.5	472	9	22	MYC
132	15	1865	191.5	137	8170	14	627	12	28	MYC
133	19	821	70	62	4921	11	267.5	13	17	MYC
134	19	830	69	67	4716.5	10	290	13	16	MYC
135	14	334.5	134	56	6475	10	110.5	8	20	MYC
136	15	313	131	54	6379.5	10	105	8	22	MYC
137	13	2644	79	161	10234	17	955	37	16	MYC
138	14	2791	93	185	9612	16	1002	39.5	17.5	MYC
139	24	2550	256.5	187	11764	18	837.5	29	37	MYC

140	26	2458.5	245	193.5	11497	17	838	28	38	MYC
141	26	2134	71	162.5	9517	12	705	17	15	MYC
142	26	2061	71.5	151	9784	11	667.5	16	14	MYC
143	14	27.5	9	7.5	4645	12	9	9	7.5	MYC
144	15	26	10	9	4808	10	10	12	8	MYC
145	32	296	17	22	7793	9	97	17	8	MYC
146	35	359	16	23	7712.5	11	107.5	21.5	9	MYC
147	30	2568	127	189.5	3448	26	849	57	29	MYC
148	33	2526	134	190.5	3720	27	855.5	62	24	MYC
149	20	127	117	69	4768	19	102	16	26.5	MYC
150	24	135	118	83	5800	25	116	17	36	MYC
151	17	655.5	47	72	2498	9	195	26	16	MYC
152	17	659	43	70	2518	9	197	28	17	MYC
153	26	393	44	86.5	5709	13	90.5	35	19	MYC
154	30	427	51	95	5952	14	96	35	21	MYC
155	21	2292	192.5	183	7342	16	829	44	37	MYC
156	21	2361	204.5	199	7669	19	907	47	39	MYC
157	23	14	9	13	2087	7	6	17	19	MYC
158	20	15	8	13	1974	8	5	16	16	MYC
159	19	16	59	13.5	7620	8.5	13	6.5	14	MYC
160	16	15	50.5	11	6775	8	13	6	14	MYC
161	25	26.5	13	24	16	16	12	35	18	NEG
162	26	29	12	26	18	21	11	29	20	NEG
163	14	22.5	12	13	8	10	10	12	8	NEG
164	17	24	13	16	9	13	10	17	10	NEG
165	22.5	28.5	25	72.5	79	16	77	63.5	15	NEG
166	17	23	21	43.5	49	11.5	47	45	13	NEG
167	11	17.5	9	22	10.5	7	145	7	10	REO
168	13	18	10	26	13	9	177	10	11	REO
169	18	26	22	40	19	13	16	45	16	NEG
170	20	27	20	43	23	13	14.5	55	17	NEG
171	30	20	11	11	9	14	6.5	15	11	NEG
172	38	21	12	13	9	16.5	10	22	13	NEG
173	15	24	16	16	47	12	98	39	14	NEG
174	15	24	16	17	49.5	13	84	39	13	NEG
175	14	20	11	13	36.5	13	46	11.5	14	NEG
176	15	22	11	12	40	16	48	12	15	NEG
177	22	25	87	80	71.5	15	29	129	34	NEG
178	17	21	53	52	33	10	15	67	18.5	NEG
179	25	29	30	54.5	170	17	20	97	21	NEG
180	21	26	22	38	126.5	14	14.5	68	15	NEG
181	16	19	24	18	14	11	6	39	10.5	NEG
182	14	17	22	13.5	12	10	6	32	10	NEG
183	24	26	29	19	21.5	20	19	16	21	NEG
184	21	22	21	13	18	14	12	11	17	NEG
185	13	19	22	10.5	6	7	6	13	10	NEG
186	10	17	17	8.5	6	6	5	11	8.5	NEG

187	14	18	11	13	34	10	13	93	10	NEG
188	13.5	18	11	13	38	8	12	90.5	9	NEG
189	16	21	10	9	25	16	6	10	14	NEG
190	16	22	9	8	32	17	8	10	15	NEG
191	15	18	15.5	9	9	10	9	14	10	NEG
192	13	18	14	11	9	10	8	12	10	NEG
193	14	19	15	10	8	9	6	64	9	NEG
194	13	21	17	10	9	9	7	69	9	NEG
195	12	22	17	8	18	7	29	8.5	8	NEG
196	11	21	16	8	15	6	25	7	7	NEG
197	13	17	10	14	14	7	5	24.5	8	NEG
198	10	16	10	12	13	6	5	23	7	NEG
199	12	19	9	10	6	8	6	61	7	NEG
200	11	19	9	9	6	7	5	49	7	NEG
201	14	21	9	12	8	8	5	74	14	NEG
202	12	18	8	8	6	6	5	55.5	11	NEG
203	13	24	15	36	10	11	8	7	36	NEG
204	10.5	19	12	26	7	8	7	5	19	NEG
205	9	18	24	9	7	5.5	9	5	8	NEG
206	10	17	21	8	6.5	6	8	5	7	NEG
207	9	14	10	9	5	5	5	5	8	NEG
208	10	16	10	10	6	6	6	5	8	NEG
209	19	22	14.5	26	15.5	14.5	10	11	33	NEG
210	16	20	12	24	10	11	7	9	27	NEG
211	14	20	14	20	8	11.5	65	7	16	NEG
212	12	18	13	16	8	8.5	49	7	14	NEG
213	8	16	7	6	6	5	4	7	6	NEG
214	8	15	8	6	5	6	4	5	6	NEG
215	11	15	8	12.5	6	6	4	66	7	NEG
216	11	19	8	14	7	5	5	78	7	NEG
217	11	15	9	8.5	6	6	5	34.5	7	NEG
218	12	17	9	11.5	7	7	6	50	8	NEG
219	14	22	17	25.5	14	9	9	77.5	13	NEG
220	13	20	15	20	11	9	10	60.5	12	NEG
221	12	16	16	45.5	14	7	8	27	10	NEG
222	16	19	19	69	21	9	10	48	15	NEG
223	22.5	18	16	12	9	10	6	166	11	NEG
224	25	18	18	10	9	10	6	157	10	NEG
225	16	25	11	15	9	11	5.5	77	29	NEG
226	16	23	11	18	10	11	6	85	30.5	NEG
227	14	17	12	12	216	9	5	32	15	NEG
228	17	18.5	12.5	14	281.5	10	7	37	17.5	MYC
229	19	27	11	16	12	11.5	11	21	30.5	NEG
230	23	30	12	18	14	16.5	13	27	34	NEG
231	14	20	9	11	9	8	5	20	13	NEG
232	15	20	11	15	10	9	6	24	14	NEG
233	17	25	33	24	13.5	11	8	48	30	NEG

234	17	23	31	20	12	9	8	45	26	NEG
235	26	30	13	32.5	10	13	8	49	44	NEG
236	24	27	11	26	9	12	6.5	39	35	NEG
237	19	37	13	68	40	10.5	11	9.5	19	NEG
238	18	36	13.5	77	41	10	11	10	17	NEG
239	13	21	13	12	6	7	6	7	19	NEG
240	15	23	16	12	9	10	6.5	9	30	NEG
241	12	18	13	15	10	8	17	15.5	10	NEG
242	12	14	14	15	11.5	11	25.5	16	13	NEG
243	7	11	7	5	4	4	4	6	4	NEG
244	8	11	9	5	3	4	4	6.5	6	NEG
245	9	11	8	11	20	4	4	14	9	NEG
246	8	12	9	12	19.5	5	4	13	12	NEG
247	11	13	10	11	7	6	4	10	11	NEG
248	11.5	12	10	11	7	4	4	11	9	NEG
249	12	15.5	11	17	13	9	17	37	10	NEG
250	11	15	11	16	13	9	16	29	9	NEG
251	11	15	11	17	34	6	24	20	9	NEG
252	11	13	9	14	26	5	21	18	9	NEG
253	9.5	11	10	8	6	5	5	12	6	NEG
254	10	12	9	8	5	5	4.5	14	8	NEG
255	10	11.5	9	9	8	5	5	8.5	6	NEG
256	11	13	8.5	10.5	9.5	5	5	10	7	NEG
257	12	13	13	21	13	8	36	43	11	NEG
258	10	14	12	24	14	9	41	52	12	NEG
259	8	10	9	7	12	795	3	8	5	PVM
260	8	11	8	7	12	770	4	8	6	PVM
261	12	13	12	22	13	659.5	6	18	8	PVM
262	13	13	13	20.5	16	594	7	19.5	8	PVM
263	8	11	10.5	7	7	911.5	4	3	8	PVM
264	10	13	13	8	6	1087	5	4	10	PVM
265	9	10.5	10	9	6	887.5	5	9	9	PVM
266	8	13	10	8	6	933	5	9.5	9	PVM
267	10	11	11	13.5	10	636.5	4	35.5	7	PVM
268	10.5	11	10	15	11	646.5	5	30	7	PVM
269	7	9	8	6	511	867	8	3	6	PVM
270	8	11	8	7	544	935	10	2	6	PVM
271	8	11	7	8	5	450	4	3	7	PVM
272	8	12	8	8	5.5	388	4	4	7	PVM
273	12	11	9	16	12	543	5	10	7	PVM
274	13	12	9	15	14	495	5	8	6	PVM
275	8	9	6.5	6.5	5	804	5	4	5	PVM
276	7	10	6	6	7	1033	5	3	5	PVM
277	8	11	7	8	14.5	1169	6	5	6	PVM
278	9	11	9	8	24	1281.5	6	6	7	PVM
279	8	10	7	8	6	1000	3	5	10	PVM
280	7	10	7	7	6	859.5	3	5	7	PVM

281	9	12	11	12	8	6.5	1797	9	7	REO
282	10	13	12.5	14	10	7	1918	10	9	REO
283	10	10	14	12	10	6	1411	20	7	REO
284	13	12	14	11.5	10	6	1521.5	22	9	REO
285	9	11	8	7	7	7	1134	18	7	REO
286	10	11	7	8	6	5	1124	16	6	REO
287	11	12	9	12	9	7	1848.5	10	8	REO
288	11	12	8	12	8	6	1665	9	7	REO
289	9	11	7	10	8	4	1899	11	5	REO
290	8	10	8	11	7	4	1904	11	6	REO
291	8	9	7	7	5	4	999	2	5	REO
292	7	8	6	4.5	5	4	1043	2.5	5	REO
293	9	10	7	5	4	4	1200.5	3	5	REO
294	11	9	7	5	4	3.5	1137	3	4.5	REO
295	9	9	6	7	19	5	1151.5	4	5	REO
296	9	10	7	7	20	5	1103	3	6	REO
297	8	15	55	10	60	5	1368.5	8	6	REO
298	8	14	59	10	31	7	1519.5	10	6	REO
299	7	9	77.5	7	10	4.5	1119	11	5.5	REO
300	8	9	81	8	10.5	4	1181	12	5	REO
301	8	10	7	10	7	3	1383	6	5	REO
302	8	11	7	10	7.5	4	1499	8	6	REO
303	10	75	9	10	17	5	1412	11	9	REO
304	8	56	6.5	8	14	4	1211	9	5	REO
305	8	10	9	10.5	8	5	647	6	6	REO
306	9	11	9.5	11	12	6	747.5	7	7	REO
307	8	10	12	7	5	4	691	4	5	REO
308	8	9	13	7	4	4	635.5	3	6	REO
309	8	9	8	5	3.5	3	270	2.5	5	REO
310	7	11	10	5	4	3	271	3	5	REO
311	7	10	6	5	7	4	633	3	5	REO
312	7	7.5	7	5	8	3	686	4	4	REO
313	12	12.5	8	8	5	9	729	6	6	REO
314	9	11	7	6	5.5	7	753	5	6	REO
315	7	10	6	6	7	3	832	2	4.5	REO
316	8	11	7	7	8	5	959	3	5	REO
317	6	6	5	3	3	2	526	2	2	REO
318	7	9	6	5	6	4	1373	3	5	REO
319	12	12	9	4	7	4	728	3	5	REO
320	13	13.5	10	7	26	5	758	3	5	REO
321	11	14	10	13	10	6	6	3285	8	SEN
322	10	11	8	13	11	5	4	2838	7	SEN
323	10	13	14	8	6	8	6	6994.5	8	SEN
324	11	11	14	9	7	7	5	6262	8	SEN
325	9	11	8	8	9	6	4	1363	7	SEN
326	8	9	7	6	7	4	4	1249	6	SEN
327	26	14	10	11	7	5	6	4595	8	SEN

328	27	13	11	11	7	5	6	4350	7	SEN
329	9	10	9	10	7	8	5	7394.5	10.5	SEN
330	10	13	9	10	7.5	13	6	8240	11	SEN
331	17	13	7	11	7	5	3.5	5173	6	SEN
332	12	11	8	10	6	5	4	4907	6	SEN
333	9	11	8	9	7	5	4	982	7	SEN
334	8	11	8	12	9	5	5	1061	6	SEN
335	8	10	8	7	8.5	6	12	3215.5	6.5	SEN
336	8	10	8	7.5	8	5	11	3646	6	SEN
337	10	14	11	17	12	6	6	6739	8	SEN
338	9	14	12	20.5	12	7	6	6398	8	SEN
339	8	10	11	7	6	5	4	4166	6	SEN
340	8	10	11	7	5	4	4	4364	6	SEN
341	10	10	9	20	6	7	7	5	2509	VAC
342	9	11	7	21	6	5	8	4	2517	VAC
343	9	13	12	9	4.5	5	7	3	3064.5	VAC
344	10	16	12	11	6	4	6.5	4	3224	VAC
345	10	13	10	16	7	6	5	3	2662	VAC
346	10	13	9	17	7	5	5	3	2362	VAC
347	11	15	12.5	29	30	5	7	10	3286	VAC
348	12	14	11	24.5	30	6	6	10	3401	VAC
349	8	11	8	13	7	4	4	3	3040	VAC
350	8	13	9	14	7	5	5	4	3110	VAC
351	6	11	6	6	4	4	4	2	2145	VAC
352	7	11	8	7	4	4	4	2	2131.5	VAC
353	11	14.5	8	11.5	6	5.5	5	2	4539	VAC
354	13	19	8	14	8	6	6	3	5806.5	VAC
355	8	11	9	8	6	5	4	3	2342.5	VAC
356	8	12	8	8	7	7	4	3	2502	VAC
357	8	14	7	16.5	7	7	7	5	3686	VAC
358	7	12	7	13	6	5	4	3	3208	VAC
359	12	11	7	10	4	6	5	3	4022	VAC
360	13	13	7	10	5.5	7	5	5	4475	VAC
361	12	13	16.5	14.5	5	5.5	237	153	42	REO
362	15	18	27	29	6	8	369	95	40	REO
363	708.5	14	17	15	5	4.5	15	8	7	EDI
364	12	113	19	15	5	7	16	83	27	GD7
365	14	1303	21	28	5	6	16	138	16	GD7
366	10	444	18	14.5	6	6	14	16	6	GD7
367	10	441	17	15	5	5	14.5	12	7	GD7
368	12	16	19	71	5	5	15	163	19	NEG
369	13	40	18	136	5	6	16	118	11	MVM
370	12	19	18	49	8	6	13	67	7	NEG
371	14	20	20	45	7	6	13	72	7	NEG
372	12	15	17.5	21	4	6	15	43	8	NEG
373	15	13	20	16	4	5	14	7	33	NEG
374	16	13	20	16	5	5	13	134	10	NEG

375	13	17	20	21	5	5	14	141	23	NEG
376	13	17	22	19	4	5	14.5	128	10	NEG
377	12	16	18	16	5	1097.5	15	8	12	PVM
378	12	15	18	15	5	1164	14	122	12	PVM
379	10	15	19	16	4	1454	12	164	12	PVM
380	10	13	16.5	15	4	1410	13	34	6	PVM
381	12	19	21	25.5	5	891	14	70	41	PVM
382	10	14	16	17	4	717.5	11.5	7	6.5	PVM
383	10	13	15.5	16	3	754.5	14	6	6	PVM
384	10	12	15	15	4	998.5	14	16	7	PVM
385	14	15	21	30	7	6	396	65	4	REO
386	11	15	17.5	16	5	6	101	40	6	NEG
387	12	15	19	28	5	6	351	20	8	REO
388	11	16	21	15	5	5	14	1934	11	SEN
389	11	110.5	19	15	6	6	14	78	41	GD7
390	11	14	16	21	4	6	14	45.5	8	NEG
391	2706	18	27	26	6	7	17	97	32	EDI
392	1140	18	27	30	8	9	18	165	6	EDI
393	969	15	211	20	7	7	15	104	17	EDI
394	2019	18.5	18	20	5	6	14	10	10	EDI
395	1866	15	17	18	5	5	14	9	9	EDI
396	719	14	17	16	5	5	14	8	7	EDI
397	18.5	145.5	17	19	6	8	15.5	83	38	GD7
398	15	1313	23	29	9	8	15	44	41	GD7
399	12	413	17	15	4	5	15	11	6	GD7
400	12	413.5	18	14	4	5.5	13.5	10	6	GD7
401	10.5	635	18	13	5	6	15	11	7	GD7
402	11	621	18	14	4	4	14.5	11	6	GD7
403	13	209	20	19	4	6	13	30	7	GD7
404	12	509.5	18	17	5	7	16	11	8.5	GD7
405	16	441.5	22	37	6	7	14	32	11.5	GD7
406	14	2279	17	18	5	8	17	17	8	GD7
407	13	15	249.5	15	6	6	13	123	39	MHV
408	13	13	253.5	16	5	8	15	27	11	MHV
409	15	20	143.5	27	6	8	14	165	37	NEG
410	12	16	95	22.5	4	6	14	81	8	NEG
411	11	16	325	16	6	7	14	15	9	MHV
412	14	15	19	71	6	6	15.5	163	11	NEG
413	12	39	19	128.5	4	6	14	18	32	MVM
414	11	15	16	13	6	5	14	14	7	NEG
415	10.5	14.5	17	12.5	7	6	12	16	6	NEG
416	19	24.5	28	77	8	8	18	137	44	NEG
417	15	18	24	28	8	9	16	37	11	NEG
418	14	16	20	26	6	6	17	5	25	NEG
419	14	20	23	27	7	6	14	88	17	NEG
420	12	14	17	18	5	1107	13	70	24	PVM
421	12	14	18	18	5	1246	14	75	25	PVM

422	12	15	20	19	5	932.5	15	162	25	PVM
423	13	14	19	19	4	870.5	14	152	15	PVM
424	15	16	18	21	6	1707	17	31	16	PVM
425	14	18	20	31.5	7	862	15	18	41	PVM
426	13	17	20	24	5	837	14	70	33	PVM
427	23	15	16	13	7	1049	13	24	11	PVM
428	25.5	14	16	14	7	1039	13	159	42	PVM
429	14	16.5	21	28	6	383	13	34	9	PVM
430	15	20	28	37	5	650	13	38	8	PVM
431	12	19	20.5	16	5	1562	13	8	9.5	PVM
432	11	12	16	15	5	1064	15	10	7	PVM
433	13	14	17	19	5	1055	15	12	7	PVM
434	11	13	17	24	5	1548.5	14	19.5	9	PVM
435	11	15	17	23	5	1619	17	19	7	PVM
436	10	12	16	14	4.5	1024	15.5	16	7	PVM
437	14.5	40	49	35	6	7	391.5	111	43	REO
438	14	17	25.5	29	6	6	329	149	26	REO
439	12	13	17	16	5	7	239.5	114	24	REO
440	13	11	16	12	4	4	354	23	33	REO
441	12	13	16	13	3	4	366	138	20	REO
442	12	13	18	13	4	4	143	138	42	REO
443	11	12	16	13	4	5	147	123	28	REO
444	14	17	19	16	4	6	89	8	8	NEG
445	11	14	19	16	5	5	116.5	48.5	7	NEG
446	12	15	17	14	4	6	139	22	8	REO
447	13	15.5	19	25	7	7	286.5	20	8	REO
448	14	16	18.5	28	6.5	7	568	71	10	REO
449	12	16	19	28	7	7	600	73	11	REO
450	11	13	35	22.5	6	5	260	29	8	REO
451	12	14	35	24	5	6	258.5	31	8	REO
452	13	14	20	32	6	6	361	23	7	REO
453	11	39	16.5	22	4	5	227	25	7	REO
454	12	16	20	16	5	6	14	2585	11	SEN
455	15	17	22	25	5	8	15	3935	9	SEN
456	13	16	20	21	5	5	13	4346	9	SEN
457	13	14	17	19	4.5	5	13	4088	10	SEN
458	11	16	19	14	4	5	14	1724	10.5	SEN
459	13	14	19.5	20	5	7	14	9629.5	19	SEN
460	13	17	24	36	8	6	16	6639	15	SEN
461	13	30	22	29	4	5	15	20	2850	VAC
462	24	25	22.5	45	5	6	15	9	4270	VAC
463	24	25	24.5	45	5	6	15	10	4064	VAC
464	17	29	27	20	5	6	16	14	1120.5	VAC
465	16	20.5	21.5	41	6	8	15	48.5	2767	VAC
466	13	16	17	15	4.5	6	15	11	3583	VAC
467	12	16	17	16	4	5	13	10	3634.5	VAC
468	11	13	18	16	6	8	17	11	3408	VAC

469	965	18	20	22	6	7	15	11	9	EDI
470	14	15	410	18	8	9	17	12	9	MHV
471	12	14	321	16	6	8	16	14	8	MHV
472	14	19	23	29	6	5	15	103	9	NEG
473	13	18	18	16	5	6	107	10	7	NEG
474	14	17	20	29	6	7	14	3803	10	SEN
475	10	12.5	16.5	14	5	9	18	12	3280	VAC
476	1941.5	20	20	26	11	8	18	63	14	EDI
477	797.5	20	23	33.5	8.5	8	17	112	6	EDI
478	2546	17	22	24	7	6	16	57	41	EDI
479	1786	14	18	15	5	8	15	51	24	EDI
480	987	15	195.5	20	5	6	15	122	11	EDI
481	2359.5	18	25	19	8	5.5	14	16	8	EDI
482	3563	15	17	24	5	10	20	19.5	9	EDI
483	3592.5	15	18	24	6	9	20	19	10	EDI
484	19	151	17	20	6	7	15	126	22	GD7
485	17	1368	24	33	9	8	15	99	38	GD7
486	16	132	25	27	6	7	16	151	19	GD7
487	16	1736	24	37	6	7	17	31	26	GD7
488	11	619.5	16	14	4	5	15	39	17	GD7
489	12	1534	21	17	6	7	16	9	9	GD7
490	13	1145.5	22.5	26	6	6.5	15	47	17	GD7
491	25	526	45	30	6	7	18	91	11	GD7
492	13	757	23	29	8	7	14	53	7	GD7
493	13	672	19	17	6	7	14	9.5	8	GD7
494	14	197	20	22	4	6	16	31	7	GD7
495	13.5	504.5	17	18	5	7	15	11	8	GD7
496	14	3158	18	20	6	8	17	19	8	GD7
497	16	2237	19	18	5	8	16	16	8	GD7
498	15	17.5	685	20	8	9	18	123	33	MHV
499	17	19	135	40	7.5	9	19	42	25	NEG
500	16	20	130	38	6	8	18	123	41	NEG
501	15	17	106	36	10	8	18	35	27	NEG
502	13	15	94	22	5	5.5	13	77.5	8	NEG
503	15	20	107	30.5	6	7	15	28	8	NEG
504	15	22	116	34	8	6.5	16.5	30	9	NEG
505	13	14	325	18	7	8	16	29	8.5	MHV
506	12	16	317	18	6	8	16	27	8	MHV
507	16	19	29	115	7	8	16	84	5	MVM
508	16	20	30.5	114	8	6	16	33	5	MVM
509	14	22	20	99.5	5	6	14	68	19	MVM
510	13	44	21.5	70.5	6	7	16	33	10	NEG
511	15	88	23	35	10	7	18	31.5	9	NEG
512	14	21	19	238	7	7	16	15	10	MVM
513	14	34	18.5	161	7	8	19	33	10	MVM
514	20	18	22	93	7	9	17.5	171	11	MVM
515	18	26	32	56	8	8	16	216	14	NEG

516	16	19	20	24	7	7	15	35	12	NEG
517	14	17.5	22	21	6	6	14	106	5	NEG
518	14	20	20	19	6	5	16	150	40	NEG
519	15	18	21	23	5	7	17	93	8	NEG
520	15	18	22	27	7	8.5	15	198	9	NEG
521	16	18	21	29	6	8	17	188.5	10	NEG
522	15	21.5	26	28	6	6	17	41	25	NEG
523	15	23	25	27	8	5	17	92	30	NEG
524	16	18	21	23	10	10	19	45	11	NEG
525	18	26	28	36	6	7	17	104	37	NEG
526	18	19	21	23	6	6	16	66	27	NEG
527	12	15	17	18	5	1137.5	14.5	110	4	PVM
528	12.5	15	20	19	4	1264	14	162	43	PVM
529	13	17	21	18	6	1415.5	15	63	13	PVM
530	13	17	20	17	6	1373	15.5	8	30	PVM
531	14.5	18	19	24	7	1843	17	108	18	PVM
532	14	17	22	35	6	826	16	36	11	PVM
533	15	18	24	36	6	904	15	163	35	PVM
534	14	17	21	29	7	879	15	140	40	PVM
535	12	16	19	18	6	1586	14	9	8	PVM
536	14	17	22	29	6	384	14	36	10	PVM
537	16	21	27	38	7	659	14	39	8	PVM
538	13	19	22	17	6	1588	16	8	10	PVM
539	9	12	16	16	5	1027.5	14	9	7	PVM
540	17	18	22	36	7	526	16	46	10	PVM
541	16	18	23	37	8	515.5	15.5	44	11	PVM
542	13	16	19	21	7	734	15	13	12	PVM
543	19	21	25	48	9	953	18	44	12	PVM
544	13	14	19	19	4	1042	15	12	8	PVM
545	14	40	52	34	6	6	397.5	46	27	REO
546	13	14	18	30	7	6	350	5	26	REO
547	16	21	26	32	10	11	353.5	9	31	REO
548	14	20	23	29	8	9	285.5	101	28	REO
549	16	20	25.5	38	8	9	464	66	36	REO
550	13	12	18	16	5	6	303.5	103	19	REO
551	13	13	16	16	5	5	286	28	42	REO
552	13	16	21	16	5	7	94	8	7	NEG
553	14	15	20	15	6	7	167	10	7	REO
554	20	14	18	14	5	9	114	9	8	NEG
555	21.5	14	18	14	5.5	8	111	9	7	NEG
556	12	15	17	12	5	6	181.5	10	8	REO
557	14	16	18	14	5	7	195	10	9	REO
558	15	18	28	33	6	6	147	63	9	REO
559	13	19	28	31	6	6	131	53	7	REO
560	13	50	18	16	5	7	227	53	9	REO
561	18	18	33	44	8	9	266	37.5	9	REO
562	12	14	18	24	6	7	298	18	8	REO

563	13	16	18	15	6	6	16	2908	12	SEN
564	13	18	24	27.5	9	9	17	2160	13	SEN
565	13	18	23	38	7	6	15.5	6610	17	SEN
566	13	15	19	18	5	6	17	2281	13	SEN
567	12	14	19	19	5	7	14	7076	12	SEN
568	16	18	24	46	7	8	17	9320	18	SEN
569	12	32	21	34	5	6	15	20	3039	VAC
570	17	20	20	31	7	7	14	27	217	NEG
571	14	18	21	28	9	10	15.5	78	2068	VAC
572	15	22	20	48	7	7	15	62	2778	VAC
573	12	14	21	29	7	9	16	11	988	VAC
574	14	16	20	29	7	8	19	12	1049	VAC
575	13	19	18	34	7	8	17	15	5822	VAC
576	15	20	21	38	8	8	16	177	896	VAC
577	265	19	93.5	21	8	10	22	12	10	EDI
578	2300.5	17	23	18	7	6	16	17	8	EDI
579	1106	22	26	23	6	7	16	11	10	EDI
580	15	30	20	182	7	7	17	160	26	MVM
581	15	18	21	20	9	9	20	48	11	NEG
582	13	15	18	18	5	1578.5	15.5	10	8.5	PVM
583	12	17	19	15.5	5	7	106	10	7	NEG
584	13	15	18	22	5	8	508	15	9	REO
585	17	25	27	33	6	7	17.5	8349	23	SEN
586	12	14	19	18	5	8	16	2498.5	12	SEN
587	758	19	23	34	10	9	16	101	7	EDI
588	1681	19	20	22	9	8	16	46	41	EDI
589	852	20	23	31	8	8	15	54	26	EDI
590	1817.5	14	17	15	7	7	15	39	21	EDI
591	274	19	98	22	6	8.5	20	13	10	EDI
592	762.5	19	22	29	9	9.5	16	68.5	12	EDI
593	997	18	22	23	6	7	16.5	12	10	EDI
594	1035	22	24	28.5	9	11	23	30	18	EDI
595	19	1541	27	45	9	10	17	78	33	GD7
596	15	127	24	28	6	9	15	68	32	GD7
597	15.5	181	23.5	35	7	7	16	106	18	GD7
598	12	591.5	17	14	4	6	13	67	36	GD7
599	12	1549	21	19	4	7	18	9	7	GD7
600	15	1173.5	21	28.5	5	7	15	51	19	GD7
601	23	520.5	49	34	6	7	15	85	11	GD7
602	15	1571	26	33	7	7	17	32	9	GD7
603	15	1765	26	35	8	9	17	33.5	9	GD7
604	14	781.5	24	33	9	8	15	54	8	GD7
605	15	120	23	39	6	6	16	75.5	9	GD7
606	15	3243	18	19	6	7	14	20	9	GD7
607	15	120	25	37	10	9	16	100	11	GD7
608	16	127	22	39	10	8	17	112	10	GD7
609	12	216	19	19	6	8	15	14	9	GD7

610	16	446.5	21	39	7	8	15.5	30	12	GD7
611	15	18	674.5	20	8	8	17	84	28	MHV
612	14	18	1994.5	24	9	10	19	76	17	MHV
613	15	22	151.5	26.5	7	7	17	44	36	NEG
614	16	17	56	27	7	8	16	127	16	NEG
615	13	19	150	30	6	6	16	15	9	NEG
616	15	23	180	30	11	10	17.5	16	11	MHV
617	14	16	256	20	9	9	17	14	10	MHV
618	14	17	259	21	10	11	18	13	9	MHV
619	13	16	417	18	7	9	19	13	8	MHV
620	13	14	328	16	7	7	16	14	8	MHV
621	15	27	20	189	7	8	16	111	37	MVM
622	13	46	21	74	7	7.5	16	34	10	NEG
623	19	18	24	98	7	9.5	17.5	177	12	MVM
624	23	28	28	31	7	6	18	163	41	NEG
625	17	20	23	79	14	13	17.5	34	11	NEG
626	13	17	20	16	7	9.5	16	115	8	NEG
627	13	18	20	18	7	9	18	54	35	NEG
628	22	27	38	65.5	11	9	19	234	16	SEN
629	15	23	30	22.5	6	6	17	56	10	NEG
630	16	22	27	40	7	9	16	29.5	12	NEG
631	14	16	22	26	9	7	15	89	29	NEG
632	14	16	21	26	9	9	16	145	19	NEG
633	14	18	22	23	10	9	16	53	14	NEG
634	19.5	22.5	30	29	9	11	18	96	13	NEG
635	15	21	21	19	6	9	15	81	8	NEG
636	16	20	20	19	7	8	16	78	9	NEG
637	30	21	26	29.5	11	12	17	180.5	13	NEG
638	20	19	24	20	9	10	19	120	9	NEG
639	18	24	26	42.5	7	8	16	123.5	15	NEG
640	15.5	21	24	22	11	11	16	19	29	NEG
641	16	23	24	21	10	11.5	15	53	38	NEG
642	17	26	28	39	7	8	15	156	37	NEG
643	16	20	23	23	5	6.5	14	113	30	NEG
644	16	17	20	20	9	9	20	29	11	NEG
645	16	21	26	23	10	1020	20	57	25	PVM
646	14	20	22	26	12	821	16	31.5	11	PVM
647	19	22	26	46	10	931.5	19	42.5	12	PVM
648	14	15	19	19	6	1618	16	19.5	9	PVM
649	14	19	20	16	5	6	172.5	11	8	REO
650	14	19.5	48	30	8	7	375	16	9	REO
651	14	20	43	28	8	7	328	15	8	REO
652	13	53	18	17	5	6.5	236.5	53	8	REO
653	11	14	17	14	4	5	133	22	6	REO
654	14	14	18	17	6	6	184	16	8	REO
655	13	15	18	19.5	6	8	529.5	15	10.5	REO
656	13.5	46.5	21	21	8	7	234	26.5	10	REO

657	12	16	19	17	6	6	14	2674	11	SEN
658	14	16	19	18	5	6	14	2586.5	10	SEN
659	12	14	19	16	5	5.5	14	2611	11	SEN
660	18	30	33	41	8	9	17	11360	21	SEN
661	17	20	25	25.5	7.5	10	16	9180	27	SEN
662	16	20	24	27	8	8	17	8972	24.5	SEN
663	18	19.5	24	34	8	9	16	1696	13	SEN
664	14	15	22	24	7	7	18	2785	13	SEN
665	17	19	25	42.5	7	9	17	12160	19	SEN
666	17	19	26.5	42	7	9	18	12043	18	SEN
667	12	13	18	20	6	6	14	6769	13	SEN
668	17	17	23	45	7	9	17	9353	16	SEN
669	19	20	26	29	9	8	17	1161	15	SEN
670	16.5	21	25	30	8	9	17	1078	16	SEN
671	13.5	19	20	20	5	6	15	10	4730.5	VAC
672	17	19	21	33	7	7	15	30	202	NEG
673	14	15	21	26	9	9	17	75	2067	VAC
674	16.5	22	22	32	12	11	17	53.5	2244	VAC
675	17	19	23	28	13	14	17.5	48.5	2228	VAC
676	18	18	21	36.5	6.5	9	18	58	1054	VAC
677	16	18	19	39.5	7	8	16.5	171	854	VAC
678	19	32.5	26	36	7	9	18	93	1941	VAC
679	19	36	27.5	36.5	7	7	19	99.5	1836.5	VAC
680	18	104	21	22	11	12	16	14	10	GD7
681	16	19	118	43	7	8.5	16	13	11	NEG
682	16	20	160	37	8	8	16	39.5	10	NEG
683	15	22.5	20	105	6	6	15	5	18	MVM
684	14	34.5	18	163	7	9	24	38	10	MVM
685	16	18	22	27	10	9	17	16	12	NEG
686	18	27.5	25	26.5	9	11	20	80	14	NEG
687	19	19.5	24	28	10	1301.5	17.5	64	14	PVM
688	14	17	19	23	8	746	16	12	11	PVM
689	17	22	28	36	8	7	560.5	15	43	REO
690	20	18	20	33	12	13	684	66	10	REO
691	14	20	20	23	8	8	16	12	5310	VAC
692	725	20	23	33	9	6	15	141	18	EDI
693	1525	18	21	22	6	9	16	138	22	EDI
694	1364.5	20	32	34	11	10	20	120	22	EDI
695	690	19	23	27	10	9	19	67	12	EDI
696	1114.5	21	26	22	6.5	9	16	11	12	EDI
697	1187	19	26	25	9	9	18	22	14	EDI
698	1137.5	20	22	24	8	8	17	22	11	EDI
699	476	17	22	41	7	9	16	56	13	EDI
700	1094	20.5	23	30.5	8	9	22	30.5	19	EDI
701	21	1591	27	47	8	10	18	140	35	GD7
702	18	195	24	39	9	8	15	67	13	GD7
703	19	1007	24	29	5	8	16	20	42	GD7

704	17	1154.5	23	27	10	10	18	112	15	GD7
705	13	652	18	18	7.5	7.5	15	9.5	8.5	GD7
706	13	196	17	19	5	6	13	13	8	GD7
707	18	101	22	22	10	12	16.5	13	9	GD7
708	15	19	2057	25	11	11	19	157	22	MHV
709	21	26	219	50.5	14	12	22	42	7	MHV
710	19	20	229.5	25	9	10	28	40	17	MHV
711	17.5	20	107.5	35	11	9	19	88	22	NEG
712	15	17	109	44	7	9	17	13	11	NEG
713	13.5	18	156.5	33	7	8	17	15	10	NEG
714	15	27	159.5	28	11	9	18.5	16	10	NEG
715	12	15	353	16	8	8	17.5	17	10	MHV
716	15.5	95	25	38	11	7	17	32.5	9	GD7
717	26	24	24	74	9	10	18	13	16	NEG
718	25	22.5	21	74.5	10	9	17	12	14	NEG
719	14	20	19	243	7	7	17	15	9	MVM
720	27	24	30	32	10	10.5	18	117	16	NEG
721	29	35	41	53	8	11	21	155	32	NEG
722	19	22	33	73	13	11	20	30	14	NEG
723	17	20	26	80	13	13.5	19	33	12	NEG
724	17	25	23	33.5	11	14	19	54.5	13	NEG
725	14	19	22	17	30	10	17	123	14	NEG
726	22	28	42	32.5	9	10	17	97	25	NEG
727	19	26	33	42	12	10	19.5	95	9	NEG
728	22	21	27.5	60	7	10	17	104	12	NEG
729	15	21.5	32	22	4	7	17	47.5	10	NEG
730	18	27	27	78.5	7	8	19	115	16	NEG
731	18	21	29	38.5	7	7	17	28	11	NEG
732	23	19	21	19	8	10	18.5	200	13	NEG
733	17	18	26	90	8	10	17.5	24	13	NEG
734	15	18	22	24	11	11	16	50.5	14	NEG
735	19	25	28	30	9.5	13	19	45	10	NEG
736	31	24	27	31	10	12	20	187	14	NEG
737	19	19	26	25	13	14	19	24	18.5	NEG
738	17	19	25.5	26	15	10.5	17.5	26	18	NEG
739	20.5	29	35	59	13	8.5	18	92	30	NEG
740	19	18	31	46	11	12	17	39	15	NEG
741	19	19.5	32	45	13	13.5	17	40	14	NEG
742	16	18	23.5	27	9	9	18	16	11	NEG
743	19	28	25	25.5	9	11	24	79	15	NEG
744	17	19	21	20	11	11	20	29.5	11	NEG
745	16	20	25	25.5	16	937	18	37	12	PVM
746	17	20	24	34	11	1372	18	67	10	PVM
747	16.5	22	27	38	10	1493	19	75	13	PVM
748	20	23	28	40	8	1137.5	19	67.5	11	PVM
749	20	24	29	36	10	1172	20	68.5	12	PVM
750	19	21	27.5	38	9	1730.5	18	108	11	PVM

751	14	17	19	20	6	1620	17	22	9	PVM
752	13	15	18	18	5	1366	16	26	8	PVM
753	18	25	29	38	9	1141	24.5	139	14	PVM
754	18	21	26	31	9	1209	24	132	13.5	PVM
755	20	26	30	53	16	14	697	25	10	REO
756	19	19	25	37	8	7	569	143	10	REO
757	15	22	25	17	5	6	288	12	9	REO
758	15	20	23	16	5	6	319	10	8	REO
759	17	19	20	16.5	6	7	159	13	12	REO
760	16	17	19	16	6	8	154	15	11	REO
761	14	15	17	18	5	7	191	19	8	REO
762	18	20	34	45	8	11	250.5	38	9.5	REO
763	12.5	16	20	17.5	6.5	7	14	2966	12	SEN
764	13	17	27	25	9.5	9	18	1981.5	14	SEN
765	13	14	19	20	4	6	13	8786	17	SEN
766	16	24	26	33	5	7	18	8415	18.5	SEN
767	18	21	25	34	8	9	18	1796	15	SEN
768	14	21	23	24	6	5	16	9	4598	VAC
769	14	21	20	25	7	7	14	11.5	4996	VAC
770	17	28	29	34	7	9	18	30	615.5	VAC
771	15	28	28	36	8	9	17	34	704	VAC
772	18	29	29	20	5	7	18	12	1109	VAC
773	19	20	19.5	30	7	13	18	22	4773	VAC
774	18	19	27	48	10	11	20	61.5	3705.5	VAC
775	19	22	28.5	47	11.5	13	18	123	1252	VAC
776	18	19	20	31	7	9	17.5	52.5	1034.5	VAC
777	15	1950	21	23	7	8	17	12	10	GD7
778	27	31	33	31	10	10	18	78	24	NEG
779	20	25	111	31	11	9	19.5	66	14	NEG
780	16	19	22	32	12	815	19	90	13	PVM
781	16	20	30	24	13	11	19	8617	26	SEN
782	14	18.5	19	33	7	7	17	15	5559	VAC
783	1536	18	20	21	6	8	17	117	29	EDI
784	1226	26	27	39	11	13	21	148	37	EDI
785	678	28	32	61.5	10	14	25	121	42	EDI
786	618	25	31	58	12	14	24	125	24	EDI
787	474	16	23	46	8	8	17	62	12	EDI
788	19	1004.5	24	30	6	7	14	30	18	GD7
789	18	127	26	48	7	8	18	86	9	GD7
790	15.5	2036	21	25	7	9	17	12	9	GD7
791	18	23	228	48.5	15	9	21	107	28	MHV
792	17	25	212	45.5	9	10	18	49	12	MHV
793	20	19	247	25	12	12	17	20	14	MHV
794	14	17	562.5	19	9	11	19	21	13	MHV
795	17	21.5	133.5	42	10	11	18	111.5	13	NEG
796	21	22	126	46	11	10.5	19	110	13.5	NEG
797	18	274	36	132	8	9	27	12	16	GD7

798	36	77.5	37	69	185	14	288	27	16	REO
799	29	22	28	28	11	10	18	112	15	NEG
800	27	41	41	53.5	8	12	21	142	28	NEG
801	21	26	32	70	13	9	21	27	14	NEG
802	22	27	28	37	14	16	23	56	16	NEG
803	44	21	23	22	10	12	21	28	13	NEG
804	45	21	23	23	10	11	17	27	15	NEG
805	17	26	23	35	12	13	18	23	13	NEG
806	22	27	43	32	7	10	18	164	8	NEG
807	18	27	32	41	10	9	16	107	14	NEG
808	19.5	25	32	97.5	12	11	17	81	13.5	MVM
809	20	26.5	32	100	13	11.5	19	81.5	14	MVM
810	21	20	27	60	8	10.5	16.5	141	39	NEG
811	22.5	34	33	89	10	12	20	341.5	21	SEN
812	15	18	22	29	11	11	19	16	14	NEG
813	17	18	27	90	9	10	17	25.5	13	NEG
814	18	18	25	56	9	10	16	30	12	NEG
815	17	19	24	53	7	9	17	33	12	NEG
816	17	20	23.5	22	10.5	11	18	46	14	NEG
817	21	24	30	50	9	9	16	135	14	NEG
818	21	21	27	52	8	11	20	112	16	NEG
819	20.5	19	26	56	8	13	20	112	16	NEG
820	20	30	34	57	12	9	17	138	29	NEG
821	20	25	115	31	9	11	18	65	15	NEG
822	15.5	18.5	22	18.5	9	10	17	23	10	NEG
823	18	19	28	49	8	826	17	76	14	PVM
824	18.5	20	27	47	8	788	18	131	20	PVM
825	15	18.5	27	24	9	991	21.5	142	20	PVM
826	18	21	23	28	10	1313	19	60	14	PVM
827	21.5	23	26	41	9	1695	17	106	11	PVM
828	17	18	21	20	9	1960	18	15	10	PVM
829	17	19	21	34	12	785	20	91.5	15	PVM
830	14	18	19	17	5	1430	16	25	8	PVM
831	21	24	29	43	12	11	526	12	30	REO
832	20	18	22	33	11	15	647.5	66.5	9	REO
833	20	23	28	39	11	12	954.5	75.5	16	REO
834	15	16	20	19.5	6	7.5	16	5900.5	14	SEN
835	19	20	28	30	8	10	19	9905.5	27	SEN
836	18	21	25	30	9	10.5	19	10076	23	SEN
837	16	19	30	25	13	12	20	8843.5	26.5	SEN
838	18	20	20	31	7	10	17	22	4854	VAC
839	20	20	25	46	10	11	20	63	3488.5	VAC
840	21	22	27	48	11	12	18	126	1166	VAC
841	22	17	20	21	7	10	18	22	6147	VAC
842	2491	19	39	33	10	12	18	29	36	EDI
843	1615	20	25	26	10	12	19	27	20	EDI
844	21	20	209	20	8.5	12	28	166	37	MHV

845	16	19	752	21	10.5	11	18	24	17	MHV
846	19	23	149.5	33.5	9	10	16	66	12	NEG
847	22	23	32	41.5	11	10	21	52	10	NEG
848	13	17	20	18	9	9	15	22	11	NEG
849	20	26	30	52	15	13	720	66	28	REO
850	21	50	36	56	6	9	18	105	3142	VAC
851	38	34	33	39	7	9	18	15	5331	VAC
852	1264	23	25	36	11	14	20	39	14	EDI
853	1346.5	19	179.5	34	9	10	21	14	15	EDI
854	1666.5	18	35	53	9	8	18.5	18	11	EDI
855	1736.5	19	34.5	57	8	10	17	19	10	EDI
856	2103.5	27	30	45	17	15	21.5	157	16.5	EDI
857	1557	20	25.5	24	10	10	18	28.5	21	EDI
858	1397	24	27	44	15	14	21	100.5	18	EDI
859	937	20	22	31	8	10	21	21	13	EDI
860	1272	16	19	23	9	13	19	17	11	EDI
861	1279.5	16	18	23	10	13	16	18	10	EDI
862	18	1165.5	22	28	10	10	18	47	7	GD7
863	20	24	118	34	15.5	17.5	26.5	23	42	NEG
864	15	19	528	26	10	10.5	18	53	13	MHV
865	18	24	660	30	15	19.5	26	26	19	MHV
866	21	17	237	24	12	11.5	18	20	14	MHV
867	15	17	740	20	11	11	18	23	18	MHV
868	17	276	38	136	9	9	27	123	10	GD7
869	24	29	38	41	17	15	21	610.5	18	SEN
870	21	27	34	34	13	13	20	140	15	NEG
871	18	22	28	25	12	14	25	75	19	NEG
872	20	21.5	24	39	12	13	18	29	14	NEG
873	29	46	42	57	12	12	23	77	16	NEG
874	22	32	34	86	9	11	20	348	21	SEN
875	16	19	24	31	11	11	17.5	16	15	NEG
876	23	18	22	22	7	9	19	199	12	NEG
877	20	19	21	21	8	8	20	370	10	SEN
878	27	36	42	64	13	10.5	24	16	34	NEG
879	24	21	26	23	10	12	21.5	78	5	NEG
880	17	19	24	22.5	10	11	18	43.5	14	NEG
881	21	33	33.5	26	10	10	19	54	43	NEG
882	20	33	31.5	25	9	10	18	61	8	NEG
883	25	23	29	42	9	1144	22	47.5	13	PVM
884	17	18	22	20	9	1864	18	14	10	PVM
885	20.5	22.5	29.5	43	12	12	500	73	29	REO
886	21.5	20	24	39	9	9	684	147	42	REO
887	16.5	18	24	22	8	10	288	22	10	REO
888	17	18	21.5	19	8	10	303	21	9	REO
889	18	18	22	23	11	13	177	22	10	REO
890	22	19	28	29	8	10	19	8225.5	22	SEN
891	20	21	29.5	50	8	9	18	8075	19	SEN

892	19	22	28	50	9	10	20	8445	20	SEN
893	22	52	38	57	8	10	19	110	3586	VAC
894	22	20	19	25	7	9	18	23	6206	VAC
895	2557	22	37	31	11	11	19	125	18	EDI
896	2078	24.5	28	47	16	14	22	165.5	15	EDI
897	18	264	21	27	9	10	17	18	11	GD7
898	62	35	31	111	1218.5	21	54	35	44	MYC
899	36	77.5	37	63	193.5	14	323.5	26	17	REO
900	23	24	27	38	16	967	22	177.5	18.5	PVM
901	21	20	26	27	8	9	19	7937	20	SEN
902	23	26	22	38	10	11	18	17.5	7952	VAC
903	1341.5	18	185	34	9	9	19	12	16	EDI
904	633	28	33	55	12	12	21	63	14	EDI
905	275.5	21	26	65	6	11	18	9	8	EDI
906	2629	20	25	35	10	13	23	15	13	EDI
907	2697	19	23	36	10	13	22	15	14	EDI
908	1355.5	25	26	44	12	15.5	22	104	19	EDI
909	2047	20	531	43	11	12	21	43	26	EDI
910	17	271.5	24	26	8	9	16	17	10	GD7
911	19	21	287.5	26	12	14	21	27	38	MHV
912	18	23	282	26	12	13	20	110	29	MHV
913	15	22	571	27	11	11	20	49	13	MHV
914	17	23	677	29	12	19.5	23.5	25	18	MHV
915	35	28.5	288	32.5	11	13	21	23	13	MHV
916	14	16	574	20	10	8.5	17	23	11	MHV
917	18	17	325.5	20	8	10	19	50	11	MHV
918	18	18	311	19	8	11	18	45	11	MHV
919	20.5	48.5	30.5	134	9	10	18	112	22	MVM
920	20	65	32	170	11	11	20	22	14.5	MVM
921	18	59	30	152	12	10	21	21	13	MVM
922	18	191	33	251	9	9	25	38	11	MVM
923	53	128	29	304.5	10	17	27	22.5	21	MVM
924	61	34	32	107	1133	22	57	37	18	MYC
925	55.5	36.5	25	25	1701	33	35	21	17	MYC
926	23	27	35	38.5	13	12	22	137	14	NEG
927	14	20	21	18	35	11	18	30	10	NEG
928	18	24	25	24	12	11	23	13	19	NEG
929	21	32	34	33	9	9	18	13	12	NEG
930	21	30	30	29	8	10	18	13	10	NEG
931	16	27	23	35	12	12.5	19	24.5	12	NEG
932	17	24	28	25.5	9	12	16	13	12	NEG
933	29	46	43	57	10	10.5	22	67.5	16	NEG
934	26	27	33	47	12	14	21	116	42	NEG
935	19	19	35	32	10	12	21	62.5	18	NEG
936	29	40	45	63	14	14	26	13	21	NEG
937	22	23	27.5	36	14	1501	22	63	18	PVM
938	21	23	30	36	13	1438	21	59	19	PVM

939	24	24	29	55	9	1555	22	38	12	PVM
940	23	24	27	40	15	969	20	179.5	17	PVM
941	20	23	29	42	12	14	943	74	16	REO
942	22.5	34	40	52	10	9	18	13264	25	SEN
943	15	16	19	19	7	7	15	5660.5	13	SEN
944	15	15.5	21	25	7	8	15	2755	11	SEN
945	31	29	31	60.5	14	14	21	79	4297	VAC
946	37	34	30.5	42	6	8	18	16	5272.5	VAC
947	19	24	150	36	9	10	19	62	12	NEG
948	26	56.5	36	156	11	11	22	80	42	MVM
949	41	25	37	41	16	18	21	169	19	NEG
950	39	22.5	39	100	10	12	19.5	20.5	18	MVM
951	20	22	27	36	16	16	21	24	19	NEG
952	680	28	34	49	13	12	20	66	13	EDI
953	271	20	27	62	6	11	17	9	9	EDI
954	1644.5	30	34	55.5	14	17	23.5	316	18	EDI
955	3343	24	44.5	34	19.5	23.5	27	33	22	EDI
956	910	18	23	33	8	10	19	20	11	EDI
957	1694	21	33	25	18	15	24	45	15	EDI
958	1801	19	37	26	14.5	19	23	44	14	EDI
959	2004	19	538	46	13	10.5	20	44	29	EDI
960	21	23.5	121.5	31	16	18	25.5	19	41	NEG
961	22	212	35	257.5	10	11	31.5	38	12	MVM
962	67	45	30	53.5	837.5	24	41	62	14	MYC
963	64	45.5	29.5	50	740	23	38	23	18	MYC
964	71	37	34	48	246.5	24	46	100.5	56	MYC
965	68	36	35.5	43	232.5	23	52	92	56	MYC
966	24	30	36	44	16	17	22.5	651	18	SEN
967	18	43	24	26	11	14	17	31	15	NEG
968	18	43	26	25	11	12	21	30	16	NEG
969	18	26	30.5	28	9	10	18.5	15	12	NEG
970	28	28	33	100	17	19	26	320	22	SEN
971	33	20	21	24	14	30	19	20	9	NEG
972	17	21	31	31	10	10	19.5	18	17	NEG
973	40	21	38	102	10	12	20	20	16	MVM
974	22	22	25	45	11	13	22	33	15	NEG
975	21	21	25	41	10.5	14	20	33	16.5	NEG
976	19	21	24	21	9	8	20	376	10.5	SEN
977	21	31	42	35	12	12	23	30	16	NEG
978	23	26	29	47	14	15	306	54	18	REO
979	29	23	24	24	12	12	343	24	13	REO
980	33	30	31	62	14	14	21	81	4618	VAC
981	28	35	34	97	7	10	18	17	9151	VAC
982	24	31	32	90	8	11	17	15	8805	VAC
983	21	25	21	36	10	10	18	18	8367	VAC
984	20	18	33	30	12	12	20	58	22	NEG
985	21	29	32	48	13	14	20	22	15	NEG

986	1581	29	31	56	15	17	25	312	16.5	EDI
987	37	27	297	35	12	12	19	23	14	MHV
988	51	129	30	279	11	17	28	22	19	MVM
989	25	206	45.5	69	12	16	35	78	17	GD7
990	22	32	26	275	10	14	21	49	27	MVM
991	22	31	30	278.5	9	12	22	49	26	MVM
992	22	530	51	50	3523.5	10	42.5	18	18	MYC
993	53	36	26	26	1694	30	31	23	17	MYC
994	45	26	40	42	18	17	23	184	20	NEG
995	25	27	30.5	58	23	17	25	35	16	NEG
996	19.5	21	23	41	10	13	18	30.5	13	NEG
997	18	23	28	41	9	9	18	69	36	NEG
998	28	28	31	34	19	20	21	57	30	NEG
999	33	19	19.5	23	14	24	19	20	9	NEG
1000	20	30	41	34	11	13	22	29	15	NEG
1001	26.5	30	34	48	12	1430	23	112	15	PVM
1002	24	25	31	50	11	1553.5	20	35	12	PVM
1003	19	19	23	24	11	12	182	23	10	REO
1004	28	22	23	26	10	12	307	22	13	REO
1005	23	18	21	22	12	14.5	161	19	14	REO
1006	18.5	19	24	26	10	11	19	10573	31	SEN
1007	18	23	29	40	10	10	17	34	29	NEG
1008	189	24	31	27	19	32	23	102	20	EDI
1009	26	24	31	45.5	10	1157.5	22	46	13	PVM
1010	3156	23	41.5	31.5	19	18	26	33	22	EDI
1011	3385.5	22	32	26	11	13	20	27	11	EDI
1012	3497	24	34	27	15	17	20	27	13	EDI
1013	37	22	39	264.5	22	14	22	46	35	MVM
1014	26	23	32	31.5	19	21	21	89	6	NEG
1015	24.5	26	41	120	19	15	25	105	26	MVM
1016	22	26	39.5	122	20	13	23.5	98	27.5	MVM
1017	24	32	33	50	14	13	23	22	18	NEG
1018	19	22	27	37	14.5	15	20	21	22	NEG
1019	25	26	30	43	16	17	300.5	55	20	REO
1020	18	19	24	26	10	11	21	10768	34.5	SEN
1021	28	21.5	371.5	28	14	18	23	60	15	MHV
1022	24	186	45	68.5	12	15	31	74	18	GD7
1023	1761	24	26	35	12	17	27	20	15	EDI
1024	1721	25	24	35	12	15	26	18	15	EDI
1025	29	21	352	28	14	16	23.5	60.5	15	MHV
1026	35	427	54	53.5	801	15	39	147	6	MYC
1027	24	525.5	54	49	3591	11	40	19	18	MYC
1028	28.5	28	37	100	17	19	28	334	22	SEN
1029	22	29.5	46	97	27	17	24	61	31	MVM
1030	18	23	33.5	34	12	13	22	22	20	NEG
1031	34	33.5	39	43	15	31	24	88	20	NEG
1032	29.5	37	41	62	19	17	25	18	44	NEG

1033	173	24	29	28	19	34	22	102	20	EDI
1034	26	28	33	48	13	1310.5	25	108	16	PVM
1035	527	52	45	86	42	32	29	96	36	EDI
1036	517	53	45	79.5	40.5	31	29	88	29	EDI
1037	24	353	43	214	11	12	30	132	34	GD7
1038	23	356	43	207	10	11.5	30.5	97	8	GD7
1039	38	24	39.5	275	23	14	21	50	32	MVM
1040	18	262	39	195	9	11	29.5	33	9	GD7
1041	35	457	55	58	841	14.5	44	129	16	MYC
1042	70	35	64	46	2741	29.5	29	52	54	MYC
1043	31.5	33	57.5	155	9	24	32	79	34	MVM
1044	22	28.5	44	98	26	14	23	56	36	MVM
1045	26	31	43	57.5	20	17	27.5	102	31	NEG
1046	3220	36	37	75	20	25	24	126	27	EDI
1047	3230	33	38	74.5	19	21	24	60	31	EDI
1048	19	254	36	196	9	11	28	37.5	10.5	GD7
1049	30	33	59.5	151.5	9	18	32	144	42	MVM
1050	31	406.5	44	76	13	17	76.5	82	13	GD7
1051	31	407	43	72	12	17	82	75	12	GD7
1052	27	33	45	60	21	18	28	99.5	30	NEG
1053	35	35	38	42	17.5	32	26.5	87	22	NEG
1054	35	35	55	78	27	20	30	115	40	NEG
1055	46	33	34	23.5	19	34	24	47	15.5	NEG
1056	31	24	31	36	16	18	26	47.5	24	NEG
1057	30	24.5	32	36	17.5	20	27	54	22.5	NEG
1058	43	395	41	232	12	12	36.5	127	17	GD7
1059	32	39	43	67	21	17	26	18	40	NEG
1060	78	37.5	63	48	2616	33	29	54	60	MYC
1061	33.5	34	47	56	28	21	29	83	36	NEG
1062	33	34	50	80	24	17	29	115	37	NEG
1063	48	34	40	26	21	36	27	50.5	18	NEG
1064	38	32	508	58	24	24	27	115	24	MHV
1065	30	33	36	48	22	25	25	46	22.5	NEG
1066	35.75	41.85	42.395	82.36	29.21	21.42	38	44.45	21.12	NEG
1067	43	406	45	236	11	14	33	25	24	GD7
1068	97.5	492.5	72	193	1178	27.5	51	28.5	37	MYC
1069	84	540.5	53	60	2396	26	59	85	24	MYC
1070	81	528	53	60	2399	25	57	84	24	MYC
1071	27	35	72	135	33	15	30.5	123.5	49	MVM
1072	29	36	74	137	33.5	17	32	119	54.5	MVM
1073	32	33	35	49	20	26	28.5	45	21.5	NEG
1074	38	32	516	56	22	23	30	118	23	MHV
1075	36	516.5	61	97	15	21	60	80	19	GD7
1076	38	242	124	61	1905.5	20	35	22	30.5	MYC
1077	29	29	45	46	30	24	32	50	37	NEG
1078	32.5	330	44	207	14	16	29.5	95	10	GD7
1079	32	335.5	44	220	14	17	34.5	103	11.5	GD7

1080	35	499	60	97	15	20.5	67	84	17	GD7
1081	56	679	69	83	1184.5	24	66	105	30	MYC
1082	71	458.5	71	144	1533	35	810	112	73	MYC
1083	34	37.5	48.5	58.5	28	25	31	83.5	37	NEG
1084	28	29	44	44	30	27	30	49	38	NEG
1085	35	784	66	137	20	27	147	28	13	GD7
1086	62	687.5	72	84.5	1292	26	69.5	77	32	MYC
1087	39	255.5	129	66	1912.5	19	38	25	30.5	MYC
1088	137	481.5	72	181	1190.5	41	53	31.5	34.5	MYC
1089	71	466	71	145	1526	32	776.5	114	77.5	MYC
1090	34	789	65	145.5	19	28	146.5	28	16	GD7
1091	35	811	127	78	2511	15	82.5	25	42	MYC
1092	51	244	59	85	15	22	43.5	123	34	GD7
1093	40	586	70	220.5	24	27	57	282	28	GD7
1094	36.5	786	121	80	2407	16	71	24	45	MYC
1095	38	550.5	68	225.5	24	28	53	294	25	GD7
1096	30	1063	86	104	15	24	76	97	10	GD7
1097	48	225	58	78	15	23	36	34	33	GD7
1098	57.5	69.5	101	179.5	20	32	34	259	83.5	SEN
1099	30	943.5	128	100	1722	17	87.5	28	26	MYC
1100	69	180.5	90.5	93	2184	50	81.5	111.5	95	MYC
1101	48	38	58	453.5	31	32	38	38	36	MVM
1102	67	200.5	90	88	1892.5	41	86	106	88.5	MYC
1103	56	74	107	175.5	19	41	38	266.5	80	SEN
1104	123	540	106	310.5	1708.5	44	553	227.5	89	MYC
1105	33	961	135	101	1666	14.5	91	87	14	MYC
1106	117	541	106	304.5	1830	45	538	221	82.5	MYC
1107	61	52.5	111.5	241	65	54	70	149	79.5	MVM
1108	46	38	62	426	30	35	37	38	37	MVM
1109	30	1068	85	106	16	25	83	147	44	GD7
1110	101	985	115	173	1406	46	122	153	24	MYC
1111	101	992	116	165	1402	46	118	147	43	MYC
1112	42	1185	161	107.5	2133	27	89	16	39	MYC
1113	61	50	108	243	60	54.5	86	149.5	77	MVM
1114	39	1171	161	98	2028	22	90.5	26	33	MYC
1115	86	1759.5	127.5	138.5	218	32	128	52	27	GD7
1116	77.5	1757.5	126	139	246	34.5	131	51.5	28	GD7
1117	26.5	1502	177	126	2039	17	103	30	4	MYC
1118	84	1269	117	218	298.5	32	284.5	168	60	GD7
1119	35	1616	180	144	2168.5	17	110	30	41	MYC
1120	79	1241	117	218.5	298	31	296	156	59	GD7
1121	72.5	2143	227.5	201	4223.5	30	166	110.5	73	MYC
1122	48	1871	274	185	960.5	32	128	157	21	GD7
1123	26	2160	154	160	2667	19	153	101	23	MYC
1124	75	2103	217	199	4036.5	30	154.5	103	71	MYC
1125	27	2237	157	161.5	2700	19	163	94	26	MYC
1126	60	1610	197	149	2866.5	27	119	46	53.5	MYC

1127	44	1930	211.5	169	1958	26	147	163	35	MYC
1128	50	1914.5	268	190.5	977	32	136	47	11	GD7
1129	58	1625	207	144	2943.5	27	127	44	53	MYC
1130	47.5	1868.5	213	173	2060	26	140.5	104	13	MYC
1131	67	1866	120	173	38	51	148.5	76	27	GD7
1132	142	500	92	132	65.5	116	86.5	284.5	25	GD7
1133	160	1916	168	250	1337	62	199	164	54	GD7
1134	170.5	1972	174	261.5	1430.5	64	188	163	57.5	GD7
1135	23	2435	240	187.5	1163.5	17	159	51	33	GD7
1136	157	553.5	99	135	79	103.5	91	309.5	28	GD7
1137	25.5	2588	266	206	1243.5	18	164	107	27	GD7
1138	265	2400	165	331	3085	81	1370	110.5	77.5	MYC
1139	51	3111	196.5	234	173	34	233	84	14	GD7
1140	240	2474	180	365	3221	70	1533	122	81	MYC
1141	46	2988	249	252	273	24	201	81	75.5	GD7
1142	53	3367.5	218.5	269	191.5	38	233	86	23	GD7
1143	62	2659	253	277	1498.5	47	218	215	119.5	GD7
1144	57	2107	408	217	1282	25	170	83	108.5	GD7
1145	50	3057.5	261	273.5	293.5	26	198.5	82	85.5	GD7
1146	84	3135.5	341	304	976	49	447.5	138	57	GD7
1147	84	3294.5	322	303.5	898	49	366	143.5	60.5	GD7
1148	68	2804	260	276.5	1511	46	218	199.5	100.5	GD7
1149	59	2197	415.5	241	1413	27	193.5	106	107	GD7
1150	99	3908	322	399	151	61	1437.5	198	41	GD7
1151	115	3660.5	268	365	3643	44	369.5	120	48	GD7
1152	113.5	3837	288.5	360	3874.5	40	380.5	121	49	MYC
1153	107	4145	277	357	908	74	454	266	117	GD7
1154	88	3673.5	311.5	408	152	57	1285	187	42	GD7
1155	114	4229.5	278	367.5	918.5	74	599	261	119.5	GD7
1156	62	52	417	434	28	108	50.5	91	15	MVM
1157	62	51	394	447	32.5	113.5	49	154	20	MVM
1158	392	4244	701	907	1581	223	3431	230.5	313.5	GD7
1159	368	4248	714	892	1854	215	3364.5	242	328	GD7
1160	359	6092.5	702.5	686	938.5	407	888	521.5	173	GD7
1161	367	6403.5	711	705.5	915	425	938	492	195	GD7

Table S3: Testing Set

Test	EDI	GD7	MHV	MMV	MYC	PVM	REO	ECTRO	SEN
1	3	3	3	2	3	6	1	2	3
2	5	1	3	2	1	3	6	3	1
3	3	3	1	2	4	4	1	2	3
4	4	1	7	4	5	4	3	4	2
5	3	2	3	7	3	1	1	4	3
6	4	3	2	4	7	2	4	5	5
7	4	2	2	4	5	0	5	4	7
8	7	3	2	3	6	3	5	4	3
9	5	7	1	5	7	4	6	5	4
10	2	3	1	4	3	6	7	3	6
11	4	4	4	4	1	5	7	1	6
12	2	4	5	5	5	6	8	5	6
13	0	4	2	2	3	6	3	6	3
14	6	5	2	4	5	1	2	3	3
15	2	1	1	2	4	1	5	4	2
16	4	1	3	3	1	2	2	2	1
17	9	5	9	11	6	4	4	8	5
18	0	2	3	1	6	7	0	3	3
19	4	3	3	4	2	5	4	4	10
20	15	16	10	4	16	3	3	11	3
21	13	19	12	5	23.5	3	2	12	3
22	8	8	8	6	4	3	10	5	2
23	19	31	17	13	22	4	10	15.5	13
24	16	21	12	5	23	4	3	14	3
25	17	18	11	5	21	3	3	14	3
26	19	24	13	4	25	4	3	14	4
27	12	17	10	3	18.5	3	2	10	2
28	25	28	17	5	31	4	3	17	5
29	21	25	15	5	29.5	3	4	16	4.5
30	18	21	14	5	23	4	2.5	12	3
31	4	3	3	3	3	1	2	4	1
32	4	4	3	3	3.5	1	2	4	1
33	6	8.5	6	3	7	2	3	7	1
34	7	9	5	4	8	3	5	6	2
35	3	4	3.5	3	5	1	2	5	0
36	6	6	4	3	5	2	3	6	1
37	4	5	4	3	4	1	2	4	1
38	4	5	3	4	4	1	2	5	1
39	3	3	2	3	2	1	2	5	1
40	14	15	11	4	18	3	3	11	3
41	14	15	11	4	18	3	3	11	3
42	14	15	11	4	17	3	2	11	4
43	13	16	11.5	3	18	3	2	11	3
44	15	18	12	4	22	3	2	11	3
45	31	30	19	6	35	4	4	18	6

46	16.5	31	17	17	23	10.5	7	13	9.5
47	14	18	12	4	22	4	2	12	3
48	14	19	11	6	20	4	6	11	3
49	16	20	13	10	21	4	5	13	4
50	23	27	16	5	32	4	3	17	4.5
51	25	28.5	17	7	32	4	5.5	15	5
52	15	19	14	5	23	4	5	13	4
53	26	29	19	6	33	5	4	18	5
54	10	6	7	7	6	4	2	6	6
55	17	21	12	5	23.5	4	4	11	4
56	20	22	15	5	26	4	6	14	4
57	16	16	11	5	17	3.5	4.5	11	3
58	15	17	11	6	20	4	5	12	5.5
59	16	19	14	5	22.5	4	3	12	3
60	17	22	13	6	23	3	6	13	4
61	5	5	3	5.5	4	4	2	4	2
62	18	23	15	5	24	3	3	13	4
63	5	5	3	4	4	2	8	5	1
64	8	5	4	5	7	2	3	6	2
65	6	6	4	3	5	2	2	5	1
66	7	7	5	6	6	3	4	5.5	3
67	8	7	6	6	7	3	4.5	4	4
68	5	7	3	19	4	4	9	5	6.5
69	7	9	3	29	4	3	2	4	2
70	5	5	4	3	4	2	3	6	0
71	3.5	4	3	4	3	2	4	4	1
72	22	27.5	17	4.5	34	5	4	15	5
73	22	27	17	7	32	5	5	15	7
74	18	19	14	6	24	6	5	10	22
75	16	17	12	4	20	4	2	13	3
76	15	19	12	6	22	4	4	12	5
77	17	20.5	13	6	22	3	4	12	4
78	15	17	11	5	20	3	3	10	4
79	15	17	11	5	20	3	3	10	4
80	22	26	16	6	27	5	3	15	5
81	16	20	14	4	22	4	3	14	3
82	19	22.5	15	8	26	4	4	15	5.5
83	16	20	14	4	22	4	3	14	3
84	19	22.5	15	8	26	4	4	15	5.5
85	25.5	32	17	5	35	5	4	19	5
86	14	19	11	4	20	3	3	12	3
87	25	34	21	5	38	4	3	18	6
88	17	20	13	8	22	4	6	12	6
89	17	19	12	5	20	3	3	12	4
90	17	20	13	6	20	5	3	11	5
91	15	18	12	5	20	5	2	10	6
92	16	18	12	5	20	4	2	11	8

93	16	20	12	6	24.5	4	3	12	4
94	26	29	18	6	35	4.5	4	17	5
95	23.5	43	36.5	17.5	60	13	5	16	8
96	29	36	21	6	37	5	3	19	6
97	18	21	15	8	24	4	5	13	6
98	18	14	12	6	21	4	5	10	7
99	18	20	15	8	26	4	5	12	6
100	21	25	16	5	31	5	3	16	4
101	17.5	20	12	12	25	5	4	11	10
102	21	25	15	28	25	7.5	9	12.5	8
103	10	3	9	5	8	12	11	4	11
104	27	30.5	18	7	36	5	6	17	6
105	28	29	17	4	38	4	3	18	5
106	26	32	18	7	36	4	6	19	5
107	25	27	17	5	35.5	4.5	4	17	5
108	19	21	24	10	26	5	5	14	5
109	21	28.5	14	56.5	26	7	5	12	6
110	20	24	16	7	30	4	6	14	6
111	18	19	13	7	23.5	5	3	12	6
112	22	21	15	10	26	6	7	14	6
113	21	19	12	9	24	5	5	13	6
114	18	19	13	11	23	9.5	5	12	5.5
115	17	20	14	7	21	5	3	12	4
116	17	21.5	13	5	23	5	3	13	4.5
117	17	21	14	7	25.5	4	5	12	4
118	18	19	14	7.5	25	5	5	13	5
119	7	5	10	8	6	5	5	6	2
120	10	6	9	6	4	6.5	4	5	2
121	30	33	21	6	40.5	5	4	20	6
122	18	22	14	6	26	5	6	15	5
123	8.5	13.5	7	19	10	4	5	5	5
124	9	7.5	6	10	7	3	6	6	4
125	8	7.5	9	16	7	4	38	6	8
126	6	5	3	4	5	3	3	4	2
127	8	6	5	9	8	4	5	5.5	5
128	8	6	5	9	6	4	5	7	3
129	26	29	18	8	34	6	6	16	8
130	17	18	12	10	22	6	4	12	5
131	13.5	17	11.5	6	19	3	3.5	11	5
132	13.5	17	11.5	6	19	3	3.5	11	5
133	37	33	19	6.5	38	6	5	18	10
134	21	23	16	6	26	5	4	14	6
135	19.5	24	15	7	28	8	4	15	6
136	27	33	21	6	34	5	4	20	5
137	27	33	21	6	34	5	4	20	5
138	14	19	13	6	22	4	4	12	4
139	14	19	13	6	22	4	4	12	4

140	19	22	13	10	25	4.5	5.5	14	7
141	21	21	13	10	25	4	5	14	6
142	17	19.5	12	6	22	4	3	12	5
143	19	20	13	9	22	4	3	12	6
144	17	18	13	5	22	5	4	12	5
145	16	19	13	5	22	4	3	12	3
146	16	18	12	6	21	5	3.5	11	4
147	14.5	17	13	5	21	4	3	12	4
148	23.5	30	17	5	33	4	3	18	5
149	20	24.5	18	11	29	9	4	13	11
150	17	22	15	7	28	5	3.5	13	4
151	19	23	14	8	26	4	5.5	14	4
152	18	23	20	11	25	6	4	12	5
153	18	18	12	9	22	5	4	13	6
154	14	19	16	9	21.5	4	6.5	12	4
155	18.5	19	15	6	23	5	4	13	5
156	17	22	14	11	26	4	5	13	7
157	22	28	19.5	6	36	5	4	17	5
158	21	26	14	15	26	6	5	12	6
159	22	27	18	12	33	6	6	16	9
160	21	28	16	11	32.5	6	6	17	7
161	17	20	13	24	23	7	4	12	5
162	17	28	14	34	26	6	34	12.5	13
163	22.5	21	13	13	23	6	25.5	12	6
164	15	23	14	14	28	9	9	11	6
165	24	24.5	17	9	31	5	6	16	6
166	24	24	17	8	30	6	9	16.5	7
167	20.5	21.5	16	11	26	7	6.5	14	7
168	40	19	12	15.5	23	7	7	12	5.5
169	19	20	14	16	22.5	5	5	12	7.5
170	28	31	20	13	36.5	8	8	18.5	10
171	22	25	16	28	34	8	18	13	7
172	20	22	15	15	29	7	6.5	13	7
173	30	28	18	14	34	6	7	16	9
174	30	23	13.5	11	25	5	8	13	6.5
175	21	21	16	11	26	6	6	13	8
176	18	20	14	10	23	5	5	11	6
177	26	26	18	11	33	6	6	16	9
178	21	21	29.5	11	24	9	4	13	5
179	19	23.5	17	12	26	6	5	13.5	7
180	20	23	28	9	26	5	4	14	4
181	18.5	21	16	11	23	9	5	13	5
182	27	22	15	11	32	12	6	13	6
183	20	23	15.5	11.5	23	5	5	13	6
184	18	18	23	13	24.5	5	4	11	7
185	20	19	14	11	24	5	7	13	8
186	21	20	17	22	17	6	5	11	7

187	27	31	20	9	37	6.5	5	17	7
188	24	27	16	16	29.5	6	4	14	7
189	22	22	18	7	27	6	4	12	5
190	36	38	26	7	46	7	6	25	7
191	20	22	15	11	27	6	6	14.5	8
192	19	20	13	6	25	5	3	15	4.5
193	8	6	5	9	6	3	5	6	4
194	6	7	25	11	4.5	3	4	5	2
195	9.5	6	11	9	5	4	3.5	6	4
196	22	25.5	17	17	48	15	12	17	8
197	25	23.5	17	13	36	15	6	16	10
198	9	9	6	12	8	4	4	6	4
199	8	9	7	18	9	5	4	7	5
200	10	7	7	11	9	5	7	6	5
201	7	6	6	9	6	9	4	4.5	32
202	8	7	9	19	6	4	3	5	4
203	7	4	4	10	4	4	4	4	39.5
204	9	6	33	8	7	3	5	5	4
205	6	7	5	11.5	5	4	3	5	3.5
206	6.5	6	3.5	16	8	5	3	4	9
207	17	21	13	11	22	9	4	13	10
208	18.5	20	11	11	20	6	4	10.5	17
209	42	37	21	12	37	7	8	20	13
210	39.5	36	20	12	39	8	8	21	13
211	35.5	38	24	10	43	7	8	20	10
212	19	18	13.5	6	23	5	4.5	11	8
213	22	29	18	9.5	32	5	6	16	7
214	25	31	18	10	33	5	5	16	7
215	22	29	18	9.5	32	5	6	16	7
216	25	31	18	10	33	5	5	16	7
217	24	30	20	11	35.5	6	6	16	7
218	18	20	14	7	25	4	6	13	6
219	26	30.5	19	11	34	5	7	18	8
220	20	23	16	11	26	5	6	16	8
221	16	21.5	14	10	22	5	4	12	5
222	19	21	11	8	23	5	4	11	6
223	18	20	14	7	21	5	4	11	4
224	18	21	12	8	21.5	5	3	11	5
225	18	19	11	7	24	5	3	11	5.5
226	19	20	14	10.5	19	5	3	12	9
227	19	20	12	7	22	5	3	12	4
228	24	26	14	9	28	6	4	15	9
229	21	29	17	22	28.5	5	4	14	9
230	21	26	17	13	28	6	4	14	6
231	22	25	15	9	88	9	5	15	6
232	24	26	17	17	32	8	5	14	8
233	23	26	17	20	32	12	5	16	9

234	21	23.5	16	10	26.5	8	5	15	6
235	23	25	16	11	32	10	6	15	7
236	17	19	13	7	20	4	4	12	5
237	21	19	13	17	21.5	8	5	12.5	8
238	60	59	208	64.5	95	32	96	209	140
239	20	21.5	13	12.5	27	6	4	13	6
240	21	26	15	15	25	5	10	13	19
241	20.5	21	14	31	28	7	4	12	13
242	18	19	13	11	23	5	5	12	6
243	21	22	15	11	28.5	6	7	15	7
244	17.5	22	15	16	23	8	5	14	7
245	19	19	13	11	23	6	4	12	5
246	26.5	31	17	17	36	9	9	16	13
247	35	31	21	16	38	8	9.5	19	14
248	23	26	18	12	25.5	8	7	20	6.5
249	23	24.5	14	13	28.5	11	7	13	11
250	27	19	13	11	22	6	7	12	6
251	20.5	19	14	9	21	8	7	17	6
252	26	26	19	10	33	8	7	17	8
253	25	23	17	17	30	7	5	15	8
254	25.5	25	22	9	30	6.5	5	16	6
255	22	23	60	16	29	7	4	15	8
256	21	24.5	14	12	27	6	9	15	6.5
257	20	20	15	15	68.5	5	4	12	10
258	22	21	13	9	24	5.5	4	12	13
259	28	29	20	10	36	8	8	16.5	9
260	21	22	14	8	26	5	4	13.5	11
261	22	27	14	9	67	9	5	14	6
262	26	22	16	18	28	10	7	13	14
263	24	22	16	24	32.5	13	16	12	11.5
264	23	21	15	14.5	29	12	9	13	6
265	24	26	14	13.5	22.5	14	7	12	7
266	18.5	21	12	13	20	6	4	11	30
267	10	7	6	9	7	3	5	6	4
268	8	5	5	6	4	3	4	5	3
269	23	24	17	21.5	38	7	5	15	7
270	21.5	27	18	13.5	31	7	8	16	10
271	21	22.5	14.5	14	26	6	5	20	23.5
272	12	12	10	15	10	5	6	8	3
273	9	11	6	13	9	4.5	5	7	3
274	9	11	6	15	8	5	4	8.5	5
275	10	6	4	16	6	8	6	6	7
276	6	6	5	12	5	6.5	4	5	4
277	7	7	5	11	5	4	5	5	4
278	8	9	6	9	9	4	6	7	4
279	9	8	5	11	10	8	5	6	3
280	11	9.5	6.5	18	8	9	13	5	58.5

281	15	17	13	23	7	17	12	6	8
282	8	7	5	19	8	8	3	18.5	6
283	20	20	15	11	23	9	6	13	50.5
284	20	19.5	13	24	26	6	5	11	14
285	19	17	13	11	21.5	6	5	12	5
286	17	17	11	4	16	4	3	10	4
287	17	17	11	4	16	4	3	10	4
288	23	28	20	10	31.5	7	7	16.5	8
289	19.5	23	15	11	26	12	8	12	7
290	27	29	16	39	69	47.5	7	17	19
291	22	31	19	31.5	28	7	5	14	20
292	24	25.5	17	15.5	29.5	15	4	15	9
293	22	31	19	31.5	28	7	5	14	20
294	24	25.5	17	15.5	29.5	15	4	15	9
295	26	31.5	20	9	38	6.5	8	19	11
296	19	20	16	12	21.5	7	3	12	11
297	29	28	15	9	27	9	8	14	7
298	20	21.5	20	9	22	7	4	13	7
299	21	23.5	22	15	24	5	5	12	6
300	20	22	13	12	25	7	5	15	7
301	20	20	12	8	19	5	3	11	4.5
302	20	19	14	9	21	5.5	4	11	9
303	19	20	12	8	21	7	3	11	4
304	19	18.5	13	10	21	7	3	12	7
305	22	20	15	6	22	5	7	11.5	6
306	23	26	19	14	30	9	5	14	10
307	21	24	17	10	34	7	4	15.5	7
308	22	26	16	10	26	12	7	14	10
309	18.5	21	13	6	25	5	3	13	7
310	21.5	23	16	16	26	8	8	14	9
311	18	20	13	8	19	5	4	11	6
312	21	20	13	8	21	7	5	13	6
313	14	18	14	12	16	6	19	7	4
314	25	33	20.5	10	37	7	7	20	8
315	21	24	15	19	27	7	5	39.5	7
316	37	37	23	13	43	10	9	21	12
317	8	7.5	5	10	10	5	7	5	4
318	24	22	14.5	26.5	30	8	11	13	14
319	23	27	16	13	23	22	8	12	12
320	19	23	15	6	27	6	4	14	5
321	18	23	15	9	26	5	4	12	7
322	20	21	14	9	24	7	4	13	7
323	21.5	22.5	14	14	29	6	5	12.5	8
324	21	24.5	16	19	34	7	5	13.5	6
325	23	22	13	12	26	7	6	11.5	11
326	22	23	15.5	14	27	9	5	13	11
327	21	27.5	17	15	29	7	5	14	11

328	21	24	16	20	27.5	6	6	13	6
329	20	16	11	11	21.5	4.5	4	10	5
330	19	25	13	29	24	4.5	5	13	13
331	31	30	20	16	38.5	8	9	18	11
332	25	24	17	10	28.5	6	10	16	5
333	21	27.5	23	13	25	10	25	12	7
334	30	34	21	15	42	7	11	20	12
335	29	44	22	92	40	8	44	16	8
336	28	30	18	10	34.5	7.5	5	17	9
337	38	31	16	52	27.5	11	29.5	12	16
338	28	27	19	19	29	7	6	15	8
339	22	23	15	9	28	20	5	15.5	9
340	23.5	24	16	13	30	17	31	15	10
341	21	20	14	10	27	5	4	13	8.5
342	18	17	18	15	30	10	6	13	6
343	24.5	30	29	19	29	11	15	15	24
344	25	35	14	11	39.5	8	9	13	5
345	21	22.5	15	11	33	6	13	12	10
346	17	16	11	10	21	7	8	11	12
347	25	24	17	12	29	7	5	16	7
348	33	37	25	13	44	7	10	22	11
349	43	22	12	24	22	9	11	12	11
350	7	8	6	42	6	4	3	125.5	5
351	28	24	18	25	31	8	4	15	8
352	26	26	17	23	56	8	10	15	10
353	31	31	21	17	32.5	10	7	15	11
354	20.5	24	15	21	26.5	6	5	14	13
355	11	6	5	9	23	9	3	5	4
356	9	5.5	5	12	9	5	4	5	7
357	8.5	8	5	17	56	6	5	6	24.5
358	11	8	7	23	9	26	5	6	15
359	8	8	5	13	9	5	8	5	4
360	7	6	4	11	6	3.5	3	4	4
361	21	20.5	16	7	24	6	4	13	9
362	20	22	14	8	26	7	4	13	14
363	20	20	12	10	23	8	4.5	13	11
364	17	17	12	5	20	5	3	11	5
365	16	21	13	29	21	5	6	11	13
366	19.5	18	20	21	20	13	166	12	28
367	16	21	13	29	21	5	6	11	13
368	19.5	18	20	21	20	13	166	12	28
369	27	22	18	14	30	7	4	14	15
370	25	26	15	16	28	7	4	14	13
371	25	26	15	16	28	7	4	14	13
372	46	23	14	9	24	8	4	13	12
373	23.5	20	14	12	24	9	5	12	8
374	21	23	17	11	50	14.5	6	12	6

375	28	24.5	15	12	25.5	12	7	13	9
376	25	20	11	10	20	9	3	11	6
377	25	26	20	15	32	10	5	15	17.5
378	32	84	21	69	29	25.5	15.5	15.5	10
379	23	24	17	11	28.5	10	7	15	6
380	43	25	16	10	24	8	6	16	6
381	21	24	15	26	26	6	4	14	6
382	23	24	14	9	26	8	4	14	10
383	25	20.5	18	13	27	7	4	13.5	9
384	21	21	14	11	24	6	4	12	7
385	20	22	14	16	22.5	6	5	13	7.5
386	15	19.5	12.5	7	22	5	4	12	5
387	19	20	13	7	21	6	3	12	6
388	19	21	13	30	23	8	14.5	10.5	14
389	12	7	5	11	7	7	4	5	9
390	11	7	5	11.5	11	5	5	5	12
391	10	7	6	8	6	6	5	5	6
392	21	20	13	9	22	7.5	4	13	6
393	26	32.5	19	26	36.5	9.5	5	16	10
394	19	19	13	25	22	11	5	11	21
395	21	29	15	34	29	8	7	12.5	12
396	19	24	14	30	25.5	6	5	13	16
397	18.5	19	12	12	33.5	31.5	12	11	26
398	24	24	17	9	36	7	12	14	7
399	22	24	17	15	30	6	5	14	13
400	24	26.5	17	10	36	11.5	5	15	9
401	26	28	17	14	34.5	30	9.5	17	11.5
402	24	32	41	44	28	11	13	13.5	12
403	23	33	102	40	28	11	10	13	13
404	20	25	14	14	22	6	9	11.5	6
405	19	19	12	8	20.5	10	6	11	8
406	20	18	13	14	20	6	4	11	5
407	43	28	18	14	30	9.5	8	16	13
408	24	25	16	13	31	11	8	15	7
409	25	24.5	16	14.5	26.5	9	10	14	8
410	22	24	13	9	25	7	4	13	7
411	20	19.5	13	25	25	7	6	11	9
412	30	33	19	23	36	12	20	16	10.5
413	28	34.5	20	13	29	9	16	16.5	9
414	24	29	16	76	33	9	6	14	10
415	33	38	23	10	45	8	7	19	9
416	21.5	22	12	17	22	8	6	11	11
417	27	23	14	16	24	8	4	12	19
418	23	33	16	74	224	11	7	15	48.5
419	23	20	15	12	27	9	4	13	7
420	21	21	41	12	22	5.5	5	11	6.5
421	26	25	17	22.5	25	9	5	18	27

422	267.5	232	940	284.5	436	154	496.5	1175.5	699.5
423	18	11	10	22	11	15	15	7	10
424	12	10	6	22	7	7	5	7	8
425	25.5	29	18	24	29	8.5	6	13	13
426	24	23	18	18	1306	23	5	42	18
427	32	34	18.5	139.5	32	19.5	59.5	17	13
428	26	24	15	16	25	7	6	15	17
429	19	17	13	20	20	8	15	10	15
430	10	10	4.5	25	13	6	4	4	30.5
431	13	11	8	14	13	6	10	8	8
432	13	8	7	17	16	6	11	6	55
433	10	12	6	12	8	7	7	5	4
434	16	14	10	16.5	11	13	8	8	27.5
435	19	8	6	16	7	12	14	5	4
436	14	10	8	30	325	14	16	7	17
437	28	22	15	19.5	25	9	6	15	8
438	19	21	14	8	142	8	6	12	16
439	27	22	16	10	27	12	4.5	13.5	11
440	27	28	18	14	32	7	4	16	7
441	27	28	17	16	30	8	4.5	16	7
442	22	26	16	17	27	7	5	14	12
443	27	28	18	14	32	7	4	16	7
444	27	28	17	16	30	8	4.5	16	7
445	22	26	16	17	27	7	5	14	12
446	20	22	13	11	25	6	4	12	15
447	20	22	13	11	25	6	4	12	15
448	17	21	13	14	99	8	6	12	12
449	21	21	14	14	22	8	3	12	6
450	22	25	16	12	26	8	5	14	7
451	22	23	23.5	9	26.5	10	4	14	13
452	23	24	18	15	26.5	11.5	5	14.5	18
453	24.5	27	17	31	25	9	13	12	34.5
454	20	21	13	11	64.5	6	6	12	8
455	19	24	15	16	25	6	28	13	14
456	20	21	13	8	22	9	5	13	7
457	20	20	12	11	23.5	7	3	11	7
458	22	20	13	10	20.5	6	4	12	7
459	24	19	16	13	26	10	7	13	6
460	27	27	18	13	31	9	7	16	10
461	26	24	17	31	29	6.5	6	16	8
462	26	41	20	43	28.5	7	68	16	8
463	28	26	18	16.5	28	21	6	15	9
464	23	22	15	10.5	21.5	10	4	12.5	7
465	19	20	14	7	22	6	3	12	6
466	23	20	14	12	24	7	6	12	7
467	25	22	15	9	25	7	5	14	9
468	24	20.5	14.5	13	25	9	6	12	12

469	29	28	17	24	30	9	7	15	10
470	23	24	24	19	23.5	10	24	11	14
471	20	21	13	11	23	6	6	13	13
472	19	23	13	27	24	5	5	12	22
473	21	23.5	13.5	29	25	5	5	12	10
474	17	24	15	39	24	6	6	12	11
475	29	27	17	12	32.5	9	20.5	15	8
476	22.5	34	25	58	27	10	8.5	15	18
477	24	26	16	20	29	9	8	14	9
478	22	22	17	20	29	9	12	13	11
479	133	151	456	149	241	84.5	235	630.5	291
480	31	32	19	33	55	8.5	6	18	13.5
481	29	28	16	12	31	10	5	17	10
482	28.5	32	18	87	28	47.5	131	14	14
483	26	19	18	19	24	26	16	30	24
484	43	35.5	21	57	37	18	35	17	22
485	29.5	21	19	32	33.5	18	6	14	19
486	24.5	22	18	14	22.5	9	7	14	7
487	24	21	16	24.5	25	9	8	13	5
488	19	17	13	16	21	4	4	11	5
489	31	32	19	29	45	11	8	19	7
490	29.5	31	21	14	52	12	8	18	13
491	22	22	14	11	30.5	6	5	14	6
492	22	25	17	18	26	6	4	13	6
493	24	24	16.5	16	30.5	8	8	13	6
494	26	26	15	31.5	115.5	16	7	14	52
495	23.5	30	18	18	25	8	6	14	10
496	26	27	17	17	26	7.5	7	15	9
497	28	23	16	11.5	33.5	6	17.5	12.5	14
498	25	29	19	24	50.5	10.5	18	14	10
499	23	24	28	12	28	9	9	14	7
500	23	24	14	35	42	8	7	12	48
501	28	28	19	39	32	11.5	10	13	15
502	25	36.5	19	21.5	32	6	11.5	17	7
503	24	29	17	32	138.5	11	31	13	29
504	26.5	23.5	17	25	29	14	8	12.5	53
505	23	21	15	13	30	9	9	14	8.5
506	11	7	5	18	5	4	5	4	3
507	8	8	4	14	7.5	7	5	5	6
508	40	40	29	21	46	11	16	26	17
509	28.5	28	17	14	28	9	6	15	10
510	31	26	18	11	29	7	5	15	11
511	26	25	16	14	30	6	4	15	8
512	27	27	16	21	28	10	6	13	12.5
513	26	27	17.5	20	27	10	6	14	9
514	28	29	20	19	35.5	9	13	19	14
515	29	30	19	19	64	13	8	17	31

516	27	27	18	26	42	8	5	17	21.5
517	30	32	19	88	38	11	8	15	10.5
518	14	12	8	17	14	7	12	7	8
519	16	10	8	18	29	9	5	6	25
520	14	12	6	18	10	6	4	6	10
521	14	9	7	18	10	7	5	7	13.5
522	13	8	7	19	7	6	4	6	22
523	16	9	7	15	32.5	8	5	5	9
524	6	5	5	9	15	4	3	5	8
525	26	28	18	6	32.5	5	4.5	17	6
526	27	23	14	13	27.5	11	6	13	16
527	38	21	16	10	24	8.5	36	14	10
528	24	24	22	13	25	10	14	12.5	21
529	21	21	17	12	26	9	8	13	7
530	19	30.5	15	55	51	12	9	13.5	10.5
531	42	35.5	23	22	41	10	9	20	14.5
532	26	22	17	13	45	10.5	5	15	26
533	31	26	18	12	26	11.5	5.5	14	8
534	32	28.5	20	21.5	41.5	11	5	18	10
535	23.5	25	15	28	25	8	4	14	10
536	32	28.5	20	21.5	41.5	11	5	18	10
537	23.5	25	15	28	25	8	4	14	10
538	23	22	15	22	26	9	5	14	10
539	23	22	15	22	26	9	5	14	10
540	24	21	13	16	21	10	5	11	6
541	23	19	15	21.5	21	10	4	12	7
542	41	23	19	24	26	11	6	14	18
543	23	23	14	13	23	9	6	13	10
544	23	25	13	11	23	6	5	12	6
545	28	27.5	19	20	32	18	8	14	10.5
546	25	20	16	11	27	8	7	11	18
547	24	20.5	14	12	22	8	4	12	6
548	26.5	29	25	16	29.5	8.5	7	16	10
549	27	24	16	12	26	8	4	13	9
550	27	24	16	10	26	8	4	15	9
551	210	210	801	259	397.5	129.5	389	915	536
552	23	20	16	12	24	11	8	12	20
553	24	21	14	13	23	8	3	11	9
554	23	20	14	10	22	10	3	13	6
555	23	25	15	23	39	8	3	14	9
556	23	25	15	23	39	8	3	14	9
557	19	19.5	15	7	23	8	5	12	7
558	20.5	21	14	14	22.5	6	6	13	6
559	22	22	15	18	25	7	6	12	11
560	18	23	13	15	25	6	4	12	5
561	20	19	13	25	23	7	5	11	9
562	24	26	18	27	32	8	11	16	12

563	25	24	16	13	30	7	6	14	7
564	30	26	18	20	36	31.5	13	21	18
565	26	23	17	14	27	8	5	15	13
566	25	26	16	35	41	7	8	13	13
567	22	25	17	18	28	10	8	14	9
568	26.5	23	13	17	25	9	7	13	6.5
569	25	20.5	14	10	22	10	8	14	9
570	24	21	14	12	38	9	5	12	21
571	39	32	19	15	34	14	7	17.5	13
572	67	24	16	23.5	28	15	6	16	17
573	24	21	15	18	22	7	6	15	7
574	27	22	13	18	23	10	5	12	20
575	26	20	13.5	14.5	19	7	5	12	23
576	31	28	15	19.5	33	14	5	14	12
577	23	21	21	10	27	6	4	13.5	6
578	23	21	26	11.5	28	7	6	13	6
579	32	27.5	21	16	35	10	14	19	17
580	24	21	14	10	26	8	6	14	6
581	20	22	14	21	25	9	12	12	14.5
582	28	29.5	20	18	33	11.5	7	16	8
583	29	27	19	11	29	10	6	16	7
584	28	24	16	18	29	10	5	14	13.5
585	23	36	17	16.5	25.5	9	6	16	6
586	32	21	15	19	24	11	6	13	18
587	29	30	19	27	42	8	8	18	17
588	28	25	16	14.5	33	9	6	19	202.5
589	27	28	19	11	34	8	12	66	10
590	25	33	17	17	32	8	8	15	7
591	33	28.5	17	16	31	8	6	13	15
592	28.5	24	15	27	48	21	132	15	15
593	23	20.5	20	27	26	9	11	14	5
594	10	6	8	20	6	5	5	5	9
595	14	14	14	22	7	7	7	6	22
596	14	9	10.5	24	410	7	5	5	76
597	8	9	5	14	9	6	5	5	10
598	26	8	9	56	8.5	12	6	6	8
599	22	21	14	11	20	10.5	6	12	9
600	29.5	15	17	10.5	25	-1	3	8	11
601	24	19	13	17	22	18	6	11	17
602	24	19	13	17	22	18	6	11	17
603	36	24	17	18	30	10	6	14	33
604	24	24	15	11	26	6	4	15	8
605	34	27	16	11	29	11	5	16	19
606	26	22	15	11	25	8	5	13	18
607	26	25	15	13	27	10	6	15	7
608	27	22	15	20	26	7	5	14	10
609	24	24	15	11	26	6	4	15	8

610	34	27	16	11	29	11	5	16	19
611	26	22	15	11	25	8	5	13	18
612	26	25	15	13	27	10	6	15	7
613	27	22	15	20	26	7	5	14	10
614	31	23	15	14	25	11	4	15	10
615	34	25	15	13.5	26	9	7	14	10
616	32	28	21	39	27.5	11.5	11	17	14
617	21	26	13	27	28	10	4	13	11
618	25	22	16	18	24	6	5	13	9
619	24	24	14	24	25	9	4	12	19
620	24	23	14	23	24	9	8	14	19
621	23	22	16	15	24.5	9	5	14	9
622	23	22	14	22.5	27	8	4	11	58.5
623	31	20	14	13.5	23	12	4	13	9
624	25	20	13	10	22	6	3	11	7
625	32	21	14	28	24	8	8	13	10
626	28	20	13	20	20.5	9	5	11	10
627	24	25	17	17	34	8	6	15	7
628	27	34	18	33.5	38.5	9	36	16	11
629	32	33	23	58	35	16	10	16	16
630	27.5	25	23	15	34.5	12	9	15	15
631	33	28	17	26	30	18	5	16	9
632	27	25	19	16	25	8	4	15	8
633	23	24	15	12	24	8	7.5	13	9
634	21	17	13	16	24	9	7	11	10.5
635	25	24	16	17	28	9	10	15	10
636	26	24	17	21	70.5	10	5	14	13
637	268	232	954	277	406	151.5	468	1057.5	663.5
638	28	21	16	11	24.5	11	5	14	8
639	26	20	15	17	25	8	6	12	7
640	25	26	16	27	28.5	6	5	13	7
641	33	49	18	36	33.5	19	17	16	10
642	27	34	20	73	28	13	14	13	15
643	24	23	16	25.5	25.5	11	6	12	25
644	22	23.5	16	27	23	6	6	12	17
645	22	27.5	17	34	25	7	7	17	17
646	238	194.5	738	240	312.5	119	382	780	532
647	26	26	13	31	27	9	6	14	16.5
648	20	19	14	12	21.5	5	5	11	5
649	149.5	165	531.5	207	213	90.5	271	686	399
650	23	23	14	25	24	7	6	14	7
651	28	21.5	16	20	23	12	5	11	22
652	35.5	35.5	20	40.5	29	7	16	17	13
653	26	22.5	16	13	57	11	8	15	13
654	28	24	16	24.5	31	10.5	6	15	16
655	24	25.5	15	31	31	9	5	13	12
656	36	25	20	16	35.5	34	9	12	13.5

657	23	24	14.5	25	25	6	4	14	5
658	26	25	15	20	34	10	15	21.5	9
659	27	26.5	18	15	27	9.5	9	12	8
660	47.5	22	16	10	25	8.5	6.5	15	8
661	26	23	14	15	27	9	18	12	9
662	29.5	32	17	17.5	38	9	43	15.5	9
663	24	22	16	11.5	25	6	5.5	13	7
664	27.5	28.5	17	23	29	10	7	13	15
665	240	233.5	776	274.5	415	142	407	965.5	538
666	25	28	18.5	14	29	8.5	6	13	8
667	26	24	16	23	27	9	5	13.5	40
668	255	258	862.5	305	495	150	452.5	1046	650
669	21	24	20	19	24	7	8	13	12
670	26	25	17	36	28	10	6	14	38
671	23.5	27	16	11	29.5	10.5	18	13	7
672	33	24	15	27	23.5	15	6	14	21
673	18	8	5	24	7	10	5	5	7
674	34	27	19	21	35	10	8	21	21
675	30	30	17	14	33	9	7	15	10
676	40	30	17	18	30	17	11	16	28
677	38	30	20	19	90.5	14.5	8	18	37
678	34	27	23	19	29	12	9	15	12
679	24	23	17	30.5	35	10	6	15	9
680	28	26	16	23	31	13	178	16	20
681	23	25	16	80.5	211	11	20.5	23	9
682	30	35	18	44	32	33	25	15	14
683	26	28.5	16	32	28	11	5	13	13
684	18	10	6	22	18.5	8	5.5	6	19
685	15.5	11	8	25	13	8	6	5	16
686	245	220.5	868	281	439	143	435.5	1027.5	646.5
687	15	8	9	16	8.5	11	6	6	39
688	28.5	8	11	15	73.5	6	8.5	7	8
689	76	9	5	22	6	10	18	6	9
690	25.5	24	14	19	26	16	5.5	13	64
691	31.5	27	18	16	30	16	6	15	20
692	240	225	879	261.5	389.5	128	403	894	537
693	255.5	230	916	270	392	139.5	400	935.5	579
694	26	20	13	14	19	15	5	13	17.5
695	24	22	13	26	19	7	6	11	47.5
696	255.5	230	916	270	392	139.5	400	935.5	579
697	26	20	13	14	19	15	5	13	17.5
698	24	22	13	26	19	7	6	11	47.5
699	29	29	18	16	31.5	10	6	16	18
700	30	29	17	14	30	9	5	16	7
701	32	26	18	13	31	9	5	15	9
702	33	27	16.5	23.5	28	10	6	16	10
703	32	28.5	16	14.5	29	11	4	15	13

704	30	29	17	14	30	9	5	16	7
705	32	26	18	13	31	9	5	15	9
706	33	27	16.5	23.5	28	10	6	16	10
707	32	28.5	16	14.5	29	11	4	15	13
708	25	22	16	19	24	9	4	13	6
709	25	22	16	19	24	9	4	13	6
710	27	25.5	17	20	27	11	5	14	17.5
711	57	21	16	24	27	9	5	13	26
712	31	21	14	19	23	15.5	6	14	10
713	31	29	19	17	30	7	5	14	11
714	30	27	20	21	31	9	7	16	11
715	38.5	25	16	20	30	18	7	13	13
716	34	19	12	11	23	14	4	13	8
717	20	19.5	14	13	20	8	4	13	10
718	28	21	13	11	23	11	4	16	7
719	25	21.5	13	16	22	7	4	13	11
720	25	22.5	14	11.5	28	8	5	14	11
721	32.5	26	18	14	30	18	6	15	9
722	32	23	16	16	26	11	4	13	9
723	26	20	14	10	26	8	4	12.5	8
724	32	18	13	16	22	12	5	13	7
725	27	21	14	15	23	10	5.5	15	10
726	30	18	14	19	21	12	4	14	7
727	328.5	270	1938	134	543.5	185	538	802	949
728	21.5	23	13	24	21	9	27	13	10
729	16	10	9	28	8	9	10	6	22
730	25.5	20	15	8	26	7.5	5	12	8
731	26	26	16	18	33.5	9	9	14	8
732	24	21	14	26	31	9	9	12	5
733	279	235	981	265	442	163	441.5	931.5	659
734	28	29.5	19	38	28	18	10	14	47
735	18	18.5	12	28	22	6	4.5	11	6
736	18	23	12	29	22	6	5	12	12
737	22	22	11	24	22	5	4.5	12	12
738	29	26.5	19	97	54.5	13	9	16	10
739	27	30.5	17.5	27	35	12.5	8	16	35
740	41	20	36	24.5	21	13	5	14	16
741	32	25	16	26.5	32	14	8	13	12
742	26	26.5	17	26	31	9	8	13	8
743	27	19.5	14	8	25	10.5	6	11	10
744	37	35	22	24	50	17.5	9	17.5	10
745	37	35	21	27	33	12	10	12	10
746	23	21	15	14	41	13	5.5	12	10
747	25	21	14	16	32	9	9	13	12
748	22	25	15	20	23	8	9	11	8
749	34	26	31.5	18	31	17	12	13	14.5
750	33	49	40	168	37.5	20	18	17.5	11.5

751	24	21	14	18	31	11	5	64	28
752	22	22	17	14	26	7	6	14	6
753	22	19	12	12	24	5	6	12	9
754	29	23	15	17.5	28	12	5.5	13	33
755	19	6	5	14	5	6	5	5	6
756	18	6	6	17	6	8	4	5	8
757	19	10	9	19	9	11	6	5	14
758	36	33	20	21.5	37	15	12	17	13.5
759	31	29	20	14	33	10	7	16	33
760	29	29	22	25.5	33.5	9.5	7	19	19
761	273	237	993	282.5	486	174	488.5	1155.5	649
762	21	15	10.5	36	12	11	9	8	16
763	18	12	7	35	7	10	5	6	17.5
764	286	235	1025	258	512	172.5	505	1076	711.5
765	17	13	11	35	12	11.5	9	7	26.5
766	16	10	6	18	11	12	7	5.5	23
767	13	5	6	11	5	6	5	6	3
768	10	7	7	15	9	7	6	5	5
769	26	28	34	23	26.5	7.5	6	16	8
770	37	22.5	17	12	32	27	13	16	9
771	32	21	14	15	20	11	5	12	14
772	23	18.5	16	26.5	20	10	5	13	12
773	32	21	14	15	20	11	5	12	14
774	23	18.5	16	26.5	20	10	5	13	12
775	47	41	23	28	39	13.5	9	20	17
776	30.5	30	22	17	89.5	12	6	17	14
777	37	30	18	22	36.5	11	5	17	8
778	27	27.5	16	14	29	11	8	16	13
779	26	21	14.5	13	27	7	4	16	8
780	37	30	18	22	36.5	11	5	17	8
781	27	27.5	16	14	29	11	8	16	13
782	26	21	14.5	13	27	7	4	16	8
783	23	20	15	18	24	8	5	11	16
784	31	22	15	25	24.5	11	6	13.5	10
785	24	25	15	13.5	27	11	4.5	15.5	12
786	57.5	25	16	19	28	12.5	9	14	19
787	55	24	15	17	25	13.5	6	14	15.5
788	43	27	16	31	27	12	6	15	23
789	35	26	17	22	28	10	6	14	12
790	32	22	17	20	25	13	7	9	8
791	29	21	13	18	21	10	4	14	9
792	28	29	17	25	31	10	10	15	9
793	29	24	15	16	26	10	4	14	13
794	20	20	15	20	22.5	9.5	8	13	9
795	297	299.5	1739	154	404.5	192	509	1010.5	937
796	29.5	28	17	19	32	9	7	15	12
797	8	7.5	5	15	5	6	5	5	8

798	41	36.5	23	19.5	43	12	6	19	16
799	31.5	23	15	22	25	9.5	6	13	10
800	66.5	24	16.5	20	28.5	17	9	14	19
801	31	26	18.5	26	31	15	6	13	30.5
802	31	22	18	19	27	10.5	20	14	24
803	34	26	21	18	36	12	14	18	13
804	29	28	29	29.5	28.5	16	7	15	20
805	22	25	14	23	29	5	5	14	17.5
806	26	23	14	28	29	15	7	13	24
807	21.5	20	12	19	21	7	3	11	8
808	29.5	29	18	31	31	15	9	15	18
809	32.5	34	50	33	34	14	37	17	19.5
810	27	31	17	48	39	10	7	15	32
811	40	29	20	23	43	22	9	19	23.5
812	27	28	17	41.5	29	12	22	15	12
813	29	27	15	38	26.5	12	7	18	23
814	33	24	22	70	36	11	26	14	14
815	230	213	757.5	230	366.5	133	378	981.5	540
816	41.5	30	18	21	35	16	6	19	33.5
817	25	27	22	15	26.5	9	9	16	8
818	26	21	14	21	25	6	5	13	7
819	28	22	14	36	21	15	7	11.5	36.5
820	28	23	17	13	29	6	5	14	8
821	28	24	19	13	26	7	4	13	8.5
822	54	45	21	43	61.5	15	7	18.5	11
823	251	233	885	265.5	360	152	431	935	624
824	35	28	19	17	35	20.5	7	18	20
825	28	25	16	21	37	10.5	9	15	15.5
826	35	41	30	71	37	23	12	16	16
827	26	26	17	12	27	10	12	15	30
828	30	27	20	23	26	17	7.5	15	27
829	21	33	16	41	60	9	9	12.5	17
830	15	8	17	24.5	5	4	5	4	4
831	17.5	7.5	6	16	8	10	6	6	17
832	28.5	29	17	14	30.5	8	5	15.5	11
833	27	24	18	15	36	8	6	15	24
834	25	25	16	21	32	11	7	15	19
835	24.5	25	17	19	38	10	6	14	14
836	37	23	16	19	29	16	6	14	15
837	17	10	6.5	25.5	8	8	5	6	24
838	25	9	8	27	11	5.5	7	6	15
839	15	27	9	60.5	10	11	15	15	117
840	13	13	8	36	11	17	7	5	18
841	27	23	17	15	31	12	10	14	36
842	22.5	22	20	18	24	7	14	13	16
843	30	30	16	43	27	14.5	15	15	52
844	26	22	17	16	26	12	7	12	9

845	25.5	20	14	17	21	10	6.5	11	30
846	31.5	21	20	22	21	14.5	12	11	11
847	24	19	14	19	20	11	6	11	18
848	24	19	14	19	20	11	6	11	18
849	284	243	1042	298	433	156	464	1072	600.5
850	32.5	26	18	10	28	10	5	15	8
851	42	36.5	25	26	34	12	6	17	11
852	29	24.5	16	16	32	8	6	13	22
853	42	36.5	25	26	34	12	6	17	11
854	29	24.5	16	16	32	8	6	13	22
855	295	254	1066.5	315	421	171.5	523	1154.5	699.5
856	25	21	15	17	22	11	6	13	7.5
857	26	21.5	18	37	27	9	5	13	11
858	34	21	14	18	24	12	5	13	13
859	295	254	1066.5	315	421	171.5	523	1154.5	699.5
860	25	21	15	17	22	11	6	13	7.5
861	26	21.5	18	37	27	9	5	13	11
862	34	21	14	18	24	12	5	13	13
863	27	28	17	24	30	12	5	16	11
864	28.5	24	16	20	23	12	6	13	23
865	31	24	15	21	29	10	8	13	10
866	23.5	21	14	18	24	8	4	14	23
867	26.5	29	18	20	31	9	5	15	12
868	58	24	15	16	28	9	9	14	17
869	241	221	872.5	266	465	145	412.5	995	576
870	48	40	19.5	16	32	11	10	17	8
871	23	22	13	15	26	9	3	12	10
872	275	260	953	267	490.5	168	478.5	962	673.5
873	19	9	13	22	7	7	9	7	13
874	34	27	16	22	37	12	5	15	12
875	53	29	18	25	27	11	10	14	24
876	42	23	16	19	27	11	7	15	44
877	25	24	14	14	42	7	5.5	13	11
878	31	34	21	34	32	11	9	17	15
879	30	23	17	28	26	20.5	7	15	16
880	33	33	30	36	34	20	18	16	14
881	35	24	19	27	30	15	6	16	13
882	24.5	22	14	16	31	11	6	14	42.5
883	35	25	15	18.5	25	9	5	13	30
884	33	25	15	25	25	17	7	13	12
885	30	21	14	19	25	17	5	12	22
886	34	23	13	19	23	13	6	12.5	48
887	43	35	24	32.5	38.5	12	52	21	18.5
888	35	39	28	26.5	46.5	11	15	22	14.5
889	302	237	984	270	441	155	429.5	988.5	637.5
890	32	22	16	14	26	6	6	12	6
891	28	26	17	14	32	13	10	15	12

892	35	35	22	18	34.5	8	7	18	11
893	31	28	19	21	35	9	9	16	11
894	31	30	16.5	31	74	23	7.5	13	29
895	34.5	43	23	76	62	24.5	85	15	17
896	36	25	15	32	23	16	6	12	33
897	18.5	24	18.5	25	26	6	12.5	12	18
898	16	9	10	24	6	7	6	6	13
899	39.5	31	21	23	35	15	7	18	28
900	30	31	24	25	42.5	10	38	16	20
901	298	267	1103	345	488.5	173	539	1135.5	670.5
902	32.5	26	18	29	24	17	7	16	25
903	31	52	19	31	29	14	7	15	18
904	26	9	7	25.5	16.5	12	6	7	16
905	22	8	6	19	9	12	64.5	4	23
906	12	9	6	22.5	10	5	4	5	21
907	16	11	16	39	102	12	17	7	26
908	8	7	5	17	6	6	4	6	3
909	-1	11.5	19	21	13	3.5	12	8.5	25
910	26	18	16	17	21	16	7	10.5	22
911	33	18	14	21	21	10	7	10	14
912	28	19	14	17	21	18	6	14	10
913	23	19	14	22	20	13.5	8	12	26
914	26	18	16	17	21	16	7	10.5	22
915	33	18	14	21	21	10	7	10	14
916	28	19	14	17	21	18	6	14	10
917	23	19	14	22	20	13.5	8	12	26
918	37	28	23	20.5	39	13	8	17	33
919	39	31	17	16	32	14	6	18	12
920	46	28	19	17	34	16	6	17	10
921	34	27	17	17	31	12	6	17	8
922	31.5	27.5	20	18	30	22	5	17	17
923	41	25	17	16	36	15	6	16	13
924	27	23	15	20	25	7	5	13	10
925	34	24	16	19	26	17	5	23	11
926	32	30	19	19	33	8	6	17	8
927	39	31	17	16	32	14	6	18	12
928	46	28	19	17	34	16	6	17	10
929	34	27	17	17	31	12	6	17	8
930	31.5	27.5	20	18	30	22	5	17	17
931	41	25	17	16	36	15	6	16	13
932	27	23	15	20	25	7	5	13	10
933	34	24	16	19	26	17	5	23	11
934	32	30	19	19	33	8	6	17	8
935	29	28	17	15	28	10	8	15	16
936	37	32.5	19	54	28	17	6	16	20
937	33	27	16	16	30	15	6	15	15
938	32	20	14	16.5	24	15	5	14	10

939	274	257.5	1012.5	273	537.5	163	463	1004	663
940	24	23	14	25	35.5	11	5	13	12
941	41	34	18.5	30	32	16	6	17	16
942	35.5	24	18	18	25	12	7	16	8
943	56	24	16	17	29	15	9	14	12
944	40.5	26	17	18.5	27.5	12	6	15	17
945	26	19	15	16	25	18	4	14	9
946	27	27	20.5	27	39	13	8	16	16
947	26	21	14	8	24	7	4	13	7
948	31.5	27	15	24	25.5	18.5	8	15	24
949	35	24	16	23	27	15	5	15	12
950	31	23	15	14	24	8	4	13	10
951	33	26	30	29	31	11	10	14	51
952	33	25	16	14	32	8	7	15	12
953	26	19	15	19	21	13	5	13	13
954	26	19	15	19	21	13	5	13	13
955	42	26	21	15	30	17	6	15	10
956	60.5	28	19	14	30	22	5	15	9
957	60.5	28	19	14	30	22	5	15	9
958	24	23	15	21	26	6	8	14	8
959	532.5	428.5	3251	211.5	1058.5	367	901	1321.5	1409
960	24.5	26	13	23	31	6	33.5	13	8
961	20	25	15	35	32	6.5	7	14	19.5
962	28	34	20	19	30.5	15	10	17	11
963	30	30.5	20	27	38	16	11	16	31.5
964	29	26	17	21	31	9	6	17	17
965	38.5	25	14	9	29	13	4	14	10
966	28	24	15	17	24	11	7	12	10
967	24	17	15	11.5	24	9	6	13	7
968	27	21	13.5	21	30	7	6	13	9
969	27	29	17	23	28	11.5	8	13	12
970	41	27	22	29	35.5	19	6	18	26.5
971	40	24	18	21	33	21.5	6	15	13
972	35	28	15	33	19	17	5	14	17
973	26	20	12	20	23	24.5	6	14	11.5
974	34	27	16	13	36	9	8	16	9
975	29	26	18	18	31	9	6	15	12
976	259	224	839	227	416	138	408.5	865	570
977	27	26	19	27	32	15	9	27	34
978	30	31	15	23	27	9	6	14	10
979	46.5	34	22	21	26	16	23	14	16
980	28.5	22	17	29	24	11.5	7	13	52.5
981	45	40	21	15	41.5	9	7	19	25
982	29.5	24	17	12	31.5	10	19	14	10
983	483	431.5	3012.5	208	914.5	309	757.5	1266	1266.5
984	36	25	15	18	26	14	5	12	59
985	556	469	3696.5	230.5	1110	358	922	1389.5	1429

986	36	20.5	14.5	26	22	14	6	13	21
987	463	471	3456	182	951.5	319	918	1411	972
988	17	9	7	23	11	10	11	5	28
989	309	262	1092.5	310	549	202.5	539.5	1095	621
990	26	21	14	23	83.5	9	7	12	14
991	16	12	8	26	11	12	8	6	15
992	18	7	6	14	8	8	4	6	17
993	16	10	9	15	6	10	11	7	38
994	14	7	7	14	5	4	4	16	5
995	21	14	9.5	31	10	12	7	6	32
996	23	11	10	43	10	12.5	10	7	46
997	9.5	10	6	16	8	9	4	5	4
998	36	31	21	25	35	18.5	8	16	20
999	24	24	17	19	24	9	9	13	26.5
1000	41	22	14	18	22	19	5	14	15.5
1001	38	20	14	16	21	16	6	13	14.5
1002	30	18	13	20	20	13.5	6	12	14
1003	41	19	14	16	23	18	6	11	17
1004	28	27	15	25	25	12.5	7	12	12
1005	30	18	13	20	20	13.5	6	12	14
1006	41	19	14	16	23	18	6	11	17
1007	28	27	15	25	25	12.5	7	12	12
1008	39	32	23	20	37	15	7	17	14
1009	39	31	23	20	35	14	9	16.5	17
1010	41.5	32	22	18	38	15	7	17	34
1011	39	23	17	10	28	12	5	15	36.5
1012	30	24	17.5	14	29	11	5	15	14
1013	34	25	21	18	29	10	7	14	13
1014	29	29.5	20	31.5	32	8	6.5	15	13
1015	34	26.5	18.5	13	28	11	4	16	9
1016	32.5	28	17	17.5	29	12	5	16	17
1017	29	26	15	19	30	11	5	16	23
1018	33	25	18	17	27	14	6	13	20
1019	29	29.5	20	31.5	32	8	6.5	15	13
1020	34	26.5	18.5	13	28	11	4	16	9
1021	32.5	28	17	17.5	29	12	5	16	17
1022	29	26	15	19	30	11	5	16	23
1023	33	25	18	17	27	14	6	13	20
1024	38	23	15	23	25	17	6	15	18
1025	38	23	15	23	25	17	6	15	18
1026	32	33	20	28	33	11	8	16	29
1027	31	26	17	33	29	14	9	15	29
1028	30.5	21.5	15	19	27	11	4	12	36
1029	43.5	40.5	25	26	41	30	16	22	16
1030	63	22	14	15	26	14	6	15	20
1031	60	28.5	18.5	35	29	16	7	16	24
1032	31.5	23.5	17	24	23	14	8	12	19.5

1033	299	263.5	989	265	510	181.5	447.5	987	629.5
1034	38	38	29	30	47.5	14	24	24	28
1035	23	19	13	12	21	6	4	13	9
1036	22	27	22	40	23	7	7	13	13
1037	32	31	20	21	32	14	8.5	15	24
1038	35	46	22	81	52	16	18	13	27
1039	84	53	34	63	65	80	35	15	37
1040	59	41.5	24	37	64	14	21	21	16
1041	29	10	8	19	8	14	4	7	7
1042	20	10.5	6	22	11	9	7	6	16
1043	31	8	5	18	8	14	6	5	10
1044	27	16	13	18	19	10	6	11	13.5
1045	27	16	13	18	19	10	6	11	13.5
1046	27	24	19	21.5	27.5	9	9.5	14	14
1047	39	26.5	19	19	31	14	6	16	22.5
1048	33.5	29	16	21.5	32	11	6	16	16
1049	32	24	16	22.5	30	10	4	14	19
1050	24	25	16	11	30	8.5	5	15	7
1051	29	21	15.5	16.5	24	13	6	11	10
1052	44	35	20	33	36	17	14	15	18
1053	46.5	30	20	34.5	33	46.5	15	19.5	34
1054	32	25	18	34	86	19	7	15	12
1055	46	26	16	23	30	19	6	17	14
1056	22	23	15	27	23	6	5	12	18
1057	290	272	932.5	290	446	161.5	466	1034	639
1058	50	34.5	21	34	36	21	9.5	18	24.5
1059	34	23	17	16	31	14	6	14	30
1060	24	20	14	20	31.5	9	8	12	9.5
1061	42	31	21	19	36	18	10	20	16
1062	38	31	17	23	31.5	20	7	15	18
1063	28	24	15	20	24	12.5	6	13.5	21
1064	38	30	20	23	33	17	8	16	31
1065	33	22	15.5	22	24	13.5	6	14	11
1066	41	21	16	29	28	19	6	14	17
1067	255.5	225	880	247	450	137	388	958.5	532
1068	32	30	20	26.5	38	10.5	8	18	9
1069	39	28	20	26	33	18.5	8	16	29
1070	42	28	21	22	36	14	9	17	15
1071	40	29	17	24	42	32	15	17	37
1072	30	26	28	12	35	10	36	13	26
1073	54	22	14	12	29	8	6	14	9
1074	41	22	16	24	29	16	5	14	10
1075	32	20	14	18	28	10	5	13	13.5
1076	27	23	13	17	26	8	15	11	47.5
1077	38	32	24	22	42	16	10	16	14
1078	28	31	17	30	32	8	7	16	9
1079	36	25	16	23	26	15	6	13	19

1080	40	26	22	15	32	13.5	6	18.5	26
1081	31	43	23.5	52.5	30	15	16	15	48
1082	34	29.5	17	34	29	13	7	16	12
1083	31	29	17	28.5	27	15	7	14	22
1084	26	25	17	32	41.5	10	5	14	15
1085	22	12	7	21	8	10	6	6	23
1086	27	10	6	18	8	14	4	7	20
1087	21	10	7	27	8	11	7	7	10
1088	29	13	9	38.5	12	20	8	8	16.5
1089	16.5	12	6	27	10	11	9	6	34
1090	314	260.5	1100	279	532	177.5	553	1177	773
1091	18	11	6	23.5	34.5	10	6	6	14
1092	21	12	12	26	10	10	7	7.5	13
1093	20	8	7	23	8	10	8	7	18
1094	20.5	12	6	26	9	11	6	7	14
1095	689	477	3963	215.5	1053	375	1018.5	1455	1677
1096	19	7	5	23	11	11	5	11	14
1097	36	23	18.5	21	38.5	19	7	14	15
1098	596	471.5	3952	240	1074	375	962	1267.5	1505
1099	37	21.5	16	25	27	28	7	14.5	22
1100	47	21.5	14	24	24	28	7	14	15
1101	30	19	15	17	26	21	7	13	17
1102	562	459	3749.5	230.5	1009	391	879	1301.5	1423
1103	35	18.5	14	18	19	14	6	12	9
1104	562	459	3749.5	230.5	1009	391	879	1301.5	1423
1105	35	18.5	14	18	19	14	6	12	9
1106	273.5	277	1014	315.5	483	185	485.5	1141	683
1107	54	31	23	25	34	33.5	9	16	17
1108	30	24	18	11	29	6	5	14	14
1109	35	25	18	18	31	10.5	5	16	14
1110	44	29	17	16	32	18	5	16.5	9
1111	42	33	24	29	33	19	6	17	11
1112	42	28	21	18	29	19	6	17	33
1113	34	26	16	24.5	28	17	6	15	14
1114	27	25	15	13	26	9	5	15	16.5
1115	29	26.5	17	17	29	9	5	16	10
1116	35	25	18	18	31	10.5	5	16	14
1117	44	29	17	16	32	18	5	16.5	9
1118	42	33	24	29	33	19	6	17	11
1119	42	28	21	18	29	19	6	17	33
1120	34	26	16	24.5	28	17	6	15	14
1121	27	25	15	13	26	9	5	15	16.5
1122	29	26.5	17	17	29	9	5	16	10
1123	43	31.5	20	39	27	20	8	16	21
1124	38	22	27	21	26	18	7	15	19
1125	43	31.5	20	39	27	20	8	16	21
1126	38	22	27	21	26	18	7	15	19

1127	36	24	15	18.5	25	17	7	15.5	10
1128	23	24	15	21	25	14	6	12	14
1129	27.5	25	16	14	25	9	5	13	16
1130	43	24	18	18	26	16	7	16	19
1131	31	20	15	18	20	13	5	13	10
1132	27	18	14.5	20	25	11	5	11	12
1133	282	239.5	969	259	489.5	148	436.5	935	650.5
1134	516	568	2575	303	1949	491	1085	1580.5	1648.5
1135	48	37	23	30	35	15	10	16.5	51
1136	34	29	19	22	28.5	14	6	14	15
1137	44	25	16	16	28	13	5	15	14
1138	49	24	18	23	27.5	10	7.5	17	17
1139	479	417.5	3204	209.5	1005	360	798	1193.5	1259
1140	46.5	28	19	27	27	17	8.5	18	13
1141	68.5	25.5	16	11	34	14	24	16	7
1142	38.5	28	15	20	26	14	6	15	17
1143	27	23	15	13	25	9	4	13	9
1144	39	26	16	34	28	12.5	7	15	18
1145	39	26	18	23	28	15.5	7	16	12
1146	29	20	17	17	28	8	6.5	12	17
1147	43	30	19	26	29	20	8	17	15
1148	36	28	19	23	32	14.5	7	16	32
1149	55	20	14	22.5	21	17.5	5	13	24
1150	55	20	14	22.5	21	17.5	5	13	24
1151	34	31	18	28	35	15	8	15	49
1152	25	27	19	21	37	7	11	14	6
1153	23.5	24	14	25	23	9.5	27	12	14
1154	39	23	15	30	23	21	8	15	21
1155	33	22	18	27	29	11	5	14	16
1156	41	28	18	26	32	19	9	16	14
1157	38	26.5	19	43	24	21.5	10	15	46
1158	25	27	16.5	30	39	10	7	12	18
1159	35	28	20	15	37	6	8	19	13
1160	28	25	17	15	31	19	5	14	11
1161	26	28	13	45	32	11	4	14	13
1162	72	34	25	23	32	18	18	16	25
1163	38	46	22	69.5	35.5	45	17	15	28
1164	36	24.5	17	29	25	17.5	6	14	59
1165	31	26	18	21	26	12	7.5	14	22
1166	44	31.5	20	16	33	12	9	16	14.5
1167	31	27	20	21	34	14	9	18	23
1168	45.5	26	21	40	35	15	7	14	33
1169	24	22	14	12	46	9	16.5	15	8
1170	31	24	20	15	29	11	8	18	34
1171	643.5	503.5	3952.5	217	1238	417	1058	1566	1616.5
1172	31	13	11	30.5	13	14	6	8	11
1173	23	11	8	26	9	16	6	8	24

1174	23	11	7	19.5	10	11	111.5	6	22.5
1175	26	10	7	25	14	15	6	7	19
1176	785.5	539.5	4520.5	261.5	1312	478	1160.5	1583	1915.5
1177	15	10	7	15	9	8	5	6	10
1178	27	10	6	23	16	9	6	7	12
1179	17.5	9	6	22	7	9	7	4	14
1180	17	8	7	11	9	16	6	6	13
1181	290	256	964	264	445	144	412	930	552.5
1182	30	23	14	41	29	14.5	22	13	21.5
1183	30	26	17.5	26	29	11	8	12	30
1184	40.5	22	18	24.5	23	17.5	8	14.5	13
1185	35	19.5	13	18	24	18	7	12	15
1186	40.5	22	18	24.5	23	17.5	8	14.5	13
1187	35	19.5	13	18	24	18	7	12	15
1188	42	30	25	22	34.5	18	10	18	61
1189	590	468	3787	242	1068	351	1012.5	1359.5	1613
1190	37	24.5	19	12	29	11	6	14	12
1191	45	24	16	15	28	25	6	17	13
1192	349	287	1196	323	488	187	548	1167	748.5
1193	349	287	1196	323	488	187	548	1167	748.5
1194	321	275	1135	300	580	190	524	1123	701
1195	33	28	19.5	20	29.5	11	6	15	24
1196	31	25	16	23.5	26	13	5	15	17.5
1197	32	22	18	21	25	13	7	13	20
1198	46	22	15	18	27	20	6	15	11
1199	35	22	18	18	22	13	5	13	11
1200	38	25	17	17.5	29	11	6	15	18
1201	56.5	23	17	25	33	10	8	16	25.5
1202	35.5	26.5	17	39	28	12.5	6	17	12
1203	28	22	17	11	22	10	8	12	11
1204	40	25	17	32	27	18.5	8	16	14
1205	40	32	22	32	36	13.5	9.5	17	14
1206	28	22	13	15.5	25	8	4	13.5	7
1207	43	22	15	15	30	16	5	15	18
1208	32	21.5	13	11	21	10	4	12	8
1209	27	22	14	20	21	9	6	11	14
1210	35	31	22	27	34	14	10	16	18
1211	43	26	16	22	31	27	6	15	33
1212	276	222	845.5	250	491	152	410	936	549.5
1213	32	26	14	27	27	15	7	14	50
1214	38	17	12	19	18	16	6	12	10
1215	38	17	12	19	18	16	6	12	10
1216	47.5	27.5	20	20	29.5	22	7	15	40.5
1217	36	24	16	30	25	15	10	14	15
1218	36	24	16	30	25	15	10	14	15
1219	43	20	15	10	24.5	7	6	11	6
1220	597.5	487	3838	223.5	1127.5	399.5	936	1301	1465

1221	35.5	25	18	25	28	24	7	15	25.5
1222	29	23	16	16	27	7	5	17	9
1223	33	24	18.5	40	26	-1	-1	14	-1
1224	32	33.5	19	30	36	15.5	8	20	36.5
1225	31	42	27	50	40	15	10	17	20.5
1226	44	30	17	24	59	45	51	16.5	19
1227	31	39	26	29	33	32	46	17	30
1228	46	31	18	26	35.5	19	8	18	17
1229	44	32	19	19	36	24	8	20	25
1230	44	23	16.5	21	26	20	5	16	15
1231	24	23	23	27	33	18	24	30	17
1232	48.5	26	16	26	29	22.5	7	16	52
1233	36	20	15	25.5	25	16	6	13	25
1234	41.5	74	31	34	39	25	11	18	12
1235	35	26	19	20	125	15	7	16	97.5
1236	46	27	17	15	278	12	8	16	17.5
1237	42	23	21	27	26.5	17	9	15	38
1238	27	35	16.5	34	27	13	13	13	29
1239	37	21	17	37	28	14	6	12	18.5
1240	37	39	38.5	82.5	45.5	27	73	40	17
1241	49	43	22	40	41	44	14	18	116
1242	29	9	8	19.5	8	15	11	8	14
1243	15	8	10	23	7	10	5	6	10
1244	23	10	8	26	9	13	6	6	11
1245	28	7.5	8.5	21	6	14	7	6	12
1246	35	8	7	24.5	15	11	6	6	19
1247	40	27	32	27	28	15.5	6	17	18
1248	33	13	14.5	26	39	18	19	9	16
1249	33.5	12	8	21	9	15.5	5	8	9
1250	35	10	7	22	8	13	4.5	7	16.5
1251	28	8	9	19	7	13	5	6	16
1252	28	14	9	30	13	17	8	10	26
1253	20	14	8	29	13	10	8	9	22
1254	35	8	6	19	8	18	7	8	14
1255	21	15	10	39	11	15	14	7	42
1256	28	8	9	17	102	14	6	6.5	19
1257	17	9	6	32	7	9	5	6	13
1258	18	8	5	26.5	6	8	5	5	12
1259	50	29	25	23	30	13	21.5	13	13
1260	38	18	13	14	20	15	6	11	12
1261	22	18	12	13	18	10	5	10	8
1262	38	18	13	14	20	15	6	11	12
1263	22	18	12	13	18	10	5	10	8
1264	50	30	24	18	40.5	17.5	7	20	42
1265	46	29	24	20	53.5	12	10	16	25.5
1266	46	31	21	18	36	24	6	19	19.5
1267	43.5	28	19.5	18	34	17	7	22	11

1268	42	28	21	17	34	15	9	17	119.5
1269	50	25	20	18	32	11	8	15	13
1270	41.5	24	17.5	24	30	15	6	17	13
1271	41.5	24	17.5	24	30	15	6	17	13
1272	37	32	19	23	32	12.5	7	16	24
1273	47	29	23	36	31.5	17	7	14	24
1274	43.5	27.5	18	35.5	27.5	13.5	6	16	11
1275	37	27	18	26	29.5	15	9	14	37
1276	45	22.5	16	18	25	16	5	15	20
1277	30	20	13	23	24	10	3.5	11	10
1278	38.5	35	21	26	37	17	11	16	19
1279	35	30	21	17	33	12	6	17	20
1280	32.5	31.5	22	31	30	12	9	16	26
1281	31	23	16	20	25	9	11	13.5	11
1282	43	31.5	20	22	35.5	15	8	15	19
1283	36	23	40	21	24	12	5	13	11
1284	49.5	22	14	24	23	22	5	13	10
1285	20	22	13	29	23	5	6	11	13
1286	45.5	31.5	16	24	28.5	55	3	30	9.5
1287	32	13	15	32	27	13	8	8	24
1288	16	9	8	18	10	10	7	6	13
1289	52	29	21	22.5	35	24	6	19	36
1290	44	30	21	18.5	33	9	6	17	17
1291	44	30	21	18.5	33	9	6	17	17
1292	40	21.5	16	24	24	23	6	12	12
1293	36	22	15	16	22.5	13	11	16	25
1294	326	259	1077	255	497	179	475	922	582.5
1295	27	23.5	14	22.5	26	8	5	13	10
1296	30	24.5	18	16	24	6	5	13	11
1297	39.5	27	15.5	15	30	10	4	13	15
1298	30.5	20	14	25	32	6	12	11	13
1299	44	25	16	37	23.5	30	8	14	13
1300	30	31	15	41	22	7.5	5	12	25.5
1301	28.5	12	9	16	14	20	6	11	8
1302	46.5	27	17.5	28.5	29	18	8	17	24
1303	30	20	15	19	21	11.5	6.5	12	8
1304	40	25	15	25	24	22	7	13.5	12
1305	44	22	18	30.5	22	20	7	16	20
1306	81	36	26	27	40	11	14	18	11
1307	41	34	25	37	34	13	17.5	16	20
1308	29.5	27	17	17	28	8	9	15	13
1309	36	28	20	34	36	14	8	17	25
1310	34.5	25	21	21	33	12	6	14.5	18
1311	49.5	21	16	15	25	8	5	14	12
1312	659	470	4151	244	1167	384.5	1029	1319	1723.5
1313	40	29	21	23	33	17	7	18	44
1314	54	24	17	29	26.5	20	12	15	15

1315	30.5	21	12	12	21	10	7	12	9
1316	25	8	6	18	7	11	5	7	12
1317	35	30	17	17	32.5	14	10	14	15
1318	777	559	4970	303.5	1373	510	1226.5	1668.5	1874
1319	38	92.5	21	46.5	29.5	17.5	9	15	10
1320	39	28	19	41	32	17	8.5	41.5	19
1321	51	15	10	21	13	27	6	9	8
1322	42	14	7	27	13	19	6	10	19
1323	26	9.5	9	23	10	13	63.5	8	11
1324	28	12	9	25	11	11	6	7	9
1325	23	13	9	29	10	13.5	5	6	24
1326	40	14	8	24	12	25	7	9	37
1327	20	8	5	12	10	5	4	5	12
1328	22	9	8	27	9.5	10	5	6	9
1329	41	11	8	24	14	25	7	9	13
1330	22	7	10	22	5	8	5	4	13
1331	50	28	19	24	34	31.5	7	17	45
1332	36	22	18	21	24.5	21	7	13	37
1333	36.5	19	19.5	19	24.5	18	10	13	20
1334	36.5	19	19.5	19	24.5	18	10	13	20
1335	346	292.5	1222	311	565	222.5	567	1169	811
1336	42.5	31	24	21	39	15	7	33	28
1337	46	30.5	21	22	31	21	7	18	23
1338	39	36.5	44	42	45	23	13	17	22
1339	49.5	25	18	18	27	17	6	16.5	18
1340	66	30	18	27	30	21	6	18	23
1341	38	32	20	38	28	14	6	30	13
1342	51	29	16	25.5	29	20	5	16	14
1343	41	31	16	18	29	17	7	15	12
1344	66	30	18	27	30	21	6	18	23
1345	38	32	20	38	28	14	6	30	13
1346	51	29	16	25.5	29	20	5	16	14
1347	41	31	16	18	29	17	7	15	12
1348	32	22	14	22	25	10	5	11	14
1349	32	22	14	22	25	10	5	11	14
1350	34.5	31	21	22.5	33	12	5.5	15	14
1351	34	27	19	34	28	11.5	8	15	23
1352	690.5	517	4163	254	1348	463	1096	1461.5	1756.5
1353	31	21	14	20	26	11	5	13	11
1354	41	31	19	24	32	15.5	7	16	37
1355	30	28	18	23	28	11	16	13	34
1356	53	26	20	25.5	27	15	8.5	14	23
1357	70	27	20	32	35	20.5	9	17	40
1358	31	17	13	14	21	8	4	10	12
1359	52.5	53	27	43	37	34	104	19	25
1360	45	35	26	26	37	12	8	16	19
1361	34	28.5	21	26	37	15	9	17	24

1362	38	24	22	22	28	18	7	16	12
1363	34	26.5	15	12	30	14	7	15	26
1364	28	21	15	22	20	13.5	8	13	21
1365	28	21	15	22	20	13.5	8	13	21
1366	38	32	23	16.5	41	10	7	20.5	14
1367	46	24	17	27	28	17	6	15	12
1368	46	24	17	27	28	17	6	15	12
1369	36	33	18	25	31	10.5	8	16	16
1370	43	30	18	44	24	15	7	13.5	14
1371	44	23	17	26.5	27.5	28	8	14	12
1372	87	34.5	20	55.5	27	26	10	14	51
1373	54	34	21	28.5	51	19	26	20.5	23
1374	41	30	19	87	44	17.5	9	17	12
1375	37	23	15	20	28	9	7	15	15
1376	655	483	3767.5	248	995.5	397	991.5	1329	1578
1377	54	28.5	16	32	30	26.5	7.5	17	30
1378	52	40	20.5	42.5	32	23	7	17	28
1379	29.5	27	21	39	51	10	7	12	22
1380	39	35	43	50	50	12	12	19	21
1381	45	28	18	24	32	18	8	18	27.5
1382	34	29.5	17	18	29	13	10	16	12
1383	43.5	28	27	17	39	15.5	9	16	17
1384	60	28	20	28	29	25	10	21	18
1385	42.5	25	17	27	24	14.5	6	14	18.5
1386	549	452	3496.5	225	1106.5	344	902	1412	1295.5
1387	33	29	16	29.5	32	13.5	7.5	14	22
1388	47	23	15	17	25	19	18	14	23
1389	46	24	22	25	22	21	8	14	12.5
1390	48	40	20.5	26	38	17.5	8	18	19.5
1391	51	29	21	21	33	15	10	16	11
1392	41	23.5	16	26	34	16	6	14	13
1393	50	28	20	32	30	18	7	16	18
1394	15	8	6	14	7	7	7	6	13
1395	17	6	5	22	7	8	4	5	8
1396	31	15	8	27	10	17	7	9	10
1397	32	24	16.5	68	69	13	8	12	15
1398	37	22.5	15	20	27	13	6	12	15
1399	40	22	15	17	23	17	5	13	29.5
1400	48	19	17	19	23	23	6	17	17
1401	33	20	13	16	21	13	5	11	22.5
1402	54	25	14	25	24	29	6	15	27
1403	48	22	18	23	22.5	32	6	14.5	10
1404	48	25	16	28.5	26	22	7	13	19
1405	35	18.5	13.5	21	19.5	13.5	6	11	12
1406	37	20	14	19	23	19.5	8	13	19
1407	44	20	15	22	20	27	7	14	22
1408	48	22	18	23	22.5	32	6	14.5	10

1409	48	25	16	28.5	26	22	7	13	19
1410	35	18.5	13.5	21	19.5	13.5	6	11	12
1411	37	20	14	19	23	19.5	8	13	19
1412	44	20	15	22	20	27	7	14	22
1413	63.5	35	25	22	43.5	20	10	19	22.5
1414	51	26	19	19	31	20	7	19	11
1415	51	26	19	19	31	20	7	19	11
1416	31	22.5	15	23	25	14	6	15	10
1417	31	22.5	15	23	25	14	6	15	10
1418	31	35.5	18	31	40.5	14.5	8	15	21
1419	42	20	14	19	23	16	5	13.5	22
1420	42.5	24	17	23	28	16	6	15	14
1421	38	29	19	25	30	14	7	15	35
1422	57.5	29	17	24	28	16	13	15	25
1423	73	25	18	19	26.5	24	8	18	18
1424	39	25	16	12	28	20.5	4	16	10
1425	31	23	13	23	30	9	32	12.5	18
1426	42	29.5	20	36	37	14	9	17	23
1427	13	9	9	5.5	2.5	7.5	0	17.5	2
1428	59	28	22	18	36	25	7	19	21
1429	45	37	20	27	38	20	11	18	28
1430	39	33	19	26	32	29	10.5	21.5	35
1431	37.5	25	22	20	33	15	10.5	12	15
1432	42	33.5	19	29	29	19	14.5	14	16
1433	33	25	17	30	26	21	8	16	26
1434	53	30	23	32	37	33	6	16	31
1435	50.5	30	20	39	35.5	30	8	18	24
1436	41	30	19	44	39	32	24	18	23
1437	42	30	21	24	36	21	8	16	35
1438	55	25	24	25	30	26	7	16	49.5
1439	48.5	40	23	41	103.5	23	17	18.5	32
1440	52	23	20	24	29	36	8	17	18
1441	50	27	18	31	38	23	8	17	23
1442	52	30	16.5	35	26	34.5	7	13	23
1443	45	25	16	26	24	25.5	7	12.5	13
1444	42.5	20	17	21	29	20	8	13	12
1445	45.5	22	13	26	26	23	8	13	37
1446	49	29.5	20	23	30	16	14	15	20
1447	36	22.5	19	11	31	8	5.5	14	13
1448	48	26	18	26	51	15	10	14	11
1449	55	56	18	26	44	17	18	16.5	9
1450	61	24	16	25	26	26	10	17	18
1451	47	22	16	21.5	40.5	21	7	17	17
1452	42	24	17	27	26	16	7	14	17
1453	51	22	15	17	23	20	6	16	10
1454	41	27	22	22	26	15	8	16	21
1455	42	23	21	32	26	19	20.5	16	35

1456	30	12	10	29	25	17	10	6	15
1457	26	5	4	11	5	13.5	3	5	9
1458	24	25	15	18	28	7	11	15	13
1459	36	13	9.5	26	13	15	7	10	22.5
1460	44	10.5	7	23	8	18	4	7	15
1461	510	462	3545	184	951.5	295	967	1444	950
1462	37.5	9	8	15	25	13	9	6	6
1463	43	24	17.5	9	30	9	26	15	9
1464	35	28	16	68	22	11.5	6	13	11
1465	67.5	21.5	16	25	25	36	8	14.5	22
1466	43.5	24	17	31.5	25	23	8	13	27
1467	28	24	18	19	22	12	7	12	15
1468	52	22	13	24.5	24	37	5	16.5	20
1469	42.5	20	16	25	22	23	10	14	18
1470	35	22	14	21	23	20	6	13	15
1471	41	21	16	21	22	20	6	14	12.5
1472	42.5	20	16	25	22	23	10	14	18
1473	35	22	14	21	23	20	6	13	15
1474	41	21	16	21	22	20	6	14	12.5
1475	58	34	25	17	37	23	8	17	15
1476	57.5	35	35	30	38	31	8	24	22
1477	56	31	21	23	35	24	8	18	31
1478	36	30	21.5	22	45	18	8	17	47.5
1479	56	29	19	15	34	21	7	17	13
1480	37	27.5	21	25	29	15	9	16	17
1481	32	26	18	14	27	8	4	16	18
1482	114	24	19	13	34	41	5	16	9
1483	55	24	19	16	33	22.5	7	15	16
1484	36	25.5	19	21	27	11	6	15.5	14
1485	45	22	14.5	20	21	20	6	15	17
1486	45	22	14.5	20	21	20	6	15	17
1487	36	29	20	27	35	12	7	15	16
1488	42	28	24	52	28	17	7	16	15
1489	42	24	21	23	29.5	18	8	21	45
1490	57	23	17	20	30	18	6	16	15
1491	44	20	15	20	26	17	5	13	11
1492	39	22	15	22	24	19	5	13	12
1493	722	511	3855	246	1255.5	447	1025	1425	1613
1494	69	25	15	13	26	15	7	15	22
1495	62	26	16	29	25	23	6	18	12
1496	46	44.5	37	39	59	19	37	31	39.5
1497	56.5	31	22	40.5	29	21	10	20	29
1498	57	30	20	22	34	28.5	9	21	21
1499	39	30	27	31	43	18	9	16	16.5
1500	22	16	14	11	22	6	6	12	5
1501	55	27	25	32	33	24	9	15	27
1502	78.5	25.5	17	17	29	35	6	17	23

1503	46	33	19	47	34	15.5	6	17	12
1504	46	33	19	47	34	15.5	6	17	12
1505	29	26	15	25	35	9	4.5	13	20
1506	29	26	15	25	35	9	4.5	13	20
1507	35	24.5	17	22	27.5	15	8	15	14
1508	40	25	15	27	25	13	5	14	15
1509	49	34	21	27	44	27	8	18	67
1510	35	23	13	19	24	8	7	12	13
1511	45	27	19	29	42	21	9	17.5	20
1512	40.5	46.5	24	65	27	17	29.5	17	15.5
1513	666	525.5	3993	241	1175.5	434.5	1009	1482	1630.5
1514	52	27	19	29	35	24	7	18	13
1515	696.5	519	4097	260	1139	462	1092.5	1562	1791.5
1516	55	25	18	25	31.5	29	9	16	15
1517	35	26	16	24	25.5	29	7	14	19
1518	45	24	18	22	25	28	7	16	14
1519	35.5	32	18	36	34	15.5	10	14	14
1520	589	473	3369	229	1037	399	984	1441	1465
1521	36	30	22	30	38	9.5	12	18	21
1522	-1	26	14	26	45.5	21	-1	12	27
1523	54	27	18	25	31	15.5	7	15	26
1524	44.5	25	19	13	30	19	11	14	63
1525	53.5	24	21	24	30	26	7	18	24
1526	27.5	22	19	37	23	12	9	10	12
1527	26	8.5	6	21	19	11	6	5.5	12
1528	38	114.5	21	34	58	18	33	18	22
1529	48	29.5	19	41.5	30	25	8	16	35
1530	20	10	9	27	7	8	6	6	30
1531	42	10	7	25	11	24	6	8	19
1532	28	18.5	7	35	10	16	14	7	19
1533	18	14	12.5	39	12	11	7	16	43
1534	38	10	7	24	12	14	8	7	13
1535	18	16	19	47.5	12	9	21	6	15
1536	38	10	7	25	7	29	6	9	14
1537	29	4.5	5	13	6	14	4	5.5	6
1538	44.5	20	12	19	22.5	27	6	15	11
1539	44.5	20	12	19	22.5	27	6	15	11
1540	38	35	22	17	37	13	7	19	19
1541	56.5	35	27	23	39	24	9	20	17
1542	61	32	29	24	37	30	8	18	22
1543	53	45	25.5	19	38	23	7	19	17
1544	53	27	23	23	33	20	16	16	16
1545	43	29.5	20	20	34	18	5.5	19	16
1546	56	29	17	24	32	22	6	17	12
1547	47	25.5	20	25	32.5	10	7	15	25
1548	36	29	15	21	30	16	5	16	15
1549	42	29	19	20	30	14	6	17	20

1550	35	23.5	16	13	27	11	5	15	11
1551	49	28	17	20	29	24	7	18	14
1552	46	25	16	24	28	14	5	16	16
1553	43	29.5	20	20	34	18	5.5	19	16
1554	56	29	17	24	32	22	6	17	12
1555	47	25.5	20	25	32.5	10	7	15	25
1556	36	29	15	21	30	16	5	16	15
1557	42	29	19	20	30	14	6	17	20
1558	35	23.5	16	13	27	11	5	15	11
1559	49	28	17	20	29	24	7	18	14
1560	46	25	16	24	28	14	5	16	16
1561	844	566	4847	300	1261	527.5	1258.5	1638.5	2061.5
1562	46	22	15	36	23	17	6	18	18
1563	844	566	4847	300	1261	527.5	1258.5	1638.5	2061.5
1564	46	22	15	36	23	17	6	18	18
1565	696	545.5	4126	258	1317	447.5	1135	1541	1653.5
1566	40	33.5	24	26	30	13	8	16	18
1567	49	28	20	26	34	17.5	7	17	15
1568	30	31	23	23	29.5	8	18	15	17
1569	54.5	27	16	26	29	25	6	17	17
1570	46	35	22	36	37	25	8	16	58
1571	37	9	7	21	6	16	7	8	10
1572	51.5	29	21	39	31	46	17	17.5	27
1573	48.5	30.5	17	29	26	42	21	15	48.5
1574	51.5	29	21	39	31	46	17	17.5	27
1575	48.5	30.5	17	29	26	42	21	15	48.5
1576	58	29	21	16.5	35	25	9	19.5	18
1577	51	29	19	24	33	23.5	7	15	33.5
1578	24	20	15	24.5	23.5	7	8	12	10
1579	53	27.5	19	25	35	27	7	18	15
1580	52	29	16	30	32	25	6	17	14
1581	46	19	20	26	22	19	9	13	34
1582	49	24	16	37.5	23	31.5	7	15	26
1583	34	26	14	35	34	10	6	12.5	25
1584	36.5	28	18	29	31	16	8	16	18
1585	44	27.5	19	24	28	19	9	19	24
1586	28	24	19	11	43.5	9	6	15	12
1587	42.5	24	15	24	28	19	7	16	27
1588	50	30	23	26.5	41	22	10	18	17
1589	59	43	30	24	47	12	8	21	46
1590	55	25.5	16	26	31	23	8	16	17
1591	58.5	23	16.5	36	25.5	26	8	15	19
1592	40	28.5	16	17.5	25	16.5	6	14	49
1593	27	26.5	17	26	28	10	6	13	14
1594	57	32	18	27	29	27.5	6	16	36
1595	32	27	19	23	29	21	18	16	24
1596	55	28	19	32	31.5	25	9	18	15

1597	49.5	29	19	27	79	26	12	19	28
1598	36	28	19	22	34	15	14	15	22
1599	21	8	5	23	7	6	4	5	38
1600	37	14	12	25	13	19	9	10	24.5
1601	26	14.5	13	30	12	12	9.5	7	15
1602	35	8.5	6	18	7	13	5	7	11
1603	40	22.5	14	24	28	21	10	14	24.5
1604	43	19	13.5	17	23	21	7	13	16
1605	41	19	16	21	21	24.5	7.5	12	12
1606	41	19	16	21	21	24.5	7.5	12	12
1607	37	29	21.5	17	33	16.5	8	17	16
1608	58	27	23	17	32	23	14	15	43
1609	43	25	17	14	31	17	5	16	13
1610	54.5	34	24.5	22	39	20	9	20	14
1611	54.5	34	24.5	22	39	20	9	20	14
1612	34	30	16	34.5	24	9	6	14	13
1613	50	22	16	24	28.5	17	7	15	11
1614	34	30	16	34.5	24	9	6	14	13
1615	50	22	16	24	28.5	17	7	15	11
1616	30	28	20	22	34	9.5	6	16	19
1617	50	28	19	23	290	23.5	6	17	17
1618	47	29.5	21	30	43	17	8	18	20
1619	652	515.5	4068.5	240	1133	402	969	1457.5	1586.5
1620	32	21	15	11	26	10	5	14	12
1621	29.5	21	15	8	22.5	8.5	4.5	12	10
1622	53	60	16	112	24.5	20	15.5	16	33
1623	51	30	18	27	29	26	10	16	21
1624	40	8	8	21	6	24	6	9	19
1625	18	8	6	21	28	10.5	5	6	10
1626	35	11	5	23	13	16	7	7	12
1627	33	21	16	21	26	14	7	13	22
1628	65	34	24	23	35	29	7	19	36
1629	56	26	20	16	32	32.5	6	18	15
1630	39	24	18	14.5	27	17	7	15	12.5
1631	44.5	29	21	25	39	17	16	21	18
1632	25	26	18	26.5	213	8	13.5	15	14
1633	93	23	16	15	27	10	5	16	22
1634	49	29	16	37	31	25	10	21	19
1635	652	468	3648	236	1001	359	975	1355	1567
1636	55	31	22	28	33	21	9	17	18
1637	52	29.5	20	24	36	23.5	9	17	17.5
1638	53	30	22	31	35.5	22	8	17	40
1639	67	24	16	28	27	43	8	16.5	26
1640	38.5	23.5	20	23	25	17	7	13	19
1641	575	487	3440	212	1061.5	368	919	1428.5	1386
1642	45	28	16	24	33	18.5	24	17	17
1643	67	21.5	15	23	28	26.5	7	37	15

1644	60	24	18	22	29	26	7	19	15
1645	160	36	23	28	37	23	10	20	26
1646	36	31	25	42	91	23	15	15	13
1647	29.5	43	15	23	27	13	11	16	9
1648	42	22	15	22	28	15.5	6	14	14
1649	49.5	27	19	27	28.5	21	10	17	29
1650	23	16	15	12	25	7	7	12	5
1651	41	29.5	20	31	28	15	16	17	51
1652	46	25	17	33	32	21	9	14	16
1653	57.5	32	25.5	41	49	21	12	18	22
1654	84	32.5	20	53	33	13	11	17	23
1655	52	24	17	33	24	22.5	8	16	14
1656	114.5	32.5	22	19	35	14	13	17	15.5
1657	38	25	17	18	26	7	7	14	13.5
1658	46	11	10	29	11	20	11	9	15
1659	23	12	11	24	8	10	6	7	17
1660	45.5	10	8	21	324	20	5.5	8	15
1661	40	13	8	30.5	12	20	8.5	9	16
1662	42	27	16	23	30	16	18	17	25
1663	45	21.5	14.5	22	31	25	8	15.5	20
1664	63	22	16	25	28	30	7	16	15.5
1665	50.5	22	20	30	24	25	6	14	20
1666	48.5	21	15	21	24	31	7	12	18
1667	50.5	22	20	30	24	25	6	14	20
1668	48.5	21	15	21	24	31	7	12	18
1669	55	31	26	23	42.5	24	8	19	29
1670	50	35	23	22	33	20	7	18	31
1671	44	28	20.5	21	36	18	7	16	40
1672	45	30	17.5	21	32	16	7	18	28
1673	45	30	17.5	21	32	16	7	18	28
1674	66.5	31	20.5	27	41.5	24	8	18.5	39.5
1675	42	35.5	24	38.5	38	15	8	17	17
1676	51	29	18	24.5	31.5	22	7	17	21
1677	65	26	35	20	30	21.5	6	17	23
1678	35	56	21	56.5	38.5	18	18.5	21	28
1679	28	25	14	28	25	7	7	12	12
1680	54.5	33	23	38	37.5	31	11	22	41
1681	41	42	22	89	29	11	37.5	15	19
1682	50.5	26	19	25	31.5	19	8	16	14
1683	35	12	10	30	10	7	7	7	10
1684	40	12	8	26	10	22	7	9	10
1685	51.5	21	17	25.5	24	29	6	15	18
1686	51.5	21	17	25.5	24	29	6	15	18
1687	53	31.5	20	27	33	23.5	18	16	39
1688	32	32	21	32	34	18	23	14	37.5
1689	54	25	15	26	26	30.5	7	16	17
1690	30	27	18	33	27	14	10	15.5	19

1691	32	25	16	26	33	16	8	16	13
1692	56	37	38	42.5	115	22	15.5	30.5	17.5
1693	33.5	23	19	54	199.5	13	51	14	21
1694	56	28	20	20	36	24	9	20	27
1695	45	29.5	20	33	30	24	8	16.5	28
1696	35	42	48	35	33	30	35	27	41
1697	62.5	33	21	34	35.5	31.5	9.5	19	24
1698	40	29	20	35	41	12.5	9	13	37
1699	46.5	22	15	22.5	26	19	7	14	15
1700	31	20.5	18	24	29	17	6.5	11	16
1701	40	28	19	27	31	16	7.5	15	26
1702	45	24	18	27	28	18	9	15	24
1703	46	22.5	19	25	25.5	14	8	13	45
1704	48	27	18	31	31	19	7	17	15
1705	68	43	27	36.5	54.5	30	11	25	32
1706	37	24	21	14	28	12	10	13	16
1707	32	22	19	15	24	9.5	5	11	71.5
1708	837	600	4797	299	1414	555.5	1227	1658	1943
1709	38	32	25	37	41	21	42	24	39
1710	43	12	10	23	13	16	8	10	27
1711	35	12	7.5	18	16	17	7	7	28
1712	101.5	10	19.5	27.5	16	18	10	7	12
1713	38	10	10	29.5	8	18	8	9	12
1714	36	9	6	23	6	24	7	9	18
1715	48	11	8	24	7.5	29	7	12	11
1716	21	6	5	15	12	9.5	4	5	10
1717	43	22	15.5	23	26	15	49.5	13	21
1718	49.5	20	15	15	23	26	6	15	12
1719	54	33	23	18	46	19	7	19	15
1720	67	33.5	24	20	34	17	9	17.5	14
1721	59	29	32	25	114.5	28	7	18	29
1722	60	27	22	19	32	25	7	17	21
1723	57	31	19	28	28.5	26	8	16	17
1724	72	24	15.5	31	26	32	7	21	12
1725	50.5	22	17	23	25	20	5	15	12
1726	35	22	17	24.5	22	10	7	13	17
1727	57	31	19	28	28.5	26	8	16	17
1728	72	24	15.5	31	26	32	7	21	12
1729	50.5	22	17	23	25	20	5	15	12
1730	35	22	17	24.5	22	10	7	13	17
1731	49	28	18	26	34	21	7	19	35
1732	46	26	19	21	30	16	6	16	77
1733	58	24	17	27.5	28	25	8	16	42
1734	63	39	23	35	85	25	10	21	31
1735	66	39	26	32	37	30.5	10	24	40
1736	34	22	15	15	21	8	5	11	11
1737	30	26	15	25.5	23	9	8	13	26.5

1738	713.5	482	4001	246	1225	451	1039	1380	1582
1739	37	30	19	30	41	17	7	20	45
1740	61	24	25	28	32	26	8	18	19
1741	62	24	23	24	39	25.5	6.5	16	16
1742	43	26	17	20	61	18	11	14	24
1743	32	13	7	18	19	14	5	7.5	16
1744	43.5	24	22	26	27	16	8	15	22
1745	63	30	21	21.5	31	28	7	21	28
1746	52	36	19	25	34.5	19.5	9	19	18
1747	52	36	19	25	34.5	19.5	9	19	18
1748	37	21	28	9	26	12	5	12	25
1749	73.5	32	20.5	32	33.5	25	10	19	23.5
1750	64.5	37	27	51	42	24.5	18	22.5	31
1751	43	29	28.5	27	34.5	28.5	8	18	30
1752	52	23	16.5	33	25	28	8	15.5	31
1753	63	29	19	33	37	29.5	9	17.5	18.5
1754	41	38	37	35	34	33	34	35	47
1755	38	41	27	62	44.5	19	13	15	63
1756	58	32	18	25	37	27	8	18.5	14
1757	64	28.5	18	35	34.5	31	9	17.5	52
1758	51.5	23	18	21	27	22	8	17	19
1759	61	30	20	21	41	21	6	18	35.5
1760	57	26	17.5	28	27	20	6	15	13
1761	42	24	15	18	22	16	6	15	20
1762	64	22.5	18	34	30	30.5	10.5	18	25
1763	31	27	22	18	28	13	7	13	11
1764	48.5	28	16	43.5	28	18	7	15	17
1765	49	24	17	24	26	23	12.5	16	13
1766	57	15	10	30	12	24	8	10	16
1767	25	12	10	32	11	17	8	11	16.5
1768	693	517	3633	237	1136.5	448.5	1050	1437.5	1466.5
1769	38	24	16.5	21	32	12	9	15	13
1770	56	20.5	16	20	22.5	39	7	13	19
1771	42	25	16	21	20	18	6	11	22
1772	51.5	22	16	28.5	21	23	8	14	16
1773	55	19	29	29.5	25	30	9	15.5	21.5
1774	42	25	16	21	20	18	6	11	22
1775	51.5	22	16	28.5	21	23	8	14	16
1776	55	19	29	29.5	25	30	9	15.5	21.5
1777	636	591	4235.5	261.5	1186.5	487.5	1099	1589.5	1697
1778	55.5	37	27	25	41	21	10	20.5	14
1779	62	31	23.5	18	34.5	30	8	19	15
1780	56	40	26	32.5	39	22	9	21	15
1781	56	40	26	32.5	39	22	9	21	15
1782	30	23	15.5	26	24	9	5	12	13
1783	30	23	15.5	26	24	9	5	12	13
1784	49	31	24	27	33	23	7.5	18	15

1785	52	29	19	25	44	17	9	17	18
1786	60	23	15	29	27	29	6	17	16
1787	43	32.5	22	29.5	35	14	10.5	16	25
1788	52	27	22	46.5	33	22	10	16	38
1789	39	31	15	64.5	28.5	13	6	14	9
1790	30	26	16	23	25	12	7	14	11
1791	63	20	15	22	28	31	7	16	14
1792	38	24	20	32	27	19	7	13	17
1793	59.5	29	21	32.5	30	30	11	22	21
1794	50	26	19	24	25.5	25	8	18	38
1795	30	26	17	43.5	24	9	7	14	17
1796	30	26	17	43.5	24	9	7	14	17
1797	54	29	22	23	37	23	8	19	20
1798	42	20	18	19	26	19	6	12	10.5
1799	49	34	26	44	39.5	25.5	13	17	27
1800	70.5	31	21	44	34	39	9	21	16
1801	56	30	18	46	30	27	11	16	38
1802	37	35.5	19	70	64	18	12	17	102
1803	60	27	18	38	31	30	9	21	23
1804	53	25	18	27	27.5	25	7	15	55
1805	48.5	23	18	28	24	24.5	7.5	16	13
1806	37	23	13	17	24	15	9	13	13.5
1807	39	59	24	23	35	18	10	17	19.5
1808	66	30	21	26	37	20	9	19	31
1809	36	30	17	47	34	14	10	15	57
1810	36	58.5	24	26	49	13	12	16	28
1811	35	19	16	20	27	14	10	13	11
1812	48	24	17	21	24.5	18	8	17	23.5
1813	73.5	33	23.5	30	35	47	34	16	18
1814	28	10	7	24.5	8	8	5	6	47
1815	81	13	9	32	14	43	7	13	52
1816	36	10	7	22	14.5	19	6	7	15
1817	31	9	6	22	9	13	5	6	9
1818	33.5	8.5	8	22	9	21	7	9	17.5
1819	64	35	20	21.5	40	30.5	9	18	21
1820	51.5	21.5	16	30	23	24	8	13	15
1821	57	24	16	26	25	32	13	15	20.5
1822	39	19	14	24	20	16	6	12	27
1823	44	25	15.5	31	24	27	7	17	15.5
1824	39	21	15	16	23	17	6	13	18
1825	54.5	21	16	29	25	30	7	15	31
1826	30	19	17	22	21	16	5	12	17
1827	58	20	16	19	28	29	6	12	17
1828	22	20	14	9	18	8	5	12	13
1829	39	21	15	16	23	17	6	13	18
1830	54.5	21	16	29	25	30	7	15	31
1831	30	19	17	22	21	16	5	12	17

1832	58	20	16	19	28	29	6	12	17
1833	22	20	14	9	18	8	5	12	13
1834	74	36.5	24	19	37.5	35	8	20	16
1835	43.5	25	17	13	30	13	6	16	19
1836	61	29	22	20	34	28	7.5	20	17
1837	38	27	17.5	28	36	13	10	16	20
1838	46	29	32	24	28	18	13	15	20
1839	61	25	18.5	28	28	28.5	8	15	15
1840	52	21	15	23	24	21	7	15	17
1841	71.5	25	20	23	27	19	8	16	26
1842	75	27	20	37	42	27	7	19	14
1843	54	26	20	35	45	25	9	18	19
1844	63	24	16	22	26	31.5	8	14	12
1845	56	24	17	26	28	37	7	16.5	17
1846	69	34	22	28	36	30	9	20	17
1847	53	26	17	18	29.5	17	7	15	53
1848	47.5	23	20	30.5	30	16	10	13	38
1849	40	8	6	24	10	20	5	10	38
1850	79	57	28	36	58	35	26	22	31
1851	54	13	8	33.5	15.5	23.5	7	9	28
1852	89	38	21	38	42	33.5	11	20	79
1853	28	27.5	18	23	28	16	19	15	30
1854	35	22	17	30	30	12	6	12	11
1855	59	28	18	35	31	28	12	18	20
1856	35	58	20	57.5	39	12	12	13	102
1857	46	30	19	14	37	9.5	5.5	17	14
1858	112	28	16	14	40	33	7	15	16
1859	60	31	21	27	38	35	10	22	19
1860	65	33	20	35	41	34	9	22	19
1861	48	28	18	22	30	20	8	17	25
1862	56.5	28	21	34	28	28.5	10	16	30
1863	58	23	18	22	55	31	7	15	14
1864	774	531	4076	242	1166	436.5	1048	1515	1730
1865	32	20	14	26	23	14	7	16	23
1866	38.5	26	16	19	27	12	16	13	9
1867	75.5	26	17	26	25	34	8	16	38
1868	52	31	21	23	31	21	7	16.5	26
1869	62	25	19	27.5	24.5	23.5	12	17	24
1870	102	39.5	31.5	48	44	37	12	25	37
1871	32	11	9	27	9	14	9	6	9
1872	33	9	7	19	18	12	4	5	12
1873	870	572	4688	251	1326	504	1278	1742	2024
1874	23	14	12	31	10	10	11	6	16
1875	26	9	6	25	10	12	6	6	34
1876	48	8	31	15	25	13	9	5	5
1877	20	41	27.5	45	7	31	34	13	62
1878	64	27	19	29	35	37	7	20	21.5

1879	65	22	16	24	24	31	9	16	18
1880	49	22.5	18	21	21.5	21	7	14	13
1881	58	24	15	26	22	27	8	15	15
1882	58	24	15	26	22	27	8	15	15
1883	67	33	27	22	38	30.5	8	20	17
1884	65	39	30	39	45	34.5	12	21.5	67
1885	56	32	26.5	24	41	17	7	19	20
1886	88	35	26	24	38	25	10	22	44
1887	54	21	18	13	26	26	6	18	14
1888	36	25	20	21	32	9	14	15	22
1889	65	29	22.5	20	31	25	7	17	30
1890	48	28	22.5	17.5	35	17	7	18	10
1891	55	33	22.5	28	37	23	8	21	29
1892	58.5	35	25	31	37	28.5	8	22	18
1893	62	36	21	35	46	25	8	19	13
1894	40	31.5	21	22	45.5	13	7	17	21
1895	48	28	22.5	17.5	35	17	7	18	10
1896	55	33	22.5	28	37	23	8	21	29
1897	58.5	35	25	31	37	28.5	8	22	18
1898	62	36	21	35	46	25	8	19	13
1899	40	31.5	21	22	45.5	13	7	17	21
1900	36	22	17	24	25	10	8	13	19
1901	36	22	17	24	25	10	8	13	19
1902	59.5	27.5	20	31	29	25	8	16	34
1903	52	20	16	21	23	19	5	14	13.5
1904	45	32	20	23	32	15	6	16	17
1905	35	24	17	16.5	23	10.5	7	13	11
1906	50	37	25	48	38	29	11.5	17	73
1907	51	25	19	37	27	35	9.5	15	19
1908	40	23	38	38	27	28	10	14	100
1909	37	30	21	40	360	15	12	14	55
1910	56	21	17	24.5	56	23	8	14	15.5
1911	48	28	21	29	35	27	7	18	19
1912	38	30	21	29	43	16	14	14	17
1913	57	12	8	21	10.5	25	6	8.5	12
1914	28	12	9	27	8	12.5	6	14	14
1915	41	20	19	17	23	13	8	13	10
1916	52.5	26	20	32	26	15	7	15	32.5
1917	62.5	31	19	38	30	30	9	18	19
1918	57.5	36	24	31	43	24	9.5	20	22
1919	29	35	19	50.5	130	17.5	10	17	62
1920	48	27	18	27	27	25	9	17	20
1921	48	26	19	23	38	20.5	7	18	14
1922	49	30	13	18	37	29	2	-1	18
1923	40	27	18	24	32	14.5	7	15	38
1924	64	22	18	26	24.5	28	8	17	15
1925	76.5	31	18	48	60	12	11	18.5	11.5

1926	40	29	25	28	49.5	15	11	16	13
1927	48	27	20	29	30	20	8	15	26
1928	63	30	20	37.5	37	30.5	10	20	31
1929	59	21	17	14	26	11	9	13	39
1930	73	28	21	43	42	30	8	17	16
1931	73	25	17	27	29	32	7	17	12
1932	60	24	17	29	27	27.5	8	17	20
1933	66	23.5	16	30	27	34	7	17	16
1934	61.5	24	25	21	27	27	8	20	40
1935	63	28.5	19	33	30	34	8	19.5	17
1936	35	11	9	23	9	16	7.5	7	20
1937	116	35	18	32.5	77.5	44	39	10	13.5
1938	43	8	6	26	9	22	5	9	22
1939	45	11	8	29	9.5	21.5	7	11	13
1940	53.5	24	17	22	23	29	7	17	17
1941	46	19	15	18.5	24	19	6	13	19
1942	60	19	15	22	22	30	7	16	19
1943	46	19	15	18.5	24	19	6	13	19
1944	60	19	15	22	22	30	7	16	19
1945	67	32	23	22	38	26	7	19	20
1946	55.5	24.5	15	29	32	26	8.5	17	18
1947	72.5	38	23	41	41	32	11	21.5	48
1948	45	25	16	13	23	11	4	13	20
1949	67	30	20	27	36	19	8	22	20
1950	39	21	16	17	33.5	9.5	6.5	12	9
1951	104	36	20	30	39	50	10	22	34
1952	55.5	29	23	29	35	24.5	9	18	23
1953	65.5	27	18	34	33	37	9	20	21
1954	56	25	17.5	26	32	26.5	8	18	16
1955	81	43	27	51	53.5	35	12	24	31
1956	43	12	9	28.5	10	19	8	9	14
1957	50	44	20	24.5	22	21	10	14	25
1958	52.5	23	15	21	23	29.5	6	14	24
1959	56	31	23	28.5	33	20	12	18	19
1960	60	31	21	30	41	24.5	9	18	13.5
1961	88	42.5	25	43.5	43	43	12	24	26
1962	78	40	23	44	40	57.5	13	19	41
1963	38	21	15	18	25	9	5	12	35
1964	55	26	16	33	36	38	10	16	24
1965	71	25	17	29	30	33.5	10	18	17
1966	60	35	19	41.5	39	26	9	18	21
1967	62	28	21.5	32	35	22	8.5	18	21
1968	36.5	26	20	22.5	36	22	7	14	19
1969	39	23	18	19.5	29.5	20	8	15	17
1970	52	21	18	26	25	19	7	12.5	16
1971	61.5	21	15	20	23	28	7	14	19
1972	47	34	20	28	37	20	8	18	21

1973	63	31	21	21	35	29	11	20	20
1974	57	32	23	42	39	31	16	44	33
1975	841	562	4368	269	1285	537	1147	1472	1929
1976	76	28	20	20	33	32.5	10	20	13
1977	73	30	21	34	39	31.5	26	36	40
1978	40	26	18	22	23.5	11	7	14	51
1979	43	24.5	17	27	28	13	8	14	40
1980	47	26	22	19	31	14.5	12	21	21
1981	58.5	29	23	31	27	22.5	9	19	23
1982	84	16	12	29	17	40	9	15	19
1983	27	20	25	54.5	16	14	21	10	30
1984	38	16	11	29	42	11	8	7	13
1985	39	8	7	16	6	17	6	6	11
1986	80	33	21	43	39	42	9	20	16
1987	63	30	20	28	55	27	10	19	25
1988	77.5	30	18	29	33	42.5	8	26	27
1989	59	23	21	22	30	34	10	17	18
1990	55	26	17	21	32	35	7	17	20
1991	63	21.5	16	22	33	46	7	17	12
1992	47	18	17	19	23	24	8	14	21
1993	52.5	19	15	20	32	22	6	15	15.5
1994	52.5	19	15	20	32	22	6	15	15.5
1995	895	606.5	5038.5	304	1418	594.5	1415	1687	2152
1996	92	35	31	23	44	53	10	23	34
1997	69	31	23	20	39	34	11	22	18
1998	65	26	21	18	32	26.5	9	17	28
1999	49	27	18	32	32	21	7	16	35
2000	49	27	18	32	32	21	7	16	35
2001	37.5	31	18	18	30	12	5	17	17
2002	49	21	14	15	26	13.5	4	14	14
2003	54.5	29	20	31	30	16	8	14	20
2004	52	27.5	18	27.5	25.5	19	7	16	28
2005	59	25	17	33	24	25.5	9	16	32
2006	40	33	16.5	44	27	9	6.5	13	28
2007	62	26	19	35	30	32	10.5	18	20
2008	38.5	19	17	24	22	16	6	11	15
2009	38.5	19	17	24	22	16	6	11	15
2010	109	43	38	57	41.5	56	12	24	20
2011	72	36	26	28	38	32	12	21	18.5
2012	78	31	22	31	35	40.5	9	22	20
2013	93	45	27	37	40	35.5	11	25	21.5
2014	50	33	22.5	33	34	22	11	19	35
2015	68	27.5	30	30	41	40	9.5	20	20.5
2016	37	38	19	39	34	18	37	16	26
2017	66	35	27	37	42	24	13	19	52.5
2018	61.5	26	15	33	25	33	22.5	24	33
2019	52	23	17	20	26	21	7	14	17

2020	43	21	16	20	20	17	6	12	15
2021	56	28	22	28.5	29.5	23	10	21.5	29
2022	61.5	25	17	25	33	28	8	18	19
2023	75	22.5	16	29	28	32	7	18	26
2024	75	26	18	42.5	31	41	10	22	52
2025	44	32	16	27	24	11	6.5	13	27.5
2026	107	23	26	15	82	48.5	13	15.5	15
2027	58	10	8	23	15.5	30	12	12	21
2028	55	35	24	38	43	23.5	11	21	29
2029	65.5	30	21.5	23	27	30	8	17	20
2030	16	12	6	22	15.5	6	6	6	8
2031	47	16	9	27	10	22	7	9	14
2032	45	9	9	25.5	12	20	8	9	29
2033	62	28	21	16	37	26	6	18	55
2034	54	31	20	35	34	15	7	17	16
2035	46	27	20	24	29	16	4	16	19
2036	81	32	23	42	33	38	7	20	16.5
2037	54	31	20	35	34	15	7	17	16
2038	46	27	20	24	29	16	4	16	19
2039	81	32	23	42	33	38	7	20	16.5
2040	95	25	17	20	28	26.5	7	15	22
2041	35.5	22	14	22	22	15	9.5	13	11
2042	34	8	6	20	15	13	6	5	16
2043	45	27	20	26	32	13	11	15	17
2044	46	31.5	20	40	85	22	8	17	32
2045	58	28	30.5	37	33	34	13	17	19
2046	77	23	16	22	28	41	6	18	24
2047	46	32	18	32	36.5	37.5	13	17	21
2048	71	26	17	32	31	38	8	27	37
2049	85	28	18	27	31	43	9	18	15
2050	54	28	16	28	27	22.5	8	17	16
2051	62	33	20	46	34.5	41	10	20	37
2052	92	25	18	38	28	41	10	21	17
2053	86	29.5	17	46	27	43	8	18	28
2054	66.5	23	18	29	28	42	7	18	19
2055	125	187.5	69	99	31	191	74.5	73	64
2056	61.5	29	21	26.5	34	34	9	22	16
2057	74	40	27	42	44.5	39	14	24	39.5
2058	46	31	16	29	25	19	6	14.5	31.5
2059	62	23	16	25.5	26	31	6	17	16
2060	68	35.5	26	34	47	27	9	21.5	31
2061	74	31	24	36.5	36.5	32.5	9	18	27.5
2062	54	13	9	24	13	31	8	12	19
2063	54	10	10	27	9	29	6	10	10.5
2064	54	9	8	23	11	22	6	10	19
2065	73	24	18.5	26	30	36	7	19	17
2066	65	23	14	25	22	32	8	15	16

2067	61.5	20	15	23	28	27	7	16	16
2068	50.5	23	15	21	23	33	7	14	18.5
2069	37	19	14	20	23.5	11	6	12	17
2070	40	20	16	19	26	25	6	14	18
2071	50.5	23	15	21	23	33	7	14	18.5
2072	37	19	14	20	23.5	11	6	12	17
2073	40	20	16	19	26	25	6	14	18
2074	63.5	36	28.5	24	37	12	24	17	97
2075	76	28	22	23.5	34	28	8	17	23
2076	53	29	22	25.5	32	18	8	17	28
2077	52	31	23	25	31	20	10	17	17
2078	53	29	22	25.5	32	18	8	17	28
2079	52	31	23	25	31	20	10	17	17
2080	43.5	22	18	29	27.5	18	8	14	16
2081	43.5	22	18	29	27.5	18	8	14	16
2082	52.5	27	20	26	39.5	26	9	20	16
2083	63	26	17	21	28	23	6	16	13
2084	78	30.5	21	26	36	26	9.5	19	15
2085	62	30.5	19.5	24	38	31.5	9	19	19
2086	68	23	19	32	27	40.5	9	20	25
2087	36	22	14	13	57.5	6	5	12	14
2088	50	31	21	39	35	30.5	14	23	75.5
2089	69	25	16.5	26.5	31	34	9	18.5	23
2090	71	24	20	30	35	35	8	22	38
2091	76	31	19.5	31	56	41.5	9	21	34.5
2092	39.5	20.5	15	18.5	28	19.5	6	11	32.5
2093	78.5	22	15	44	24	38.5	8	13	32
2094	78.5	22	15	44	24	38.5	8	13	32
2095	59	29	19	28	27	22	8	17	20
2096	59.5	29	24	38	33.5	28	13	19	23
2097	47	25	19	28	29	18	9	14	17
2098	59	23	15	32	30	32	8	16	24
2099	45	26	18	44	27	36	8	14	36
2100	23	27	15	33	23	6	4.5	12	13
2101	111.5	35	23	36	41	71.5	11	30.5	31
2102	55.5	27	19	36.5	28.5	24	10	14	24
2103	52	25	20	22	28	27	6	17	13
2104	85.5	31	20	27	36	45	9	19	22
2105	70	27	18	23	25	24.5	8	15	16
2106	42.5	28	22	48	24	13	6	13	19
2107	81	35	21	37	37	35	13	19	26
2108	52	25	16	35	26	18	6.5	14	28.5
2109	65.5	25	17	27	25	21	8.5	14.5	21
2110	44	7	7	19	8	20	6	8.5	12
2111	43.5	39	18	27	29	11	5	15	23
2112	32	12	9	47	13	13	6	9	33
2113	28	15	14	27.5	10	10	8	6	22

2114	44	12	14	29	10	21	9	9	46
2115	38	10	15	26	20	16	6	8	16.5
2116	42	17	10	34	12	21	10	9	15.5
2117	55.5	30	23	28	31	21	12	18	28
2118	69.5	38	15.5	29	41	17	33	25	23
2119	49	22	16	23	25	27	9	17	21
2120	60	30	21	17	38	24.5	8	18	16
2121	73	26	22	19	30	34	10	22	22
2122	40	29	19	18	30	14	5	22	23.5
2123	84.5	25	18	34	27	34	7	17	42
2124	79.5	30	19	35.5	29	28	9	21	19
2125	45.5	26.5	20	29	32	12	9	15	16
2126	50	31.5	20	20.5	34	21	7	19	17
2127	43	28	18.5	21	59.5	15	32	16	14
2128	96	25	17	21	29	43	6.5	18	23
2129	64	24	18	24	31	27	9.5	18	37
2130	38	27	15	37.5	25.5	11	11	13	29
2131	54	29	25	19	31.5	14.5	8.5	16	22
2132	71	29	21	33	39.5	33.5	11	21	27
2133	41	24	15	14	28	8	4	14	16
2134	69.5	29	22.5	43.5	29	27	12	18	24
2135	122	26	22	23	36	21	15	17.5	21
2136	64.5	26	17	36	28.5	34.5	7	19	23
2137	81	28	21	33	37	39	8	21	23
2138	59	25	15	22	22	23	6	14	10
2139	67	32	22	26	35	31	10	23	19
2140	42	27	16.5	29	105	14	31	15	70
2141	54	23	22	36	29	21	8	15	53
2142	72.5	33.5	21	39.5	35	38	9	20	18
2143	65	12	8	19.5	11	29	5	9	25
2144	64	15	25	30	12	31	7	19	26
2145	84	13	8	23.5	15	27	9	11	18
2146	47	17	9	29	12	19	9	9	32
2147	59.5	16	14	30	17	32	10	15	15
2148	49	12	9	28.5	11	19	7	9	23
2149	62	10	8	28	10	36	7	11	19
2150	66	31.5	20.5	21	36	35	7	19	22
2151	56	28	18	31	29.5	22	12	18	22
2152	48.5	27	15.5	22	34	20	9	15	16
2153	71.5	23	16	24	23	39	6	15	82
2154	91	20.5	15	27	25	55	7	18	14
2155	74.5	21	15	35	23	51	6	15	13
2156	74	20	15	22	21	41.5	7	16	28
2157	91	20.5	15	27	25	55	7	18	14
2158	74.5	21	15	35	23	51	6	15	13
2159	74	20	15	22	21	41.5	7	16	28
2160	62	32	19	24	29.5	27.5	7	18	21

2161	62	32	19	24	29.5	27.5	7	18	21
2162	43	23	17	27	24	13	7	14	17.5
2163	77.5	33	21	41	40	41	9	21	21
2164	41	31	22	50	34	21	36	109	61
2165	61.5	31	19	29	39	19	15	19	21
2166	79	33	22	30	39	37	9.5	24	34
2167	72	36	25	40	52	29	12	130	22
2168	62	20	17	21	27	25	9	15	18
2169	45	16	8	38	17	24	9.5	8	22
2170	51	9.5	9	28	10	26	9	8	16.5
2171	73	28	23	24	31	37	9	18	28
2172	63	33	31.5	40	65	19	12	16	43
2173	49	23	16	26	27	21	6	13	29
2174	52	25	15	27	35	26	7	13	20
2175	67	25	23	46	29	34	6	16	15
2176	60	43	24.5	41	48.5	17	13	20	22
2177	79	26	20	36	31	53	10	20	30
2178	53	25	23	33	33	28	8	16	24
2179	62	34.5	20	42	34	40	15	16	26
2180	53	26	21	38	34	25	10	16	18
2181	70	28	22	34.5	33	33.5	12	21	24
2182	46.5	25	18	22	26	15	5.5	14	34.5
2183	38	9	9	25	12	22	9	11	35
2184	73	39	25	39	43	23	11	23	30
2185	74.5	25.5	17	27.5	29	32	8	18	31
2186	48	11	7	26	9	19	6	8	29
2187	40	13.5	12	40	11	21	51	8	22
2188	98	34.5	20	42	36	55.5	8	36	26
2189	61	26.5	20	23	34	25	42	17	25
2190	61	28.5	21	28	30	50	9	18	16
2191	69	21	17	22	25	32	7	17	18
2192	67	32.5	28	17	45	36	8	18.5	17
2193	81	36	25	27.5	38	31	8	22	35
2194	55	25	18	13	28	20	6	16	26
2195	1010.5	608	4927	301	1388	588.5	1392	1717.5	2082.5
2196	1010.5	608	4927	301	1388	588.5	1392	1717.5	2082.5
2197	73	32	25	77	46	31	15	17	27
2198	56	27	20	31	29	26.5	9	17	21
2199	62	32.5	25.5	29	35	29.5	9	20	20
2200	66	30	22	32	29	51	14	17	26
2201	37	26	24	25	29	10	6	14	21
2202	87	36.5	25	26	41	36.5	11	22	17
2203	68	32	23	22	31	28	9	18	24
2204	61.5	27	19	35.5	31	23	12	17	21
2205	61.5	27	19	35.5	31	23	12	17	21
2206	106.5	30	19	35	33.5	56.5	11.5	19	104
2207	81	31	21	40	32.5	49	9.5	20	22

2208	48	24.5	17	19	26	12	10	16	13
2209	51	32	20.5	24	37.5	20	8	17	37.5
2210	50	29	20	26	32	17	8	16.5	27
2211	82	31	20	45	51	38	14	22	34
2212	64	29	19	28	32	27	8	18	21
2213	55.5	27.5	20	29	35.5	24	7	17	21
2214	55	24	18	23	28.5	20	7	15	20
2215	78.5	24	17	29.5	27	38	21	17	25
2216	67	26	19	34	35	42	8	20	21
2217	39	35	22	33	40.5	14	8	17	19
2218	71	31	22	38	37	38	11.5	22	47
2219	57	27.5	22	30	41	22.5	9	20	19
2220	88	34	25.5	55	46	35	12	25	38
2221	44	10	8	23	8	11	7	7.5	26
2222	27	10	8	18	164	12	10	6	16.5
2223	41.5	15	9	31	10.5	20	8	8	38
2224	58.5	9	8	28	11	29	10	14	15
2225	64	29	22	20	34	30	8	18	20
2226	58	25	18	24	27	22	8	15	15
2227	72	21	16	21	29	33	8	18	21
2228	57	22	20	23	27	30	82	16	33
2229	57	22	20	23	27	30	82	16	33
2230	39	25	16	23	26	14	6	15	17
2231	39	25	16	23	26	14	6	15	17
2232	47.5	30	30	23	52	13	5.5	15	22
2233	79	28	17	31	27	34	9	17	16
2234	61	36	35.5	190	33	22	13	18	19
2235	88.5	37	23	35	44.5	35	11	23	32
2236	112.5	39	24.5	45.5	50	66	14	24	26
2237	54	24	19	26	25	25	9	15	22
2238	94	33	25	21	38	41	8	21	40.5
2239	53	26	20	17	32.5	15	6	20	18
2240	53	26	20	17	32.5	15	6	20	18
2241	79.5	24	17	25	57	37	7	17	19
2242	72	30	20	40	32	24.5	9	17	31
2243	77	32	20	37.5	38	44.5	11	23	20
2244	95	28	17	33	26	41	6	15.5	16
2245	76	32	21.5	36	38	43	9	21	24
2246	57	40	34	49	79	29	100	21	66
2247	46	13	10	13	16	15.5	5	10	13
2248	84	32	23	34	30	33.5	9	22	23
2249	65	32	24	34	32	30	10	18	22
2250	54.5	30	17	19	33	19	11	15	18
2251	81	26	16	39.5	28	47	11	19	36.5
2252	39	45	24.5	27	35	18	10.5	34	19.5
2253	42	56	53	84	41	35	61	54	57
2254	61	28	20	33	29	26	10	19	21

2255	77	26	17	29	33	31.5	9	20	29
2256	80	33	25	44	41	37	9.5	23	44
2257	42.5	11	10	22	49	16	7	7	30
2258	71	30	24	42	32	30	11	24	52
2259	53.5	13	8	26	13	24.5	7	11	32
2260	39	9	7	23	12	13	4	5	61
2261	57	12	8	30	29	24	8	10	15
2262	75	31	20	27	36	29.5	7	20	54
2263	71	32.5	21	25.5	35	34.5	7	19	23
2264	74.5	34.5	23	24	36	25.5	10	22	29
2265	39	27	19.5	32	24.5	12	8	13.5	27
2266	39	27	19.5	32	24.5	12	8	13.5	27
2267	57	33	22	25	29	21	18	17	20
2268	56	31.5	22	31	32	22.5	11	17	26
2269	29	43	29	37	24	8	6	13	7
2270	43	22	18	29	25	17	7	16	16
2271	70	32	31	41	47	28	16	19	102
2272	85	33	24	24	38	26	11	20	19
2273	77.5	29	22	27.5	31.5	47	9	20	27
2274	96	24	18	42	28	47.5	8	17.5	20
2275	60	27	17	34.5	56	30	10	16	19
2276	82	27	20	43	29	44	11	21	21
2277	94	24	17	28	24	59	12	20	24
2278	60	24	18	30	25	32	9	17	23
2279	90.5	24	16	22	49	39.5	8	16	23
2280	83.5	27	21	28	37.5	35	9	21	41.5
2281	105.5	71	24	152	54	66	47	21.5	61
2282	58	23	19	21	27	29.5	8	16	18
2283	58	23	18	22	27	26	7	17	20.5
2284	91	39	23	41.5	43	34	12	21	32
2285	60	27	24	27	32.5	11	6	17.5	32
2286	34.5	13	11	32	18	10	8	7	42
2287	30	11	7	21	9	11	7	6	34
2288	66	32	22	33	37	42	10	21	24
2289	68	29	21	26	38.5	33	12	17	46.5
2290	47	24	18	15	22	28	8	14	12
2291	47	24	14	17	21	13	7	10	27
2292	47	24	14	17	21	13	7	10	27
2293	91	36	27	19.5	57	33	11	20	20
2294	76	37	27	25	38	33	8	24	19
2295	52	25.5	20	15	28	22	8	16	19
2296	48	25	15.5	16	25	15	5	15	16
2297	47.5	22	17	34.5	29.5	18	8	14	33
2298	75.5	32	25	38	258	43	12	20	17.5
2299	161	35	18	55	26	66	17	24	9
2300	136	30	24	26	36	30	9	17	34
2301	64.5	28	19	29	79	32	10	18	21

2302	79.5	30	19	29	37	39.5	9	21	43
2303	87.5	26	19	26	32	39.5	10	25	20
2304	33	20	22	12	81	11	12	10	15
2305	44	12	10	24	15	20	9	11	18
2306	64	25	17	22	30	30	7.5	18	22
2307	71	34	25	40.5	50.5	36	15	17	68
2308	77	46.5	29	38	45	34	19	21	46
2309	63	33	22	30	42	23	10	19	20
2310	91	25	18	34	34	35	9	16	31
2311	54	30	18	38	35.5	19	9	14	23
2312	74	33.5	24	32	37	27	12	21	24
2313	81.5	32	21	35	44	57	12	27	29
2314	124.5	24	19	23	24.5	32.5	8	16	26
2315	73	24	21	41	32	49	11	18	20
2316	74	95	84	102	85	56	94	98	70
2317	64.5	37	26	79	36	28	10.5	21	20
2318	72	46	29	40	42	28	55	21	31
2319	109	30	20.5	31	32	44	13	21	23
2320	78.5	25	23	33	31.5	30	8	17	29
2321	63	27.5	23	38	28	30	8	18	23
2322	155	44	30	64	53	70	16	34	48
2323	76	17	8	25.5	11	36	7	11	29
2324	48	13	10	30	13	24	7	13	24.5
2325	64	23	16	22	24.5	37.5	7	15	24
2326	54	21	19	21	22	19.5	6	14	27
2327	47	28.5	22	19	34.5	14	7.5	16	25.5
2328	64	29	20	78.5	33	30	10	70	24
2329	64	29	20	78.5	33	30	10	70	24
2330	56	34	20	29	40	21	8	35	62
2331	92	37	23	35	39.5	39.5	14	38	28
2332	76	32	21	32	40	34	13	24	32
2333	65	25	17	24.5	32	26	7	17	19
2334	58	26	20	24	34	22	7	21	23
2335	43	31	22	24	37.5	12	11	17	13
2336	70.5	33.5	24	23	63	30	10	22	19.5
2337	58	23.5	15	29.5	26.5	31	7	14.5	35
2338	85	29	19	32	29	40	10	18	22
2339	104.5	35	25	34	39	54	13	25	23
2340	79.5	26	18	40	27	44	10	20	34
2341	82	35	24	30	42	33	11	22	29
2342	97.5	31.5	23	34	38	41.5	10	22	29
2343	76	23	20	28	29	30	10	19	38
2344	61	27.5	21	34	29	29	14	18	21
2345	58	10	8	24	9	11	5	9	44
2346	27.5	9	6	20	11	10	5	17	33
2347	78.5	28	18	29.5	29	43.5	9	19	37
2348	51.5	25	18	31.5	28	23	8.5	13.5	30

2349	69	25	17.5	30	27	30.5	7	19	40
2350	48	19.5	15.5	19	21	14	5	12	31
2351	48	19.5	15.5	19	21	14	5	12	31
2352	86	36	30	22	48	37	10	22	29
2353	45	27	16	24	31	18	6	15	33
2354	45	27	16	24	31	18	6	15	33
2355	49	23.5	15	21	25.5	20	9	16	15
2356	49	23.5	15	21	25.5	20	9	16	15
2357	59	24	17	14	24.5	15	5	13	16
2358	64	37	26	34	35	27	8	18	23
2359	54.5	26	18	27.5	27	16	10	17	27
2360	82	29	23	28	98	37	7	21	18
2361	43.5	23	16	16	63	8	7	14	16
2362	53.5	20	14	22	27	27	6	13	21
2363	50	41	25	41	36.5	16	25	17	14
2364	73	22	16	23	28	41	8	17	28
2365	73	22	16	23	28	41	8	17	28
2366	73	30	22	36	33	27.5	13	19	36.5
2367	93.5	30	21	34	32.5	43	13	19	20
2368	58	32	18	45	38	19.5	16	16	35
2369	64.5	32	19	27.5	35	19	7	16	22
2370	89	31	20	37	37	39	9.5	26	26
2371	103	34	22	37	42.5	56	10	24	47
2372	72.5	31	18.5	47	32	54	17	19	24
2373	75.5	22	16	27	27	42	7	17	13
2374	53.5	27.5	17	27	30	21.5	8	14	31
2375	89	29	24	32	31	39.5	15	25	46
2376	105	27	20.5	41	36	47.5	10.5	25	51
2377	76	29.5	21	28	34	37	10	26	26.5
2378	94	45	27	43	48.5	51	16	25	29
2379	65	38.5	23	30	37	27	10	23	17
2380	79	39	27	47	43	31	14	26	29
2381	65	28	17	25	28	15	11	16	71
2382	77.5	18.5	12	37.5	15	37	9	14	21
2383	73	15	12	40	17	34	14	14	24
2384	87	21.5	17	26.5	27	50	8	22	26
2385	50	28	20	33	28	15	9	16.5	11.5
2386	50	28	20	33	28	15	9	16.5	11.5
2387	48	23	17	26	28	14	6	14	61
2388	69.5	28	22	20.5	30.5	19	8	17	25
2389	47	27	16	31	28	13	8	13	42
2390	95.5	31	22	34	39	49.5	10	24	31
2391	63	26	20	28	35	24	7	15	20
2392	92.5	29.5	27	37	38	44	10	32.5	21
2393	87.5	34	23	27	34	39	11	20	23
2394	82	34	24	21	40	37.5	63	21	43
2395	49	31.5	21	22	34	12.5	8	17	31

2396	92.5	33.5	24	39	46	41	12	23	31
2397	71	26	20	38	48	43	9	19	31.5
2398	73	35	23	65	38.5	52	19	21	52
2399	93	25	17	35	26.5	53	15	18	36
2400	86.5	26	18	33	30	41	10	19	33
2401	89	31	24	37.5	34	41.5	10	22	24
2402	76	34	27	38	39	32	41	24	47.5
2403	79	35	22	34.5	36	37.5	9.5	22	27
2404	120	49	31	53	48.5	72	15	32	37
2405	83	32	22.5	34	38	56	10	20	27
2406	118	29	33	33.5	41	72	19.5	28	26
2407	94	28	19	32	32	60	12	19	17
2408	71	40	20	53	50	42	21	18	40.5
2409	68.5	32	20	25.5	37	25	8	20	26
2410	83	28	20	35	40	42	10	20	25.5
2411	96	39	24.5	73	40	39.5	51	19	27
2412	106.5	27	25	32	39	19	13	17	31
2413	101	31	29	34.5	28	54	9	19	24
2414	89.5	38	25	34	48	33	10	24	30
2415	90	32	23.5	44	32	44.5	11	22	51
2416	82	30	21	38.5	31	39	12	25.5	24.5
2417	92	21.5	14	55	18	52	11	20	27
2418	72	13.5	11	32.5	14	34	8	12	16
2419	65.5	27	20	31	48	39	9	16	25
2420	76.5	23	20.5	27	23	42	8	16	22
2421	76.5	23	20.5	27	23	42	8	16	22
2422	48	26	20	14	30	13	7	15	15
2423	62.5	25	17	30	85	38	7	16	29
2424	62.5	25	17	30	85	38	7	16	29
2425	87.5	36	22	34	33.5	32.5	8	21	28
2426	102.5	24.5	16	28	29	51	6	17	31
2427	57.5	25	17	25	28	20	7	14	55
2428	99	29	17	46	32	50	44	20	39
2429	76	35	21	34	41	36	14	23	26
2430	78	33	26	36	45.5	37.5	11	23	32
2431	68.5	37	25	31	43.5	19	11	18	26
2432	53	32	20	24	30	16	32	15	27
2433	79	26	18	30	30	39	20	19	51
2434	77	35	20	32	55.5	38	11	20	32
2435	41	19	9	20.5	28	19	9	17	23
2436	52.5	27	17	18	29	15	7	15	35
2437	77	32	23	33	36	39	17	24	43
2438	63	39	26	46	30	25	51.5	14	29.5
2439	69	30.5	21	32	39.5	29	13	18	29
2440	72.5	26	24	30	31	37	9	18	33
2441	109	36	34	53.5	34	53	14	26	29
2442	85	15	12	36	22	50	10.5	19	32

2443	60	15	11	33	14.5	29	10	12	19
2444	41	28	20	29.5	30	14.5	8	15	30
2445	60	24	19	27	25	25	15	16	33
2446	59	25	16.5	20	28	18	6	14	21
2447	84.5	26	21	40	31.5	44	47	19.5	64
2448	99	29	19	32	39	49.5	10	19.5	15
2449	71.5	35	24	39	40	24.5	11	22	26
2450	67	24	20	30.5	24	19	7	15	23.5
2451	67	24	20	30.5	24	19	7	15	23.5
2452	60	29	19	24.5	31	21	9	17	26
2453	101	26	17	25	27	41	7	17	39.5
2454	69	44	28	42	42	24	12	19	44
2455	75	39	24	31	38	48.5	13	22	30
2456	65.5	35	23	32.5	39	23	9	16	51.5
2457	63	33	25	28	51	16	11	17	27
2458	97.5	35	24	51	41.5	47	12.5	24	25
2459	87	32	20	40	37	41	32	28	35.5
2460	83.5	30	23	31	39	44	11	21	24
2461	43	67	66	82	57	48	58	66	62
2462	64	22.5	15.5	25	34	39.5	10	15	42
2463	57	30	24	22	37	14	12	20	21
2464	69	22	17	27	30	28	10.5	16	19
2465	54	30.5	17	87	33	13	13	15	19
2466	79	30	21	27	33	25	10	18	18
2467	63	27	18	39	30	29	9	20	41
2468	67	27	20	55	29	30	10	16	32
2469	97	44	29	57	52	49	14	32	28.5
2470	48.5	28	18	23	28.5	11	31	14	26
2471	49	57	31	55	36	24	13.5	15	29
2472	69	79	74	79	72	47	53	65	73
2473	70	23	17	21	25	32	10	16	16
2474	95	47	36	54	53	47	19	30	42
2475	82	42	27.5	40.5	44	39	67	21	31
2476	126	27	20	32	33	57.5	9	21.5	21
2477	54	28	16	21	280	17	6	14	20.5
2478	73	12	9.5	25	16	40	7.5	13	23
2479	81	13	10	31	19	42.5	11	13	51
2480	91	33	22	35	43	43	10	19	51
2481	71	27.5	20	24	32	31	11	19	28
2482	84	41	30	30	43.5	44	19	29	55
2483	96	34	22	25	39	56.5	9	25	19
2484	51.5	30	21	19	32	13	6	17	26
2485	50	21	17	31	27	16	11	14	20
2486	58	29	15	35	25	16	7	16	27
2487	57	28	20	25	28	12	9	13	15
2488	86.5	30	19	28	36	43	9	20	26
2489	66.5	12	11.5	25.5	13	35.5	10	10	16

2490	51	27	19	26	31.5	24	9	17	15
2491	74.5	25	20	32.5	34.5	32.5	9	19	26
2492	69	30	23	24	40	17	8	16	17
2493	36	23	18	25	28	11	9	14	20
2494	57	27	15	27	29.5	22	8	18	20
2495	36	29	28	47	33	13.5	9	19	40.5
2496	75	37	20	29	35	32	10	19	24
2497	102	34	23.5	43.5	43	58	13	33	52
2498	88	36.5	27	37	43	70	17	24	33.5
2499	100	35	22	45	39	56	12	27	44.5
2500	98	29	22	34	35	51	24	20	32
2501	58.5	22	21	23	25	22	9	13	18
2502	92	29	28	52	39	48	13	21	40
2503	95.5	30	21	33	32	38	9	21	41
2504	75	34	20	30	28.5	32	10	20	19
2505	77	86	93	79	79	67	75	90	86
2506	101	32	23	47	34	52	12	26	29
2507	39.5	13	14	33.5	11	15	9	9	19
2508	51	12	9	24	10	26.5	8	8.5	24
2509	94	26	18	29.5	43	31	12	21	25
2510	40	27	17	25	24.5	16	6	13	14
2511	40	27	17	25	24.5	16	6	13	14
2512	70.5	40	26.5	42	42	17	12	20	27
2513	82	34.5	21	34	37	30	10	19	22
2514	50	27	42	202	27	28	9.5	15	51
2515	82.5	33.5	22.5	32	38	47.5	11	25	26
2516	126	31	25	36	34	63.5	13	25	28
2517	96	31	18	35	36	41	8	23	22
2518	60	28	19	22	32	16	6	18	48
2519	60	28	19	22	32	16	6	18	48
2520	76	31	27	38	38	29.5	10	20	44
2521	94	36	23	36	47	42	11	23	26
2522	94	33	23	36	37.5	41	11	22	27.5
2523	96.5	26	21	32	33	44	9	19	17
2524	109	37	19	48	38	55	11	21	36
2525	91	33	25	52	44	62.5	14	22.5	27
2526	118	31	18	32	32.5	64	16	21	30
2527	87	28	20	35	31	44.5	10	21	20.5
2528	59	29	24	20	39	11	10	17	14
2529	78.5	29.5	21	28	36	43	15	25	23
2530	52	27	18	25	36	20.5	8	16.5	21
2531	111.5	37	25	48	44	48	12	43.5	30
2532	42	13	9	17	69.5	17	7	8	17
2533	69.5	15.5	9	28	15	29	8	8	86
2534	44	36	28	49	38	16	16	19	66
2535	67	28	19	27	32	35	9	17	26
2536	77	20	18	37	23	33	7	44.5	19

2537	75.5	21	16	25	23	30	9	14	22
2538	75.5	21	16	25	23	30	9	14	22
2539	99.5	30	26	41	41	48.5	15	25	35
2540	109	26	18	34	29	52	11	23	22
2541	127	39.5	26	37	49	53.5	13	25	29
2542	88.5	37	25	37.5	77	47.5	12	22	27
2543	85	25	19	27	30	45	11	19	28
2544	32	18	14	27	21.5	11	6	11	10
2545	101	29	20	39	30.5	53	10	22	23
2546	70	62	75	86	61	66	86	72	78
2547	99	30	22	30	36	49	10	26	22
2548	89	25.5	21	34	30.5	37.5	11	20.5	30
2549	174	15	12	29	11	63	9	12	13.5
2550	64	85	74	106	73	62	79	83	76
2551	51.5	32	18	23	30.5	14	5	18	12
2552	73	28.5	22	19	32	29	8	19	18
2553	51.5	32	18	23	30.5	14	5	18	12
2554	73	28.5	22	19	32	29	8	19	18
2555	58	34	21	30.5	33.5	16.5	8	16	27
2556	40	27	9	27	26	7	6.5	15	29
2557	117	38	24	39	37.5	68.5	13	25	21.5
2558	85	30	20	35	41	47	9	22	51.5
2559	79	29	22	39	32	28	10	18	37
2560	92	26.5	19	30	30	53	10	21	41.5
2561	129	42.5	28	46	41	55	14.5	27.5	55
2562	72	33	23	52	32	35	11	19	24
2563	106.5	28	20	44	34	48	11	20	23
2564	106.5	28	20	44	34	48	11	20	23
2565	72	29	25	57	29.5	28	8	17	19
2566	110	30	23	45	32	51	9.5	20	31
2567	68.5	25	19	23	28	29.5	7	18	15
2568	86	29	22	30	33	36	10	21.5	32
2569	64.5	35	22	50	33	25	9	18	29
2570	70	39	29	21	41	22	9	22	31.5
2571	76	34	27	36	40	27	7	20.5	27
2572	123	29.5	28	34	46	50.5	10	20.5	44
2573	59	33.5	22	26	34	19	10	15	25
2574	53	30	18	18.5	28	13	5	14.5	36
2575	81	37	26	31	44	36	13	22	28
2576	121	35	25	34	48	52.5	15	29	39
2577	115	29.5	21	45	44	54	10	54	37.5
2578	53	31	22	27	36	18	7	17	16
2579	86.5	28	19	32	32	46.5	10	21	19
2580	76.5	28	20	41	32	38	12	17	32
2581	75	30	21	24	33	35	12	19	44.5
2582	66.5	28	22	31	32	35	12	19	48
2583	99	27	18	30	35	47	9	21	28

2584	86	30	24	39	37	47	12.5	27	59
2585	113	33	26	39	38.5	55	13	26	30.5
2586	102	29	21	32.5	32	45.5	9	22.5	22
2587	92	27	19	33	30	39.5	13	20	39
2588	16	7	5	8	6	13.5	4	6	6
2589	52	71	27	81	36	38.5	55	21	164
2590	106	28	21	37	31	53	9	23	19
2591	66	13	16	48	13	28	8	11	11
2592	116	39	30	25	45	50	11	23	22
2593	80	34.5	26	63.5	31.5	33	17.5	21	26
2594	99	35.5	30	39.5	41	45	11	22	38
2595	85	28	22	38	36	43.5	20	24	34
2596	102	71.5	44	83	99.5	67	89	37	52
2597	80	258.5	84.5	142	90	79	35	20	28
2598	38.5	24	18.5	14	27	8	8	14	26.5
2599	117.5	12	9	29	12	36.5	117	12	27
2600	82	30	21.5	44	34	31.5	17	19	27
2601	62	26	17	24	28	30	8	16	32
2602	61	26.5	15	17	27.5	14	14	16	43
2603	103.5	26	17	27	41	53.5	10	20	24
2604	99	31	23	42	34	29	10	18	35
2605	81	31	23	37	40	44.5	11	22	34
2606	131	30	22	35	53	69	13	21.5	18
2607	56	37	23	43	39	25	10	18	42
2608	60	28	20	28	56	21	7.5	14.5	13
2609	92.5	40.5	27.5	42	49	46.5	17	28.5	58
2610	74	15	11	32	18	38	11	47	19
2611	66.5	17	13	38	15	40	10	20	22
2612	38	11.5	7	24	12	20.5	7	8	17
2613	137	41	25.5	28	46	102	12	27	33
2614	93	31	24	44	36	38	13	21.5	39
2615	49	26	19	22	48	17	12	15.5	30
2616	92.5	37	23	42	41	49	14	24.5	28.5
2617	140	40.5	31	38	51	57	15	31	35.5
2618	71	29	21	32	35	28	11	21	23
2619	60	27	20	33	26	15.5	17	14	24
2620	55	27	19	25	28	26	9	18.5	17
2621	101	25	21	29	31	57.5	9	23	19
2622	80.5	24	18	26	30	43	9	21	29
2623	39.5	18	18	21	17	22	13	8	22
2624	85.5	30	19	27	33	26	7	20	21
2625	85	33	20	49	34	47	13	20	24
2626	33	26	17	30	25	8	6	11	23
2627	102	35	25	49	37	39	10	27	28.5
2628	79	33.5	27	54.5	40	35	18	26	61
2629	67	28	19	25	32	25	7	17.5	20
2630	54	85	67	101	64	52	62	76	60

2631	61	31	19	38	38	11.5	9	17	25
2632	100.5	26	20	32	32	51	9	23	29
2633	83.5	35	24	41	40	43	14	26	42.5
2634	142.5	31	23	38	38	71	16	27.5	33
2635	131.5	33	38	35.5	44	49	16	24	27
2636	104	34	42.5	66	33	47	11	22.5	32
2637	82.5	17	17	34	18	35.5	12	18	33.5
2638	68	15	19	35	18	35	12	20	37
2639	31.5	10	8	30	10	10	8	8	21
2640	112	45.5	34	41	57	59	20	31	48
2641	83.5	30	24	23	37	41	11	26	29
2642	51.5	23	17	18	22	11	7	14	8
2643	75	31	22	30	32	25	10	19	33
2644	97	32	19	31	33	40	10	24	26
2645	114	30	23	43	33	54	12	23	23
2646	286	32	22	35	37.5	104	10	24.5	43
2647	82	25	18	27	30	35	8	17	28
2648	90.5	35.5	23	41	38	37	10	28	21
2649	101	47	27	60.5	43	73	23	21	32
2650	100	36	22.5	38	39	46	14	23	25
2651	77.5	24	17	25	40	44	10	16	43
2652	113	33	27	35	35.5	60	16	28	33
2653	51	9	9	24	11	25	8	9	26.5
2654	84	28	16	40	25	31	7	16	38
2655	87	39	26	29	42	20	10	20	37
2656	64	28	16	11.5	28	8	5	16	29
2657	64	28	16	11.5	28	8	5	16	29
2658	53	23	18	23	26	17	8	14	18
2659	53	23	18	23	26	17	8	14	18
2660	125	33	26	35	38	-1	-1	26	-1
2661	90	28.5	23	34	30	46	15	23	20
2662	46	30	21	29	32.5	13	9	17	13
2663	70	32	26	36	36	28.5	24	17	44
2664	65	28	17	27	26.5	20	8	13	28
2665	99.5	29	22	34.5	28.5	44	18	17	20
2666	107	36	23	36	40	54.5	11	24	28
2667	106	14	11	24	17	51.5	10	17	18
2668	65	31	23	30	42	23	10	18	29
2669	95	32	20	36	31	58	15	22	46
2670	67	23	18	26	29	14.5	10	13	40.5
2671	83	28	19	26	26	38	9	17	33
2672	80.5	28	18	38	30	31	6	16	23
2673	80.5	28	18	38	30	31	6	16	23
2674	51	22	17	93	42	18	12	14	23.5
2675	141	38	32	46	51	65.5	18	32	38
2676	97	30	22.5	44	35	53	14	26	27
2677	31	14	9	30	12	13	7	9	83

2678	44	23	16	29	24	10	8	12	28
2679	137	29	20.5	34	33	62	8	21	23
2680	86	35	25	27	39	47.5	8	22	33.5
2681	47	168.5	26	182	33	36.5	27.5	18	32
2682	86	27	19	35	36	43.5	10	20	21.5
2683	111	24	21	36	33	63	12	25	27
2684	97	27	22	35	26	50.5	11	19	90
2685	74	23	18	30	26	29	6	17	21
2686	130	19	13	36	18.5	53	13	17	22
2687	99	18	15.5	36	19	44.5	12	19	47
2688	105	31	20	35	32	69.5	11	29	57
2689	72	31	23	18	34	25	9	18	17
2690	113	37	26	33	48	52	13.5	29.5	44
2691	137.5	35	25	35	43.5	60.5	12	26	24
2692	102	28	27	36	38	53.5	12	21	37
2693	79	32	18.5	31	30.5	29	8	20	27.5
2694	69	33	24	25	38	21	13	18	77
2695	56	35	21	35	33.5	25	9	16	29
2696	171.5	39	26	45	48	80.5	13	35	30
2697	67	28	19	23	36	34	7	17	29
2698	59	22	18	21	31	18.5	8	16	22
2699	119	30	21	41	34	56	12	26	30
2700	79.5	32	17	24	28	34	8	19	29
2701	83.5	23	20	24	28	35	8	19	22
2702	105	28	20	31	32	45	11	21	47
2703	40	26	23	16	29	12	8	16	9
2704	42	9	7	22	12.5	13	5	6	21
2705	64.5	12	9	24	16	38	7	11	22.5
2706	123	31	23	25	37	60.5	10	28	44.5
2707	60	28	20	24	29	17	16	14	54
2708	114	26	22	32	30	46	10	23	25
2709	137	31	21	32	43	61.5	9	24	22
2710	74.5	22	18	21	24	33	9	16	37
2711	71.5	34	19.5	30	31	33	9	17	28
2712	152	30	23	31	37.5	80	9.5	21	36
2713	85	32	23	47	31	46	12	21	60
2714	65.5	33	21	34	36	21	10	17	29
2715	73	40	25	60	31	23.5	14	16	133
2716	73	31	34	37	32.5	21	10	16	16
2717	126	40	31	41	49	56.5	17	35	40
2718	117	46.5	32	46	53.5	60.5	16	30	41
2719	59	59	65	78	68	61	65	75	60
2720	106	29	24	37	36	46	12	21	20
2721	61	15	14	25.5	11	17	10	9	23
2722	56	13	8	26.5	9	22.5	8	10	23
2723	81	33	24	20	42	28	7	20	18
2724	150	37	27	29.5	43	88	10.5	26	24.5

2725	117	31.5	22.5	18	32	45	9	21	19
2726	82	32	19.5	26	33	26	8	20	21
2727	82	32	19.5	26	33	26	8	20	21
2728	54.5	23	16	20	24	19	5	14	31
2729	54.5	23	16	20	24	19	5	14	31
2730	120.5	30	20	26	28	47	7	20	26
2731	59	28	21	23	30	18.5	10	18	23
2732	111.5	34	27	49.5	35	59	20	28	32
2733	48.5	10	8	20	9	9	6	6	22
2734	81.5	22	15	26	31	34.5	11	17	36
2735	45	10	15	15	18	32	8	20	16
2736	103	23	21	32	30	55	10.5	19	19
2737	102.5	34	34	33.5	42	41.5	16	28	28
2738	93	26	22	27	32	35.5	12	22	37
2739	89	28	22	32	35	29	10	20	22.5
2740	115.5	33	24	52	38	53	16	29	28
2741	96	40	29	48	47	44.5	17	29.5	38
2742	124.5	33	24.5	46	40	67.5	14	34	28
2743	120	33	23	37	37.5	70	11	25	19
2744	65.5	14	15	37	11	24	10	10	18
2745	49	10	7.5	22	9	17	5	7	16
2746	79	31	21	41	31	37	9	18	39
2747	70	32	21	23.5	34	27	8	18	23
2748	161	33	25	54	41	80	14	27.5	70
2749	51	26	22	31	51.5	12	37	14	61.5
2750	78	34	21	27	44	28	8	21	31
2751	102.5	36.5	35	43.5	43.5	57	17	24	70
2752	79	37	24	33	39.5	37	13	22	23
2753	92	68	25.5	125	35	33	15	21	31
2754	61.5	36	19	50	39	26	8	18	40
2755	69	36	23	31	40.5	20.5	9	21	39
2756	97	42	59	63	38	52	19.5	27	35
2757	147	60	27	51	60.5	103	26	38.5	48
2758	122.5	11	18	25	10	57	9	12	19
2759	100	37.5	25.5	37	44.5	44	13	24	26.5
2760	138.5	44.5	32	53	50	60	17	34	52.5
2761	121	29	22.5	34	103	63	12	26	33.5
2762	85	21	19	36.5	25	45	20	26	29
2763	46	25	237	78	15	18	12	7	31.5
2764	66	28	21	29.5	28	29.5	7	30	21
2765	63	24.5	19	23	29	16.5	11	16	19
2766	114	36.5	25	36.5	48	41.5	15	25	51
2767	92	41	28	47	43	39.5	14	23	36
2768	92.5	31	26	31	34.5	36	11	22	37
2769	109	33	23	32	36	49	10	24	32
2770	93	23	18	32	31	41	11	19	27
2771	74	55	70	58	51	63	64	67	71

2772	113	37	23	40	46	53	11	28	78
2773	102.5	31	24	37.5	36	48.5	12	24	25
2774	79.5	32	25.5	29.5	36	24	11	19	24
2775	79.5	32	25.5	29.5	36	24	11	19	24
2776	83	35.5	23	33	32	27	10	20	31
2777	70	22	16	26	24	22	7	16	21
2778	35	26	16	37	26	9	8	12	22
2779	117	38	29	44	46	55.5	17	31	69
2780	151	31	26	41	37.5	62	16.5	28	41
2781	67	28	18.5	44	30	40	14	22	35
2782	84.5	32	19	23.5	36	22	8	18	56.5
2783	192	28	18	41	31.5	84	7.5	18	20
2784	119	42	28	43	53	52	13	29	33
2785	77	101	96	108	81	61	78	115	91
2786	60	26	17	20	22.5	12	6	13	26
2787	69	10	11	31	9.5	17	10	7	23
2788	92.5	30	21	27	33.5	28.5	11	18	34
2789	174	43.5	23	77.5	38	36	13	23	53
2790	67	31.5	20	27	39	22	10	29	23
2791	93	30.5	17	40	29	27	7	16	37
2792	126	48	34.5	51	66	64.5	20	40	44
2793	84	31	21	43	35	44.5	9	18	36
2794	49	23	17	24	24	8	5	13	16
2795	100	36	25	44	51	42	12	25	48
2796	123	38.5	26.5	42	40	54.5	21	24	29
2797	103	44	27	39	48	41	12	23	40
2798	136	39	24	41	64	72	12	29.5	31
2799	131	40	28	41	47	58.5	16	30	37.5
2800	88	23	15	21	27	41	8	17	34
2801	95	22	17	24	25	45.5	6	17	28
2802	95	22	17	24	25	45.5	6	17	28
2803	101	30	19	46	33	42	12	21	46
2804	107.5	42	28	34	42	49	12	24	38
2805	111.5	29	22	39	31	60	12	22	26
2806	90	37.5	22.5	36	36	30	11	20	38
2807	99	34	21	33	30	38	10	21	46
2808	81.5	29.5	20.5	29	32	32.5	8	17	32
2809	72	67	80	103	70	64	84	71	70
2810	74	34	23.5	35	46.5	33	14	24.5	44
2811	90	33	27	30.5	45	41.5	10	25	33
2812	112	30	22	37	49	53	12	25	33
2813	115	38	27	44	46	46.5	14	28.5	37
2814	70	9	10.5	25	9	25.5	9	11	25
2815	59	67	87	109	81	66	98	81	90
2816	77	28	22	26	30	16	10	14	32
2817	83	35	24	27	37	21	8	20	49
2818	153	40	28	49	46	66.5	15	32.5	35

2819	79	35	26	39	38	48.5	10	22.5	30
2820	123.5	35	23	37	39	62	14	30	62.5
2821	78.5	30	23	27	32	29	10	16.5	25
2822	81	32	19	24	33	22	11	18	30.5
2823	103.5	32	23	25.5	44	28	12	21	25
2824	127	50	35	48	61	50	26	40	48
2825	136	60.5	37.5	129	453	45.5	83	33	116
2826	118	46	39	46	66	51.5	18	30	81.5
2827	134	45	30	41	48	56	14	27	32
2828	123	31	21	36	36	59	12	23	45
2829	161.5	42	30	32	69.5	79	17	26	80
2830	91	32	26	29.5	42.5	40	13	26	23
2831	56	30	19.5	23	26	13	9	14	113
2832	56	32	19	21	29	16.5	6	15	19
2833	120.5	35	26	50	42.5	61	14	31	26.5
2834	53	66	79	75	54	39	68	85	74
2835	50	21	14	16.5	26	11	9	11.5	13
2836	131	33	24	34	40.5	63.5	14	25	36.5
2837	126.5	45	30	46	52	45	14	29	55
2838	132	29	23	33	37.5	76	12	28	54
2839	113	15	14	30	14	46	9	15	26
2840	69	27	17	19	27.5	18	5	15	17
2841	70	24	16	30	28	17.5	7	13	85
2842	124	34.5	32	38.5	67.5	61	16	28	35
2843	76	27	21	31	28	24	10	16	38
2844	93	32	21	31	33.5	36	11	27	27
2845	55	26	15	18	101	10	6	13	22
2846	67	69	79	90	48	50	60	71	67
2847	182.5	26.5	19	33.5	33	59	16	23	44.5
2848	117.5	27	23	35	43	47	12	25	31.5
2849	135.5	43	32	59.5	49	54.5	14	28	32
2850	118	44	30	50	48.5	42	15.5	26	48
2851	70	28	19	25	32	17	7	15	60
2852	76	27	19	22	25	45.5	9	15	26
2853	102	126	123	167	124	91	143	114	109
2854	143	35	25	42	41	66	13	28	38
2855	140	32	24	39	40	83.5	14	34	37
2856	78.5	26	27	32	43	26	9	18	22.5
2857	112	30	19	32	32	55.5	9	21	51.5
2858	67	19	12	17	22	19	6	12	21.5
2859	195.5	61.5	430.5	139	65.5	388.5	37	21	53
2860	150	51.5	35	48.5	56	76	24	35	40.5
2861	166	36	25	49	45	73	17	27.5	45
2862	63	27	15.5	54	23	18	7	14	23
2863	88	37	20	24	37	25	6	22	34.5
2864	112	35	22	32	42	47.5	14	27	29
2865	75	37	24	36	39.5	23	10	20	45

2866	91	124	108	117	109	82	90	97	104
2867	120	32	24	22	57	16	14	21	45
2868	174.5	45	41	53	48	95	15.5	33	61
2869	67	26	19	23.5	25	30.5	9	14	87
2870	91.5	27	18	36	32	32	8	16.5	31
2871	216	36	25.5	33	38	77	47	21	31
2872	81	105	88	109	90	76	97	80	80
2873	108	37.5	22	32.5	55.5	44	10	27	42.5
2874	49	27	19	24	25.5	22.5	6	13.5	31
2875	114.5	14.5	15.5	39	17	56	11	18.5	19
2876	40.5	22	14.5	19	27	11	13	13	11
2877	111	33	24.5	38	41	52.5	14.5	26	51
2878	99	34	25	43	48	34	19	20.5	126.5
2879	106	33	21	35	35	45	10	21	32
2880	160.5	15	15	42.5	19	72	12	18	24
2881	101	42	28	38	49	52.5	14	31	55.5
2882	110.5	40	27.5	38.5	57	37	11	24	45
2883	88	132	108	114	95	73	85	123	100
2884	118	36	23	40.5	41	48	9	22	71
2885	122	32	26	41	48	60	14	24	24
2886	93	103	119	145	115	83	121	101	108
2887	82	29	22.5	32	31	33	9	20	28
2888	95	42	27	24	40	34	7	20	22.5
2889	137	46	34	81	39	65	24	23	52
2890	59	26	23	33	32	29	10	20	29.5
2891	78	33	28	40	41	22.5	11.5	20	26
2892	90.5	28	18	23.5	26	50	9	18	27
2893	117	40	23	73	49	62	23	24	41
2894	98	30.5	23	37	45	34	12	19	23
2895	117	35	24	38	36	56.5	12	24	74
2896	99	31	23	37.5	34	44	11.5	24	25
2897	80	34.5	27	33	35	13	10	17	22.5
2898	150.5	33	27	35.5	37.5	66	14	27	32
2899	119	34	18	24	25	41	7	16	44
2900	119	34	18	24	25	41	7	16	44
2901	104.5	24.5	19	15	62	13	7	14	36.5
2902	171	43	39	59	53	106	21	45	43
2903	107	35	25	45	35	52	13	25	28.5
2904	113	127	123	161	98	85	140	110	106
2905	90	85	94	103	75	83	85	114	88
2906	36	27	28	21	43	20	9	16	15
2907	107	36	27	44	41	49	13	29.5	32
2908	138	46	35	48	54	58.5	51	35	36
2909	160.5	46	33	53	53	76.5	19	38	44
2910	109	36	28	50	48	49	17	28	48
2911	147	43.5	35.5	52	51	76.5	17	34	41
2912	144	34	29	46	43.5	65	22	32	37

2913	87	25	20	21	28	20	7	14	25
2914	103.5	30	22	45	31	53.5	12	23	32
2915	143	41	34	59	53	70	32	35	65
2916	151	35.5	23	37	38	71.5	10	24	36
2917	70.5	29	13	23	28	12	7.5	15.5	20
2918	160	44	32.5	53.5	52.5	73	25	34	73
2919	208	39	25.5	38	47	101	12	26	32
2920	177	28	20	33	39.5	63	11	19	30
2921	138	31	26	42.5	43	75.5	15	30	30
2922	97	30	21	30	31.5	36	9	17	37
2923	68	22	21	25	24	22.5	12	13	43
2924	68	22	21	25	24	22.5	12	13	43
2925	75	23	18	20	90	19	8	13	24
2926	75	23	18	20	90	19	8	13	24
2927	99	30	23	28	44.5	22.5	10	20	46
2928	143	43	27.5	44	42	74	14.5	28	35
2929	57	15	12	28	11	17	12	9	16
2930	95	43	27	38	53	34	11	25	43
2931	157	42	26	54	45	83	16	32	36
2932	157	42	26	54	45	83	16	32	36
2933	108	38	27	50	39	53	16	25	28
2934	90	101	129	113	91	85	117	100	115
2935	130	41	27	43	46	56	16	29	55
2936	446.5	31	19	26	35	149	8	22	56
2937	138	47	36	50	63	66	15.5	33	51
2938	98	113	113	146	111	79	107	98	108
2939	123	19	20	48	26.5	78	16	34	49
2940	111	28	19	29	30	71.5	9	24.5	31
2941	85	30	20	32	34	28	10	16	34
2942	141	31	29	50	37	59	16.5	25	37
2943	40	24	17	19	23	14.5	10	13	11
2944	76	101	99	145	94	101	84	115	75
2945	71	102	105	117	107	77	85	105	100
2946	89.5	31	19	21	29	31	12	16	38
2947	172	39.5	26	49	42	72.5	17	34	36
2948	137	41	28	41	46	52	15	25	56
2949	76	10	10	33	10	29.5	6	9	14
2950	77	23	17.5	22	30	11	6	14	30
2951	59	28	20	24	24	16.5	7	15	24.5
2952	59	28	20	24	24	16.5	7	15	24.5
2953	151	35	29	37	45	59	13	29	38
2954	108.5	32	25	32	38	46	12	21	38.5
2955	162	37	29.5	51	46	96	14	31	37.5
2956	177	35	34	68	60	85	21	44	52
2957	78	36	19	28	33	18	6	17	53.5
2958	95	141	117	133	112	83	122	120	118
2959	148.5	54	44	70.5	63	78	27	54	60

2960	90	34	27	31.5	36	31	11	25	38
2961	90	34	27	31.5	36	31	11	25	38
2962	114.5	43	31	44	43.5	54	17	29	38
2963	125.5	44	30.5	52	63.5	52	20	32	46
2964	174.5	35	25	43	133.5	89	13	27	41
2965	65	86	79	113	77	69	81	88	94
2966	144	50	33	56.5	56	72	23	35	40
2967	164	46	30	48	60	76	18	33	48
2968	155	40	35	48	47	97.5	15	33	30
2969	108	42	28	33	42	26	13	22	50.5
2970	58	25	16	20	25	19	6	15	15
2971	155	40	23	109	33.5	53.5	14	24	23
2972	75	33	25	49	36.5	29	15	51.5	76
2973	80	95	90	88	81	78	86	96	75
2974	169	47	33.5	50	54	81	17	35	49.5
2975	157	36	9	47.5	26	97.5	12	15	42
2976	73	12	8	28	9	16	7	7	41
2977	95	27	19	31	28	30	8	20	71
2978	108	28	18	36	26.5	24	25	15	48
2979	182	36	21.5	39	40	54	10	22	38
2980	164	39	30	40	53	86	20	33	49
2981	66.5	34	17	29	27	18	7	15	39
2982	66.5	34	17	29	27	18	7	15	39
2983	94	35	21	26	30	26.5	9	17	43
2984	87	94	86	112	88	83	93	80	105
2985	104.5	29	27	38	35	40.5	11	26	28
2986	130	37	28	42	46	53	11	25	23
2987	163	19	15	38.5	29	75	15	19	44.5
2988	103.5	11	12	26.5	13	41	9	13	22.5
2989	52.5	8	5	21	5	7	5	5	21
2990	85.5	25	15	24.5	26	10	4	11	33
2991	106	28.5	23.5	26	38.5	30	17	18	41
2992	102	36	29	48	47.5	61	20.5	29.5	34
2993	80	103	96	118	96	87	108	118	94
2994	144	38	33.5	65	56	74	22.5	85	121
2995	147	49	37	62	57	89	30	39	51
2996	107	31	27	31	37	45	11	23	33
2997	116	32.5	43	61	35.5	48	27.5	24	29
2998	160.5	42	32	33.5	54	54	12	30	50
2999	163	44	30	71	41.5	90.5	20	35	98
3000	125.5	33	19	53	34	55	16	19	44
3001	95	128	116	141	114	86	120	121	88
3002	82	94	85	95	68	82	101	85	91
3003	88	31	31	30	30	32	11	18	39
3004	100	123	111	134	84	89	112	117	91
3005	81	36.5	22	29	41	29.5	9	20	52.5
3006	136	39	28	46	46	58	16	28	42

3007	141	38.5	34	41	46	70	13	36	39
3008	101	140	112	176	125	101	111	123	104
3009	97	42	28	41	39	23.5	12	30	76
3010	83	28.5	18	26	28	23	7	15	58
3011	59	14.5	23	28	16	19	9	8	62.5
3012	89	117	133	146	117	101	119	117	129
3013	145	28	21.5	31	34	59	14	26	32
3014	66	35	22	41	35	23	18	17.5	36
3015	183	51	40	56	63	82	19	43	53
3016	97	124	127	117	97	81	93	140	118
3017	168	55	39	64	62	77	27	42	68
3018	77	14	8	88	11	27	16	8	17
3019	142.5	35	33	56	82	88	20	37	41.5
3020	108	131	140	175	126	88	119	111	120
3021	145	32	26	43	37	68	15	29	36
3022	109	95	118	124	88	89	99	102	124
3023	164	56.5	39	74.5	65	71.5	22	43	62
3024	93	92	107	102	89	75	105	125	99
3025	107	153	143	176	124	109	120	119	117
3026	73.5	23	21	32	28	21	8	15	24
3027	105	121	117	138	113	102	110	122	93
3028	152	35	22	41	43	49	13	18	55
3029	85	31	18	30.5	29	22	7	16	41.5
3030	123	139	123	167	102	100	131	138	118
3031	97	122	130	112	111	93	107	143	112
3032	124	53	31	64.5	32	51	14.5	22	122
3033	88	35	23	26	40.5	35	10	17	47
3034	107	25	22	49	32	56	13	21	29
3035	84.5	27	17	34	24	33.5	9	15	27
3036	88	37	26	22	37	26	10	21	26.5
3037	78.5	24	18	14	31	9	5	15	32
3038	78.5	24	18	14	31	9	5	15	32
3039	92	24	21	13	30	12.5	6	15	30
3040	80	23	26	35	40.5	44	9	17	31
3041	96	103	114	127	86	91	106	86	93
3042	126	38	26	41	46	51	13	27	64
3043	145	35	27	48	49	63	16	28	48
3044	149.5	36	29	39	44	64	20	32.5	47
3045	191.5	51	36.5	58	50	85	16.5	36	62.5
3046	119	161	137	181	128	112	146	143	125
3047	89	25	18	24	32	25	10	15	28
3048	105	39	22	47	30	30.5	11	15	62
3049	103	29	23	30	29	33	11	17	141
3050	73	24	46	14	30	16	12	14	34
3051	177	53	31	55	57.5	86.5	16	36	53
3052	97.5	23	15.5	15	24	15	6	14	34
3053	73.5	29	27	31	35.5	35	15	25	31

3054	82	103	89	91	82	60	90	87	87
3055	96	108	101	130	95	97	103	119	103
3056	103	107	120	140	108	91	108	113	104
3057	135	35	23	35	47	57	13	27	25.5
3058	202	48	32	48	55	80	18	30	62
3059	109	141	127	124	111	86	113	121	130
3060	53	11	8	23.5	12	11	8	6	37
3061	241	32	25	24	34	91.5	10	19	28
3062	120	107	145	134	107	110	101	113	112
3063	124	100	127	117	113	118	131	121	118
3064	138	135	137	126	118	115	131	141	131
3065	105	122	118	125	109	104	100	123	132
3066	100	129	121	137	120	110	141	138	139
3067	114	122	140	125	107	105	109	100	153
3068	122	110	125	117	100	117	135	115	115
3069	119	123	118	120	137	104	110	116	136
3070	110	118	127	126	123	107	106	136	113
3071	111	133	136	155	126	112	106	131	131
3072	132	140	161	141	122	113	142	148	148
3073	115	115	119	121	105	119	153	129	138
3074	114	122	126	127	106	105	130	127	131
3075	117	102	128	106	128	103	102	114	122
3076	121	123	116	110	123	100	100	124	128
3077	118	138	132	106	121	119	113	139	101
3078	104	132	156	150	113	112	149	143	132
3079	125	114	129	153	107	100	127	135	126
3080	115	124	116	104	119	106	114	139	126
3081	109	130	130	136	102	121	100	126	125
3082	112	108	126	118	105	106	122	107	135
3083	112	127	136	137	122	100	131	134	117
3084	141	132	146	129	119	101	141	116	136
3085	116	129	134	144	118	107	128	114	126
3086	100	105	127	106	128	107	132	140	160
3087	116	117	124	120	114	107	125	124	133
3088	122	109	126	125	108	103	109	115	159
3089	103	115	119	137	127	100	130	110	119
3090	107	112	123	117	100	100	102	100	104
3091	125	131	127	129	128	146	115	140	126
3092	113	138	115	120	108	108	121	134	133
3093	112	128	116	122	116	100	139	128	139
3094	100	121	131	133	115	106	133	119	118
3095	109	116	121	132	111	104	126	105	119
3096	106	116	115	126	113	107	118	127	122
3097	107	115	125	143	115	105	139	131	130
3098	122	128	112	114	110	105	111	124	100
3099	118	116	115	118	100	105	105	111	112
3100	130	133	130	122	144	124	124	128	126

3101	47	12	7	29.5	12	10	6	28	23.5
3102	126	121	119	118	101	115	141	136	135
3103	113	133	138	107	133	107	104	127	113
3104	101	115	141	111	101	101	141	124	119
3105	100	113	118	111	108	105	139	121	118
3106	125	136	150	173	126	107	125	129	116
3107	127	126	112	127	102	102	107	121	117
3108	112	114	129	117	102	100	110	125	112
3109	122	119	143	121	126	111	122	132	120
3110	122	119	143	121	126	111	122	132	120
3111	106	136	128	125	117	109	115	142	110
3112	104	106	106	113	133	100	121	136	130
3113	132	138	140	128	123	100	145	141	130
3114	101	104	127	128	115	117	100	105	114
3115	105	110	105	134	121	105	126	125	121
3116	110	142	129	120	120	106	136	120	137
3117	118	129	107	101	114	102	118	131	137
3118	100	121	123	134	128	100	129	142	154
3119	100	145	131	148	103	111	118	136	131
3120	107	145	135	168	130	108	152	154	148
3121	109	135	136	150	120	104	142	150	130
3122	100	134	131	149	111	111	137	135	121
3123	135	126	119	151	111	100	117	126	128
3124	113	127	122	182	127	116	108	122	126
3125	127	140	154	170	135	98	145	151	139
3126	127	140	154	170	135	98	145	151	139
3127	129	131	126	153	102	100	107	131	124
3128	130	153	132	158	142	113	117	166	138
3129	539	33	21	40	60	266	61	29	51
3130	126	128	122	154	131	95	122	135	127
3131	123	146	124	160	126	100	144	131	151
3132	119	124	137	160	110	122	142	137	133
3133	190	52	39	55	980	89	17.5	35	108
3134	144	159	154	165	148	95	155	139	122
3135	135	150	138	161	158	100	133	135	131
3136	135	150	138	161	158	100	133	135	131
3137	128	148	139	178	152	100	112	138	130
3138	144	127	139	164	148	100	141	134	153
3139	94	129	145	162	134	106	129	126	120
3140	119	146	143	181	131	104	147	112	138
3141	282	68	45	72.5	74	115	30.5	55	66
3142	102	166	148	181	154	100	150	133	141
3143	102	166	148	181	154	100	150	133	141
3144	109	128	116	165	108	100	144	144	124
3145	127	144	154	172	139	123	147	148	124
3146	115	121	125	174	135	110	143	140	156
3147	121	159	124	182	111	102	133	132	140

3148	116	100	114	169	120	100	126	103	104
3149	122	177	165	188	132	97	127	150	131
3150	134	121	127	172	123	100	129	123	144
3151	434.5	62.5	49	94	81	210	34.5	72	76.5
3152	100	129	139	174	143	99	117	153	130
3153	138	140	152	187	150	110	152	147	147
3154	139	144	151	179	106	129	134	164	148
3155	110	138	130	187	134	125	152	135	123
3156	110	138	130	187	134	125	152	135	123
3157	396	68	53	78	81.5	151	34	62	68.5
3158	123	141	165	200	144	100	141	152	129
3159	792.5	36	109	31	34	273	9	30	32.5
3160	777	45.5	32	87	69	411	38	59	83.5
3161	469.5	86	59	203	81.5	227	58	37	123
3162	297	59	48	68	116	127.5	29	55	70.5
3163	559	36	21	62	52	355.5	27	45.5	65
3164	334	78.5	68	93	92	160	50	71	85
3165	362	40	27	61	46	147.5	17	36	69
3166	360.5	58	41.5	87	68	230	25.5	74	68
3167	535	68	57	104	92	263	38	84	90
3168	32	23	16	88	38	34	14	21	17
3169	415.5	451	87	3392	65	895	833	291	369
3170	36	22	15	45	27	34	10	13	16
3171	76	31	22	71	32	54.5	15	19	36
3172	28	16	8	36	13	21	6	17	15
3173	33	25	15	52	27.5	31	11	18	23
3174	43	30	16	50.5	19	36	14	30	34.5
3175	446	480	95	3525	93	906	788	294	401
3176	51	34	41	91	29	41	17	16	24
3177	55.5	45	16	47	61	49.5	13	35	57.5
3178	103	83	57	70	35	92	32	77	69
3179	44.5	31.5	16	44	22	42	9	23	28
3180	42	30	12.5	40	16	42	11	46	24
3181	40	26	10	41	17.5	34	10	33	11
3182	32	22	10	45	18	27	10	26	17
3183	32	19	9	39	17	25	8	21	5
3184	39	18	10	37	13	26	8	19	95
3185	23	12	15.5	44	27	24	12	24.5	96
3186	41	31	13	33	15	41.5	9	24	21
3187	31	20	10	40	13	25	8	20	11
3188	42	28	11	36	16	34	11	26	23
3189	54	41.5	17	53	25	45.5	15	50	51
3190	37.5	23	12	33	22	32	8.5	18	82
3191	32	21	16.5	40	18.5	20	8	37	20
3192	66	50	27	55	28	54	21	47	80
3193	33	21	14	26	15	26	9	24.5	28.5
3194	51	34	14	37	17	40	11	27	28

3195	110.5	89	46	80	43	90	37	83	83
3196	53	37.5	21	47	24	42	14	32	31
3197	37	27	10	40	14	34	8.5	18	20
3198	47	28	13	45	18	31	11	30	31
3199	69	52	24.5	54	23.5	59	20	50	58
3200	42	28	12	49	22	34	12	26	47
3201	42	28	16	46	18	31	12	22	24
3202	117	24	15	53	15	28.5	8	25	14.5
3203	37	19	12	32	15.5	20.5	6	21.5	13
3204	37	23	12.5	39	24	27	8	19.5	16
3205	51	37	17	45	25	41.5	15	33	47
3206	40	23	13	44.5	17	30	12	26	26
3207	68	45	23	55	27	47	16	41	44
3208	34.5	21	19	47.5	16	24	9	22	24
3209	41	24	11	34	20	28	14	22	38
3210	30	15	8	35.5	18	20	7	25	27
3211	39.5	29	14	40	25	34	12	27	44
3212	59	42	21	51	22.5	47	17	36	47
3213	41	29	12	36	24	32	12	26.5	17
3214	33	20	11	42	14	25	10	21	35
3215	44.5	28	14	56	19	36	12	29.5	41
3216	36	16	10	52	15.5	20	17	38	9
3217	34	20.5	9	25	13	24	7	16	16.5
3218	27	15	7	24	15	17	6	13	7
3219	34	20	9	51.5	16.5	26	31	33	20
3220	30	20	7	33	16	28	7	24	8
3221	27	21	7	21	13	25	6	15	4
3222	35	19	17	53.5	21	25	10	32	53.5
3223	29.5	14.5	13	46	18	21	9	35	30
3224	24	16	6	20	13	20	6	13	20
3225	33.5	14	9	18	15	19	8	14	70
3226	39	18	9	19.5	16	20	12	14	5
3227	30	15	9	25	13	20	9	17	20
3228	29	19	8.5	20	19	23	7	17	6
3229	42	12	6	16	16	16	8	13.5	9
3230	650	15	21	23	15	17	27	13	40
3231	30	12	6	21	13	15	6	10	3
3232	30	19	70	48	41	23	34.5	29	4
3233	21	14	15	33	19	16	12	17	7
3234	30	17	8	54	18.5	26	14	28	14
3235	32	17	8	51	14	21	36	30	10
3236	21	14	6.5	18	11.5	16	4	12	9
3237	40	18	8	37	15	19	8	18	44
3238	28	15	9	25	14	20	6	13	6
3239	25	13	6	24	12	15	7	13	12
3240	20	14	7	22	12	16	5	13	4
3241	36	16	17	40.5	19	19	30.5	20.5	21

3242	27	16.5	7	21	14	21	7	15	4
3243	37	19	13.5	55	52.5	23	16	21	12
3244	35	20	11	25.5	19	26	14	17	17
3245	27	12	8	24	15.5	16	8	36	5
3246	38	20	32	18	16	21	9	15	27
3247	25	15	7.5	19	17	17.5	7	13	6
3248	68	18	9	38	1004	27	7	53	25
3249	26	17	8	58.5	14	24	8	34	4
3250	25	16	8	24	15	17	19	22	3
3251	28	22	8	18	13	36	7	94	24
3252	27	14	7.5	26	12	18	8	15.5	2
3253	122	30	13	58.5	21	38.5	11	42	5
3254	21	11	6	20	17	12	8	11.5	4
3255	35	15	10	36.5	18	19	30	20	7
3256	52	19	16	26	20	22	21	13	13
3257	19	12	7	17	15	18	18	15	9
3258	23.5	12	10	26	13	10	17.5	6	8
3259	33	15	52	18	32	23.5	8	10.5	15
3260	47	19	71	23.5	27	28	11	13	16
3261	43	14	10	12	16	18	10	15	15
3262	30	14.5	9	20	29	17	7	21	16
3263	32	15	9	16	15	19.5	9	13	5
3264	115.5	12	10	25	20	14	7.5	14	246.5
3265	17	12	5	14	10	12.5	5	9	3
3266	15	15	5	14	12.5	17	5	10	3
3267	16	11.5	7	13	9	16	6	10	2
3268	19	12	7	21	10	13	5	11	4
3269	24.5	14	6	59	12	19	9	20	18
3270	40.5	22	11	54	18	29	14	64	13
3271	23	15	7	21	11	18.5	6	14	1
3272	18	12	5.5	11	12	16	6	9	2
3273	26	11	34.5	30	19	13	9	12	11
3274	80	15	21	50	54	20	23	25	12
3275	59	20	14	67	23	24.5	71	28	9
3276	19	12	10	15	23	13	6	11	8
3277	22	11	7	24	12	14	13	14	50
3278	61	14	8	38	19	17	7	28	20
3279	23	13	7	20	24	15	11	12	16
3280	31	17	9	36	19	23	9	17	38
3281	2	1	1	3	1	1	0	2	0
3282	38	16	10	38	13	18	9	38	16
3283	2	1	1	3	1	1	0	2	0
3284	27.5	10	8	12	11	13	10	10	11
3285	2	0	2	3	1	1	0	2	0
3286	11	8	6	7	6	10	4	8	1
3287	2	1	1	3	1	1	0	2	0
3288	23	15	8	18	13	15	9	14	8.5

3289	2	1	1	3	1	1	0	2	0
3290	52	40	13	38	18	47	11	31	51
3291	2	0	1	3	1	1	0	2	0
3292	22	12	8	21	11	15	9	12.5	16
3293	2	1	1	3	1	1	1	1	0
3294	33	22	10.5	26	12	25.5	11	23	18
3295	2	1	1	3	1	1	0	2	0
3296	28	15	12	27	21	19	8	17	11
3297	2	1	2	3	1	1	0	2	0
3298	78	55	21	43	22	60	18	51	71.5
3299	2	1	1	3	1	1	0	2	0
3300	38	26	10	25	15	31	11	24	34
3301	2	1	2	3	1	1	0	2	0
3302	32	21	13	29	13	25.5	11	23	23
3303	2	1	1	3	1	1	0	2	0
3304	44.5	31	15.5	40	13	30	17	29	25
3305	2	0	1	3	1	1	1	2	0
3306	31	24	10.5	22	11	20.5	13	23	25
3307	2	1	1	3	1	1	0.5	1	0
3308	44	37	15	32	14	44	10	35	43
3309	2	1	1	3	1	1	0	1	0
3310	28	17	10	28	11	19	8	20	11
3311	2	1	1	3	1	1	0	1	0
3312	22	9	7	20	8	11	6	13.5	5
3313	2	1	1	2	1	1	0	1	0
3314	32	21	14	27	13	25	9	32.5	20
3315	2	1	1	3	1	1	0	2	0
3316	37	29.5	12	30	13	29	11	31	33
3317	2	0	2	3	1	1	1	2	0
3318	28	18	9	26	12	19	9	20	12
3319	2	1	1	3	1	1	0	2	0
3320	24	9	7	20.5	8	11	7	16	7
3321	2	1	1	4	1	0	0	1	0
3322	2	0	2	3	1	1	0	2	0
3323	26	18	9	21	9	18	18	16	9
3324	2	1	1	2	1	1	0	2	0
3325	30	22	11	23	12	21.5	10	23	18
3326	2	1	1	3	1	1	0	2	0
3327	56.5	27	13	32	15	27	12	27	28
3328	2	0	1	3	1	0	1	1	0
3329	25	10.5	9	21.5	7	15	7	14	10
3330	2	1	1	3	1	1	0	2	0
3331	34	18	14.5	23	11	21	8	22	19
3332	2	0.5	2	3	1	1	0	2	0
3333	33	10	8	21	9	13	7	13	11
3334	2	0.5	1	3	1	1	1	1	0
3335	30	19	21.5	26	13	21	11	19.5	27

3336	2	0	1	2	1	0	0	2	0
3337	2	1	1	3	1	1	0	2	0
3338	25.5	13	9	26	13	15	9	21.5	22
3339	2	1	1	3	1	1	0	2	0
3340	31	20	10	25	16	23	9	18	16
3341	2	0	1	3	1	1	0	2	0
3342	44	28	15	34	15	30	11	27	24
3343	2	1	1	3	1	1	1	1	0
3344	39	29.5	12	27	11	31	10	28	31
3345	2	1	1	3	1	1	0	2	0
3346	26	19	8	24	10	22	7	12	30
3347	2	0	1	3	1	1	0	2	0
3348	43	20	14	24	12	21	10	23.5	20
3349	2	1	1.5	3	1	1	0	2	0
3350	45	31	17	31.5	15	39	15.5	75.5	42
3351	2	1	2	3	1	1	0	1	0
3352	104	8	7	17	11	12	12	12	9
3353	2.5	1	1	3	1	1	0	2	0
3354	2	1	1	3	1	1	0	2	0
3355	26	16	10	24	11	19.5	8	18	26
3356	2	1	1	3	1	1	0	2	0
3357	21	12	10	25	9	13	7	15	7
3358	2	1	1	3	1	1	1	2	0
3359	91	70	29	54	29	70	26	65	70
3360	2	1	1	3	1	1	0	2	0
3361	53	33	19	36	25	35	16	34	32
3362	2	0	2	3	1	1	0	2	0
3363	36	13	9	19.5	10	14	9	15	19
3364	2	0	1	3	1	1	0	1	0
3365	36	27	13	27	13	29.5	9.5	25	39
3366	2	1	1	3	1	0	0	2	0
3367	35	22	11	27.5	14	24	11	22	19
3368	2	0	1	3	1	0	0	2	0
3369	21	10	8	21	9	14	11	14	9
3370	2	1	2	3	1	1	0	2	0
3371	2	1	1	3	1	1	0	2	0
3372	2	0	1	2	1	1	0	2	0
3373	30	11	9	20	9	11	7	13	13
3374	1	1	1	3	1	1	1	2	0
3375	2	0	2	3	1	1	1	2	0
3376	2	1	2	3	1	1	0	2	0
3377	20	12	8	18.5	8	12	7	13	28
3378	2	1	1	3	1	1	0	2	1
3379	2	1	1	3	1	1	0	2	0
3380	83	53	16	43.5	23	45	20	35	43
3381	2	1	1	2	1	1	0	2	0
3382	20	13	9	24	10	18	11	13	9

3383	2	0.5	1	3	2	0	1	2	0
3384	14	10	5	8	5	12	4	7	1
3385	2	1	1	3	1	0	1	2	0
3386	13	9	6	6	6	13	3.5	7.5	2
3387	2	0.5	1	3	1	1	0	2	0
3388	13	10	5	7	6	13	5	8	1
3389	2	0	1	3	1	1	1	2	0
3390	14	10	6	7	6	11	5	8	2
3391	2	1	1	3	1	1	0	2	0
3392	16	12	6	7	6	14	4	8.5	1
3393	2	1	1.5	3	1	1	0	2	0
3394	14	11.5	4	7	5	13	5	7	1
3395	2	1	1	3	1	1	0	2	0
3396	14	10	6	7	6	13	4	7	2
3397	2	1	2	3	1	1	0	2	0
3398	16	12	6	8	7	14	6	9	1
3399	2	1	2	3	1	1	0	2	0
3400	16	12	5	8	6	14.5	4.5	8	2
3401	49	20	14	26	21	26	14	55.5	60
3402	22	23	9	15	17	28	20	14	21
3403	27	23	8	19	11	29	7	15	4.5
3404	77.5	27	14	24	17	37	16	19	13
3405	27	26	9	23	19	27	8	15	7
3406	34	20	10	18	18	29	12	16	8
3407	26	19	7	18	12	23	7	16	4
3408	26	18	10	20	14.5	23	15	19	4
3409	38	35	24	56	14	40	19	174	23
3410	35	21	9	16	15.5	21	26	14	5
3411	28	16	7	22	12	21	6	14	4
3412	26	20	9	17	13	25	9	14	14
3413	28	25	11	16	22	31	8	17	13
3414	34	20	10	12	13	25	9	13	7
3415	49.5	26	45	27	18	30	22.5	20	14
3416	56	28	15	16	274	39	11	20	14
3417	77.5	14	11	19	15	18.5	15	13	49
3418	19	15	6	16	10	19.5	6.5	12	2
3419	32	20.5	9	31	16	26	8	17	41
3420	31	22	10	26	12	22	9	18	34
3421	35	23	11	22	14	26	10	21	11
3422	45	34	14.5	31	18	38	14	29	24
3423	63	23.5	9	25.5	13	26	11	17	24
3424	43.5	30	17	35	22	33	12	32	23
3425	45	30	14.5	35	16	34	79	26	22
3426	50	24	18	29	17	31.5	10	30	42
3427	30	19	8	19	12	25	8	15	14
3428	36	23	13	28	14	28	10	20	12
3429	45	29	21.5	28	15	29	13	22	25

3430	64	45	20	71	20	52	19	44	99
3431	48	35	14	31	20	32	16	27	29
3432	55.5	40	18	34.5	32.5	40	14	33	40
3433	34	20	10	20	12.5	28	9	20	13
3434	35	24.5	12	30	13	27	10	25	23.5
3435	39	32	15	31	15	36	13	28	23
3436	53	42.5	22	41	20	45	17	39.5	33.5
3437	18	14	7	15	10	16	8	10	5
3438	23	19	6	26	17	21	9	18	10
3439	22	14.5	7	19	11	18	7.5	18	3
3440	29	17	8	17	14	24	11	33	8
3441	25	16	8	27.5	13	20	8	20	18
3442	27.5	16	9.5	18	12	23	10	15	6
3443	21	10	7	23	9	13	6	23	10
3444	137	17	10	30	18	21	13	24	15
3445	35	16	12	28	13.5	19	11	21	65
3446	52	17	9	28	13.5	21	12	22	47.5
3447	759	658	20	40.5	42	2017	44	109	386.5
3448	28	16	11	22	16	17	7	13	34
3449	29	15.5	8	20	15.5	20	7	14	4
3450	27	27	11	22	17	27	20	19	25
3451	43.5	14	9	48	12	18	24	19	9.5
3452	23	13.5	7	47	22	18	69	21	9
3453	27	16.5	10	42	13.5	22	31	36	10
3454	44	18	11	38.5	19	21	53	22	11
3455	41	25	12	28	14	31	12	26	22
3456	31	19	9	22	12	19	7	16	22
3457	34	15	11	41.5	13	19	15	33.5	23
3458	27	11	10	12	14	14	10	13	4
3459	25	14	7	27	10.5	22	37	45	9
3460	21	15	8	13.5	118.5	17	5	13	4
3461	40	18	17	37	29	20	51	30	43
3462	44	17	18	20	11	21	9	14.5	26
3463	26	14	11	22	12	16	28	14	14
3464	23	15	7	14	16	18	10	13	2
3465	24	14	7	19	9	14	8	13	40
3466	342	25	9	26	18.5	52	9	17	13
3467	19	12.5	7	15	11	16	23	32	4
3468	19	12	6	19	10	16	6	13	7
3469	118	86	40	76	42	92	30.5	77	86
3470	628	510	259	374.5	222	546	278	481	473
3471	59	31	17	52.5	21	37	13	34	36.5
3472	168	95	31.5	129	47	91	33	76	67
3473	143	128	63	97	53	128.5	45.5	107	122
3474	175	111	48	181	43.5	118	37.5	110	95
3475	133.5	115	51.5	102	78.5	119.5	49	108	85
3476	112	84	38	79	38	81	34	77.5	80

3477	255.5	208	99	144.5	132.5	222.5	93	178	172
3478	202	155	73	131.5	63	155	60	134	154
3479	55	32	13	46	29	36	12	31	29.5
3480	130	42.5	22	56	27	50	16	42	59
3481	53.5	30	21	56.5	26	33	17	29	45
3482	98	66	33	86	37	69.5	31	64	63
3483	149.5	114	105	92	52	124	42	110	97
3484	173	150	81.5	127	77	161	68	162	169
3485	93	87	49	82.5	42	99.5	37.5	86	70
3486	136	117	70	116	68	124.5	62	120	100
3487	62	41.5	18	51	27	45.5	16	38	30
3488	135	98	46	78	41	107	42	90	79
3489	91.5	70	38	73.5	40	74	33	63	132.5
3490	96	67	33	81	30.5	65.5	29	66	56
3491	116	70	43	72.5	32	73	29.5	61	54
3492	263	84	60.5	77	38	91	32	75.5	88.5
3493	122	103.5	58	90	65	105.5	51	101	167
3494	95	67	35.5	74	30	76.5	48	76	96
3495	70.5	51	36	54	25	53	22	56	57
3496	52	31.5	29	48	21	38	13	34	35
3497	72.5	63	30.5	64	32	64.5	28	65	78
3498	259	65.5	17	54	23	65.5	14	33	69
3499	87	58	30	70	33	63	27	59	56.5
3500	56	37	15	47	27	42	16	29	44
3501	68	57	30	61	43.5	60	24	51	61.5
3502	119	79.5	35	68	39	128	27	64.5	94
3503	67	46.5	24	76	22	55	15	45	37
3504	38	20	13	35	15	24.5	8	22	22
3505	45	24	16	37	20	27	11	24	28
3506	55	35	20	42	21	37.5	19	33	70
3507	63	44.5	21	56	27	54	19	41	79
3508	86	33	26	57	23	38	31	36	29
3509	375	331	186.5	283	166	323	169	296	286
3510	50	29	13	53.5	22	35	14	26	25
3511	90	70	29	60	36	72	25	61	65
3512	119	76	36	82	32	81	31	76	90
3513	59	23	11	43.5	34	25	7	27	26
3514	55	32	12	45	24	36	11.5	51	36
3515	42	19	10	58	13	26	8	42	17
3516	43	24.5	12	51.5	20	29	9	35	26
3517	50	22	10	50.5	28	30	8	43	53
3518	32	19	8	40.5	11	25	8.5	28	11
3519	60	9	8	16	59	11	8	13	14
3520	58	35	12	43	18	38	12	35	32
3521	36	23	10	47	20	27.5	8	41	33
3522	40	22	9	46	14	27.5	8	33	17
3523	41	25	11	43	19	34	11	54	18

3524	31	17	8	45	15	23	7	33.5	23
3525	31	18	10	41	14	21	7	21.5	11
3526	82	58	22	68	23	64	18	64	67
3527	39	25	10	40	16	29.5	11	29	18
3528	35	17	7	43	14	22	7	24	20
3529	34	17.5	8	50.5	14.5	23	7	39	35
3530	57	19	10	52	16	25	7	50.5	46
3531	23	20	7	20.5	9	25.5	16	14	3
3532	28	16	9	27	14	19	7	14.5	4
3533	23	14	7	25	10	17	6	15	2
3534	28	20	8.5	44	16	26.5	7	21	17
3535	25	15	7	28	10	20	6	14	4
3536	39	18	10	44	187	26	11	22	57
3537	49	23	14	69	60	29	26	25	8
3538	42	16.5	10	63	16	21	8	20	22.5
3539	32	19	13	18	22	21.5	9.5	22	5
3540	68	25	17	40	30	29	149	25	31
3541	52	23	19.5	43	83	28	21	24	15
3542	35	15	10	64	83	19	12	26	36
3543	144	20.5	17	56	17.5	18	14.5	44	118
3544	32	16	8	21	10	14	29	12	6
3545	22	14.5	11	20	18	15	28	14	9
3546	50	12	8	21	12	15	6	28	3
3547	27	10	7	20	37	15	6	17	3
3548	35	11	6	35	10	16	6	15	5
3549	23	14	14	15	12.5	16	26	12	8
3550	24	11	6	25.5	97	16	6	14.5	4
3551	20	14	7	12	9	14	8	9	64
3552	28	12	7.5	22	11	15	7	16	6
3553	27	17	18	16	40	16	18	13	7
3554	79	39	43	249	47	86	30	272	35
3555	26	12	8.5	39	9	14	6	21.5	27
3556	33	14	12	29	30	16	18	24	27
3557	26	13	8	73	11	19	12	22	6
3558	21.5	10	6.5	33.5	13	15	6	14	5
3559	19.5	12	6	19.5	9	16	6	11	20
3560	22	11	8	27	11	13	8	14	5
3561	21.5	11.5	6	18	7.5	15	6	11.5	5
3562	20	11	8	27	9	14	7	14	10.5
3563	20	12	8	36.5	25	15	6	27	12
3564	24	11	6	42	13	14	6	21.5	6.5
3565	20	10.5	7	21	18	14	7	14	8
3566	25	13.5	7	26	36	15	7	19	5
3567	25	12	7	24.5	14	15	9	17	13.5
3568	30	12	11	24	13	18	10	21	11
3569	32	17	9	29	66	17	13	29.5	6
3570	30	15	8	38	203.5	17	10	26	11

3571	26.5	13	13	25	1858	14	18	16	22
3572	45	17	13	52.5	12	25	13	43	31
3573	77	17	15	29	42	16	17.5	19	6
3574	28	12	20	55.5	7	17	19	46	4
3575	15	10	5	13.5	13	13	5	10	3
3576	42	25	13	35.5	25.5	26	45	24	29
3577	66	46	28	55	23	52	21	51	59
3578	56.5	36	16	34	20	39	15	33	38
3579	65	43.5	23	52	24	47	22	39	47
3580	97	84.5	37	74	36	85	33	94.5	69
3581	60	45	35	46	20	40	16	37	36
3582	47	23	13	36	12	29	11	23.5	19
3583	40	21	14	63.5	16	26	9	35	17
3584	61	38.5	20	48	23	39	18	40	39
3585	40	14.5	11	36	17	16	17	24	13
3586	54	28	17	42	17	32	13	30.5	33
3587	73	58	31.5	58	26	60	24	56.5	50
3588	36.5	18	15	40	11	21	21	20	23
3589	126	93	42	78	40	96	35	93	83.5
3590	41	24	12	37	12	25	11	24	31
3591	122.5	87	39	69	31	81.5	26	74	84
3592	64	46	23	60	25	52.5	30.5	50	41
3593	54	34	17	51	18	36	15	40	31
3594	67.5	47	26	55	33.5	58	22	57.5	35
3595	33	19	10	35.5	13	23	13.5	24	23
3596	60	44	25	49	34	46	30.5	46	37
3597	79	65	65	65	28	65	27	64.5	63
3598	93	66	43	62	54	73	28	60	63
3599	81	71	30	65	33	81	31	180.5	50
3600	87.5	65	32	68	30	70	27	62.5	63
3601	73	47	22	49	22	45	20	45	53
3602	84	74	36	59	34.5	75	29	65.5	78
3603	134	99	56	105	48	104.5	41	104	95.5
3604	122	83	47	78.5	36	94	41	83	85
3605	122	95	48	95	41	96	40.5	87	85
3606	69	32	17.5	44	23	34	15.5	33	30
3607	53	34.5	15	39	19	38	14	31.5	29.5
3608	72	51	26	60	28	53.5	22	53	58
3609	122	76	43	78.5	35.5	74	38	69	130
3610	52	38	18	52	19	37	18	38	43.5
3611	52	33.5	21	40	17	43	16	40	31
3612	40	20	12	42.5	13	24	10	27	22
3613	77	52	26	57.5	33	55	21	48.5	46
3614	94	56	33	70	37.5	65	36	64	88.5
3615	72	60	28	57	27	63	26	55.5	76
3616	45	21	15	68	18	24	12	37	30
3617	43	16	7	13	8	19.5	6	13	6

3618	73	49	19.5	62	27.5	54	21	44	34
3619	39	25	15	50	19	31	12	29	25
3620	127	72	40	77.5	40	89.5	29	77	76.5
3621	91.5	76.5	35	67	43	86	32.5	66.5	67
3622	82.5	53.5	29	63	33	63	26	50	46
3623	143.5	121	50.5	90	48	121	46	105	113.5
3624	135.5	109	50	86.5	58	109	41	97	100
3625	56	26	16	63	25	33	8	21	20
3626	59	48	11	45	40.5	45	11	24	16
3627	44	24	10	31.5	16	29.5	9	21	38
3628	55	34	18	50.5	27	38	144.5	28	29
3629	33	19	8	44	12	26	6	17	7
3630	25	18	8	13	21.5	23	5	12	5
3631	40.5	25	11	51	26	37	9	36	20
3632	47	27	10	22	12	54	6	15	54
3633	121	26.5	24	34	27	34	62	51	9
3634	60	26	18	46	22	26	12	19	11
3635	23.5	16	5	7	6	22	4	8	2
3636	90	17.5	7	35.5	9	23	11	17	4
3637	71	26	12	22	15.5	56	51	19.5	10
3638	24	17	6	14	8	25	6	12	8
3639	41	18	8	16	14	25	11	16	8
3640	44	20	13	22	87	26	11	16	16
3641	27	17	7	29	11	23	6	13	3
3642	47	19	13	27	17	23	13	19	16
3643	36	17.5	14	17	13	22.5	20	12	21
3644	28	19	16	23	11	22	11.5	16	3
3645	42	22	12	56	19	35	50	68	26
3646	56	28	11	43	12	37	78.5	31	22
3647	234	26	26	60	70	40	125	108	53.5
3648	35.5	17	66	28	96	21	16	18	15
3649	27	16.5	7	22	29	24	14	16	4.5
3650	32	18	8	16	13	23	10	12	8
3651	74.5	22	15	65.5	14.5	24	11	17	10
3652	50	26	13	44	41	33	11	25	32
3653	39	23	12.5	40.5	39	28	13	25	26.5
3654	34	17	12	32	12	24	6	16	24
3655	64	46	20	45	20.5	50	19.5	39	33
3656	53	28	13	40	19.5	35	14	28	20.5
3657	71	52	25	66	25	57	20	51	48
3658	66	50	26	57	27	55	24	47	95.5
3659	83	64	29	76	27	68	25	54.5	51
3660	122	90	54	98.5	49.5	93.5	49	84	138
3661	55	36	24	61	17	42.5	24.5	34.5	38
3662	119	98	46	76	59.5	106	38	86	97
3663	80	60	27	59	29.5	62	23	51	62
3664	80	53	69.5	65	26	59.5	24	53	49

3665	92	66.5	36	81	42.5	75	31	67.5	68
3666	122	92	45	76	41	96	36	85	85
3667	69	35	16	54	37	43	27	36	37
3668	32	20	14.5	37	11.5	23	7	15	13.5
3669	100.5	37	22	52	55	42	14	33	45
3670	50	17	10	38	13	22	7	16	27
3671	90.5	56	29.5	66	34	67	25	54	61.5
3672	35	19	10	51	14	27	8	23.5	18
3673	41.5	19	10	42	20	26	10	17	16
3674	44	15	22	53	13	22	9	23	33
3675	51	26	13	49	12	35.5	8	24	37
3676	31	21	10	34	13	22	11	13	50
3677	36	22	9	31	15	24	7	15	18
3678	54	24	12	48	16	27	12	24	23
3679	55	32	16	48	23.5	39	13	32	53
3680	44	26	13	39	17	28	13	26	62
3681	46	21.5	10	40	10	26	9	17	37
3682	41	25.5	11	38	12	30	11	25	18
3683	42	24.5	12	36	12.5	28	12	25	30
3684	35	15	9	39	14	22	8	18	21
3685	42	31	15	45	46	36	12	32	39
3686	63	43.5	24	56	22	49	18	45.5	57
3687	59.5	41	22	52	19.5	48	18	45	33
3688	74	59	27	62	29	62	21	52	66
3689	121	93	52	79	37	95	36	80	75.5
3690	49	36	22	44	19	37	12	35	28
3691	61	43.5	20	48.5	22	46	19	41	35
3692	76	48	31	63	22	59	25	46	40
3693	107	64.5	32.5	65	28	68	27	59.5	67
3694	36	24	12.5	34	14	29	10	24	26
3695	69.5	47	22	58	22	60	20	54	77
3696	38	46	26	72	37	41	16	41	105
3697	78.5	68.5	28	66	30.5	67	28	60	75
3698	31	14.5	7	28	9	18	8	14	23
3699	28	10	6	26	7	15	5	14	6
3700	24	11	6	24	6	15	12	11	3
3701	26	14	7	22	7	18	7	10.5	12
3702	31	13	9	28	15	14	21	15.5	4
3703	25.5	14	6	37	10.5	20	31	35.5	13
3704	28	12	7	35	9	14	6	9	3
3705	30	13	41	22	161.5	15	11	15	9
3706	46	12	10	16	10	13	7	12	4
3707	39.5	17	9	20	13	27	21	84	5
3708	51	11	5.5	28.5	8	15	8	12.5	14
3709	47	19	19	60	23	30	48	32	42
3710	25	11	7	18	7.5	15	6	21	4
3711	20	10	6	18.5	7	13	6	11	2

3712	20	12	6	19	7	13	17	10	3
3713	18	9	6	23	8	14	5	15	6
3714	44	11	8	22	12	19	11	46	10
3715	23	10	6	26	12	15	21	14	4
3716	64	14	8	18	15	15	6	11.5	6
3717	33	12	7	14	8	16	6	13	4
3718	40	19	17	117.5	27	27	19	68	6
3719	281	13.5	10	28	12	18	9	14	27
3720	42	8	7	20	18	8	6	9	8
3721	36	10.5	13	9.5	14	15	5	12	6
3722	42	10	17.5	22.5	10	15	12	11.5	22.5
3723	27	20	6	12	8	26	5	13	4
3724	23	11	5	16	6.5	15	6	10	2
3725	125	92.5	51.5	98	57	105	43	84	93
3726	53	34	23	41	29	39	10	29	78
3727	53	30	12	67.5	20.5	39	9	34	16
3728	64	40	18	47	23.5	44	15	39	40
3729	37	24	15	55.5	26	30	9	23	25
3730	91	53	26.5	63	33	69	27.5	51	64
3731	48	26	10	54	39	36	8	39	16
3732	51	22	11	42.5	30	29	9	24	29
3733	49	27	12	42	27	39.5	8	24	33
3734	57.5	36	17	44	26	39.5	15	29	39
3735	65.5	33.5	15	44	25	39	14	37.5	22
3736	128	58.5	29	64.5	34	64.5	26	54.5	66
3737	70	54	23.5	50	25	53.5	17	48	32
3738	57	29	17	53	17	57	11	24.5	36
3739	56	32	16	39	21	36	23	32	20
3740	32	18	8	37	18	25	6	20	42
3741	52.5	27	12	32	20	31	10	22	15
3742	41.5	26	11.5	39	23	31	8.5	28	26
3743	31	18	7	29	18	24	10	17	19
3744	83	29	17	48	28	38	13	29	25
3745	59	45	21.5	47	27	45.5	16	43	35
3746	75	54	34	61	35	60	21	53	39
3747	79	49	21	48	21	47.5	16	35	41.5
3748	52	22	21	40	22.5	27	11	31.5	24
3749	36	20	8	30	16	25	8	17	18
3750	40	27	11	48	15	31	12	25	28
3751	204	53	27	63	32	58	50	51.5	54
3752	76.5	57	25.5	59.5	48.5	59	19	58	44
3753	92	75	32	75	32	76.5	29	96	55
3754	40	25	7	18	18	29	6	39	38
3755	38	18	9	53	21	26.5	8	25	70
3756	69	41	18	51.5	27	52	17.5	44	43
3757	38	20	9	47.5	20	25	8	27	10
3758	45	20	9.5	32	20	31	8	27	24

3759	41	24	9	36	18	34	7	20	58.5
3760	47	23	13	50	24	25.5	11	28	14
3761	54	36	17	42.5	25	40	16	33	31
3762	61	32	16	48.5	27	39	14	35	20
3763	36	20	9	36	24	23	8	21	122.5
3764	56.5	35.5	14	50	22	37	13	34.5	33.5
3765	44	23.5	15	43	26.5	31.5	8	25.5	54
3766	25	16	62.5	36	32.5	21	23	15	4
3767	36	19	6	25	29	22	7	17	6
3768	26	16	7	19	12	20	5	14	3
3769	34	16	8	18	14	20	6	11	22
3770	22.5	14	6	9	14	20	4	11	9
3771	32	17	7	61	11	24	6	25.5	10
3772	31.5	17	13	24	15	19	6	15.5	3
3773	61	20	8	54	15	22.5	7	29	6
3774	29	15	28	20	13	21	9	16	3
3775	33	16	11	28	61	21.5	24	13	11
3776	48	19	12	38	35	26	9	20	44
3777	32	16	9	27	44	21	55	18	17
3778	56	24	15	46	149	45	13	29	33
3779	31	19	8	52	18	22	6	31	19
3780	29	15	8	36	16.5	18	6	24	22
3781	33	18	8	61	25	22	8	44	28
3782	29	16	7	57	17.5	21	13	28	9
3783	35	16	10	41.5	20	22	7	26	25
3784	31	17	7	35	18	21	13	20	33
3785	34	19	10	55	23	23	9	34	24
3786	54	22	24	69.5	37	26	7	36	12
3787	26	11.5	7	41	27	16	8	20	18
3788	33	15	8	32.5	13	19	6	21	4
3789	30	16	9	34	12	20.5	6	18	33
3790	24	14	6	12	12	19	6	11	4
3791	28	15	8	17	50	17	6	19	36
3792	31	12	7	35	10	16	7	15	19
3793	29	16	7	22	11	19	6	16	3
3794	23	13	6	11	10	17	11	10	2
3795	24	13	6	23	15	17	5	12	18
3796	69	41	11	20	33.5	158	19	28	25
3797	19	14	5	7	6	17	5	7	1
3798	23	25	6	9	8	32	7	13	2
3799	80	15	7	11.5	15	18.5	7	12	4
3800	33	17	8	22.5	19	19	7	14	16.5
3801	43	21	11	32	37	25.5	10.5	17	10
3802	21	14	6	9	15	17	5	9	5
3803	42	30	179	85	15	37	12	54	7
3804	162	122	55	91	48	125	40	104.5	103.5
3805	74.5	62	27	58	32	70	23	64	78

3806	25	23	8.5	42	12	23	10.5	19.5	88
3807	39.5	31	14	41	17	35	13	28	21
3808	62	51	21	54	27.5	55	17.5	46	38
3809	30	20	14	38	15	28	12	21	13
3810	48	39.5	19.5	55	30	46	17	43	72
3811	46	34	14.5	48	22	39.5	15	40	28
3812	35	27	14	38	23	34	13	27	21
3813	30.5	21	18	42	125	25	40	23	17
3814	28	20	28	52	19	26	28	30.5	10
3815	60	21	17	56	14	29	22	20.5	12
3816	68	18.5	11	25	13	26	15	28	7
3817	24	20	12	33	16	25	9	31	27
3818	22	17	8	16	11	25	5	11	7
3819	61	22	14	33	22	23	13	21	11
3820	33	17	11	38.5	18	22	11	25	26.5
3821	74	21	19	29.5	32	27	11	52	73
3822	96	52	16	24.5	19	137	33	23	24
3823	28	20	12	25	13	21	10	18	14
3824	28	15	12	23.5	13	25	12	17	10
3825	33	18	7	22	13	25	7	16.5	9
3826	65	26	16	49	20	33	11	72	49
3827	31	25	8	54	17	31	11	84.5	15
3828	23	16	9	18	12	22	8	15	5
3829	24	16	7	32	17	21	17.5	26	30
3830	24	18	13	33	13	24	14	18	8
3831	24	16	9	25	28	23.5	12	15	4
3832	76.5	19	10	24	12	21	11	16	8
3833	30	21	11.5	41	14	27	9	41	6
3834	26	20	7.5	25	13	23.5	9	18	7
3835	24	15.5	7	25	13	20.5	10	18.5	5
3836	21	15	10	19	13	21	8	17	5
3837	26	18	13	24	19	19	8	14	6
3838	38	17	9	49	14	23.5	10	23	4
3839	28	19.5	10	26	20	23	72	31	41.5
3840	17	16.5	8	13	12	20	10	11	4.5
3841	31	22	11	39	16	27	9	23	16
3842	56.5	44.5	22	51	25	53	18	45.5	43
3843	82.5	64.5	26.5	55	39	70	24	60	59
3844	105	82	41	79	38	79	32.5	82	86
3845	45	33	18	41	15	36	12.5	32	34
3846	63	56	25	50	56.5	57	20	47	47
3847	62	46	23	57	27	53	21	45	87
3848	25	18	10	37	13	24	9	21	29
3849	37	19	10	33	11	24	9	19	14
3850	57	40	18	48	19	46	16	37	32.5
3851	28	19	11	37	14.5	23	9	19	20.5
3852	73	55	29	61	28	59	23	53	65

3853	28.5	19	10	23	11	20.5	8	17	16
3854	36.5	17	6	35.5	19	20	7	19	12
3855	83	73.5	37	82.5	33	83	30	65	67
3856	45	37	18	52	25.5	40	16	37	34
3857	27.5	22	14	27	14	29	11	17	19
3858	61	51	19.5	43	23	53	18.5	42	43
3859	65	51	24.5	71	30	60	21	56	44
3860	52	41	22	47	21	43	14	40	65
3861	55	46	18	39	19	46	16	32	35
3862	88	65	32.5	80	34	66	44	68	59
3863	109	87	54	103	50	91	54	88.5	81
3864	63	47	23	50	23	49	18	47	46
3865	75	63	34	83	31	64	34	61	62
3866	41	31	20	55.5	29	35	17	35	23.5
3867	72	80	20	48	22	55	20	40	58
3868	64	49	22	50	24	57.5	20	45	44
3869	27.5	19	14	24.5	12	20	15	14	9
3870	19	14	12	33	10.5	20	8	15	3
3871	22	15	8	29	12	18	7	20	7
3872	22.5	13	14	24	12	17	70	16	8
3873	22	14	35	31	13	18	6	11	18.5
3874	23	16	16	67	13	41	10	174.5	13
3875	22	17	7	34	10	18	9	19	19
3876	17	14	7	15	9	18	6	9	4
3877	32	19	10	33.5	18	24	9	28	9
3878	20	14	6	30	11	20	8	14	4
3879	34	15	9	43	47	19	7	30	77
3880	41	23.5	10	44	32.5	28	8	63	50
3881	26	15	21	33	14	22	13.5	21	12
3882	29	18.5	11	40	14.5	23	16	21	19
3883	27	17	8	47	20	20.5	9	37	11
3884	23	15	8	42	12	21	7	25	17
3885	16	14	8	15.5	11	17	7	13	3
3886	50	24	9	24	11	31	9	18	4
3887	35.5	28	10	55	20.5	38	9	42	21
3888	32	25	11	28	18	34	11	17	29
3889	25	22	7	27.5	24	32	7	22	5
3890	19	17	7	17	10	24	7	13	5
3891	29	34	10	26	43	32	27	17	9
3892	26	14	11.5	32	19	18	8	31	18
3893	112	97	69	116.5	54	97	52	283	73
3894	37.5	26.5	24	50	26	28	19	97.5	38
3895	200	126	76	121	78	130.5	67	128	131.5
3896	56	39.5	23	55.5	30	45	15	53	21
3897	83	70	44	80	38	70.5	31	114	50
3898	72	58	38	72.5	37	58	32	119	47
3899	106	63	36	88	32.5	66	32	83	71

3900	78	59	35	64	35.5	62	26	95.5	42
3901	37	23	16.5	42.5	20	25	11	33.5	19.5
3902	39	22	14	37	16	26	10	49	37
3903	38	26	22.5	41	22	30	14	73	27
3904	34	24	16	36	17	29	11	43	13
3905	70	46	27.5	56	32	41	25	100	43
3906	92	87	57	108	66	94	45	371	89
3907	60	31	19	41	21	33	16	61	35
3908	52	36	23	49	23	43	16	57	29
3909	162	23	17	35	44	28	13	37	12
3910	66.5	60	46	82	33	56	28	175.5	67
3911	74.5	65	33	60	29.5	67	24.5	89.5	57
3912	52	34	19	46	27	31	16	50.5	23
3913	121.5	93	48	78.5	45.5	87	33	96.5	102
3914	68	53	32	51	34	55.5	23	78	41
3915	40	28	21	58	140	29.5	15	58.5	28
3916	31	21	20	44.5	23.5	16	14	97	18
3917	107	97	52	81	55	97	39	123.5	99
3918	37	25	15	37	16	24	12	59	26.5
3919	41	32	18	33	20	32.5	14	41.5	28
3920	72	57	38	61	34	53	24	124.5	43
3921	75	57	35	65	37	56	25	72	201
3922	74	43	27	51	29	45	22	68	38
3923	62	43	24	46	27	46	20	51.5	33
3924	148.5	133	93	115	91	130	68	123	137.5
3925	37	20	14	39	18	23	17	42	36
3926	89.5	56	29.5	66	33	55	24	73	53
3927	70	49	33	57	35	48.5	26	70	55
3928	67	42.5	24.5	70.5	25	45	21	63	62
3929	161	125	66	106.5	54.5	125.5	59.5	123	123
3930	68	56	32	59	49	55.5	27	64.5	54
3931	44.5	33	23	41	20	35	15	47.5	38
3932	45	32	19	103	21	34	20	80.5	20
3933	64	40	29	52	27	41	22	55	36
3934	58	41	32	51	33	39	19	143.5	53
3935	22	14	9	20	30	14	7	22.5	6
3936	22	14.5	12	26	18	16	8	33	4
3937	23	16	11	27	17	18.5	9	36	6
3938	18	16	15	17.5	15	18	8	27.5	13
3939	25	18	15	20	15	21	10	31	4
3940	21	16	11	24	16	19	9	37.5	3
3941	61.5	20	29	31	27	21	24	169.5	30
3942	33	15	13	42	27	17	9	44	10
3943	26	15	17.5	30	15	15	9	48	29
3944	56	50	95	172	58.5	50	71	605	19
3945	50	16	30	27	21	19	41	47	14
3946	31.5	21	19	33	31	21	14	47	19

3947	14	8	6	10	12	9	6	15	4
3948	17	12	9	13	15.5	12	7	19	3
3949	20	12	7	13	20	14	7	19	3
3950	22	20	16	31	22	19	14	92.5	17
3951	18	13	13	17	13	14	9	29	6
3952	21	15	13.5	23	18	16	10	44	4
3953	88	31	32	110	80	30	56	135	24
3954	23	15	23	34.5	25	17	15	38	6
3955	16	10	17.5	24.5	31	10	11	89	5
3956	24	11	10	16	35.5	11	8.5	24	3
3957	19	12	8	17	13.5	14	21	18	2
3958	38	18	16	30	35.5	18	33	36.5	24
3959	34	24	26	46	38	21	19	130	146
3960	46.5	22	29	110	23	26	13	98.5	9
3961	62	25	20.5	80	21	34	20	208.5	16
3962	18.5	8	9	29	13	8	6	23	52
3963	27	20	29	73	31	19	17	186.5	5
3964	21	12	11.5	35	15	14	7	34	13
3965	21	13	10	43	15	15	8	34.5	12
3966	33	17	30	39	32	16	59	58.5	18
3967	26	15.5	12	24	15	16	8	28	27
3968	25	15	12	38	17	17	9	41	15
3969	28.5	19	10	47	15	22	8	41	13
3970	72	37	48.5	67	34	43	14	82	61
3971	129	62	40.5	216.5	59	85.5	40	711	62
3972	105	8.5	8	63.5	13	8	12	39	82
3973	23	8	32.5	31	12	10	8	23	9
3974	30	10	7	34.5	23	12	6.5	33	8
3975	25	11	8	34.5	16	13	8	28	37
3976	22	13	11	36	16	13	9	38	38
3977	31	14	9	35.5	16	16.5	8	32	36
3978	28.5	17	12	45	44	19	10	36	18
3979	47	17	13	40.5	17	20	8	41	11
3980	30	20	29.5	38	30.5	19.5	17	235	8
3981	26	12	21	31	29	13	9	96.5	13
3982	19	9	8	15	12	8	7	26.5	72
3983	50	18	19.5	22	15	16	8	34	36
3984	32	19	13	47	55	17	12	40.5	38
3985	23	12.5	20	29	36	12	15.5	47	76
3986	87	46	16	62.5	26	21	16	58.5	52
3987	31	18.5	20	58.5	24	20	13	51.5	22
3988	17	9	10.5	26	86	7	8	29	85.5
3989	39	12	12	27	15	12	9	57.5	29
3990	28	11	21.5	20	16	9	12.5	35.5	8
3991	19.5	11.5	16	28	12	13.5	8	50.5	3
3992	31	20	14	35.5	19	19	11	36	25
3993	132	103	55	93	42.5	105	43	124	95

3994	21	13.5	15.5	27	14	14	9	48.5	25
3995	43	29	17	46	19	27	14	45	27
3996	26	10	8	22	22	10	7	20.5	25
3997	41	27	15	36	20.5	26	13	46	24
3998	86	70	36	70	41	68	32	110	60.5
3999	99	40	18	41.5	35	35	17	59	45
4000	34	20.5	16.5	41	24	18	13	50.5	16
4001	54	37	23	48	31	35	19	60.5	31
4002	47	28	23	43	22	32.5	16	65.5	26
4003	69	49	30	63	34	49	23	100	51
4004	21	9	12	26	16	10	9	40	46
4005	42	29.5	19	35	24	28	15	44.5	34
4006	30	16.5	14	30	19	17	9	38	14
4007	23	12	10	40.5	28	12	8	26.5	40
4008	56	37.5	34	84	37	40	23	210.5	33
4009	58	45	29	50	31.5	45	24	96	41
4010	61	40	23	49	23	43	18	61.5	59
4011	30	18	11	29	14	18	10	24	28
4012	43	16	11	22	15	17.5	27	24	47
4013	62	42	21	38	22	39	18	42	44
4014	25	13	15.5	29	15	14	12	32	13
4015	24.5	15	9.5	27	31	15	9	25.5	15
4016	42	27.5	17	32.5	21	25	14	43	46
4017	107	73	51	76	40	69	35	153.5	58
4018	28	20	13	32	17	17	10	46.5	21
4019	70	53	32	55	33	54	26	88.5	72
4020	46	29.5	17	30	21	30.5	13	43	30
4021	20	11	10	32	13	9	7	32	26.5
4022	22	12	9	26	17	11	9	33.5	67
4023	81.5	72.5	50	82	41	66	35	242.5	63
4024	45.5	25.5	19	45	24	25	14	50.5	38
4025	19	10	8	13	11	8	9	22	2
4026	47	31.5	16	33	32	28.5	13	40	33
4027	74	59	39	63	40	59	31	119.5	54
4028	53	29	20	40	22	26	14	59	45
4029	68	47	23.5	59.5	26	48	19	64	50
4030	31	15	17	50	23	14	12	89.5	14
4031	30	19	19	32.5	17	18	10	36	18
4032	26	11	8	30.5	13	10	7	26	32
4033	32	19	13	38.5	28	19	10	43	23
4034	30	19	14	35	18	19	14	48	33
4035	137	146.5	96.5	132.5	84	123	82.5	298	124.5
4036	33	20	16	36	21	20	12	48	17
4037	32	24	12	39	16	24	10	34	67
4038	43	31	22	43	32	28	15	58.5	54
4039	81	54	29	48	24	48.5	20	61	48
4040	43	32	17	39	18	31	13	49.5	73.5

4041	24.5	13	12	28.5	20	11	8	58	37
4042	43	37	25	58	25	31	18	69.5	22
4043	60	49	27	48	24	48.5	21	63	51
4044	86.5	68	34	61	36	67	29.5	101	62
4045	49	43	31.5	51	30	41.5	26	128	32
4046	41	44	53	80	50	41	50.5	445	101
4047	19	10	24	22	19	9	13	82	5
4048	31	24	31	53	65	23	22	284	9
4049	15	8	6	12	12	6	6	12	4
4050	55	10	8	23	27	10	22.5	20	3
4051	21.5	11	15	20	13	10	7	32	5
4052	27	20	13	37	39	14	12	80	6
4053	17.5	12	11	19	11	13	9	21	5
4054	22	11	8	17	63	11	11	19	6
4055	26	16	9	26	12	17	9	24	28
4056	19	14.5	9	14	15	17	7	20	2
4057	23	19	12	28.5	36	19	25	100	4
4058	16	11	7.5	19	13	14	9	18	2
4059	103	48	68	149	74	47	15	99	48
4060	36	23	17	32	24	25	12	49	36
4061	24	16	12.5	33.5	16	17	9	26	17
4062	51.5	43	26	49	31	42	24.5	73	37
4063	27	15	10.5	24.5	44	18	9	34	18
4064	47	35.5	18.5	47	24	38	15	58	47
4065	37	27	21	44	36	28	14	72	36
4066	33	21	12	25	21	25	10	44	28
4067	29	23	14	28	21	20	13	54.5	20.5
4068	19.5	11	14.5	23.5	15	14	11	30	9
4069	24	14	9	22	17	15	6	27	49
4070	52	40	21	45	27	37	22	75	41
4071	74	55	27	59.5	29	54	91	63	86
4072	61.5	50.5	31	50.5	33	51	23	104.5	125
4073	26	19	13	32	21	18	9	47	28
4074	39	28	16	38	27	28	17	108	44
4075	55	36	19	48	27	40	26	56.5	47.5
4076	53	40	23	44	32	43	18	56	39
4077	35	26	22	37.5	25	29	13	44	37
4078	48	17	13	29	16.5	17	10	61	30
4079	41	34	22	36	28	34	16	72.5	42
4080	22	12	10	26.5	18	12	10	32	75.5
4081	52	40	20	44	23	37	18	58	32
4082	16	12	9	19	10	11	8	25	4
4083	15.5	9	8	15	11	13	7	20	4
4084	23	20	16	23	18.5	16	13	27	9
4085	16	11	10.5	14	13	13	7	17	3
4086	41	23.5	34	52	55	29	23	260	8
4087	14	11	6	9	6	11.5	5	13	2

4088	19	13	8	11	13	15	7	16	4
4089	11	12	6	10	8	11	6	14.5	3
4090	22	12	7	17	26	12	7	19.5	18
4091	18	18	16	20	22	17	10	41	10
4092	25	17	16	23	15	17	10	46	15
4093	33	26	26	77.5	28	25	16	256	18
4094	16	11	9	23	11	13	12	25	5
4095	18	13	9	26	15	14	26	27	14
4096	86.5	69.5	33.5	60	35.5	70	28	103	68
4097	69	50	22	45	28	51	38	52	43
4098	54	37	20	43	29	41	22	49	39
4099	110.5	68	37	66	36	74	28.5	89	69
4100	44	28	14	46	24	32	15	46	16
4101	53	42	21	48.5	27.5	41	21	45.5	51
4102	99	33	17.5	38.5	27	31	20	38	21.5
4103	83	70	30	64	141	72	35	90	66
4104	83	59	25	57	32	64	29	62	48
4105	198	186.5	63	96	64	177	51	158	172
4106	119.5	90	37	82	42	96	27.5	86.5	65
4107	68	43.5	25	46	32	48	19	58	36
4108	107	91	55.5	93	74	91	49	102	83
4109	82	53	23	43	35.5	58	22	56	46
4110	100.5	90	41	77	65	87	35	87	87
4111	51	37	19	40	44	40	16	51	36
4112	77	64	27	52	38	62	26	68	71
4113	75	56	35	53	45	60	22	73	52
4114	72	63	31	66	32.5	63	27	102	62
4115	56	45.5	19	69	27	49	16	62.5	31
4116	71	61	28	54	31	62	24	86.5	51
4117	154	105	45	79	58	107	42	108	89
4118	57	43	23	43	27.5	44	18	50.5	45
4119	81.5	72	32	54	35	73.5	26	70	59
4120	37	23	12.5	40	18	27	10	50	15
4121	33	23	21	42	21	26	11	42.5	19
4122	68	52.5	33	50	30	60	24	60	50
4123	44	28	16	42	23	31	24	41	20
4124	81	60	31.5	55	35	64	24	78	51.5
4125	111	99	50	74	56	93	39	92.5	72.5
4126	103	82.5	44	77	44	85	37	99.5	74
4127	79	67	34	59	30	71	27	78.5	58
4128	74	55	33	55	49	59	26	69.5	59
4129	112.5	78	37	70	43.5	77	34	99	68
4130	140	116	61	85	51	119	48	130	140
4131	49	37	19	44	27	35	15	48	32
4132	39	17	9	26	23	21.5	9	33	32
4133	39	29	17	40	20	30	12	43	42.5
4134	29	16.5	15	28	46	19	8	38.5	22

4135	19	15.5	7	10	11	15	7	15.5	6
4136	18	17	7.5	14	27	16	6	27	13
4137	18	14	7	10	12	16	6	18	2
4138	24.5	19	17	24	24	19	15	39.5	13
4139	48.5	30	64	31	44	21	9	61.5	10
4140	25	15	8	18	18	20	7	21	12
4141	24	14	9	18	14	16	7	23.5	5
4142	29	16	11	22	14	18	8	24	21
4143	26	17.5	61	19	30	20	9	53.5	39
4144	20	11	7	8	20	16	6.5	15	3
4145	26	16	22	27	36	19	11	25	72
4146	30.5	15	8	14	20.5	18	8	22	7
4147	38	19	12.5	39	33	20	15	42	15
4148	47	21	21	43	35	21	42	39	19
4149	35	22	12	40	13	20	8.5	36	10
4150	32	16	9	29	11	18	7	29.5	66.5
4151	26	14	8	27	13	17	7	26	53
4152	30	12	10	21	13	14	7	21.5	60
4153	26	51	11	20	11	18	8	21	32
4154	30	22	9	30	14	20	10	23	30
4155	25	16	13	35	16	18	11	80.5	24
4156	32	24	12	33	27	16	12	29	46
4157	32	14	11	49	19	18	9	41	43
4158	34	14	13	28	20	15	10	57.5	8
4159	50	18	11	20	33.5	21	16	91.5	13
4160	32	18	15	46	24	21	11.5	102	6
4161	21	16	8	16	27	17	8	36	7
4162	29.5	17	9	20	18	19	8	36	8
4163	22	14	9.5	15	19	15	7	25	8
4164	19	14	11.5	17	19	15	29	23	5
4165	18	12	10	13	20	14	7	36.5	4
4166	22	13	19.5	25	17	15	34.5	38	3
4167	21	13	8	25	16.5	16	9	72	11
4168	20	13	16	19	17	12	10	21	4
4169	53	16	11	19	17	16	21	23.5	36.5
4170	27	15	36.5	16	16	14.5	11	38	6
4171	187.5	281	53.5	203	47	228	43	5178.5	28
4172	85.5	27	20	71.5	62	22	310.5	164	21
4173	19	9	8	14	16	9	31	13	3
4174	81	97	16	26	187	98	27	1185.5	30
4175	21	15	14	27	17	18	13	102	7
4176	18.5	12.5	10	12	19	10	20	17	4
4177	18	8	6	16	12	9	5	17	11
4178	22	9	10	20	12.5	11	9	29	6
4179	25	12	14	58	19	16	12	42	10
4180	22	14	14	35	18	15	11	25	10
4181	16	12	12	17	13	9	7	25	7

4182	22.5	13	8	26	19	12	9	23	5
4183	23.5	12	22	19	14	11	9	28.5	11
4184	22.5	13	11	30	18	14	55	87.5	7
4185	63	20.5	15	139	30	29	93	209.5	41
4186	38	15	20	46	447	13	81	56	57
4187	29	10	10	26	28	12	9	32	35
4188	21	11	8	17	16	11	6	21	33
4189	14	7.5	7	12	13	10	6	18.5	2
4190	17	9	7	12	28	12	9	14	1.5
4191	28	13	58	35.5	15	14	15.5	37.5	36
4192	22	12	9	12	20	11	8	30	5
4193	17	12	10.5	18	17	10	13	32	29
4194	21	12	11	31	14	15	10	102.5	6
4195	29	13	11	20	31	16	9	60	4
4196	91	13	14	41	29	12.5	30	43	41
4197	38	11	9	42	24	12	10	37.5	61
4198	21	15	74	24	13	14	11	92	6
4199	17	11	8	15	29	11	9	34	4
4200	25	12	9.5	23	283	14	8	73.5	8
4201	89	67	30	66	34	71	24	81.5	91
4202	25	17	15	29	14.5	18	10	26	21
4203	38	16	20	26	17	16	17	34	16
4204	21	11	11	34	19	13	12	37	22
4205	77	44.5	21	45.5	29	43	18	50	76.5
4206	43	32	24.5	36	23	30	17	44	47.5
4207	24	13.5	10	21	17	16	9	54	12.5
4208	26	14	10	33.5	17	15	9	29	15
4209	34	23	46	37	27	22	27.5	93.5	28
4210	35	12	12	26	14	14	12	27	14
4211	22.5	10	13.5	23	22	10	7	25	22
4212	20	11	9	28	12	10	8	23	12
4213	23	12	13	20	19	13	8	28.5	17
4214	22	12	13	37	15	12	8	40.5	14
4215	26	14	21	28	14	16	7	79.5	15
4216	35.5	17	11	32	74	16	13	31	21
4217	20	11	8	22	12.5	11	8	23	20
4218	18.5	7	8	17	12	10	7	20.5	21
4219	23	19	9	29	11	12	11	30	25
4220	37	25	16	36	19.5	25	15.5	66.5	22.5
4221	28	18	12.5	27	18	16	12	31	34
4222	29	14	13	32	23	16	11	51	17
4223	36	22	11	37	16	22	11	33	17
4224	23	11	9	36.5	14	12	8	26	13
4225	25	11	9	36	17	12	9	29	6
4226	38	12.5	10	23	15	13	9	27	29
4227	65.5	43	26	62	25	41	33	59	61
4228	25	10	8	22	14	10	10	24	14

4229	32	16	13	49	16	18	9	35	25
4230	115	18	13	27	26	18	10	26	16
4231	21	10	9	24	27	10	8	23	13
4232	56	27	14	31	24	25	15	39	37
4233	23.5	12	9	32	16	13	7	34.5	9
4234	31.5	15	12	26	14	13	8	26	15
4235	54	31	17	37	22	27	13	34.5	31
4236	75	48	32	55	27.5	57	28	66.5	53
4237	63	37	20	41	23	39	18	48	49
4238	32.5	11	14	22	15	12	7	47.5	24
4239	18	9	8	18	13	9	7	21	4
4240	33	10	9	21	62	12	10	25	11
4241	23	10	8	16	16	10	7	22.5	3
4242	19	10	8	20.5	85.5	12	9	54	5
4243	33	17	20	38.5	56	24.5	24	212.5	38
4244	36	11	9	45	14.5	13	8	54	5
4245	41	11	16	62.5	73	12	24	31	54.5
4246	24	11	11	22	18	12	12.5	59	7
4247	17	8.5	9	15	11	10	7	26	10
4248	33	11	11	41.5	16.5	13	8	59	4
4249	35	11	15	62.5	75	11	21.5	34.5	43.5
4250	22	12	11	21	15	10	11	40	6
4251	25	12	16	45.5	50	12	9	36	36
4252	23	12	11	33.5	16	12.5	8	29	7
4253	49	23.5	23	29	26	27	41.5	136	16
4254	33	26	15	22	20	30	10	29	8
4255	38	11	8.5	17	24	11	15	27.5	10
4256	38	22	10	50	30	23	9	27	28
4257	25	19	9	19	14	22	8	14	4
4258	39	21	11	46	27	29	9	32	16
4259	62	23	14	57	33	26	12	48.5	19
4260	32	23	9	27	17	30	7	21	6
4261	40	24	10	52	27	27	8	29	58
4262	109	25	17.5	64.5	37	27	15	29	346
4263	50	27	20	52.5	37	30	13	30	203
4264	46.5	26	16	51	27	25	12	37.5	85
4265	49.5	23	15	49	22	25	11	24	56
4266	50	29	19	32	34	45	9	34	12
4267	708	377	36	69	63.5	1403	26	79	167.5
4268	96	25.5	30	46	32.5	31	23	27.5	42
4269	40	22	11	60	63.5	27	10	29.5	150
4270	32	17	7	22	26	21	7	16	11
4271	33	16.5	14	34	38	21	12	24	14
4272	36	17	9	61	17	21	14	25	27
4273	41	22	18	38.5	19	25	10	17	31
4274	35	22	10	41.5	18	26	14	25	10
4275	28	18	9.5	36	32	24	8	50.5	16

4276	28	17	8	31	15	21	7	23	15
4277	76	61	36	48.5	26	63	23	80	59.5
4278	67.5	52.5	22	51	30	52	17	93.5	45
4279	52	35	21	41	21	39	14	69	27
4280	46	36	19	42	23	39.5	13	54.5	28
4281	85	53	32	63	39	59	26	70.5	58
4282	71.5	52.5	30	67.5	31	56	18	71.5	54
4283	41	27.5	21	44.5	19	32	11	120.5	21
4284	198	179	77	126	215.5	179	52	191	166
4285	54	42	18	49	63	43	13	68	61
4286	29	19	22	29	13	20.5	8	68	25
4287	26	12	10	24	18	12	6	37.5	20
4288	25	15	10	25	35	16	8	44.5	12
4289	42	25	16	44	22	28	10	57	21
4290	65	52	23	55	36	52	18	60	44.5
4291	63	52	26	50	25.5	54	21	59.5	68
4292	25	17	8	20	8	20	6	23	7
4293	45	28	15	35	24	32	10	54.5	21
4294	42.5	25	14	30	41	27	10	47	28
4295	25.5	14	7	22	16	16	6	25	17
4296	44	25	13	35	21	26	10	50	22
4297	53	23	16	38	37	27	11	56.5	23
4298	76	59	33.5	53.5	29	65	26	85	72
4299	50	36.5	19	42	23	36	17	61.5	36
4300	29	20	10	29	15	24	8	37.5	42
4301	35.5	18	12	31	32	21	12	37.5	46
4302	39	25	9	31	14	29.5	8	35	41
4303	65.5	52	31	52.5	30	54	21	76.5	82
4304	90.5	76	37.5	72	31	77	32	102	85
4305	33	24	17	30	13	26	11	36	35
4306	41	14	11	29	11	15	7	37	14
4307	39	19	12	30	13	20	12	82.5	30
4308	50	30	17	41.5	26	30	14	32	25
4309	28	15	8	25	23	16	6	27	33
4310	49	20.5	16	60	25	21	11	59	21
4311	29	14	12	27	14	15	10	40	19
4312	84	62	31	64	35	61	25.5	80	54
4313	27.5	16	13	32	23	19	8	92	18
4314	20	11	9	27	10	13	7.5	33.5	13
4315	29	19	10	34	12.5	22	7	50	20.5
4316	43.5	31	17	44.5	22	32	11	46	36
4317	33	23	20	45	39	27	18	316	46
4318	25	12	9.5	46	15	16	11	62	11.5
4319	15	9	6	15	6	11	4	13	3
4320	11	10	8.5	16	8	12.5	5	9	7
4321	17	9	8	32	8	11	5	22	8
4322	20	11	6	42	10	13	8	35	5

4323	27	16	8	34	9	18	7	24	50
4324	23	13	8	41	12	16	13	46.5	6
4325	88.5	23.5	21	45	22	27	19	201	19
4326	105	13	22	21	19	14	11	79.5	10
4327	21.5	10.5	8.5	14	380	13	7	25	11
4328	20	8	9	23	15	10	7	19	4
4329	48	17	26	35	19	17	11	42	24
4330	26	12	16	32	14	13	10	69	9
4331	36	17	19	29.5	113	21	12	237	15
4332	24	13	18	57.5	21	16	18	30	11
4333	17	12	7	18	9	14	6	29.5	12
4334	28	15	9	29	9	16	7	27	29
4335	40	7	9	23	18	9	14	52.5	4
4336	27	12	8	15	9	13	9	23	7
4337	207.5	17	17	38	53	18	44	27	17
4338	32	14	13	33	88	18	11	195	6
4339	39	12	13.5	36	20	13	9	45	10
4340	48	24	16.5	41	17	24	13	94.5	54
4341	31	24	18	23	33	16	14	61	18
4342	22.5	13	10	25.5	60	14	14	22	35
4343	47	12	9	22.5	14	12.5	42	48	7
4344	53.5	16.5	23.5	55.5	17	17	28.5	161	6
4345	71	45	23	65	32	47	16	64.5	42.5
4346	31	15	8	32	17	17	8	29	22
4347	48	38	16	33	18	37	12	41.5	32
4348	89	66	32	63	29.5	62	19	92.5	86
4349	69	36	16	42	27	35	12.5	42.5	32
4350	35	22.5	14.5	33.5	12	20.5	8	32	27
4351	65	45	21	51	24	47	18	60	52
4352	105.5	62	27	61	28	63	20	71	58
4353	76	63	30	58	31	61	23	70	68
4354	74	57	25	57	27	55	21	66	45
4355	95	69	29	58.5	33	70	26.5	76.5	58
4356	72	53	21	52	30	58	19	61	54.5
4357	43	15	23	60.5	23	17	13	64	54
4358	19	8	8	16	42	9.5	6	20	3
4359	22	9	9	39	17	10	10	30	30
4360	26	9	7	33	7	10	6	20.5	8
4361	28	13	11	31	29	12	8	34.5	22
4362	19	10	8	26	7	13	8	55	162
4363	17	10	9	22.5	10	10	6	71	5
4364	25	13	52.5	26	32	14	21	241.5	6
4365	10	6	5	10	6	6	5	13	2
4366	11	7	5	8	6	9	5	11	4
4367	13	9	8	17	8	9	7	40	2
4368	16	12	18	23	26.5	13	10	207	10
4369	12	8	7	9.5	9	7	5	30	3

4370	25	9	9	33	11	9	7	21	9
4371	59	9	9	33.5	15	10	8	34.5	81
4372	26	8	7	10	8	7	6.5	13.5	9
4373	97	30	80	45.5	17	30	15	179	11.5
4374	22	11	9	14	13	10	7	24	3
4375	43	19	11	51	908	14.5	9.5	44	75.5
4376	14	5	6	14	8	6	5	18	4
4377	55	37	18	38.5	31	37	19	41.5	72
4378	35	25	18	39	16	28	12	37	42
4379	54	40	22	41	28	39	17	55	52
4380	30	8	8	20	11	9	7	25	19
4381	68	57	36	50.5	32	58	24	65	54.5
4382	33	13	12	27	18	12.5	10	28	27
4383	37	30	17	33	18	32	17.5	49	39
4384	44	33	19	38.5	19	31	15	40.5	37
4385	18	9	13.5	21.5	11	10	7.5	31	17
4386	46	30	25.5	51	22	33	19	54.5	47
4387	63	51	29	46	30	51	24	86	55
4388	23	8	10	19	16	9	8	26	33
4389	29.5	11	14	28.5	15	14	9.5	33	20
4390	19	8	8	22.5	8	8	6	20	24
4391	16	8	7	25.5	10	8	5	18	12
4392	19	7	7	21.5	8	6	5	22	17
4393	31	18.5	10	35	15	17	9	30	17
4394	17.5	7	10.5	26	7	7	6	29.5	3
4395	63.5	21	11.5	28.5	13	21	9	30	15
4396	26	12	12	22	11	14	8	24	17
4397	40	29.5	15	29	14	28	12	32.5	30
4398	35	30	14	36	19	22	11	75	27
4399	38	22	15	31.5	17	21	11	44.5	24
4400	21	7	7	17	12	8	6	36	43
4401	21	21	9	23.5	10	13	7	31	12
4402	17	7	5	28	13	8	6	27	12
4403	49	40	23.5	42.5	24.5	41	17	63	37
4404	31	17	11	31.5	11	16	8	36.5	16
4405	52	43	24	49	28	44	21	85	60
4406	31	18	12	31	14	19	10	34	25
4407	36	16	10	31	12	17	10	24.5	40
4408	23	12	12	25	9	14	5	26	20
4409	28	17	12	31	12	19	10	28	21
4410	31	17	13	35	11.5	20	8	23	27
4411	19	10	9	25.5	10	10	7	70	15
4412	31	17.5	9.5	25	18	18	9	27.5	18
4413	23	8	8	23	23.5	9	5.5	20	73
4414	30	19	10	28.5	13	18	9	26	30.5
4415	33	22	14	26.5	14	21	10	34	61
4416	17	11	8	18.5	9	11	6	19.5	18

4417	14	6	7	17	8	7	5	18.5	18
4418	28	17	12	25.5	12	18	11	34	18
4419	40	29	19	48.5	22	30.5	14	42	26
4420	55.5	40	21	39	20	41	17	44	50
4421	45.5	36	23.5	39	17	35.5	17	47	36
4422	33	19	13	24.5	17	18	10.5	32	19
4423	46	35	16.5	29	21	42.5	14	34	43
4424	32	14	11	21	15	14	9	30.5	18
4425	30.5	23	13	35	16	24	11.5	39	53
4426	64.5	55	27	50	24	53	65.5	57	57.5
4427	44	31	22	32	19	31	22	40	38
4428	42	7	7	18	8	7	19	15	13
4429	20	8	9	26	13	7	9	22	38
4430	25	14	10	27	18	13	7	85.5	21
4431	24	11	11	31	11	12	10	46	10
4432	32	17	12	29	20	17	19	26	23
4433	30	22	13	32	15	21	11	30	31
4434	38	23	15	36	14	25	10	57	35
4435	40	24.5	17	35	15	26	12	33	23
4436	45	34.5	17	50	21	34	16	58	38
4437	20	10	6	26	8	11	7	21	5
4438	72	61.5	32.5	55	26	63	27	70.5	61
4439	26	24	29.5	36	23	32	12	20.5	22.5
4440	43	21	40.5	20	31.5	22.5	14	26	36
4441	62	26	22	24	26	42	15	34	24
4442	20	18.5	26	25	18	26	11	14	13
4443	22.5	15	21	24	16	19	13	20.5	10
4444	17	13	23	24	15	20	8	13	17
4445	69	35.5	61.5	35	39	79	21.5	36	47
4446	44	24	77	30.5	36	35	17	32.5	27
4447	18	21	64	24.5	18.5	20	14	23	14
4448	27	19.5	32.5	24	18.5	20	9	15	40
4449	24	21	18.5	28	18	21	9	26	30
4450	26	19	20	30	17	25	9.5	16	19
4451	29	16	21	35	26.5	22	47	16	25
4452	30.5	18	23	45.5	26	23	20	21	23
4453	30	38	26	43	22	28	16	33.5	59
4454	43	22	24	69	29	26	60	28	27
4455	33	18	20	48	27	26	20	16	26
4456	34	18	21	38	37	20	12	23	65.5
4457	43	20	25.5	35	463	23	44	38	24
4458	40	17	23	35.5	25	24	12	22.5	20
4459	20	15	28	22	22	17	11.5	19	17
4460	720	286.5	281	190.5	425	430	309	266	375
4461	19	12	40	29	22	14.5	8	15.5	21
4462	26	17	49	35	23	21	9	15	20
4463	19	13	13	25	15	17	6	11	25

4464	24	15.5	26	30	21.5	22	10	15.5	22
4465	20	13	22	42	21	15.5	9	16	17
4466	21	12	22	35.5	17	15	10	14	21
4467	18	24	15.5	44	18	15	8	17	22.5
4468	22	12.5	35	43	20.5	13	8	14	16
4469	18	10	26	30	21	14	9	16	10
4470	25	12	19	35	21	17.5	9	13	14
4471	19	11	23	38.5	21	14	8	15	20
4472	16	13	21	26	20	14	7	13	13
4473	18	14	18	28	17	18	7	13.5	12.5
4474	17	10	16	25	19	12	7	12	40
4475	18	15	19	34	28	17	9	14	19
4476	20	12	12.5	34	14	14	6	12	26
4477	28	13	33	50	18	16	10	28	26
4478	434	16	19	20	31	22.5	11.5	109.5	22
4479	25	14	15	21	20	17	11	106.5	14
4480	24	11	9	25	18	13	8	15	20
4481	50.5	13	13	37	27	16	10	13	11
4482	21	11	9	16	16	13	11	79	8
4483	13	15	10	16	12	16	6	13	10
4484	72	57	32	64	35.5	59	25	94	61.5
4485	60.5	42	19	44	40	44	14	63.5	33
4486	36	29	17	36	26	35	13	65	23
4487	136	136	63.5	102	57	122	52	148	116
4488	72.5	61.5	34	66	39	63	34	126	127
4489	47	32.5	24	42	29	37	11	70	28
4490	27	24	11	32.5	28	29	11	50	11.5
4491	166	140	75	119	67	144	61	178.5	120
4492	107.5	106	56	109	47	106	48	143	81
4493	43	37	22	54	24	40	14	68	74
4494	41	41	15	42	22	44	13	48.5	35
4495	134	123	67	109	57	128	55	141	100
4496	292.5	266	123	178.5	98	253.5	93	292	282
4497	46	36.5	26.5	45	22	41	18	62.5	65
4498	34	25	21	34	18	24	16	46.5	28.5
4499	89	87	48	91.5	44	84	46	158	70
4500	25	18	12	37	15	25	9	37.5	9
4501	89	89	42	81.5	44	91	36	111	84
4502	70.5	65	35	60	44.5	67	27	100	97
4503	25	22	16	35	22	22	17	79.5	28
4504	108	101	52	89.5	43	104	41	129	96
4505	54	49	22	39	22	49	31	57	48
4506	101.5	88.5	52	88	49	92	44	160	88
4507	90	82	43	80	42	85.5	37	94	125
4508	72	64	33	69	39	67	29	75	56
4509	49	40	20	38	20	43	17	53	23
4510	161	148.5	83	113	69	152.5	70	143	118

4511	81	70.5	32.5	53.5	32	67	27	68.5	57
4512	24	20	10	29	14	23	10	33	12
4513	29	20	17	34	15	20	11	40	22
4514	28	25	19	37	32	29	12.5	98.5	11
4515	45	32	16.5	53	26	36	11	49	36
4516	85	75	42.5	71	51	71	34	112	118
4517	29	25.5	14	35	18	27	15	53	29
4518	110	99	56.5	83	49	100	46	108	73
4519	40	23	14	35	20	23.5	11.5	81	37
4520	46.5	45	31	56.5	26.5	44	23	75	40
4521	93.5	68	40	64	48	70	37	87	59
4522	30	18	13	32.5	15	20	9	40.5	32
4523	16	12	10	24	15	14	8	28.5	11
4524	25	14	9	23	12	16	7	22	20.5
4525	41	13	9	16	12.5	16	10	26	5
4526	24	17	9	23	12	17	8	27	13
4527	23	18	11	41.5	16	19	8	43	34
4528	13	12	9	19	15	15	7	29	3
4529	24	15	10	32	16	17	9	42	5
4530	23	17	11	22	20	18	11	55	64
4531	21	17	9	17	12	19	8	31	14
4532	25	17	14	41	16	15	25	51	19
4533	43	23	18	33	65.5	27	22	173	20
4534	40	16	10.5	14	13	20	11	17	13
4535	15	14	14	16	15.5	14	9	62	3
4536	42	23	18.5	43	26	22	17.5	121.5	20
4537	25	12	9	20	14	12	8	27	13
4538	17	10	9	33	20	13	42	27	40
4539	56	27.5	16	44	207	20	65	59	21
4540	26	19	13	32	29	16	9	60	24.5
4541	12	14	8	12	8	15	6	15.5	3
4542	21	16	9	27	15	16.5	12	32	4
4543	16	14	9	18.5	13	15	7	18	3
4544	22	16.5	14	44	12	18	9	30	7
4545	14.5	12	9	18	13	12	9	25	16
4546	21	17	19	47	29.5	15	11	53	58
4547	76	16	15	23	21	14	12	55	6.5
4548	47	17.5	13	25.5	18	15.5	14	68	14
4549	22	17	25	21	44	19	11	39	7
4550	28	17.5	11	21	24.5	17.5	10	33.5	8
4551	29	14	35	24	20	14	12	21	6
4552	34	17	16	25.5	28	20	11	64	7
4553	27.5	16	9	18	10	17	12	21	22
4554	61	38	14	28	17	33	13	32	60.5
4555	22	11	6	32	14	12	7	21.5	7
4556	20	12	8	38.5	23	13	13	21	7
4557	14	12	10	18	13	12	8	34	3

4558	16	13	9	16	13	15	11	19.5	3
4559	36	40	30	50	31.5	44	15	220	16
4560	48.5	27	37.5	41.5	83	27	29	54.5	33.5
4561	38	24	73.5	29	33	22	15	34	25
4562	25	22	33	66.5	40	23	14	21	32
4563	26	19	34	43	28	23	16	19.5	22.5
4564	48	29	60.5	58.5	33	25	19	125	19.5
4565	41	11	10	27	25	13	10	25	7
4566	56	19	137	35	44	26	22	38	31
4567	56	23	78	30	35	27	34	76	24
4568	22	16	43.5	23	19	19	12	15.5	17
4569	30	17	37.5	28	32	20	27.5	18	14
4570	17	16	20	36	22	18	9	13	11
4571	27	15	17	30.5	31	18	11.5	54	12
4572	34	12	10	25	38	13	7	14	6
4573	22.5	16	34	37	95	21	22.5	38.5	14
4574	35	14	15	33	25	14	12	80	15
4575	29	14	9	14	21	10	12	19	10
4576	37	22	41	24	37.5	26	16.5	24	17.5
4577	42	21	99.5	45	98.5	26	23	82	19.5
4578	49	27	264	45	52	31	31	78	29
4579	700.5	23	194	47	56	30	40	31	19
4580	720	38	22	42	80	26	56	104.5	37
4581	93	20	14	32	30.5	15	18	72	20
4582	203	114	392	312.5	126.5	105	56	67	160
4583	194	50.5	64.5	176	115	51	25	44.5	104
4584	74	30	82	128	47	31	32	58	48
4585	9	11	10	19	11	13	5	10	7
4586	28	20	19	19	27	29	11	16	16
4587	14	12	10	19	11.5	13.5	7	20	5
4588	32	41	39.5	53.5	30	32	27	56	45
4589	25	35	38.5	45.5	25	27	25	33	32
4590	41	87	72	79	52.5	50	44	74	103
4591	24	20	37.5	47	23	19	14	26	48
4592	33	46	38.5	53	27	28	22	44	41
4593	48.5	74	42	70	56	44	35	62.5	78
4594	39	60	67	64	35	42	32.5	55	62
4595	47	86	78	88	58	47.5	47	83.5	84
4596	53	96.5	79	98	50	58	43	87	86.5
4597	36.5	78	48	83	32	48	34	66.5	70
4598	41	71.5	59	71	36	43	37	69.5	68
4599	50	64	50	73.5	44	41	30	68	72
4600	60	71	64.5	72	39	54	33	58	68
4601	51	73	57.5	72	35	61	28	58	66
4602	28	41	42	66	34	31	24	55	56
4603	43	82	67.5	74	45	47	41	74	86
4604	46	62	56.5	66	31	42	31	56.5	68

4605	32	21	82	49	34	22	20	29	151
4606	27	13	48	25	56	13.5	13	22	21
4607	25	12	78.5	30	27	14	13	28	14
4608	37.5	24	27.5	33	59	33	12	21	27
4609	22	10	27.5	29	25.5	12	10	22	18
4610	24	17	17	30	25	14.5	10	20	30
4611	21	12	22	32	34	16	11	13	12
4612	15.5	11	34	30	25.5	12	9	15	11
4613	31	16	31	36	33	16	13	22	22
4614	20	14	25	42	28	16	9	18	22
4615	14	9	18	16	18	9.5	8	9	9.5
4616	21	14	23	24	18	18	7.5	14.5	12
4617	26	13	42	31	18	14	9	28	16
4618	47	15	72	27	19	18	12	28	13
4619	12	10	12	14	15	11	7	10	7
4620	357.5	28	30	89	31.5	39	22	23.5	35
4621	57	22	11	34	58	24	9	63	27
4622	52	14	10	38	31	15	10	27	31.5
4623	37	20	24	27	309	22	11	28.5	17
4624	29	21	10	22	18	23	9	64.5	17
4625	108	27	14	52	34	31	21	34.5	20
4626	36	18	31	31	33	20	17	30.5	20
4627	85	18	20	27.5	22	22	45	26.5	26
4628	57.5	29	27	23	259.5	30	10	24.5	14.5
4629	132	105	49.5	89	61	106	45	93	109
4630	131	100	50	87	46	102	39.5	88.5	85
4631	135	109	57	81	50	109.5	44	108	113
4632	194	172	88	107	71.5	172	67	166	166
4633	118	102	54	77	66	104	47	99	102
4634	154	129.5	58.5	83	55	134	47.5	117.5	119
4635	41	28	17	28	26	32	14.5	33	22
4636	146	124	52	82	51	124	45	110	113
4637	117	96	42	85	45	95	40.5	84	81
4638	119	109	49	79	48	109	42	104	96
4639	164	144.5	68	103	61	146.5	56	129	151
4640	100	84	41	64	35	85	32	68.5	81
4641	159	139	63	95	72	138	66	118.5	157
4642	75	57	27	94	41	57	24.5	55	68
4643	55	43	21	111.5	18	53	14	30.5	38.5
4644	108	101	56	88	57	100	57	116.5	109
4645	34	37	18.5	113	15	48	8	31.5	36.5
4646	368	351	175	247	155.5	390	156	304.5	368
4647	49	34.5	18	74	21	47	8	29	42
4648	20	11	7	11	9	13	5	13	8
4649	15	9	6	11	13	13	5	9.5	2
4650	19	11	6	11	8	12	6	11	7
4651	25	14	10	17.5	9	15	9	12.5	9

4652	16	9	5	11	12	11	4	12.5	7
4653	15	9	4.5	15	8	11	4	12	3
4654	17	8	6	11	9	10	5	10	3
4655	27	13	18	13	29	13	6	14	20
4656	19	10	5	11.5	136	14.5	4	11.5	2
4657	48	9	7	8	10	12	5	11	4
4658	247.5	123	19.5	31	46	341.5	12.5	26	61
4659	19	13	7	16	9	13	6	15	27
4660	25	9	7	15.5	14	10	7	29	17
4661	21	10	6	12	7	11	5	10	16
4662	27	13	7	16	10	16	6	12.5	6
4663	21.5	10	7	12	10	11	6	19	23
4664	20	10	7	11	9	13	7	16	9
4665	16	10	8	15	9	11	4	13	9
4666	16	7	5	16	7	8	5	13	10
4667	38	10	9	14	19	11	13	41	32
4668	248	116	59	248	179	105	693	871.5	69
4669	70	20	71	29	35	21	20	26	21.5
4670	24	10	6	20.5	15	10	6	16	12
4671	24	12	6	27	14	14	7	14	6
4672	32.5	23	8	33	43	31	11	22	6
4673	40.5	28	16	42	25	31	11	20.5	37
4674	96	73.5	37	101.5	53.5	85	23	81	57
4675	118.5	87.5	52	83.5	57	99	45	82	80
4676	224	192	111	150.5	95	202	99	176	188.5
4677	77.5	54	22	56.5	33.5	56	19	62	39
4678	37.5	25	12	35	28	30	10	22	28
4679	89	79	33	76	34	75	30	65	79
4680	33	17	7	33	13	22	6	18	20
4681	68	53	29	50	33	58	26	46	57
4682	72.5	57	24	48	34	58	17.5	47	39
4683	39.5	24	9.5	42.5	11	32	8	22.5	10
4684	150	31	12	46	30	37	10	26	18
4685	74	36	39	45	23	44	13	35.5	33
4686	143.5	143	64	91	63	133	52.5	128	124.5
4687	141	106	52	95	48	112	50.5	88	99
4688	29	18	13	30	35.5	20	6	18	36
4689	57	32	17	50.5	27	42	11	28	57
4690	42	21	11	40	17	26	8	18	11
4691	37.5	22	8	34	17	25	6	17	18
4692	98.5	60	33	63.5	37	64	24.5	54	67
4693	129.5	111	53.5	94.5	54	118	52	103	108
4694	74	67	32	61	25	69	24	57	54
4695	31	16	8	34	14	21	10	21	19
4696	23	13	11.5	28	20	19	9	15.5	22
4697	33.5	19	9	29	17	23	8	15	24
4698	60	39	18	45	27.5	39.5	15.5	34	33

4699	65	47.5	33	44	30	48.5	22.5	39.5	38
4700	59.5	47	26	56	25	52	20	45	55.5
4701	95	81	40.5	68	30.5	89.5	34	69.5	65
4702	129	101.5	41	86	44	102.5	86	79.5	75
4703	33.5	18	12	27	16	23	9	13	31
4704	80	19	13	37	54	23	9	21	18
4705	112	100	49.5	72	48	100	40	80	90
4706	136	91.5	44	75	40.5	90	39	82	87
4707	155	112.5	51	81	49	108	41	97	147
4708	47	35	16	50	24	44	13	38	40
4709	46	29	15	43	18	34	12	30	46
4710	43	20	10	35	20	24.5	9	20	17
4711	139	60	34	88.5	50	53.5	17	48	47.5
4712	70	45	20	59	33	50	16	45	38
4713	92	78	37	69	32	81	23	68	68
4714	60	52	31	56	23	59	48	53	57.5
4715	88	68.5	35	62.5	30	74	29	61	57.5
4716	65	56.5	30	55	27	57	19	46	52
4717	139	101.5	50.5	90	63	106	47	91	85
4718	75	67	29	55	30.5	71	22	65	60
4719	67	30.5	16	35	21.5	35	11	28	22
4720	88.5	58	26.5	68	30	63	24	56.5	60
4721	77	39	21	51	24	43	18	37.5	53
4722	132	120	50.5	85.5	46	126	46	207.5	106
4723	36	21	9	34	12	23	8	17.5	15
4724	148.5	65.5	34.5	63	45	68.5	26	58	81
4725	91	74	35	62	36.5	76	35	68.5	74
4726	125.5	103.5	53	100	54	104	48	97	159.5
4727	44	26	14	36	17.5	35	13	24	33
4728	110	100	46.5	80.5	43	96	33	101	91
4729	31	16	10	42	17	20	11	18	41
4730	40	19	10	34	22	23	9	20	34
4731	26	15	12	32	52	19	6	15.5	19
4732	52	44	20	58.5	21	47	16	43.5	47
4733	16	9.5	4	6	5	12	3	7	1
4734	21	10	5.5	19	35	14	5	11	3
4735	26	11.5	7	26	22	15	5	13	5.5
4736	24	13	6	23	11	15	8.5	12.5	4
4737	21	12	6	33	11	16	6	10	4
4738	21	14	6	30	17	16	6	13	8
4739	60	19	11	60	36.5	20	25	136	12
4740	18	11	6	20	48	15	6	10	11
4741	24	12	5.5	25	11	16	5	11.5	6
4742	27.5	11	7	21	13	13	4	9	14
4743	17	11	5	20	9	14	5	10	7
4744	23	11.5	7	27	16	18	6	13	8
4745	29	13	9	29	18	16	12	22	81

4746	22	19	7	20	14	30.5	7	106	9
4747	17	10	5	10	9	15	4	7	3
4748	20	59	34	13	11	15	18	11	4
4749	19	10	5	16	11	13	4	9	3
4750	21	13	48	16	10	13	5	10	3
4751	17	11	5	13	6	13	6	8	5
4752	21.5	14	43.5	22	10	15	6	13	4
4753	20	12	5	16	9	20	4.5	18	8
4754	68	17	11	21	19	24	8	15	6
4755	29	17	9	22	27	23	11	36	7
4756	29	36	307	22	16	20	16	21	5
4757	31	15	8	23.5	20	20	9	31	58
4758	87	58.5	18	55	35	64	14	55	54
4759	91	70	31	58	34.5	81	28	67	59.5
4760	58	34.5	11.5	44	26	38	12	28	20
4761	46	26	7.5	42.5	20	35	8	43	31
4762	40	25	8	36	25	31	7	33	13
4763	71	47	16	48	43.5	48	15	58	33
4764	75	43	15	51	38	49	15	69	70
4765	65	43	15	60	25	43	15	46	43
4766	123	103	40	74	70.5	109	31	99	92
4767	66	45	22	43	39	47	14	44	49.5
4768	169	125	55	98	66	133	49.5	113	110
4769	80	64	30.5	65	30	75	20	66	80
4770	91.5	68	22	54	33	68.5	20	52	50
4771	67	41	14	50	24	51	14	56	54
4772	54	33	13	49	28	39	13	41	48
4773	95	76	27	66	29	69	22	65	52
4774	25	15	7	34	46	21	10	27	32
4775	32	14	6	24	11	19	6	24	9
4776	26	18	7	41	20	25	7	42.5	12
4777	37	18	9	21	11	20.5	9	12	6
4778	106	20	7	32	20	27	6	27	7
4779	15	8	6	14	6	9	15	8.5	3
4780	58	13	29.5	36	12	16	9	18	4
4781	42	23	10	25	23	26	12	23	26
4782	34	14	9	31	25	20	7	18	5
4783	31	16	9	39.5	22	22	9	30	44
4784	40	16.5	20	38	323	23	19	31	21
4785	35	15	9	36	18	18	13	23	34
4786	50	21	12.5	42	376.5	33	14	122	71.5
4787	45	16.5	12.5	17	14	20	10	20	23
4788	15	10	4	6	5	14	3	7	1
4789	18	13	8	15	6.5	17	3	12	2
4790	105	80.5	31	69	30	91	24	64	76
4791	167	110.5	49	83	33	104	34	92.5	86
4792	79	39	16.5	42	20	41	16	36	52

4793	62	42.5	31	46	25	44	20	39	42
4794	101	75	38	71	39	75	38	74	68.5
4795	103	71.5	27	55	35	78	23	57.5	56
4796	59	38	15	40	23	44.5	15	41	46
4797	68	54	24	63	23	60	27	50	61
4798	94	80	39	70	30	84	31	71	68
4799	84	53	25	47	24	59	19	54	46
4800	41	27	14	38	16	32	13	28	62
4801	77	58	30	55	52.5	63	27	53	51
4802	47.5	32	14	41	25	33.5	19	29.5	33
4803	88	67	35	68	36	76	27	65	64
4804	99.5	69.5	31	56	29	73	26.5	56	56
4805	48	37	16	37	15	41	16.5	32	30
4806	47	32	15	35	15	35.5	13	29	34
4807	51	40	21	41	22	40	15	35	45
4808	71	45	22.5	52.5	25	52	19	41	41.5
4809	64.5	48	21	53	24	53	18	45	47
4810	89	66.5	34	60	52	72	27	58.5	107
4811	52	39	16	39	16	36.5	15	38	38
4812	19	12	82	14	8	18	7	11	5
4813	77	56	25.5	50	29	61.5	19.5	51	46
4814	160.5	71	37	61.5	46.5	72.5	29	73	63
4815	61	45	22	47	19	46	19	41	37
4816	82	59	27	54	24	56	24	52.5	61
4817	86	62	27	56	27	66	22	56	67
4818	97.5	80	34	57	38	77	25	67.5	75
4819	49	40	17	33	20	44	12	32	40
4820	52	41.5	18	42	19	41	13	36	76
4821	66	51	25	48	23	59.5	19.5	50	44.5
4822	63	42.5	21	52	63	49	20.5	41	35
4823	72	60	24	55	29	61	21	45	66
4824	90	65	34	62	43	70	27	63	66
4825	86.5	40	16	45	27.5	40.5	16	40	32
4826	54	27	13	35	13	28	11	24	31
4827	38	19	18	32	17	22	10	19	27
4828	64	47	25	58	27	57	18	43	44
4829	47.5	25	14	47	15	31	12	26	41
4830	84	57	27	58	30	64	22.5	53	53
4831	43	25	13	34	21	32	11	24	33
4832	152	121	60	117	67	132	58.5	105.5	119
4833	152	121	60	117	67	132	58.5	105.5	119
4834	48	42	17.5	38	20	44	17	35	43
4835	48	42	17.5	38	20	44	17	35	43
4836	51	44	20	50.5	26	47.5	15	36	30
4837	51	44	20	50.5	26	47.5	15	36	30
4838	47	35.5	15	44	21	39	40.5	37	24
4839	47	35.5	15	44	21	39	40.5	37	24

4840	68	47	24	47	41	51.5	21	48.5	66
4841	68	47	24	47	41	51.5	21	48.5	66
4842	34.5	23	14	31	17	28	11	23.5	21
4843	34.5	23	14	31	17	28	11	23.5	21
4844	72	55	25	57.5	34	56	22	54	46
4845	72	55	25	57.5	34	56	22	54	46
4846	65	56	27	51	36	63	22	126	51
4847	65	56	27	51	36	63	22	126	51
4848	122	56	26.5	60	30	58	24.5	53	44
4849	122	56	26.5	60	30	58	24.5	53	44
4850	76	57	74	56.5	32	67	30	61.5	56
4851	76	57	74	56.5	32	67	30	61.5	56
4852	70	49	27	58	44	56	24.5	57	43.5
4853	70	49	27	58	44	56	24.5	57	43.5
4854	70	59	26	66	36	61	21	56	63
4855	70	59	26	66	36	61	21	56	63
4856	85	69.5	38	70	80	76	31	69.5	65
4857	85	69.5	38	70	80	76	31	69.5	65
4858	76	49	27	53	34	57	23.5	46	50
4859	76	49	27	53	34	57	23.5	46	50
4860	76	70	36	59	39	71	25	65	72
4861	76	70	36	59	39	71	25	65	72
4862	85.5	50	23	46	29	56	20	45	34.5
4863	85.5	50	23	46	29	56	20	45	34.5
4864	137	53	31	71	30	55	20	55	53.5
4865	137	53	31	71	30	55	20	55	53.5
4866	86	65.5	30	60.5	27.5	67	24	71	120
4867	86	65.5	30	60.5	27.5	67	24	71	120
4868	87	71.5	35.5	59	33	81	32	68.5	59
4869	87	71.5	35.5	59	33	81	32	68.5	59
4870	41.5	21	13	40	32	24	9	21	29.5
4871	41.5	21	13	40	32	24	9	21	29.5
4872	72	60	28	52	33	70	25	55	59
4873	72	60	28	52	33	70	25	55	59
4874	78	64	32	59	31	71	25	60	76
4875	78	64	32	59	31	71	25	60	76
4876	60	40	18	50	27	43.5	17	36	43
4877	60	40	18	50	27	43.5	17	36	43
4878	60	32	19	39.5	26	35	17	31.5	26
4879	60	32	19	39.5	26	35	17	31.5	26
4880	34	18	8	31	15	18	8	16.5	14
4881	34	18	8	31	15	18	8	16.5	14
4882	31.5	17	14	34	12	18	8	14	41
4883	31.5	17	14	34	12	18	8	14	41
4884	78	68	33	60.5	34	73.5	25	68	61
4885	78	68	33	60.5	34	73.5	25	68	61
4886	110	36.5	22	52	29	46	23	37.5	58

4887	110	36.5	22	52	29	46	23	37.5	58
4888	41	29	12	39	20	29	9	29	23
4889	41	29	12	39	20	29	9	29	23
4890	89	78	32.5	59	36	79.5	28	66	76
4891	89	78	32.5	59	36	79.5	28	66	76
4892	78	59	29	61	36	64	24	58.5	52
4893	78	59	29	61	36	64	24	58.5	52
4894	67	49	24	52	27	53	17	44.5	54
4895	67	49	24	52	27	53	17	44.5	54
4896	101.5	80	36	59.5	30.5	82	29	70	76
4897	101.5	80	36	59.5	30.5	82	29	70	76
4898	57	40.5	20	48	20	47	18.5	57	45
4899	57	40.5	20	48	20	47	18.5	57	45
4900	143	115	51.5	78	46	113.5	47	99	106
4901	143	115	51.5	78	46	113.5	47	99	106
4902	75	60	25	52	27	59	23	49	52
4903	75	60	25	52	27	59	23	49	52
4904	62	43	24	53	24	54	18.5	47	53
4905	62	43	24	53	24	54	18.5	47	53
4906	160.5	135.5	63	105	66	139	51	110.5	149
4907	160.5	135.5	63	105	66	139	51	110.5	149
4908	74	61	24	54	26	64	22	50	51
4909	74	61	24	54	26	64	22	50	51
4910	83.5	60	29	72	39	60	24	61.5	57.5
4911	83.5	60	29	72	39	60	24	61.5	57.5
4912	57	44	18	49	28.5	48	17	40	47.5
4913	57	44	18	49	28.5	48	17	40	47.5
4914	95	75	34	89	31	75	25	67	68
4915	95	75	34	89	31	75	25	67	68
4916	83.5	72.5	39	75	40	73.5	33	68	68
4917	83.5	72.5	39	75	40	73.5	33	68	68
4918	76	72	36	62	38	69	28	71	60
4919	76	72	36	62	38	69	28	71	60
4920	16	12	5	28	9	15	5	13.5	2
4921	16	12	5	28	9	15	5	13.5	2
4922	27.5	14	7	26	9	15	4	15	3
4923	27.5	14	7	26	9	15	4	15	3
4924	20	12	6	40	12	16	6	14	4
4925	20	12	6	40	12	16	6	14	4
4926	321.5	13	5	14	13	18	7	25	6
4927	321.5	13	5	14	13	18	7	25	6
4928	21	14	6	33	14	19	8	15	4
4929	21	14	6	33	14	19	8	15	4
4930	19	12.5	5	19	16	17	4	12	3
4931	19	12.5	5	19	16	17	4	12	3
4932	48	14.5	8	13	14	19	11	11	2.5
4933	48	14.5	8	13	14	19	11	11	2.5

4934	46	11	10	17	27.5	12	6	12	6
4935	46	11	10	17	27.5	12	6	12	6
4936	21	11	8	15	47	14	8	15	4
4937	21	11	8	15	47	14	8	15	4
4938	22	13	8	17	39	16	6	12	4
4939	22	13	8	17	39	16	6	12	4
4940	30.5	12	8	26	12	15	8	17	4
4941	30.5	12	8	26	12	15	8	17	4
4942	34	14.5	10	16	15.5	18	10	13	9
4943	34	14.5	10	16	15.5	18	10	13	9
4944	23	13	9	16	12	17	5	13	3
4945	23	13	9	16	12	17	5	13	3
4946	95	11.5	8	16	12	14	5	12	4
4947	95	11.5	8	16	12	14	5	12	4
4948	30.5	14	11	38	16.5	15.5	9	20.5	74
4949	30.5	14	11	38	16.5	15.5	9	20.5	74
4950	32	13	8	24	15	15	12	11	7
4951	32	13	8	24	15	15	12	11	7
4952	35	17	14	32	22	16.5	23	38	12
4953	35	17	14	32	22	16.5	23	38	12
4954	29	15	12	23	16	16	6	12	27
4955	29	15	12	23	16	16	6	12	27
4956	50.5	12	7	43	11	17	7	21	29
4957	50.5	12	7	43	11	17	7	21	29
4958	37	19	11	50.5	25	27	18	34	124
4959	37	19	11	50.5	25	27	18	34	124
4960	44	26.5	9	36	18	31	7	19	29.5
4961	84.5	64	24	53	28	67	19	50	46
4962	36	21.5	10.5	29	15	27	7	19	25
4963	87	57	25	64	23	67	28	53.5	51
4964	189	64	26.5	71	30	69	20	53	48
4965	108	20	9	34	16	25	7	16	13
4966	45	22	8.5	29	14	27	7	15	19
4967	52	28.5	12	39	14	31.5	7	19	26
4968	54	29	13	33	16	38	8.5	25	31
4969	66	36	19	43	27	41	20	30	26
4970	46	25	12.5	33	15	29	13	21	38
4971	74	41.5	18	38	21	48	12	38	33
4972	120.5	76.5	38	66	37	86	29	75	65
4973	41	25	8	37	267	35	9	21	25
4974	59	30	19.5	36	48	34	12	25	30
4975	43.5	23	14.5	39	12	32	7	23	10
4976	113.5	87	40	70	34	92	31	75	75
4977	83	59	30	55.5	26	67	26	55	54
4978	57	27.5	18	35	21.5	34	14	24	84.5
4979	77	40.5	17	45	21	40	12	32	33
4980	44	21	12	33	21	28	43	28.5	51

4981	101	69	29	59.5	45.5	76	22	59	75
4982	87	39	20	50	21	46.5	15	35	74
4983	59	41	33	40	19.5	49	19	31	34
4984	90	51	27	50	30	60	20	49	57
4985	59	39.5	16	40	18	43	12	33	32
4986	45	24	12.5	40	37	30	13	23	51
4987	75	47	19	47	18.5	56	16	39	40
4988	90	58	28.5	63	37	66	26	51	47.5
4989	48	33	12	42	14	38	10.5	24	32
4990	62	39	23	53	23	44	16	38	56
4991	79	53	28	61.5	32.5	57	25	50	49
4992	102	64	30	67	28	71	22	55	59
4993	162	92	39	66	42	107	34.5	78	72
4994	76.5	70	28	46	28	59	24	44	117
4995	85	64	27	55	29	63	24	50	72
4996	79.5	58.5	23.5	66.5	35	58	20	48	40
4997	93.5	47	16	45	91	51	16	38	55
4998	42	22	11	25.5	14	28	12	18	17
4999	56	33	18	36.5	23	37	13	27	57
5000	77	54.5	23	54	26	62	20	47.5	53
5001	72	43	20	71	24	51	20	42	45
5002	100	72	35.5	79.5	35	83	38.5	71.5	71
5003	87	57	28	50.5	26	64	23	46.5	40
5004	25.5	15	7	14	9	20	5	10	4
5005	87	43	13	78.5	15	53	16	77	62
5006	27	15	6	25	11	21	6	12	4.5
5007	25	16	5	29	8	21	5	11	7
5008	32	17	9.5	15	11	21	19	11	24
5009	41	21	9	84	38	28	62	33	19.5
5010	35	16	10	21	8	21	6	11	5
5011	50.5	18	9	54.5	12	26	10	29	24
5012	25	16.5	6	30	10	20	5	10	7.5
5013	43	18.5	11	88	13	28	9	59.5	8
5014	44	23	12	36	17.5	32	14	45	53
5015	31	21	15	16	54	36	11	14	14
5016	47.5	19	13	16	47	24	26	14	11
5017	28	17.5	7	23	8	22	5	13	10
5018	57	13	9	17	7	19	8	11	4
5019	33	17	7	28	7	21	7	13	12
5020	33.5	16	8.5	37.5	12	22	9	12	13
5021	56.5	22	16.5	64	15.5	30	17.5	90	38
5022	41.5	15	7	28	12	19	6	15	4
5023	23	16	22	16	14	20	7	11	9
5024	401	19	16	35	39	22	14	13	6
5025	46.5	16.5	8	18	8	21	7	13	46
5026	711	388	99	201.5	101	343	117	249	307
5027	69	23	20	64	28	31	32	56	26

5028	21	13	6	17	8	16	5	8	8.5
5029	27	18	6	23	31	23	6.5	11.5	4.5
5030	31	16.5	6	10	10	21.5	6	9	3
5031	29	15	6	15.5	8	18	4	13	2
5032	30	13.5	6	13	12	18	17	10	11
5033	26	20	5	17	24	25	5	9.5	4
5034	42	13	10	21	14	15	8	18	5
5035	36.5	13	10	33	12.5	19	14	19	16
5036	74.5	25	15	101	38	28	14	48	40
5037	372	107	18	64	190.5	318	22	43	73
5038	30	17	9	15	23	21	19	14	9
5039	108.5	13	11	31	37	15	9	14	37
5040	38.5	14.5	9	44	40	19	12	15	19
5041	51	15	11	20	37	20	22	15	15
5042	19.5	11	6	19	8	14	5	12	5
5043	26	11.5	7	37.5	12	17	6	20	25
5044	25.5	13	7	31	11	16	7	15	24
5045	27.5	15	13	20.5	11	19	21	25	10
5046	23	13	15	27.5	9	20	11	15	4
5047	15.5	9	5	8	5	13	4	7.5	1
5048	24	12.5	8	14	7	13	14	12	11
5049	235.5	13	10	18	19.5	17	11.5	13	12
5050	361	15	9	14	14	20	5	10	8.5
5051	454.5	11	7	11	13.5	14	6	10	7
5052	27	16	35	27	24	23	9	34	8
5053	51	15	9	25	9	18	17	11	7
5054	26	14	7.5	18	9	16	14.5	14	5
5055	20	12	7	12	18	17	14	12	7
5056	60	17	10.5	44	67	22	14	18	19
5057	41.5	15	10	38	15	18	10	25	38
5058	17	12	51	7	14	14	4	9	1
5059	25	18	101	21	7	20	6	15	3
5060	56	37	24	339	34	55	16	35	47
5061	63.5	37.5	27	333	31	66	13	41.5	68
5062	71	46	25.5	428	43	58	12	41	63.5
5063	131	57	25	336	42	75	17	46	86.5
5064	52	16	10	37	13	21	8	16	16.5
5065	50	32	19	189	23	49	9	29	59
5066	29	12	6	29	11	18	8	11	23
5067	55	35	21	266	28	57	8	35	53
5068	52	33.5	21.5	40	22	38	19	32	42
5069	68.5	53.5	28	317	38	73	17	42.5	71
5070	72	66	32	352	45	76	21	51	116
5071	30	11	6	24	17	17.5	6	13	16
5072	63	39	22	307.5	33	53	13	38	91.5
5073	72	53.5	31	51	22	54	20	43.5	52
5074	57	43	23	337	32.5	63	13	45	82

5075	66.5	58	21.5	321.5	32	64	16	44	63
5076	62	44	21	46	24	50	18	38	36
5077	74	43	20	317	30	62.5	13	46	68
5078	89	46	37	52	21	48	21	39.5	44
5079	61	45	23	283.5	31	60	15	41	72
5080	48	29.5	17	47	19	34	12	27	34
5081	57	37	24	272.5	36	54	11	34	53
5082	66	46.5	18	48	19	49	14	42.5	47
5083	57	34	21	344	27	55.5	10	34	63
5084	32	19	12	29	17	25	10	18	23
5085	77.5	51	21	262	29	78	12	49.5	68
5086	25	12	7	21	13.5	15	9	10	8
5087	60	43	25	240.5	31	61	13	37	65
5088	38	18	9	29	30.5	20	6	16	26.5
5089	58	42	20	37	19	43	15	34	32
5090	72.5	47	25	305	40	86	14	47	67
5091	54	34	18	34	19	37	13	27.5	25
5092	95	54	24	330	60	66.5	15	50	68
5093	78	49	22	45	48	55.5	20	45	48
5094	63	48.5	23.5	255	38.5	62	16.5	38	74
5095	44	28	15	34	15	32	15	25	27
5096	126	86	41	73	36	85.5	35	75	92
5097	58	32	20	305	25.5	53	9	34	57
5098	83	49	24	51	23	56	18	44	42
5099	124	66	33	333.5	54	91	27	65	76
5100	80	59	32	58	27	63.5	27	54	67
5101	52	34.5	20	220	24	54	10	31.5	47
5102	55	31	14	42.5	18	34	15	26	46
5103	92	46	28	441	35	59	14.5	50.5	63
5104	59	42	22	42	122	45	16	37.5	46
5105	59	33	20	213	42	51.5	9	31	77
5106	39	18	9	23.5	10	19	8	16	23
5107	62	42	22	398	31	58	15.5	41.5	68
5108	104	89	48	69	38.5	92	36	74	89
5109	63	38	20	311.5	48	55.5	12	34	80
5110	53	34	24	276	36	57	9	40	53
5111	89.5	49	34	47	27	53	22	44	43
5112	60	38	23	256.5	34	60	12	32	49
5113	28	10	7	26	8.5	15	7	13	12
5114	87.5	36	20	222	30	99	9	57.5	66
5115	49	31	24	264	43	49	10	38.5	44.5
5116	30	18	9	26	10	20	7	17	12
5117	63.5	35	24.5	300	27	53	11	42.5	54
5118	40.5	24	10	32	20	30	13	24	34
5119	60	36	26	700	37	52	10	57	46
5120	115.5	31	16	41	13	35	14	26	27
5121	72	62	24.5	362.5	46.5	69	22	47	67

5122	136.5	102.5	40	85	56.5	111	41	85	87.5
5123	66	59	26	269	57	62.5	24	47	79
5124	52	31	13.5	48	18	38	12	25	25
5125	69	54	28.5	354.5	43	68	22	45	89
5126	39	19	11	38	13	22	8.5	19	17
5127	84.5	54	25.5	51	22	63	20	48	108
5128	62	41	23	464	35	52	11	39.5	45
5129	49	31	18	300	29	54.5	10	31	40
5130	92	63	30.5	49	24.5	62	24	57	57
5131	68	64	27	294.5	34	68	23	48	66
5132	37	23	14	40	11	25	9	22	20
5133	61	50	22	254	29	56.5	19	43	65
5134	90	62	33	59	29	64	24.5	56	116
5135	61.5	34.5	23.5	288.5	35	53	13	32	58
5136	46	23	14	42	17.5	23.5	13	21	33
5137	111	55	26	289	34	141.5	14	99.5	73
5138	27	15	6.5	28	31	17	7	11	11
5139	77.5	82	30.5	322	59	68	31	56.5	84
5140	21	11	8	23	16	13.5	6	10	13
5141	60	50	20	361	29.5	59	23	41	73.5
5142	28	13.5	7	32.5	8	15	6	11	45.5
5143	57	37	22	343.5	29	57	15	36	66
5144	62	42	21	48	19	48	18.5	36	42
5145	42	28	14	154	15	47	6	27	32
5146	95.5	73	32	61	31	76	33	64	69
5147	48.5	33	14	269.5	23.5	52	9.5	31	36
5148	37	13	7	40	10	17	6	13	36
5149	47.5	30	15	103.5	19.5	46	8	27	36
5150	50	37	16	44	14	40	16	33.5	34
5151	50.5	27.5	17	353	19	48	8	29	32
5152	62	44	26.5	52	21	48	24	42	62.5
5153	53	30	15	166.5	20.5	64.5	7	33	38
5154	38.5	25	12	33	13	27.5	10	24	16
5155	53	31.5	19.5	283	20	56	7.5	36	42
5156	71	59	27	51	24	58	21	53	49
5157	54	25.5	15	299	20	48.5	7	31	35
5158	37.5	20	11	29	13	24	10	19	17
5159	46	26	14	198.5	15	46.5	7	27	31
5160	124	86	36	71.5	35	84	38	68	71
5161	43	25	13	176	16	43	6	24	28.5
5162	73	53	31	56	21	54	25	44.5	44.5
5163	41	24	13	161	14	45	9	25	28
5164	101	74	37	69	31	79	31	70	67
5165	40	25	9	28	15	44	6	24	29
5166	82	56	26	49.5	23	57	23	47	49.5
5167	46	28	16	285	17	47	7	32	35
5168	154	104	49	81.5	121	112	52.5	95	91.5

5169	43	25	10	21	13.5	50	5	24.5	29
5170	105.5	82	42	88	47	82	44	70	66
5171	48	24	10	129.5	15	54	6	26	33
5172	60	29	15	38.5	15	36.5	15	32	24
5173	42	26	15	318	18	46	8	26	27.5
5174	54.5	35	16	48	19	36	15	32	27
5175	39	22	7	18	13	46	5	24	29
5176	44	17	9	68.5	15	22	9	50.5	176
5177	55	25	16	278	17	43	9	27.5	30
5178	25	13	19	35	152	17	8	20	44
5179	41	23	12	171	15	44	7	24.5	29
5180	23	10	5	23	7	14	5	11	5
5181	36	19	8	13.5	12	44	5	21	22
5182	38	26	9	17	16	45.5	5	23	29
5183	335.5	188.5	51.5	107.5	58	168	68	118	169
5184	42	25	12	191	22	46	6	25.5	32
5185	75.5	11	6	26	15.5	15	22	13	17
5186	43	25	12	155	17	49	5	24	29
5187	20	10	5	13	8	16	6	9	3
5188	43	24	12	168	24	49	6	26	30
5189	29	23.5	13	13	8.5	32	5	12	12.5
5190	21	18	9	15	9	24	5	12	12.5
5191	43	22.5	12	154	16.5	47	8	22	26
5192	22	13	7	27	27	16	6	15	26
5193	42	26.5	11	109	22	53	5	25.5	30
5194	55	28	11	44	25.5	30	18	20	25
5195	50	26	12	95	26	43	10	28	28
5196	29	22	9	41	17	28	16	32.5	7
5197	46	31	18	219	37	45	15	35	32
5198	58	20	7	18	12	24	26	14	6
5199	71	43.5	23	373	22	74	11	70	78
5200	69	21	8	40	19	25	8.5	17.5	23
5201	70.5	28	19	347.5	60.5	47	7	34	76.5
5202	30	22	12	17	12	28	6	15	6
5203	76	33	24	330	383	62	9.5	38	165
5204	41	64	36	53	21	25.5	18	24	15
5205	50	27	16	106.5	28.5	50	9	29	30
5206	175.5	70	80.5	172.5	165	67	16	95.5	59
5207	92	29	19.5	235	20	50	9	28	35.5
5208	31	18	8	33	19	20	5	31	22
5209	78.5	29	21	143	25	48	26	27	35
5210	67	51.5	22	60	23	51	19	51	42
5211	50	38	14	46.5	20	40.5	12	42	32
5212	58.5	30	18	100	20.5	56	9	29	53
5213	74	58	20	53	25	64	18	50.5	43
5214	61	31	30	312	46	45	12	33	50
5215	44	23	12	159	16	47	6	24	34

5216	36	27	9	37	31	32	8	39	25
5217	37.5	24	12	84	16	46	5	30	28
5218	25	20	8	30	35	24	7	26.5	15
5219	40	24	11	151	15.5	44	6	21	25.5
5220	71	60	22	63	47.5	58	18	61	49
5221	39.5	24	11	245.5	14	44	5	22	26
5222	38	21	10	43	21.5	31.5	9	38	47
5223	51	27	15	151	19	47	6.5	62	33
5224	66	47	13	48.5	25	50	13	47	34
5225	37	22	13	122.5	32	39	7	22	29
5226	29	20	8	23	11	22	6	16	25.5
5227	47.5	28	21	278	20	47	6	27	33
5228	27	18	6	13	14	21	5	14	15
5229	47	24	16	153	19	50	8	29	35
5230	51	20	6	14	13	19.5	11	15	7
5231	54	28	19	203	24	51	10	31.5	37.5
5232	29	21	17	21	21	23	11	18	14
5233	38	22	10	59	15	43	5	21	27
5234	60	18	8	30	30	26	10	47.5	37
5235	43.5	25	18	553	32	44.5	7	28	33.5
5236	26	20.5	9	29	37.5	26	23	14.5	3
5237	38.5	23	13	79	17	42	6	24	33
5238	26	19	6	21	11	21	6	14	14
5239	50	27.5	18	216.5	23	43.5	13	34	49
5240	83	22	8	24	18.5	26	10	22	10
5241	53.5	28	12	96.5	21	60	9	24.5	36.5
5242	49.5	20.5	12	32	24	30	11	25	12
5243	60	24.5	19	156.5	31	44	13	26.5	34
5244	30	19	8	23.5	12	23	7	17	80
5245	50	26	37	83	47	46	7	25	29
5246	57	44	16	263.5	23	46	9	32	52.5
5247	30	16	25	24	14	15	32	15	24
5248	36	23	10	136	16	45.5	5	24	31
5249	25.5	19	9	26	14	21	6	19	12
5250	45	24.5	17	289	20	45	6	42	32
5251	29	19.5	5.5	18	9	23	4	12	10
5252	41.5	23	12	129	26	45	6	26.5	27
5253	39	22	12	194	18	43	6	23	34
5254	61.5	53	18	61.5	30	54	15	47	56
5255	42	22	10.5	161	15	43	6	23	37
5256	42	26	12	44	25	34	17	30	26
5257	49	25	15	151.5	541	45	9	23	34
5258	57	35.5	20	48	18	37	18	33	37
5259	42	24	20	106	29	46	6	26	29
5260	44	36	14	33	29	38	11	37	30.5
5261	46	29	14	158	18	45	6	25.5	33
5262	40	29	14	32	17	32	13	28	24

5263	42	27.5	13	178.5	27	42	7	26	32
5264	53	34	18	45	24	39	16	32	54
5265	186	25	16	313	48.5	49	18	32	34
5266	42.5	30	18	38	31	35	12	26	37
5267	48	27	18	182	23	45	8	29	28.5
5268	68	57	23	49	36	60	21	51	49
5269	58	28	17.5	211	81	71	8	41	44
5270	59	33	12	47	15	35.5	12	30	24
5271	49.5	26	16	281.5	52.5	44	7	28	31
5272	30	22	8	34	18	24	6	20	19
5273	67	30	22	295	124.5	61	9	32.5	40
5274	65	42	20	45	22	50	14.5	38	42
5275	55.5	30	25	428	24	47	8	42	45.5
5276	36	25	11	41	19	32	10	26	21
5277	45.5	29	23	193	21	45	10	33	36
5278	45	31	13	35	15	35.5	10	24	26
5279	41	26.5	13	150	18	47	7	28.5	30
5280	36	25	11	33	14.5	33	11	23	22
5281	60	25.5	19	208	19	56	7	30	43
5282	49	37	15.5	47	20	43	12.5	36	37
5283	50	35	14	37	20	52	13	38	21
5284	36	21	11	46	23.5	25	77	21	21
5285	51.5	31	16	46	36	33	14	30	30.5
5286	50	37	20	275	24	50	10.5	30.5	57.5
5287	32	22.5	8	37	13	26	8	24	17
5288	46	27.5	18	286	43	44	11	32	74
5289	40	23	14	38	17	30	9	22	24
5290	48	45	17	231.5	30	50	13.5	32.5	59
5291	64	46	14	37	17	42	12	31	34
5292	59	39	20	226	52	51	13	34	53
5293	101.5	73	30	62	30	74	23	65	69
5294	64	49	22	306.5	31	60	18	40	63
5295	48.5	30	14	37	18	32	11	29	42.5
5296	41	26	17	208	24	42	8	27	36.5
5297	101	87.5	46.5	73.5	56	90	35.5	86	94
5298	43	25	15	255	26	45	6	28	34
5299	76.5	56	23	51	28	60	21	52	57
5300	47	24	16	215.5	17	47.5	7	26	37
5301	22	16	7	18	28.5	18	7	16	32.5
5302	59	40	19	278	23	72	12	37	43
5303	513	21	14	43	18	26.5	23	32	18
5304	46	27	19	269	22	42	8	28	49
5305	27	21	15	80	35	26	35	46.5	19
5306	80	41	19	260	26	112	11	51	65.5
5307	15	13	5	7	5	18	4	8	1
5308	53	38	30	289	27	54	18	31.5	52
5309	16	14	5	17	10	18	5	17	7

5310	69	46	31	315	29	56	15	35	50
5311	27	23	11.5	248.5	13	30.5	16.5	65	3
5312	48	36	26.5	257	23	43	10	30	43
5313	45	22	15	198.5	17	43	6	33.5	38
5314	44	26	16	239	15	41.5	9	24	41
5315	59	42	27.5	81	26	48	20	36.5	39
5316	63	35	17	261.5	19	106.5	7	33.5	77
5317	116	98	47.5	67.5	44	97	40	94	112
5318	54.5	31	16	267	23	47	11	28	45
5319	88	46	26	54	40	49	19	49	65.5
5320	45	28.5	17.5	287	54	43	8	38	37
5321	72	48	25	89	35	56	23	60	57
5322	44.5	27	14	235	22	46	9	29	39
5323	139.5	84	42	73.5	48	86	30	66	102
5324	54	41	19	274.5	26	54	16	34	49
5325	107	87	38	84	54	91	39	81	92
5326	43	26	18	231	20	42	6	26	32
5327	53	33	39	276	41	49	18	30.5	38.5
5328	64	38	20	48	24	41	13	41	39
5329	59	54	21	259	27	51	14	35.5	47
5330	134	107.5	49.5	98	69	125	104	103	119
5331	38	23	14	206	15	42	6	22	34
5332	89.5	77	33.5	67	37	84	32	75	59
5333	50	40	19	239.5	29	59	9	33	49
5334	88	66.5	30	70	70	65	43.5	57	73
5335	57	50	21	297.5	27	59	21	42	56
5336	99	71	33.5	73	55	78	28	81.5	70
5337	54	46	21	280	28	58	14	34	57.5
5338	37	20	11.5	33	23	26	9	23	19
5339	52	35	21	212.5	27.5	52	12	32	55
5340	61	43	26	55	33	49	23	40	41
5341	56	39	21	377	29	50	14	34	47
5342	52	31.5	18.5	273.5	21	46	10	31	63
5343	52	39	15	50	20	43	18	31.5	35
5344	42.5	24	15	246.5	19	43	8	24	36
5345	87	67	33	68	34	67	36	57	58
5346	43	27	16	306	24	41.5	9.5	28	35
5347	100.5	75.5	43	75	45	82	36	78.5	76
5348	45	28	17	299	22	46	9	28	57
5349	62	35	19	56	33	42	16	38	32
5350	46	27	14	267	19	48	7	28.5	30
5351	93	67	30.5	60	33	73	24.5	63	81
5352	51.5	28.5	19	256	25	45	8	26	58.5
5353	45.5	27	16	181	17	42	8	27	34
5354	106	54.5	25	63	30	57	22	50.5	66
5355	44	28	16	243	22	44.5	7	29	34
5356	61.5	35	18	44	24	33	16	34	33.5

5357	47	27.5	16.5	255.5	18	44.5	8	28	39
5358	28	14	7	29	16	21	6	18	16
5359	45	34.5	14	318.5	20	48	10	28	42
5360	45.5	32.5	17	280	21	45	11	31	58
5361	106.5	48	24.5	59	37	53	21	52	57
5362	39	27	15	280.5	17	36.5	8	27	32
5363	148	99	39	82	68	95	31	86	93
5364	35.5	22	14	282.5	25	35	6	23	38
5365	403.5	82	23	308	28	607	15	327	127
5366	83	64	34	62	34	72	26.5	89	66
5367	49	25	12	253	15	44	6	26	35
5368	62	58.5	20	254.5	32	58	20	51	54
5369	68	38.5	20	264	26	81	10	35	49
5370	66	42	18	51	24	46.5	14.5	45	40
5371	174.5	33	22	352	27.5	51	11	30	51
5372	89	65	30	61	37.5	70.5	24	59	58
5373	69.5	64	41	325	49.5	78	26	47	72
5374	46	32	18	317.5	19.5	42	8	28	35.5
5375	46.5	17.5	11	33	38	20	12	23	13
5376	63	38	21	401.5	29	67	12	40	64
5377	87	34	14.5	49	24	40.5	10	34	25
5378	55	43	26	381.5	33	46.5	13	35	60
5379	67	26.5	14	42	21	28	11.5	23	13.5
5380	48.5	27	15	232	23	60	6	30	34
5381	111	80.5	36	68	62	83	25.5	79	78
5382	46	27	16	226	26	47	9	27	33
5383	49	31	16	49	18	35	11	27	35
5384	51	35.5	22	329	27	49	10	33	53
5385	71	31	16	48	21	37	15.5	31	40
5386	102	73	57	434.5	258	60.5	14	42	57
5387	84	61.5	39	69	41	69	32	65	88
5388	74	59	24	396	30	80	14	50	64
5389	63	38	19.5	57	37	47	19	45	95
5390	59.5	51	21	292.5	81	62	14	44	52.5
5391	68.5	48.5	20	54.5	28	46	17	42.5	57
5392	61.5	43	25	413	34	54.5	16	35	64
5393	93.5	59	26	54.5	25	59	21	51.5	68
5394	69	49	19	343	26	87	11	43	71
5395	50	30	24	279	22	49.5	9	32	45
5396	33	15	18	53.5	30	22	16	40	10
5397	63	36	22	264	28.5	51.5	9	35	56
5398	34	17	32	53	14	22	11	35	12.5
5399	59	35	30	458	23	52	10	28	45.5
5400	43	19	9	68	16	32	13	75	18
5401	65	29	13	85	16	55	18	262	20
5402	84	83	27	312	53	73	30	52	88
5403	43	28	56.5	57.5	11	48.5	72	149.5	14

5404	52.5	32	20	317	18	48.5	10	31	63
5405	45	19	61	63	13.5	31	20	63	22.5
5406	67.5	35.5	23.5	249.5	26	53	10	42	55
5407	29.5	14.5	13	49.5	86	19	41	21	22
5408	53.5	20	28	68	23	27	21	46.5	37
5409	57	40.5	24	400	28	65	11	39	67
5410	46	18	16.5	42	15	21	12	26	26
5411	59	15	13	55	52	21	26	35	55
5412	48	32	23	309.5	27	46.5	10	33.5	56
5413	49	17.5	18	77	22	20	12	50	103
5414	55	38	23	289.5	32.5	55	13	37.5	65
5415	52	17	14	64.5	138	22.5	11	42	44
5416	70	61	26	320	40	68	22	48	71.5
5417	43	18.5	13	44	16	22	10	32	22
5418	56	43	22	322	22	59	15	35	53
5419	44.5	18	19	58	13	27	15.5	70	71
5420	67	34	19.5	305	25	59	10	38	55
5421	30	14	7	16	13	19	5	12.5	8
5422	67	52.5	26	398	36	65	23.5	51	109
5423	24	11	12	36	8	17	11	13	8
5424	62.5	56.5	30	411	38	63	20	49	73
5425	27	12	7	31	8	17	6	15	21
5426	27	14	7	41	15	19	21	28	28
5427	44	25	19	185	18	46	7	26.5	54
5428	32	19	9	43	16.5	27	6.5	72	34
5429	58	58	21.5	335	29	58	20	42	117
5430	33	16	10	34	30	21	8	26	45
5431	51	39	21	285.5	30	50	12	31	96
5432	33	14.5	16	43	25	18.5	8	25.5	34
5433	54	35	19	164.5	21	48	10	29	77
5434	62	16	13.5	32	19	21.5	62	21.5	74
5435	52	33	19.5	230	20	52	9	29	68.5
5436	41.5	18	14	62.5	38.5	22	15	70	146
5437	57	38	27	367.5	24	49.5	10.5	38	71
5438	62	38	24	164.5	20	60	8	37	60.5
5439	30	16	14	50	26	17.5	38.5	20	33
5440	46	29	20	236.5	16	50	7	27	85
5441	52	21	42	156	477.5	27	90	74	19
5442	56	38	20	294	24	50	12	32	105.5
5443	338	15.5	11	50.5	43	20	16	16	34.5
5444	48	35	17	182	21	48	8	30	85
5445	25	12	6	14	10	14	5	9	6
5446	54.5	29	19	222	22	49	8	35	54
5447	26	11	5	13	10	14	7	9	7
5448	50	36	23	206	342	46	10	30	128.5
5449	21	12	6	11	9	13	4	9	8
5450	57	47	24	249	29	75	15	40	67.5

5451	23	13	6	10	12	15	4	8	4
5452	52	27	17	423.5	21.5	43	8	31	47
5453	19	12	5	10	11	14	7	12	4
5454	56	45	21	261	31	89	12	33	63
5455	17	10	6	13	14	13	5	11	3
5456	58.5	38	20	304.5	30.5	51	10.5	31	48
5457	43	14	49	73.5	24	18	20	27	33
5458	49	40	21	362	28	48.5	13	33	67
5459	32	12	7	56	13	15	18	16	9
5460	37.5	13	12	45	44	17	87	36	21
5461	59	46	22	256	28	64.5	15	37	63
5462	24	11	5	11.5	30	15	9	7	3
5463	107.5	48	21	315.5	27	151	11	74	78.5
5464	82	33	17	72	81.5	41	13	33	52
5465	62	55	23	276	32	59.5	19	42	57
5466	75	17	21	74.5	65	18	33	67	43
5467	64.5	27	18	300	25	39	7	26	33
5468	25.5	11	8	19	17	13	9.5	14	13
5469	49	31	16	229.5	28	42.5	7	27	96
5470	22	8.5	5.5	13	11	11	7	8	3
5471	62	27	17.5	278	22.5	62	9	37	41.5
5472	118	14	11	69	77	15	8	26	30
5473	48.5	29	16	297	39	43	7	29	49
5474	64	28.5	29	45.5	76	33	13	68	117
5475	55	50	32	278.5	29	58	14	37	73
5476	48	37	16	317.5	23	48	11	32	60.5
5477	21	9	5	19	7.5	11	4	9	3
5478	59	45	21	227	32	58	14	33	63
5479	18	11	5	9	8	14	5	9	3
5480	18	8	6.5	37	8	12	19	14	2
5481	74	59	26	269	25	85	11.5	45.5	66
5482	17	9	6	18	14	11	10	13	3
5483	39	24.5	15	290.5	19	43	6	26	28
5484	20	8.5	5	16	10	11	5.5	9	6
5485	52	34.5	19	283	25	44	10	31.5	46
5486	82	27	10	35	35.5	17	28	26	25
5487	53	23	22.5	441	27	43	8	32	41
5488	19	7	6	16	11	10	5	8	6
5489	22	10	7	16	17	11	6	10	5
5490	63	57	25	475.5	39	71	25	45.5	73
5491	83	11	33	24	21.5	14	19.5	16	10
5492	49	36	23	391	30	56	10	33	46
5493	24	9	6	22	10	11	14	11	8
5494	52	42	31.5	230	26	49	16	37	89
5495	32	10	9	19	12.5	11	6	10	10
5496	56	53	23	342	29.5	54	17	40	77
5497	41	12	26	37.5	30	14	9	16	14

5498	41	24	17	395	18.5	40	7	32.5	37
5499	17.5	9	6	17	11	10	6	9	6
5500	72.5	94	33	558	50	74	33.5	61	84.5
5501	37	11	9	25	30	15	11	15	11
5502	26	12	9.5	28.5	15	15	7	16	516
5503	60	53	22	302.5	31.5	61	24	40	68
5504	23	8	6	17	11	10.5	6	9	4
5505	65.5	71.5	27	466	40	66	29	63	87
5506	88	56.5	31	53	32	54	23	53	40
5507	48	36.5	22	338	27	50	11	33.5	42
5508	49	17	11	41	13	22	11	23	12.5
5509	85	69.5	34	59.5	37	74	29	57	84.5
5510	67	47	30	398.5	30	61	15	41	76
5511	56	49	21	361	29	58	15	39	94
5512	409	106	24	48.5	25	107	19	57	47.5
5513	47	35.5	20.5	357.5	26	48	11	34.5	46
5514	66	39	16	39	23	48	36	30	40
5515	57	50.5	22	225	29	61	17	45.5	48
5516	69	46	24.5	62	30	48	21	41	52
5517	55	43	21	597	40	48	12	35	47
5518	82.5	43.5	27	492	60	57	34.5	41	59
5519	45	34.5	16	35	19	34	14	28	36
5520	45	28	18	288	23	43	9	28	51
5521	83	60	28	51	27	55.5	23	57	52.5
5522	47	27.5	19	326	28	44	8	28	53.5
5523	59	29	18	313.5	25	41	13	29	45
5524	87	71	39	65.5	35	69.5	28	68	90.5
5525	53	31.5	21	297	27	52	8	33	40
5526	49	29	19	365.5	28	47	9	31	55
5527	60.5	35	22	320	35	56.5	11	35	61
5528	64	46	23	53	22	49	18	45	46
5529	76	38	31	397.5	37	60	10	40	96
5530	74	56	30	50	26	63	23.5	52	53
5531	73	48.5	32	537.5	41	59	15	46.5	63
5532	35	22	12	34	15	27	9	23	26.5
5533	63.5	39	21	406	26	58	9	35	57.5
5534	48	15	9	33	13.5	15	8	19	11
5535	48.5	27	16	132	21	46	7	30	33
5536	24	10	8	32	10	12.5	11	13	11
5537	43	28	14	97	22	48	8	28	35
5538	48	35	14	46	15.5	35	18	25	47
5539	70	36	23	388	34.5	61	8	40	48
5540	40	13	8.5	33	12	13	6	17	26
5541	68	30	19	256	25	52.5	16	36	45
5542	64.5	36	20	210.5	64	52	10	35	44
5543	44	23.5	14	37	15	27	9	27	19
5544	74	39.5	30	194	49	60.5	13	37	55

5545	52	30.5	19	44	17	36	14	44	61.5
5546	65.5	39	28	344	30	55.5	10	44	56
5547	34	18	12	31	13	20	8	18	14
5548	65	32	19	191	57	58.5	8	37	43
5549	54	28	15	38.5	22	31	14	29.5	27
5550	60.5	33.5	23	494.5	50.5	51	12	51.5	120
5551	40	18	11	29	13	20	10	17	22.5
5552	61.5	29	22.5	263.5	47	57.5	16	33	40
5553	42	26	12	34.5	16	28	10	26	19
5554	94.5	81.5	34	59	33	77	26	63.5	61
5555	76	41	37	807	35	59	14	71.5	91
5556	59	35	18	40	19	38	15	34	53
5557	53	30	22	357	37	47	10	46	52
5558	56	42	20	46.5	20.5	42	31	43	43
5559	57	42	21	534.5	47	57	9	35	49
5560	63	44	27	44	22	51	17	47	47
5561	83	34	24	255	52	51.5	11	35.5	52
5562	67	43	21.5	40	20	47	18	42	42
5563	72	39.5	23	309	55	55	13.5	36	69.5
5564	92.5	59	29	63	35	57	25	54.5	71
5565	51	29	20.5	193.5	22	50.5	9	31.5	41.5
5566	63	39.5	21	47.5	20	42	17	38	40
5567	45	26	17	235.5	26	45	8	30	40.5
5568	85.5	56	31	59	38	62	33	53.5	52
5569	39	15	9	34.5	12	18	8	18	16
5570	31	12	12	39	12	15	6	18	26
5571	28	13.5	7	29	10	14.5	6	17	25
5572	56	39	21	44	19	45	16	39	41
5573	47	33	18	43	20	35	14	37.5	40
5574	41	30	13	33	13.5	33	11	26	43.5
5575	53	33.5	14	50	16	39	14	34	38
5576	46	32	16	39	17.5	31.5	13	31	36
5577	51.5	27	14	39	15	33	12	30	37
5578	50	29	16	43	17	38	14	35	34.5
5579	89	35	15	42	18	37	13	30.5	28
5580	89	66	25	56	30	64	25	55	55.5
5581	93	81	34	70	36	85	31	77	91
5582	99	10	10	25	11	12	9	12	4.5
5583	22.5	10	8	14	14	11	12	18.5	3.5
5584	24	10	7	26	16.5	12	19.5	15	27
5585	23	9	6	21	31	9.5	5	11	7
5586	34.5	9	9	13	12	11	8	10	16
5587	77	29	37	123.5	39.5	30	6	36.5	21
5588	27	10.5	9.5	25	14	12	6	45	10
5589	59	10	14	55.5	17.5	14	9	24	9
5590	26.5	12.5	6	19.5	13	14	6	11	8
5591	26.5	8	5	17	17	11	6.5	8	6

5592	29	9	6	22	13	12	7	10	5
5593	103.5	22	8	19	88	27	11	18	6
5594	21	19	7	15	11	21	5	11	14
5595	73.5	46	21	64	180	27	57.5	21	24
5596	44	24	11	30	28	28	12	19	7
5597	23	19	7.5	13	19	23	8	11	3
5598	117	19	10	18	13	23	25	13	36.5
5599	41	19	8	23.5	19	30.5	8	14	10
5600	23	23	7	18	14	27	7	14	4
5601	36	21	18	19	53.5	21	7	15	11
5602	22	17	6.5	22	22	21	5	12	3
5603	66	22	12	63.5	822	27	10	38	31
5604	70	36	15	60	446	57	12	85	69
5605	21.5	20	7	12	12	21	6	12	3
5606	34	26	8	21.5	16	37	11	113.5	7
5607	23	18	8	33	14	22.5	6	15	5
5608	29	18	8	38.5	27	22	31	16	7
5609	41	18	21	28	25	22	10	19	6
5610	33	15	8	27	16	20	7	15	10
5611	95	77	45.5	70	43	82	36	74	72
5612	83.5	64	26.5	67	34	66.5	24	59	60
5613	96	78	31	62	31	78	29	60	55
5614	97	69	31	61	30	73	30	65	68
5615	77	60	27	53	27	59	18	49.5	78
5616	29	23	11	33	16	27	9	18	14
5617	49	42	22	49	26	44	16	38	41
5618	51.5	39	22	43.5	22	45.5	17	33	30
5619	46.5	30	14	40	22	36	38	30	24.5
5620	44	34.5	19	55	33	37	16	34	41
5621	91	78	35	69	32	76	32	75	83
5622	42	33	15	40	16	31	13	29	28
5623	30	18	8	33	16	22	15	18	20
5624	68	56	27	49	23	64	21	49	44
5625	80	68	30	59	51.5	73	24	59	86
5626	42	27	14	36	18	36	15	27	42.5
5627	444	457.5	194	275	149.5	449	175	374	362
5628	646.5	305	195	209	103.5	518.5	184	60	402.5
5629	36	18	20	34	15	21.5	8	21	8
5630	28	17	8	26	9	19	8	13	4
5631	31	18	9	95	11	20	14	17	4
5632	16	12	5	7	6	17.5	4	7	1
5633	23	14.5	8	18	11	18	7	14	5
5634	24	14	6.5	16	10	19	4	10	3
5635	23.5	17	10	24	12.5	20	7	14.5	5
5636	26	18	10	22	15	21	7	13	12
5637	60	18	12.5	45	27	22.5	10	13	7
5638	26	15	6	22	12	20	8	15	7

5639	35	15	16	19	17	17	21	14	8
5640	36	14	9	50	13	19	7	18	10
5641	53	19	10	78	19	34.5	13	96	15.5
5642	19	14	5	18	12	16	4	10	2
5643	23.5	17	7	31	13	21.5	6	16	5
5644	112.5	97	44	63.5	50	89	34	85.5	78
5645	98	68	44	65.5	43	70	36	78.5	77
5646	46	32	21	34	30	36	15	39	56
5647	90.5	64.5	31	57	46	66	27.5	60	69.5
5648	65	44	21	44	28	46	17	82.5	48
5649	34	22	12	26	903	27	11	30	47.5
5650	121	81	43	72	51	80	42	75	77
5651	46	25	12	24	20	29	9	24	16
5652	376	339	149	206	139.5	325	140	295.5	300.5
5653	157.5	110.5	55.5	77	51.5	114	48	119	101
5654	93	69	26	45	33	69	24	59	75
5655	58	14.5	8	14	18	19	6	15	12
5656	115	83	46	62.5	44	87	38	75.5	104
5657	387	355	197.5	244.5	161	355	181	292.5	347.5
5658	87	60	25	46	34	67.5	24	53	56
5659	36	28	11	25	16.5	27	9	31	18
5660	116	81	42.5	58.5	71	89	33	78	71
5661	100	63	48.5	52	36	63	39	54	67
5662	39	26	9	22	22	32	7.5	21	46
5663	97	73.5	38	53	41.5	73.5	30.5	77	56
5664	75	51	28	46	47	52	27	54	46
5665	62	45	20	36.5	26.5	47	18	40	48
5666	54.5	34	13	53	17.5	37	13	40.5	65
5667	98	74	38	76	37	77.5	31	77	90
5668	65	45	16	35	23	48	18.5	44	36
5669	21	16	7	11	10	17	5	17	6
5670	16	11	4	8	6	14	4	9	3
5671	30	14	10	14	13	15	5	22	4
5672	26	28	16	34	23	27	22	131	4
5673	31	11	7.5	10	23.5	13	4	12	2
5674	19	10	5.5	12	11	14	4	11	3
5675	49	15.5	14	17	25	16	11	21	40
5676	40.5	16	37	40	33	17	32	61	16
5677	20	11	5	10	73	13	5	14	3
5678	26	13	5	9	10	15	5	9	3
5679	61	16.5	12	17.5	45	17	11	40	6
5680	45	16	12	26.5	102	17	10	20	21
5681	29.5	13	8	13	21	14	13	11.5	5
5682	22	12.5	4	16	18	15	4	12	15
5683	49	18	7	20	11	17	7	18	60
5684	26	11	6.5	20	7	15	5	16	42
5685	37.5	20	13	17	9	21	7	15	57

5686	27	14	7	16	8	17	6	13	67
5687	26	14	8	16	9	14	8	12	17
5688	24	14	10	17	154	16	9	33	7
5689	20	12	9	13.5	15	15	7	39.5	12
5690	51	16.5	15	44.5	23	19.5	15	38	11
5691	34	33	19	32	48	33	22	138.5	12
5692	65	14.5	13	16	18	15	12	19	12
5693	25	14	8	14	13	15	8	21	5
5694	40	12	9	17	20.5	14	8	12	3
5695	21	9	8	19	14	10	7	14	7
5696	20.5	10	6	22	16	12	7	16	32
5697	19.5	19	12	23.5	18	21	10	100	3.5
5698	47	39	21	33	34	39	21	35.5	60
5699	17.5	11	6	11.5	16	13	6	23	9
5700	65	10	7	27	11	12	133	23	16
5701	33	11	7	28	15	14	27	22	7
5702	21	10	14	27	10.5	13	12	15	16
5703	20	9	6	13	9	10	5	12	3
5704	19	10	11	35	14	11	9	19	3
5705	29	15	15	14	18	13.5	9	17	9
5706	28	13.5	48	25.5	20	16	10	21	33
5707	32	10.5	8	24	17	13	7	21	71
5708	27	10	12	30	29	13	23	14	25
5709	39	16	12	24	26	25	18	29	25
5710	25	20	17	20	24	21	18	82	6
5711	29	11	11	21	20	13	13	17	35
5712	27	12	7.5	22	24	14	15	13	16
5713	33	9.5	20	15	17	12	7	16	36
5714	19	10	4	6	9	12	4	7	2
5715	40	15	19	26	21	12	16	22	9
5716	17	10	7	10	7	12	6	9	3
5717	23	12.5	14	9	6	10	15	9	3
5718	17.5	5	10.5	10	9	6	10	13	5
5719	15	8	6	9	7	11	4	11	2
5720	16	7	6	9	7.5	10	7	8	2
5721	17	10	12.5	15	14	8	8	15	6
5722	46	13.5	11	18	25	15	9	40	11
5723	21	10	8	16	31	11	18	12.5	6
5724	11	4	4	5	3	5	3	5	1
5725	16.5	8	7.5	8	7	12	8	12	4
5726	24	9	12	13	9	9	40	12	6
5727	34	13	9.5	17	26	13	16	20	8
5728	72	20	16	23.5	26	21	21.5	91	23.5
5729	55	20	12	18.5	31.5	33	722	31	20.5
5730	42	20	12	21	66	17	18	48	9
5731	64	30	20	97.5	30	29	28	94	19
5732	51	6	11	16.5	77	5	10	11	27

5733	54	10	8	37	38	10	10.5	34	72
5734	106.5	10	9	15	10	10	11	12	6
5735	27	20.5	13	16	17	23	8	38.5	5
5736	13	6.5	5	6	5	9	3.5	6.5	1
5737	198	30	16	30.5	20	28.5	12	32	40
5738	67.5	41	13	27	16	40	10	31.5	36
5739	37	23	15	31	15	25	12	34	21
5740	47	23	15	32	17	23.5	11	27	25
5741	47	34.5	17	31	16	33	17	31.5	35
5742	60.5	45	21	37	23	42	16	39.5	39
5743	67.5	49	22.5	41	22	53	20	52	65.5
5744	28	12.5	9	20	11	14	7	17.5	22
5745	52.5	32	19.5	32	15	33	15	43.5	47
5746	22	10	9	16	20	10	5	12	28
5747	49	32	18	30	18	34	16	36	38.5
5748	78	53	21	41	20	56	30	51	62
5749	45	28.5	14	26	16	32	12	31	76
5750	37	15	13.5	20	21	15	22	18	25
5751	43	28	15	36	14	28	11.5	32	23
5752	60.5	39	19	33	26	37.5	17	39	37
5753	32	9	8	19.5	15	10	10	15.5	42
5754	23.5	11	7	14	9	13	5	12	9
5755	37	19	13	27	24	20	12	20	21
5756	17	7	5	20	9	7	6	13.5	19
5757	22	9	9	21	10	10	6	14	10
5758	36	20.5	13	25.5	13	22	11	29	22
5759	51	16	8	24	10	18	7	18	19.5
5760	67	50	30	40	21	48	20	45	49
5761	84	40	24	37	33	40	14	36	39
5762	36	12	9	13	8	11	6	12	26
5763	48	31.5	13	28	19	37.5	12	28.5	38
5764	44	33	15	30	18	31	12	30.5	53
5765	20	8	4.5	16	8	11	7	11	25
5766	52	38	21	51	19.5	38	17.5	36	40
5767	25	13	8	20	11.5	15	10	15	17
5768	27	12.5	10	22	30	13	6	22	23
5769	82	52.5	23	40	23	53	22	51	53
5770	29	8	7	20	10	9	13	18	12
5771	26.5	14	8	17	8	14	7	16	16
5772	50	27	16	33	17	28.5	12	31	28.5
5773	131.5	12	9.5	18	11	13	7	15.5	27.5
5774	18	10	6	17	17	10	6	12.5	6
5775	76	67	29	52	28	66	23.5	78	59
5776	47	28	14	31	26	28	11	30	32
5777	50	37	19	35	22	38	17.5	38	54
5778	49	32	14	31	19.5	35	13	28	44
5779	57	33	19	41	19	36	13	43	32

5780	59.5	30	15	33	28	33	12	33	34
5781	25	9	8	48	13	10	7	30	25
5782	29	9	14	20	11	9	8	19	13
5783	28	10	7	19.5	12	11	7	17.5	8
5784	26	9	8	20	13	11.5	6	39	22
5785	27	12	9	20	16	10	7	23	6
5786	18	7	6.5	26	77	10	6	16	9
5787	53	16	19	33	13	19	10	22	25
5788	26	12	7	17	11	13	7.5	30	2
5789	19	11	10	14	12	10	10	33	53
5790	23	6	6	9	17	6	26	11	10
5791	21	9	8	22	9	9	7	13	18
5792	221.5	7	7	15	8	7	6	10	7
5793	18	5	7	14.5	16	5	5	15	9
5794	20	7	6	29	12	9	6	13	7
5795	19	10	10	12	7	8	9	9	3
5796	21	8	9	17	11	11	12	16	15
5797	27	9	11	19	13	10	13	25	6
5798	20	7	7	8	8	9	6	12	6
5799	42	8.5	24	15	45	10	22.5	12.5	6
5800	26	8	32	13	407	9	13	11	5
5801	109	15	13	18.5	43.5	11	19	18	26
5802	72.5	23.5	39	86.5	89	17	88	71.5	48
5803	19	7.5	7	14	13	8	6	16	6
5804	18	7	6	14	8	9	6.5	11	12
5805	453	9	10	12	9	14	5.5	14	3
5806	16	6	5	8	8	8	6	8	2
5807	44	32	32.5	35	43	26	44	125	10
5808	26	17	15	14.5	18	18	12	22	6
5809	66	52	21	23	21	23	39	40	11
5810	24	18	12	19	17	19	10	56	7
5811	26	19	12	16	22	22	9	40	5
5812	32	15	8	15.5	13	19	10	18	3
5813	53	56.5	30	122	37	57	14	41	77.5
5814	55.5	23	16	21	20	21	11	35	12
5815	52.5	59	28	130	85	60	15	40	55
5816	39	27	19	27	37	27	19	92.5	6
5817	76	91.5	32.5	155.5	44.5	77	25	56	84
5818	24	80	19	51	18	19	10	37	26
5819	53.5	59	34	134	33	63	16	40	73
5820	16	11	8	14	11	15	7	21.5	5
5821	64	58	29	137	33	61	11	41	90
5822	25.5	18	32	17	16	19	11	28.5	10
5823	54	58	29.5	139.5	33	65	17	38	53
5824	67	46	28	176	47	55.5	12	41	39
5825	21	15	8.5	27	15	16	9	18	24
5826	54	50	34.5	142.5	29	56	11	38	48

5827	23	15	15	23	19	17	10	24	40
5828	75	104	42	166	56	78	33	58.5	89
5829	19	13	10	14	12	16	36	16	5
5830	58.5	41	30	135	28	53	13	38	76
5831	18	14	9	16	12	14	7	21	6
5832	79.5	63.5	41	305.5	64	86.5	23.5	50	71
5833	24	18	14	36	23	16	15	60.5	8.5
5834	48	52	36	132.5	48	55	15	39	86.5
5835	17	13	7	15	9	15	6	16	9
5836	71	72	40	174	34	68.5	21	48	92
5837	32.5	20	13	25	24.5	17	19	55.5	223
5838	57.5	51	35	147	43.5	64	15	40	65
5839	23	14	8	14	18	16	16.5	17	5
5840	77	78	39	167.5	37	71	22	50.5	84
5841	31	16.5	16.5	29	140	18.5	24.5	30	42
5842	62	52	35	144.5	75	53	15	36.5	67
5843	44	24	27	46	53.5	19	22	112	54
5844	53	42	32	144	26	59.5	13	36	52
5845	60.5	20	21	38	114	18	14	66	67
5846	70	62	37	179	69	62.5	20	51	79.5
5847	41	18	16	28	69	18	20	40	27
5848	66.5	73	35	176.5	44	70	22	53	69
5849	58	62	34	146.5	32	58	26	41	83
5850	45	17	17	17	63.5	16	14	28.5	11
5851	64.5	54	25	118	26	75	14	42.5	64
5852	60	26.5	22.5	54	71.5	28	26	72	31.5
5853	56.5	51	30	140	36	54	16	40	174
5854	52	32	23	42	39	26	21	88	18
5855	52	41	24	130.5	51	50	11	32.5	115
5856	23	18.5	9	15.5	77	18	13.5	18.5	8
5857	54.5	31.5	26	43	45	19	36	119	27
5858	65	73	33	158	47	65.5	25	46	67
5859	27	18	14	31	23	17	12	27.5	13
5860	34	23	13	29	23	28	13	29	11
5861	97	74	47	157.5	45	87	29	89	111
5862	53.5	60	31	148	26	59	16	36	134
5863	40	23	16	27	24	23	13	50.5	18
5864	56	22	17	25	21	24	21	80	66
5865	48	54	28	115	29	56	13	37	72
5866	40	24	22	24	36	23	29	51	9.5
5867	79.5	52	45	161.5	53	61	17	69	128
5868	45	20	17	22.5	16	21	13	40	35
5869	61	73	35.5	139	32	62	21	49	73
5870	109	53.5	32.5	48	32	49	25	123	39
5871	55	62.5	33	154	31	61	18	40	127
5872	65	38.5	19	37	27	39	17	59.5	54
5873	59	56	28	146.5	34	68	19	41	47

5874	83	36	31	39	25.5	29	17	111.5	25
5875	48	48	29	118.5	34	49	11	33	103
5876	111.5	91.5	47.5	62	62	86.5	33	96.5	98
5877	56	68	29	162.5	54.5	63	18	48	69
5878	53	33	24	35	25	36	18	60	33
5879	47	49	28	165.5	30	57	11	41	52
5880	61	19	13	23	20	19	12	42.5	28
5881	99	84	69	180	39	94	21	49.5	106
5882	46.5	35	27	117	24	46	9	30	44
5883	48	28.5	19	30	21	25	19	54	27
5884	54	40	28	131	29	45	9	36	87
5885	44	27	30	32	28	27	15.5	92	29
5886	68	65	38.5	172	36	59.5	18	45	49
5887	49	21.5	14	30	19	22	11	50.5	30
5888	63	53	31	167	39	45	22.5	38.5	76
5889	51	24.5	24	25.5	21	24	13	44	13
5890	48	39	30	149	60.5	50	12	41.5	123
5891	55	53.5	33	170.5	31	57	16	73	52
5892	86	73	33	155	36	97	20	42	68.5
5893	35.5	22	17	26	17	20	14	75	23.5
5894	67	20.5	14	26.5	23.5	21	14	64	17
5895	55	49	31	135	31	54.5	25	45	59.5
5896	49	36	21.5	31	23.5	32	16	53.5	38
5897	52.5	60	33	144.5	30	57	19	42	72.5
5898	100	64	39	50	34	64	28	78.5	75
5899	63	61	31	175	39.5	60	18	41	105.5
5900	64.5	36	23	32	23	32	18.5	70.5	49
5901	59	37	25	126	28	48	14	32	63.5
5902	62	20	17	22	23	20	17	36.5	46
5903	109	39	46	128.5	23	47	11	33	50
5904	56	23.5	15	25	24	22	11	48.5	23
5905	44.5	43	24	123	26	46	10	32	87
5906	72	55	24	40	25	46	26.5	60	60
5907	58.5	74.5	29	145.5	34	67	19	43	70
5908	60	49	34	126.5	31	57.5	11	44	77
5909	39	20	15	26	18	20	12	51	22
5910	58	36	21	34	40	32	16	42.5	48
5911	68	87	38.5	169.5	42	79	29	47	85
5912	47	30	31	37	29	29	24	154	51
5913	38	21	16	29	34	21	13	48	69
5914	50	44	24	124	22	45.5	13	32	49
5915	46	23	16	27	20	21.5	14	46	102.5
5916	51	43.5	29	140.5	23	51	12	34.5	43
5917	104.5	24	25	64	23	23	20	56	27
5918	34	34	18	93	17	45	7	29.5	42
5919	107	44	23	37	34	40.5	19	82.5	46
5920	33	31	20.5	92	27	43.5	7	27	28

5921	50	34	23	42	55	28	16	98.5	33
5922	33	26	19	88	18	35	11	19	24
5923	34	15	15	21	18	16	12	61	31
5924	40	29	21	77	24	38	22	33	44
5925	119.5	67	35	52.5	35	69	26	90	80
5926	33.5	33.5	19	78.5	17	45	6	27	31
5927	55	32	16	37	27	28	22	61.5	38
5928	53	34	23	86.5	19	46	34	31.5	32
5929	30	17	12	21.5	15	17	9	31.5	35.5
5930	60	19	14	26	38	22	12.5	43.5	29
5931	66	40	27	87	20.5	49	12	35	58
5932	56	36	28	38	20	30	16	65.5	28
5933	347.5	182	28.5	143	29	258.5	13	83	111
5934	42	43	11	23	12	18	10	37	36
5935	109	50	62	139.5	99	50	21	43	48
5936	61.5	45	30	184	32.5	65	14	43	63
5937	42	19	20	24	19	18	12	48	42
5938	53.5	34	26	82	28	43	18	32	30
5939	64	39	28	137	41	43	19	35.5	173.5
5940	59	31	18	30	28	29	16	46.5	43
5941	48	35	22	85	76	45	9	26	40
5942	38	24	18.5	25	16	21	14	43.5	38.5
5943	43	28	25.5	152	18	36.5	15	36	32
5944	43	22.5	15	24	18	18	11	40.5	40.5
5945	90.5	43	36	346	32	52	15	91.5	36
5946	35	18	11	19	18	16	10	39	67
5947	57	42	31.5	190	22.5	50	25	55.5	41
5948	51	29	17	32	18	27.5	15.5	42	58
5949	146.5	41	34	213	20	50	18	142.5	37
5950	85.5	46	17.5	39	23	46	18	46	69
5951	70	41	69.5	236	43.5	50	179	127	61.5
5952	46	39	15	28	17	21	11	37	62.5
5953	54	34	47.5	316	18	47	18	107	44
5954	42	19	12	30	17	20	12	28	18
5955	85	81	40	329.5	36	62	39	240	82
5956	46	20	13	25	21	18	10	31.5	20.5
5957	46	37	27	141.5	28	47.5	332	43	50
5958	39.5	18	12	28	26	17	12	28.5	17
5959	90	46	33	132	30	126	15.5	47	119
5960	35	16	13	28	13	16	9	28.5	38
5961	214	531	73	590.5	173	220	138.5	167	274
5962	46	22	11	26	22	24	11	33	32
5963	272	58	58	403.5	75	67	49	157.5	99
5964	95	52	24	47	27	51	21	69	50
5965	59	40	27.5	143	21	53	22	41	57
5966	31	19	11	25	17.5	17	12	29	73.5
5967	45	39	29	146.5	457.5	53	11.5	53.5	71

5968	49	27.5	14	30	21	24	13	35	46
5969	62	37	33	143	74.5	45	10	40	53
5970	76	47	23	39	36	44	22	50	56
5971	40	35.5	23	96.5	24	43	7	26	30.5
5972	46	22	14	30.5	23	18	12	39	31
5973	48	39	29	168	33	48.5	8	87.5	65.5
5974	90	61	31	48	28	52	25	55.5	58
5975	38	29.5	21	136	2397	40	33	31.5	33
5976	129	79	38	52	32	70	29	74.5	70
5977	42	35	26	135	50	39	12	50	44
5978	35	14	10	29	11	13	9.5	32.5	22
5979	48.5	54	26	166	20	50.5	12	36	62
5980	33	13	11	17	11	12	14	29.5	6
5981	47.5	53	19	125.5	25	55	12	30	100
5982	36	16	11	31.5	13.5	15.5	13	30	24
5983	71	50	24	92	20	50	12	38	54
5984	34	20	14	29	20	16	19	61.5	20
5985	37	31	26	118	19	41.5	35.5	28	45
5986	28	12	9	27	10	11	12	23	6
5987	68	58	33	161.5	31	67	16	62	120
5988	33	16.5	19	20.5	14	14	13	38	43
5989	62	46	39	269.5	55.5	52	29	75	214
5990	32	16	16	29	42	15	19	69	9
5991	32	14	12	28	8.5	14	11	29	7
5992	38.5	39	24	119	18.5	46	9	28	92
5993	33	17	18	29	111	14	58.5	30	15
5994	380	37	29	176	34	44	17	45	113
5995	52.5	16	22	24	17	15	186	33	23
5996	48	39	31	161	98	49	11	60	105
5997	46	19	13	19	15	18	14	45.5	14
5998	38	26	21	91	16	36	9	23	29
5999	58.5	23.5	28	30	29.5	19	34	46	27
6000	36.5	30	17.5	78	16	40	6	24	26
6001	41.5	15.5	14	16	88	15	9	25	13
6002	37	31	19	112	20	42	11	23	34
6003	67	18	37.5	27	19	17	18.5	96	62
6004	32	28.5	17	58	17	40.5	6	23	24
6005	74.5	22	12	23	22	16	12	42.5	40
6006	30.5	30	17	57	49.5	39	6	20	26.5
6007	28.5	28	16	75	13	39	7	20	33
6008	47	19	13	16	15	18	13	33	8
6009	63	31	21	100	20	39.5	8	26	34
6010	28.5	18	12.5	18	14	15	12.5	59	6
6011	33	31	16	54	16	38.5	17	21	24
6012	46	14	10	19	13	16	13	32.5	13
6013	36.5	37	17	93	19	43	10	25	38
6014	23	14	10	14	17	12	9	32.5	4

6015	44	35	27	203	26	47	11	33.5	93
6016	24	14	11	16	10	13.5	8	26.5	9
6017	51	37	22	74	142	59	10	28	54
6018	90	14	15	19	14	14	15	38.5	5
6019	42	25	21	137	75.5	40	15	31.5	83.5
6020	32	18	13	20.5	15	14	17	54.5	15
6021	57	41	34	167	49	51	17	68	72
6022	362	28	23	33	24	23	28	176	10
6023	47.5	38	23	162	22	53	22	37	69.5
6024	108	31	18	94	16	44	8	27	28
6025	85	18	28	30	22	14	24	79.5	10.5
6026	31	21.5	19	67	54.5	31	6	22	22
6027	26	12	18	15	14	11.5	8	32	8
6028	39	31	24.5	140	21	39	10	26	26
6029	58	30	21	89	19	39	10	37.5	31
6030	34	20	13	18	14	15	13	34	37
6031	58	18	20.5	35	32.5	16	36	31	169
6032	40	35	21	148	17	41.5	11	25	41
6033	234.5	27	21	41	25	18	21	76	159
6034	46	49	25	144	26	47	21	46.5	41
6035	51	26	12	19	11	10	11	33.5	7
6036	50	34	22	128	65.5	48.5	37	26	49
6037	48	9	49.5	14	7.5	9	7	19	5
6038	48	34	33	120	62	44	28	38	69
6039	50	29.5	15	29	21	28	16	43	41.5
6040	78	30	28	99	34	32	22	47.5	98
6041	42	21	18	20	17	20	11	31.5	22
6042	59.5	31	25	87	29	43	12	54	50
6043	47	20	13	22	18	16	28	34.5	22
6044	190	35	19	68.5	22.5	65	12	29	40
6045	63	47	25	86.5	46	49.5	15	36	39
6046	38	22	16	25	31.5	22	14	34.5	25.5
6047	59	63	25	131	23	60.5	17	31	65
6048	58	18	16	23	21	16	13	33.5	30
6049	36	30	21	98	24	36	7	33	45.5
6050	66	50	30	41.5	24	46	25	64	62
6051	189	46	38	210.5	29	118.5	13	80.5	133.5
6052	27	13	27	20	17	11	9	33	11
6053	63	30	18	70	24.5	38	9	24	57.5
6054	27	27	14	50.5	11	35	5	17	21
6055	28	11	9	17	28	11	8	24.5	13
6056	32	26	17	48	25.5	33	6	20	24
6057	103	32	21	28	25	31	24	42	42
6058	40	34	27.5	95.5	19	42	9	45	32
6059	51.5	27.5	16	29	22	26	16	43.5	40
6060	47.5	42	23	130	20	48	11	30	45
6061	40.5	24	14	25.5	16	20	13	38.5	40

6062	46	53.5	25	125.5	46.5	52	18	36	100
6063	31	18.5	8.5	21	15	16	8.5	30	22
6064	44	34	23	126	23	37	11	30	50
6065	37	17	14	30.5	14	15	16	35	27
6066	47	37	26	137	81.5	47	12	36	42
6067	59	49.5	27	120.5	26	64	13.5	30.5	61
6068	27	14	11.5	21	11	11	9	40	30.5
6069	40	36	23.5	108	31	41	9	27	37
6070	34	14	11	19	11	14.5	10	26	34
6071	54	51	26	122	25	61	17	33	99
6072	36	11	12	17	13	11	10.5	26	14
6073	53	34	20.5	100	24	43	8	28.5	58
6074	66	13	11	21	15	12	10	23	23
6075	39.5	32	32	111.5	22	40	8	31	51
6076	90	24	19	33.5	97	20	19	60	18
6077	49	21	16	25	18	16	16	120	12
6078	48	38	26	155.5	51	39	12.5	36	60
6079	62.5	35.5	16	32	22	32	24	44	70
6080	77.5	55	27.5	139	26	87	15	40.5	54
6081	42	13	13.5	19	13	10	9	22.5	16
6082	53	68.5	25	147	30	69.5	15	45	53
6083	27	10	12	17	14	11	8	23	12
6084	67	66	26	163	29.5	102	19.5	61	63
6085	30.5	14	23	18	18	10	10	28	28
6086	30	12.5	10.5	18.5	17	13.5	9	24	20
6087	36	34	20	89	20	36.5	8	25	45
6088	36.5	19	9	18.5	12	19	11	28	40
6089	41	15	14	18	18	12	11	37	17
6090	61.5	35	24	144	29	51	10	34	51
6091	35	12	11	18	13	11	11	25.5	13
6092	39	14.5	12	17	15.5	12	21	21	22
6093	24	9	11	16	13	9	8	23	13
6094	26	10	9	16	10	11	14	21	12.5
6095	41	44	23	141	26	41	12	32	38
6096	33	9	12	16	11	9.5	9.5	19	13
6097	57	46	21	138.5	75	51	27	34	56
6098	25	14	10	20	10	11	9	32.5	14
6099	46.5	35	25	154.5	24	41.5	10	30	79
6100	29	14	10	21	11	10.5	10	32	24
6101	46	35	19	27	17	32	14	44	27
6102	61	59	29	148	31	74	12	40	74.5
6103	32	11	15	16	14	11	20	22	19
6104	41	47	30	122	20	44.5	14	28	52
6105	37	14	12	22	18	13	11	22	45
6106	44	42	23	132	23	43	21	31	47
6107	901	180.5	24	30	24	155.5	14	62	38
6108	55	37	24	139	29	40	12	31.5	67.5

6109	51.5	17	21	53.5	57	14.5	16	36	29
6110	105.5	43	25	121	19	71	9	31.5	49
6111	32	15.5	13	23	24	12	19	52.5	38
6112	46	32	25	119	19	41	11	36	46
6113	47	23.5	12	20.5	17	15	12	32	37
6114	118.5	31	21	99	21	41	10	25.5	74
6115	43	17	11	32	12	17	11	34	18
6116	95	40	28	135.5	24.5	46	14	36	39
6117	40	36	21	112	29	41	10	35	97.5
6118	26	13.5	11	21	14	11	12	27	21
6119	68.5	51	22.5	158	22	65.5	12	37	79
6120	43	12	15	25	232	13	11	25	24
6121	68	63	34	167.5	27.5	62	20	43	117
6122	34.5	15	12	18	17	10	14	25	28.5
6123	55.5	65	25	158	39	52	20	40	60
6124	27	18	16	21	14	11	19	59.5	19
6125	73	55	32	135	40	60	24	57	62.5
6126	78	58	29	46	32	53.5	26.5	91.5	54
6127	214.5	101.5	31	136	19.5	513	8	144	110
6128	47	31	18	29.5	20	28.5	15.5	39	37
6129	43	50	22	128	23	52	14	34	68
6130	24	12	11	18	12	11	8	29	100
6131	63	92	27	185	39	64	30	61	71
6132	24	10	9	17	12	9	8	25	19
6133	39.5	23	12	30	27	16.5	13.5	52	52
6134	40	36	33	92	15	44	10	27	34
6135	55	77	35	186	50	58	25.5	49	84
6136	64	41	32	140	39	52	15	60	49.5
6137	52	57	32	155	30	52	18	37	79
6138	43	35.5	16	28	22.5	25	15	42	33
6139	47.5	44.5	32	176.5	28	59	14	70	51
6140	64	59	26	250	30	54	20	46	56
6141	62	37	23	35.5	24	37	18	47	61
6142	64	63	28	140	31	55.5	23	49	64
6143	59	37	21	28.5	18	39	16	49.5	40
6144	59.5	73	27	169	33	68	23	44.5	85
6145	31.5	14	11	18	10	13.5	7	25	35
6146	25	13	11	22	11	10	12	24	21
6147	50	50	28.5	121	30	52.5	15	43	55
6148	63	71	35.5	158	39	67	25	53	61.5
6149	31	13	11	20	13	14	11	31	14
6150	59	53	29	131	30.5	56	19.5	40	66
6151	31	15.5	14	20	27	12	12	35	18
6152	50	50.5	30	129	27	54	15	43	72
6153	47.5	34	20	32	18	28	15.5	56	72
6154	67	56	41	147	30.5	78	14	56	76.5
6155	72	51	32	131	30	57	26	40.5	67

6156	27	14.5	11	18	45.5	11	12	31	23
6157	52	51	30	116	28	57	12	39	64
6158	27	14	11	20	16	14	9	29	14
6159	63.5	63	43	137	31	60	18	46	48
6160	61	59.5	31	166	35.5	54	16	43	84
6161	73	62.5	34.5	141	30	66	19	43	66
6162	132	40.5	21	38.5	23	41	21	67	38
6163	38	22	16	24	17	22	14	34	26
6164	44	38.5	33	122	22	47	11	36	53
6165	50.5	37	26	149.5	26	48	10	38	51
6166	47	27.5	25	28	24	26	15	38	55.5
6167	46	35	25	97	20.5	45.5	10	27	30
6168	26	12	13	18	15	9	8	27	46
6169	62.5	43.5	23	37	22	37	20	57	39.5
6170	256	142.5	38	152	34	739	15	306	133
6171	65	66	34	149.5	33	66.5	20	43	84.5
6172	25	11	9	18	10	9	8	21	27
6173	44.5	38	28	148	23	49	15	32	38.5
6174	42.5	16	13	22	15	16	12	44.5	23
6175	49	47	32.5	130	29	49	17.5	62.5	80
6176	35	17	13	22	17	13	10	32	36
6177	45	41.5	26	105.5	34.5	45	11	29.5	49
6178	53	53	28	140	30	54	17	37	51
6179	32	15	15	33	22	13	9	36	16
6180	50	42	28	127.5	30	54	14	31	45
6181	32	18	14.5	24.5	17	17	14.5	30	43.5
6182	65	84	32	164	38	66	27	48	72
6183	52	28.5	16	26	18	26	16	40	38
6184	67	74	32	166	34	59	20	46	72
6185	41	78	13	24	16	21	15	55	9
6186	63.5	93	33	191	39	77	29	57	85.5
6187	26	12	11	16	12	11	30	40	6
6188	49	55.5	28	152	25	52	21	36	53
6189	23	23	34	18	15	30	8	16	6
6190	68	102	42	211	41	71	32	74	80
6191	66.5	41	11	25	16	26	15	22.5	11
6192	58	71.5	36	167	78	60	25	54	97
6193	26	72	16	34	24	26	37	22	7
6194	64.5	57	33	176	34	62	16	45	55
6195	31	23	13	22	19.5	26	12	17	13
6196	59	70	30	152	34	66	23	45.5	68
6197	27	23.5	18	27	26	28	26.5	23	13
6198	53.5	51	29	129	29.5	51	14	40	47
6199	38	20.5	11	19	17	26	16	16	5
6200	54.5	57	28	145.5	34.5	55	15	36	53.5
6201	36.5	19.5	8.5	25	15.5	32	11	21	3
6202	41	39	29	134.5	322	47	15	35.5	70

6203	43	23	9	37	20	31	9	29	5
6204	54	47.5	34	152.5	83	51	15	38	54
6205	31	19	22	21	19	24	13	19	10
6206	50.5	41.5	66.5	134	25.5	46	22	34.5	103
6207	32.5	19	9	39	15	22	7	17	7
6208	71.5	61	35	136	37	72.5	19	46	65.5
6209	36	37	22	31	1209	27	15	21.5	44
6210	60	65	35	149	39	59	28	49.5	97
6211	29	22	9	22	17	29	12	19	7
6212	53	53	29	137.5	31	47	20	41	48
6213	51	30	17	57	19	33.5	16	28	21
6214	64	57	31	148	54	56	20	41	108
6215	43	24.5	14	46	20.5	35.5	13	23	30
6216	50	58	32	141	32	53	16.5	37	64
6217	59.5	28.5	15	47	21	36	15	29.5	28
6218	84	70.5	25	55.5	28	73	23.5	57	70
6219	62	56	31	142	27	55	21	45	79
6220	80	57	27	69	30.5	61	24	60	60
6221	52.5	41	24	149	29	56.5	12	32	45
6222	46.5	37.5	13	42	22	40	12	29	35
6223	54	60.5	28	137.5	38.5	55	19	43	67
6224	35	21	13	46.5	22	28.5	8.5	24.5	14
6225	55	50	30	131	21	44.5	15	38	63
6226	53	55	27	131.5	28	51	14	41.5	89
6227	30	22	7	30	14	27	11.5	19	21.5
6228	57	59	74	160.5	54.5	52	31	46	87
6229	44	39	27	114	24	43	18	33.5	56
6230	46	31.5	16	48	24	38.5	14	30	32
6231	53	53	29	117	27	51	37	39	49
6232	61	47.5	22	59	26	59.5	21	44	68
6233	45	40	31	121	22	44	12	38	102
6234	46	33.5	15	68	21	32	18	39	51
6235	49	42	28	147	22	42.5	12	32	84
6236	36	25	12	58	17	30	10.5	34	17.5
6237	52	39	24.5	98	19	71	10	33.5	46
6238	68.5	53	25	62	25.5	56	22	53	41
6239	39.5	29	21	140	21.5	40	8	25	31
6240	34.5	26	17	36	17.5	31	9	22.5	17
6241	50	52	28	117	27	47	22	32	90
6242	26	20	10.5	30	16	23	11	21	12
6243	52	45	27	120	26	49	31	32	74
6244	41	25	11	32	16	30	11	22	27
6245	84	66.5	38	70	43.5	73	39	65	60
6246	52	48	25	122	25	46	16	32.5	45
6247	73	57	37.5	58	29	65	29	67	61
6248	57	46	39	130.5	32	44	15	42	65
6249	40	26	12	27	16	25.5	12	17	46

6250	43.5	43	28	158	24	43.5	14	31	56
6251	40	28	12.5	25	17	31	10	20	24
6252	60	41	25	89	18.5	77	13	40	38
6253	31	20	11	29	15	25	6	16	67
6254	41	32	23	107	21	40	12	32	36
6255	26.5	18	13	56	15	22.5	18	29	8
6256	41	36	26	120.5	24	36	10	30	121
6257	26	18	9	20	16.5	21	9	14	39
6258	44	42	32	121	27	45	14	33	43.5
6259	48	30	10.5	34	15	34	10	23	21
6260	52	35	26	120	25.5	46	11	29	41
6261	124.5	25.5	15	28	35	28.5	14	31.5	12
6262	113	54	26	87	25	110	13.5	43	65
6263	48	35	23.5	88	21	43	10	32	29
6264	36	19.5	13	49	24	22	14	42	28
6265	45	34	24	235	28	50	12	34	105.5
6266	40	25.5	19	30	15	31	12	20	104
6267	58	37	27	135	22	51	16	39	54
6268	76.5	29	11	26	20	30	19	18	32
6269	51.5	40	24	105.5	86	46	18	43	31
6270	41	16	17	28	39.5	25	13	33	10
6271	25	16	13.5	35	17	18	19	20	23
6272	58	37	24.5	114	66.5	43.5	41	32	43
6273	24	14.5	6	19	174	19	11	16	11.5
6274	55	35	27	107.5	22	44	13	28	58
6275	22	14	8	25	15	18	6	14	11
6276	49	44	22	81	26	52	12	31	34
6277	21	12	6	22	11	18	10	14	5
6278	160	37	22	65	19	48	11	28	38
6279	18	14	21	15	13	19	39	13	7.5
6280	45.5	32	22	132	19	39.5	9	31	27
6281	22.5	20	275	27	19	22	9	27.5	36
6282	51	56	31	87	29.5	57.5	16	40	49.5
6283	46	16.5	13	16	22	20	9	19	13
6284	83	80	34	184	53	67	36	71.5	59
6285	26	19	34	26	21	21	16	53.5	11
6286	47	34.5	29.5	116.5	27	40.5	14	39	47
6287	155	21	17.5	28	27	23	16	19.5	14
6288	36	32	20	78.5	27	35	8	28	32
6289	21	18	7.5	17	16	18	10	12	11
6290	35	33	22	101	25	40	10	26	48
6291	29	17	10	35	15	27	9	24	8
6292	100	49	20	81.5	56	114	11	36	62
6293	21	13	6.5	14	8	17	7	13.5	5
6294	38	31	22	133	19.5	41	10	38	26
6295	28	18	7	20	12	21	12	16	42
6296	33	30	20	80.5	23	37.5	10	43	31.5

6297	58	14	8	16	19.5	20	8	13	6
6298	44	35	25	119	37	44	11	35	50
6299	67.5	76	31	72	390	93	22	1236	54
6300	39.5	29.5	24	135	22	41.5	8	33	43
6301	30	19	15	24.5	19	23	9	21	12
6302	38.5	32	24	160	17	37	8	36	34
6303	28	18	17	33	17	24	8	20.5	19
6304	72.5	31	20	110	20	41	10	40	32
6305	28	16	14	14	12	18	8.5	13	5.5
6306	56	38	28	129	28	46	13	64	48
6307	33	17	13	43	12	24	5	47.5	12
6308	65	33	23	139	19	47	10	43	48.5
6309	22	15	8	17	12	22	8	14	4
6310	34	32	23	75	19	38	10	40	26
6311	15	13	8	19	11	17	9	12.5	13
6312	51	38	23	126	43	50	9	30.5	35.5
6313	17	11	8.5	19	10	17	8.5	12.5	3
6314	57	30	19	90	21	39	9	22	30
6315	26	22	12	55	44	33.5	11	187	13
6316	37	34	21	90	20	39	9	27	33
6317	20	16	6	29	11	20	7	13	7
6318	41.5	31	31	58	21	43	13	30.5	42
6319	39	22	23	33.5	23	29.5	16	19.5	12
6320	95	33.5	22	84	24	40	10	25	34
6321	30.5	22.5	30	19	17	24	13.5	16	11
6322	80.5	78	43	175	39	86	26	62	68
6323	28	19	12	58.5	19	29	11	33.5	10
6324	72	56	33	150	59	63	22	42	50
6325	27	19	7	57	15	25.5	16	89	7
6326	62.5	55	39	176.5	38.5	68	21	49.5	82.5
6327	31	21	7	28	19	27	7	19	6
6328	62.5	61.5	41	147	37	71	20	52	63
6329	490	583	74	235	80	1258.5	21.5	671.5	452
6330	117.5	71	37	67.5	29	81	28	65	79
6331	71	50	35	164.5	45.5	59	15	76	61.5
6332	92	58	30	64	41	56	27	57.5	58.5
6333	71	67	37	147	34	68	23	43	66
6334	51	19	12	51	13	23.5	10	25	33
6335	53	27	13	31	18.5	31.5	13	25	15
6336	69	57.5	36	155	41	69.5	37.5	49	72
6337	77	57.5	25	58	29	60	21	57	136
6338	71	53	43	148.5	36	77	14	44	65
6339	69	64	38	188.5	53	73	21	45.5	94
6340	75	47	24	56.5	27	46	23.5	45	61
6341	42.5	47.5	23	67	23	64	10	30	40.5
6342	84	75	45	188	42	72	38.5	52	141
6343	42	23	20	50	16	29	12	28	43

6344	61	62.5	37	140	35	65	19	50	64.5
6345	68	39.5	25	59	21	45	23	46	52
6346	61	59	35	136.5	34	67	18.5	43	69
6347	93	73	43	81.5	34	83	32	67.5	66
6348	81	62.5	40	158.5	36	74	18	53.5	61
6349	73	75	42	158	38	80	25	54.5	99
6350	79	147	76	201.5	37	94	27	692	58
6351	33.5	18	9	32	15	22	9	19	17
6352	82	65	36	172	43	70	22	55	71.5
6353	80	54	26.5	55.5	30	50	26	51	52
6354	59.5	47	35	152	36	51	16	42	67
6355	91	54.5	27.5	95.5	38	60	22.5	60.5	60
6356	94	92	46	193	49	77	38	57.5	80
6357	70.5	73	40	188	40	76	25	57.5	86
6358	49.5	24	14	44	24	28	12	24	69
6359	75	64	39	169	536	76	20	56	93
6360	36	22	12	33	16	27	10	28	63
6361	78	27.5	14	63	22	34	20	59.5	27.5
6362	84	77	41	190	46	78	31	63	143
6363	110	85	46	80	55	88.5	40.5	90	75
6364	236	33	16	54	14.5	36	12	37.5	31
6365	53	55	28	102.5	27	59	16	35	48
6366	79	37.5	19	62	31	42	27	63	55
6367	80	74	34.5	156	36	84	22	50	131
6368	76.5	57	27	81	29	53	20	53.5	54
6369	73.5	83	38	152	42	74	22	49.5	71
6370	117	39	23	55.5	23	48	34	41.5	39.5
6371	47	23	10	42.5	14	26	10.5	24	25
6372	74	30	15	41	19	36	16	31	26
6373	102	54.5	42	179.5	52	60	18	48	58
6374	90.5	67	41	162	38	65	19	61	64
6375	98	73.5	38	79	45	78	34	65	63
6376	77	62	57	159.5	34	61	21	50	92
6377	93	39	17.5	52.5	21	38	31.5	33	34.5
6378	66	51	42	167	33	59.5	15	46	67.5
6379	72	41.5	24	67.5	23.5	44.5	22	52	42
6380	80	68	43	169	41	78	24	53	68
6381	59.5	20	13	48	14.5	23	13	42	41
6382	46.5	22	14	43.5	22	23	41	22.5	15
6383	79.5	88	49	223	42.5	76	29	60	115.5
6384	43.5	20.5	8	37	10	29	8	16.5	63
6385	36	26	21	52.5	19	33	8	23	24.5
6386	53	29	13	36	14.5	36.5	10	22	33
6387	47	98	27	140	24	50	11	32	34
6388	71	35	20	55	20	43	20	39	57
6389	44.5	47.5	34	141	29	60	13	37	38
6390	35	41	9	46	11	20.5	8	21	23

6391	78.5	49	29	85	29	62	14	43	45
6392	53	24	10	36	13	29	13	27	34
6393	50	47.5	30.5	134	29	57.5	8.5	35	45
6394	43	36	30	77	19	50	9	25.5	35
6395	47.5	26	12	50	17	29	12	30.5	58
6396	132	46	42	103	56	57.5	42	85.5	47
6397	45.5	12	10	35	15	15.5	10	22.5	15
6398	74.5	47	42.5	125	40	64.5	20	50.5	51
6399	41	17	14	44	16	23	14	21	23
6400	93	44	273.5	101	111	52	37	58	57.5
6401	88	62	28	63	28	64	26	55	65
6402	61	44	29	174	25	56	13	62.5	153
6403	45.5	28	11	42.5	12	28	10	30	27
6404	52	46.5	32	127	37	54	13	33	44
6405	40	15	11	37	22	20	11	21.5	15
6406	62	59	34	197	27	56	15	40	60
6407	37	21	11	43	12	23.5	10	23	38
6408	48	16	10.5	42	70	20	8	20	21
6409	59.5	46	31.5	80.5	37	60	21	39	50
6410	34	26.5	8	45	9	21	8	34	11
6411	72	49	31	174	29	63	25.5	54.5	112.5
6412	26	14	9	25	9	16	7	15	7
6413	33	12	9	32	11	16	7	13	5
6414	43	36.5	28	110.5	21.5	39.5	9	34	30
6415	31	15	9	53.5	13	19	8	39	26
6416	53	42	27.5	164	30	52	21	44	42
6417	26	15	6	22	10	16	7	10	3
6418	39	35	21	61	18	43.5	9	26	32
6419	27	11	8	23	9	16	6	15	2
6420	63	43	28	114	26	51	14	32	35
6421	52	17	11	56	15	21	17	39	25
6422	96	25	20	62	66.5	25	12.5	40	40
6423	65	43.5	28.5	73	30	69.5	15	36	43
6424	61	29	11	93.5	17	20.5	44.5	44	14
6425	74	43.5	27	117	24	57	12	32	36
6426	28	14	8	14	11	17	11	11	5
6427	51	33	29.5	151	39	41	16	35	55
6428	48	18.5	13	29	24.5	19	11	17	28.5
6429	57	46	34	175	399	58	33	65	60
6430	55	67.5	24	129	19	93.5	18	708.5	27
6431	58.5	40	28	161	72	49	13	47	69
6432	81	15	14.5	57.5	26	17	13	22	32
6433	56	42	32	128	25	51.5	12	41	127
6434	64	20	13.5	167	16	28	38	30	18.5
6435	43	39	26	132	23	50	11	31	50.5
6436	59.5	27	8	24	14	77	12.5	26.5	18
6437	43	37	27	118	41	49	11	34	56

6438	33	16	12	21	15.5	21	7	26	5
6439	54.5	40	59	132	37.5	50	17	98	87
6440	45	15	6.5	20	9	16.5	6	24.5	12
6441	45	40	30	77.5	25	48	20	35	51
6442	29	14	8	18	7.5	14	7	12	3
6443	54	41	47	124	27	53	14	68	49
6444	32	13	8	28	12	17	5.5	20	8
6445	56	36	28	131	24	48.5	13	48.5	36.5
6446	25	12	7	18	11	14	6	11.5	3
6447	50	46	29	96	25	52	62	46	37
6448	31	12	8	21.5	25	15	10	15	13
6449	40	33	23	80	21	42	11	26	36
6450	58	18	12	27.5	25	18	14	21	6
6451	46	41	53	133	27	52	11	35	38
6452	45.5	38	29	127	23.5	50.5	15	34.5	52
6453	42	15	14	34	15	19	21	25	13
6454	67.5	40	37.5	103	58	52	13	49	49
6455	200.5	13	9	25	29	16	18	12	12
6456	56.5	41	30	133	28	50	20	33	46
6457	32	13	8	24	10	18	16	28	7
6458	40	41	22	71	18	53	9	28	32
6459	95.5	14	12.5	42	12	18	10	18	7
6460	65	37	34.5	178	42	48	47	51	49
6461	48	39	29	109	31	49.5	34	45	34
6462	42	21	11	44	15	28	11	20	15
6463	44	23	56.5	182.5	25	29	22	102	7
6464	37	26	25	109	20	26	10	23.5	29
6465	20	16	5	7	5.5	19	5	10	1
6466	16	9	5	8	5	14	5	8	2
6467	40	31	23	84	20	42	9	26	29
6468	28.5	12	6	28	10	15	9	13	7
6469	42.5	43	26	98	22	45	16	30	35
6470	29	11	6	27	7.5	13	7	12	33
6471	54	38	30	152	24	53	14	31.5	40
6472	28	14	8	32	11	14	9.5	25	10
6473	53.5	41	32	106	33	52	12	53	121
6474	33	13	8	35	14	16	7	24.5	37
6475	57	33.5	38	145.5	20	41	14	27	34
6476	331	205	6	36	10	1146	12	25	95
6477	36	16	12.5	88	29	27	47.5	99	9
6478	102.5	112	44.5	293	40	76	31	304	78.5
6479	30	12	13	33	10	16	14	19	15
6480	57.5	41	32	117.5	34	49	17	38	48.5
6481	25	9	7	31.5	10	11	33	20	27
6482	48.5	37	26	108	27	44	13	32	52
6483	45	11	9	35	12	13	7	20	15
6484	45	14	9	35	9.5	18.5	10	17	17

6485	39	33	20	67.5	22	39	203	24.5	27
6486	24	11	8	34	20	14	7	15	5
6487	199	48	28	176	50	138	17	46	77.5
6488	30	13.5	9	39	14	16.5	10	25	34
6489	39	32.5	21	71.5	20	40.5	9	22	31
6490	42	21	13.5	23	16.5	26	33	22	9
6491	67	54	35	123.5	27	53	34	94	48
6492	41.5	35	23	89	21	47	12	36.5	33
6493	139	10	9	35	19	11	8	15	6
6494	114	43	33	93	27.5	59	19	58.5	48.5
6495	89	10	8	16	11	12	7	10	8
6496	41.5	37	28	104	22	49	13	100.5	33
6497	27	18	8	30	16	14	8	30.5	58
6498	42	35	21	81	17	43.5	10	25.5	28.5
6499	45	15	10	23	12	16	10	21	11
6500	38	34	25	90	22	46	14.5	28	34
6501	35	13	11	78	26	15	23.5	37	11
6502	339.5	39	27	165	35	48.5	10	47	47.5
6503	23	9	8	18	12	10	7	12	13
6504	63	75	30	162	30	53.5	22	54.5	125
6505	31.5	17	9	30	9	16	8	14	24
6506	47	39.5	32	134	24	49	12	38.5	43
6507	31	13.5	10	24.5	17.5	14	7	13	19
6508	40	35	23	66	30	40	12	26	41
6509	362	49	85	355	41	59	47	148	49
6510	44	12	10	26	9	14	8	12	14.5
6511	49	39	28	93	30	45.5	12	35.5	37
6512	36	14	16	32	17.5	17	21	21	9
6513	83	40	27.5	85	25	49	13	30.5	58.5
6514	257	11	9	29	11	12	13	19	11
6515	73	33	26	114	64.5	42.5	28	39	43
6516	65	39	31	97.5	58	51	20	36	41.5
6517	45	12	13	36	10	17	24	19	9
6518	46.5	29	27	114	25.5	33	13	28.5	65
6519	34	17	9	20	11	17	8.5	12	210.5
6520	48	39	28	99.5	26	51	17	53.5	40
6521	35	12	8	26.5	11	11	8	11	8
6522	68.5	44	31	86.5	75.5	51	20	55	50.5
6523	28	10	11	26	21	13	16	16	18
6524	105.5	40	32	130	1234	46	15	48	59
6525	32	18	9	36	15	13	11	17	57
6526	796.5	66	37	211	34	73	22	89	140
6527	44	11	9	45	14	15.5	11	38	47
6528	46	41	24	92.5	132.5	43	14	29	37
6529	45	51	9	24	18	13	9	12	48
6530	40	33.5	25.5	104	18	45	9	31	30.5
6531	32	14	8	43	9.5	16	10	36	6

6532	58	62.5	26	135	27	53	20	38	59
6533	52.5	32	17	42.5	18	32	14	30	53
6534	64.5	57	71	177	47	61	19	54	84.5
6535	105.5	35	10	42	13	47	7	33	40
6536	48	43	29	143.5	25	45	13	33	64
6537	45	20.5	14	35	17	22	12	25	44
6538	60.5	69.5	35.5	142	30.5	59	23	46	118
6539	88	72	36.5	73	30	69	29	73	64
6540	88	49.5	34	147	28.5	58	21	47	57
6541	49	27	13	38	15	26	11	27	30
6542	50	51	30	138	26	46.5	14	34	62
6543	36.5	30	24	113	18	40	10	26.5	40
6544	55	27	15	42	17	30	12	37	35
6545	74	51	31	156	32	62	15	52.5	124
6546	44	25	10.5	41.5	34	26	14.5	26	43
6547	43	33	24	119	34.5	39	11	35.5	75.5
6548	43	21	12	41	14	25	12	30	31
6549	49.5	52	31	129	31	46	17	35	60
6550	64.5	43	22.5	51	33	45	21	43	44.5
6551	48	42	31	140.5	24	46.5	14	35	52
6552	64	39	19	53	25	42	17	38	50
6553	49.5	39	30	138	26	47	11	37	59
6554	39	19.5	11	49.5	12	19	9	24	22
6555	56	43	32	128	25	46	15	34.5	49.5
6556	45	35	36	129.5	22	44	12	36	78
6557	36	14.5	9	40	10	17	8	18	44
6558	44.5	50	34	142	28	47	18	48.5	51
6559	38	17	10	36	11	20	8	19	40
6560	34	15	8	33	12	15	11	13	13
6561	63.5	55	43	187	35	50	24	69	104
6562	49	29	14	38	13	37	14	26	41.5
6563	69	90	40	203.5	73	65.5	30	55	127
6564	34	12	8	40	15	15	8	16	22
6565	58	78	44	187	36	85	24	79.5	83.5
6566	39	10	9	28	13	12	7	15	14
6567	20	8	8	29	8	12	6	13	8
6568	40	12	10	46	13	17	8	52	50
6569	43	38	32	149.5	28	39.5	24	33	77.5
6570	43	11	9	33	9	13	7	16	19
6571	65	80	40	194	44	63	32	59	115
6572	50	30	16	42.5	19	29	16	31	54
6573	57	59	40	166.5	29	55.5	21	40	120
6574	28	10	11	33	10	13	9.5	16	17
6575	65	58	32	277	37	59	21	56	88
6576	75	42	21	55	20	48	21	45.5	46
6577	69	49	25.5	211.5	31	49	17	42	81
6578	27.5	9.5	14	36	9	13.5	7	14	17.5

6579	72	83	33	143	34	57	26	57	68
6580	32	11	8	29	27	13	6	18	24
6581	41	19.5	13	44	12.5	23	11	23.5	23
6582	54	58	29	155	43	50	18	56	54
6583	39	18.5	13	40	15	23.5	13	26	32
6584	69	76	33	169	83	62	26	48	70
6585	31	12	9	33	10	16.5	9	15	28
6586	58	51	27	146	27	59	14	46	67
6587	45	22	11	35.5	13	27	10.5	25	34
6588	61	80	32	169.5	36	62	22	43	71
6589	57	31	21	53	23	32	18	43	44
6590	46	42	38	133	26	47	14	32	57
6591	60	39	18.5	48	18	41	17	44	48
6592	62	56	36	151	30	53	19	46	53
6593	47	13.5	9.5	39	13	14	13	18	16
6594	70	51	31	156	24	64	15	40.5	75
6595	56	32.5	14	44	15	36	10	29	24.5
6596	62	63	30	140	32	59	19	42.5	52
6597	35	13	11	41	16	14	10	32	36
6598	66.5	89	50	206.5	54.5	64	32	57	101.5
6599	24	11	8	29	8	16	9	15	18
6600	85	59	33	177	29	49.5	21	47	67
6601	33	11	14	34	9	12	7	17	30
6602	71	50	27.5	166	23	65	14	36	55.5
6603	38	13	9.5	38	11	13	7	16	12
6604	65	48	26	55	20	46.5	25.5	49	52
6605	49	47	27.5	155	25	44	16	38	62
6606	36	24	12	40	12	23	12	25	19
6607	61	95.5	34	175	33	64	26	50	90.5
6608	57.5	75	33	135	33	53	22	41	90.5
6609	57	51	26	134	27	47	17	37	51
6610	68	43	19	49	21	48.5	18	39	45
6611	42	38	26	125.5	20	46	10	29	46
6612	50	36	26	149.5	18.5	48	9	31	88
6613	50	31	19	58	20	33	13.5	36	29
6614	69	40.5	25	137.5	38	54	10	31	58
6615	49	20	14	44	18	20.5	12	29	35
6616	77.5	61	31	153	27	75	15	42	63
6617	47	30	15	49	17	31	12	38	31
6618	45	39	28	143	25	45	12	34	39
6619	34	18	14	40	11	19	12	21	15
6620	46	33	31.5	127	24	42	13	37	49
6621	27	11	9	27	10	18	8	13	56.5
6622	62	60	102	166.5	47.5	54	23	43	60
6623	28	12	7	31	11	13	7	30	7
6624	101	49.5	28	123.5	108	55.5	16	40.5	59
6625	78	102	32	226.5	46	65	30	51	96

6626	28	9.5	10	31.5	14	14	12	15	7
6627	37	31	25	128	20	37	11	27.5	73
6628	22	11	9	36.5	9	17.5	9	59	10
6629	20	9	7	23	9	10	5	9	5
6630	96	82.5	42	174.5	87	81	30	98	96
6631	22	9	7	24	10	12	6	12	10
6632	60	65.5	38	136	31	64	17	54.5	82
6633	34	12	13.5	21	11	12	9	12	7
6634	56	72	35	212	39	63	20.5	47	80
6635	26	10	8	11	9	15	9	7	6
6636	49.5	54	27	124.5	35	58	16	37.5	65
6637	19	7	4	12	7	7	5	7	3
6638	61.5	69	36	166.5	59.5	70	23	50	72
6639	20.5	10	10	23	10	14	6	32	29
6640	48.5	50	28	127	35	47.5	14.5	33	69
6641	43	9	9	17	10	12	6	11	12
6642	19	7	7	20.5	8	8	6	14	5
6643	104.5	71	34	179	50	63	25	47	87.5
6644	38	9	8	20	7.5	10	8	10	24
6645	60	52	27.5	125	33.5	50.5	17	41	69.5
6646	27	11	9	17.5	10	16	9	11	6
6647	49	10	13	20	12	10	12	12	4
6648	116	48	27	134	28	49	13.5	33	53
6649	17	7	8	14	8	10	9	10	8
6650	65.5	58	32	132	48	83.5	12	40.5	64
6651	25	14.5	6	14	11	20	7	11	3
6652	27.5	16.5	8	26	12	18	6	19	6
6653	49.5	53.5	27.5	149	32	51	18	48	61
6654	41	19	11	25	12	20	9	22	3
6655	53.5	43	23	102	20	66	14	31	37
6656	24	14	6.5	21	10	20	5	13	6
6657	41	67.5	25	98	17	46	12	51	32
6658	27	15	9	26	11	22	7	16	5
6659	54	53	23	82	24.5	58	14	27	37
6660	520	454	12	41	27	1933	46	75	212
6661	56	37	25	130	18	51	15	35.5	49
6662	26.5	16	7	15	12	20	10	12	7
6663	40	38	23	104	17	43.5	20	29	32
6664	93	35.5	22.5	89.5	19	41	18	25	30
6665	42.5	19	11	45.5	28	22	10	58	15
6666	39.5	37	21	93	24	41	16	24	28
6667	71.5	22	11.5	51	562.5	24	12	30	42.5
6668	35	16	9	31	13.5	17	7.5	22	13
6669	29	17	8	24	13	20	6	15	52
6670	38	22	13	35	16	24	8	19	15
6671	52	41	31	191.5	35	64	30	59	62.5
6672	49	32	14	36	15	37	12	32	23

6673	43	36	27	173	28	48	27	40	32
6674	40	24	12	32	17	27.5	15	25	25
6675	64	39	42	150.5	29	48	33	42	35
6676	57	38.5	22	53	25	39.5	17	45	45
6677	114	90	53	104	118.5	115	32	99	109
6678	29.5	15	11	31	12	21	7	15.5	30
6679	68	71.5	31	126.5	35.5	93	12	80.5	85
6680	350	47	22	60.5	25	43.5	21	44	55
6681	55	40	31	131	22	46	17	37	44
6682	33	18	9	36	15	22	8	21	29
6683	47	35	23	127	23	44.5	9	37	50
6684	81	50	37	65	37	62	22	63	69
6685	50	36.5	17	114.5	17	51	10	38	35
6686	57	41	21	51	27	48	22	45.5	39
6687	41	33	19	97	18	43	14	32	31
6688	58	39.5	20	49	25	47	21	40	38
6689	32	34	22	97	135	41	8	32	30
6690	32	17	12	31	26.5	18	9	17	32
6691	130	107	54.5	87	43	101.5	37.5	88	115
6692	66.5	34	19	63	61.5	41	8	28	28
6693	61	40	18	45	19	39	21	34	34
6694	53	30	19	59	17	37	9	27.5	28
6695	40	20.5	12	37	15	24	20	25	22
6696	71	34	21	73	23	40	17	36	39
6697	47	28	18	48	17	35	13	34.5	37
6698	46.5	40	21	61.5	17	53	10	23	63.5
6699	59	33.5	16	51	20.5	41	14	40	51
6700	53	39	25	85.5	32	66.5	11	28	38
6701	43	26	10	42	18	32	11	28	31.5
6702	45	43	24	118	28	40	16	32	84
6703	63	36	18	56	18.5	38	16	34	38
6704	38	37	23	118	48	46.5	12	36	46
6705	43	15	9	35	22	18	7	16	10
6706	52	56	29	135	29	51	21	44	55
6707	90	62.5	31	68	33	64.5	23.5	61	111
6708	44	50	25	146	25	49	22	41	53
6709	35	23	8	39	12	29	9	25	35
6710	45	41	23	136.5	21	44	11	34	43
6711	88	67.5	54	68.5	35	75	27	64	70
6712	47	49	24	132	21	44	12	38	81
6713	37.5	23	12	43	34	25.5	10	27	23.5
6714	48	48	33	126	28.5	48.5	14	35.5	54.5
6715	69	34	15	49	24.5	34	12	31	29
6716	49.5	59.5	28	151	26	51	24	47.5	71
6717	32	21	11	29	23	24	9	18	16
6718	34	30	46	122	23	36	10	34	33
6719	96	28	13	92	22	45	93	232	12.5

6720	38	36	22	116.5	17.5	39	10	30	47.5
6721	31	13	9	53	13.5	18	6	30.5	6
6722	360	331	40	186	29	1028	15	500.5	238
6723	25	16	9	28	12	19	7	15	6
6724	47	44	26	145	25.5	41.5	15	36	73
6725	22	12	6	26	10	14	7	14.5	11
6726	41	41	25	137	23	44	12	57.5	52
6727	45	15	11.5	44	21	19	9	29	28
6728	39.5	37	25	139	22	37	12	31	40
6729	30	12.5	13	25	12	12	7	11	5
6730	38	35	23	101.5	19	38.5	14	28	36
6731	37.5	35	12	27	19	29	8	18	13
6732	40	32	21	100	19	35	10	25	37
6733	50	17	14	29.5	20	18	12	14	36.5
6734	53.5	48	26	135	29	48	17	38	50.5
6735	80	54	28	108	27	56	19	38	49
6736	24	13	9	19	11	15	7	14	5
6737	1788	1191.5	414.5	692	271	966	331	1012	861
6738	52	41	24	120.5	19	51.5	11	33	37
6739	66	13	7	20	13	14.5	15	21	18
6740	39	41	25	124.5	20	48	12	37	41
6741	34	16	11	31	32	19	8	18.5	12
6742	23	10	10	22	11	14	6	12	2.5
6743	42	34	20	89	23	44	9	25	32
6744	28	13	12	31	16	17	9	21	5.5
6745	40	38	23	121	22	45	11	30	43.5
6746	20	13	7.5	27	12	14.5	5	11	5
6747	54	57.5	36	133	34	51	19	46	61
6748	25	12	7	22	13	16	6	13	3
6749	57	70	27	159	60	51	22	44	52
6750	22	14	8	27	14	16	7	32	20
6751	47	55	28	129	103	46	18	48	66
6752	33	17.5	11	27	15	18.5	9	17	19
6753	41	39	21	116	21.5	39	12	33.5	50
6754	24	19	12	34	9.5	17	12	14	16
6755	39	39	23.5	114	20	43	10	27	53
6756	89.5	68.5	34	65	44	76	27	72	58
6757	39	44	23	114.5	27	43.5	13	29	92
6758	46	46	33.5	130	25	44	15	33	68.5
6759	46.5	48	29.5	110.5	34	43	15	38	57
6760	63	35	21.5	143	36	39	10	33	41
6761	38	22	12.5	41	24	27	11	25	28
6762	48	36	21	127	18	47	9	33	46
6763	75	49	23	56	27.5	53	20.5	51	46
6764	68	70.5	36.5	141	43	69	26.5	68	62
6765	36	32	23	105	19	32.5	11	29	99
6766	58.5	34	21	124	31.5	40	10	31	37

6767	87	62	29	64	30	68	37	60	58
6768	45	34	20	118	17	44.5	10	32	42
6769	54	34.5	17	50	29	39.5	16	58.5	82
6770	64	56	35	137.5	37	73	26.5	59	56
6771	40	36	24	119	19	43	12	32	96
6772	90	67	37	81	34	77	27.5	79	89
6773	47	44	25	128	29	50.5	16	34	53
6774	36	34	17	86	19	46	9	32	50
6775	34	32	22	124	18	43	10	38	40
6776	31	17	10	39	19	19	10	47.5	30
6777	30	29	20	111	19	37	9	27	33
6778	34	22.5	14	31.5	13	28	7.5	20.5	14
6779	31	29	17	81.5	14	35	7	27	24.5
6780	49	33	26.5	184.5	19	40	19	47	40
6781	29.5	24	18	85	24	33	7	19	21
6782	78	39	35	67	28	41	17	44	49
6783	80	33.5	23	152.5	22	42	16	34	43
6784	42	20	26.5	51	15	24	11	20	20
6785	73	46	37	65	27	58	22	56	45.5
6786	53	32	14	56	32	37	13	34	43
6787	40	19	11	40	20	22	8	39	92
6788	46	15	10	36	15	22	10	21	37
6789	88	61	38	70.5	28	63	22.5	74	71.5
6790	60	39	23	57	24	41	16	41	39
6791	39	22	10	44	24	23	17	24	30
6792	46	30	16.5	43	67	31	16.5	34	48
6793	60	39	26.5	54	18	45	16	44	41
6794	51	24	13	48	16	30	12	29	45
6795	60	28	14.5	57	21	35	21	40	38
6796	54	32	20.5	56	25	39	20	43	71
6797	49.5	19	9	48	17	21	9	27	23
6798	58	50	31	111	34	54	15.5	41.5	66
6799	75	50.5	26	66	47	54	21	47.5	56
6800	94.5	65.5	33	123	36.5	96	15	38	72
6801	118	71	30	65	31	80	25	64.5	81
6802	87	60	31	130.5	31	91	15	42	75
6803	58	39	30	123	53	49	14.5	33	133.5
6804	42	28	10	46	21	25	10	27	28
6805	62	37	30	144	25	55	14	37	51
6806	108	79	37	81	36.5	82	27	86	86
6807	50.5	39	27	116	28	40	11	26.5	41
6808	54	33	17	41.5	25	35	16	33	41.5
6809	58	37	30	135.5	29	46	11	35	56
6810	56	33	20	47	23.5	31	14	35	25
6811	79	53	28	130	31	116	9	73	67
6812	67	50	28	59	30	53	23	48	67
6813	45	35	43	105	37	42	11	31	49.5

6814	71	53	23	60	27	55	19	51	59
6815	51	39	36	141	26	43	17	33	39
6816	58	39	19	66	19	40	16	40	37
6817	31	12	11	39	15	16	8	22	33
6818	49	41	28	125	26	46	11	32	55
6819	38	22	13	46	16	23	12	23	24
6820	58	42	33	150	26.5	57	11	37	46
6821	52	22.5	16	44.5	16	26	15	23	24
6822	58	36	30	138	40	43	15	38	54
6823	46	19	12	39	21	21	11	25	41
6824	48	35	32	119	26	44	10	36	47
6825	50	31	16	44	19	35	14	34.5	26
6826	51	42	38	122.5	30	51.5	13	38	58
6827	52	36	18	54	22	41	15	33	39
6828	54	23	12	37	19	25	10	40	80
6829	54.5	55	26	135	32.5	51.5	16	33.5	69
6830	32	16	9	38	29	18.5	9	19	10
6831	45	38	28	97.5	25	44	15	27	52
6832	45.5	29	17	54	28.5	32	14	56.5	29
6833	54	40	30.5	109	27	55	11	40	48
6834	27.5	14	7.5	45	25	16	10	32	20
6835	45	39	28	109.5	24	42	13	29	45
6836	54	30	18	50	23	32	15	31.5	53
6837	50	41	29.5	119	25	41	13	30	44
6838	46	35	19	42	17	36	14	33	27.5
6839	53	39	30	116	31	41	18	34	66
6840	68.5	34	27	140.5	30	41	38.5	32	71
6841	25	13	10	39	13	18	8	39	8
6842	39	16.5	12	62	23	20	10	26	40
6843	64	47	30	124	35	46	16	31	51
6844	30	16	9	43	12	20	8	19.5	15.5
6845	54	37	29	123	24	38.5	15	27.5	49
6846	53.5	40	30	112	29	44	11	35	53
6847	645	119	9	41	26	122	9	31	45
6848	48	27	29	83	23.5	31	10	24	55.5
6849	114	73	34	83	41.5	76.5	35	79	72
6850	69	44	21	56	21	46	18	47	50.5
6851	54	45	26	132	25.5	47	15	33	62
6852	102	86.5	32	97	40	80	27	95	79
6853	84	56	44	133	29	84	14	38	85
6854	38	31	9	39	16	25	8	28.5	20
6855	44.5	32	25	113	24	39	8	26	49
6856	94	66	24	74	25	67	24	90	62
6857	83.5	64	31	163	32	53.5	22	40	59
6858	73	43	22	62	27	42	17	45	55
6859	30.5	15	7	32	15.5	16	19	20	26
6860	55	37	58	122.5	29	45	10	37	58

6861	71	49.5	15	53.5	16	63.5	14	39	40
6862	59	41.5	26	104.5	26	57	9	32	48
6863	93	60	23	70	23	59	20	58.5	67
6864	67	85.5	33	167.5	42	65	26	55	86
6865	145.5	109.5	49	91.5	43.5	110	42	150	99
6866	72	57	29	124.5	31	77	14	39	52
6867	41	23	7	37	12.5	38	8	28	26
6868	75	52	26	57	27	52	17	54	58
6869	92	67	30	76	25	66	24.5	65.5	51
6870	53	43	28	110	27.5	50	14	30	53
6871	72	47	19.5	58	23	47	20	47	36.5
6872	49	41.5	26	141.5	30	43	15	35	45
6873	80	52	21	70	23	54	20	60	68
6874	50	37.5	28	143	28	39	12	31	68.5
6875	93	67	31	68	30	71	26	64.5	52
6876	42.5	38.5	33	127	23	37.5	10	28	45
6877	21	10	6	32	11	13	8	12	4
6878	24	11	6	28.5	84	13.5	6	13	11
6879	49	31	24.5	123	26	40	10	35	96
6880	19	11	7	34	10	14	6	16	6
6881	49	30	24	93.5	24	39	9	31	41
6882	34	13	6	30.5	235	12	49	14	9
6883	47.5	39	27	119	27	43	25	35.5	42
6884	23	8	6	23	38.5	12	9	10	6
6885	66.5	38	28	122	35	45	14	42	65
6886	29	11	9	40	17	17	14	17	14
6887	50	45	30	126.5	26	39	12	31	46
6888	22	9	7	30	13	12	8	11	8
6889	55.5	59	30	142.5	31	45	22	34	56
6890	21	11	6	27	16	13	6	14	4
6891	37	26	21	86.5	15	34	8	25	29
6892	24	15	8	29	12	15	6	13	25
6893	39	27.5	23	86	28	36	7.5	21	28.5
6894	43	17	6.5	32	15	36	8	14	11
6895	35	28	23	83.5	17	35	7	22	26
6896	28	9	8	26	24	15	12	15	6
6897	38.5	27	21	99	17	34	7	21	37
6898	36	14.5	8	29	15	16	7	12	76
6899	45	37	29	145.5	21	39	11	31	42
6900	40	13	9	36	17	13	8	18	6
6901	33.5	30	24	105	15	35	8	25	28
6902	33.5	12	10	20.5	50	12	7	17	14
6903	37	35	21.5	97	19	29	10	27	27
6904	17	8	5	17.5	7	10	5	7	2
6905	34	26.5	19	76	15	33	7	22	23
6906	79	27	32	128.5	45.5	58	165	551	14
6907	39	28	22	107.5	16	36	9	21	26

6908	23	9	8	18	10	12	8	25	8
6909	50	18	12	30	18	23	15	17	7
6910	34	28	20	86.5	15	34	7	19.5	28
6911	22	9	6	29	10	11	8	12	9
6912	33	23	22	89	17	28	6	19	27
6913	20	9	6	25.5	14	10	6	12	6
6914	37	26	41	95	16	35	8	23	23
6915	20	7	5	26	23	10	5	14	47
6916	35	24	24	75	16	29	8	18	26
6917	30	10	59	51	105	13	25	14	30
6918	29	18	20	59	13	23	6	17	27
6919	25	29	9	48	17	18.5	9	36	99
6920	51.5	28	19	73	18	38	8.5	20	28
6921	31	15.5	9.5	45	15	19	11	24	101
6922	33	25	17	54	14	31	5	17	22
6923	31	11	11	21.5	16	15	19	13	5
6924	36	24	19	85.5	18	31	7	19	27
6925	62	16	27	53	31	25	23	64	26
6926	42.5	28	18	67	29	34	7	21	37.5
6927	18	9	9	28	11	10	10	12	8
6928	36	25	21	103	18	35	9	19	29
6929	111	10	9	17	12	10	9	17.5	13
6930	48	55	27	157	25	48	16	33.5	47.5
6931	28	9	11	21.5	15	10	8	14	37
6932	31	24.5	15	49	12	28	5	16.5	21
6933	33.5	28	15	49.5	14	35	6	17	23
6934	27	9	12	19	16	10	6	11	7
6935	55	29.5	23	128	311	39	25	48	44
6936	17	8	5.5	21	6	9	6	12	3
6937	50.5	39	27	157	47	38	18	41	46
6938	19	11	27	21	13	8.5	10	9	2
6939	46.5	26	24	114	17	35.5	9	31	30
6940	25	9	5	26	8	9	5	11	3
6941	54	33	29	74	45.5	42.5	33	32	46
6942	20	8.5	7	67	8	17	5	33.5	3
6943	15	8	6	20	8	10	6	10	2
6944	90	30	26	69	26	35	34.5	42	26
6945	17	8	5	25	8	10	5	11	6
6946	53.5	30	25.5	95	43	46	10	28	34
6947	25	11	25	21	25	11	12	12	5
6948	259.5	34	42	102	38	40	13	38	34
6949	33	10	9	27	113	12	20	13	7
6950	110	35	25	70.5	23.5	39	12	30	106
6951	44	8	9	16	13	11	9	10	5
6952	30	11	14	57	18	11	22	13	10
6953	40	26.5	19	66	22	34	11	24	26
6954	38.5	14	9	19	24	27	14	12	8

6955	37	24	18	83	20	32	9	25	28
6956	19	10	7	13	12	19	7	12	4
6957	41	27	18	71	18	30	10	22	38
6958	52	7	7	19	11	9	173	10	6
6959	63	27	20	68.5	22	33	10	26	24
6960	47	10	9	40	12	11	8	23	36.5
6961	60	30	19	75	30	35.5	9	22	29
6962	36	11	10	43	13	14	14	28.5	7
6963	93	27	28	75.5	73	39.5	8	26	53
6964	20	13	6	23	11	9	7	16	30
6965	406.5	118	37	355	51.5	515	32	150	144.5
6966	69	21.5	13	77	18.5	27	10	65.5	31
6967	36.5	59	26	144	16	23	19	27	29
6968	25	9	19	29	9	12	9	15.5	4
6969	30.5	23	16	73	27	30	5	17.5	22
6970	34	13	9	32	15	15	10	14	13
6971	34	23	19	78	16	29	7	21	26
6972	52.5	27.5	15	48	17	27	13	24	25
6973	99	74.5	23	172	60	54	19	37	51
6974	41	23	14	50	17	26	14	36	21
6975	71	34	22	64.5	25.5	46.5	28	28	56
6976	22	11	15	30	15	13	6	15	19
6977	81	93	52	285.5	68	77	19	522	63
6978	24	11	13	36	11	12	9	15	20
6979	41	33	23	78.5	555.5	35	24.5	30.5	48.5
6980	35	20	13	35	15	23	11	24	26
6981	60	29	24	95.5	18	36	10	36	86
6982	19	9	6	25	10	9	6	13	26
6983	39	31	21.5	133.5	16	36	8	29	50
6984	77	53	29	57	25	55	23	53	55
6985	37	23	17	89	17	33	6	17	25
6986	35	23	11	38	14	21	23	24	29
6987	38	33	19	101.5	18	37	9	22	51.5
6988	52.5	28	16.5	50	16	29.5	13	30	32
6989	37.5	27	19	88	14	32.5	8	27	26
6990	32	14	9	27	10	16	10	18	63
6991	38	36	21	108	17	36	9	22	58.5
6992	41	28.5	15.5	45.5	16	26	16	37	30
6993	35	27	19	62	28	37	9	21	26
6994	36.5	25	18	68	21	31	6	23	24
6995	66	46	23	48.5	24	50	17	47	47
6996	60	31	33	115.5	23.5	43	11	34	36
6997	21	9	10	30	12	13	9	13	20
6998	46	32	24	76.5	21	39	12	30	98
6999	42	29	17	43	16	28	12	31	33
7000	73	27	22	63	19	32	10	25	29
7001	32	16	11	30	12	16	8	17	44

7002	76	34	25	75	60.5	66	10	42	40
7003	92	65	29	57	27	71	23	60	61.5
7004	57.5	26	19	66	36	32	11	21	28
7005	62	24.5	21	78	126	33.5	13	25	31
7006	34	11	14	45	12	12.5	12	17	18
7007	54.5	28	23	89.5	43	39	15	39	36
7008	56.5	35	23	108	20	37	11	27	121
7009	68	32	17	45	17	35.5	14	37	30
7010	46.5	25	30.5	67	24	35	11	26	46
7011	26	12	9	26	12	15	10	17	42.5
7012	44	28	19.5	61	18	36	7	24	24.5
7013	44	12	14	39.5	16	12	11	20	46
7014	38	25.5	28	106	16	29	6	24.5	23
7015	61	44	26	143	29.5	47	12	37	44
7016	20	8	6	20	8	9	6	11	20
7017	47.5	39	25	105	29.5	45	12	30	41
7018	82	51	29	63	29	55.5	26	54.5	50
7019	67	56	29	140.5	24	43.5	16	38.5	87.5
7020	18.5	8	5	30	623	9	5	11	16
7021	51	42	24	96	23	49	11.5	27	60
7022	43	37	22	104.5	20	36	10	27	41
7023	41	15	12	43	28	18	10	19	23
7024	43	38	22	162	22	36	16	29	40
7025	39	24	13	40	18	27	11	37	36
7026	62	44	26	60	22	43	19	46	58
7027	47	25.5	16	57	17	31	15	36	42
7028	61	41	23	61.5	22	42	21	47	86
7029	38.5	29	20	102	20	34	9	24	29
7030	37	24	14	42	18	25	12	29	49.5
7031	38	31	25	106	18	32	9	27	51
7032	22	10	8	34	11	14	9	16	19
7033	60	64.5	24	149	27.5	65	16	38	84
7034	43.5	39	22	106	21	35	12	24	37
7035	65	47	26	57.5	27	50	21	50	58.5
7036	42	39	22	120	21	40	13	27	38
7037	49	30.5	18	39.5	16	32	14	32	31
7038	67	44	22	54	23	43	20	53	52
7039	52	41	27.5	130	25	40	14	33	54
7040	41.5	36	19	115	20	35	9	30	57.5
7041	61	41.5	21	60	20	45	18	43.5	42.5
7042	40	30	21	115.5	19	32	24	25.5	39
7043	55	38	20	48	22	38.5	15	38	47
7044	64.5	40.5	22	110	55	40	11	29.5	41.5
7045	50	32	18.5	47	18	31	16	34	33
7046	42	43	25	126	28	40	15	30.5	47
7047	47	23	13	34	13	21	23	24	26
7048	40	44.5	24	121	26	39	13	38	46

7049	68	14	8	40.5	10	15	9	17	16
7050	56.5	43	21.5	113.5	71	39	12	29.5	63
7051	48	30	15.5	50	18	33	16	33	37
7052	49.5	38	35	120	30	42	12	28.5	50
7053	41	21	15	44	15	22	12	27	22
7054	48	31	23	134	23	36	14	30	42
7055	38	28	21	92	18	32	9	26	36
7056	26	9	8	27	14	10	9	13	15
7057	44.5	33	22	128	22	36	9	24	61
7058	60	36	25	48	75	38	20	38	39
7059	54	68.5	31	156	47	49	25	37	51
7060	36	15	9	33.5	12	18	9	18	20
7061	60	37	25	116	24	36	13	34	65
7062	17	8	8	39.5	8	9	6	14	4
7063	38.5	31	20	107	20	35	8	23.5	90
7064	21	10	7	33	14	11	6	15	12
7065	35	21	13	56	12	24	10	33	33.5
7066	56	37	26	109	22	41.5	11	35	43
7067	30	16	11.5	33	13	16	8	16	13
7068	42	34	26	122	26	34	10	31	43.5
7069	55.5	21	12	68	19.5	31.5	13	59	41
7070	43	31	21	122.5	21	36	8	26	30.5
7071	25	14.5	10	33	13	18	8	16	12
7072	48	38	27	138	27	37	15	41	41
7073	63.5	52.5	24	128	24	71.5	11	32	43
7074	30	23	18	54	14	23	29	45	65.5
7075	45	25	22	85.5	18	30.5	9	21	38
7076	31	16	7	20	12	21	5	12	3
7077	36	31	23	101.5	22	34	11	26	30
7078	18	14	8	18	13	18	8	15	3
7079	44.5	42	23	131.5	31	45	13	34	46
7080	36	16	12	28.5	17	21	29	19	5
7081	46	32	24	105	26	34	10	27	57
7082	41	33	24	106	20	38.5	11	26	56
7083	476	466	14.5	35	18	1988	39.5	62	147
7084	50.5	58	25	181	34.5	45	20	34	90
7085	20	12	6	16	809	16.5	14	11	4.5
7086	37.5	36	20	117	27	35.5	11	24	40
7087	38.5	28.5	22	109	24	33.5	8	25	31.5
7088	43	44	22	120.5	23.5	40	12	30	65
7089	38	14	9	17	19	14	6.5	14	5
7090	32.5	26	20	86	24	30	10	20	32.5
7091	36	16	9	29	18	17	31.5	18	16
7092	111.5	55	22	116	20	97	10	33.5	43
7093	43	18	10	23	30	19	12	19	10
7094	47	53	25	124.5	27	42	17	33	71.5
7095	32	79	20.5	66	13	54	18	338	8

7096	34	28	19	73	17	35	7	24	24
7097	23.5	11	7	31	10	17.5	9	15	4
7098	37	29	23	89.5	16	39	14	74	25
7099	22	12	11	21	10.5	15	9	12	3
7100	29	22	18	77	17	28	8	22.5	22
7101	65.5	19	11	33	21	26	13	21	19
7102	43	24	18	59.5	17	28	5.5	17	21
7103	19	12	5	23	10	14	8	15	7
7104	55	26	17.5	71.5	16.5	33	12	20	26
7105	19	10	7	28	10	14	7	12	3
7106	30	20	18	93.5	16	25	5	16	42
7107	257.5	32	50	158	43	40	15	42	56
7108	33	18.5	16	40	34	23	11	23	17
7109	28	23	16	49	15	28	6	16	21
7110	62.5	44.5	33	73	25	47	20.5	45	40
7111	42	28	23	72.5	18	38	8	24	25
7112	44	27.5	17	51	16	30	11	29	36
7113	55	23	19	66.5	16	29.5	9	20	27
7114	35	18	11	37	23	19	13	19.5	18
7115	31	22	16	63.5	15	29	6	22	22
7116	28	15	8.5	40	15	22	9	22	28
7117	22	14	15	57	13	13	6	17	34
7118	90.5	69	34	73	47	70.5	31	70	62
7119	41	22	16	71	59.5	27	10	24	25
7120	29.5	23	18	70	21	28	6	29.5	22
7121	37	25	18	63	18	31	10	22	23
7122	56	44	34	57	22	46	19	45	51.5
7123	1152	215.5	27	121.5	35	1639	25	178.5	396
7124	1044	255	37.5	145	38	1746.5	33	199.5	441
7125	54	37.5	19	57	19	38	19	44	37
7126	34	21.5	20	84	38.5	28.5	7	26	32
7127	45	25	16	50.5	24	29.5	38	36	20
7128	45.5	46	27	99.5	33	55	14	39.5	43.5
7129	56	41	24	60.5	25.5	43	133.5	44	37.5
7130	54	38	26	82	40	47.5	12	29	114
7131	52	41	26.5	82.5	59	52.5	11	40	36
7132	130	123	57	95.5	51	119	48	119	127
7133	26	16	9.5	38	11.5	18	8.5	22	35
7134	30	23	15.5	43.5	14	25	14	22	39
7135	37	22	13	54	15	23	12	27	23
7136	27	15	9	47	12	19	6	22	18
7137	52	42.5	16	62	23	30	17	39.5	28.5
7138	41	21.5	18	60	23	23	13	32	37
7139	71.5	62	36	80	29	65	27	60	57.5
7140	36.5	22.5	9	32	13	22	8	23	19
7141	63	50	31	67	26.5	50	23	53	68
7142	84	65	34	76	30	67	25	75	64

7143	46	30	18	52	17	32	41	34	28
7144	59.5	40.5	21	68.5	24	44	19	48	31
7145	28	17	10	43.5	12	20	8	21	12
7146	42	34	21	53	18	33	15	69.5	38.5
7147	35	25	13	49	20	26	14	30	26
7148	19	10	6	40	10	14	6	17	4
7149	17	11	10	40	8	15	7	18	5
7150	16	9	8	25	12	13	17	19	3
7151	67	58	27	138.5	28	77.5	15	40	83.5
7152	19	11	7	28	11	15	10	13.5	4
7153	49	39	27	110.5	27	51	12	31	40
7154	17	11	7	26	29	16	29	24	6
7155	47	55	30	130	30	53	18	39	57
7156	38.5	10	17	24	14	12	12	19	7
7157	54.5	54	31	114.5	32	51.5	20	35	79.5
7158	40	42	28	93	33	48	11	28	56
7159	30	17	9	21	12	18	9	26	113
7160	45	42	26	112	22	44	9	31	36
7161	38	10	8	41	13	15	70	29	31
7162	69.5	59	28	121.5	31	53	17	43	53.5
7163	37	22	60	68	106	28	34	31	26
7164	54	68	31	133	43	57	22	41	93
7165	18	10	10	24	10	10	6	13	7
7166	32.5	13	11	18	13	15	13	15.5	6
7167	58.5	49	26	132.5	28	71.5	13	37	53.5
7168	42	12.5	11	26	19	14	8	11	6
7169	53	41	29	121	25.5	48	14	33	44
7170	21.5	11	8	44.5	17.5	13	9	13	26
7171	63	66	33	147	35	59.5	20	40	64
7172	22	14	13	23	12.5	18	16	17	5
7173	57	53	28	126	36	61	16	37	58
7174	55.5	32	18	57	43	36	18	32	24
7175	50	53	28	108	29	53	17	34	61
7176	14	9	7	19	10	11	5	10	2
7177	53	53	28	122	29	50	15	38	62
7178	12	9	5	7	6	10	5	7	1
7179	49	50.5	27	134.5	30	49	14	35	57
7180	121	23	10	40	17	27	16	20	26
7181	66	50	28	121.5	29	50.5	16	35	54
7182	60.5	39	20	51	23	45	15	33	40
7183	51	41	25	101	29	48	11	29	57
7184	78	41	18	50	23	38.5	18	43	32.5
7185	34	15	9	27.5	15	22	7	23	22
7186	62	55	33	141.5	182	62	15	39	73
7187	47	26	13	51.5	18	32	13	34	51
7188	59.5	65.5	30	154.5	34	52	21	36	68
7189	54	33	18	38	16	33.5	13	38.5	31

7190	38.5	37	31	112	39	35	13	42	37
7191	47.5	17.5	16	54	16	20	9	24	22
7192	48	45	28	100	29	44	16	33	78.5
7193	53	50	29	131	34	48	18	39	69
7194	63	42	23	49	24	48.5	34	44	44
7195	57	44	28	111.5	29	61.5	11	74	51
7196	49	18	10	41	16	23	8	22	29
7197	48	53	26.5	121	27	50	17	36.5	56
7198	41	17	9	35	33	19	10	19	16
7199	62	48.5	28	122.5	27	57.5	16	37	80
7200	125	106.5	49	77.5	43	103.5	36	87	104
7201	48	43	29	146	24.5	47	13	34	57.5
7202	88.5	67	23.5	81	24	57.5	22	60	52
7203	40	35	22	104	22	45	10	30	37
7204	40	38	24	103	23	44	11	44	38
7205	34	16	10	42	12	22	11	21	12
7206	38	38	23	105	20	44	9	28	39
7207	77	60	27	58	25	51	24	49	56
7208	54	37	26	96	23	39	11	29	44
7209	83	66	33	64	27	68	26	63	58
7210	55	46	23.5	126.5	23	54	13	34	47
7211	71	48	27	59	26	54	18.5	49	56
7212	47	30	21	82	21	46	9	22.5	62
7213	101.5	78	35.5	78.5	36	83	30.5	114.5	62
7214	59	42	35.5	169.5	33	59	28	55	54
7215	65.5	39	20	47	22	40	16	44	34
7216	38.5	32	26	102	25	41	12	28	32
7217	352.5	470.5	268	588	316	314	453	2496	249
7218	161	24	13	67	22	28	38	43	42.5
7219	54	35	20.5	87	305	40	10	35	32
7220	36	19	8	30	14	19	11	17	21
7221	43	34	24	140	24	39	10	45	43.5
7222	50	16	13	44.5	18	20	12	29	22
7223	40	42	22	122	17	44	11	33	81
7224	97	72	31	72	30	80	30	68	64.5
7225	37	18	12.5	43	15	21	8	20	37
7226	63	41	26.5	124.5	23	47	11.5	49	78
7227	35	19.5	7	42	12	23.5	9	31	16
7228	62	39	26	143	32	53	21.5	43.5	49
7229	38	16	10	39	17	19	7	20.5	26.5
7230	37	39	24	82	24	44	10	23	54
7231	32	17	9	47	16	20	7	22.5	15
7232	54.5	42	23	91	32	66	11	32	35
7233	40	15	11	38	12	18	10	19	8
7234	87	43	28	87	31	53	14.5	35	80
7235	38	24	12.5	37	15	23.5	12	22	34
7236	38	30.5	28	71	29	38	7	22	201

7237	77	24	16	53	33	28	14	32	37.5
7238	34.5	29	19	50	36	37	8	20	23
7239	48	38	25	81	33	44	13	31.5	35
7240	43	35	24	142	31	41	292	47	89
7241	42	19	16	44	12	19	12	23	36
7242	40.5	33	27	155	31	40.5	11	38	33
7243	35	16	8	41	19	17	8	19	25
7244	39	35	25	95	23	41	13.5	40.5	37
7245	43	31	9	44	15	17	8	40	23
7246	39	32	24	83.5	22	45	7	24	85
7247	42	28	12	44	20	34	15	27	44
7248	48	32	25	104	24	41	9	31	42
7249	30	12.5	12	35	11	15	8	17.5	22
7250	38	30	24	109.5	22	38	8	32	37
7251	38	37	23	88	18.5	42	9	24	88
7252	38.5	19	19.5	30	14	24	10	25	42.5
7253	44	26	13	50	18	26.5	10	27.5	24.5
7254	38	16	10	37	13	21	10	22	41
7255	37	15	11	30	15	20	7	17	52
7256	115.5	75.5	46	87	38	79.5	30.5	82	89
7257	53	38	21	47.5	20	39	17	40	44
7258	69	42	21	58	21	51.5	18	48	80
7259	66	18	19	38	13	22	9	26	54
7260	43	22	12	42	15	25.5	12	25	22
7261	37	20	11	35.5	12	26	7	25	31
7262	37	19	9	32.5	9	15.5	7	22	35
7263	38	14.5	9	40	216	16	9	20.5	33
7264	59	37	19	48	21	40	18	52	31
7265	104	92.5	49	72.5	38	93	40	87	95
7266	70	47.5	20	66	24	51	22	77	55.5
7267	56	33	20	52	20	36	15	39	49
7268	95.5	74.5	32	66	29	72	29	74	82.5
7269	20.5	9.5	6	24	11	11	7	11	3.5
7270	22	11	8	23	14	15	6	17	5
7271	22	11	7	26	8	12	5	11	10
7272	28	12	11	18	9	12.5	7	11	5
7273	57	19	15	80	251	30	59	66	29.5
7274	50	11	22	16	12	14	7	11	13
7275	50	17	13	26	13	34	11	127	22
7276	34.5	13.5	7.5	36	8	17	6	27.5	13
7277	18	8	5	21	12	10	5	10	6
7278	33	10	23	18	23	13	9	12	7
7279	35	13	9	29	22	16	9	14	10
7280	28	13	8	23	165	14	9	13	7
7281	28	14	8	13	14	16	9	11	7
7282	61	38.5	18	55	19	42	14	48.5	34
7283	67	32	14	62	26	35	14	53	44

7284	46	20	9	48.5	12	21	9	31	30.5
7285	84.5	61.5	24	78	23	51	25	73.5	53
7286	28	12	8	34.5	11	15	9	26	39.5
7287	98	51.5	19.5	70	27	47	19	61.5	62
7288	48	28.5	10	50	16	30	11	37.5	19.5
7289	35	19	11	51	16	20.5	8	36	18
7290	47	21	11.5	55	15	23	10.5	41	27
7291	64	31.5	13	54	16	32	13	45	34
7292	20	8	5	27	11	11.5	6	10	6
7293	20	8	5.5	26	11	9	6	13	4
7294	19	10	5.5	17	17.5	12	6	12	2
7295	28.5	13	9.5	68	12	19	11	58	20
7296	24	9	7	20	12	11	6	12	11
7297	28	9	23	43	10	12	8	15	4
7298	32	12	8	19	9	13	7	14	10
7299	32	17	8	44	18	19	18	15	4
7300	17	8	7	15.5	10	11	6	10	7
7301	22.5	10	5	11	12	10	7	9	5
7302	68	13	10	63	27	18	11	63	110
7303	54	13	22	34	18	12.5	15	14	7
7304	28	14	10	51.5	18	20	8	94	8
7305	25	10	7	24	8	11	5	11	14
7306	21	9	10.5	34	11	11	10	13	5
7307	18	8	5	31.5	8	10	6	12	7
7308	20	8	7	39	10	10	7	12	6
7309	27.5	11	20	53	16	15	21	36	9
7310	29	9	8	16	14	10	15	9	18
7311	69	14	12	37.5	19	13.5	9	31	7
7312	44	11	9	65.5	19	13	13	19	14
7313	23	9	7	21	13	10	29.5	9	9
7314	24	8	9	16	11	10	6	13	4
7315	46	16	17	42	10.5	17	7	17	23.5
7316	35	11	17	85	18	14	52	47	48
7317	701	545	29	54	57	2309	22	73	632
7318	450	9	9	19	11	11	9	14	17
7319	30.5	10	10	17	14	11	9	15	4
7320	29	8	8	47	10	10	9	19	6
7321	21	7	5.5	20	8	10	5	9	4
7322	46	14	9.5	48	12	21	10	164	5
7323	23.5	10	7	43.5	12.5	11	8	28	49
7324	156	26	21	34	31	21	11	21.5	56
7325	38	8	7	26	155	12	8	20	19
7326	49	9.5	6	23	1335.5	10	8	14	19
7327	14	6	5.5	8	6	8	5	6	2
7328	24	8	5.5	24	10	11	7	14	6
7329	43	9	9	17.5	14	10	7	12	5
7330	30	10	10	26	14	14	39	16	65

7331	24	7	8	23	17	12	7	15	19
7332	34	10	23	31	33.5	12	12	24	59
7333	28	12	29	39	16	16	8	52	21
7334	27	9	7	25	10	10	10	58	4
7335	26	7	7	22	10	8	6	11	15
7336	29	11	7	33	12	12	8	16	99
7337	59	33.5	12	35	18	26	11	26	26
7338	34	18	11	31	14	18	12.5	23	19
7339	50.5	27	14.5	37	15	29.5	14	29	23
7340	25	11	14	30.5	11	15	9	17	23
7341	26	10	8	31	10	12.5	7	15	11
7342	44	17	10.5	38	14	15	9	18	16.5
7343	34	16	10	38	15	15	9	18	19
7344	58	34	20	44	16	38	13	35	46.5
7345	58	39	24	48	19	42	17	50.5	49
7346	38	22	8	33	13	22	8	19	31
7347	31	13	10	30	15	16	7	19.5	18
7348	43	24	11	43.5	15	26	11	26	22
7349	24	8	6	28	9	11	5	15	6
7350	59	35.5	16	39	20	39	34	36	44
7351	36	13	10	45	14	16	8	25.5	8
7352	25	10	6	30	7	9.5	5	13	7
7353	69	41	18	48	233	45	17	46	39.5
7354	54	31	16	40	15	34	13	31	27
7355	24	11	6	30.5	9	12	7	15	17
7356	35	23	10	39	12	22	8	21	23
7357	30	18	10	30	13	18	8	20.5	21
7358	46	24	12.5	31	15	24	16	23	23
7359	23	8	8	33.5	8	10	8	12	62
7360	32	21	10	32	16	32	8	14	18
7361	22.5	10	7	29	9	9	6	11	22
7362	99	68	35	67	34	65	26	60	83.5
7363	49	26.5	16.5	46.5	19	29	15	36	32
7364	31.5	18	9	37	14	19	9.5	23	24
7365	74	51	23	67	22	49	19	52	46
7366	21	9	7	36	9	10	8	18	7
7367	26	19	11	56	15	29.5	6	16	25
7368	32	15	9	39	8.5	18	7	18	38
7369	34.5	18	8	37.5	9	19	7	17	35
7370	39	11	9	41.5	16	13	8	18	18
7371	28	11	9	30.5	12	14	9	17	20
7372	24	14	7	38	17	14	7	18	18
7373	29	14	9	36	14	15	7	16	38
7374	25.5	12	7	30	11	15	6	16.5	20
7375	22	15	6	29	13	16	7	15	78
7376	37	25	9	45	13	25.5	8	18	40
7377	60	33	20	53	21	34	17	43	61.5

7378	57.5	33	19	45	19	35	16.5	39	58
7379	31	15	10	32	14	16	8	19	17
7380	52.5	30	32	54	26	31	14	36	28
7381	40.5	19.5	10	44.5	12	20.5	10	33	20
7382	73	48	20.5	55	23	50	21	54	81
7383	42	11	15.5	46	18	13	9	17.5	19
7384	38	43.5	21	71	24	46	16	30.5	46.5
7385	127	53	44	193.5	29.5	64	76	433	48
7386	39.5	36.5	21	70	29	47	14	28	46
7387	37	49	27	84	28	51	18.5	36	49
7388	63	44	249	108.5	42	59	16	27	45
7389	89.5	85	62	138.5	38	76.5	32	69.5	86
7390	51.5	49	29	79.5	32	53	25	47.5	38.5
7391	39.5	41	18	68	22	38	15	26	32
7392	43	46	25	74	29	53	20	34	50
7393	25	27	20	58.5	15	33.5	10	14	35
7394	80.5	50	20	71	21	39	10	23	23
7395	41	38	11	28	15.5	53	7	12	24
7396	30	19	35	122	21	23.5	10.5	26.5	56
7397	21	26	9	22	13.5	36	13	11	15
7398	54.5	47	23.5	67	28	60	16	35	44
7399	33.5	32	15	39	23	39	9	16	26
7400	26	25.5	13	42.5	19	33.5	7	14	33
7401	48	45	21	92	25	43	14	31	60
7402	30	25	12	80	20	38	10	16	21
7403	27	26	9	32	16	35.5	6	10	16
7404	60	30	36	77	31	39	15	49	38
7405	66	27	13	46	21.5	37	10	20	28
7406	23	27	12	34	25	39	8	12	17
7407	26	25	12	41	17	34.5	7	12.5	18
7408	39.5	29	14	82	29	41	11	30	79.5
7409	38	33	20.5	78	22.5	42	14	28.5	31
7410	27	28	15	48.5	14	35.5	8	21	19
7411	81	34	23	63	34.5	47	14	68	33
7412	43	43	23	59	22	45.5	20	36	43
7413	60	89.5	39	86	40.5	69	31	68	85
7414	35	43	22	68	24	43	16	37	51
7415	99.5	36	18	57.5	19	44.5	13	25	25.5
7416	32	28	14	68.5	17	34	9	19	43
7417	26	24	12	50	18	38	10	14	22
7418	84	73	31	75	24	75	23	43	61
7419	30	27	20	47	16	36	9	16	33
7420	25	25	13	69.5	13	34	10.5	19	52
7421	32	24	14.5	74	19	35	8	19	51
7422	57	50	23	82.5	29	47	18	38	53
7423	36	26	15	57	17	32	9	20	23.5
7424	32	26	15	61	17.5	39.5	12	21	29

7425	49.5	42	22	72	23	40	18.5	35	59
7426	32	33	19	80	18	42.5	14	28.5	30
7427	22	20	12	56	13	28	6	15	15.5
7428	38.5	56	39	69	26	48	16	39	50
7429	28	36.5	18	71	17	34	14	25	52
7430	41.5	27	16	55	22.5	34	11	34	22
7431	29	22	21	74	15	32	8	19	26
7432	41	38	23	59.5	16.5	41.5	14	28	44
7433	35	23	14	67	14	37.5	8	16	33.5
7434	29	24	17	55	16	37	8	28	29
7435	30	38	20	102	22	36	20	33	34
7436	31	29	18	57	18.5	36	11	23	27
7437	27	22	14	48.5	24	31	8	19	29
7438	32	35	18	62	20	36	14	25	34
7439	27	27	15	48	17	38	10	19	25
7440	39	28	22	67	21	26.5	15	25	33
7441	36	46.5	24	68	24	43	17	35	47.5
7442	30	32	18	62	18	35	13	26.5	35
7443	33.5	42	20	62	22	39	13	27	37
7444	43	57	27	78	36	56	23.5	55	70
7445	40	41.5	22	66	20	44	14	29	33
7446	29	26	15	68	16.5	33	10	27	29
7447	36.5	53	24.5	58	25	34	23	42	59.5
7448	37.5	46	21	69	22	38	16	41	49
7449	38.5	25	14	60.5	16	45	8	16	23
7450	59.5	66	38	97.5	35.5	57	28	58	77
7451	28	22	15	88	14	32	9.5	21	32.5
7452	29	26	16	57.5	18	39	13.5	19	19.5
7453	24	22	11	64	17	30	8	39	15
7454	28	23	20	47	15	30.5	6	15	19
7455	22	25.5	11	48	14	31	9	14	14
7456	43.5	23	20.5	35	22.5	31	10	58	27
7457	28.5	24	16	113	22	33.5	12	23	27
7458	29	26	15	108	28.5	35	17	20.5	36.5
7459	27	23.5	15	76	17.5	33	8	18	16.5
7460	34	18	9	49	13	25	7	12	15
7461	30.5	23	14	53	17	33	6	13	14.5
7462	28.5	18	7	22	18	25	7	11	10
7463	22	23	10	56	14	30	7	17	36
7464	25	23	18	81	16.5	32	8	20	20
7465	28	20	12	66	15	31	7	14	18
7466	26	21	10	37	33.5	33	9.5	32	14
7467	25	22.5	9	40.5	17	30.5	11	13	14
7468	24	24	11	31	17	31	18	19	17
7469	76.5	30	17	45	22.5	40	9	14	37.5
7470	131	106	48	80.5	61	114.5	37	99.5	95.5
7471	77	55	20	48	26	69	15.5	46	51

7472	114	75	28	61	40.5	83	25	69	70
7473	98.5	76	36	66	59.5	85.5	27.5	88	83.5
7474	176.5	51	22	64.5	29	65	19	54	57
7475	69	57	18.5	52	27	69	19	61	48
7476	128	85	37	80	48	91	30	94.5	59
7477	129	45	16	52	32	58	15	46	39
7478	65	32	10	52	50	33.5	8	20	69.5
7479	96	69.5	26	67	40.5	75.5	22	56	78
7480	100	84.5	32	80	178	89	28	82	67
7481	85	33	9	41	17	40	8	38	32
7482	46	34	15	50	38	39	10	29.5	77
7483	90	71	28	64.5	44	82	22	64	56
7484	47	34	13	44	29	43	11	32	26
7485	203	168.5	81	113.5	61	181	76	155	146
7486	40.5	32	14	47	31	35	10	26	28
7487	79	51	26	56.5	36.5	53	17	40	63.5
7488	65.5	45.5	26	48	24.5	53	13	54	21
7489	140	113	51.5	98	50	121.5	41	128	102
7490	49.5	31.5	12	53	27	41	10	38	19
7491	50	33	13	49	27	42	10	30	21
7492	93	64	28	62.5	98	65.5	29	58	90
7493	70	50	27	62	31	57.5	14	60	49
7494	94	65	30	70	32	81	20	64	69
7495	51.5	30	19	42	33	39	21	24.5	23
7496	60	37	15	44	26	43	15	36.5	35
7497	41	30	14	38	26	35.5	8	22	25
7498	44	32.5	10	38	20	39	9	21	18
7499	84	59	21	56	51	66	19	46	67
7500	34	17	9	30.5	20	21	7	21	24
7501	57	41	12	50	28	47	14	29	69
7502	74	61.5	25	78.5	40	66	19	52.5	69
7503	77.5	60	26	57	32	68.5	19	50	100
7504	78.5	53	25	58.5	47	56.5	20	50	64.5
7505	110.5	80	33	78.5	48	85	29	70	68
7506	94	73	37	67	44	74	24	69	58
7507	72	89	22	51	45	59	17	46	60
7508	76.5	25	8	22	23	30	6	17	4
7509	37	25.5	10	21	23	35	8	33	4
7510	68	44	16	38.5	69	54	15	274	11
7511	42	24	9	27	14	33	6	17	6
7512	30	29	7	26	14	32	7	39	6
7513	34.5	23	7	22	16	29.5	6	16.5	8
7514	42	21	12	23	51	28	19.5	18	7
7515	59	25	53	23	445	31	18	24	10
7516	51	20	10	19	28	29	9	13	52
7517	157	35	21	44	127	39	73	36	27
7518	117	26	10	23	24	32.5	21	26	20

7519	245	53	18.5	138.5	69.5	47	22	115	28
7520	38	24	13	27	27	34	13	18	13
7521	26	15	6.5	13	15	18	5	10	6
7522	28	22	6	17	13	30	6	11	4
7523	32	23	8	29.5	53	31	7	13	4
7524	27	18	7	13	13	26	6	11	4
7525	28	21	8	18	14	27	6	14	4
7526	29	25	10	17.5	19	32	12	17	11.5
7527	96	26	17	18	23	33	26	18	72
7528	33	23	7	17	50	34.5	6	16	3
7529	97	27	13	27	24.5	30	21	20.5	82.5
7530	59	26	27	45	48	32	53	20.5	35
7531	64	35.5	21	118	290	53	218	456	55
7532	71	26.5	27	54	617	34	29	23	15
7533	53.5	30	76	92	78	44	86	113	13
7534	48	27	11	53.5	23	35	13	31	6
7535	46.5	21	13	36	13.5	23	10	17	35
7536	38	24.5	9	39	14	34	19	19	13.5
7537	37	23	8	57	22	31	9	38.5	12
7538	50	26	10	100	27.5	34	22	95	15
7539	40	23	8	62	43	29	43	35	11
7540	70	26	17.5	79	23	39	18	96.5	61
7541	646.5	338.5	83	180.5	134	293	91	227	264.5
7542	31	21	9	24	23.5	26	6	15	10
7543	36	25	19	66	24	32	12.5	24	17.5
7544	46	28	10	44	15	35	11	26	138
7545	27	21.5	6	21	18	27	5.5	13	9
7546	45	35.5	14	191	20	50	24	117	20
7547	44	26	9	58	28	52	10	94	16
7548	53	25	13	91	106	38	15	93.5	217
7549	113	23	17	76	816	30	12	61.5	51
7550	52	29	9	59	29.5	38	11	25	11
7551	50.5	61	11	37.5	21	28.5	18	21	127
7552	135	25	14	68	48	28	16	26	91
7553	74	35	15	71	83	37	21	90	51
7554	31	24	8	28	702.5	33	11	105	7
7555	34	29.5	6	9	11	36	6	14.5	2
7556	62	37	7	13	589	74	7	22	17
7557	66	46	22	51.5	28	53	23	49.5	44
7558	48	30	14	33	25	35	11.5	24.5	24
7559	49	32	12	40	22	37	12	26	30.5
7560	50	29.5	12	35	29	35	12	23.5	23
7561	49	35	33	50	24	40	17	38	26
7562	70	36	15	44	21	40	13	33.5	55
7563	411.5	56	26	68	35.5	61	21	53	64
7564	68	33	13.5	50	21.5	40	14	33	38
7565	63	46	18	42	27	48	15	56	29

7566	37	20	9	43	20	27.5	7	22.5	22
7567	54	24	12	40	24	26	7	21	39
7568	48	36.5	13	41	20	39	12	31	32
7569	42.5	25	9	38	20	33	10.5	27	29.5
7570	65	36	15	54	29	41	23	35	28
7571	56	39	22	87	22	45	79.5	56	47
7572	87	64	25	61.5	37	80.5	23	54	74
7573	66	47	18	48	25	54	17	45	61
7574	66	44	21	51	26.5	44.5	17	38.5	40.5
7575	97	64	28	68	42	65	25	81	55
7576	43	27	13	45	20.5	36	11	46	42
7577	73	30	13	44	52.5	37	12	28	24
7578	50	33	14	49	27	35	15	31	32
7579	49	26	30	52	72	29	40	32	17
7580	43	23	8	34	19	27	7	20.5	12
7581	48	28	10	47.5	26	34	12	24	39
7582	36	16	8	39	15	22	9	15	33
7583	34	20	9	32.5	21	24	8	20	20
7584	34	20	9	37	22	24	8	21	35
7585	37	21	10	52	19	26	8	19	28
7586	35	24.5	13	40	18	29	9	22	19
7587	48	30	15	40	21	34	11	26	33.5
7588	67	47	22	54	29	54.5	16	55	43
7589	84	59	36	69.5	41	65	21	53	60
7590	53	32	23	57	24	34.5	18	41	24
7591	75	57	31	70	33	64	22	54	80
7592	93.5	77	32.5	72.5	35	92	25	80	92
7593	89	78	30	63.5	32	78	29	59	89
7594	75	51	23	62	30	64	18	57	73
7595	66	42	33	66	28	50	24	40.5	73
7596	79	51.5	20	58	31	57	20	67	50.5
7597	54.5	42	19	45	27	48	19	44.5	41.5
7598	66	50	27	64	34	53.5	21	48	51
7599	68	44.5	24	67	34	50	19.5	47	48
7600	46	29	13	54.5	19	37	12	29	32
7601	69	46	18	55	31	50	22	39.5	67
7602	47	30	18	42	24	40	15	31	30
7603	86	40	18	67	34	47	14	41	67
7604	51	35	17	44	22	40	11	39	35
7605	93	60	27	62	30	65	67	53	61
7606	60.5	42	15	50	28	44	15	38	31
7607	35	21	8	36	24	24.5	11	20	22
7608	50	37	15	43	23	39.5	14	40.5	29
7609	48	29	18	130	21.5	34	12	40.5	41
7610	64	29	13	42.5	22	34	11	30.5	18
7611	46	22	11	46	19.5	32	9	24	28
7612	49.5	26	12	44	17	33	8	24	23

7613	60	48	33	100	32	57	21	40	53.5
7614	77	56	25	87	30	82	15	33	59
7615	75.5	52	21	88	34.5	67	15	30	73.5
7616	50.5	77	41	105	44.5	63	28	63	94
7617	60	63	35	120.5	39	54	24	50	70
7618	60.5	68	32	90	55.5	67	24	50	101
7619	41	56.5	27	79	42	50	20.5	41	72
7620	216	27	18.5	66	29.5	51	24	22	76
7621	35	45	28	73.5	46	46	17	33	59
7622	78.5	97	46	154	54	77	36	76.5	79
7623	65	55.5	26	114.5	34.5	62	21	45	49
7624	39.5	49	26	85	35	49	18	65	52
7625	40.5	42	22	73	33	51	15	33	44.5
7626	111	71	36	104	39	86	28	52	70
7627	41	56	28	93.5	30.5	50	19	36	69
7628	47	26	17	87	35.5	35	12	24.5	38
7629	32.5	43	21	71.5	40	42	14	30	41
7630	77	46	24	88.5	25.5	46	23	38	56
7631	40	53.5	24.5	76	26	54.5	18	34	51
7632	57	58	22	88	27.5	53	20	39	67
7633	62	53	41.5	85.5	35.5	48	23	40	53
7634	48	37.5	18	65	25	54.5	13	26	45
7635	35.5	46	26	86.5	26	47	18	39	52
7636	34	24	13	65	25.5	32.5	10	16	26
7637	71.5	54.5	39	99	50.5	52	23	50.5	62
7638	56	56	25	81.5	28	68	18	37	51
7639	49	51	52	98.5	33	54.5	22	39	51
7640	37	34	21	72	27	41	12	25	30
7641	36.5	41	22	75	24	43	15	37	43
7642	47	75	32	106	45	59	27	59	74
7643	15	18	4	11	8	23	3	7	9
7644	13	18	5	12	7	22.5	5	8	9
7645	29.5	26	19	56	24	35	15	52	15
7646	25	21	7	44	17.5	31	5	11	13
7647	24	22	69	40.5	16	27	6	12	13
7648	58	26	22	50	29	41.5	15	20	26
7649	42.5	31	22	60.5	35.5	37	24	29	134
7650	34	21	22	106	25	31	36	34.5	33
7651	20.5	16	14.5	65	27	26	10	17	16
7652	34	20	19	76	61.5	34	17	19	23
7653	82.5	21.5	12	45	31.5	34	9	18	20
7654	52	27	28	127	24	44	11	33	20.5
7655	67	72	30	95.5	48	84	23	48	79.5
7656	100	42	20	68	26	63	16	29	34
7657	87	90.5	44	105	45.5	91	35	72	86
7658	57	49	26	154	33.5	63	15	36	59
7659	80	69	34	100	42	97	25	47	69

7660	64	42	24.5	64	40.5	62	16	39	34
7661	81.5	43	22	75	29	90	13	31	78
7662	63	55.5	27	75	48	68.5	20	32	50
7663	265	50	30	102	34	88	17	34	50
7664	53	38	22.5	90	27	58	13	25	45
7665	203.5	51	37	99	64	75.5	34	81	74
7666	53	41	27	89	28	62	14	29	33
7667	41	32.5	22	78	22	54.5	11	21	34
7668	46	37	16	58	26	59.5	10	18	35
7669	63.5	63	30	77	37	76	21	41	66
7670	113	45	20	73	28	59.5	15	27	57
7671	61	40	26	69.5	37	58.5	16	24	53
7672	74	47	27	88	115	75	16	30	42
7673	51.5	40	23	83	26	56.5	15	25	52
7674	72	95	41	94	40.5	79	28	58	143
7675	69	75	43	115	50.5	78	33.5	58.5	78
7676	73.5	70	45.5	104.5	43	72	30	56.5	85
7677	63	60	32	106	40	64	25	56	104
7678	57.5	46	26	71	30	67	16	34	49
7679	100.5	51	27	101	35	100.5	16	47	56
7680	61	34	25	71	39	55	16	28	35
7681	307	37	23.5	80	40	101	11	26	47
7682	63.5	34	22.5	85	32	58	11	31	34
7683	95	56	33	88	41	77	17	36	78
7684	76	35.5	23	72	33	54	13	29	51
7685	57	55	28.5	100	30	68	17	39	103
7686	70	33	22	115.5	29	52	11	27	51
7687	52.5	31	22	92	31	51.5	14.5	17.5	44
7688	50	55	30	81	30	61	18	44.5	56
7689	60	42	21	88	28	57.5	14	32.5	56.5
7690	51	49.5	30	69	30	55	18	39	47
7691	31	28	7	13	10	47	6	9	15
7692	7	9	3	7	5	11	3	5	4
7693	42.5	42	12	35	28	47	20	16	21
7694	36	29	12	66	21	45	8	29	21
7695	46.5	26	15.5	64	30.5	45	45	20	35
7696	41.5	45	17	50.5	27	51	20.5	16	24.5
7697	359	46	36	150	42.5	115	21	40	92.5
7698	45	32	15	54.5	48	53	18	19	35
7699	95	28	18	25	39	45	11	66	28
7700	255	36	19	93	32	54	13	82	77
7701	50	41	16	34	37.5	52	11	25	30
7702	43.5	28	14	47	21	49	10	15	22
7703	39	27	15	63	23.5	46	10	20	29
7704	54	27	13	46	26	44.5	7	18	18
7705	37.5	30	30	56	30	40.5	12	21	32
7706	39.5	31	21	50	35.5	44	21.5	28	22

7707	40.5	30.5	13	43	27	51	8	14	30
7708	62.5	31	24.5	109.5	46	51	19	31	29
7709	39	27	12	30	96	44	11	16	23
7710	41	28.5	16.5	38	24.5	50	55	15	28
7711	167	32	15	35	36	50	12	19	27
7712	74	31	16	44	26	49	11	25	25
7713	83	62	43	106.5	60	79	45	60	58
7714	44	32	17	63	84.5	48.5	12	52	99
7715	44.5	26.5	40.5	96.5	27	47	12	21	22
7716	43	25	22	78.5	94.5	44	9	20	27
7717	42	32	15	51	50	48	18	15	22.5
7718	38	28.5	18	75	37	47	9	32	59
7719	39	28	25	48	22	44	12	16	31
7720	332	31	17	85	26	87	11	22	46
7721	53	30	16	72	27	45	66	44.5	23.5
7722	69	35	14	24	28	55	9	14	80
7723	34.5	28	9	18	18	42	11	13	16
7724	59.5	39	36	99	45	59	22	40	40
7725	60	34	16	30	43	50	12.5	15	29.5
7726	58	39	21.5	215.5	26	55.5	20.5	34	59
7727	49	34	40	38	40.5	58	18	20	33
7728	46	26	16	31	36	46	21	18.5	27
7729	49	29.5	15	52	34.5	43	10	17.5	81
7730	49	25.5	14	46.5	43.5	52	12.5	18	24
7731	36.5	32	15	78	20	44	9	19	89
7732	55.5	40.5	15	58	19.5	66	9	16	84
7733	35	26	16	76	19	46	11	15	58
7734	37.5	31	15	52.5	20	45	10	17	44
7735	43	32	19	76.5	20	47	10	20	153
7736	41	27	15	70	22	41.5	10	18	70
7737	44.5	32	24	71	27	52	21	17	50
7738	60	25	13	30	25	37	19.5	66	16
7739	48.5	32	19.5	98	23	51.5	13	32	79.5
7740	50	28	16	80	104.5	46	14	26.5	21
7741	41.5	28	14	77.5	24	48.5	10	67.5	46
7742	53	31	25	68	52	45	12	68	44
7743	57	29	17	41	51	45	13.5	21	24.5
7744	45	32.5	21	64.5	29	46	11	21	53
7745	44	42	22	71	29	48	21	34	53
7746	151	66	35.5	88	41	65	29	57	75
7747	74.5	55	28	80	37	58	21	43	58
7748	40	28	19	66.5	23	46	12	20	104
7749	103	94.5	32	122	36.5	91	25	64.5	78
7750	52.5	41	24	68	23	50	16	33	36
7751	55.5	33	21	63	24	50	14.5	33	56
7752	40	25	23	54	20	43	10	20	23
7753	36	24	15	76	21	40.5	9	23	47

7754	48	47	30	79	33	47	19.5	39	111
7755	51	57	29	89	32	58	23	46	70
7756	50	47	25	77	30	57.5	21	40	56.5
7757	55	45.5	25	77	29	59	16.5	33	69
7758	62	59	29	84	34	61	29	48	60
7759	47	51	27	71	24	58	19	40	52
7760	53.5	62	29	74.5	37.5	57	24	49	70
7761	43	23	15	61.5	22	42	7	20	48
7762	46	38	18	58	22	49.5	13	29	36.5
7763	38	25.5	15.5	58	20	40.5	10	20	24
7764	113.5	34.5	20	70.5	34	62	13	23.5	42
7765	46	29	16	61	25	47	9.5	18	26
7766	49	43	24	88	95	54	15	35	50
7767	44	34	22	57	48	44	15	28.5	44
7768	39	23	16	61.5	21	40	7	20	22
7769	50.5	44	26	133.5	38	53	18	40	64
7770	38.5	25	13	46	23	40	9	18	23
7771	72	32	30	63	42	74	11	25	55
7772	47.5	25	16	52	22.5	40	8	20.5	30
7773	42	55.5	27	58.5	28	53	20	44	50
7774	68	62	32	129	36.5	64	25	49	65
7775	57	93	45.5	89	67	73	33	68.5	94
7776	38	24	16	119	20	42	9	21	29
7777	36	36.5	21	62	28	41	15	35	33
7778	148	26	18	52	21	42	12	24	21
7779	49	46	26	83	31	52	18	40	40
7780	55	48	29	75.5	33	55.5	19	41.5	47
7781	72	88.5	48	122	41	72.5	36	77	93.5
7782	115	37	19	66	29	49	15	34	58
7783	55	44	21	76	29	58	14	33	86
7784	42	40	15	47	22	54	12	25	63
7785	41	32	17.5	75	22	38	11	31	37
7786	56	57	40	84	29.5	57.5	24	49	70
7787	57.5	27.5	29.5	114	26	43	23	36	22
7788	45	26	22	90	34	42	18	26	50
7789	63	26	18	42	25	48.5	10	23	26
7790	41.5	28	17	26	24	44.5	10	16	24
7791	37.5	22	9	25	16	36	7	10	16
7792	31	22	11	23	20	39	7	12	14
7793	42	26	19	68	24	42.5	16	27	31
7794	31.5	22.5	13	30	26.5	42.5	8	14.5	13.5
7795	31	21	9	28.5	17.5	35	8	14	14
7796	40.5	25	17	52.5	25	42.5	10	20	23
7797	42	27.5	16	33	22	41	11	162	24
7798	50	37.5	25	41	31.5	60.5	15	110	24
7799	44	31	14	61.5	23	51	9	52	20
7800	47	43	22.5	88	42	53.5	19	36	164

7801	36	31.5	13	32	29	44.5	8	20	22
7802	83.5	32	25	83	34	48	21	39	270
7803	34	29	22	73	37.5	42	13	22	57
7804	30	27	10	28.5	26	42	8	13	21
7805	35	29	51	83	23	46	14	20	19
7806	28.5	25	13	57	20	34	10	14	49
7807	32.5	27	11	42.5	27	40	9	12	21
7808	31	30	12	28	25	40	11	11	18
7809	48	34	29	150.5	29	46	17	40	43
7810	58.5	31	29	33	28	46.5	10	17	21
7811	30	25.5	9	29	26	33	6	12	19
7812	60	33	15	71.5	20	62.5	10	18	25
7813	41	43.5	21	69.5	25	45	14	34	31
7814	39	35	19	70.5	19	43	12.5	24	31
7815	32	38	16	55	23	44	13	25	30
7816	51	61	30	81	32	59	24	40	86
7817	40.5	60	29	73	37	56	23	51	94
7818	169	77	38.5	69	34.5	75	30	60	79
7819	53	54	27	72	25	72	17.5	45	46
7820	38	35	21	55	24	42	15	25	29
7821	40	54	28	76	31.5	49	19.5	38	138
7822	45	54.5	29	89	26.5	51	22	40	44
7823	40	64	32.5	70.5	26	55	21	47	68
7824	47.5	66	30	87	30	51	23	46	58
7825	35	34	18	59	20	38.5	11	22	29
7826	41	57	29	63	32	51	24	37	50.5
7827	38.5	54	25	73	31	55	18	39	75
7828	47	71	31	83	34	60.5	26	58	73
7829	50	66	35	90	39	59	27	55	69
7830	50.5	48	21	60	23	59	15	35	39.5
7831	45	52	29	69	37	53.5	20	40	49
7832	47	47	24	71	27	55.5	20	37	44
7833	69	77	32.5	85	32	78	25	50	60
7834	396.5	65	35	97.5	39	100	23	54	76
7835	1164	75	32	82	34	367	20	45	91
7836	31	35	21	64	23	40	14	27	36
7837	56.5	86.5	44	117	40	66.5	35	68.5	98
7838	41	41	22	72	28.5	54	15	30	54.5
7839	39	48.5	23	62	27	47	17	36	44
7840	42	62	32	89	30	54	41	63	66
7841	44	64	32	105	33	58.5	25	49.5	74
7842	55	72	31	90	37	73	24	49	71
7843	42	65	29	83	29.5	49	21	40	65
7844	55	93	50	100	48	73	42	73	97
7845	59	96	49	113	41.5	72	39	75	86
7846	32	47	23.5	71	25	41	16	36	61
7847	38	47.5	26	73	32	46	23	38.5	43

7848	25	20	15	54	18.5	32	14	19	17
7849	29	23	11	37	17	33	7	14	27
7850	24	24	14	87	23	34.5	7	23	16
7851	24	21	14	56	21	31	7	15	26
7852	24	20	10	46.5	14	32	10	17	18.5
7853	42	25	17	115	23	32	13	77	17
7854	71	19	11	26	24	28	8	13	17
7855	28	23	11	27	20.5	43	9	16	32.5
7856	34	25	18	69	26	39	11.5	24	57.5
7857	69	32	34	76	41	40	22	45.5	53
7858	46.5	34	25	90	42	41	13.5	44	69
7859	45.5	23	21	81	27	34	18	36	77
7860	389.5	93.5	25	104.5	33	351	35	48	137.5
7861	23	22	12	39	22	31	8	16	31
7862	37	26	21	68	38	34	11	27	33
7863	32	24	16	70	23	35.5	50	19	31
7864	36	21	16	66	83.5	39.5	11	25	24
7865	25.5	20	17	61	20	32	9	20	37
7866	179.5	52	45.5	126	42	62	32	129	69.5
7867	25	22	17	58	17	29.5	9	19	33
7868	26	19.5	14	55	17	33	6	14	15
7869	24.5	19	12	65	24	30	7.5	14.5	25
7870	35.5	28	20	83	23	37	10	25	27
7871	29	22	14	73	31	35	12	23	28
7872	22	18.5	18	63	18.5	30	7	16	25
7873	31	23	15	88	17	31	8	22	18
7874	225	22	14	64.5	18	65	7	17	34
7875	27	21	11	56	16	34	7.5	16	29.5
7876	34.5	27	17	60	23.5	38	10	25.5	30
7877	25	25.5	14	53	45	29	12	16	16
7878	25	20	15	71	17	30	7.5	16	18
7879	40	18	16	59.5	17	35	7	24	24.5
7880	26.5	20	20	67	21	34	9	19	32
7881	25	18	13	62	22	26.5	7.5	15	16
7882	34	21	14	49	26	34	9	19	18.5
7883	17	10	9	27	18	12	5	11	12
7884	17	10	5	13	15	15	6	8	8
7885	15.5	10	5	9	14	13	6	8	6
7886	33	29	12	32	26	35.5	6.5	12	15
7887	77.5	9	6	18	15	21	5	10	9
7888	56	74	42	89	32.5	73	25	65	72.5
7889	77	100	38	119	36	84	37	73	70
7890	62	74	36	121	38	63	27.5	54	64
7891	49	36	22	102	23	49	12	26	44
7892	54	52	27	94	26	53	17	39	73
7893	60	35	26	91	27.5	50.5	15	27.5	52
7894	156.5	59	28	72	31	68	22	46	56

7895	79.5	54	25	96	35	66	19	39	60
7896	58	71	32	99	30	68	26	54	85
7897	76	37.5	22	117.5	27	55.5	12	28	30
7898	45	44.5	28	101	25.5	50	17	43	45
7899	53	53	29.5	128	42.5	56	33	43	67.5
7900	46	34	16	69.5	28	44	11.5	20	26
7901	179	67	32	102	30	74	23	53	128
7902	48	52	32.5	110	100.5	59.5	20	64	104
7903	72	71	32	102	35	77.5	93.5	54	74
7904	65	63	30	104	34	62	23	48	75
7905	42.5	37	19.5	79	27	45	14	24	33
7906	45	38	24	95	156	47	15	35	60.5
7907	81.5	61	18.5	77	28.5	100.5	12	29	58
7908	60	61	33	112.5	37	69	26	51.5	71
7909	68	74	42	122.5	42.5	69	33.5	66	70
7910	53	66	33	92.5	37	66	25	50	64
7911	78	47	29	94	36	69	19.5	53.5	54
7912	64.5	90	46	118	41	70	45	70	92
7913	73	130	39	120	44	84	32	59	229
7914	52.5	61	26	84	40	59	21	41	52.5
7915	90	95	52	133	51	96	37	73	97
7916	47	51	28	101	33	54.5	20	40	50
7917	68	72	35.5	117	33.5	70	25.5	60	73
7918	42	44	26	98.5	23	46	17	38	70
7919	46.5	44	25	96.5	27	50.5	17	50	60
7920	41	33	25	75.5	29.5	45.5	14	30	38.5
7921	47	44	23	98	21	53.5	14	37	35
7922	34	26	10.5	62	15	40.5	8	13	18
7923	29	24	22	83	16	37	7.5	13	20
7924	31	28	9	29	16	39.5	8	15	20
7925	35	28	14	34	23	40.5	10	14	19
7926	37	30	14	46	23	43	12	22	24
7927	29	24	11	39.5	20	41	7	12	24
7928	89	26	17	72.5	31	47	13	21	22
7929	46	27	11	33.5	19.5	39	9	18	21
7930	33	24	8	47	17	39	7	13	16
7931	38	37	15	76	20	43	11	21	49.5
7932	31	25	10	62	19	39.5	5	15	18
7933	54.5	26	11	43	17	40	9	13	17
7934	41	30	27	73	32	49.5	21	35	50
7935	54	28	59	54	64	44	20	31	33
7936	63.5	43	29	69.5	30	39	13	30	78.5
7937	68	29	14	80	37.5	45	17	44	23
7938	37	30.5	15	81.5	18	43	13	21	68.5
7939	30	24	13	61	18	37	8	13	14
7940	28.5	28	11	66.5	17	42	8	14	19
7941	43	33	18	42	25	50	15	57.5	34

7942	29	22	10	44	18	33	7	14	14
7943	27	23	33	43	16	37	10	18	32
7944	50	29	20	108	49	40	17	30	139.5
7945	45	26	18	72	21	35	11	21.5	24
7946	35	18	14	55	24	29	29	20	50
7947	37	31	11	57	19	43	11	16	19
7948	21	23	6	15	9	37.5	6	10	13
7949	51	20	10	45	23	33	14	14	19
7950	36	26	16	60	27	36	13	26	19.5
7951	41.5	37	21	86	41.5	53	15	17	27
7952	56	28	25	77	25	44	15	25	28
7953	42	27	23	131	25	39	27	29	24
7954	30.5	25	35	61	20	39	8	14.5	15
7955	38.5	24	15	97	15	36	9	16	25
7956	46	28	29	132	26	46	16	33.5	38
7957	30	25	21	61	15	36	7	14	15
7958	29.5	26	10	41	16	33	9	18	41
7959	100.5	25	14	46	31	56	12	49.5	19
7960	36.5	24	17	44	21.5	36	13	41	18
7961	35	22	17	67	50.5	37	18	21	28
7962	33.5	28	23	79	19	40	11	17	34
7963	30	23	12	41	17	33	8	15	16
7964	31	24	12	41	20	35.5	9	16	16
7965	28	22	12	41	28	35	7	16	13
7966	33	21	12	51	18	34	8	16	18
7967	40.5	32	17	77.5	28.5	43.5	12	27	33
7968	36	29.5	22	120	34	39	12	29	29
7969	39	25	20	74	26	35	10	22	18
7970	46	26	31	105	169	36	11.5	35	32
7971	37	31	23	108	27	38	11	35	45
7972	38	31	20	84	26	39.5	12	30	45
7973	55	51.5	25	111	40.5	51	19.5	44	52
7974	43	35.5	18.5	92.5	22	40	12	29	35
7975	40.5	25	22	118	25	40.5	13	41	27
7976	46	33.5	23	114	27	39	13	37	41
7977	42	28	18	92	30	36	10	26	33
7978	44	29	22	112	121.5	43	12	34.5	31
7979	45	42	23	94.5	32	43	14	34	53
7980	52	38	32	119	37	41	16	43	77
7981	62.5	80	37	157.5	35	63	26	69	77
7982	43	27	20	99	72.5	39	11	53	113
7983	93.5	28	27	135	30	42.5	27	37	47
7984	58	24	17	56	24	43	16	35	21
7985	28	21	10	28	16	37	7	13.5	13
7986	33	25	25	161	13	34	10	31.5	19
7987	39.5	33	34	74	32	41	85	36	50
7988	36	24	16	78	23	39	10	21	41

7989	34	22.5	16	80	20	36	8	19	32
7990	45	54.5	30	85	33	49	19	51	90
7991	50	36	18	95	28	52.5	14	29.5	46
7992	48	38	22	99	27	46	16	49	67
7993	41	26	19	69	21	41	12	24	24.5
7994	39	53	26	73	32	36	32	56	64
7995	47	27	25	72	20	37	12.5	25	30
7996	53	27.5	17	79.5	30	55	9	19	30
7997	37.5	25	18	73	19	37	12.5	21.5	23.5
7998	36.5	25	19	84	20	38	11	24	26
7999	35	33	19	60	53.5	39	13	33	32
8000	46	24	35	98	25	37.5	28	28	47
8001	44	50	23	90	24	44	18	39	62.5
8002	61.5	47	29	134	30	49	19	58.5	76
8003	44	46	25	85.5	30	45	19	52.5	51
8004	46	37	22	94	22	44	16	36	35
8005	40	50	26	72	34	42	19	54	39
8006	30	24	16	92	27	34	9	25	21
8007	31	21.5	14	90	17	33	8	21	40
8008	52.5	46	25	134.5	64	54	19	44.5	43
8009	59	31.5	17	99	24	31	18	41	36
8010	42	36	25	95	23	41	15	40	50
8011	305.5	41	19	96.5	20.5	41.5	15	36	44
8012	29	21	16	84	18	33	9	21	20
8013	42.5	38	19	63	25	43.5	16	30	32
8014	45	38	18.5	81	21.5	46	14	42	42.5
8015	31	21	12	54	16	34	8	21	42.5
8016	30	22	15	70	14	36	7	18	25
8017	26	17	14	60	52	20	10	29	13
8018	32	22	13	92	18	35	9	20	15
8019	38	30	17	83	20	37	11	33	26
8020	41	54	29	104	27	38	25	60	98
8021	33	26	16	78	28.5	27.5	11	26	23
8022	64	20	15.5	85.5	20	31	10	27	30
8023	31	20	12.5	52	21	30.5	9	18	21
8024	38	22	15	79	20	33	13	26	37
8025	29.5	21	18	86	18	20	11	32	29
8026	137	29	17	74.5	25	42	12	24	24
8027	44	40.5	22	86	34	46	20	55	42
8028	35	24	14	73	19	35	10	25	19.5
8029	47	55.5	24	82	26	44	19	58	59
8030	29	15	13.5	73	16	21	10	25	16
8031	37	18	12	68	30	33	9	17.5	63
8032	33	26	18	103	22	27	36	32	30
8033	53	25	15	77	24	34	10	22	30
8034	48	35	21	135	24	39	13	30	29
8035	43	20	15	55	21	31	11	20.5	25

8036	32	16	11	57.5	18	25	14	20	26
8037	35.5	22	13	65	16	34.5	9	29	17
8038	48	20	14	83	19	33	27	32	26
8039	48	67	32	121	30	55.5	28	60	92
8040	55	66	31.5	92	35	46	31	61	74
8041	42	37	22	78	25	35	15	39	52.5
8042	64	36	21	93	26	53	13	30	40
8043	74	99.5	49	131	49.5	84	32	77	84
8044	47	41	22	83	22.5	64	13	27	56
8045	28	27.5	13	40	23.5	41	9	15	19
8046	92	43.5	25	69	79.5	83	21	37	49
8047	42	37	16	47.5	42	54	12	32	26
8048	56	33	23	67	41	44	21.5	33	26.5
8049	55	36	33	142	36.5	56	14	32	42
8050	48.5	37	27.5	51.5	29	52	11	23	26
8051	83	36	19	50	36	49	23	37.5	23
8052	33	31	14	49	28	45.5	8	17	22
8053	43	30	16	55	27.5	45.5	16	18	20
8054	114	34	19	51	36	50	13	32	33
8055	47	60	79	103	29	50	24	54	65
8056	34.5	32	13	52	21	46.5	9	20	20.5
8057	51	32	15	55	26	48	11	26	27
8058	35	30	17	76	872.5	38	8.5	17	21
8059	45	35	21	94	37	50	15	37	40
8060	41	31	32	101	29	49	12	26	26
8061	34	29	20	63.5	24	44	12	20.5	37.5
8062	37	37	20	69	25	48	12	25	80.5
8063	34	28	18	97.5	19	41.5	10	20	24
8064	54	77.5	43	99	37	63	32	66	75.5
8065	78	53.5	36	89.5	35.5	61	18	43	56
8066	44	34	21.5	99	79.5	47	12	29	34
8067	36.5	28	15	73	24	42	11	45	32
8068	34.5	29	17	77	22.5	42	10	22	25
8069	46.5	51	23	87	28	53	17	36	48
8070	52.5	36	16	77	26.5	48	11.5	37	30.5
8071	39	56	28	103	33	54	18	43.5	59
8072	40	36	18	82.5	25.5	48	12	24	29
8073	38	37	21	105	26	47	13	29	37
8074	39	47	23	107	28	52	15	36	47
8075	50	45	22	105	48	54	15	32	41
8076	47	74	33	114	35.5	60	25	67	62
8077	38	25	15	63.5	25	37	10	20	33
8078	45.5	41	23.5	81	29	54	14	35	33
8079	36	37	24	94.5	30	40	15	31	52
8080	36	34.5	24	69	27	47	14	26	47
8081	48	53.5	29	84	42.5	55	25	58	55
8082	32.5	30	20	83	40.5	41.5	10	23	74

8083	41.5	53	25	90	27.5	52	19	35.5	51
8084	51.5	49	27	103.5	31	50	18	44	47
8085	38	51	27	90	28	51	21	48	59
8086	39	41	25.5	94	26.5	48	13	32	37
8087	33.5	35.5	19	70	32	41	14	29	39
8088	39.5	42.5	25	71	27	43	17	31	40
8089	34	33	17	73.5	26	44	9	22	54
8090	51	58	27.5	117	28	57	20	48	88
8091	36.5	33.5	17	60.5	23	44	16	23	45
8092	27	41	31	73	20	41	13.5	20	92
8093	31	35	15	90	25	39	9	21	26
8094	41	26	18	92	37	40	17	27	35
8095	35	31	22	86.5	35	46	15	24	33
8096	75	33	34	74	36	43	16	30	28
8097	39	36	21	72.5	25	45.5	12	21	114
8098	37	35.5	17	68	23	44	12	25	41
8099	27	30	15	50	46	36	10	19.5	26
8100	35	36	17	60	21.5	41	14	27	36
8101	88.5	39	18	66	27	74	15	32	47
8102	29	32	15	47	19	39	10	26	48.5
8103	32	27	16	76	22	42	8	18	27
8104	34	32	16	51.5	19.5	39.5	11	22	53
8105	30	22	17	52	18.5	36.5	8	12	41.5
8106	28	26	13	55	17	34	9	15	57.5
8107	34	25	13	50	18	33.5	8.5	18	27
8108	37	25	32	115.5	29	40	13	30	23
8109	30	24	15	91.5	25.5	40	10	19	24
8110	24	20	16	61	24	33	10	16	21
8111	23	24	14	44	25	33	10	38	25.5
8112	43.5	31	32	132	33	53	11	36	46
8113	36	25	16	54	20	38	11	20	18.5
8114	54	26	16	56	32	39	25	27.5	20
8115	34	32	19	87.5	29	50	15	27	22
8116	57	49	28	101	27.5	71	13	22	62
8117	54	49	23	73	32	72	15	22	63
8118	99	80	36	201	40	94	25.5	53.5	92
8119	120	71	47	108	54.5	93	31	99	77
8120	50.5	48	33	117	39	70	16	33	66
8121	98	50.5	25	69	31	92.5	12	55	64
8122	61	62	28	77	33	81	17	37	59
8123	71	49	46	82	42	76	12	24	50
8124	59.5	60	42	85	31	77.5	17	34	45
8125	55	42	21	72	28	70	13	21	67
8126	66	42	22	68	22	70	12	23	37
8127	62.5	46	23	79	31	66	16	29	92.5
8128	64	46.5	28	94	40	73	26	28	54
8129	62	62	27	88	40	76	14.5	50	53

8130	94	66	28	75	32	103	19	37.5	52
8131	206	49	27.5	71	31.5	71	18	25	43
8132	61.5	48.5	24	66	39	68	13	21	54.5
8133	51.5	40	23	71	59.5	66.5	12	25	38
8134	86	62	27	93.5	35	97	18	42	58
8135	62	51	25	84	38	76	13	27	53
8136	60	47	36	145	28.5	70	15	45	57
8137	56	49	22	75.5	33	67	13	24	43
8138	49	53	27	66	25	70	16	36	50
8139	60	53	26	117	31	70	44	35	57
8140	63	50	23	76	33.5	73	23	43	51
8141	55	38	20	67	26	69	11	18	41
8142	64	82	44	94	95	91.5	25.5	52.5	76
8143	59.5	68	26.5	99	27	84	18	46	59
8144	44	39	18.5	49	21	61	12	20	54
8145	66	52	23	95	29.5	70	17	29	52
8146	57	47.5	20.5	87.5	26.5	73	13	25	44
8147	59	64	30	91.5	40.5	70	21	42	69
8148	50	37	17	70	29	61	11	22	29
8149	50	40	22	49	26	69	12	17	33
8150	45	35.5	19	59	48	56	10.5	17	37
8151	49.5	40	21	70	25	66	15	24	42
8152	54.5	42	23	65	22	69	16	27	52
8153	54	38	22	74	33	67	37	23	50
8154	52.5	45	23	82	25.5	69	15	29	41
8155	50.5	33.5	16	41	22	59	10	13	23
8156	61	36	19	34	29	61.5	9	40	24
8157	53.5	40	15	67	20.5	63	17	17	27
8158	38	36	10	23	21	56	8	14	22.5
8159	52	37	20	65	26	57	11	35	30
8160	57.5	38	16	48	69.5	60	12	79	26
8161	45	37	14	26	21.5	58	9	29	24
8162	30.5	30	15	31	38	50	10	15	30
8163	62	34.5	17	45	37	63	26	27.5	26
8164	60	36	18	48	27.5	60	10	16	38.5
8165	39	33	12	41.5	33	56	9	48	20
8166	40	35	15	43	19.5	54	8	15	29
8167	61	32	29	160	38	50	34	29	34
8168	37	34	12	50	27	56	8	12.5	25
8169	32	34	11	23	24	54	9	12	21
8170	44.5	38	18	66	37	56	12	32	27
8171	54.5	45.5	19	54	36	67	13	78.5	47
8172	41	34	11	88	23.5	49	8	16	20
8173	51	36	16.5	59	27	60	19	52	25
8174	40	37	14	36	22.5	55	8	14	47
8175	55	38	19	60	36	58.5	20	18	41
8176	45	32	13	34	16	57	9	14	23.5

8177	41	37	14	24.5	19	53	29	18.5	27
8178	50	33	14	41	20	55	8.5	18	22
8179	58	34	12.5	29	20	57	8	20	19
8180	37	33	12	42	21	51	9	19	21
8181	43	33	12	51	22	52	9	14	25.5
8182	44	34.5	13	47	21	60	8	15	26
8183	83	28.5	114	47.5	26	46	14	114	113
8184	253	38	28	45	28.5	65	16	172	30
8185	62	36	15	39	40	63	16	35	27
8186	110	33	16	34.5	28	54	10.5	20	29
8187	55.5	30	16	32	28	52	12	19	24
8188	105	46	17	75	31	106	15	30	52
8189	59	33	15	33	28.5	54	13	30	23
8190	51	27	11	58	22	46	11	15	20
8191	44	32	18.5	84	20	55	9	21	38
8192	38	29	11	40	21	49	8	13.5	17
8193	43	31	12	68	19	49.5	8	14	21
8194	36	27	11	43	16.5	48	7	14	21
8195	45	44	18	69	20	54.5	13	23	42.5
8196	85	30	13	36	24.5	50	10	15	30
8197	36	24.5	13	53	17	34	9	13	46.5
8198	34	23.5	16	30.5	23	34.5	11	18	38
8199	72	41	69	116	34.5	56	18	41	52
8200	37	27.5	15	55	25.5	46	13	17.5	25
8201	61	31	25	21	22	55	10	14	30
8202	59	32	35	31	24	54	12	16	27
8203	161.5	39	18	55.5	38.5	78.5	11	19	34
8204	51	34	20	173	26	56	49	30	90
8205	30	23	13	42	21	42.5	9	32.5	26
8206	48	34	22	63	34	54	43	22	54
8207	39	34.5	18	56.5	53	51	15	16	50
8208	40	26	17	62	30	46	13	23.5	53
8209	87	33	24	113	30	55	17	38.5	54.5
8210	30	27	13	47	29	35	11	16.5	24
8211	47	41	20	114	23	56	17	33	32
8212	49	35	16	74	20	59	12	21	45
8213	54	48.5	22	74	32	65	17	29.5	44
8214	42	30	18	60	22.5	52	9	18	46
8215	48	51	24	71	25	59	17	35	91
8216	178	55.5	25	72	40	77.5	21	43.5	49.5
8217	66	100	32	81	41	70.5	24	53	85.5
8218	48	57	27	70	27	57	21	44	56
8219	54	75	38	103	52.5	68	22	56	73
8220	42	33	14	75	24	52	10	19	26
8221	46	31	15	55	20.5	47	10	17	31
8222	37	27	14	51.5	24	48	9	17	40
8223	58	43	23.5	63	28	56.5	14	32	50

8224	59.5	58	30	53	34	72	17.5	38	52
8225	51.5	54	25	83.5	44.5	63	23	45.5	55
8226	54	62	29	83.5	31	70.5	27.5	49	67
8227	43	40	25.5	69	24	51	12	23.5	34
8228	45.5	46	23	67	26	58	17	31	45
8229	60	55	30	75	28	56	29	51.5	58
8230	84	43	24	58.5	24.5	105	16	29	40
8231	50	28	17	65.5	19.5	42	9.5	31	33.5
8232	80.5	39	21	54.5	20	79	12	26	35.5
8233	40	30	15	71	19	50	11	19	71.5
8234	77	37	23.5	59	21.5	64	11	21	32
8235	51	61	26	81	29.5	62	21	42	59.5
8236	40	37	19.5	70	26.5	45	13.5	29	68
8237	53	53	24	80.5	34	58	20	34.5	68
8238	42	29	18	55.5	21	54.5	11	21	48
8239	71	53	26.5	72.5	30	50	22	41	68
8240	58	43	29	63	23	63	13	27.5	56
8241	62	67.5	28	81	29	68.5	21	56	81
8242	99	53.5	21	79	27	91.5	13	29	44.5
8243	84.5	56	24	87.5	44	85	18	39	64
8244	48	43	19	76	20	62	12	26	36
8245	65.5	50	23	93	30	79	16	38	45
8246	60	37	20	67	32	58	16	32	42
8247	45	41.5	21	103	24	56	15	30	46
8248	45	44	22	60	29.5	57	14	40	44
8249	58.5	54	28	76	33	62	21	41	43
8250	47.5	55	27	72	27	62	19	40	64.5
8251	49.5	31	20	75	19.5	49.5	17	23	34
8252	45	33	23	78.5	19	51	11	22	40.5
8253	39	34	17.5	55	23	52	13	21	28
8254	89	31	16	83	20.5	50.5	10	22	30
8255	41	39	17	63	21	45	19	33	53
8256	59	39	18	96	20	60.5	14.5	27.5	37
8257	37	35	20	54	23	45	13.5	35.5	38
8258	69	72	38	87	29	63	39.5	59.5	77
8259	54	59.5	25	73.5	29	60.5	19	63	76
8260	49.5	49.5	25	75	33	54	20	38	59
8261	55	64	29	77	29	66	22	39	67
8262	44	45	22	75.5	22	54	15	36	44
8263	44	51	26	74	39.5	50	18	43	53
8264	58	56	21	59	23	73	14	36	51
8265	65	33	21	72	21	71	11	21	25
8266	36	28	15	49	25	49	9	18	31
8267	66	32	14	67	23	75	12	21	28
8268	53	56.5	28	83	34	54	17	42	52
8269	44	40	19	64	21.5	55	12	25.5	48
8270	41	26.5	18	66	29	45	9	19	51

8271	46	44.5	22	79	23	54	17	53	38
8272	36	30	16	53	16	46	10	19	36
8273	40	31	18.5	56.5	17	47	11	20	20.5
8274	63	69	28	92	26	62	20	52	72
8275	60.5	31	24	61	26	48.5	10	18	36
8276	40	30	18	56	25	42	13	23	26
8277	39	39	30	81	29	46.5	17	36	37
8278	34	28	18	65	26	33	15	38	34
8279	42.5	35	23	105.5	24.5	41	14	32	29
8280	50	23	16	46	29	39	10	26	27
8281	44	16	12	39	19	25	12	102	13
8282	35.5	26	20	59	27	33	17	31	19
8283	30.5	28	11	34	33	41.5	8	28	21
8284	43.5	25	17	38	26	35	23	37	21
8285	23.5	24	10	29	16	29	7	14	20
8286	52	24	31	41	20	32	9	15.5	17
8287	26	21	17	61	54.5	30	7	16.5	24
8288	27	21	10	55	24	25.5	9	17	15
8289	26	24	13	46.5	21	37	9	13	16
8290	37	27	12	39	25	34	9	19	17
8291	33.5	20	12	30	17	29.5	8	20	14.5
8292	20	20	13	43	31	25	8	15	18
8293	22.5	16	16	39	34	23	13	94	20
8294	19	17	10.5	58	15	23.5	13	14	10
8295	31	25	36	80.5	99.5	34	10	22.5	40
8296	56	35	20	86.5	22.5	57	19	27	32
8297	25	22	13	52	20.5	30	9	18	17
8298	24	20	11	49	19	29	13.5	32	19
8299	52	39	946	173.5	36	40.5	24	73	70
8300	24	19	15	30	28	29	10	22	15
8301	16.5	16	11	56	15.5	20	8	17	11
8302	39	16	13	61.5	23	19.5	59	22	34
8303	31	21	16	65	26	30	12	22	33.5
8304	31	39	13	40	26	38	8	17	22
8305	31	24	25.5	149	23	34	13	38	20
8306	30	20	16	108	22	22	9	21	31
8307	102.5	20	15	45	24	28	16	87	22
8308	34	22	13	81.5	27	42	9	20	20
8309	25	17	11	63	185	25	9	19	34
8310	39	17	15	50	21	24	7	32.5	11
8311	50	25	18	100	21	40.5	11	25	35
8312	38	41	25	86.5	79	49	14	28	52
8313	35	32	17	78	30	40.5	12	20	32
8314	45.5	50	30	81.5	31	48.5	21	45	42
8315	64	96.5	48	171	45	62	33.5	59	71
8316	59.5	53.5	37	118.5	60	59	21.5	43	84
8317	47	50	26	95	45.5	48	17	42	45

8318	834.5	66	26	70.5	33.5	98	15	35.5	60
8319	53	65	31.5	88	38	56	22	61	69
8320	92	46.5	24	85	29.5	109	10.5	30	79
8321	50	38	27	96.5	47.5	49	20	32	72
8322	63	66.5	35	144	36	86	25	51	80
8323	62.5	81	43.5	142	42	63.5	33	63	92
8324	42	46	29	97	51	46.5	15	35	47
8325	52	52	29.5	100	32	57	19	36	58.5
8326	66.5	80	38	117	40.5	58	31	66	79
8327	41	44	24	85.5	27	47	22	33	46
8328	42	32	26	114.5	33	42	15	31	36
8329	41	32.5	22	80.5	32	43	13	28	27
8330	57.5	59	26	99	29	66	14	30	64
8331	57	63.5	40	110	37	61	19	43.5	66
8332	28	30	18.5	77	22	39	10	22	40
8333	33.5	36	20.5	78	22	43.5	15	27	46
8334	61	65	34	114	34.5	58	21	48	85.5
8335	42	42	19	76	36	51	13	31	41
8336	40	26	20	118.5	26	41	13	33	44
8337	35.5	26	25	66	23	38	10.5	26	24
8338	77	62	26	117.5	34	90.5	18	39.5	73
8339	41	48	31	77	31	46	19	39	80
8340	32.5	28	14	73	20	35	10	19	34
8341	39.5	32	17	82	20.5	41	12	22	40
8342	46	42.5	24	96	36.5	43.5	16	31	47
8343	39	29	28	87.5	32	39	17	38	65
8344	37	28	20	76	25	42	12	33	44
8345	32.5	28	23	83	23	33	11	23	30
8346	40	45	25	89	33	48	18	37	67
8347	41	59	29	116.5	34	50.5	20	47	52
8348	37.5	52	28	73	29	45.5	19	43	64
8349	51.5	28	20	73	30	36.5	13	24	47
8350	80	77	36	146.5	36	65	26	59.5	75
8351	31.5	33	23	93	26	37.5	14	31	47
8352	29	29	22	74	28	34	12	23.5	32
8353	60	68	29.5	89	75	63.5	23	51	65
8354	42	42	25	80	31	42	15	38	82
8355	39	33	23	114.5	27	39	15	32	32
8356	67	55	22.5	92	27	66.5	14	33	47
8357	33	24	16	89	21	34	11	22	32
8358	37	49	29	98	33	43.5	19	36.5	50
8359	46	38	22	108	92.5	40	18	37	63
8360	31	28	20.5	77.5	26	32	11	28	30
8361	41	31	23	89	35	43	13	27	85
8362	71.5	44	36	126.5	34	52.5	22	55	79
8363	46.5	33	18.5	82	40	38	16	27	40.5
8364	58	37	25	109.5	31	39.5	17	36.5	42

8365	157.5	67	38	171	31	61	26	69	70
8366	36	54	31	102	33.5	45	21	45	58
8367	47	42	20.5	78	90	43	16	35	47
8368	31	23	13	69	31	34	10	17	22
8369	30	26	17	65.5	19	34	9	16	24
8370	107	32	19	79	27	70	12	25	45
8371	27	27	13	60	25	33	10	19	22
8372	22	22	14	60	25	32	8	15	19
8373	22	24.5	12	48	19	31	7	15	13
8374	69	28	22	52	32	39	23	82.5	29
8375	28	23	14	43	36	31.5	9.5	17	24
8376	23	22	13	58	24	29.5	16	19	18
8377	24.5	23	15	62	25	33	10	17	21
8378	32	26	14	67	20	37	9	17	29.5
8379	31.5	25	16	64	26	34	11	20.5	19
8380	28	24	37.5	44	26	32.5	11	22	49
8381	22	22	127	38	19.5	32	10.5	31	16
8382	31	27	18	117	26.5	31	10	42	19
8383	30	22	13.5	66	23	29	10	17	17
8384	31	22	14	67.5	21	31	13	19	29
8385	26.5	19	12	46	19	29	13	37	18
8386	156.5	54	53	267	65.5	70.5	71	189.5	41
8387	28	22	11	41	23	32.5	7	21.5	15
8388	60.5	30	27	58	38	39	27	70	28
8389	103.5	38	26.5	88	42	42	34	97	175
8390	51.5	32	28	121	84	35.5	20	50.5	56
8391	41	26	18	52	33.5	36	26.5	102.5	31
8392	13.5	18	6	15	11	22	6	14	10
8393	35	27	24	92.5	444	32	12	27	51
8394	51.5	32	18	79	32.5	54	9	22	29
8395	21.5	18	8	46	17	25	6	17	11
8396	76	22	19	65	39	32	34	84.5	19
8397	50	22.5	17	60	41	35	20	24	23
8398	22	22	17	51.5	30.5	27	11	23	17
8399	24	19	17	148	24.5	27.5	9	23	19
8400	30	24	65	63	24	32	13	23	146
8401	28	22	17	64	22	30	9	26.5	61.5
8402	35	33	20	101	32	33.5	12	34	27
8403	38	19	16	107	24	25	18	53.5	48
8404	31	22.5	15.5	43	20	30	12	17	18
8405	28	20	14	81	20.5	27.5	18	28	11
8406	18	18	11	39	14	27	8	13	11
8407	36	21	19	90	357.5	30	13	47	81.5
8408	36	31	28	99	27.5	35.5	18	55	64.5
8409	44	26.5	17	61	44	33	24	52.5	24
8410	17	18	8	15	11.5	26	9	13	10
8411	58	32	18	28	28	41	16	28	35

8412	101	35	25	106.5	23	61	16	36	46
8413	28.5	29	15	86	24	29	10	26	30
8414	37	44	24	102	65.5	36	17	38	65.5
8415	40	28	24	100.5	22	33	13	43	46
8416	41	48.5	28	132	26	40	18	43.5	62
8417	56	21	15.5	69	26	29	13	26	23
8418	42	37.5	24	90	21	38	14	45	44.5
8419	36	30	22.5	88	27	31	16.5	34	24
8420	32.5	26.5	21	97	24	33	14	31	37
8421	36.5	49	26	94	28	39	19	50	49
8422	35.5	38	22	103	25.5	34	14	40	44
8423	37	28	18	101.5	20	32	12	34	32
8424	30	27.5	17	101	21	33	15	28	33
8425	46	53	30	133	36	46	28	57	48
8426	41	61	24	125.5	24	38	17	40	35
8427	33	28	19	89	21	30.5	13	31	37
8428	42	64	35.5	112	31	45	49	54	55
8429	38	38	22	84.5	24	37	18	34	40
8430	67	33	24	88.5	32	39	12	28	35
8431	106	41	20	86.5	35	73.5	16	29	50.5
8432	36	46	21	73	25	37.5	19	42	45
8433	42	43	24	105.5	28	37	21	48	49.5
8434	28	39	18	69.5	25	34	15	30	33
8435	27	32	23	78	21	31	13	30	37
8436	38	32	22	91	23.5	35	32	38	43
8437	51.5	26	20	103.5	36	30	18	37.5	34
8438	60	77	36	123	43	64	39	67	76.5
8439	65.5	66	27	97	35	64	20	47.5	56
8440	48	57	27	90	27.5	48.5	22	53	57
8441	37	42	26	80	26.5	37.5	20	37	41
8442	47	74	30	95	30	49.5	28	57	74
8443	55	61	31	82.5	29	54	26	57	60
8444	39	49	26	84.5	32	43	20	44	48
8445	93	38	23	76	27	33	15	40	38
8446	55.5	46	30	87	35	54	14	39	63
8447	37	48	21.5	86.5	26.5	42.5	15	40	47
8448	30	32	24	68	23.5	33	14	29	27
8449	37	51	27	71.5	33	42	25	45.5	47.5
8450	49.5	38	21	81	25	56.5	13	28	42
8451	29	20	16	62.5	25	30.5	12	29	24
8452	39.5	57	30	85	32	44	24	66	52
8453	76	45	28	100	35.5	78	19	47	69.5
8454	35	36	20	100	22.5	41	14	29	44
8455	27	27	15	63	24	29	11	32	43
8456	34	36	23	95	29	36	16	32	33
8457	29	33	17	70	27	34	14.5	25	37
8458	1353	106	40	105	47	620	31	72	115

8459	23	26	17	90	28	29	10	24	33
8460	42	36	23	76	27	43	15	42	40
8461	41	32	22	80	27	39.5	13	29	38
8462	89	57	25	104.5	34	66	19	45	60
8463	42	51	29	101.5	36	47.5	21	56	51
8464	31.5	34	32	81.5	32	39	14	33	40
8465	27.5	23	14	57	18	30	9	21	37
8466	28	36	24.5	87	38.5	35	16	35	45
8467	44	51	31	87.5	36.5	49	24	44	52
8468	69	59.5	30	98.5	36	59.5	21	49	103
8469	63	75	40	85	34	73	26.5	67	80
8470	30	37	18	53	22.5	35	13	32	41.5
8471	32	35	22	84	26.5	37	16	33	31
8472	40	57	29	102	32	44	21	55	53
8473	36	44	22	83	20	39	16	43	54
8474	44.5	62	32	123	34	47	23	48.5	75
8475	47.5	58	30	89.5	34.5	51	27	60.5	86
8476	36	39.5	28	95	30	40	20	42	51
8477	35	45	56	90	27	38	18	39	61
8478	39	79	33	99	38	51	27	61	73
8479	29.5	37	23.5	65.5	38	37	11	30	31
8480	29.5	40	27	66	27	36	23	26.5	40
8481	34	41.5	24	111	25.5	42	18	34	37
8482	25	27.5	23	78	34	37	11	19	28
8483	47.5	37	25	67.5	24	37	18	32	58
8484	40	50	27	66	39.5	41.5	20	42	77
8485	58	25	14	51	26	28	11	17	16
8486	23	28.5	13	58.5	21	33	9	15	16.5
8487	34.5	26	16	56	20	32	11	15	16
8488	25.5	27	14	44	26.5	29	12	16	18
8489	25	27	13	55	32	31	8	15	18
8490	27	25.5	15	84	30	34	8	18	27
8491	27	25	17	66	22	28	10	20	19
8492	29	29	17	63.5	23	33.5	10	19	21
8493	29	24	13	62.5	21	27	9	21	17
8494	24	24	11	61	53	27.5	8	15	17
8495	26	25	32	91	32	31.5	13	43	36
8496	31	27.5	15.5	42	31.5	30.5	16	68.5	21
8497	69	25	14	48	40	28	20	145	19
8498	33	25	17.5	75	30.5	35	12	23	21
8499	29	26	18.5	83.5	22	27	12	28	17
8500	31	25	20	90.5	19	30	13	28	18.5
8501	29	25	16	100	29	28	21	24	20
8502	27.5	24	15	74.5	33.5	28	13	21	16
8503	23	22	19	51	86.5	27	8	17	16
8504	23	22	9	68	36	27	8	14	15
8505	27	23	13	74.5	23	31	13.5	36	17

8506	23.5	21	14	38.5	19	27	10	20	17
8507	17	22	10	28	16.5	28.5	6	11	14
8508	36	30	14	66	48	32	11	17	22
8509	143	29	50	62	37	41	23	32	48
8510	37	26.5	21.5	142	41	31	12	52	40
8511	42	36	20	78	27	49	15	29.5	37
8512	27.5	28	17	61	27	30	11	29.5	32
8513	30	32	17	72	24	35	16	40	30
8514	39	30	19	73	27	36.5	10	29	25
8515	32.5	55	23.5	89	25	40	20	43	47
8516	40.5	34.5	19	97	26.5	44	13	28	31.5
8517	33	34	25	78	29.5	34	34	35	46
8518	32	33	22	99	25	36	13	32	29
8519	28	21.5	14	63	20	27	8	17.5	19
8520	32.5	37	22	83	28	36	17	43	41
8521	39	47	25	68	31	41	20.5	47	43
8522	50	37	20	84	24	35	12.5	36	54
8523	30.5	46	24	76.5	23	36.5	17	37	38
8524	43	49	24	91.5	31	42	23	44	50
8525	39	38	21	68	26	44	16	37	36
8526	41	50.5	37	100.5	32	49	25	46.5	50
8527	43.5	39	35	69.5	26.5	34	19	40.5	36.5
8528	29	39	22	80	23	33.5	18	34	40
8529	49	47	28	103.5	76.5	39	18	55	44
8530	31	42	21.5	94	27	37	17	42	56
8531	25	36	18.5	62	30	33	13.5	27	41
8532	34.5	31	16	75.5	22.5	31	13.5	26	42
8533	26	36	18	84	29	31	15	35	43.5
8534	36.5	26	19	75	39	28	12	23.5	28
8535	92	36	28	79.5	28	37.5	18	39	32
8536	75.5	45	22	77	24	37	17	34	65
8537	40	54	31	87	46	37	22	60	59
8538	30	37	23	82	28	34	14	33.5	39
8539	27	25	15	67.5	25	30	13	26	41.5
8540	34	54	28	83.5	31	39.5	24	51	61
8541	44	60	31	87	32.5	44	23.5	60.5	65.5
8542	33	31	19	72	24	29	13	24	36
8543	352	29	21.5	68.5	28.5	69	12	23.5	47
8544	37	48	27	102	37.5	37	17	44	55
8545	36	54	30	82	28	41	22	49	61
8546	28	38	21	75.5	32	29.5	13	32	45
8547	30	34	21	59	26	25	16	34	32
8548	25	35	18	67.5	22	32	15	31	42
8549	57.5	46	25	104	26	41.5	19	48	49
8550	28	41	20.5	71	28	34	17	35.5	39
8551	31	32	19	56	18.5	37	13	27.5	34
8552	27	32	25	91	24.5	32	18	31	37

8553	31	48	22	94.5	27	38	17	44	50
8554	31	38	19	79	29	36	16	31	46
8555	15	19	9	45	13	21	7	12	11
8556	19	18	13	53	25	22	7	15	11
8557	25	16	12	77	20	22	12	39	11
8558	19	18	11	58	19	22	9	16	13
8559	22	21	14	48	17	24.5	9	17	12
8560	29	19	14	46	20	22.5	11	23	15
8561	20	19.5	10	33	20.5	22.5	11	24	14
8562	26	27	13	50	147	19	13	49.5	65
8563	61	25	21.5	34	28	29	16	81	20
8564	50	24	28	112	38.5	42	14	53.5	46.5
8565	32.5	21	12	59	23	21	19	26.5	37.5
8566	29	18	11	40	32	21	10	17	12
8567	28	20	11	31	28.5	27	14	26	17
8568	23	22	22	111	23	26	61	26	150
8569	33	20.5	30	75	27.5	26	23	24	28.5
8570	18	18	10	27.5	30	24	12	16	13
8571	43	28	19	112	32.5	36	18	36	25
8572	78	35.5	23	114.5	39.5	48	16	44	37
8573	65	46	22	86	37	75.5	13	21	48
8574	71	51	25.5	99	32	72.5	17	34	44
8575	79	73	38	203	43	100	26.5	52	98.5
8576	72	59	30.5	119	41	74.5	31	48	83.5
8577	70	49.5	24	108	27	77.5	15	28	40
8578	62	56	33	98	33.5	58.5	21	40	52
8579	60	37.5	23	86	71	67	13	23.5	62
8580	63.5	46	26	102	45	64	20	49	100.5
8581	57	41	23	98	37	64	14	29	34
8582	215.5	66	33	112	43.5	79	23.5	49	107
8583	160.5	66	30.5	148	29	169.5	18.5	38	56.5
8584	74	60	30	87	40	70	21	43	50
8585	73	38.5	21.5	88	34	59	12.5	22	48
8586	79	58	32	106.5	38	65	19.5	47	75
8587	141	92	45	109	45	88	35	68	85
8588	78	42	25.5	170	23	59	13	31	46.5
8589	66.5	58	34	101	43	67.5	22	40	100
8590	77	58	27	126	31	73	28	44	88
8591	76	55	33	181	39	67.5	22	49	46
8592	59	46	24	91	38.5	62	15	31	40
8593	73.5	56.5	28.5	125	35	55.5	20.5	62	74.5
8594	61	58	31	93.5	32	66	20	41	63
8595	112.5	38.5	27	103	24	60	15	36	62
8596	61.5	38	20	68	28	75.5	11	22	37.5
8597	64.5	39	21	72	27	55	13	26	56
8598	84	45.5	27	120	26	72	18	37.5	74
8599	172	126.5	122	146	135	181.5	138	98	134

8600	62	35.5	21	111	57.5	73.5	12	24	66
8601	56	44	22	103	28	52.5	17	58	39
8602	69	42	22	112	24	67	14	31	61
8603	120	55	31	101	53	69	19	44	80
8604	371	81	32	166	25	355	18	29	74.5
8605	59	74	34	107	34	72	25.5	119	84
8606	56	55	31	129	33	60	22	42	51
8607	53	47	27	90	32.5	54	16	48	41
8608	75.5	56	29.5	99.5	31.5	62	19	41	65
8609	69.5	70	34	150.5	29.5	73	25	76	83
8610	61.5	30	17	77.5	43	49	15	29	34.5
8611	43	28.5	13	58	22	46	7	15	23
8612	37	34.5	22	92	22	42	14	21	20
8613	43	33	16	80	161	55	11	50	24.5
8614	81	27	35	52	66	45	12	20	26
8615	42.5	28	14	61.5	22	51	10	19	48
8616	44	29	12	65	22	48	10	18	23
8617	43	30	18	97	21	43	10	21	39
8618	61.5	31	12	39.5	19	47	9	16	21
8619	74.5	35	24	190	36	62	32	50	27
8620	50.5	32	14	58	22	52	11	23	42
8621	85	59	45	134	52.5	61	21.5	49	73
8622	74.5	94	59.5	171	148	68	25	62	74.5
8623	98	41	25	119	29	57	16	39	37
8624	53	29.5	25	102	26.5	51	10	21.5	44
8625	66	48	29	101	37.5	58.5	14	35	63
8626	56.5	34	23	125.5	233	54	12	31.5	49
8627	66	50	30	119	41.5	61	18	45	64
8628	58	40	21	97	20	60	12	28	40
8629	68	47	22	108.5	28	62	24	37	49
8630	45	34	27	107	29	53	10	24	37
8631	53.5	33	24	124	29	51	13	29	32
8632	56	30	25	160.5	19	48	14	30	47
8633	64	52	29.5	121	32	55	19.5	44	107
8634	58	30	19	86	28	54	11	25	38
8635	59.5	48	27	116	40	58	17	45	44
8636	57	28	20	138	33.5	48	11	51.5	38
8637	40.5	28	11	26	19	48	8	18	16
8638	44	29.5	13.5	68	39.5	51	9	17	24.5
8639	41	27	12	58	154.5	42	10	18	18
8640	43	27	17	71	17.5	47	10.5	19	21.5
8641	63	34	21	135.5	22	56	11	34.5	35
8642	55	27	579	39	49	42	12	24	20
8643	104.5	27	21	86	31	46	34	27	26
8644	50	26	17	43	31	38	15	69	21
8645	49.5	29	16	37	129	46.5	19	21	24
8646	68.5	27.5	35	72	46	46	23	29	33

8647	55	32	22	62	46	52	13	45	52
8648	49.5	28	18	158.5	28.5	45	13	29.5	22
8649	57	49	23	88.5	21.5	50	17	40	73
8650	46	27	33	93.5	32.5	44	13	48	166.5
8651	111	40.5	25.5	92.5	20.5	46	16	35	46
8652	73	25	16	55.5	16	57	10	20	40
8653	38	32	20.5	81	18	46	10	21	177
8654	41	25	42	103	28.5	38.5	17	23	35.5
8655	40.5	23	23	82	114	41	125	23	38
8656	56.5	27	21	129.5	102.5	52	28	25	45.5
8657	45	45	18	45	22.5	37	11	27	23
8658	97.5	25	13	65.5	152	41	12	107.5	31
8659	59	27	17	57	27	47	13	20	25
8660	49	27	18	84.5	36	42	12	26	62
8661	34	18	11	38.5	16.5	38	9	16	16.5
8662	31.5	27	16	23	13	41	9	13	17.5
8663	54	27	23	98.5	23	47	11	30	21
8664	41	22	14.5	94	16	35	9	23	15
8665	29	22	7	15	8	35	7	12	14
8666	34	21.5	9	35	38	38	8	17.5	16
8667	49	24	13	52	57.5	46	11	19	21
8668	32.5	23	12	33	17	35.5	15	23	18.5
8669	40	29	13	54	24	44.5	23	58	40
8670	37	21.5	14	50	16	38.5	10	25.5	30
8671	44	27	15	47.5	27	41	10	18.5	20
8672	38.5	24.5	14	46.5	20	44	11	23.5	26
8673	60	29	31.5	71	841.5	42.5	16	57	53
8674	86	29	22	110.5	44	41	23	29	96.5
8675	57.5	27	28	82.5	39	45	21	39	27
8676	27	23	8	36.5	15.5	41.5	8	13	15
8677	40	26	16	76	25	37	11	23	24
8678	54	41.5	25.5	102	41	53	18	37	61
8679	63	84.5	38	169	55.5	59	35	74	105
8680	43	25	17	62	25.5	41	11	21.5	55.5
8681	53	24	27	73	26	36	11	24	31
8682	62	79	36	137	33.5	69	32	67	79
8683	56	44	28	97.5	29	54	18.5	53	61.5
8684	65	53	27	84.5	57	54.5	19	47.5	65
8685	64	47	28	156	48	58	17	46	54
8686	35	24	17	61.5	19	37	12	24	28.5
8687	54	37	21	94	25	46	15.5	32	51.5
8688	40	52.5	18	71	22.5	35	12	37	27
8689	49.5	24	21	102	26	42.5	60	25	33
8690	45	24	21	117	40	40.5	12	33	21.5
8691	56	31	24	118	29	43	16	36	40
8692	45	28	26	80.5	27	48	11	61	38
8693	44.5	28	24	113	35	42.5	12	45	39

8694	48	25	19	84	63	40	10.5	22	30
8695	50	33	20.5	111	26	49	13	33.5	43
8696	59	72	28	100	32	57.5	24	69	66
8697	47	31	19	142.5	23	52	11	27	46
8698	57.5	38	25	99	33	47	16	44.5	38
8699	48	48.5	35.5	109	27	48	19	40	53
8700	46	30	20	100	41	41	20	30	43
8701	59.5	49	26	87.5	89	50	15	41	63
8702	62	23	18	77	33	54.5	12	26	34.5
8703	66	29	19	77	27.5	58	14	23	48
8704	51.5	37	22	92	44	48	18	34	39.5
8705	48	25	16	114	25	42	9	26	51
8706	46	42	23	96.5	32.5	43	15	35	107
8707	310	37	24	88	30	49	17	36	54
8708	49	27	15	89	25.5	46	12.5	26	45
8709	60	26	18	86.5	44	42	15	25	31
8710	46.5	26	20	70	24	38	14	23	49
8711	50	25.5	17	88	28.5	45	13	25	51
8712	60	33.5	23	117	26	45	14	45	54
8713	49	24.5	18	84	31	45.5	11	22	24
8714	47	26	20	99.5	28.5	41	14	26	38.5
8715	46	38	23	88	28	44	17	55	122
8716	60	44	27.5	113	40	52	19.5	50	63
8717	51	33.5	19	78.5	52.5	43	13	34.5	48.5
8718	53.5	48	33	90	31	57.5	23	47	79
8719	61	22	21	99	20	37	10.5	31	28
8720	54	37	24	88	29	41.5	18	35	72
8721	47.5	24.5	23	81.5	23	43	13	41	26
8722	45	39.5	27.5	98.5	37	51.5	16	34	46
8723	55	52	29	105	36	52.5	26	49	84
8724	61	30	24	104	31	44	17	35	65.5
8725	44	32	22	80	20	44	13	26	52
8726	57	51	32	91	37	59	24	54	66
8727	58	40.5	26	84	31	45	19	39	67
8728	60	53	31	96	29	52.5	22	45	60
8729	43	47	22.5	68	34	56	15	34	51
8730	68	58	32	120	33	85	20	45	65.5
8731	46.5	45.5	26	95	47	52	15	45	66
8732	62.5	87	43.5	148	47.5	77.5	33	66	90
8733	31.5	38	15.5	69	23	47.5	8	14	24
8734	68	45	53.5	52.5	111	56.5	22	46	45
8735	35	35.5	19	40.5	40	46	11	17	32
8736	45	45	25	100	37	62	15	26	84
8737	54	41	27	101	59	57.5	47	35	37
8738	30	36	16	41	110.5	47	13	19	47
8739	36	33	16	57	39	39	11	20	38
8740	32	35	26	67.5	35	50	10	22	42

8741	30	36	20	55	29	49	10	17	51
8742	44	36	25	52.5	36	53	11	20.5	63
8743	37.5	40	22	49	44	55	16	19	77
8744	30	38	16	57	24.5	52	10	16	43
8745	41	38	24	90.5	28.5	52	11	20	44
8746	55	33	15	68	45	48	10.5	20	27
8747	31	36	17	109.5	28	44	10	20	38
8748	33.5	37	14	49.5	40	49	11	28	23
8749	29	41	17	51	96	45.5	13.5	60	52
8750	43	37	17.5	79.5	32	48	14	35	24
8751	40	36	16	55	39	47	14	27	50
8752	42.5	37	17	51	30	47.5	13	17	24
8753	39	37	22	112	70	46	12	19	31
8754	45	30.5	13	44	26	41	9	18	25
8755	32	38	23	77	29.5	42	16	36	36
8756	51	57	29	101	92	58	25	49	58
8757	39	36.5	25	81	35	49	14	26	51
8758	37	47	28	95	42	53	20	34.5	54
8759	39.5	33	19	93	44.5	48	14	27	30
8760	54	50	24	79	38.5	69.5	17	35	58
8761	63	50	20	101	43	76	14	28	42
8762	52	61	26	80	28.5	72	18	38	47
8763	68	70	45	113	64.5	61	33	81	76
8764	48	49	27	104	37	55	17	34	51
8765	48	44	24	88	36	55.5	16	32	47
8766	41	50.5	24	90	30.5	56	18	35	63
8767	33.5	35	21	70	30	42	16	23	25
8768	52.5	45	24	74	32	54	15	30	44.5
8769	38.5	56	23	76	50	55	20	37	45
8770	36	39	22	81	24	45	12	29	34
8771	38	31	23	61.5	27	40	15	28	29
8772	42	51	26.5	98	33	52	21	37	53
8773	103.5	86	38	102	43	102	29	176	90
8774	45	63	34	113	41	57	23	52	71.5
8775	37.5	41	19	70	26	48	13.5	29.5	39
8776	43	52.5	24	85	31	59	19	39	44
8777	37	52	22	71	36	51	19	37	52
8778	68	56	31	95	74	61	19	42.5	89
8779	40.5	36	17	64	33	47	14	31	26
8780	52	63.5	40	96	44.5	62	32	55	63
8781	142	32	19	82	38	71	15	22	49
8782	28	29	17	79.5	22	39	15	20	24
8783	34	39	19	82	32	48	16	26	42
8784	49.5	32	18	45	39	46	12	56	41
8785	44	32	20	94.5	31.5	45	13	20	36
8786	50	33	20	48	44.5	60	13	24	38.5
8787	28	28	15	66	28.5	37	9	16	20

8788	34	32	15	56	26	44	9	43	19
8789	38	34	18	63	34	49	15	36	31
8790	35	30	24	92	59.5	40	28	18	21
8791	29	27	15	54	348.5	41	17	16	67
8792	35.5	29	27	48	36	41	22	26	74
8793	40	27	288	46	31	41.5	14	18	59
8794	29	28	20	54.5	29	40	18	17	21
8795	98	31	16	43	29	41.5	22	14	24.5
8796	35	28	22	63	29	41	12.5	17	24.5
8797	32.5	29.5	28	64	32	39.5	12	15	25
8798	30	29	18	50	30	42	38	17	30
8799	35	30	24	50	30	50	17	21	35
8800	34.5	31.5	26	107.5	38	42	16	23	22
8801	39.5	29	13	43.5	27.5	42	15	20	20
8802	43	30	24	60	35	49	12	18	27
8803	62	30	23	59	32	43	19	33	39
8804	33	29	16	63	32.5	45	13.5	17	24
8805	40.5	39	20	45	120.5	60	16	17.5	29
8806	44	35	22	77.5	35	43	16	30	40
8807	40.5	29	17	46.5	29	36	9	18	33
8808	67	72.5	41	80	59	59.5	25	55	79
8809	59	44	26	73	35	57	19	35	50
8810	45	69	36	86	51.5	58	30	57	72.5
8811	51	58	28	88.5	35.5	61	20.5	46	61.5
8812	76	67	34	94	51	74.5	23	59	91.5
8813	52.5	34	20	82	31.5	46	16	33	44
8814	42	38	24	109	43	38	53	60	55
8815	38	40	27.5	69	29.5	41	16.5	34.5	63.5
8816	92	22	17.5	59.5	25	34	8	18	51
8817	44	27	18.5	66	26	36.5	11	25	51.5
8818	37	32	19.5	64	35	36	18	26	30
8819	29	25.5	12.5	40.5	20	34	8	15	31
8820	51	66	33	134	36.5	49	25	49.5	69
8821	48.5	33	18	69	30	39	13	25	51.5
8822	53	56	32	92	40	56	31	48.5	77
8823	46	46	33	78.5	37	46	16	55	44
8824	58	42.5	26	63	35	46	17	37	44
8825	77.5	39	23	51	35	46	16	35	87
8826	60	104	35	129.5	43	59	29	56	98
8827	58	55.5	31	57	38	56	21	40	85
8828	47	70	32	86	40.5	49.5	28	65	74
8829	49	39	18	51	22.5	55	11	73	39
8830	44	62.5	33	80	36	47	26.5	52	70
8831	35	38	24	62	33.5	39	15	31	44
8832	72.5	82	46	86.5	47	80	33	60	90
8833	68	91	56	126	75	74	49	81	111.5
8834	55	71	38	86.5	39	59	31.5	63.5	72.5

8835	34	25	15.5	69.5	45.5	31	9	20	24
8836	45	40	26	84	90	41	18	37	45
8837	37	40	26	82	36	42	18	40	75
8838	35	18	25	68.5	25	30	9	17	43
8839	43	36	36	77	42.5	30	15	35	48
8840	65	67	37	90	43	55	25	58	77.5
8841	34	20	29	60	30	30	11	19	65
8842	34	22	14	56	22	27	11	21	36
8843	43	21	12	51.5	25	28.5	9	17	51
8844	28	17	14	60	31	25.5	9	16.5	64
8845	27	25	14	63	26.5	27.5	10	19	38
8846	26	14	17.5	55	33	21	8	32	18
8847	31	21	14	52	20	25	8	19	16
8848	36.5	42	21	59	29	39	16	37	41
8849	47	21.5	16	56	38	40.5	8	18	45
8850	23	16	13	47	28	24	9.5	15	34.5
8851	25	23	16	55	24	28	9	24	23
8852	73	17	15	80.5	70.5	26	8	18	16
8853	40	41	29	69	35	36	19	42	46
8854	30	19	14	73.5	26.5	27	10	19	37
8855	55	58.5	28	71.5	33	47	22.5	62.5	81
8856	103.5	50	31	83.5	33.5	47	23	45	60.5
8857	47.5	60	30	91	39.5	46	23	47	57
8858	96	32	17	76	24	41	13	27.5	34
8859	18.5	15	7	16	512.5	24	5	9	15
8860	20	16	8	15	23	27	5	8	12
8861	19.5	16	8	34	32	25	5	10	12
8862	17	13	6	22	21	20	5	9	9
8863	20	17	9	20	26.5	25	6.5	12	34
8864	22	17	7	15	22	26	5	8	13
8865	26	17	16	61	59.5	22	12	17	43
8866	17.5	13	5	13	27	21	6	9	9
8867	23	16	11	45	29	22	12	19	27
8868	34	16	103	40	49	32	12	18	19
8869	44	19	22	55	36	34	14	32	46.5
8870	24.5	13	13	49.5	24	21	8	14	13
8871	19	18	10	20	56	26	7	11	12
8872	38	21	14	48	38	29	13	76.5	22
8873	34	18	15	72	37	28	31	19	26
8874	38	23	14	43	19	38	10	17	28
8875	26	17	13	80	25	28	10	18	16
8876	27	17	25	77	26	30	8	20	33
8877	28	28	18	64	20.5	31	11	25	71.5
8878	42.5	20	16	93.5	37	30	14.5	23	32
8879	29.5	18	18	63	106.5	28	14	18	47.5
8880	100	21	25	90	416.5	32.5	65.5	113	49
8881	34	19	17	67	46.5	29.5	10	43	44

8882	37	21	74	180	41.5	30.5	60	45	29
8883	29	18	15	56	46.5	25	46	25	25
8884	46.5	21	21.5	98	41	36	14	25	53
8885	32.5	20	21	96	37	29	11	25	56
8886	27	14	21	88	20	22	9	19	11
8887	21	13	16	37	21	22	13	13	22
8888	49	16	19	79	28	25.5	17	20	18
8889	27	75	25	63	24	25	8	17	17
8890	26	16	15	81.5	24.5	23	8	22	20
8891	23	16	13	65	29	24	6	15.5	21
8892	30.5	15	12	59	82	25	20	16	36
8893	31	16	36	72	37	22	9	26	90
8894	54	19	26	86.5	70	33.5	15	27	269
8895	41	18	14	55	37	36	11.5	29	156
8896	34	18	34	81	34	28	14	26	36
8897	42	16	20	70.5	206	23	24	24	30
8898	35	21	22	71.5	41	26	12.5	24	43
8899	28.5	17	18	81	27.5	24	16	20	59
8900	26	15	12	51	36.5	20	19	17	12
8901	57.5	21	33.5	111	242	29.5	20	38.5	67
8902	22	14	15	56	34.5	23	9	21	30.5
8903	66	23	16.5	48.5	37.5	35	11	19	27
8904	27	16	10	22.5	26.5	23	9	22.5	19
8905	159.5	20	22	44	55	23.5	85	22.5	19
8906	83.5	27	22	103	39	53	46.5	132	28
8907	120	45	21	41	32.5	86.5	20.5	30	35
8908	22	12	7	36	20	21	5	11	11
8909	20	13	9	30	19	22	6	9	8
8910	25	14	8	25	22	22	9	37	10
8911	22.5	13	6	29	41	22	6	13	15
8912	27	22	16	46	28	33	21	15	20
8913	23	16	8	26	25.5	22	15.5	16.5	9.5
8914	113	22	18	68	24	25	14	28	28
8915	19	14	13	47.5	19	17	9	17.5	15
8916	32	22	24	70.5	31	24	18	33	27
8917	37	45	30	110	29	36	23	42.5	72
8918	40	45	27	72	34	38	19	47	60
8919	41.5	50	26.5	62	34	42	21	44	46
8920	27	24	17.5	61	27	24	14	39	28
8921	38	25	26	77	27	21.5	13	23	35
8922	31	19	18	83	26	24	14	23	23
8923	33	47.5	27	78	33	32.5	21	43	68
8924	31	30	112	79.5	27	25	17	36.5	46
8925	203	15	14	77	21	35	13	18	24
8926	26	20	18	47	21	19	13	21	41
8927	35	46	31	120	31	34	21	47.5	40
8928	49	70	39	98	44	43.5	31	69	69

8929	27	28	20	60	25.5	23	14	28	73.5
8930	34	15	23	47	26	29	8	17	14
8931	28	16	17	59.5	25.5	22	14	26.5	36
8932	53	42	28	74	27.5	35.5	20	44	54
8933	40	51	30	79.5	35	37	23	52	55
8934	23	14	15	48	47	18	9	15.5	21
8935	27	16	13	61	24	26	8	23	37.5
8936	55	130	20	119.5	26	77	11	34.5	64
8937	28.5	36	20	67.5	26.5	25	17	34	34
8938	66	52	27	87	32	42	21	49	79
8939	41	68.5	34	103	37	43.5	29	64	65
8940	27	37	42	84.5	27	28	18	34	34.5
8941	25	24	21	84	24	22	14	23	37
8942	38	64	36	104	33.5	44	22	51	59
8943	20.5	13	12	51	23	17.5	10	17	23
8944	24	13	13	65	20	19	14	42	19
8945	33	43	23	82.5	39	31	18	49	85
8946	69	67	38	89	39	46.5	30	63.5	72
8947	31	24	20	90	29	26	13	26	38
8948	25	13	17	82	23	17	9	20	31
8949	23	16	18	61	25	20	10	25	26
8950	23	27	19	73	22.5	23	13	32	31
8951	30	39	34	77	31	26	15	36	47
8952	35.5	61	33	88.5	30	36.5	24	52	62
8953	40.5	61	32	111	36	37	28	54	66
8954	31	11	14	67.5	22	14	10	24	23
8955	52	61	45	103	41	51	39	58	101.5
8956	34	17	19	66	26	22	16	35	31
8957	53	32	17	59	31	48	14	31	36
8958	29	24	20	65	25	19	13	30	28
8959	30	32	22	76	26	25	16	32	39
8960	124	55	29	90.5	34	40	23	59	63
8961	25	21	16	66	26	20	13	25	40
8962	26	38.5	24	71	24	25	17	37	39
8963	44	57	37	90	36	39.5	29	55.5	68
8964	43.5	65	30.5	85	35	33.5	25	62	68
8965	22	13	34	62.5	24	18	9	20	59
8966	18	15	12	45	21	15	11	19	14
8967	74	11	15	60	22	15	8	32	13
8968	20	11	11	54.5	18	15	8	16	11
8969	27	32	27.5	88	27.5	22	15	45	44
8970	41	23	18	61.5	25	19	14.5	33	25
8971	32	50	28	84	35	31	21	43	62
8972	31	33	21	71	26	25	18	34	58
8973	28	34	21	81	28	25	15	40	40
8974	20	13	18	53	38	18	9	17	34
8975	27	21	17	63.5	21.5	20	12	28.5	55

8976	32	48	27	104	32	33	21	49.5	64
8977	28	26	19	60.5	27	22.5	15.5	33	36
8978	29.5	26	18	85	30	23	17.5	28	37
8979	34	40	28	85	45	32	23	46	56
8980	43	75	42	102	44	46	33	86	84
8981	30	37	22	89	31.5	26	18	45.5	39
8982	42	64.5	28	100	31	37	28	57	63
8983	117	35	27	107	33	36	31	39	44.5
8984	24	19	17	78	24.5	22	10	67	42.5
8985	34	38	27	55	30	29	20	38	52
8986	29	28	24	70	26	24	17	31	47
8987	25	12	10	52.5	20	21	8	15	15
8988	55	53	36	100	36.5	39	31	61	55
8989	22	14	14	53.5	22	16	9	19	27
8990	27	30	22	97	25	26	18	34	42
8991	34.5	27	24	82	29	29	18	33	46
8992	34.5	46	26.5	82.5	37	32	24	53	76
8993	32	21	20	71	24	29.5	12	32	27.5
8994	23	19	16	52	21.5	17	11	45.5	19
8995	20	11	12	66	21	14	15	18	17
8996	20	11	12.5	51	17	14	9	15	13.5
8997	29	30	22	49	25	28	15	30	29.5
8998	38	38	24	64	31	35.5	21	33	46
8999	32	21	22	67	28	27	13	21	26
9000	36.5	47	32	86	38	38	23	45.5	50
9001	108	47	22	97.5	26	104	13.5	27	50
9002	55	53	39	82	45	58	63	70	49
9003	33	46	28	69	29	34	20	41	42
9004	44	42	27	91	33.5	35	20	44	44
9005	32	33	21	89	27.5	30	17	36	34.5
9006	38.5	41	26	86	30	36	22	37	41
9007	51	30	29	131	31.5	41	15	32.5	42
9008	35.5	30	24	94.5	32	29.5	20	30.5	49
9009	30	18	16	48	28	23	10	15	36
9010	44	18	21	93	40	24	21	30	41
9011	59	18.5	14	36	29	25	19	75	28
9012	16	12	8.5	47	20	16	7	11	11
9013	39	19	19	96	51.5	25	11	21	19
9014	30	16	15	106.5	21	23	12	19	25
9015	256	59	34	135.5	36	128	23	72	51
9016	53	64	41	124	41.5	46	31	61	79
9017	54.5	58	31.5	131	40.5	45	28	53	51
9018	28.5	19	16	83	20.5	26	10	20	29.5
9019	47.5	31.5	27	126.5	29	29	18	38	30
9020	45.5	42.5	31	121	34	34	22	51	75
9021	83	31	23	103	31.5	55	16	32	44
9022	49	29	21.5	99	24	33	16	33	31

9023	42	40	30	104	30	37	20	46	51
9024	38	39	28	102	26.5	31	19	38	39
9025	34.5	20	18	77	28	25	12	28	26.5
9026	27	27	20	75	23.5	22	12	38	28
9027	42	31	26	126.5	26	27	29	36	31
9028	31	25	21	98	26.5	21	16	35	48
9029	85	42.5	23	116	26	54	18	43	49
9030	37	31	25	95.5	26	27	18	34.5	35
9031	48	59	40	175	38	43	28	62	89
9032	49	75.5	39	118.5	35	44	32	71	116
9033	49	45	30	114	30	31	26	46	47
9034	37	45	32	104	30	35	21	44	63
9035	44	50	28	108.5	29	36.5	23	47	53
9036	36	16	22	103	29.5	21	12	28	31
9037	35	39	21	75	26	26	15	36	43
9038	66.5	38	29	102	29	55	17	37	38
9039	28	17	18	100	23	22	13	25.5	27
9040	44	77	38.5	117	37	46	29	64	100
9041	32	31	24	96	24	30	15	37	49
9042	36	43	29	108	29	32	19	57	49
9043	35	38	24.5	82	28.5	28	19	42	49
9044	43	45	32	117	31.5	35.5	23	52	49
9045	46	38	26	96.5	33	28	21	46	48
9046	52.5	50	40	150	36	35.5	25	58.5	51.5
9047	33	31	22	101	26	29.5	15	32	31
9048	31	25	24	89	25	17	16	33.5	31
9049	33	29	27	104	31	24	15	45	62
9050	33	27	18.5	86.5	25	24	13	32	63
9051	65	72	43	122	42.5	48	36	80	81.5
9052	44.5	57	40	143	37	44	38	54	109
9053	36	29	19	87	23	24	14	28	34
9054	32.5	35	87	97.5	25	30	18	44	43
9055	44	28	19	99	23	28	17	30	33
9056	26.5	13	18	77	17	16	9	17	16
9057	34	32	22	109	25	27	15	47	36
9058	26.5	15	36	62.5	26	17	123	23.5	16
9059	40.5	13.5	20	141	24	18	33	26	19
9060	270.5	146	151	401.5	249.5	192	393	337	138
9061	43	17	26	145	32	20	31.5	36	21
9062	29	11	13	126.5	18	13	25	24	30
9063	22.5	15	15	75.5	21	17	8.5	16.5	18
9064	48.5	12	12	65	29	15	26	30	32.5
9065	52	19	27	150	28	24	19	40	54.5
9066	18	11	11.5	69	22	13	7	15	11
9067	175	41	33	123	56.5	45	43	88.5	51
9068	44	14	21	111.5	28	16	12	22	29.5
9069	27	13	8	32	22	16	8	12	9

9070	31	15	32	64	25	17	10	22	14
9071	30	12	24	103	23	18	30	24	18
9072	34	12	9	40	25	13.5	8	19	11
9073	24	12	10	28	23	17	12	18	17
9074	51	18	25	145	32	27	14	37	27
9075	32	13	15.5	76	24	16	16	31	43
9076	18	11.5	13	40	22	15	12	13	10
9077	27	15	11	48	33.5	17	19	15	12.5
9078	26	15	10	90	22.5	19.5	8	20	11.5
9079	26	13.5	15	111.5	18.5	16	10	21.5	14
9080	19	11	9.5	36.5	20	13.5	15	12	10
9081	53	15	25	86	27	18	8	34	12
9082	25	13	15	109.5	27.5	16	10	18.5	24
9083	35.5	16	47	65	25	33.5	10	19.5	81.5
9084	28	13	12	77.5	24	18	16	20	62
9085	57	23	28	186.5	33	28	20.5	78	68
9086	26	12	12	73	25	15	10	17	16
9087	106.5	13	10.5	64.5	20.5	18	9	39.5	12
9088	22.5	11.5	9	38	22	13	8	13.5	8
9089	26	13	10.5	65.5	25	17	9	18	11
9090	21	12	13	80	17	14	8	13.5	39
9091	23	15	13	71	24	17	10	19	26.5
9092	24	13	13	58	20	14	9	18	27
9093	33	22	19	71	22	27	10	18	35
9094	22	13	12	78	21	14	10	16	43
9095	17.5	11	14	87.5	16	11	8	17	34
9096	93.5	20.5	17.5	107.5	24	45	17	27	18
9097	8.5	7	4	9	7.5	9	5	8	5
9098	8.5	8.5	5	11	8	8	6	11	5
9099	24	12	11	43	23	18	9	27	13.5
9100	31	12	12	89.5	21	17	9	18	12
9101	40	13	12	39	21.5	32	7	14	17
9102	104	21	23	123	456.5	24	23	31	33.5
9103	24	15	12	68	26.5	17	11	18	14
9104	14.5	8	8	28	18	11	7	10	7
9105	11	9	5	19	18.5	8.5	6	10	6
9106	17.5	9	7	33	15	12	9	14	9
9107	14.5	10	5	21	19	11	6	13	6
9108	13	9	6	19	17	9	6	10	7.5
9109	26.5	15	8	24	20	23	7	13	16
9110	19	10	11	55	23.5	11	8	14	13
9111	94.5	11	9	70	20	12	9	20	30
9112	40	14	19	100	29.5	19	26	39	31
9113	18	10	8	52.5	23	15	7	14	49.5
9114	25	13	15	17	29	16	10	13.5	10
9115	26	12	10	24	23	14	8	12	11
9116	35	15	22	108	27	18	25	30.5	19

9117	36	27	22	114.5	22	24	28	39	22
9118	32	14	21	119	29.5	16	21	109	169
9119	82	28	63	102	38	28	27	75	85
9120	99	11	13.5	87	29	16	14	67	11
9121	26	10	13	71	19	13	11.5	29.5	11
9122	71	16	21	33	31	26	14	17	21
9123	34	36	22	86	26	26	19	41	40
9124	21	10	10	88.5	14	16	6	18.5	16
9125	27	11	15	76	19	18	9	16	12
9126	47	40.5	29	103	51	30	23	40	56
9127	36	31.5	25	99	26.5	23	17	41	45
9128	53	58	34	115	30	41	25	62	57
9129	56.5	49.5	32	123	29	48	24	58	65
9130	32.5	19	21	73	22	15.5	11	23	26
9131	35	17	17	96	33.5	23.5	9	24	18
9132	34.5	21	23	109.5	19	23	14	65	15
9133	38.5	52	25	111	24	28	22	77	59
9134	26	9	17	83	21	12	9	33	88
9135	27	38	21	79.5	25.5	24	18	40	36
9136	35	40	24	106	29.5	33	27	41	45
9137	42	34	24	98	25	22	16	35	46
9138	24.5	12	12	70	17	13	15	20	27.5
9139	28	22	16	83	30	19	14	28	48
9140	29	19	17	112	21	18	12	30	39
9141	27.5	22	15	78	18	20	12	27	51
9142	37	32	24	83.5	24	25	17	34.5	59
9143	43.5	59	33	177	33	44	29.5	63	59
9144	24	20	19	95	21	16	11	29	30
9145	30	24	22.5	88	21	22	14	34	35
9146	39	38	20	86.5	26	37	22	38	38
9147	22.5	18.5	13	82	20	16	10	24	32
9148	58	37	24	111	22.5	29	17	48	49
9149	33.5	16	16	87	22	17	13	27	28
9150	23	15	21	100	18	15	9	26	38.5
9151	50	35	30	146	34	27	24	41	31
9152	30	41.5	23	87	24.5	29	19	42	40
9153	31	17	13	83	18	21	11	21	19
9154	31	22	21	104	21	16	22	33	73
9155	24	25	22	80.5	31	18.5	13	27	37
9156	34.5	36	20	87	20	26	16	44	62
9157	24	27	20	79	25	21	13	38	28
9158	34.5	34	27	96.5	26	28	20.5	41	39
9159	21	11	12	75	17	14	7	18	88
9160	29	11	18	94.5	19	13	11	20.5	24
9161	56	33	15	67	18	44	11	26	30
9162	58	23	26.5	92	18	18	11	28.5	31
9163	22	9	14	57.5	14	11	8	16	11

9164	25	10	15	106	18	15	9	27	27
9165	24	12	13	69	15	17	10	16	28
9166	22	9.5	11	74	15	14	7	16	9
9167	24	13	14	72	19	11	9	20	17
9168	29	8	12	52	17	13	7	14.5	10
9169	37	30.5	23	109	35	24	29	40.5	43
9170	44	51.5	31	93	31.5	34	24	51	72
9171	31	17	14	73	20	17	11	25	18
9172	67	28	16	66	21.5	56	11	27	25
9173	34	19	16	92	20	14	16	37	34
9174	34	31	18	78	21	32	17.5	70.5	47
9175	25	16	16	75	22	15	12	23	23.5
9176	45	57.5	38	126	38	35	60.5	67	64
9177	79	62	34	101.5	37	61	25	57	73
9178	49	36	41	108	29	29	25	44	37
9179	127	49	42	150	35	41	60	58	60
9180	47.5	21	38	129	38	28	16	34	39
9181	59.5	75	46	135	45	57	35	78	186.5
9182	35	39	26	129	30	28	18	38	71
9183	63	23	19	97	25	60.5	10	29	27
9184	41	20	23	144.5	31	20	12	26	46
9185	58	30	41	132	53	42	50	36	56
9186	45	23	21	83	26	26	14	78	34
9187	104	26	21.5	86	23.5	35	17	37	87.5
9188	26	11	13	36	31	13	16	26	12
9189	23	13	13	59.5	23.5	13	8	16	15
9190	45	28	30	148	33.5	31.5	20	47	58
9191	41	12	16	64	89	18.5	10	28	31
9192	23	14	9	42	24	19	10	13	11
9193	23	18	20	81	23.5	17	20	16.5	13
9194	30.5	16.5	16	105.5	26	20	314	23	35
9195	17	11	14	70	18	12	7	22	19
9196	27.5	10	16	60.5	23	16	8	18	68
9197	29	23	21	58	26.5	16	13	32	41.5
9198	27	11.5	11	24	24	15	9	13	14
9199	39	24	16	82	68	27	13	35	24
9200	29	21	15	61	20	20	8	20	64
9201	23	14	14	48.5	28	17	8	14.5	10
9202	22	18	15	41.5	123	18	9	21	51
9203	33	14	13	72	22	17.5	9	24	10
9204	25	11	13	45	28	12	8	19	22
9205	19	10	10	34	18	10	8	12	93
9206	39.5	14	17	77	31	17	14	25	37
9207	126.5	12	13	54	28.5	15	16	108	19
9208	26	15	24	88	20	22	9	18	39
9209	44	54	39	115	37	39	28	61	72
9210	28.5	17	19	153.5	30	19	14	23	23

9211	149.5	71	116	262	749	75	55	73	118
9212	58	16.5	13.5	69	25	48.5	7	16	17
9213	29.5	47	28	90	25	28	21	49	52
9214	-1	43	25	148	22	11	13	21.5	33
9215	24	20	20	150	21	18	14	33	53
9216	28.5	12	18	176.5	20	-1	13	26.5	-1
9217	-1	27	27	-1	20	18	21	-1	52.5
9218	36	28	27	89	26	31	13.5	31	29
9219	37	25	23	111.5	25	22	12.5	80	29
9220	38	40	28	112	31	24	21	53	50
9221	203	16	29.5	77	24	29	9	21.5	25
9222	29	11	23.5	91.5	25	14	11	25	27
9223	28	14	17	97	22	15	10	27.5	25
9224	30	27	22.5	111	22	21	13	35	39
9225	32.5	34	22	103	27	24.5	18	42.5	31
9226	36	38	28	104	24	23	76	43	47
9227	30	33	22	114	23	24	13.5	42	56
9228	27	40	24	111	28	23	17	44	46
9229	15.5	8	10	76	14	10	7	16	21
9230	32.5	37	26	116	25	27	18	41.5	46
9231	26.5	19	14	65	18	21	8	20	30
9232	41	13	16	88.5	22	12	10	69	40
9233	61	12	15	84	22	15	8	19.5	24
9234	28	12	15	92	20.5	16	10	20	23
9235	63	15.5	15	108	23	14.5	10	28	62.5
9236	31	37	29	110	26	23	16	42	47
9237	33	48	27	119	27	27	22	51	49
9238	36	55	33	113	31	33	27	55.5	65
9239	30	45	25	84.5	23.5	32.5	18	42	47
9240	31	31	24	97.5	26	22.5	18	39	63
9241	34	14	19	76	18	16	12	31	20
9242	44	22	29	139	31	24	15	38	40
9243	34.5	17	26	92	22	21	20.5	29	24
9244	30	15	16	75	22	22	9	22	36
9245	42	19	20	91	23.5	24	13	29	81.5
9246	23	12	20	83	33	14	9	22	36
9247	21.5	13	17	82	17.5	14	9.5	31	27.5
9248	38	33	24	98	26	32	17	44	39
9249	49.5	25	20	109	24.5	42.5	13.5	32	34.5
9250	41	22	23	121	38	25	27	45	34
9251	50	62	34	113.5	37.5	36	28	61	73
9252	42	31	26	110	31	24	19.5	51.5	38
9253	23	11	12	130	18	14	8	25	18
9254	32	43	29	106	27	30	20.5	57	43
9255	80	28.5	17	99	24	45	13	26	68
9256	44.5	62	44	173	33.5	43	30	68	68
9257	44	58	31	162	28	40	26	63	51

9258	36	32	25	110	25	26	20	35.5	34
9259	65.5	47	39	137	36.5	40.5	38.5	60	80.5
9260	29	34	23	93	22.5	25	17	38	66
9261	15.5	9	8	69	15	11	7	20.5	7
9262	24	12	18	87	20	10	9	25	10
9263	20	12	17	94	19	11.5	10	30	38
9264	20	10	15	94.5	20.5	14	9	16	13
9265	14	7	6	35	14	9	6	9	6
9266	21	11	10	112	17	11	7	17	8
9267	35	15	15	84	22	19	10.5	27	38
9268	10	6	5.5	13	14	7	5	9	4
9269	17	7	11	75.5	16	9	7	14	8
9270	55	18	23	99	30	23	14	99	33
9271	185	17.5	24	53	38	20	19	141	17
9272	28	19	20.5	89	27.5	20	12	32	148
9273	115	37	39.5	267	45.5	63	27	214	520
9274	43	21	23	142.5	27	23.5	14.5	42	106
9275	8	7.5	4	9	7	7	5	9	4
9276	27	10	10	111	17	13.5	10.5	29	7
9277	28	12	15	73	21	17.5	10	25	11
9278	20	10	9	73	18.5	18	8	18	7
9279	54	26	22	136.5	29.5	30.5	15	30.5	60
9280	54	13	10	59	24	15	18	100	20
9281	25	14	12	157	22	17.5	9	17	8
9282	21	9	8	61.5	18	12.5	7	11.5	19
9283	14	8	9	53	21	10	8	13	7
9284	13.5	9	7	46.5	17.5	11	7	14	7
9285	17	9	10	51	25	12	11	17	8
9286	31	20	16	57	20	26	10	16	22
9287	135	21	19	91	25.5	29	15	28.5	35
9288	34	26	20	68.5	20	26	14	25	25
9289	53	48	32	81	29	44	30	37.5	88
9290	48.5	65	35	79	36	59.5	27	51	87
9291	56	47.5	25	68	26.5	40	21	38	67
9292	33.5	19.5	16	73	25	25	11	45	27
9293	61.5	75	35	139.5	33	54	24	65	70
9294	44.5	31	22	94.5	24	27	13	41	27
9295	38	23	30	76	21.5	30	14	25	31
9296	36	38	24	68.5	29	32	19	38	46
9297	40.5	25.5	25	91	26	26.5	27.5	33	80
9298	39.5	36	51.5	87	31	31	18	40	42
9299	51	32	27.5	90	26	34	18	38	164.5
9300	36	25	21	73	24	25	13	32	42
9301	56.5	48	29	98	25.5	51.5	20	40	55
9302	47	54	38	99	34	42	30	51	60
9303	22	18	14	53	20.5	22	10	22	41
9304	53	54	31	99	36	38	27	66	68

9305	44	52	41.5	94	27.5	38.5	25	48	73
9306	58.5	27	20	89.5	24	29	19	29	44
9307	39	22	19	84	34	26.5	12	33.5	25.5
9308	27	20	16	72	19.5	23	9	17	23.5
9309	159	63	40	110	31	47	30	54	59
9310	54	38	37	97	28.5	31	19	42	48
9311	38	28	21	72	28.5	27	15	30	38
9312	30	34	25	81	23	30	16	33	42
9313	42.5	41.5	27	80	37	34	22	39.5	52
9314	27.5	22	17	63.5	22	25	12	24	26
9315	31	31	23	78	23	27.5	16	28	34
9316	53.5	51	32	88	30	37	24	48.5	50.5
9317	87	18	21	99	25	25	15	39.5	28
9318	32	16	19	72	19	22	11	23	20
9319	44	61	32	109	34	39.5	43	55	83
9320	53	28	21	82	35.5	27	17	37	132.5
9321	40	39	28	94.5	51.5	36	21	42	91
9322	32	21	17.5	87	23	22.5	12	23	30
9323	31	23	18	77	23	22	14	27	23
9324	34	20	13	51	18	27	13.5	18	27
9325	29	25	20	76	19	23	14	26	27
9326	26	18	15	77	17	22	13	20	29
9327	84	70	31	117.5	32	106.5	23	47	73
9328	45.5	56	32	91	29	34	25	50	69
9329	31	18.5	18	67	25.5	23	14	23	24.5
9330	28	18.5	17	59	22	23	12	28	28
9331	51	60	31	102.5	39.5	38	25	56	59
9332	17	12	9	26	17	17	13	13	11
9333	81.5	36	30.5	144.5	26	35	23	71	36
9334	38	25	18	68	22	26	15	26	44
9335	23	15	10.5	43	20	17	9	19	14
9336	43	22	34	129.5	28	28	32	37.5	32.5
9337	29	15.5	17	97.5	27	20	25.5	24.5	21
9338	30	17	15	67	26	20	19	16	20
9339	27.5	19	13	35	19	24.5	12	18	20
9340	28	20	19	73	21	21	13	17	45
9341	25	15	14	42	18	20	23	21	20
9342	31	14	14	36	19	20	13	12	14
9343	33	18	15	35	29	21	50	28	19
9344	34	16	14	49	18.5	18.5	14	40.5	15
9345	31	17	13	45.5	23	22	18	30	15
9346	28	16	14	82	21.5	19	11	28	12
9347	52.5	38	19	71	24	29	19	34	54
9348	17.5	11	9	43	17	15	8	12	10.5
9349	41	13	11	153	18	20	8	21	9
9350	24	13	14	68.5	24	16	38	21	13
9351	57	79	14	63	20.5	19	16	20	20

9352	31	16	13	38	18	19	11	40	12
9353	36	15	16	93	24.5	22.5	17	24	19
9354	33	16	14	88	23.5	20	22.5	24	239.5
9355	35	16	17	65	24	23	14	23	20
9356	31.5	14	14	38	22	23	10	24	22
9357	30	23	13	39	22	21	12	22	70.5
9358	51	26	19	54	37	22	33	42	28
9359	23	16	12	52	13	18	9	15	32
9360	25	16	13	66.5	24	19	8	18	17
9361	19	12	8	25	15.5	17	9	12	11
9362	20	13	9	20	17	17	11	12	9
9363	67.5	28	39	99	43.5	28	21	62	29
9364	28	12	10	35	24	18	12	29	11
9365	21	16	11.5	43	20	19	9	18	20
9366	55.5	14	14	44	26	20	17	23	19
9367	23	12	9	57	16	16	7	13	11
9368	25	16	14	68.5	19	19	12.5	23	43.5
9369	19.5	18	10	45.5	16	19	9	19	19
9370	43	23	24	89	30	29	15	64	38
9371	50	19	28	127	24	21	17	41	44
9372	24	12	11	42	18	16	8	14	11
9373	23	13	18	70	19	16	11	17	21
9374	26	15	15	54	19	19	10	55	15
9375	34	17	17	94	20.5	22	11	27.5	60
9376	28	12.5	15	73	19	16	10	18	29.5
9377	27	21	14	65	18	17	11	23	20
9378	35	24	18	76	20	19	15	29	32.5
9379	29.5	15	17	87	19	16.5	10.5	24	19
9380	24.5	16	16	76	15.5	18	8	24	22.5
9381	20	12	14	78	19	15	8	21	35
9382	27.5	21	17	65	19	17	10	31	28.5
9383	28	18	19	81	19	16	10	23	30
9384	37	22	19	84	19	21	13	29	33
9385	43.5	20	15	77.5	16	17	9	22	32
9386	22	12	10	33	15	14	8	12	11
9387	24	13	14	65	18	15	8	18	17
9388	55	16	17	67	66	18	14	77	57
9389	51.5	22	26	123	29	24	15	46	111
9390	70	21	18	42	28	37	18	34	34
9391	25	14	11	41	19	16.5	9.5	23	11
9392	33	15	15	27	55	19	12	22	18
9393	39	33.5	22	73	20	29	17	33	61
9394	27	17	15	59	15	16	9	20	27
9395	36	34	21	92.5	26	27	19	33	47
9396	70	66	37	98	41	42	33	63	72.5
9397	33	46.5	27	85	27	28	23	49	55
9398	49	45	26	87	30	35	24	67	59

9399	26	15	13	50	17	16	11	23	21.5
9400	31	34	21.5	74	23.5	25	17	46	41
9401	33	30	19	70	20.5	22	15	34	39.5
9402	36	41	26	72.5	27	25.5	20	48	44.5
9403	47.5	65	36	102.5	80	39	28	65.5	70
9404	29	31	19	59.5	22	21	16	34	33
9405	28	25.5	18	78	19.5	21	16	31	35
9406	30	16	24	62.5	19	18	13	26	28
9407	28.5	17	16	72.5	20	18.5	10	24.5	56.5
9408	49	46	27	96.5	34	46	23	53	50
9409	43	60.5	35	104	37	40.5	28	58	60
9410	43.5	33	22	85.5	25	23	16.5	38	111
9411	26	23.5	17	68.5	20	20	14	26	36
9412	34	35	22	98	24	23.5	17	45	35.5
9413	25	16	16	63	23	18	11.5	21.5	24
9414	31	13.5	14	66	18	17	10	20	18
9415	43	44	31	90.5	33	35	25	49.5	61.5
9416	31.5	40	23.5	81	30	30.5	20	43	50
9417	34	10	11	58	15.5	15	8	16	15
9418	29	28	21	55	18.5	20.5	15	29	28
9419	27	15	15	59	18.5	15.5	11	22	19
9420	32	39.5	24	76.5	24	27	20	41	42
9421	33	27	16	81	25	19	15	29	44
9422	29	13	15	61	19	18.5	10	22	24
9423	64.5	61	34	94	34.5	49	28	60	74.5
9424	27	18	13	53.5	29	17	11	24	31
9425	54.5	51	27	83	27	35	23	56	74.5
9426	43	40	25	81.5	47	33	21	41	63
9427	39	46	39	93	52	29	24	45	48.5
9428	40	48	29	112.5	39	38.5	23.5	51	57
9429	33	39	23	80	30	28	19	43	50
9430	42	33	26	68.5	33.5	27.5	20	41.5	36
9431	32	33.5	24	92.5	22	32	18	50	35
9432	41	47	29	79	31.5	28	60.5	50	49.5
9433	53	79	40	107	35.5	50	35	71	82
9434	28	29	18	57	21.5	26	15	30	36
9435	30	38	23	73	22	25	17	37	46
9436	45.5	28	22	75.5	24	31	16	34	32
9437	47	58	30	88.5	32	38	22	55	56
9438	34	22	18	57	18	29.5	9	20	48
9439	29	14	14	71	20	19	10	20	21
9440	27	16	17	68	17	17	17	23	24.5
9441	29.5	15	14.5	72	17	19	12	24	18
9442	20.5	11	13	53	15	14	7	15	15
9443	36	20	26	99	24	21	14	29	64
9444	35.5	24	19	79	26	21	15	29	34
9445	22	11	12	53	16	13	6	13	19

9446	40	43	32	75	37	28.5	25.5	50	47
9447	33	36.5	26	78	30	26	17	36	46
9448	30	35	21	79	24	24	18	36	53
9449	33	24.5	14	55	34	16	30	28	20
9450	52	11	10	39	32	13	11	89	10
9451	24	18	18	62.5	27	15	16	16	19
9452	86.5	14	14	32	20.5	17	17	21	15
9453	37	13	10.5	37	16	17	9	21	12
9454	36.5	32	15	49	27	19.5	26	19	20
9455	26	13	12	42	17	17	12	65	21
9456	27	13	10	48	15	20	7	11	9.5
9457	29	17	11.5	51	22.5	21	14	17	17
9458	33	22	27	58.5	33	23	16	48	20
9459	183	42	57.5	192	288.5	51	44	154.5	48.5
9460	262.5	322.5	80.5	377	69	189	47.5	426	269
9461	68.5	20	22	37.5	26	29	12	22	16
9462	48.5	16	11	44	22	37	11	15	16
9463	43	23.5	23	63.5	42.5	28	20	40.5	49.5
9464	44	24	23	73.5	40	38	22	29	58
9465	41	23	36	91	36	26	22	34	27
9466	18	16	11	51	19	18	10.5	14	11
9467	21	16	10	48	19	18	10.5	12	11
9468	22	18	18	52.5	22	20	10	13	13
9469	19	14	10	42	16	19	9.5	12	11
9470	41	20	12	66	23	43	10	16	15.5
9471	19	16	13	43	20	20	9	13	11
9472	29.5	18	14	57	20	27	11	25	15
9473	23	14	11	52	19	31	12	78	11
9474	17	12	10	73	17	16	7.5	12.5	11
9475	16	13	10	42	16	17	9	13.5	9
9476	16	12	10	46	24	15	9	12	10
9477	17	13.5	9	31.5	18.5	18	8	16	10
9478	47	54	24	88.5	52.5	42	24	48	55
9479	52	44.5	30	85	28.5	40	22	50.5	44
9480	43	57.5	32	86	33	49.5	28	62	59
9481	35.5	52	27	76	33	28	22	48	43.5
9482	70	60	28.5	80	33	62	23	56	121
9483	36	74	53	88	56	39.5	35	74.5	78
9484	49	65	34	82	40	38.5	22	64	71
9485	50	80	40	105	48	47	33	77	75
9486	26	17	13	69	18	23.5	10	21	15
9487	54	52.5	38	97	41	44	35	68	55
9488	38	48	29	72.5	32	33	26	51	45
9489	44.5	60.5	33	99.5	35	46	30.5	57	71
9490	47	72.5	46	95	36	49.5	32	71.5	75.5
9491	40.5	13	14	61	20	21	10	18	19
9492	25	20	14	61	23	22	18	19	23

9493	25	21	16	70	21.5	21	10	21	26.5
9494	49	60	31	83.5	35	39	27	58.5	82
9495	34	17	14	69.5	18.5	30	11	18	16
9496	54.5	34.5	25	79	29	28.5	19.5	43	48
9497	46	45	27	83	30.5	37	25	43	45
9498	34	24	19	74	25	23.5	14	24	32
9499	52	51.5	31	116.5	42.5	39	27	59.5	56
9500	52	81	31.5	92	40.5	49	35.5	69.5	87
9501	31	27	20	68	23	21	14	30	29
9502	48.5	74	37	142.5	38	49	34	72	72
9503	43	56	32	93	29	38.5	26	59.5	68
9504	42	45.5	27	102	30	34	23	44	50
9505	32	33	20.5	97	23.5	28	17	33.5	44
9506	39	70	32	101	31	44	30	69	101
9507	13	13	21	30	15.5	14	9	12	8
9508	21	19	10	34	15	19	10	15	12
9509	23	19	18	53.5	18	19	15	20	31
9510	15	12	9	43	15.5	13.5	6	12	9
9511	22	16	12	51	18	17	11	17	34
9512	26	15	17	82.5	19	18.5	11	25	20.5
9513	25	19	16	67.5	20.5	20	11	22	44.5
9514	17	15	11	63	18	15	9	16	13
9515	20.5	13	13	57	21	16	10	15	12
9516	21	14	18	56.5	19	16	9	20	20
9517	34	13	15	66	30.5	17	10	24	33
9518	21	120.5	12	41	15	15	7	16	67
9519	31.5	14	15	104	20	18	13	43	14.5
9520	38	47.5	14.5	53	21	28.5	12	41	38.5
9521	33	19	16	72	70.5	30	20	21	43.5
9522	19	12	21.5	83	19.5	13	25	19	11
9523	20	11	9.5	50	16	14	8	12	12
9524	19	13	9	47	18	18	7	13	14
9525	24	12	15	61	51.5	16	30	17	21.5
9526	20	18	13	44	21	16	14	38	56
9527	16	10.5	8	44.5	16	13	8	21.5	8
9528	25.5	25	18.5	54	18	19	13	25	22
9529	12	10	5	16	13.5	14	11	11	7
9530	93	12	12	67	19	17	12	26	11
9531	23	21	14	33.5	40	26	11	14	16
9532	52	60	39	106	38.5	50	25	53	94
9533	66.5	82	45	118	50.5	60	42	70	75
9534	49	63	41	138.5	45	45.5	30	64.5	71
9535	53	60.5	35	105	43	52.5	30	53	66
9536	85	75	41.5	105	46	58.5	36	67.5	86
9537	77	29	23	60	28.5	66	12	22.5	37
9538	67	55	33	114	36	61	26	52	62.5
9539	33	23	16	58.5	23	31	12	19	73

9540	42	56	31	92	31	39	25	50	56
9541	45	35	23	95	29	33	18	36	32
9542	278	43.5	30	74.5	32	73.5	22	38	38
9543	35	19	18	85	33	29	11	20.5	44.5
9544	49	62	34	86	41	49	30.5	59	76
9545	89	39	26.5	68.5	37	45	25	44	118
9546	49	78.5	39	98	35	52	29	65	109
9547	37.5	41	29	61	28	37	19	33	56
9548	35	38	24	75	26	32	17	37	51
9549	48	40	28	87	37	38	20.5	39	54
9550	42	33	24	67.5	32	31	19	32	36
9551	36.5	36	30.5	83	46	33	18	36	44
9552	42	47	29	66	33	39	23	44	82
9553	63	66	33.5	173	31	58	22	55	60
9554	56.5	31	23	74	33	35	27	34	36
9555	38.5	20	20	86	29	25	12	30	43
9556	82.5	56	30	89	40.5	72	22	45	52
9557	62	85	45	103	43	57	37	74	93
9558	54	76	36	100	49	55	30	59	83
9559	36.5	25	19	80	25	28	16	31.5	74.5
9560	39.5	23.5	23	71	34	31	16	29	45
9561	97	25	21	58	26	42	14	26.5	43.5
9562	89.5	41	26	66	52	67.5	18	31	71.5
9563	47	19	18	60	23	29.5	14.5	32	29
9564	52	64	39	94	43	47.5	31	71	69
9565	46	54	31	90	34.5	42.5	27	60	61
9566	53	88.5	54	106	39	51	32	65	111
9567	77	66	38	168.5	41	57.5	31	57	81
9568	28	16	19	68.5	20	23	10	25.5	27
9569	36	19	18.5	95	32	25	18	29	21
9570	32	19	18	91	32	23	16	22	21
9571	140.5	27	53	164.5	36	33	29	84.5	30.5
9572	20	14.5	9	39	19	19	8	12	14
9573	25.5	14	11.5	32	25	21	14	18	39
9574	24	16	10	35	28	22	10	18	14
9575	40.5	20	21	80	28	27	18	21	31
9576	25	14	14	40	25	20	11	14	18
9577	52.5	19	19	34	44	28	14	18	26
9578	71	20	17	48	38	25	15	116.5	19
9579	32.5	20	16	49	31.5	29	20.5	113	93
9580	48	42	19	60	27.5	48	15	28	44
9581	28	16	15	61	27	21	10	18	18.5
9582	45	22	24	74.5	48	29	15	51	176
9583	23	14	11	32	23	20	10	127	11
9584	25	13	18	39	18	20	7	11.5	11
9585	23	14	10	34	24	19	11	28	24
9586	23	12	9	51	23	17	7	14	11

9587	39	22	13	46	34	24	10	51	15
9588	23	13	10	38.5	25	18	8	12	12
9589	30	15	12	57	27	22	21	26	20
9590	30	15.5	14	30	31	20.5	12	21	22
9591	164	25	34	49	36	30	20	30	25
9592	43	17	20	32	31.5	23	15	17	16.5
9593	74	30	23	85	48	28	31	64	27.5
9594	30	15	12	37	30	20.5	14	76.5	25
9595	172	70	77	288	249	73	55	147	96
9596	30	18	13	33	32	25	12.5	98.5	15
9597	22	14	10	44	19	19	8	14	12
9598	22	13	10	38	21	19	10	12	13
9599	30.5	17	23	88	25	22	11	58.5	20
9600	47	18.5	13.5	70	25	22.5	18	24	15
9601	33	14	16	34	26.5	17.5	9	15	10
9602	25	18	12	52	22	18.5	9	15	16
9603	156.5	27	20	44	47	99.5	15	31	47
9604	29.5	15	15	71.5	56	20	19	25	22
9605	34	16	24	89.5	29	23	12	45	24
9606	20	15	11	18	23	18	10	13	10
9607	34	18	19	56.5	29	20	86	59	16
9608	57	14	12	61	26	31.5	9	35	17
9609	35	16	30	62	39	27	27	31	20.5
9610	187	169	54	227	47	101.5	332	142	136
9611	36	19	17	45.5	196.5	27	15	24	24
9612	14	11	6	11	8.5	15	6	8	7
9613	12	11	5	10	9	14	5	7	7
9614	28	13	14	76.5	23.5	22	17.5	21	14
9615	52.5	15	15	61	21	43	9	18	20
9616	29	13	11	89	23	18	22	21	16
9617	20.5	13	12	61.5	18.5	18	10	14	12
9618	34.5	20.5	22	281.5	29	27	21	43	16
9619	40	19	39	192	90	28	76.5	43	16
9620	23	15	25	86	28	19	65.5	22	50
9621	21.5	13	11	43	26.5	18	9	16	17
9622	31	15	18	93	27	19	11	30	26
9623	30.5	14	15	64	117	16.5	18	18	20
9624	144.5	60	107	360	52.5	63.5	35	273	79
9625	37	15	12	44.5	21	19.5	11	17	14
9626	45.5	36	45	94.5	32.5	29	28	39.5	56
9627	181	24	27	71	43	36.5	22	53	33
9628	35	14	18	58	26	17.5	19	18	17
9629	27	13	13	70	27	19	9	18	18
9630	23	13	28.5	76	22	19	10	25	16
9631	25	18	15	62	30	18	9	25	26
9632	29	16	19	43	25	17	10	30	34
9633	33.5	18	19	87	34	20	16	33	48

9634	59.5	15	13	33	24	33.5	10	16	27
9635	34	24	31	39	28	29	10	22	18
9636	60	18	14	38	27	24	14	20.5	18
9637	61	26	36	94	51	33	34	47	30
9638	53	28	22	66	28	24	20	48	36
9639	27	21	23	47	24	22	12	23	23
9640	37	15	14	69	23	20	9	23	32
9641	36	15.5	13	77	17	19	10	19	20
9642	41.5	34	24	59.5	29	30	20	36	45
9643	35	25	19	62	153	26	13	28	29
9644	36	52	31	80.5	29	31	38.5	53	46.5
9645	27	17	15	52	23.5	23	10	23	16
9646	34	48	26	84	29	33.5	22	46	47
9647	34	37	22	68	25.5	31	19	37	40.5
9648	28	18	18	60	22	21	23	26	48
9649	31	15	16	52	22	19	9	27	31
9650	22	12	11	62	17.5	14	8.5	16	49
9651	25	12	14	51	20.5	16	15	17	33
9652	35	30	19	68	29	25	16	33	45
9653	25	13	11	51	22	17	10	15	37
9654	34.5	28	17	66	26	26	15	33	25
9655	35	16	17	63	21	28	11	19	30
9656	29	25	21	67	22	22.5	14	29	43
9657	33	28	18	81	23	23	14	28	36
9658	59	34	30	77	36	58.5	10	22	52
9659	19	11	13	43	17.5	12	8	16	10
9660	322	40	21	91.5	28.5	108	13	24	50
9661	79	25	14.5	49	21	71	18	17.5	33.5
9662	79	38	22	79.5	35	64	15	37	37
9663	39	15	17	51	26	17	11	25	23
9664	44	38	23	79	29	34	24	44	45
9665	23	13	13	56	19	19	9	17	21
9666	44	57	34	77	77	39	27	74	64
9667	45.5	45	27	70.5	27	31	21	41	65.5
9668	22.5	13	16	66	88	16	11	19	47
9669	34	18	15	60.5	22	26.5	10	17	26
9670	40	30	34	85	27	26.5	21.5	36	41
9671	28	29	18	51	20	23	17.5	30	35
9672	30	18.5	18	76	22	21.5	11	25	17
9673	23.5	12	13	60	18	16	8	17	67
9674	27	26	24	80	23	22	14	30	34
9675	53	24	16	63	18	53	11	30.5	36
9676	51.5	69.5	41	105	35	43	31	82	93
9677	41	12	13	70	23	16	10	40	45
9678	40	46	26	90.5	30.5	35	22	45	54
9679	24	15	14	58	16	17.5	10	19	16.5
9680	32	37	22	72	23.5	28.5	15	37	47

9681	21.5	13	10	37.5	16	17	13	14	18
9682	26	18	17	68	25	23	17.5	59	67
9683	22	11	13	61	23	16	22	17	14
9684	38	37	30	95	31	31.5	24	43	49
9685	54	49	33	90	35	45	25	51	56
9686	32	14	20	53	39	19	12	26.5	20
9687	25	10	12	42.5	17	14.5	9	19	18
9688	57	50	79	115.5	53.5	34.5	28	73	69
9689	49	13	15	70.5	19	16	11	23.5	19
9690	41.5	57	30	154.5	32	36	22	51	60.5
9691	28	14	14	43	21	19	8.5	19	14
9692	24	11	14	77	17	16	9.5	22.5	31
9693	22	16	15	74.5	30	18	11	20	25
9694	31	17	13	27	24.5	20	11	18	28
9695	41.5	22	25	66.5	32.5	34	16	35.5	21
9696	32	13.5	14	38	35	17	9	17	48
9697	16	13	9	36	29	17	8	11	10
9698	41	15	19	56	40	20	12	19	20
9699	80.5	33	12	22	37	81.5	16	16	19
9700	45.5	15	11	24	31	18	11	17	12
9701	31.5	13	11	19	27.5	16	8	12	16
9702	23	14	9	46	23	18	19	36	11
9703	30	13	12	52.5	24	16	9	40	14
9704	860	17	18	131	32.5	23	13	30	59
9705	245.5	19	34	66	34.5	27	22	27	20
9706	21	12	13	51	30.5	14	14	18.5	9
9707	16	10	8	39	27	15	6	9	13
9708	28	10	8	34.5	20	14	7	11	13
9709	35	13	11	97	89	16	16	40	10
9710	15	10	8	41.5	28	14	6	11	7
9711	99	19	24	85.5	211.5	28	15	38	19
9712	40	15	13	42	38	18	12	33	14
9713	29	13	10	40	30	16	9	21	11
9714	24	13	19.5	51	30	16.5	23	84	13
9715	20	11	9	35	21.5	13.5	6.5	11	8
9716	16	10	6	38	27	15	6	11	7
9717	15.5	10	9	35	19	13	7	43	9
9718	31	29	21	67	26.5	22	20	38	44
9719	26	31	20	76	34	26.5	15	47	43
9720	21	13	14	60	21	16	9	28	38.5
9721	25.5	17	14	60	25.5	18	10	22	22
9722	29	31	21	84	29	24	17	35	49
9723	24	19	14	52	21.5	21.5	10	19	41
9724	50	55	27	81	30	43	22	47	52
9725	36	36	23.5	72	25	27	19	44	38
9726	34	67	28	81	33	34	24	62	65
9727	34.5	50	26	89	42	33	27	47.5	55

9728	37	43	28	86	39	31	24	42	85
9729	34	54.5	28	101	31	32.5	22	56	59
9730	29	12	14	61.5	25.5	16	8	23	18
9731	52	46	23	111	26	45.5	13	36	44
9732	27	39.5	22	62	24	28	16	39	43
9733	30	22	16	58	24.5	32	10	21	33
9734	22.5	25	18.5	59	24	18	13	28	38
9735	17	13	11	51	20	16	8	17	15
9736	36	22	22	73	38	18	13	53	26
9737	18	12	13	65.5	23.5	14	8	16	23
9738	23	27	16	49.5	22.5	18	11	25	23
9739	44	55.5	27	85	31.5	45	22	60	54
9740	35	53	27	76	34	34.5	24	49	76
9741	25.5	18	19	69	33	16	11	22	35
9742	26	31	24	72	25.5	25	17	34	34
9743	29.5	29	19.5	98.5	24	22.5	15	41	33
9744	28	30	21	71	26.5	24	16	32	54
9745	16	11	10	43	18	13	7	16	14
9746	27.5	39	21	63	27.5	26	16.5	35	35
9747	41	56	32	95.5	43	38	30	64	68.5
9748	44	37	20	89	25	39	13	34	32
9749	19.5	14	12	59	23	15	9	20	21
9750	26	37	23	87	29	24	18	39	46.5
9751	102	37.5	19	94	24.5	63	14	30	63
9752	31	33	24	76	25	27.5	18	37	38
9753	44	30	21	74	33	26	15.5	39	51.5
9754	45.5	60	33	91.5	36.5	36	27	57	61
9755	35	35.5	25.5	83.5	33	30	20	48	40
9756	63.5	59	35.5	105	36	40	29	80	61
9757	23	34	29	63	26	24	15	36	32
9758	26	21	13	52	21	24	11	23.5	29
9759	25	30	19	64	25	23	16	33.5	32
9760	35	64	29	74	35	35	27	60	62
9761	31	44	26	78	26	27	18	53	38.5
9762	17	12	12	41	20	14	7	16	25.5
9763	31	38	25	79	29.5	26	19	42	60
9764	25	35.5	25.5	70	27	24	16	37	38
9765	34	38	27	87.5	35	27.5	21.5	48	47
9766	45	40	22	88	33.5	39	15	41	47
9767	28.5	27	17	82	25	25	12	25	45
9768	17	10	10	64.5	26.5	12	8	14	18
9769	58.5	11	9	49	18	20	8	26	20.5
9770	26	14	14	56	21	15	10	18.5	17
9771	18	10	12	78	21	12	9	16	18
9772	19	10	13	67.5	70	13	7	17	41
9773	21	15	15	63	23	18	10	21	24
9774	26.5	37	19	73	27	25	17	35	43

9775	37	46	26.5	134	30	33	19	71.5	42
9776	28	33	23	77	29.5	26	17	30.5	39
9777	29	18	16.5	63	30	23	11	26	17.5
9778	21	17	15	71.5	19.5	20	9	18	21
9779	58	70	43.5	100	38	59	28	57	69
9780	37	36	23	82	62	32.5	16	34	41
9781	20	17	13	56	21	21	8	21	15
9782	33	28	21	76	26	29	15	32	29
9783	35.5	33	42	105	32	32	15	33	54
9784	28	17	16	110.5	21	23	9	24	13
9785	18	14	9	31.5	24	19	7	11	11
9786	32	15	11	62	33	21.5	25	23	12
9787	39	14	10	62	26	20	7.5	14	11
9788	20	16	11	52	26	20	11	32	15.5
9789	22	16	11	45	22	19	9	16	10
9790	21	16	11	38.5	30	19	18	14	12
9791	28	14	11	69	22	19.5	12.5	33	13
9792	37	18	15	66	35	22	15	142	36
9793	33	17	13	38.5	29	22	10	22	103.5
9794	37	13	13	56	34	20	18	20	15
9795	45	20	26	68	55	30	57.5	34	48
9796	43	17	17	70	28	27	11	61.5	39
9797	27.5	17	14	60	27	21	13	20	746
9798	54	20	15.5	99	1633	23	71	25	75
9799	31.5	21	18	48.5	132	29	17.5	27.5	23
9800	29.5	15	17	57.5	135	21	18.5	19	14
9801	63	24	19	110	37	55	19.5	58	21
9802	30	13	16	110	29	20	10	35	27
9803	22	18	10	37.5	25.5	17	9	11	13
9804	17.5	12	10	57	19	15	8	12	9
9805	22	13	12	62	30	17.5	10	16	12
9806	24	19	21	41.5	26	25	10	24	13
9807	72	64.5	53	123	32	60	29	63	63
9808	41	35	24	93.5	52.5	37.5	19	37	37
9809	62.5	85.5	47	121	54	58	41	77	90
9810	50	54	28	118	35	50	22	46	66.5
9811	71	33	33	66	29	41	12.5	25	34
9812	45	33	23	90	27	33	12	27	40
9813	33	17	14	68	56	21.5	9.5	21	22
9814	30	21.5	13	79	29	27	9	22	30
9815	50.5	49	30	88	37.5	45	21	44	77
9816	36.5	25	20	110	27	31	14	30	56
9817	42	40	22.5	97.5	30	39.5	18	37	48
9818	35	26	21	69	29	30	12	31	37
9819	38	27	17	63	24	38	11	27	28
9820	69.5	39	28	108	42	43	32	56	44
9821	28	20	14	65	22	25	10	24	66

9822	39	49	25	75	28	34	21	42	36.5
9823	32	21	18	83	24.5	27	9	20	21
9824	50.5	28	20	81	23	36	16	34	37.5
9825	37.5	28	17	61.5	25.5	34	12	26	44
9826	38	22	24	70	24.5	34	11	22	25
9827	45	17.5	14	99	20.5	28	10	32	36
9828	62	21.5	16	68	51	25	13	34	35
9829	46	69	44	104	87	46	32	62	71
9830	43	33	24	80	27	34	19	44	57
9831	53	44	26	89	46	36	15	32	55
9832	35	29	18	72	36.5	32	18	27	45
9833	45	35	28	76	30	36	20	36	128.5
9834	33	19	13	60	59	24	10	19	22
9835	45.5	52	28	91	38	39	23	51	84
9836	80	30	17	80	23	73	12	28	40
9837	65	40	27.5	98.5	39	47	39	41	48
9838	30	19	16	82	42	25	11	21	26
9839	48	51.5	33	102	32	44	22	49	67
9840	31	19	14	49	22	27.5	9	16	25
9841	33.5	16	14	110	32	21	8	22.5	22
9842	42.5	51	38	95	36	43	30	57	65
9843	56	43	34	100	53	42	27	56	80
9844	43.5	41	29	104	39	37	22	50	61
9845	76	89.5	51	132	40	63	48.5	91	87
9846	80	61	29	93.5	30.5	46	25	50	55
9847	54	71	33	112	32	51	28	68	70
9848	31	20.5	23	92.5	24	28	11	27	49
9849	41	30	23	112.5	25	29	14	34	117
9850	32.5	21	18	88.5	45.5	23	16	25	92.5
9851	54	63.5	33	97	28.5	45	24	66	77
9852	35	20	20	89	28	26.5	12	24	18
9853	59	63	36	130	35	49	27	63	109
9854	36	26	18	122.5	28	31	22.5	25.5	24
9855	111	34	99	214	51	48	189	147	34
9856	18	12.5	9	66	22	16	10	14.5	24
9857	22	13	10	71	27	18.5	21	18	10
9858	238	26	15	51	39	31	21	175.5	20
9859	24	15	8	51	20	21	8	14	9
9860	105.5	31.5	28	71	61	45	55	64.5	31
9861	85.5	27	23	93	133.5	37.5	24	185	46
9862	45.5	17	14	33	27	25	12	16	15
9863	33.5	16	15	95	27.5	22	10	25	33
9864	61.5	25	30.5	127	29	37	19	41	64
9865	36	19	18	94	38.5	25	15	58	26
9866	39	19	14	33	35	29	12.5	48	17
9867	70.5	20	18	36	75.5	29	20	44	19
9868	37	21	22	90.5	190.5	27	13	37	26

9869	75	15	13	42	33	25	34.5	67	16
9870	38	11	10	43	25	18	13	18	12
9871	53	17	16	43.5	30	28	13	31	27
9872	32.5	15	17	52	18	21	8	20	12
9873	26	15.5	10	84.5	19	24.5	10	14	58
9874	23.5	15	9	36	18	23	6	13	13
9875	22	18	10	36	24	26	8	15	16
9876	22	15	8.5	52	23	21	8	13	27
9877	30	12	10	29	20	16	9	15	15
9878	70	16	15	54.5	36	31	14	20	36
9879	69.5	17	12	47	24	26	10	44	20
9880	32	14	12	30	75	19	13	19	15
9881	31	12	12	33	26	20	10	17	14
9882	1799	87	50	169	43.5	864	26	47.5	164
9883	22	12	10	40	25	19	8	24.5	22
9884	70	103	33	97	50.5	78	28	93	75
9885	76.5	26	27	56	43	29	51	48.5	28
9886	24	13	11	69	21.5	16	9	14	14
9887	30	15	19	85.5	119	23	13	26	21
9888	18	14	11	96	18	14	7	15	21
9889	23	11	13	127	21	17	7	18	10
9890	24	18	16	36	28.5	20	8	22	11
9891	68	18	13	40	25.5	27	13	69	26
9892	41.5	15	14	53.5	25	21	14	17	16
9893	23.5	12	14	38	153	18.5	11	18	74
9894	19	12	11	41.5	58	15	6	37	25
9895	52.5	10	15	169	19	16	9	103	70
9896	62	17	21	34	30	29	15	63.5	17
9897	18.5	13	27.5	29	17.5	18	7	17	8
9898	70	25.5	19.5	44	58.5	38	20	19	33
9899	22	12	9	55	16	17	7	14	15
9900	44	59	31	131.5	32.5	39	25	64	66
9901	51	78	38	104	42	52	34	77	92
9902	33	44	21	80	29.5	30	21	47.5	47
9903	17	9	11	60	18	10	8	15	15
9904	28	14.5	12.5	72	33	18	8	21	17
9905	30	20	12.5	53	26.5	17.5	9.5	28.5	22
9906	43	41	29	78	34	37	21	41	52
9907	36.5	47	24	92	26	36.5	20	46	55
9908	30	19	16	71	23	17	10.5	30.5	16
9909	59.5	71	36	122	37.5	39	39	73	90
9910	26.5	18	12	48.5	21	17	10	26	18
9911	40	24	15	66	22	28	13	29	25
9912	48	69	36	93	35	43	30	67	70
9913	39	56	28	86	30	36	30	58.5	55
9914	32	14	11	66	31	23	8	18	18
9915	38.5	50	29	111	31.5	36	24	52	61

9916	40	38	23	82	22	34	15	47	62
9917	51	44	27	90.5	39.5	35	30	48	57
9918	39	48	30	90.5	29	33.5	22	47	58.5
9919	40	31	23	89	25	26	61	36.5	39
9920	33	38	21	67	28	24	17	40	47
9921	37	45	29	78	28	32	24	47	50
9922	22.5	13	18	74.5	22	12	10	22	39
9923	38	38	25	63	32	28	17	46	35
9924	34	29	22	70	23.5	30	17	31.5	37
9925	33	40	23	74	27	30	19	42	38.5
9926	78	41.5	21	90.5	26	68.5	15	34.5	59
9927	24	15	15	70	29	16	10	22	29
9928	21	17	12	46	19	17	10	25	36
9929	41	48	27	83	25	32.5	41	46	48
9930	40	57	39	100	30	38	26	56	70
9931	54	69	41	108	43	48	27	59.5	69.5
9932	36	19	16	74	29.5	24	17	35	26
9933	24	14	11	54.5	18	21	8	27.5	24
9934	45	63	43	108	34.5	37	29	60	63
9935	28	20	15.5	67	18	17	17.5	24	42
9936	37.5	34	23	74	25	29	17	48	44
9937	32	33	23	67	28.5	22	18	37	81
9938	25.5	6	10.5	49.5	18	11	7	17	13
9939	47	46	29	77	35	34	25	44	44
9940	43.5	42	21	77	26.5	41	19	42	46
9941	27.5	14	12	73	18	19	8	19.5	23
9942	44	26	17	64	20	19	13	31	42
9943	35	46	27	77.5	29	29	21	50	69
9944	24	10	10	52	20	16.5	8	17	35
9945	29	12	12	50	18	17	9	17	34
9946	15	10	6	28	7	13	4	9	17
9947	34	34	23	91.5	23	28	16	66	54
9948	41	53	30	80	29	40	24	51	56
9949	23	18	9	42	16	17	7	12	10
9950	23	11	8	27	16	13	8	10	9
9951	25	24	9	35	19	15	7	18	13
9952	41.5	19	14	39	19	23	31	76	17
9953	24	17.5	15	104	23	19	12	20	18
9954	40	15	12	41	24	19	19	20	14
9955	44	17	11	33	21	30	12	19	17
9956	27	12	10	34	18	18	7	12	8
9957	22	27	23	38	17	16	8	13	10
9958	33	26	19	88	27	22	16	28	32
9959	32.5	16	15.5	48	36	16	37	17	15
9960	23	14	13	24	22.5	19	8	14	12
9961	66	27	43	123	45	35	17	42	68.5
9962	44	28.5	30	109	351.5	34.5	16	37	36

9963	143	26	24	62	36	40	18	45	29.5
9964	55.5	25	29	120	38	33	21	88	147
9965	52	30	37	119	-1	34	18	81	156
9966	22	16	12	71.5	21	17.5	10	18	30
9967	28	16	15.5	64	23	23	9.5	17	44
9968	30	20	26	120	25	23	15	26	22
9969	55	20	15	57	28	24	35	28	24
9970	48	22	30	99	27	29.5	15	37	22
9971	27	20	19	63.5	22.5	18	7	22	33
9972	21	18	13	58.5	26.5	20	14	17	15
9973	27	16	10.5	43.5	24	20	8.5	18	23
9974	18	13	9	49	17	17.5	7	11	10
9975	20.5	15	12	62.5	18	20	9	18	13
9976	25	14	13	65	91	21	8	16.5	16
9977	20	16	13	75	29	20	9	20.5	22
9978	34	40	24	87	29	28	18	41	38
9979	43	73	35	112	37.5	46	32.5	69	122
9980	31	34	23.5	101	44.5	31	18	35	62
9981	54	38	22	87.5	29.5	36	39	77.5	33
9982	29	35	21	85	27	28	15	39	47
9983	58.5	79	42	112.5	41.5	52	35.5	87	100
9984	41	72	43	114.5	36	43	29	68	81
9985	48.5	43.5	31	92.5	32	37	23	54.5	51
9986	37	17	18	72.5	24	23	10	23	27
9987	40	18	17	137	15	23	17	78	35.5
9988	28.5	43	28	77	28	31	20	43	55.5
9989	44	81.5	41	90.5	37	42	29	69	89
9990	30	30	25	100	29.5	25	14	33.5	34
9991	39	43.5	26	133	30	36.5	23	48	77.5
9992	42	49	30	91	35	34	25	52.5	69
9993	36.5	49.5	34	100.5	29	34	22	65	53
9994	24	15	16	87.5	24	16.5	11	23	14
9995	41	67.5	33	120	38	42	30	67	58
9996	47	59	50.5	112	36	41	25	61	74
9997	37	46	30	90.5	40	35.5	19	45	48
9998	45	74.5	39.5	137	43.5	49	32	78.5	83
9999	38.5	57.5	29.5	101	32	41	23	51.5	80
10000	36	67	31	109	36.5	38	26	67	75
10001	27	23	19	119	23	22	11	32	27
10002	41.5	57	33	110	35.5	39	26	58	78
10003	48	20	19	70.5	22	32	15	23	43
10004	21.5	12	12	54	19	18	14	17	15
10005	49	28	19	77	23	34	13	28	32.5
10006	32	13	16	85.5	22	18	11	21.5	18
10007	37	18	14	85.5	19	31	9	19	38
10008	25	31	19	83	23	19	15	36	34
10009	164	84	24	139	30.5	219.5	18	45	69

10010	31.5	28	17	73.5	22	25	12	31	25
10011	30	36	18	75.5	22	24	14	45	40
10012	25.5	14	13	67	18.5	17	10	21	17
10013	23	14	14.5	77	18	18	9.5	17.5	16
10014	17	11.5	11	53	19	15	7	14	21
10015	21	14	14	90	19	16	6	20	14
10016	128.5	18	16	43	25.5	23.5	11	46	14
10017	14	12	6	25	17	16	6	10	10
10018	18	12	9	69	19	17	6	17	12
10019	27	22	17.5	67.5	33	22	11	21	32.5
10020	54	46	28	83	26.5	31	20	61	70
10021	22	14	13	58.5	22	19	8	52.5	18.5
10022	34	14	39	33	25	17	14	103	14
10023	35	14	14	76.5	26.5	19	23	21	27
10024	21	14	13	96	21	18.5	15	18	18
10025	55	45	31	115	32	38.5	25	67	80
10026	224	24	28.5	135.5	31.5	91.5	16	45	54
10027	28.5	14	12	58	22	17.5	12	19	17
10028	16	11	11	44	33	14	25	70	14
10029	26	14.5	19	66	18	18	10.5	165	14
10030	24	12	23	63	28.5	14	9	17	13
10031	29	13	12	31	21	16	10	21	23
10032	25	15	17	87	21	22	9	38	49
10033	18	14	17	86	20.5	16	8	12	9
10034	21	13	12	55	20	19	8	17	9
10035	39	24	14	48	31	31	12	17	22
10036	59	37	32	139	35	37	24.5	57	58
10037	30.5	24	18.5	52	30	29.5	12	18	23
10038	63	38	42	286.5	49	43	53	174.5	31
10039	56	27	25	150	29	37.5	16	38	21
10040	23	19	12	85	24	27.5	9	15	15
10041	98.5	74	42	113.5	38	55	31	57	64
10042	34.5	22.5	20	66	29	27	10	24	53
10043	49	42	26	82	39.5	39	18	47	44
10044	161	52	23	102	32	95	17	40	62.5
10045	58	71	42	135	73	52	26.5	64	69
10046	52	49.5	33	96.5	26	42	20	48	46
10047	76	55	30	113	30	45.5	24	50	52
10048	50.5	34	24	69	27.5	34	15	30	51.5
10049	47	51	35	95	34	43.5	25	51	57.5
10050	44	37	23	72	24	37	18	37	44
10051	47	65.5	51	91	34	44	26	56	69.5
10052	55	44	24	102.5	23	35	15	34	36
10053	53.5	64	31	130.5	32	49	32	56	70
10054	47	26	18	63	129.5	26	15	28	41
10055	41.5	17	21	77	24	26	10	33	31
10056	46	42.5	32.5	88	26.5	39	21	51	54

10057	46	29.5	22	75	19	29	14	36	53
10058	64	65	36	126	40	43.5	32	67.5	69
10059	47.5	37.5	22	86	21.5	36	16	36	45
10060	46.5	52.5	32	102	25	35	21	58	53.5
10061	56	59	33.5	112	31	46	27	59.5	70.5
10062	35	19	17.5	84	16	20	9	25.5	41
10063	74.5	44	20	104	22	62.5	15	43	70
10064	97	61	37	132	37.5	42	28	69.5	89
10065	35	31.5	22	63	23	26	15	30	42
10066	42	38	27	70	24	34	17	48	61
10067	55	80	37	104	33	52	31	77	79
10068	99	30	24	80	22.5	31	15	46.5	41
10069	44.5	78	32	105	28.5	48	28	68	69
10070	39	23.5	40	88	18	30	12	33	27
10071	40	37	20.5	90	26	33	18	35	46
10072	46.5	55	28	93	29	49	24	49	65.5
10073	68	38	25	125	27	48	16	48	78
10074	57	55.5	31	113	28	41	24	61	65
10075	36.5	39.5	26	92	25	35	16	49	42
10076	47	36	21	83	21	32	16	34	47
10077	59	36	24	97	30	51	15	54.5	51
10078	58	50.5	29	123.5	34.5	42.5	28	81	79.5
10079	59	37	22	99	28	33.5	39	39	41.5
10080	50	65	36	114.5	27	46	36	67	100
10081	54	58	30.5	118	33	43	25	63	71
10082	56	49	26	87.5	29	40	25	44	47
10083	32	26	19	112	17	26	16	30	28
10084	40.5	43	27	108.5	21.5	32	17	49	55
10085	27	17.5	10	38	21	23	10	16	26
10086	22	14	11	37	14	19	8	11	10
10087	21	14	9	63	11.5	20	6	12	11
10088	47	16	11	25	18	20	8	15	13
10089	79	14	8	25	14	34	8	13	17
10090	36	16	14	25	20.5	22	10	40	18
10091	29	16	13	130	710	20	8	21	13.5
10092	33	13	8	90	14	19	7	17	10
10093	36	17	28	109	23	20	9	24	23
10094	23.5	13	8	41	12	17.5	11	11	9
10095	74.5	15	11	91.5	20	29	20	57	13
10096	57	12	9	43	16	18	7	18.5	26
10097	20	10	8	42	19	18	5	9	10
10098	27	10	8	30.5	25	13	10	96	10
10099	53.5	55	27.5	116	35	42	25	61.5	55.5
10100	42	46	24	107	31	33	18	47	55.5
10101	38	45	25	79.5	32	30.5	20.5	46	52
10102	38	48	22	101	22	42	18.5	45	50
10103	73	70.5	33	98	30	50	30	78.5	82

10104	44	52	29.5	84	34.5	33	22	77	117
10105	43	60.5	26	76	25	34	25	57	55
10106	39	41	24	96	21	31	15	38	69
10107	31.5	23	16	70.5	16	21	11	27	63.5
10108	66	17	16	81	24	22	26	27	19
10109	112	44	26	92.5	22	35	18	48	58.5
10110	39.5	56	30.5	87	25	38	22	52.5	54
10111	26	18	12	62	24	22	9	23	31
10112	29	21	18	84	20.5	23	10	32	47
10113	40	34	28	92	20	29	16	44	59
10114	44.5	21	14	60	13.5	22	10	21	23
10115	48	27	22	71	36	29	20	45	32
10116	48	58	33	106	37	40.5	29	61	96
10117	28	24.5	18	87	21	22	11	55.5	36
10118	37.5	45	25	82	23	33	21	49	47
10119	33	45.5	26	74	23	32.5	20	53	80
10120	47	42	26	94	27	32.5	20	45	51
10121	35	49	22	78	24	30	17	43	40
10122	40	30	24	97	22	27	16	40	43
10123	35.5	32	20	75	15	23	15	41	44
10124	56	64	35	92	50	41	28	62	119
10125	41	61	30	122	28	36	44	60	66
10126	91	64	35	107	30	44	25	61.5	61
10127	119	50	32	79	24	43	19	44	51
10128	44	46	27	91	29	37.5	21	49	60.5
10129	44	44	26	78	26.5	31	17	44.5	44
10130	47	58	31.5	103.5	30.5	36	25	61	61
10131	66	46	26	96	32	45	20	46	67
10132	48	45	29	80	27	33	24	43	53
10133	64	43	28	86	27	48.5	21	53.5	52
10134	40	48	27	82	31	34.5	21	56	87.5
10135	73	72	38	123	34	46	34	74	82
10136	76	56	29	106	32	39	23	58	76.5
10137	61	73	39	118	65	48	30	77.5	117
10138	43	39	22	82	21.5	33	20	39	43
10139	35	34	20.5	90	35	31.5	16	40.5	38
10140	45	76	32	93	29.5	42	28	68	77
10141	57	71	35	109	42	44.5	25	73	79
10142	67	37	21	62	21	29	15.5	37	67.5
10143	72	45	27	93.5	29.5	32.5	17	45	38
10144	58	53	25	96	32	38	22	55	57
10145	45	42	24	79	22.5	30	22	46	49
10146	64	65	38	144	31	46	29	73	70
10147	40.5	53	30	83	35	37	23	55.5	61
10148	2910	102	97	112.5	277	742	22	45	117
10149	2079.5	102	111	153	331.5	468.5	25	60	102
10150	93	76.5	39	159.5	34	47	30	84	70

10151	15.5	9	4	12	10	13	7	21	7
10152	36	16	17	83	178	19	13	65	43
10153	37	20	22.5	55	903	21.5	13	61	13
10154	29	14	14	39.5	22	25	10	22	16
10155	27	19.5	12	40	19.5	27	8	19	20
10156	26	17	15	82.5	16	17	8	29	21
10157	109	34.5	32	213	39	32.5	17	52	83
10158	58	23	17	85	23.5	28	28	70	14
10159	77.5	25	31	154.5	54	38	29	36	28
10160	33	25	26.5	309	41	34.5	15	39	28
10161	33	23	19	71	24	28	21	50	18
10162	42	21	12.5	85.5	136.5	24.5	8	22	18
10163	32.5	20	14	54	23	24	15	21	55
10164	30	21	8	55	13	27	8	19	15
10165	50	30	29	312	63	39	13	83	25
10166	28	20	15	100	17	28	9.5	24	18.5
10167	22	17	11	48	15	21	8	13	11
10168	32	20	10	46	23.5	32	10	16	18
10169	45	16	15	139.5	15	21	8	28	24
10170	51	20	23	134	24.5	29	10	36	17
10171	28	17	12	72	15	23.5	12	44.5	15
10172	32	16	8	62	15	24	6	22.5	12
10173	21	17	8	53.5	14	24	6	13	11
10174	19	13	9	53.5	16	22	7	12	9.5
10175	20	15	12	52	13	21	6	12	12
10176	1214	29	34	117.5	47.5	38	30	52	32
10177	65	20	15	91	27	27	11.5	90	21
10178	43	18	13	81	20	32	10	23	31
10179	32	19	13	55.5	21	25	11	46	237
10180	41	18	10	56.5	47.5	26	8	18	14
10181	67	24	23	121	27	34	23	80	37
10182	33.5	19.5	12	56	17.5	29	12	51	16
10183	161	32	45	283	50	53	46	117	61
10184	64	19	16	117	29	27	14	26	15
10185	29	30	16	87	16	26	13	26	28
10186	46.5	20.5	18	122	25	26	37.5	35.5	34
10187	20	14	11	65	10.5	20	14	18.5	11
10188	25	19	10	67	100	21	9	18	23
10189	28	22	12	88	16	25	10	21	82
10190	25	17	17	89	22	20	14	21	23
10191	39	22	17	54	15	24	10	17	54
10192	40.5	22	44	116.5	43.5	24	16	37	31
10193	38	19	16	118	22	24	11	31	21
10194	44.5	17	13	57.5	23	21	15	23	15.5
10195	52	14	9	45	15	21	8	14.5	33
10196	30	17	10	48	17	24	8	51.5	12
10197	19	14	8	45	21	20	6	12	15

10198	19	13	8	59	17	17	7	14	11
10199	22	14	11	70.5	13	20	9	16	12
10200	21	14	10	67	12.5	17	7.5	16	8
10201	20	13	7	28	10	20	6	13	9
10202	20	13	8	55	10.5	20	7	15	9
10203	23.5	17	11	101	12	19	9	18.5	40
10204	22	15	9	101	28	22	8	17.5	11
10205	66	23	17	97.5	21	28	16	33	21
10206	53	24.5	21	106	25	33	18	86	34
10207	62	57	34.5	122	48	51.5	27	45	68
10208	21	22	9	47	15	23	7	98.5	26
10209	221.5	18	12	54	32	25	31	36	28
10210	344	13.5	14	97	21	20	14	18	18
10211	47	25	17	63	20	45	11	24	26
10212	30	14	9	39	19	21	8	14	11
10213	21	15	8	74	12.5	20	7	15	10
10214	21.5	12	7	49	16	18	7	14	9
10215	54	15	12	63	22	19	10	78	17
10216	21	11	8	57	13	19.5	6	14	9
10217	19	13	7	63	12	18	8	16	8
10218	31	16	12	71.5	23	22	9	117	12
10219	32	12	9	74.5	12.5	17	7	13	10
10220	29	16	10	72.5	21	21	19	45	11
10221	19	14	7	67.5	13	18	6	15	18
10222	47	14	9	46	24	20	13	108	9
10223	17	11	7	49.5	13	16.5	6	22	8
10224	21	12	8	30	14	17	6	13	8
10225	22	12	7	54	14	17	8	46	13
10226	126	46	26.5	114.5	21	37	29	114.5	39
10227	20	13	6	43	13	18	6	17	8
10228	26	16	16	117.5	15	20	9	39	11
10229	43	86	42	119	46	55	34	75	79
10230	58	63	35	95.5	39	60.5	26	53	60
10231	52.5	75.5	40	114	45.5	51	31	60	70
10232	59	43	29	95	33	44	20	44	62
10233	35	43	25	80	33	36	20.5	40	46.5
10234	41	53.5	27	75.5	35	39	20.5	50	59
10235	57	78	44	110	158	52	30	67	93
10236	55	38	23	73	32	53.5	17	32	52
10237	28	33	22	81	33.5	33.5	16	30	40
10238	41	33	26	85	42	35	17	40	37
10239	48	47	32	102	44	40	20	97	55
10240	39	49	31	99	39.5	34	20.5	47	60.5
10241	41	51	31	98	50.5	43.5	29	48	56
10242	46	55	30	119	35	42.5	22	50	57
10243	46	73	39	113.5	45	49	30	64	82
10244	48	57.5	39	108	42	49	33	59	74

10245	64.5	57	36	128	41	66	28	72	69.5
10246	36	57.5	32	86	44	39	22	51	85
10247	39	50	27	89	32.5	36	22	47	78
10248	39	38	24	106	30	33.5	16	41	76
10249	39	50	27.5	97	43.5	38	19.5	45	53
10250	45	41	30	67	33	44	18	33	54
10251	33.5	41	26	83	43.5	33	20	37	53
10252	50	64	63	246	47	51	25	67	72
10253	30	38	23	77	38	28	18	35	50
10254	33	46	34	90	34	36	21	44	51
10255	38.5	81	44	120.5	38.5	44	32.5	71	73
10256	60	54	38	112	42	55	22	61	62
10257	32	31	23	103	38.5	27.5	17	36	36
10258	35.5	37	27	125.5	37.5	32	21	43	50
10259	41	52.5	35	111.5	40	41	23	50	52
10260	45	43	38	112	47	37	21	53	61
10261	53	59.5	34	131	39	48	28	54	59.5
10262	45	70	37	97	44	45	29.5	72.5	70
10263	34.5	37	29	78	32	25	19	44	51
10264	39	53.5	31	138.5	37.5	39	22	50	51
10265	39	38	31	97.5	41	34.5	21	41	52.5
10266	38	48	32	97	37	37	22	48	58
10267	33	36	23	78	30	31	18	34	41
10268	52	59	31	72	35	49	26	51	69
10269	30	38	27	93.5	38	27	38	42	61.5
10270	48.5	65	37	110	36.5	44	31	53	62
10271	26	34.5	24	99	30	26	17	38.5	55
10272	30	44	27	117	32	30	21	40	46
10273	60.5	56.5	36	109.5	43.5	44	28	55	67.5
10274	21	14	12	67.5	23	19	7	15	26
10275	21	14	15	77	32	18	46	20	14
10276	16	14	10	40	20	16	8	12	21
10277	22	11	9	52	21	17	8	11	8
10278	17	13	12	46	32.5	19	8	12	61
10279	23.5	20	16	48.5	24	22	10	15.5	34
10280	35.5	18.5	19	61	108	24	12	29	74
10281	31	18	14	40	37	20	11	16	30
10282	22	17	14	57.5	35	20	15	20	23
10283	63.5	31	19	101	37	63	19	59	128
10284	18	11	9	33	33	15	12	15	11
10285	17	14	12	75	44	17	10	15	29
10286	54	21	32	120	36	21	22	53	163
10287	62.5	15	18	93	41	17	13	29.5	94.5
10288	25.5	14	21	234	28	21	24.5	54.5	16
10289	58	24	32	158	95.5	30.5	20	87.5	36
10290	26	13	23	99	28	18	10	23.5	16
10291	30	16	42	165	30.5	22	16	50	17

10292	30	17	45.5	107	29	19	15	28	23
10293	23	15	13	45	30.5	17.5	17	17	19
10294	39	23	23	126.5	48.5	26	18	56	40
10295	51.5	21	29	109	51	27	15	46.5	102
10296	35	17.5	25	92	187	23.5	48	36	48
10297	68	33	33	78	46	34	34	52	45
10298	26	12	8	49	48.5	16	7	12	9
10299	19	12	7.5	24	27	17	8	11	12
10300	16	12	7	58	22	16	6	11	9
10301	13	12	7	21	20	16	8	8.5	8
10302	73	11	9	35	27	19	24	9.5	7
10303	16	11	7	35	25	19	7	11	13
10304	15	8	10	71	60	11	11	18	25
10305	41	11	12	52.5	29	17	13	17	12
10306	49	20	18	71	36	33.5	13	27.5	31
10307	45	17	17	74	143	23	14	27	34
10308	27	16	36	92	167	21	30.5	18	28
10309	18	12	10	15	29.5	15	8	13	8
10310	16	11.5	10	29	28	15	8	10	10
10311	38	12	11	55	29	15	15	20	16
10312	34	18.5	14	49	36	20	26	43	13
10313	32	15	16	51	47	21	58	79.5	47
10314	39.5	19	17	52	38.5	23	14	22	30
10315	22	14	11	37	32	18	9	72	14
10316	50.5	14	10	32	43	19	45	21	12
10317	17	11.5	10	38	27	15	19	12	21
10318	92	20	16	45	111.5	37	11	23	24
10319	52	14	10	32	30.5	27	10	31	13
10320	52	19	27	93	246.5	24	16	80	143
10321	41	14	20	92	33	15	13	28	58
10322	66	18	20.5	68	36	22	17	29	20
10323	87	17	15	45	109.5	34	11.5	27	26
10324	55.5	15	12	35	33	25	10	35	12
10325	42	15	22	82.5	220	19	15	70.5	122
10326	35.5	14	22	87	31.5	18	12	27	53
10327	29	22	17	49	30	30	16	24	16
10328	39	15	13	43.5	41.5	31	13	24	85
10329	32	30	18	72	27	22.5	15	35	39
10330	55.5	26	18	80	25	36	13	30.5	51
10331	49	38	25	89	47.5	28	21	43	66
10332	26.5	28	20	71	29	24.5	14	32	34
10333	37	41.5	29	87	34	29	19	42.5	43
10334	33	43	24.5	89	32	27	22	45	44
10335	36	42.5	38	87	33	32	20	50	55
10336	39	38	24	77.5	30	37	18	37	63
10337	26	30	19	92	26.5	23	14	35	50
10338	41	51	26	82.5	29.5	28	18	39	35

10339	37	52	33	113.5	41	34	29	54	66
10340	29.5	41	20	79	29.5	27	16	40	66
10341	21	24	15.5	58	23.5	23	12	25	24
10342	39.5	33	21	80.5	27	33	17	38.5	42
10343	37.5	33	19	87	28	26	15	41	35
10344	29	26	17	59	26	26.5	15	29	27
10345	25	29	17	54.5	26	24	13	29	43
10346	37.5	34	22	73	28	32	16	34.5	36
10347	43	44	37	92	34	32	22	47	67
10348	45	28.5	19	85	23	38	13	29	29
10349	34	39	28	139	36	26	22	52	42
10350	27	29	21	74	37	24	18	34	43
10351	30	40	22.5	88	33	26	18	40	37
10352	46	33	24	74	41.5	44.5	15	42	34
10353	32	30	31.5	53	22.5	28	14	28	47
10354	79	41	19	65	27	69	16	31.5	46
10355	33	34	20	77	25.5	29	15	32	54
10356	40	37	26	89	33	30	18.5	44	55
10357	27	30	18	65	26.5	27	16	37	48.5
10358	36	51	34	97	35.5	33	25	54	86
10359	32	43	24	72	30	28	22	41.5	56
10360	25	29	20	61.5	30	19	15	30.5	31.5
10361	28	27	17	67	24	24	13	38	31
10362	55	72	35	106	40	40.5	27	71.5	75
10363	23	12	11	76	19	19	7	21	16
10364	32	40	25	76	29	30	19	44	51
10365	27	27	19	71.5	25	21	15.5	31.5	49.5
10366	32	36	24	90	35	24	20	47	61
10367	49	30	18.5	93	22	39	12	32	29.5
10368	32	42	25	99	33.5	27	20	43	55
10369	29.5	45	21	79	26	29.5	19	41	43
10370	61.5	22	21	70	23	33	12	30	28
10371	44	41.5	23	85	30	39	18	48	50
10372	30	36	23	70	36	25.5	17	38	49
10373	32	35	20	71.5	28.5	25	18	40	45
10374	35.5	57	32	80.5	34	38.5	26	54	75
10375	29	35	21	80	31	26	17	37	55
10376	37	51.5	29	98	33	38	23	48.5	53.5
10377	59	52	30.5	96	43	34	34	65	59
10378	27.5	30.5	18	68	24	24.5	14	31	35
10379	44	47.5	30	100	36	35	24	55	60
10380	39	40	21	75	27	29	17	39	44.5
10381	32	23	16	73	31	18	13	26	27
10382	33	34.5	18	76.5	25	28	15	31	42
10383	20.5	13	12	45	19.5	14	9.5	21	19
10384	76	52	27	73.5	34	42.5	23	56	60
10385	31	33.5	19	140	27	26	17.5	39	41

10386	40	37	21	79	31	28.5	16	42	41.5
10387	34	53.5	31	89	80.5	37	22	47	55
10388	17.5	9	7	20	25	11	7	11.5	11
10389	14	10	7	25	16	11	7	34	5
10390	17	11	12	54	29	16	11.5	19.5	10
10391	16.5	12	9	52	28	17	8	16	12
10392	17	8	7	35	22	11	6	10	5
10393	18	12	8	41.5	19	15	10	15	6
10394	26	18.5	11	51	17.5	20	11	75	13
10395	75	25	14	45.5	16	61	12	17	16
10396	18	19	12	34.5	24	24	9	14	14
10397	30	19	18	90	22	29	11	32	15
10398	30	20	16	114	19	25.5	10	22	22
10399	19	14.5	10	51	14	20	8	13	11
10400	28.5	17	13	71.5	14	23	7	12	14
10401	19	16	8	28	17	19	6	16	10
10402	40	22	18.5	35	21	32	11	22	23
10403	68.5	23.5	14	39	21	58	99	19	36
10404	30	14	17	65	34.5	21	12	22	23
10405	72	64	39.5	111	61.5	68	25	62	58
10406	36	49	28	95.5	31.5	36	23	49	70.5
10407	35	40	24	139.5	27	32	17	38	41
10408	36	40	35	110.5	34.5	29	18	43	43
10409	218	47	28.5	103	26	63	20	43	55
10410	44.5	41	25	105	26	38	18	37	89
10411	69	37	27	144	30	34	17	42	40
10412	50	39	23	98.5	24	28	20	37	46
10413	78.5	54	33.5	95	29	64	22	48	46
10414	54	61	40	98	44.5	42	31.5	62	62
10415	44	42	34	101	39	36.5	21	51	46
10416	36	30	20	98	220	30	17	31	36
10417	36	39	27	81	30	31	21	42	49
10418	50	41	42	83	38	34	23	42	44
10419	481	52	29	102	143.5	36	23	51	62
10420	48	61	31	122.5	27.5	37	26	61	57
10421	52.5	30	21	90	22	27	17	34	36
10422	39.5	32	22	94	20	31	17	36	39
10423	52	35	23	101.5	24.5	34	43	39.5	61
10424	31	27	63	73	32.5	25.5	16	29	59
10425	75.5	16	19	54	34.5	21	16	26	12
10426	9	12	6	11	6.5	13	7	8	7
10427	17	14.5	9	41	14	16	7	19	10
10428	68	18	17	116	52.5	26	13	21	26.5
10429	32	36	18	88	32	20	10	23	54
10430	30	16	18	85	19	21	11	21	25
10431	34.5	16	17	81.5	18	19	11	22	21
10432	24	14	16	97	17	19	9	21	34

10433	30	14	15	83	14	17	11	19	21
10434	41	13	23	128	18	22	11	29.5	19
10435	30	16	20.5	91.5	35.5	18	9	30	17
10436	26	11	16	121	196.5	13	8	31	15
10437	50	17	17	95.5	22	24.5	10	27	18.5
10438	47.5	61	40	126.5	29	42	31	72	70
10439	33	20	22	85	27.5	19	11	32	26
10440	37	14.5	19	97.5	22	23.5	11	29	33
10441	30	15.5	17	107	18.5	21	10.5	22	13
10442	33	12	20	79	17	20	8	23	24
10443	18	12	14	68	15	14	7	15	17
10444	30	13	17	98	20	18	7	24	23.5
10445	36	41	23	108	167	17	9	26	26
10446	32.5	17	19	89.5	24.5	24	11.5	33	35
10447	32	18	13	60	140	28	12	17	22
10448	37	16	20.5	123	30	18	13	53	39
10449	23	14	9	29	188	19	8	15	13
10450	36.5	23	12	56.5	32.5	39	10	34	24
10451	539	14	11	44	23	21	11	45	14
10452	12.5	9	7	27.5	23	11	6	8	7
10453	20	14	26	102	29	18	8	21.5	18
10454	30.5	25	24.5	69	32.5	26	15	23	37
10455	39	45	40.5	115.5	38.5	41	21	55	51
10456	37	51	32	87	38	39.5	25	48	90
10457	35.5	35	32	145	29.5	37	20	47	47
10458	57	49	33	147	33	46	23	44	77
10459	144	44	30	79	29.5	37	20.5	63	56
10460	60.5	53	35	119	38	45	25	59.5	107
10461	33.5	40	26	83	27	42	19	40	43
10462	35.5	55	33	94	33	37	24	47	70
10463	30	21	20	98	31	32	10	21	32
10464	47	50	34	136	37	40	25	51	58.5
10465	38	31	23	94	26	28	14	33	39
10466	46	45	26	131	33	44	20	42	112
10467	28	28	18	86.5	25	29.5	13	24	37
10468	45	61	34	113.5	48	52.5	26	54	63.5
10469	62	43	34	135	55	34	26	57	143
10470	39	43	31	104	33	43	20	35	43
10471	33	42.5	26.5	98.5	30	31	19	41.5	39
10472	83	53	31	106	34	84	21	42	56
10473	45	51.5	32	104.5	31	35.5	24	52	69
10474	46.5	47	33	104	34	35	23	46	61
10475	67	55	34	99	41	67.5	25	56	60
10476	127.5	45	26	101	30	63	19	43	47
10477	58	46	32	98.5	25	38.5	23	44	56
10478	43	45	34	110	33	38	25	46	74
10479	42	53	31	99	37.5	41	26	49	54

10480	34	39	28	98	33	32	27	41	49
10481	278.5	54	35.5	119	36	53	27	54	76
10482	76	66	48	136	42	55	31	67	71
10483	43.5	78	43	110	42	50	32	75.5	93
10484	45.5	35	30	85.5	31	51	17	41	57
10485	35	42.5	31	90	30	36	23.5	43.5	52
10486	33.5	42	28	77	31.5	33.5	21	38	74
10487	58.5	50.5	32	118	32	36	26	53	59
10488	36	55.5	30	133	32	36	23	49	62
10489	32	48	30	87	32	33.5	22	45	54
10490	44	42	29	85	31	38	16	41	40
10491	35	45	33	103	33	37	21	44	48
10492	56	50	30	123.5	42	40	23	49	57
10493	106	47	37	110	36	42.5	28	48	47
10494	36.5	47	29	90	32.5	36	22	44	52
10495	47	50	29	128	72	36	64	47	57
10496	46	64	32.5	116	33	46	30	64	69
10497	16	13	14	68.5	19	18	9	13	12
10498	49	27	28	206.5	42	32	74	55	30
10499	28	17	11	53	19	19	7	17.5	13
10500	53	23	40	134	31	29	19	49.5	24
10501	29	17	15.5	94	24	21.5	9	39.5	12
10502	28	23	22	108	24	20	11	25	25.5
10503	38	23	44.5	105	36	27	15.5	31	39
10504	22	17	18.5	48	26	20	9	37	39
10505	52	22	22	74	28	27	13	40	18
10506	143.5	77	98	406	70	64	89	287	90
10507	28	15	11	43	26	21	8	16.5	13
10508	51	32	26	75.5	28	33	16	29	35
10509	42.5	22	30	66	31	28	30	85	21
10510	19	18	14	46	88	19	14.5	16	15
10511	27.5	17	15	98.5	26.5	21	11	42	14
10512	16.5	13.5	7	22	19.5	18	243	10	8
10513	17	14	9.5	41.5	20	19.5	9	14	14
10514	21	14	12	51	23	19	9.5	13	15
10515	14	12	8	44	23	18	7	18	13
10516	15	14	10	73	24	14.5	9	13	9
10517	17	12	9	46	24	16	9	11	11
10518	30	14	8	43.5	31	17	7.5	48	10
10519	28	18	21	55	27	21	9	23	19
10520	14.5	13	8	17	23	16	8.5	11.5	9
10521	49	21	19	114	40	28.5	15	43	22
10522	33	20	14	38	36	22	12	18	15
10523	20.5	14	14	80	26.5	17	11	18.5	16
10524	22	13	12	39	34	17	10	14	17
10525	18	9	12	76.5	21	13	12	14	8
10526	27	11	15	79.5	42	14.5	8	20	12

10527	20	14	11	70	25.5	19	21	17	11
10528	15	11	12	38	21	13	15	12	8
10529	19	12	10	41	30	15	13	17	11
10530	30	13	23.5	75	27	18	10	28	31
10531	19.5	20	12	58	27	24	9	17	11
10532	23	14	11	72	21	13	9	14	12
10533	18.5	12	8.5	54	22	15	10	22	14
10534	21	15	14	41.5	51.5	16	11	12	10
10535	92.5	19	21	99.5	33	31.5	23	212	23
10536	23	16	16.5	94	36	18.5	12	17	29
10537	115.5	27	32	118	47	31	26	149	76.5
10538	28.5	13	8	25	21	16	8	13	11
10539	41	16	12	51	25	21	12	15	13.5
10540	22	12	12	38	28	16	9	18	25
10541	18	17	13	58.5	19	15.5	9	16	68
10542	20	15	16	64	19	19	10	18	39
10543	17	14	15	58	20.5	16	8	16	25
10544	26.5	23	17	62	24	21	15	25	24
10545	26	23	16	80	18	22	11	28	56
10546	22	18	13	50	20	16	9.5	20	56
10547	32	15	14	70.5	25	20	10	19	16
10548	25	14	11	44	30.5	16	9	16	17
10549	47	14	12	50	24	29	9	43	24
10550	39	18	19	70	37.5	21	18	79	17.5
10551	86.5	15	15	61	44	21	19	47.5	18
10552	43	15	12	41	20	21	13	15	19
10553	28	17	13	44	76	21	13	23	37
10554	33	21	19	96	28	22	16	37	106
10555	28.5	15	16	59.5	29	21	11	71	15
10556	20	13	18	57	64	17	12	57	26
10557	29	17	46	90	36	21	12	42	164.5
10558	29	14	19	84.5	82.5	19	16.5	154	12
10559	38	19	24	133	22.5	30	16	35.5	19
10560	33	31	24	66.5	29	25	14	31	38
10561	29	33.5	24	82	29.5	29	19	39	39
10562	34	43.5	25	102	29	27	20	44	54.5
10563	26	21.5	20	69.5	28	22	13	33	23
10564	43	45	24	105.5	28.5	33	19	41	42
10565	32.5	46	63.5	82.5	27	32.5	20.5	45	44
10566	52	52.5	25	107	30	48	18	37	53
10567	38.5	45	28	114	31	34	21	43	53
10568	43.5	79	40	112	45	47.5	38	78	72
10569	34.5	42	26	96	28	26	22	43	52.5
10570	34	34	23	76.5	27	30.5	15	37	38
10571	49.5	39.5	26	121	166	38	22	55	41
10572	42	63	31	117	36	39	27	61	85
10573	54	52	29	94	29	61	22	46	53.5

10574	31.5	38	23	83	26.5	30.5	18	40	48
10575	51.5	56.5	30	102	32	37	23	54	69
10576	36	52	31	98	33	37	27	53.5	55
10577	53	52	28	88	40.5	48.5	20.5	55	55.5
10578	27	34	19	76.5	23.5	28	13	32	47
10579	32.5	36	24	93	31	26	27	41	42.5
10580	28	33	23.5	91	27.5	29	17.5	36	43
10581	41	53	29	93	34	42	25	49.5	60
10582	35.5	38	24	93.5	29	29	20	42	61
10583	25	21.5	16	63	21	18	11	31	23
10584	42	53	30	105	35	35	24	71.5	76
10585	30	42	24	96.5	27	26.5	19	39	71
10586	35	44	27	101	30	31	23	43	42
10587	36	48	26	82	31	35	22	47	51
10588	37.5	47	25	92	35	33.5	21	40	53
10589	31.5	39	24	93.5	23	30	17	35.5	42
10590	28	34	21	86	25	22	16	37	36
10591	37	46.5	24	70.5	22	40	21	57	48
10592	42	50	29	102	32	35	31	54	48.5
10593	41	68.5	37	100	37	48	29	65	80
10594	71.5	54	32.5	88.5	34	41	29.5	56	55.5
10595	106	63	35	148	33	56.5	28	61	68.5
10596	59	58.5	30	103.5	28	66	22	48.5	60
10597	12	12	6	10	8	14	6	11	6
10598	24	26	18	84	24	20	14	26	28
10599	24	23	18	59.5	21	20	14	29	29
10600	27	40	30	95.5	134.5	29	18	43	46
10601	25.5	27	17	87	25	23.5	13	27	35
10602	38.5	31	19	87	21.5	23	14	34	33
10603	23.5	25	18	70	23	21	15	28	68.5
10604	39	31	20	90.5	24.5	31.5	15	29	36
10605	24	21	16	63.5	21	19	12	26	24.5
10606	35	55	28	96	29	35	25	53	74
10607	32.5	28	22	90.5	24	22	14	33	24
10608	36.5	48	31.5	118	43	30	24	65	59
10609	25	19	21	86	25	24	14	26	27
10610	31	42.5	27	79	31	29	18	48	47
10611	40	37	23	118	27.5	31	18	41.5	48
10612	57	41	26	101	27	40	18	38	72.5
10613	35.5	31	21	96	27	28.5	15	33	41
10614	33	21	19	79	23	27	10	16	23
10615	32	24	26	78.5	33	30	54	39	31
10616	173	27	29	59	49	38	30	36	35
10617	45	22	24	33	29.5	24.5	15	17	33
10618	27	22	20	24	34.5	29	25	14	22
10619	18.5	19.5	15	27	22	21	14	14	16
10620	98	23	16	57	102	26.5	13	67	18

10621	27	22	24	62	26	32.5	12	62	15
10622	23	19	21	72	31	24	9	18	19
10623	11	17	8	14	12	21	7	8	10
10624	19	17.5	14	51	31	21	10	15	13
10625	19	16	16	114.5	19.5	23	8	19	11
10626	59	18	14	40	28.5	23	10	14	13
10627	23	30	21.5	70.5	102.5	25	11	18	15
10628	29	17.5	17	65	31	26	11	28	17
10629	24	21	14	47	21	27	11	32.5	17
10630	16	17	13	53	33	20	9	13	13
10631	21	24	15	39	25	23	11	24	21
10632	18	17	12	44.5	18.5	20	9	17.5	11.5
10633	29	39	23.5	65	28	32	15	44	36
10634	94	56	28	74.5	30	104.5	18	38	68
10635	34.5	43	27	87	32.5	38	20	42.5	38
10636	36.5	40	22	72.5	29	39.5	14	35	35.5
10637	41.5	43	29	90	33	34	24	53	48
10638	42	79	45	113	47	43	33	73	66
10639	25.5	33	26	82	27.5	30	17.5	37	63
10640	36	35	26	88	28	30	18	42	32
10641	50.5	77	42	105	69.5	55	33	75.5	84
10642	36	47.5	29	79	31	35	19	38	40.5
10643	66	47	30	95	34	41.5	28	46	55
10644	55.5	40.5	28	94.5	31	35	19	43	36.5
10645	31.5	50.5	34	103.5	34	36	23.5	46.5	47
10646	43	42	37	116	48	35	27	46	46
10647	55	53	36	99.5	41	43	24	48	46
10648	37	46	30	82	32.5	35	20	43.5	56
10649	36	64.5	38	98	39.5	45	32	63	75
10650	41	39	33.5	87.5	29.5	34	19	40	37
10651	40	46	29	77.5	34	37	18.5	40.5	44
10652	37	21	21	78	22	26	9.5	19	23
10653	34	17	28	89.5	27	19	13	23	12
10654	10	14	8	11	18	16	7	9.5	8
10655	18	17	13	67.5	23.5	18	9	49	17
10656	27	18	18	69	27.5	23	24	40	12
10657	32	19	16	73.5	47.5	34	12	17	20.5
10658	36	21	27	124.5	26	33	12	30.5	16
10659	43	31	21	88	27	29	16	33	59
10660	30	20	21.5	83	23	22	13	26	86.5
10661	31	18	30	76	45.5	20	13	44	89
10662	55	25	39	129	28	28	19	75.5	134
10663	23	16	18	51	55	20.5	11	17	117
10664	939	164	31	106	66	833	26	93	288.5
10665	32	18	18	53	31	23.5	23	23	17
10666	41.5	18	17	50	65	22	15	21	21
10667	20	18	19	67	19.5	16	8	17	56

10668	28	18	15	48	25	19	11	16	26.5
10669	22	23	16	46	30	20	11	17	37
10670	20	20	19	51	22	18	10	19	32
10671	28	16.5	14.5	58.5	24	19.5	12	20	38
10672	32.5	17	25	72.5	22	21	12	21	40
10673	26	19	16	51.5	22	21.5	9	17	23.5
10674	18	18	14	64	17.5	22	10	18	35
10675	19	16	13	28	17	18	10	25	29
10676	14	11	15	27	19	16	8	14	12
10677	24.5	14	13	25	29	18	10.5	17	12
10678	21	14	16	64	20	17	10	19	14
10679	36	22	18.5	43	87	35	18.5	32	19
10680	14	13	9	25	21	16	7	20	9
10681	15	14	9	49	15	16	6	13	9
10682	52	17	15	51.5	121	21	16	23	31
10683	12.5	13	9	36.5	19.5	14	6	11	28
10684	13.5	13	10	29	21	15.5	8	17	9
10685	62	72	43.5	120	43	60	31	61.5	109
10686	48.5	61	38	112	39	44.5	25	54.5	74
10687	40.5	50	33	122	41	44.5	30	48	87
10688	64	81	37	164.5	62.5	65	34	152.5	83.5
10689	57.5	79	41	126	45.5	60.5	28	67	78
10690	63	92	59	147	84.5	62	35	80	97.5
10691	39	43	26	101	61.5	37	20	35	41
10692	49	56	34.5	88	34	46.5	23	44	57
10693	52.5	54	37	101	33	45	26	52	84.5
10694	45	57	32	129	36	44.5	23	58	72
10695	49	57	33	131	37.5	48	29	49	75
10696	43	25	18.5	161	42	35	12	26	42
10697	54	69	41	124	48	53	33	62	68
10698	44	50	32	117.5	31	46	21	63.5	48
10699	43.5	55	39	139	38	49	31	56	86
10700	47	63	37	114	40	45	28	54	77
10701	41	53	34	134	36	44	29.5	55	82
10702	144	44	30	99	28.5	35	18	42	48
10703	40	45	29	106	30	37.5	21	38	40
10704	80	61.5	29	116	31	61	25	50.5	54
10705	45.5	44	28	102.5	30	43	21	47	57
10706	58.5	66	37	121	39.5	57	30	74	88
10707	40.5	50.5	34	109	33.5	43	26	76	46
10708	61	49.5	31	135	24.5	51	17	41.5	45
10709	37	50	35	113	34	43	23	50	48
10710	59	70	41	106	44.5	48.5	31.5	100	73
10711	43	36	26	125	27	40	16	33	34
10712	46	53	29	108	27	45	19	48.5	58
10713	28	20	16.5	84.5	24.5	23	10	20	46
10714	50	66	42	177	41	38	31	71	77

10715	73	63	46	135.5	42	55	25.5	53.5	89.5
10716	43	35	21.5	141.5	61.5	41	21	35.5	42.5
10717	32.5	35	20	98	25	32	17	32	35
10718	51	51	35.5	134.5	35	39	22	56	87
10719	56	53.5	29	123.5	33	41	24	60	71
10720	32.5	34	23	114	41	38	18	36.5	46
10721	48	57	30	118	38	57	22	47	62
10722	41.5	52	30	126	32.5	36	21	49	62
10723	16	16.5	9	52	17	22	7	19	12
10724	32	19	20	135	28	30	26	23	44
10725	20	18	10.5	62.5	27	24	8	15	14
10726	24	22	15	70	21	25	9	16	21
10727	30	18	19	130	37	26	19	27	18.5
10728	17	14	10	50	17	21	7	12.5	10
10729	47	21	16	42	40	27	20	67	19
10730	43	25	22	132	50.5	32	22	113.5	39
10731	439.5	20	16	48.5	27	30	25	67	21
10732	36	20	18	42	41	29	15	22	43
10733	35	18	15	89	71	26.5	12	48	16
10734	48	20	18	71	50	34	32	22.5	26
10735	38	22	20	51	43.5	25	39	27	47
10736	30.5	22	18	83	23	32	11	21	42.5
10737	22	12	12	63	23	18	9	24	17
10738	34	16.5	12	48	31.5	24	10	16	13
10739	25	16	12	88	18.5	23	14	20	12
10740	44	19	13	46	883	27	24.5	15	15
10741	59	15	12	62	20	22	11	19	14
10742	40.5	22	18	83	26.5	27	10	26	60
10743	26	17.5	14	58.5	18	22	9	16	19
10744	41	50.5	26	68	39	34	16	38	36
10745	29	26	22	209.5	18	31.5	11	77	33.5
10746	30	16	16	79	18	22	11	18	19
10747	20	13.5	16	55	15.5	24	5	11	9
10748	16	15	10	62	22	17	8	16	9
10749	24	15	10	90.5	15	18	8	17	33
10750	15	13.5	8	30	16	17	7	11	9
10751	13	13	9	37.5	10	19.5	7	10	9
10752	20.5	15	9	73	12	19	7	17	8
10753	19	14	8	61	11	21	7	12	9
10754	16	15	13	72	15.5	20	9	13	10
10755	16	15	9	71	14	19	7	13	9
10756	10	16	9	116	3	9	5	7	9
10757	22	16.5	13	100.5	24.5	21	10	17.5	18
10758	16.5	14	11.5	77	17	17	7	12	11
10759	25	24	14	109.5	17	20	10	16	15
10760	16	15	13	65	15	18	7	16	10
10761	22	58	12	83	18	20.5	8	27	12

10762	18	12	9	56	12.5	14	7	14	8
10763	24	21.5	18	69.5	18	29.5	9	15	14
10764	12	11	5	19	10	14.5	7	9	7
10765	27	15	25	172	38	20	16	23.5	16
10766	20	14	12	93	16.5	16.5	7	16	24
10767	18	13	10	52.5	13	17	7	12	11
10768	43	17	19	148	21	23	21.5	32	12
10769	28.5	15	12	92	28	17	12	18.5	10
10770	22	17	16	74	21	22	11	14	109
10771	18	13	9	28	22	19	9	12	21
10772	37	20.5	17	78	28	23.5	235	26	32
10773	47	15	12	57	23	21	9	15	22
10774	43	17	15	48.5	30	25.5	19	61	22
10775	93	16	15.5	91	35	25	23	60	23
10776	18	11.5	10	89.5	17.5	14	8	15	10
10777	24	18	19	47	33	19	11	20	43
10778	40	16	44	112	27.5	22	13	29	96
10779	27	13	22	50	26.5	21	14	111	13
10780	15	14	10	48	41.5	17	9	20	9
10781	38	60.5	27.5	117	29	34.5	22	56	67
10782	49	37.5	23	152	23.5	40.5	18	42	40
10783	52	40	29	94	26	48	18	39	129.5
10784	34	42	27	90	25.5	28	19	42	41
10785	49.5	38	19	83	22	50	17	35	44
10786	33	30	22	94	24	25	14	33	80
10787	29	32	30	88.5	24	27.5	16.5	34	33
10788	34	31	23	104	28	28	17	43.5	32
10789	53.5	49	28.5	87	32	49	25	52	53
10790	30	38	21	96.5	22.5	29	19	38	38
10791	26	17	13	50	36.5	20.5	8	19	15
10792	39	43	23	106	26	34	17	41	66
10793	22	20	14	67	19	20	11	22	23
10794	37	45	27	101.5	36	31.5	19	50	43
10795	34.5	32	22	88.5	25.5	24	16	50.5	37.5
10796	30.5	24	20.5	82	22.5	24	13	57	37
10797	41	42	24.5	126	53	40	20	41	78
10798	38.5	40.5	27	125	30	28	21	49	49
10799	33.5	44	31	126.5	28	32	21	48	64.5
10800	36	51	30	113	24	29	17	41	42
10801	42.5	41	26	98	27	33.5	20	46	42
10802	77	23	17	82	20	26	14	26	22
10803	70	57	37	113	44.5	42	36	84.5	87
10804	26.5	28.5	21	74	23	26	14	35	33
10805	33	26	23	89	23	25	16	33	32
10806	38	43	31	107	33	36	21	44	61.5
10807	30	32	20	81	22	24	14	32	36
10808	29	30	21.5	90	27	25	14.5	32	32

10809	30.5	18	16	87	18	24	10	27	23
10810	41	38	23	89	24	29	18	42	38
10811	52	33	19	109	22	52	15	34.5	37
10812	42	46	26	120	31	36	22	51	51
10813	54.5	37	20	92	24	49	13	32	44
10814	30	30	22	94	23.5	26	16	33	41
10815	38	39	29	83	32	30.5	22	45	39
10816	38	34	23	80	27	26	18	42	46
10817	31.5	35	26	95	28	24	17	39	34
10818	29	31	25	93	28	27.5	14	43	34
10819	34.5	40	26	122	30	33	19	43	50
10820	27	33	22	96.5	22	27.5	34	33	47
10821	29	40	26.5	80	65.5	32	19	37	53
10822	42	35	20	80	23	45	13.5	31	68
10823	30	32	20	81.5	20	25	15	35	35
10824	33	40	30	83	28	30	18	39.5	51.5
10825	33	38	26	101	24	32	17	35	45.5
10826	31	45	25.5	107	26.5	32	23	49	55
10827	26	23	24	66.5	20.5	25	11	27	28.5
10828	57.5	58	39.5	138	42	43	30	66	60
10829	36	44	22	106.5	32	35.5	17	40	49
10830	28	32.5	22	89.5	35	26.5	16	38	30
10831	54	41	36	110	37	34	23	79.5	50
10832	50	64	43	147	40	46	31	67.5	79
10833	44	31.5	24	118	22.5	25	19	36	34
10834	45	47.5	29	118	26	34	22	53	44
10835	95	58.5	39	117	30	40	26	75	76
10836	19	11	10	44	24	15	7	13	10
10837	32.5	13	14	70	20.5	16	25	26	32
10838	25	15.5	10	57	20.5	17	10	19	11
10839	23	13	9	65	25	21	8	16	10
10840	37	15	43	114.5	30	21	11	48	260
10841	46	17	15	83	26	31	10	46	14
10842	30	12	10	48	15	17	8	17	10
10843	52	16	19	132	30.5	21.5	13	43	19
10844	30	12	18	148.5	19.5	18	13	26	17
10845	19	12	13	72	17.5	18	9	30	32.5
10846	31.5	12	11	33	14.5	22	8	16	8
10847	17	18	15	53	21	23	9	14	24
10848	17	16	12	67	28	20	7	14	12
10849	19	18	14	73	25	20	9	16	14
10850	34	17	12.5	63	32	23	11	126	13
10851	20	17	13	59	25	22	10	19	21
10852	17	19	9	46.5	25	23	11	19	10
10853	25	18	11	74.5	30	26	9	20	16
10854	60	31	24	66	37	47.5	16	65	27.5
10855	30	18	18	56	34	21	13	19	17

10856	39.5	22	20	68	30	23	12	21	52
10857	30	19	33	92.5	37	23	32	31.5	25
10858	38.5	17	16	26	27	26	12	20	19
10859	24.5	19	14	34	27.5	25	10	17	18.5
10860	44	24	40	154	37	30	21	88.5	26
10861	73	42	35	109	42	35	30	52	58
10862	42.5	22	23	130	48	27	18	38	118
10863	54	20	32	155	54	28	22	28.5	36
10864	40.5	26	31	96	50.5	28	20	42	31
10865	41	19	18	147	40	26	18	32	29
10866	32	21	10	69.5	24.5	37	8	14	11
10867	76	71	50	120	46.5	58.5	31	63	89.5
10868	84	69	38	101	40.5	63	44	61	91
10869	46	49	36	117	40	45	23	44	66
10870	46.5	30	24	75	39	35	18	29	30.5
10871	54	71	43.5	111	45	49	39	63	103
10872	43	43	35	108	52	35	19	45	70
10873	43.5	45	30	102.5	35	37	22	44	55
10874	39.5	53	35	106	37	40	22	43	78
10875	36	33	28	124	27	32.5	14	32	36
10876	79	38	25	84	26	57	20	31	62
10877	54.5	44.5	35	124	35.5	48	22	43	49
10878	47	30	21	93.5	26	28.5	20	41	53
10879	41	38	25	92	44	31	17	41	47
10880	40.5	40.5	32	102.5	30	36	21	50	58
10881	43	31	34	92	33	33	23	32	52
10882	44	35	26	107	34.5	33	15	34	51
10883	53	40	29	93.5	40.5	36.5	22	43	55
10884	35	38	25	90	38	34	17	36	54
10885	42	45	28	84	43	37	20	42	51
10886	40.5	39	23	85.5	26.5	30	18	48	53
10887	30	27	22	81.5	29	27	14	28	46
10888	57	45	37	119	31.5	41	21.5	48	51
10889	42.5	41	34.5	93	37.5	33	24	63	53
10890	53	63.5	30	88	57	52	33.5	52	60
10891	25	22	22	86	39	24	12	25	26
10892	67.5	30	31	84	31	29	23	33	35
10893	35	39	31	90	71	28	18	45	46
10894	32	33	25	77	23.5	28	16	35	53
10895	70	41	36	124	31	32	20.5	44	40
10896	54.5	39	28	87	46	34	19.5	77.5	53
10897	39.5	35	22	89.5	26	32	15	33	50
10898	41	54	30	115	34	40	23	51	62
10899	56	74	41	113	34.5	56	27	60	72.5
10900	47	51	33.5	127	31	39	23	53	96
10901	48	56	33.5	89	31.5	38.5	23	53	58
10902	45	39	24	88	28	36	18.5	35	43

10903	60.5	43	23	67	26	54	17	40	52
10904	40.5	51	41	96	37.5	33.5	23	46.5	73
10905	48	44	31	114	31	37	21	51	49
10906	48	44	29	89	37	34	19	52.5	56.5
10907	37	24	18	106	21	29	11	25	61
10908	43	15	10	46	16	19	9	67	13
10909	19	13.5	14	66.5	16	19	13	18	31
10910	28	16	19	105.5	20.5	23	12	35.5	28
10911	21	13	11	47.5	17	19	7	17	10
10912	25	14	13	66	17	20	7	15	9
10913	26.5	17	18	62	21	23	11	20	26
10914	48	16.5	26	98	23	21	13	38	44.5
10915	24	14	10	53	20	17	9	13	17
10916	26	17	23	58	26	21	10	21	24
10917	36	15.5	15	81.5	42.5	32	55	20	57
10918	24	17	16.5	57.5	24	20	30.5	26	25
10919	48	15	15	60	24	20	12	100	28
10920	18	14	11	19	13	18	7	14	9
10921	25	13	14	44	18	16	8	17	15
10922	20	12	10	31	25	18	7	14.5	10
10923	23	13	11	54	23.5	18	17	16	32
10924	23	16	14	39	15.5	20	9	12	37
10925	16	12	9	46	14	16	6	10	9
10926	62	21	11	72	17.5	60.5	7	16	17
10927	36	15	27	65	66	20	10	14	19
10928	62.5	14	11	39	21	20.5	11	48	12
10929	18	13	14	80	21	19	7	58.5	9
10930	23	13	14	50	35.5	19	8	59	10
10931	102	28	24	57	27	51	15	36.5	37
10932	74	26	27	108	51	37	22	36	39
10933	203	24	25	72	45.5	35	42	167	26
10934	47	17	22	56	62	24.5	14	117.5	21
10935	39	16	15	36.5	24	22	16	29.5	19
10936	40	18	19	37	21	23	12	19	18
10937	112	16	12	45	27	24	11	16	23
10938	19	11	11	49.5	22	13.5	8	12	10
10939	21	14	13	54	16	16	9	19	9
10940	16	7	9.5	31	23	10	9	18	6
10941	29	14	23.5	142.5	21	18	16	27	28.5
10942	15	12	9	35	13	15	6	10	8
10943	17	14	9	47	14	16.5	8	11	10.5
10944	24	13.5	12	27	19	16	14	16	12
10945	29	19	13	36.5	24	21	18	30	17.5
10946	31	14	16	73	23	24	33.5	18	118
10947	52	23	23	57	49.5	26	26	35	37
10948	30.5	13	14	37	22	18	10	15	19
10949	82.5	19	28.5	93	356	24	12	27	34

10950	43.5	20	25.5	49	24.5	33	15	33	19
10951	64.5	15	22	57	30	20	28	25	43
10952	65	19	15.5	107.5	171	27	19.5	44	21
10953	73	16	367	72	30	20.5	12	30	17.5
10954	24	19	16	63	18	21	15	20	20
10955	37	33	61	102	20	27	12	34	71
10956	23	26	16	55	16	20	11.5	22	27
10957	36	16	20	156.5	37.5	18.5	23	28.5	34
10958	55	20	22	68	35	28	12	91	12
10959	17	10	9	35.5	16	14	15	12	7
10960	37	15	16	98	61	19	17	33	76
10961	21	13	12	43	34.5	22	9	14	17
10962	877	129	115.5	205.5	485	151	286.5	451	129.5
10963	34	13	14	53	26	21	12	26.5	27
10964	30	16	19	66.5	23	23	11	24	100
10965	33	23	20	60	22	25	13	23	51
10966	142.5	16	16	51	22	24	14	107	30
10967	48.5	32.5	26	68.5	53.5	31	23.5	37	82
10968	22.5	13	14	75	22	18	10	34	42
10969	39	13	32	145	23.5	22	12	40	61
10970	20	12.5	19	41	21.5	17	13	21	16
10971	26.5	21	15	119	31	20	9	39	9
10972	28	28	21	68	24.5	22	15	33	30
10973	35.5	41	29	83	27.5	29	18	44	41
10974	39	53	27	103.5	28.5	36	25	52	62
10975	35	28	28	89	39	24	14	29.5	35
10976	98.5	54	28	97	32	35.5	24	59	56
10977	43	42	25	92.5	29	33	24	41	48
10978	26	29	19	69	19	23	12	32	31
10979	31	35	19	76	22.5	27	16	35	49
10980	48	49	29	90.5	29.5	40	23	49	50
10981	162	26	21	91.5	20	29	13	31	33
10982	25	30.5	25	59	24	24	16	30	28
10983	39	51	36.5	91	33.5	33	19	45	52
10984	37	22	21	103	27	23	11.5	25.5	32
10985	50	49	30	119	28	35	26	56	53
10986	43	64	40	128	33.5	45	28	60	69
10987	37	58	28	103.5	27	31	22	53	55
10988	35	48	30	106	93	35	24	48	99
10989	34.5	39.5	23	70.5	26.5	27	19	45	46
10990	20	21	17	67	23	19	16	21	26
10991	33	28	43	143	23	26	15	38	31
10992	26	32	18.5	71.5	19	24	15	34	34
10993	50	44	22	82	23	39	21.5	39	51
10994	34	39	23	95	31	31.5	17	38	44
10995	32	41	24	84.5	27	28	18.5	40	58
10996	45	55.5	31	126.5	47	39	28	58	56

10997	38	41	26	94	26	33	18	38	53
10998	57	43	28	89	27	46	28	41.5	52.5
10999	45	51	29.5	89	32	32	26.5	49	63
11000	28	33	22	108	20	26	14	36	26
11001	37	29	20	80	25	23	15	32	50.5
11002	26	24	19	85.5	19	19	13	30	24.5
11003	38.5	25	27	103	27	24	18	35	27
11004	26	43	22	81	24	28	18	40	43
11005	50.5	24	19	71	23.5	22	12	30	26
11006	49	52	30.5	95	25.5	40.5	21	54	63
11007	40.5	35	26.5	80	27.5	27	16	37	32
11008	33.5	52	31	96	30	31	19	56	60
11009	50	55	29	81	39	54	21	50	71
11010	38	31	21	78	25	34	15	36	42
11011	31	31	23	88	24	24	17	47	42
11012	28	27.5	21	67	23	23	15.5	34.5	46.5
11013	39	49	27	120.5	32	27	20	56	50.5
11014	46	53	29	99	29	49	21	58	52
11015	41.5	39	28	88	24	33	18	56.5	39
11016	26	24	17	78	20	23	14	24.5	24
11017	35.5	23	17	64	26	32	12	25	24
11018	25	37	23	70	54	24	17	36	42
11019	28.5	36	19	75.5	36	24	13	31	34
11020	19	22	14	64	20.5	19	10	23	20
11021	47	47.5	27	93	31.5	35	23	45.5	64
11022	37	46	25	90	27	31	20	43	52.5
11023	60.5	43.5	18	75	23	58	15	33	46.5
11024	22	21	17	62	22	20	12	27	29
11025	44	70	27	113	32	38	22	66.5	51
11026	26	23.5	16	60	22	22	13.5	26	24
11027	20	14.5	19	62	18	17	12	28	19
11028	30.5	24	22	82	21	19	14	32	34
11029	35.5	18	16	77.5	18	22	9.5	23	35
11030	28	25	23.5	79	26	21	15	32	32
11031	46.5	30	27	98	38.5	24	36	37	102.5
11032	30	19	12	68	33.5	26	11	27	13
11033	159	35	104.5	95	100	41.5	60	76.5	144.5
11034	13	16	11	56	24	17	8	14	14
11035	41	23	23	134	40	28	23.5	57	81
11036	27	19	14	40	42.5	19.5	13	16	31
11037	24	17	16	54.5	25	21	12	15	32
11038	19	14	15	73	26	19	18	18	45
11039	15	16	10	42	26.5	18	13	11	9
11040	20	16	12	49	42	21.5	8	10	11
11041	31	19	14.5	37	29.5	23	10	18	27
11042	21.5	16	12	61	34	18	37	17	10
11043	24.5	20	29	44	34.5	19	14	22	52

11044	24	19	30	57	48.5	24	12	14	17
11045	28	19	18	82	20.5	25.5	17	14	10
11046	28	32	21	95	33	25.5	15	31	25
11047	57.5	52	29	107.5	41	47	22	49.5	74
11048	41	56	32	123.5	38.5	45	53	62	60
11049	32	51.5	31	122	35.5	35	24	53	66
11050	33	49	32	130.5	54	31	25	50	58
11051	41	69	45	111	48.5	44	31	73	78
11052	40	40.5	31	114	36.5	31	24	44.5	49.5
11053	39.5	66	39	118.5	44	41	34	64.5	94
11054	36	69	36	123	39	42	28	67	75.5
11055	50	71	38	141	59	45	28	81	117
11056	42	67	38	124	45.5	42	34.5	75	71
11057	29.5	47	27	103.5	30.5	32	23	46.5	44
11058	48	46	32	120	35	34	22	55	49
11059	35.5	29	19.5	87	31	35	17	28	38
11060	44	37	31	126	38.5	33	22.5	42	45.5
11061	48	77	48	144	46	50.5	40	73	79
11062	35	51	29	106	32	34.5	23	51	47
11063	42	27	26	84	37.5	44	21	52	42
11064	32	43	26	117.5	33.5	31.5	21	45	63
11065	37	51	31	141	34.5	32	24	57	46
11066	24	24	24	107	28	22	14	30	27
11067	19	13	15	86	41	19	10	14	13
11068	20	14.5	13	54	64	17	13	22	12
11069	35	14	13	68	30	16.5	11	24	10
11070	20	13	11	62.5	27	16	10	24	12
11071	14	12	14	47	21	13.5	8	13	9
11072	16	13	13	56	25	14	9	15	10
11073	14	13.5	10	54	23.5	16	7	15	10
11074	195	17.5	22	98	38	28	15	70	21
11075	50	47	36	70	43.5	32	27	67	41
11076	29	19	19	101	144	20	41	30	58
11077	35	19	25	117.5	38.5	20	27.5	31	62
11078	20.5	16.5	13	72	52	22	14	53	17
11079	30	17	11	19.5	25	21	9	21	13
11080	47	18	25.5	130	44.5	21	16	143	21
11081	28.5	19.5	15	67	27	24	9	21	18
11082	17	14.5	34	96	26	17	11	24	19
11083	22	17	17	55	98	17.5	14	20	18
11084	26	41	22	73	30	28	10	19	51
11085	20	16	18	50	30	18	11	16	26
11086	14.5	11	13.5	71.5	24	13.5	7	14	23
11087	23	17	13	50.5	26	19	13	17	15
11088	82	69	39	103	38	69	24	60	126
11089	70	78	130	120	45	49	36	60	78
11090	61	88.5	47	113	51	56	31.5	70	81

11091	44	58	42	139	44.5	46	27	58	73.5
11092	55	86.5	41	120	38.5	49	40	81	69
11093	68	65	57.5	115	51.5	52	35	66	169
11094	45	63	41	116	50.5	47	33	62	63
11095	55	87	50	118	78	59.5	33	73	89.5
11096	58.5	74	45	93	54	52	34	66	77
11097	47	69	38	91	47	53	25	57	60
11098	48.5	52	31	72.5	37	41.5	25	72.5	75
11099	50	67.5	37	91	42	49.5	28	63.5	68
11100	60	63	34	101	42	45.5	31	54	65
11101	53.5	73	42	97.5	47	63	30	64	73
11102	44	64	40	96	36.5	47	27	54	56
11103	47.5	69	37	99	46	53	30	56	66
11104	35	42	22	71	38	34	18.5	34	39
11105	64.5	68	37	94	50	48	27	60	76
11106	67.5	75.5	49	114	51.5	51.5	31	69.5	83
11107	53.5	79	49	117	51	48	37	70	76.5
11108	51.5	76	45	95	71	54	60	83	86
11109	44	57	38	134	48	44	27	57.5	101.5
11110	48	66.5	37	100	47.5	51.5	30	63	87
11111	49	76	41	123	49.5	47	30	62	99
11112	53	57	41	94	40	47	25	54.5	61
11113	48	68.5	52	90	42	52	31	58	76
11114	43	79	42	96.5	47	48	32	59	82
11115	48	69.5	40	105.5	45.5	39	31	77	80
11116	38	55	34	88	41.5	39	30	50	70
11117	15	14	11.5	22	21.5	20	8	14	9
11118	12	13	9	10	15.5	18	6	8	8
11119	13	14	13	26	19	19	7	11	9
11120	15	14	14	51	16.5	19	7	10	9
11121	16	13	9	30	18	18	7	16	9
11122	27	31.5	24	87	29	31	14	30	33
11123	17	14.5	22	50	28	21	8	13	13
11124	19	15	16	71.5	18	20	8	14	13
11125	52.5	14	14	70.5	21	19	8	15	11
11126	16	12	12	56	20	18	7	15	9
11127	14	11	11	28	21	17	8	10	8
11128	14.5	14	18	44	19	18	6	11	10
11129	14	13	11	47	15	17	7	13	12
11130	21	21	16	51	20.5	23	9	15	15
11131	16	13	12	48	20	20	8	17	12
11132	617	143	198	186	311	185	196	316	133
11133	22	16	29	72	29	23	11	18	16
11134	25	17	15	43	30	24	15	17	14
11135	15	11	13	56	21	18	7	12	16
11136	15	15	13	36	20.5	21	7	11	10
11137	28	14	17	60	43	19.5	18	17	16

11138	29	150	39	64	34	24	46	29	220.5
11139	30.5	32	47	72	313.5	25	12	31.5	20
11140	38	21	37	63	392	31	18	25.5	27.5
11141	498.5	145	122.5	180.5	165	132	133	412	150
11142	17	14	19	63	39	20	15	14	14.5
11143	28	21	43.5	47	37	27	11	15	19
11144	16	15	14	36.5	28.5	20	8	12.5	15
11145	25	17	18	132	34	21	16	24	63.5
11146	22	15	19	71	46	19	10	18	41.5
11147	27	16	20	54	30.5	18	9	19	31
11148	32.5	12	12	46	30	19	8	12	10
11149	42	17	22	112	46	25	20	34	16
11150	27	16	21	104	30	22	11	23	16
11151	37	21	27	138	33	30	14	31	42
11152	42	15	15	36	35	21	9	14	23
11153	46.5	19.5	19	62	40	40	12	51	22
11154	31	20	18	24	78	26	15	96.5	16
11155	76	25	26	88	66	41	17	25	33
11156	67.5	21	29	132	34	27	130.5	71	46.5
11157	34	15	21	89.5	30	21	10	23	28
11158	31	15	17	67	46	23	14	48	24
11159	63	24	31	142	46	26	15	42	44
11160	21	19	21.5	60	35.5	19	17.5	28	26
11161	18	12	9	33	24	19	8	16	8
11162	20	11	13	60	25	14	8	14	21.5
11163	57	22	18	73.5	44.5	21	31	33	24
11164	14	13	8	20	22	14	6	12	11
11165	16.5	11	10	21	21	15	7	12	7
11166	22	15	17.5	103	28	18	10	20	23
11167	39	15	17	29	34	21	12	27	16
11168	29	16	20	43	33	25.5	24	72	36.5
11169	35	18	24	81	41	26.5	16.5	33	60
11170	23	11	11	42	22.5	15	7	11	13
11171	18	14	13	58	34	17	8	17	33
11172	59	16	33	167	40	21.5	13	66	17
11173	62	16	18	89	38	26	12	80	39
11174	23	12	19	46	36	17	9.5	17	25
11175	47	18	19	84	25	35	16	23	73.5
11176	35	13	12	30.5	30	19	10	21	12
11177	25	22	15	39	23	28	9	13	18.5
11178	27	15	12	40	36	20	11.5	13	12
11179	64	72	37	77	44.5	46	28	57	60
11180	39.5	45	33	67	39	36	22.5	45	40
11181	71	56	36	79.5	50.5	37.5	25	53	62
11182	192	65	42	89	40	57	29.5	59	78
11183	36	64	28	75	35	36.5	24	45	60
11184	31	38.5	24	70	30	27	19	43	38

11185	65.5	58	34	97	29	63	23	52.5	54
11186	55	66	35	90	34	61	28	52	67
11187	48	46	30.5	62	36.5	46	30	41	92
11188	44.5	66	45	103	37.5	43	28	59	64
11189	29	43	26	64	32	30	23	40	55
11190	44	54.5	28	89.5	46.5	35.5	23	52	51
11191	32	39.5	25	76	37.5	33	18	38	49
11192	22	24	19	52	31	24	13	22	30
11193	72	75	47	137	43	49	30	69	85
11194	33	47	29	78	39	35	19	40	52
11195	46	42	25.5	107	36	34	24	47	46
11196	29.5	46	29	72	33	35.5	21	42	50
11197	39	41	33	87	35	35	24.5	47	48
11198	43	57	39	97	50	41	27	56	88.5
11199	69	62	40	90.5	39	65	29	54	76
11200	31	39	23.5	81	35	30	20	37	36
11201	43	61	31	107	41	40.5	25	56	124
11202	53	85	42	90	36	48	35	70	83
11203	58.5	56	41	113.5	42	42	30	62	60
11204	35.5	39	26	92	30	38	19	44	53.5
11205	29	27	23	69.5	29	29	14	36	25
11206	27	22	20	69.5	26	22	13	24	72
11207	32	47	29	78	53	33.5	22	45	62
11208	43.5	58	32	105	32	37	24	55	53
11209	121.5	47	27	72	29	54	19	41	44
11210	33	34	20	58	25	27	15	31	41
11211	30	34	24	77.5	50.5	25	34	30	36
11212	74	50	30	81	39	38.5	27	44	63
11213	48	50	29	91	37	42	21	47	64
11214	30	36	24	66.5	26	30	20	32	37
11215	40	44	29	68	32.5	32	22	45	59
11216	30	47	25	61.5	28	31.5	19	43.5	47
11217	30	39	27	67.5	39	35	18.5	34	42
11218	42	51.5	31	80	33	34	22	52	65
11219	37	49	25	62	33	36	22	41.5	42.5
11220	58	64.5	36	90	42	43	31	62	90
11221	39	45	26	73	32	37	20	41	41
11222	53	74	46	97	48.5	51	37	71	68
11223	32	55	30	89.5	43	34	23	49	58
11224	26	31	22	81	49	25	16	33	41
11225	43.5	44	29.5	74.5	64	39	22	40	65
11226	41	63.5	38	99	37.5	39	24	54	61
11227	21	12	11	30	22	17	8	16	10
11228	25	11	10.5	40	22.5	15	9	14	9
11229	26.5	12	13	61	24.5	17	9	16	9
11230	22	11	10	24	20	14	7	9	6
11231	35	20	23	114	30	31	10	25	32

11232	23	12	19	70.5	26	18	5.5	18	25
11233	54	26	27	107.5	36	32	12	47	38
11234	171	45	25	102.5	38	143	19	33.5	89
11235	59	20.5	14.5	58	34	26	12	30	30.5
11236	28	22	63	51	34	27	34	30	60.5
11237	54	69	48	105	67	52.5	55	76	75
11238	41	67	40	100	51	44.5	31	60	75
11239	40.5	72	43	101	46	53	35	65	73
11240	44.5	76	57	119	46	51	37	77.5	86
11241	54.5	99	56	108	49.5	65	43	84	84
11242	55.5	63.5	39	95	46	44	30	54	69
11243	37	58	33	86	39	43	27	52.5	60
11244	47	74.5	40.5	113	46	51	37.5	77	87
11245	39	75	37	121	43	53	32	66	92.5
11246	38	56	34	99.5	37.5	39	27	52	59
11247	32	39	30	98.5	29.5	31	22	43	71
11248	49	74	43	120	49	51.5	33	69	76
11249	51	70	40	110	44.5	49	32	62	184
11250	31.5	50	31	76.5	32	39	23	44	45
11251	38.5	45	30	84	36	34	29	46	47
11252	56.5	96	57	109	52.5	66	47	79	90
11253	28.5	43.5	27	84	31	30	21	42	40
11254	52.5	81	57.5	126.5	47	53	37	70	83
11255	30	24	19	93	32	34	27.5	24	92.5
11256	23	20	22	75	29	22	10	23	16
11257	21	21	18	68	25.5	20	12	20	23
11258	17	17	14	38	28	21	9	14	20
11259	24	16	18	91	29	18	37	18	18
11260	41	29	18.5	57	32	42	14	25	31.5
11261	19	17	15	30	30	23	15	19	20
11262	26.5	19.5	16	29	31	25.5	17	31.5	23
11263	26	17	25	106.5	55	21	16	33	118
11264	30	19	22	79	33	21	22	23	36
11265	30	26	56	33	34.5	28	16	29	28
11266	50	27	27	99.5	39	35	18	39	69
11267	47	21	26	107	44	23.5	19	51	93
11268	21	23	16	33	30	20	12	19	21
11269	27	17	16	51	30	27	14	27	30
11270	25	20	21	60	34	29	12	25	38
11271	63	18.5	27	120	43	25	18	26	41
11272	19	17	15	47	25	19	10	19	13
11273	12	14	13	38	23	16	7	15	9
11274	52	26	38	146.5	46.5	32	33	37	22
11275	54.5	80	40	109	41	55	41	59	69
11276	46	53	38	91	30.5	47	23	46	47
11277	76.5	60.5	34.5	110	39.5	52	25	48	56
11278	83.5	100	59.5	153	61	65	42	90	89

11279	58.5	70	42	103	79.5	52	35	67	80
11280	40	54	32	80	47	42	23	40	51
11281	38	47	29	78	41	39	27	41	46
11282	56	77	44	95	47	58	32	62	69
11283	88.5	62.5	38	98	36.5	45	27	45.5	65.5
11284	51	73	46	101	49	56	29	57	69
11285	56	55	51	92.5	53.5	54	34	59	68
11286	75.5	81	52	142	79.5	61	46	77	99
11287	61	75	47	104.5	51.5	57	38	81	88
11288	52	84.5	32	92	30	40.5	25	46	70
11289	44	55.5	31.5	76	32	44	19.5	48	59
11290	44	56	37	108.5	44.5	41	29	52	58
11291	57	77	99	108	47.5	47	34	74	81
11292	77.5	71	46	107	41.5	49	31	68	81
11293	54	98.5	52	117	50	65	49	85.5	87
11294	45.5	67	39	94	90.5	49	34	56	64
11295	54	59	32	101.5	32	56	25.5	49	55
11296	59	73	44	116	47.5	52	33	63	75
11297	45	72	40	99	51	54	37	63	66.5
11298	43	54	35	98	33.5	37	28	48.5	55
11299	63.5	86	43	107.5	45.5	58	29	73	94
11300	48	69	40	95	42	47	35	59	66
11301	32	20	25	69.5	51	27	16	30	14
11302	34.5	17.5	19	83.5	23	30	20	22	13.5
11303	49.5	38	37	145.5	32	37	19	43	73
11304	32.5	23	22	87.5	37	34	19	44	26
11305	26.5	21.5	17	53	31.5	26	11	17	18
11306	16	13	11	34.5	21	20	8	13	10
11307	21	14	12	39.5	20	20	8.5	15	15
11308	35	20.5	24	112	29.5	26	21.5	38	23
11309	24	14	19.5	65	34.5	19	10	15.5	18
11310	20	12	17	25	30	17	9	14	8
11311	29	18	16	63	28	29	9	15	14
11312	29	19.5	21	50	30.5	26	13	19.5	27
11313	114	17	20	81	25	21	12	21	12
11314	69.5	52	42.5	273	40	44	37	76	53
11315	19	14	16.5	63	18	16	7	18	10
11316	20.5	15	15	61	25.5	20	10	18	15
11317	18	14	14	49.5	28	19	9	12	10
11318	13	13	9	12	14	18	7	11	8
11319	15	15	8	13	15	19	7.5	10.5	8
11320	15	14	10	13	16	20	8	11	10
11321	22	18	16	84	19	22	9	14	14.5
11322	23	17	17.5	81	20	18	9	19	11
11323	18	13.5	12	43	22	16	10	17	8
11324	16	13	13	63.5	22	17.5	8	12	8
11325	26	15	15.5	87.5	24	23	10	24	14

11326	15	12	10	16	18.5	18	8	10	7
11327	24.5	14	11	50	20	18	7	11	8
11328	18	14	12	35	20	19	9	14	9
11329	31	17	19	44	33	25	10	16	14
11330	41	19	129	67	42	26	53	46	147
11331	58.5	21	22	87	38.5	32	19.5	138	17
11332	28.5	18	19	71	31	21	16	78	79
11333	32.5	19	15	48	27.5	24	12	29	16
11334	84	20	25	122	35.5	38	14	39.5	91
11335	40	21	19	35	29.5	29.5	15	35	20.5
11336	26	15	20	99	23	19.5	10	21	63
11337	16	10.5	9	37	15	16	7	14	7
11338	24	14	13	51.5	26.5	17	11	15	9
11339	30	22	18	61	26.5	24	17	38	19
11340	14	14	8	23.5	18	19	6	10	9
11341	25	16	14	35	22	23	8	14	23
11342	74	15	12.5	34.5	38	21	10	28	14
11343	30.5	14	14	30	28	22	11	15.5	11
11344	106	19	15	43	22	39	10.5	14	14
11345	34	23	19	48	81	30.5	13	29	15
11346	30.5	16	16	71	52	23	10	33	13
11347	17	12	11	39	35	17	8	12	17
11348	20.5	12	9	17	18	18	6	10	8
11349	26.5	14	14	22	24	22	9	14	11
11350	21	13	21	106.5	33	20	13	20.5	13
11351	39	19	31	158	46	27.5	36	33	23
11352	19	12	11	29	21	16	8	11	9
11353	16	12.5	11	32	18	17	9	13	8
11354	17.5	13	11	56.5	20	17	8	11	10
11355	18	10	10	31.5	18	17.5	7	24	16
11356	23	19	24	54	26	20	10	20	25
11357	31	15	14	31	26	20	11	24	13
11358	99	15	16	47.5	31	20	19	101	16
11359	27	13	13	37.5	26	19	10	19	13
11360	22	12	14	26	20	18	10	25	9
11361	26	14	18	79	23	23	10	30.5	15
11362	65.5	45	22	47.5	24	21	11	18	16
11363	23	14	13	25	33	19	11	14	12
11364	19	13	12	48	23	19.5	7	12	10
11365	17	13	12	29.5	25	17	9	18	10
11366	147	15	20	148.5	23	20.5	15	142	11
11367	28	15	15	28	31	19	11	86.5	14
11368	32	12	12	23	29	21	23	40	11
11369	28	12	17	35	31	21	10	33	11
11370	28	18	18	41	40	20	22	51.5	14
11371	122	14	15	62.5	33	20	13	26.5	44
11372	37	13	19	84.5	26.5	19	29	43	13

11373	40	65	20	64	112	31	17	34	42
11374	34	14	15	30	27.5	18	14.5	20.5	12
11375	21	15	12	33.5	222	20	8	14	15
11376	32.5	18	27	68.5	33	22	14.5	40	37.5
11377	31	13	15	36	24.5	16	11	15	17
11378	18.5	16	14	28	24	17	9	14	10
11379	31	20	16	76	94.5	20	11	21	16
11380	53	57	36	96.5	32	37	28	57	63
11381	41	61	38	95	40	40	31	62	55.5
11382	29	33.5	22	60.5	30	29	18	34	27
11383	37	61	31	96	37.5	43	26	59.5	62
11384	50	54.5	27	85.5	34	59	19	47	54
11385	38	56.5	28	76	32.5	32	23	57	54
11386	34	42	24.5	59	28	31	18	48	63
11387	54	62	38	107	41	43	29	61	78
11388	43	57.5	34	89	35	42	24	56	64.5
11389	41	54	33	124	34	41	29	57	54
11390	48.5	81	42	82	44	48.5	35	74	77
11391	49.5	66	79	90	40	53	29	57	67
11392	46	71	36.5	91.5	37.5	42.5	30	64.5	67
11393	41	64.5	34	87.5	36	42.5	28	62	113
11394	30	44	26	68	34.5	30	19	40	47
11395	46	67	38.5	101	40.5	41	30	61	79
11396	57	64.5	38	97	38	51	29.5	63	67
11397	43	54	35.5	101.5	35	37	30	56	64
11398	47	74	43	101	52	46	38	70	102.5
11399	53.5	72	44	103	39	59	31	65	75
11400	52	60.5	35	106	34	46.5	31	56	62
11401	31	37	25	67	32	28	19	41	53
11402	44	80.5	39	128	40	47	32	74	87
11403	36	47	29	109	27	34	20.5	46.5	45
11404	54	57	35	100.5	47	42	25	57	70
11405	33	41.5	31	78	30	29.5	20	55	46.5
11406	46	64	35	98	38.5	42	31	59	71
11407	33	45	29.5	86	32	30.5	20	49	49
11408	44	54	34	84.5	32	37	25.5	55	62
11409	40	61.5	38	89.5	38.5	41	28	55	66
11410	47	67	39.5	112.5	37.5	41	30	65	71
11411	47.5	78	42	109	47	48	34	72	84
11412	62	74	49	90	47	46	32	74	76
11413	29	40	26	82	33	30	20	41.5	41
11414	34	50.5	34	70	28.5	33.5	22	52	61
11415	41.5	58	32	89	31.5	44.5	25.5	50	54
11416	41	70	33.5	86	35	40.5	29	61	63
11417	22	12	38	90	56.5	17	13.5	19	36
11418	20	14	13	49	23	15	8	12	34
11419	21	14	19	54.5	18	16.5	8	22	11

11420	20.5	11	11	16	18	15	8	16	11
11421	17.5	14	12	51	20	19	7	22	11
11422	14	10	15	24	17	14	6.5	9	9
11423	14	10	10	21	25	13	7	9	8
11424	14	11	10	25	18	14	6.5	9	23
11425	13	10	5	11	8	15	4	4	6
11426	11	11	8	12.5	15	15	5	8	6
11427	24	11	17	40	17	17	8.5	11	11
11428	13	9	11	17.5	12	13	6	9	7
11429	21	20	14.5	54.5	14.5	29	9	11	12
11430	20	20	13	38	15.5	24.5	10.5	13	13
11431	19	18	12	29	14	23	8	12	15
11432	18	19	13	53	18	26	8	13	13
11433	23	23	18	81.5	20.5	28.5	10	19	23
11434	40	25	17	39	25	30	14	43	23
11435	65	27	34.5	120	32.5	29	16	38	77
11436	28	23.5	29	101	202.5	27	13	33.5	57.5
11437	29	21	25.5	100	49	28	10	18	23
11438	55	25	23.5	112	25	28	12	33	36
11439	35	20	33	113	41	29	10	28	31
11440	33.5	23	20	92	25	26.5	11	21.5	38.5
11441	56	28	21	102	20	47	11	20	32.5
11442	31	23	22.5	90	22	28.5	15.5	32	48
11443	30	25	25	102.5	47	28	11	25	43
11444	35.5	24	26	98	28.5	28	13	27	36
11445	41	26	31	114	28	32	17.5	27	37
11446	34	23	20	105	23	25	12	25	42
11447	34	26	31	104.5	28.5	27	14	34	22
11448	34	20	24	119	26	28	11	27	36
11449	36	21	22	104	24.5	28	11	25	27
11450	41	23	25	114	38.5	28	12	35	45
11451	38.5	22	36.5	123	27	25.5	12	34	58.5
11452	39	21	23	81	17.5	25	12	27	24
11453	30.5	23	21	99	25	25	19	24.5	23
11454	27	21	17	98	22.5	31	14	18	18
11455	24	20	16	69	26	24	16	19	20
11456	106.5	20	22	85	43	32	13	53	28
11457	19	19	14.5	87	14	21	8	17	11
11458	18	19	15	58	20	22	9	25	11
11459	44	58	47	92.5	44	53	29	51	83
11460	47	74	52	84	44	55	33	58	84
11461	52	70	42	89	46.5	54	31.5	54	73
11462	44	61	40.5	69	48	49.5	30	47	62.5
11463	50	88	54	111.5	53.5	58	39	78	94
11464	73	50	29	94	43	75	18	38	40
11465	47	77.5	57	124	60	54	33	77	84
11466	51	76	65	128.5	47	57	34	62.5	112.5

11467	39	56	34	91	38	45	24	46	59
11468	57.5	85.5	50	102	44.5	60	44	76	93
11469	39	54	38	77	37	42	22	48.5	53
11470	52	46.5	32	71	49.5	38	23	48	45
11471	35	47	39	103	39	36.5	19	37	77.5
11472	44	67	36	102	46	49	29	61	77.5
11473	40.5	66	43	101	42	47	28.5	58	69
11474	43	49	37	92	51.5	43	24	69	43
11475	48	76	42	103	52	54	28.5	68	66.5
11476	41.5	65	40	80.5	42	43	29	58	84
11477	37	53	31	80	41	41	21	38	72
11478	57.5	60	42	86	42	47.5	30	52	58
11479	61.5	64	50	120	58	42	32	62	65.5
11480	41	73	46	110	51	53	33	61	71
11481	43	69	43	99	44	51	28	60	79
11482	34	46	32	80	35	38	25	42.5	43
11483	51	63	37	110	41	49	36	57.5	60
11484	41.5	66	40	91	44.5	49	30	60.5	76
11485	42.5	56	35	93	39	42	26	55	53.5
11486	52	81	49.5	96	48	58	33	70	86
11487	58	55	36	105	35	45	23	53	71
11488	28	18	25	68.5	28	25.5	12	17	20
11489	57	75	46	99	44	58	34	64	71
11490	52	68	44.5	109	55.5	49	33.5	59	74
11491	61	59	44	108	46	46	28	68	82
11492	48	74.5	51	116.5	51.5	47	34	71	98.5
11493	53.5	80	58	122	51.5	54	42	71	74
11494	51	56	39	86	39	53	24	48	62
11495	46	80.5	50	107	54	60	42	73	86
11496	40	47	33	119	33	40	22	43	46
11497	46.5	20	26	114	49.5	25.5	16	32	22
11498	36	19	22	98	32	29	14	21.5	26
11499	35	25	39	169	47	26.5	32	51	22
11500	32	17	36	129.5	30	24	17	26	20
11501	45	20	19	73	34	23	45	20	33
11502	28	16	22.5	58	47	18	17	18	37
11503	57	24	75	82	55.5	41	15	23	94
11504	42	17	33	124	42	23	15	38	54
11505	34	16	20	66	69	21	41	17	27
11506	35	24	31	104	44	29	36	63	30
11507	36	19	19	57.5	60	22	25	16	30
11508	32	17	29	75	44	24	17	78.5	222
11509	14	13	11.5	18	21	18	7	10	11
11510	15	13	10	16	31	20	7	10	12
11511	25	20	17	27	28	28.5	13	12	16
11512	16	13.5	11	26	27.5	19	7	11	12
11513	19	15	11	21	27	20	8	12	12

11514	17	16	13	20	26	23	8	9	13
11515	42	23	19	47	32	41	21	18	37
11516	41	19	21	61.5	31.5	28	24	18	69
11517	14	16	13	15.5	40	23	7.5	10	71
11518	25	16.5	20	27	32.5	23	10	33	34.5
11519	22	15	18	44	34	20	10	14	19
11520	23	16	15	36	42	24	10	15	14
11521	18	13	11	20	29	20	9	13	25
11522	22	11	10	28.5	24	16	6	10	9
11523	22	12	11	38.5	27.5	17	6.5	14	8.5
11524	18	12	33	42	26	16	8	14	9
11525	17	12	10	46.5	25	16	8.5	11	8
11526	32	14	18	33	34	18	31	22	15
11527	35	16	16	34	38	25	12	24	16
11528	57.5	23	35	67	54	36	41	67.5	33
11529	40	19.5	17	37	35.5	43	14	38	17
11530	73	21	18	40	37.5	41	12.5	21	30
11531	55	23	22	105	68	25	25	46	27
11532	61	16	23	81	41	25	14	45	26.5
11533	23.5	15	38	40	43	22	15	34	30
11534	28	15	16	77	29	18	13	15	29
11535	21	14	16.5	75	32	21	8	25	24
11536	20	14	15	85	21	19	9	17	13
11537	37.5	19	35	284.5	34	29	24	46	12
11538	16	13	11	56	21	19	7	11	8
11539	23.5	24	17	61	24	22	11	20	34
11540	23	13	19	59	67	19	11	15	23
11541	24	14	21	97	31	21	10	21	39
11542	34.5	16	25	66.5	35.5	20	11	30	99
11543	33	14	20	82	34	21	13	27	72
11544	31	20	22	90	47	26	20	28	39
11545	26	15	19	58	36	22	16	26	34.5
11546	66	18	23	74	38.5	24	37	33	75
11547	87.5	44	46	296	62.5	63	116	66	53
11548	24	13	18	49	34	21	13	38	29
11549	32.5	25	33	89.5	48.5	32	14	26	161
11550	31	13	12	32	30	19	9	23	18.5
11551	43	66	32.5	97	37	42	27	57	76
11552	40	57	31	94	35.5	39	25	53	62
11553	31	36	28	72	31	29	19.5	34	40
11554	28.5	25	20	59	24	27	12	25	36
11555	42	36	28	66	29	30	20	35	43
11556	48	58	39	103	43	46	28	58	70
11557	51	82	46	114	43	57	33	77	86
11558	27	32	25	62.5	28.5	28	17	43	34
11559	34	45	26	71	40	35	20	42	49
11560	48	51	29	157	32.5	46	25	43	46

11561	40	63	38.5	84	35	43	27	53	57
11562	74.5	69	42	99	45.5	46	34	68	82
11563	908	78	38	84	41	185	27	60	80.5
11564	78	59	38	116	39	38.5	26	56	58
11565	47	65	42	90	45.5	47	31	59	68
11566	42.5	53	34	75	40.5	39	26	52	57.5
11567	46	65	32	76	52	42	23	50	65
11568	34	51	32	74	36	40	22.5	45	57
11569	87	57.5	45	90.5	42	51	36.5	87.5	63
11570	34	41	32	90	38	33	22	40	40
11571	30	43	30	76	39	31	21	41	45
11572	25.5	30	20	57	31	25	14	29	48
11573	40	72	39	88	42	48	31	63	87
11574	45	67	35	95	41	43	29	50	65
11575	38	59.5	35	113.5	37	38	26	51	54
11576	38	59	42	78	48.5	43	27	72	60
11577	37	44	28	69.5	29.5	43	18	35	43
11578	53	59.5	31	83	36	56	25	47	53.5
11579	42	63	40	93	41	48	31	61	69
11580	39	46	31	84	32.5	33	23	44	75
11581	46.5	74	41.5	99	63.5	51.5	36.5	64	72
11582	37	56	34	72.5	36.5	37.5	26	51	56.5
11583	30	35	26.5	79.5	32.5	26	18	38	39
11584	49	52	33.5	73	39	39	26	47	54
11585	26	36	26	67	29	29	18	34	37.5
11586	52.5	68.5	38	100	52.5	46	31.5	56	76.5
11587	42	64	39	81	56.5	45	32	52	67
11588	52	43	30	71	33	33	19	40.5	45
11589	98	39	35	68	34	34	21	40	40
11590	53	57	34	86	43.5	40	25	49	62
11591	33	48	34	90	27	33	22	44	57
11592	52	19	23	109	28	22	14	42.5	17
11593	30	19	18	63	27	23	14	21	25
11594	45	21	22	48	42.5	25	18	26	24
11595	21	18	31	74	21	22	11	22	16
11596	19.5	14.5	16	45	33	17	9	15	10
11597	21	18	27	77	35	19.5	18	21.5	13
11598	27	17	28	105	26	20	14	19	15
11599	43	17	20	59	28.5	21	22	28.5	19.5
11600	19	16	22	33	28.5	20	14	20.5	18.5
11601	25	19	19	54.5	26	25	14	19	19
11602	30	15	14.5	40	20	20	12	75	15
11603	29	16	18	30	19	24	12	18	30
11604	48	20	19	39	27	22	16	103	48
11605	46	17	23	26	29	19	17	24	14
11606	30	16	14	47	21	19	10	20	11.5
11607	16	17.5	14	26	19	20	9	18	10

11608	28	15.5	12	35	18	18	8	42	10
11609	54	22	51	63	39	28.5	19	79	18
11610	21	19	23	57.5	26	19	10	18	21
11611	27	16	16	58	23	18	11	18	16
11612	61.5	20	20	106	31	22	14.5	35	48
11613	13.5	14	10	48	16	17	7	11	11.5
11614	37	55	37.5	70	30	37	26	55.5	48
11615	62	58	36	108	40.5	40	31.5	54	58
11616	37	43	32	81	31	32	24	70	46
11617	89	56	32	95	49	42.5	23	42	47
11618	41.5	67	34	102	35	43.5	31	57	71
11619	43	74.5	41	111	36	45	36	69	100
11620	29	40	42	106	48.5	31	20	42	52
11621	44	39	32	89	32	42.5	21	41.5	49.5
11622	72.5	61	36	102.5	31	55	29	54	55
11623	30	77	28	84	44	29	24	40	62
11624	32	49	29	91	27	33	24	46.5	62
11625	42.5	45	31	95	34	33	24.5	44	70
11626	36	62.5	36	94	37	41	31	57	63
11627	31	45	30	80	26	30	24	42	63
11628	27	27	20.5	60.5	22	23.5	15	30	37
11629	31	49	31.5	80	31.5	33	21	44.5	55
11630	28	47	35	73.5	29	33	22	43.5	46
11631	40	54	32	92	29	36	31	53.5	49
11632	32	40.5	28	78	25	30	21	35.5	38
11633	30	50	32	84	33	36	22.5	45	54
11634	41	50	35	83	30	34	22	46	45
11635	39	70	39	100	31	42	36	57	61
11636	38	54	32	76	30	36	24	50	57
11637	48	87	51	128	60.5	53	43.5	73	76
11638	47.5	68	44	116	42	45	35	65	71.5
11639	37	54	40	97	146	43.5	25	52	69
11640	32.5	47.5	26	82.5	30.5	39	21	41	50
11641	30	54.5	33	72	44	32	22	47.5	63
11642	42.5	67	37	137	32	45	32	55	67
11643	53	87	44	114.5	44	54	39	75.5	87.5
11644	41.5	67.5	35	105	32	46	26	54	58.5
11645	43	80.5	47	101	38	59	38	69	87
11646	30	48	34	74	26	35.5	22	45	53
11647	46	72	40	101	35	48	35	64	67.5
11648	42	78.5	39	96	37.5	46	32	68	91
11649	36	62	34	79.5	28.5	40	27	54	62
11650	33	60	33	80	27	37	23	49	59
11651	30	39	26	74	29	29	18	36	43
11652	51.5	77	44	94	38	56.5	38	65	86.5
11653	35.5	69	40	105	34	40	30.5	66	81
11654	48	81	44	101	36.5	47	37	68.5	88

11655	40	60	38	85	34.5	36	28	55	64
11656	33	54	29	83.5	25	41	23	45	52
11657	95.5	63	42	93	36.5	43	33	68	62
11658	36	52	30	76	27	36	24	48	49
11659	16	12	21	40	20	14	10	15	16
11660	35	58	36	87	27.5	36	33	61	71
11661	45	42	26	66	24	40	19	42	45
11662	30	41	29	95	24	33	18	38	51
11663	18.5	13	20	112	19	17	12	23	13
11664	27	36	25	66	27	28.5	19	35	35
11665	14	17	13.5	37	17.5	15.5	10	14	13
11666	13	12	9.5	44.5	14	14	7	12	8
11667	15	19	14	78	16	14	8	14	9
11668	17	15	10.5	57	15	18	29	15	11
11669	14	11	14	75	15.5	13	8	16	9
11670	11	12	8	61.5	12	14	7	11	7
11671	22	23	55	69	22	27	10	24	32
11672	17	53	16	48	23	21	8	14	13
11673	33	22	21	90.5	22.5	22	10	19	16
11674	12	20	9	15	15	21	7.5	10	11
11675	19	43	14	44	15	25	8	15	20
11676	17	18	16	101	17	21	10	21	12
11677	16	17	18	56	19	22	10	14	11
11678	16	19	15	69	16	23	13	17	12
11679	16.5	20	17	48	18	23	9	15	14
11680	40	21	16	37	39.5	27	11	22	22
11681	34	19	33.5	55	32	25	13	18	26
11682	24	22	19	46	31	28	11.5	29	26
11683	25	20	21	96	42.5	22	15	70.5	37
11684	14.5	19	13	50	19	23	9	10	11
11685	18.5	18	20	69	19.5	20	11	19	14
11686	15	18	13	34	18.5	21	9	9	10
11687	20	18	13	38	17	20	9	16	11
11688	31	18	13	32	20	23	9.5	13	10
11689	67	22	23	47	32	30	15	32	19
11690	15	21	16	39	18	24.5	9	11	12
11691	46	82	45	122	48	55.5	36	74	84
11692	43	71	30	95	42	41.5	30	60	67
11693	51	83	43	144	48.5	55.5	39	69	100
11694	58	78.5	41	118	46	64	32	66	77.5
11695	58	77	40	115	45	42	65	76	68
11696	53	86	47	146.5	63	56	37	82	81
11697	52	59	31	97	32	52	24.5	47	63
11698	43.5	65	39	110.5	42	44	28.5	62.5	77
11699	46	46	30	99.5	46	46	25	46	89
11700	42	77	40	126	51	49	37	70	82.5
11701	58.5	74	84	160	45	58	33	64	69

11702	45	61	28	112	37	41	27	53	58
11703	51	86.5	44.5	143.5	63	54	38.5	78.5	90
11704	39	47	51.5	104	35	35	22	45	64.5
11705	53	68.5	54	151.5	52.5	54	32.5	66.5	68
11706	96	92.5	52	150	53	72	48	95	114.5
11707	58	66	35	132.5	39	52	27	58	70
11708	54.5	80.5	44	131.5	50	57	39	75	81.5
11709	44	64.5	39	127	39.5	45	33	74	59
11710	51	36	41	123	43	35	27	46.5	54
11711	39	60	38	122.5	36	40	32	57	61
11712	48	73	37	126	41	44	32	67	79
11713	49	65	37	124.5	43	45	28	62	70
11714	50	79	44	119	44	52	34.5	67	84
11715	54	55.5	31	108.5	36	58.5	25	52	56
11716	64	77.5	40	123.5	42	52	35	72.5	81
11717	43.5	19	18	50	41	22	27	51.5	22
11718	30	19	16	38	33	21	15	21	15
11719	20	13	14	72	23	18	12	21	24
11720	46	19	21.5	128	31	23	16.5	32	43.5
11721	45	15	25.5	53	39.5	25.5	33	44	19
11722	36	17	18	61	33	19	20	25	14
11723	29	15	20	114	34	23	12	18	17
11724	25.5	17	15	180.5	30	22	11	35	11
11725	49	15	15	82.5	27	21	14	25	15
11726	37	20	50	218	41.5	27	22	99.5	61
11727	23	18	13	45	80.5	19.5	14	30	23
11728	33	20	16	49	153	26.5	12.5	19	16
11729	32	17	15	35	29	23	13	18	22
11730	95.5	18	56	120	39	25	29	29	16
11731	40	16	17	137	30	21	37	27	21
11732	40	19	23	131.5	33	23	20	31	20
11733	46.5	19.5	19.5	104.5	81	27	19	29	21
11734	34	16	15	54	32	21	12	20	13
11735	22	14	15	45.5	31.5	18	12	17	17.5
11736	28	18	15	57	31.5	20	14	22.5	14
11737	65	25	16	132.5	38	64	12.5	38	45
11738	23	16	12	44.5	26	23	10	26	166
11739	11	10	6	19	20	15.5	8	9	7
11740	32	14	12	25	62	22	11.5	14	14
11741	50	23	28	91.5	62.5	26.5	25	38	31
11742	29	16	17	110.5	27.5	23	15	23	15
11743	22	16	16	43	32	19	12	19	29
11744	22.5	12	14	53.5	32	18	11	14.5	14.5
11745	31.5	19	15	60.5	27.5	25	14	22	20
11746	38.5	15	37.5	152.5	67	20	22	38	23.5
11747	29	11.5	8.5	56	20	16	8	16	8
11748	23	14	17	108	25	19	11	22	23

11749	24	14.5	17	91	30	16	39	23.5	47
11750	19	13	17	89	29	17	12	17	12
11751	23	14	15.5	67.5	33	18	11	19	15
11752	18.5	14.5	12	41	25	20.5	14	24	11
11753	35	22	23	119.5	921	29	20	28.5	51
11754	41	16	20	122	74	25	13	28	36
11755	49	16	15	110.5	38	35	11	24	29
11756	48.5	18	27	131.5	33	22	22	65	35.5
11757	44.5	15	17	69	40.5	17	17	22.5	15
11758	57.5	18.5	17	109	40	52	10	22.5	19
11759	39.5	16	61	87	43	19	12	18	23
11760	12.5	10	6	11.5	26	12	8	10	6
11761	31	12	17	52	72	17	23	57	16
11762	30	19	17	99	32	21	43	37	49
11763	26	19	14	54	26	24	12	51.5	19
11764	36	34	23	98	27	22	18	42.5	58
11765	21	17	15	78	25	20	11	22	18
11766	23.5	18	16	58	25.5	16	11	22	75
11767	24	19	15	74	34	17.5	11	21	36.5
11768	25	16	12.5	46	23	16	9	21	45
11769	26	14	14	54	21	15	10	21	26
11770	32.5	17	16	54	34	25.5	26	46	32
11771	22	13	12	55.5	30	18.5	10	22.5	10
11772	30	17	17	78	33	23	16	61	20
11773	41.5	16	18	61.5	29	30.5	10	33	23
11774	37	20.5	23	99	40	26	14	34	23
11775	15	10	9	73	21	12	8	14	11
11776	28	14	13	38	26.5	18	14	27	18
11777	19	12	12	57	37.5	17.5	9.5	32	19
11778	34	16	16	87	41	19	17	36	52
11779	22	12	12	26	34.5	16	19	15	13
11780	38	55	30	96	42	32	32	60	69
11781	37.5	66	36	92	39	42	29	66	77
11782	40	65.5	33	93	41	35	29	74	62
11783	29.5	42	23	81	31.5	30	21	57	43
11784	58	54	32.5	116	36.5	34	29	64	68
11785	33	39	28	91	32	29.5	20	43	43
11786	39	60	40	97	35	41	29	60	60
11787	27	30	21	81	24	23	18	34	51
11788	33	42	25	78.5	33	29.5	26	46	55
11789	36	40	24	96	32	29.5	29	49	55
11790	29	42.5	23	92	30	33	20	43	49
11791	75.5	38	19	82	35.5	66	16.5	39	37
11792	58	54	32	115.5	33.5	35	24	58	64
11793	47	80	40	145	43	45	37	83	137
11794	53	37	25.5	90	29	29.5	19	41	51
11795	48	68	35	117	36	39	32	64	74

11796	56.5	69	35	109.5	40.5	55	29	62	76
11797	38	45	31	105	35	36	24	49.5	55
11798	27	33	19	72	28	24	18	32.5	39
11799	36.5	59	33	129	36	38	25	59	53
11800	34.5	54	28	101	32	36	24	53.5	56
11801	39	33	23	87	32	25	30	37	42
11802	54	78	39	107	40	46.5	32.5	76	92
11803	53	75.5	38	105	38	44.5	40	79.5	101.5
11804	46.5	62	33	112	36	37	30	61	55
11805	36	65	33	108	35.5	37	29	63	57
11806	40.5	41	23	79	29	30	20	43	45
11807	40	75	35	146	38	40.5	31.5	67.5	72.5
11808	34	37	24	86.5	25	27	22	40	42
11809	36	59	31	95	32	36	28	54	70
11810	38.5	35	24	95	29	30	21	43	50
11811	43	41.5	29.5	100.5	33	32	21	57	43
11812	56.5	64.5	40	127.5	32	41	30	62	85
11813	42	53	30	102	34.5	34.5	26	54	64
11814	36	47	28	96	34	32	24	41	43
11815	31.5	45	23	76	30	28	19.5	43	46
11816	42	42	25.5	71	26	32	24	40.5	39
11817	44.5	48.5	31	83.5	32	41	22	45	49
11818	50	57	32	101.5	37	46	27	60	64
11819	35.5	45.5	29	103	31	30	25	45	49
11820	40.5	53	29	85	32	41	27	54	56
11821	53.5	77	38	121	39	46	33	71	87
11822	41	46.5	26	103	28	32	23	50	50
11823	29	32	22	79	32	23	17	34	46
11824	38	49	24	102	29.5	28.5	23	43.5	48.5
11825	74	79	50	125	51	54	41	86	79
11826	45	68.5	76	88	83.5	43	84	74	85
11827	70	37	27	91.5	33.5	27	20	40	38
11828	36	50	29	88.5	33	32.5	22.5	47	53
11829	44.5	31	27	159.5	63.5	23	77	202	20
11830	10.5	9	5	10	9	13	7	10	6
11831	23	12	14	51.5	31	13	15	18	13
11832	31.5	21	14	65	31	23	14	18	20
11833	42	23	16	85	30	34.5	17	39	35
11834	69.5	20	11	77.5	98.5	26	14	40	31
11835	247	63	58	200	83	63	62.5	318	43
11836	38.5	21	31	88	32	26	20	19	19
11837	41	24	16	72	45	30	15	24	20
11838	13	13	7	12	12	18	7.5	9	9
11839	45	24	41	175	45	34	58	58	20
11840	22	21	17	58	28	23.5	12.5	22	21
11841	20.5	17	11	63	21.5	21	9	12	21
11842	53	23	17	63	43.5	34	16	27	27

11843	25	22	16	52	27	27	12	18	54.5
11844	43	21	31	132.5	38	28	87	38.5	61
11845	24.5	20	13	76	25	21	12	16	27
11846	27.5	17	19	69	27	19	13	18	32
11847	44.5	24	25	67	29	54	17	38.5	20
11848	24	20	17	42	24	25	14	24	34.5
11849	603	245	265	499.5	338	348.5	311	474	297
11850	16	14	9	21	27	19.5	13	14	9
11851	18	18	11	54	24.5	19	12	17.5	10
11852	49.5	58	31	118	50	32	26	56	73
11853	34	43	27	81	31	34	21	40	82
11854	51	79.5	41	104	46	49	37	81	87
11855	26	17	18	97	26	28	10	20.5	19
11856	41	74	43	102	37.5	45	32	71	69
11857	46	69	39	120	44	49	34.5	67	95
11858	42	78	38	115.5	43.5	49.5	34	69	95
11859	37	48	28	100	29	34	22	48	48
11860	32	49	29	96	34	36	24	42	75
11861	54	85	46.5	124	51.5	53	37.5	78	92
11862	40	56.5	32	106	38	38	27	59	72.5
11863	55	88	51	135.5	44	62	42	83	85
11864	34	41	23	87	34	32.5	20	39	54
11865	28	14	17	118	26.5	19	14	24	24
11866	37	21	27	127	37	27	14	48	46
11867	22	15	13	62	23.5	21.5	8	14	15
11868	23	16	19	98	22.5	19	10	23	32
11869	24	18.5	9	27	38	27	9	18	14
11870	26	18	19	83	28	23	13	28	38
11871	18	13	10	60.5	26	18	8	17	10
11872	21	17	12	39	27.5	21	17	40	14
11873	68	22	17	44	32.5	25	20	25	20
11874	140	19	26	84	35.5	24	18	38	58
11875	34.5	20.5	21	90	39	24.5	16.5	39.5	127
11876	207.5	48	42	117	53.5	62.5	30	62	204
11877	37	22	22	94	31	25	16	34.5	80.5
11878	48	23	27	130	30	25	17	48	26
11879	29.5	14	16	90.5	26.5	20	13	24	90
11880	115	14	14	35	35	20	20	29	17
11881	45	15	16	47.5	128	18.5	12	21	49.5
11882	29	14	18	106	23.5	20	14	24	49
11883	23	23	19	68	25	22	12	25	155.5
11884	26	17	15.5	70	20	19	12	22	17
11885	19.5	17.5	13	38.5	41	20	9	13	30
11886	39	55.5	33	90	44	44	28	44.5	57
11887	44	54	33	101	37.5	47	27	47	51
11888	54	51	36.5	107	38.5	44	23	46	56
11889	58	91	49	102	43	62	39	76	79

11890	45.5	59	33	97	33	46	25	55	72
11891	65.5	97	43	128	58	50	33	77	77
11892	51	84	46	133	53.5	65.5	37.5	67	85
11893	43.5	47	33	96	32	45	23	46	53
11894	46	62	36	85	43	48	25	55	57
11895	36	42	31	87.5	35	42	19	40.5	53
11896	46.5	58	41.5	89.5	36	50	26	47	74
11897	36.5	40	29	99	79	40	32	36	42
11898	50	79	39	109.5	39	52	32	64.5	82.5
11899	39.5	47.5	33	91	40	38	24	40	46
11900	47	70	41	105.5	40	56	30	66	79
11901	60	75	44	97	47	57	30	69	74
11902	57	71	45	120	44	52	40	70	83
11903	38	49.5	32	101	35.5	43	24	45	61.5
11904	120	72	35	97	43	132	25	47	72
11905	47	71	46	122.5	36.5	49.5	27.5	57.5	82
11906	43	63	39	106	68	50	33	55	73
11907	54	60	43	114	40.5	46	29	55	82
11908	98.5	53	34	102	49.5	43.5	27	46	70
11909	53	51	34	100	34.5	44	24	49	91.5
11910	54	54	36	126	38	43	28	55	69
11911	168	67	38	100.5	37	114.5	25	49.5	75
11912	37	55	31	89	32	41	45	46	46
11913	54	56	36	102	180	43	22	57	49.5
11914	47	67	39	120	37	49.5	29	60	67
11915	52.5	77	41	119	47	52	32	65	80
11916	43.5	60	41	115	35	47	30.5	60	62
11917	57.5	57	51	97	52	44	28	53	77
11918	70	74.5	44	125	50	54	33	95	96
11919	53.5	79	42	118	50.5	50	33	70	92
11920	45	56	34	94.5	43	40.5	27	52	62.5
11921	63	64.5	40	100	37	55	35	58.5	78.5
11922	33	20	22	68	28	30	13	18	20
11923	50	56	33	99	42	42	24	51.5	95
11924	37	32	24	105	37	30	17	29	35
11925	43	49	31	84.5	37	38	21.5	45	68
11926	25	22	18	39	26	23	12	19	29
11927	31	28	23	57	25.5	33	16	47	62
11928	45	22.5	18	56.5	27	25	12	18	47
11929	23	19	16	32	28	25	9	17	45
11930	26	22	21	40	26	25	10	18	47
11931	23	16	21	126	21	21	8	23	12
11932	31.5	18	24.5	106.5	55	28	12	21	31
11933	44.5	21	22	68	37	28	14	33	28
11934	31	21.5	23	52	33	25	15	24	31
11935	31	22	19.5	39	27	30	11	19	19
11936	25	23	16	51	21.5	24	11	17	44

11937	68	43	48	96	59	48	49	75.5	56
11938	41	45	40	83.5	32	41	21	42.5	50
11939	41	46	33.5	88	31	39	22	43	48
11940	54	70	42	116	40	50.5	32	66.5	80
11941	39	52	31	89	34.5	40	25	44	49.5
11942	31	36	29	79	27.5	33	20	35	39
11943	54.5	32	19	69	23	52	13	23	24
11944	44	66.5	39	115	43.5	46.5	33	65	64
11945	61	85	47	122	129	57	36	81.5	85
11946	73	46	31	93	36	38	25	48	51
11947	43	51	33	95	38	41	27	43	50
11948	40	44	29	94	32	41	20	42	57
11949	36.5	50	32	88	33	36	25	47.5	73
11950	32	43	29	72.5	34.5	35	24	42	56
11951	37.5	47	31.5	100	36.5	36	39	46	117
11952	37	38	26.5	85	31	30	19	35	42
11953	64	54	32	83	30	66.5	19	38	57
11954	49	57	32	90	32	44	25	55	61
11955	61	56	33.5	104	32	46	25	53.5	54
11956	27	39	27	71	31	28.5	29	42	66
11957	284	60	36	82	31	52	32	80	59
11958	37	26	25	80	30	28	19	48	95
11959	16	11	9	27	19.5	17.5	10	13	14.5
11960	36.5	42	28	83	33	34	20	41	63
11961	44	79	42	104	41	42	28	70	98
11962	37	73	35	114	39	43.5	30	69	76
11963	34	18	26	43	38.5	22	37.5	41	26
11964	40	58	31.5	101	35	38	27	55	60
11965	39	50	37	93.5	36	38	26	47.5	50
11966	24	28	20	58	32	23	16	33	45
11967	36	40	26	75	27	28	19	44	43
11968	37	56	34	102	54.5	39	24	49	62
11969	32.5	39	27	85	28	32	19	69	38
11970	33.5	47	30	93	33.5	36	23	46	62
11971	23	16	19	132	22	20	11	19	14
11972	44	63	36	114	43	40	26.5	54	60
11973	165	62	41	99	38	58	27	62	88
11974	45	83	37	111	36	48	43.5	67	102
11975	34	30	24	72	26	26	17	38	30.5
11976	21	14	12	30	30	21	12	114	19
11977	36	18	15	36	30	30.5	12	14	15
11978	42	17	17	68	534.5	24	16	197	26
11979	37.5	22	19	36	30	32	20	25.5	19
11980	20	14	9	22	17	20	8	12	10
11981	34	24	15	50	19	34	9.5	13	15
11982	19	24.5	13	42	17	28	13	13	15
11983	30	22	19	71	27	29	12	24	18

11984	25	22	16	59	25	28	11	13	16
11985	28.5	39	21	62	22	38	20	29	39
11986	19	23	13	38.5	20.5	30	10	15.5	14
11987	34.5	25	21	70	26.5	32	15	26	19
11988	39.5	23	18	62	25	32	17	66	18
11989	44	23	18	40	41	27	11	115.5	19
11990	21	22	10	19	32	27	9	9	13
11991	20	21	16	32	24	24	11	12	16
11992	34	23	17	48	49	27	19	22	61
11993	85	26.5	26	110.5	26	33	16	66	58
11994	25	26	29	116	26	31	12	47	23
11995	92.5	64.5	27	102	33	57	32	24	47
11996	70.5	25	21	89.5	31	34	14	29	29
11997	17	20	10	16	15	24.5	9	10	12
11998	19	19	12	46.5	17	25	11	14	14
11999	25	24	17.5	68	19	24	12	18	17
12000	21	24	17	71	20	26	10	23	16
12001	23	31	21	81.5	21	25	10	24	20
12002	17	20	13	16	17	24	7	14	12
12003	27.5	26	20	75.5	20	30	14	26	19.5
12004	22.5	23	13	69	16.5	24	8	14	20
12005	23	25	35	106.5	20.5	28	12	21	20
12006	18.5	20	13	46	18	23	10	12.5	12
12007	18	23	19	50	17	22	9	16	13
12008	17.5	20	14	81.5	18.5	23	9	16	13
12009	22	20	18	93	20	26	10	18	23.5
12010	19	20	17	92.5	19	24	10	19	16
12011	19	18	15	74	17	24	10	18	13
12012	24	20	18	137	17	24	11	35	14
12013	21	17	18	48	18	25	10	14	13
12014	50	21.5	25	112.5	38.5	27.5	29	36	27
12015	21.5	18.5	14	49	19	24	10	82	15
12016	29.5	25	19.5	78	22	27	14.5	26	22
12017	32.5	21	21	128	29	27.5	18	23.5	20
12018	34	20	19	75	34.5	28	15.5	104	25.5
12019	42.5	19.5	50.5	66.5	29	25	62	24	19
12020	28	20	17	44	26	26	15	23	21
12021	149	57	31.5	98	28.5	47	21	153	70
12022	31.5	21	24	66	31	28	15	69	28
12023	49	32	27	69	24	35	18	44.5	81
12024	73	23	22	70	47.5	36	33	51	30
12025	25	21	16	52	19	23.5	10	15	21
12026	27	20	18	84	25	24	11	27	18
12027	31	19	22	122	26.5	22	15	25	19
12028	31	19	20	108	33.5	25	23	28	31
12029	26	21.5	18.5	156	19	28	12	46	13
12030	37.5	34	26	94	28	38	17	38	37

12031	32	18	19	39	23	25	12	21	15
12032	25	21	21	80.5	27	27	16	24	25
12033	27	17	16.5	94	27	24	15	23	52.5
12034	28	18	27	61.5	29	21	15	22	108.5
12035	32	22	19	61	104	24	26	32	16
12036	58	23	14	35	22	67	11	15	20
12037	22	16	16	39.5	52	20.5	9	15	17
12038	28	27	23	68	29	28	16	29	126
12039	48	59	35	83	30	37.5	27.5	66	74
12040	28	17	19	64	24	25.5	11	20	14
12041	50.5	18.5	18	84	31.5	24	17.5	26	52
12042	42	57	36	95	42	47	24	57	56
12043	51	53	38	109	43	46	33	48	58
12044	59	83	43	109	42.5	61.5	37	63.5	82
12045	51	71	41	110	43.5	54.5	45	53	65.5
12046	69	81	55	127.5	49	70	35	54	92
12047	45	59	39	112	46	40.5	25	53	57
12048	49	59	45	88	40	43	29	60	60
12049	56.5	75	44	119	39.5	57	30	59	74
12050	64	75.5	46	128.5	46	63	35	63	111.5
12051	64	84	46	127	49	70	36	64	88
12052	64.5	82	44	139	49	66.5	30	55	70.5
12053	88	81.5	43.5	116	40	51	36.5	78.5	75
12054	49.5	83	45	136.5	45	50	35	82.5	95
12055	62.5	40	31	106	26	39	42	34.5	51
12056	50.5	63	43	120.5	38	53.5	33	56	82
12057	63.5	75	46	118	48	56	34	68	66
12058	59	58	34	96	34.5	53.5	25	48	47
12059	72	99	55	133	60	79	45	77.5	104
12060	85	100	61	194	63	84	45	90.5	112.5
12061	47.5	40	27	120	28	32	18	40	45.5
12062	59	74	45	127	43.5	56	74	70	74
12063	61	77	53	130.5	41	62	33	70	86
12064	48.5	55	40.5	89	32	50.5	22	44	49
12065	68.5	74	48	129	54.5	66.5	33	69	85.5
12066	39	59	38	107.5	35	39	22	52	60
12067	53	45.5	34	95	34.5	40	21	43	48
12068	49	62.5	34	89	34	49	24	49.5	60.5
12069	54.5	63	36	134	45	53	27	51	78
12070	43	42	26	89	27	32	22	38.5	79
12071	27	20	21	81.5	28	24	11	21	30.5
12072	29	23	21	67.5	23.5	28	11	17	23
12073	29	25	19	81	26	32	12	19	68
12074	54	58	41	87.5	36	48	26	46	64
12075	26	24	16	45	22	30	14	16	15
12076	54	29	23	62	40	46	25	60.5	30
12077	48	17	26	101	146	24	14	59	20

12078	86	18	18	149	29	24	16	21	17
12079	62.5	25	18	60	31	40	15	30	18
12080	66	26	20	46	28	28	12	17.5	39
12081	51.5	24	15.5	37.5	32	38	19.5	24	26
12082	210	29	29	51	43	45.5	24	135	33
12083	48	34	27	55.5	65	43.5	21	42.5	31
12084	58.5	30	24	121	29	41	18	31	40
12085	16	10	11	50	16	14	6	13	7
12086	16	14	9	32	18	18	7	12	8
12087	34	21	18	65	20	28	25	18	15
12088	27	21	26	66.5	23.5	29	13	20	23
12089	36	23	18	104	29	31	13	18	37
12090	93	28	31.5	52.5	41	39	19	34	60
12091	48	28	25	38	129	41	27	45	24
12092	68	32	35	117.5	34	46	20	99	64.5
12093	62	19	23	93	27.5	39	22	36	26
12094	44	24	17	40	31	23	13.5	26	15
12095	38	21.5	24	93	32	27	14	20	23
12096	50	21	18	44	28	27	33.5	100	19
12097	206	40	43	134	56	46	45	198	34
12098	59	38.5	18	126.5	29	57	65	32	41
12099	84	40.5	33	174	42	51	108	47.5	40
12100	220	42	24	87	38	122	22	78	68
12101	29	14	15	87	28	17.5	11	15	13.5
12102	18.5	14	10	25	15.5	17	7	18	11
12103	25	17	13	32	67.5	22	9	25	12
12104	35.5	21	15	35	25	33.5	10	74	14
12105	48	21	15	35	25	35	13	57	15
12106	44.5	21	18.5	43.5	23	33	11	21	17
12107	44.5	23	18	48.5	23	33	51	17	21.5
12108	67	28	21	176.5	36	41	20	156	28.5
12109	51	13	16.5	31	32.5	17	13	36	14
12110	34.5	18	18	55	69.5	23	26	74	21
12111	54.5	20	110.5	53	53	42	18	79.5	17
12112	354.5	67	36	42	44	330	29	83	121
12113	47	23	23	67.5	38	30	22	44	24
12114	481.5	26	23	106	29	38	16	33	79
12115	62.5	25	20	46	29.5	35	14	33	22
12116	45	24	21	103	25	34	16	26	27
12117	44.5	30	26	90	37	43	27	24	31
12118	47.5	38	28	132	34	49.5	21	28	121
12119	38	8	14	56	19	8	12	15	14
12120	16.5	11	11	45	17	12.5	8	13	12
12121	24	12	15	70.5	20	16.5	10	13	73
12122	29.5	16	24	158	24	20	15	25	11
12123	42	22	18	64	27	28	20	39	20
12124	27	17	16	65	22.5	24	15	17	30

12125	40	24	23	75	51.5	31	38	23	22
12126	101	27	23	90	29	65	13	30	28
12127	81.5	14	23	155.5	52.5	19	21	56	59
12128	21	8	15	72	21	11	9	16	17.5
12129	19	13	10	47	17.5	16	11	13	11
12130	27	13	15	31	22	19	12	19	24
12131	56.5	16	16	33	32	21	11	35	16
12132	49.5	26	27	121	41.5	34	19	60.5	46.5
12133	42	23	19	75	33	34	17	24	23
12134	145	33	30.5	95	40.5	60	20	51	310
12135	45.5	22	31	146	53	24	24	80	50
12136	15	11	9	12	14	13	7	11	7
12137	19	11.5	12	17	19	12	11	11	9
12138	20	13	12	24	31.5	16	9	15	13
12139	37	16	20	81.5	31	18	13	23	21
12140	56.5	84.5	46	112	85	56	34	67	77
12141	50	44.5	31	94	31	49	22	42	60.5
12142	51.5	72	43.5	107.5	41	42.5	33	79	73
12143	34	41	33	76	25.5	31	20	63	54
12144	35	35	29	65	24	25	17	31.5	36
12145	44	74	45	96.5	39.5	48.5	35	73.5	81.5
12146	23	21	18	75	35	21	13	26.5	24
12147	47.5	57.5	32	96	33.5	43	22	48.5	80
12148	54	82	43	126	46.5	53	35	68	108
12149	33.5	39	32	77	31	26	23	42.5	53.5
12150	32	24	18.5	90	30.5	19	16	28	20
12151	39	55	33	96.5	30	37	22	48	59
12152	46.5	56	33	112	31.5	41	24	53	58
12153	39	44	30	100	29	34.5	22	46	40
12154	23	20	16	66	23.5	23	12	18.5	28
12155	55	81	50	113	45	61	41	72	98
12156	33	52	29	80	31.5	30.5	23	47	51
12157	45	70	41	91	35	44	29.5	64	73
12158	19	13.5	12	41	14.5	15	8	13	12
12159	37	56	39	79	31	43.5	28	49.5	65
12160	42.5	61	34.5	99	36	42	26	50	84
12161	31	27	23	87	27	27	15	27	42
12162	37.5	42	27	79	35.5	40	20.5	34	53.5
12163	38	58	37	89	28	35.5	27	54	58
12164	43	57.5	33	102	36.5	35	28	56.5	65
12165	27	23	18	80.5	21	18	12	25	89
12166	53	72	43	111	59	50	35	70	84
12167	51	58	36	99	37.5	45	29	53	82
12168	52.5	53	33	101	34	40.5	24	53	52
12169	102.5	57	34	177	110	81	27	56	106
12170	34	29	23	74	25	29	18	32	36
12171	47	44	33	88	31	36.5	19	72	55

12172	39	51	37	101.5	38	36	25	68	60.5
12173	48	83	44	106	43.5	48	33	66	92.5
12174	49	47	30.5	128.5	25	49	12	36	47
12175	19.5	8	13	66	18	11	10	57	10
12176	46	48	44	91	32	36	26	88	63.5
12177	21.5	16	14	29	15	16	9	11	38
12178	57	71	43	109.5	35	44	32	66.5	69
12179	40.5	37	23	93	26	37	18	38	60
12180	69	42	26	85	29	59	21	38	50
12181	24	20	20	37	23	27	14	19	15
12182	22.5	16	14	75	24.5	17.5	10	21	22
12183	37	64	33	124	31	35	27	56	67
12184	23	13	19	63	15	14	11	15	11
12185	21.5	14	12	52	30.5	17	24.5	14	10
12186	23	14	9	35	19	18	8	14	18
12187	24	19	14	33	53	27	10	16	31
12188	23	17	13	63	24	25	11	18	16
12189	32	17	15	74	22	28	12	17.5	25
12190	37	13.5	32.5	48	43	18	54.5	27	18
12191	17	11	9	44	15.5	14	9	10	8
12192	25	12	13	29.5	43	16	12	77	18
12193	163	20	19	48	27.5	26	20	53	17
12194	26	14	13	61	20.5	21	9.5	15	10
12195	28	17	17	65	26	26	34.5	19	18
12196	32	20	16	41	27.5	33	11	16	21
12197	94	24	25.5	85	47	37	27	34	41
12198	24	19	14	50.5	26	30	10	42	13
12199	17.5	17	11	43.5	15	18	7	11	11
12200	22.5	20	14	39	21	24	11	26	12
12201	24	22	16	32	41	23	19	14.5	16
12202	22	22.5	21	54	21	26	12	17	28
12203	38	20	17	44	18.5	27	11	20	16
12204	59	20.5	15	55	40	24	10	25	15
12205	77	36	34.5	58	194	28	97	97	19
12206	22	15	13	28	17	20	10	13.5	13
12207	16.5	17	11	40	21.5	20	8	12	19
12208	17	15	13	37	19	18	9	11.5	16.5
12209	22	15	12	42	17	19	10	12	13
12210	25.5	18	20.5	40.5	31	20	11	16	16
12211	22	18	13	42	19	21	9	17	18
12212	22	18	16	43	58	21	11	15	18
12213	23	15.5	12	33	22.5	26	14	13	17
12214	28	17	16	34	21	22.5	11	109	14
12215	17	15.5	12	34	20	15	9	14	9
12216	31	17	17	49	26	22	13	17	16
12217	20.5	16	10	35	14.5	19	8	10	15
12218	1200	222	301	430	236.5	277	329.5	744	231

12219	35.5	18	24	129	25	23.5	17	26	28
12220	37.5	19	17	59	121.5	19	15	16	34
12221	29	19	20	99	24.5	21.5	19	22	17
12222	16	16.5	11	42	16.5	18	7	12	12
12223	30	18	23.5	107	21	22	10	26.5	43
12224	45	13	12	41	26	16	10	51	44
12225	26	18.5	14	48	20	23.5	10	55	18
12226	24	17	28	81	25	21	31	22	56
12227	36	19	31	135.5	27	23	13.5	49	76
12228	50	25	38	121	32	27	20	71	109
12229	24	19	16	74	22.5	23	13	23	16
12230	115	17	13	54.5	17	18	12	12	12
12231	24	15.5	15	41	23	14	10	14	28
12232	29	18	20	76	29	20	27	47.5	57
12233	13	15	12	39	14	14	7	8	7
12234	20	15	9	40	18	15	9	12	109
12235	34.5	15.5	12	40	15	16	16	21	10
12236	25.5	16	12	51	16	17.5	8	13	15
12237	14	16	11	48	16.5	16.5	8	14	11
12238	14	14	12.5	66	14.5	17	7	13	21
12239	29	16.5	17	97	21	16	61	27	79.5
12240	45.5	21	22	69.5	23	30	15	24	22
12241	25	15	13	31	19	22	10.5	15	28
12242	39	28	16	29	257	30	17	25	54
12243	39	22.5	13	19	296	19	16.5	13.5	52
12244	77	91	80	129	33	52.5	18	383	47
12245	26	15	16	47	18.5	21	15	96	17
12246	44	22	17	61	21	29	11.5	18	23
12247	19	13	65	44	18	18.5	9	20	14
12248	96.5	23	32	76.5	37	39	23	76	38
12249	18	16	12	32	21	16	13	37	13
12250	22	16	15	52	24	16	12	17	16
12251	29	15	16.5	59.5	18	21	11	12	13
12252	16	15.5	24	57	43	16	29	22	10
12253	68.5	81	51	103	61.5	75	36	67.5	86
12254	31	25	25	64	30	27	15	22	34
12255	69	87	58	136.5	57.5	65	47	72	102
12256	48	71	44	93	52	52	41	60	72
12257	37	37	26.5	90	59	40	18	32.5	54
12258	59	70	44	105	59	60	30	59	81
12259	70	85	42	101	47	65	37	68	77
12260	44.5	79	43	93	55	53	30.5	70	80
12261	53	88	41	139	51	55	29.5	58.5	77
12262	56	80	48.5	103	52.5	55.5	39	77.5	91
12263	40	55	34	100	57	42	26	45.5	65
12264	50.5	39	29	106	39	43	21	41	95
12265	58.5	65	42	89.5	55.5	52.5	32	62	63

12266	55	82.5	53	136.5	55.5	60	47	73.5	92
12267	43	63	38.5	92	58	46	31	55	65
12268	36.5	50	38	85	40	44.5	22	36	51
12269	60.5	42	37	155	59	45	30	46	45
12270	68	68	59	104	52	52	31	63	64
12271	68	80.5	45	111.5	59	61	37	72	122
12272	46	58	39	89.5	59	45	31	59	63.5
12273	43	45	30	72	85	40	23	42	37
12274	50.5	83	55	121	77	58.5	36	69.5	103
12275	49	88	46	101	54	64	38	76	82
12276	55	79.5	56	128	57	58	33	67	81
12277	55.5	96.5	50	127	74	60	42	83	100
12278	46.5	67	39	102	47	49	31	61.5	61.5
12279	38.5	53	32	91	40	38	27	52.5	68
12280	52	88	50.5	93.5	58	64.5	40	77	124
12281	44	56	33.5	94	46.5	41.5	27	49	57
12282	47	62	42	105	49.5	45	33	61	92
12283	48	60	30	82.5	47	43	26	47.5	62
12284	58	83	46	104	54.5	59	35	64	118
12285	33	19	18	73	29	32	11	18	24
12286	19	15	12	48	22	20	8.5	13	10
12287	26	17	18	79	30	20.5	10	19	12
12288	14	17	8	12	17	22	7	11	10
12289	27	17	16	55	30	23	11	20	15
12290	14.5	13	9	12	23.5	19	8	11	10
12291	23	15	16	83	24.5	18	8	15	11
12292	16	12	10	41.5	22	15	9	13	11
12293	12	12.5	7	12	20	13	8	11	9
12294	21	19	15	72	28	25.5	10	15	14
12295	22.5	16.5	16	50	36	21	10	17	16
12296	16	15	8	13	17.5	17	8	9	10
12297	17	15	9	12	18.5	20	9	11	9
12298	21	15	15	101	23.5	19	11	17	11
12299	18	17	13	54	27	22.5	10	15	11
12300	15	14	7	13	16	15	9	12	8
12301	20	16	17	50	28	22	11	12	15
12302	28	16	14	31	32	19	11	14	17
12303	18	14	12	38	27.5	20	8	12	12
12304	26	17	17	63	31	19	9	19	12
12305	27.5	15.5	13.5	61	34	22	10	15	40
12306	22	14	19	75	52.5	20	11	20	22.5
12307	34	25	36	51.5	177	33	21	52	24
12308	35	21.5	18	35	48	30	15	26.5	22
12309	38	18	22	108	45	25	14	32	118
12310	25	18	17	68	32.5	23	15.5	21	32
12311	89	25	32	152.5	657	31	40	97.5	69
12312	27	19	16	32	41	24	16	101	27

12313	22	14	23	27	35	18	13	14	69
12314	29	18	21	38	38	21	31	32	45
12315	52	19	20	50	41	31.5	18	27	19
12316	37.5	25	27	79	50	24	39	70	36
12317	26	17	17	27.5	40.5	22	14	16	17
12318	27	18	21	51	39	24.5	19.5	23	22
12319	18	15	15	50.5	27.5	20	8	13	19
12320	38	16	15	55	42	27	11	25	32
12321	47.5	20	18	45	81	25	18	76.5	13
12322	19	13	13	43	33	15	9	14	10
12323	19	15	17	50	32	19.5	10	20.5	16
12324	22	15	30	33	34	19	27.5	32	20
12325	99	18	20	42	37.5	29	20.5	111	64
12326	22	15	14	39	30	17	9	18	11.5
12327	27	17	25	79	381	27.5	16	23	17
12328	178.5	16	22	99	37.5	25	12.5	16	19
12329	18	15	14	108	30	18	15	14	13
12330	21	15	18	59	34	18	12	13	15
12331	28	17	20	140	42	24	27.5	19	54
12332	19	13	15	61.5	27	17	8	19	11
12333	57.5	22	29	66	65.5	27	23	43	25
12334	19	68.5	12	41	26.5	15	13	12	11
12335	41	19	23	107	43.5	23	15	32	53
12336	19	13	15	66	29.5	17	8	17	19
12337	36	17	21	105.5	30	21	32	27	15
12338	40.5	22	17	36	124	33	11	18	26
12339	24	16	17	96.5	69.5	24	26	23	19
12340	22	12	10.5	28	160	19	15	28	12
12341	24.5	15	17.5	23	34	21	10	27	19
12342	18	14	10	19	24.5	16	7	11	11
12343	31	16	12	29	28	23.5	14	28	12
12344	28	18	14	24	35.5	23	14.5	52	15
12345	24	21	16	72.5	40.5	18	13.5	85	33
12346	62	29	32	114	42	29	27	46.5	117
12347	40	28	28	63	63	35	19	30	24
12348	19	16	17	84	22.5	21.5	12	36	12
12349	119.5	51	31	105	40	45	24.5	46	84
12350	29	40	27	60	33	31	24	39	45
12351	39	61	38	77	44	41	33	55	222.5
12352	31	40	24	72	39	30	18	41	50
12353	29	38	23	69	53	28	17	34	40
12354	45	45	27	88.5	87	37.5	31	49	86
12355	47	60	40	161	46.5	42	31	55.5	79
12356	32.5	39	26	67.5	35	29	19	36.5	43
12357	103	59	33	77	43	51	29	51	73.5
12358	33	39	23	83	35	28	16	42	40
12359	37	59	34	78	40	44	28.5	52	56

12360	39	50	25	84	36	38	22	46.5	49
12361	33	43	25	79	30.5	28	29	38	61
12362	41	46	29	84	40.5	34	25	49	48
12363	33	47	28	65	33	34	24.5	46	46
12364	30	47	29	103	40	31.5	22	49.5	59
12365	49	75.5	38	95	50.5	48	31	65	72
12366	59	67	34	103.5	39	44	29	62	67
12367	33.5	39	28	79	35	33	20	37	41
12368	41	49	28	113	38	38.5	20	46	57
12369	28.5	38	22	64.5	33	29	17	35	37
12370	51	33	36	81	30	46	16	32	50
12371	44	54	35	102	45.5	40	25	72.5	50
12372	33	40	27	78.5	38.5	31	26	40	46
12373	33	39	34	71	35.5	32	18	44	53
12374	34	20	20	56	30.5	24	13	25	44
12375	32	40	26	61.5	31	27.5	20	41	55.5
12376	34	55	27.5	68	32	33.5	21	48	60
12377	54	76	41	104.5	49	57.5	38	65	106
12378	34	26.5	20	74	30	27	16	26.5	29
12379	59	67	36	79	39.5	51.5	26.5	59	74
12380	45	38	30	78	38	37.5	26	36	42
12381	68.5	60	29	65	37	54	38	45	67
12382	26	27.5	17	68	27	22	15	29	31
12383	34	36	24	70	34	31	18	34	51
12384	24	28	19	59	27	24	16	28	29
12385	34	46	29	93	36	31	21	43	53
12386	37	43	28	80	33.5	33	133.5	44	47
12387	39	45	34	97	44.5	32	25	41	43
12388	50.5	69	38	172.5	42	43.5	34	66	69.5
12389	27	33	22	61.5	30	25	16	28.5	67
12390	40	52	36	91	60.5	35	23	48	55.5
12391	33	43	26	69.5	33	33	21	40	41.5
12392	33	36	31	61	35	23	19	36	39
12393	36	43	31	76	35	34	22.5	45	53
12394	44	52	32	72	39	40	27	53	58
12395	67	55	32	99	42.5	37	28	53.5	103.5
12396	43	41	26	57	34.5	33	20.5	41	49
12397	55.5	66	40	92	44	47	35	61	74
12398	51.5	45.5	33	74	40.5	36	24	44	90
12399	165	56	33	66.5	35	45	27	54	54
12400	64.5	86.5	44	120.5	62	54	27	63	77
12401	68	83	50.5	140	61	61	35	73.5	110
12402	49	54	32	105.5	55	48	23	35	61
12403	55	53	37	106	62	53.5	25	40	74
12404	62	41	28	110.5	56	44	22	40	56
12405	53	41	35	122	51	48	19	56	60
12406	71.5	42	37	108.5	52	51	49	30	62

12407	74	58	39	129.5	59	57	26	42	94
12408	46	29.5	16	53	42	37	15	20	27.5
12409	79	32	18	68.5	48	40	12.5	17	34
12410	41	27.5	16	62	45	35	14	91	42
12411	33	28	18	64	43	37	10	23.5	26
12412	81	30	21	54	45	36	16	40	31
12413	53.5	39	54	119.5	50	41	21	29	77
12414	50	32.5	19	94.5	56.5	39.5	13	16	35
12415	34.5	25	17	70	41	35	12	14	24.5
12416	68.5	31	20	92	51.5	44	13	16	33
12417	46	26	17	72	52	32	11	15	23
12418	57	50.5	37	118.5	51.5	49	23	38	68
12419	56.5	53	39	100.5	52	47	25	41.5	57
12420	100	54	35	142	49	50.5	24	45	56
12421	56	42	30	119.5	51.5	44	22	42	56
12422	61	83	46	117	53.5	63	32	64	81
12423	48	52	28	96	44	46.5	19	35	58
12424	49	59	34	107	50.5	45.5	21	38	62
12425	58	82	39	104	56.5	58	33	59	97
12426	66	75	46	113	61	57	33	62	90
12427	43	40	25	101.5	56	40	15	31	57
12428	46	41	26	92	45	43.5	15	30.5	51
12429	51	56	32	152	52	48	25	43	69
12430	48	39	27	134	40.5	43	16	35	44.5
12431	61	58	35	103	53	50.5	27	45	55.5
12432	59	68	35	120.5	55	54	26	50.5	64
12433	30	17	21	83.5	31	22	26	19.5	19
12434	51	59	34	91	30	50	22	56	60.5
12435	63	78	41.5	126	59	59	37	68.5	81
12436	41.5	19	20.5	70.5	33.5	28.5	9	20	33
12437	28	16	19	106	23	22	11	23	59
12438	49	51	27	93.5	46	44	20	39.5	60
12439	20	15	9	49	16	20	7	12	10
12440	17	15	6	25	13	22	7	10	11
12441	51	53.5	29	123	50.5	47	21.5	44.5	62
12442	14	14	5	17	9	18	6	8.5	8
12443	65	48	25	118	47.5	43	18	35	56.5
12444	56	45	26	97	43	41	18	34	58
12445	52	48	25	101	38	52	17	28.5	49
12446	75.5	64	35	106	54	51	25	45	64
12447	57	53	32	95	47.5	49	22	34	57.5
12448	43	53	30.5	100	47	44	22	45	53
12449	57	63	36	98	48	56.5	27	48	73
12450	48	64	29	101	58	47.5	24.5	51	92
12451	83	80	43	146	62.5	62	39	58	78
12452	64	48	35	102	50	48.5	21	42.5	77
12453	61.5	92	32	120.5	49	55	24	53.5	69

12454	43	43	30	93	51	41	15	30	58
12455	62	56	32	118	58.5	57	24	49	79
12456	49	49	32	93	47	46.5	18.5	35	81
12457	52	64	33	102	50	51	24	49	67
12458	45	26	22	105	36	32	13	17	26
12459	43	53	28	81	45	47.5	20	38	50
12460	42	27	25	109	39	35	13	22	62.5
12461	42	42	24	96.5	46	38	18	31	53
12462	46	56	31	92	45	43	24	39	53
12463	51.5	73	35	119	49	52	26	59	83
12464	50.5	31.5	47	114	46	32	12	18	22
12465	46	45	28	105	45	42.5	19	32.5	48.5
12466	53	52.5	29	131	46.5	47	21	37	55
12467	34	23	17	118	33	29	9	16	35
12468	36	25	19	92	40	31	10	14	57
12469	31	25	14	62	38	29.5	10	13	22
12470	36.5	25	15	78	38	27	9	14	50
12471	44	26	25	127	42	33	28	24	30
12472	36	30.5	21	77	39	33	11	17	82
12473	39	28.5	24	109	40.5	32	21	24	34
12474	49	26	15	53	45	43	11	48	27
12475	51	23	17	66.5	55	34	14	21	35
12476	164	32	23.5	101	45	87	20	27	69
12477	102	26	22	57	40.5	30	18	61.5	83
12478	38	28	18	61	36.5	27.5	10	16	55
12479	31	22.5	13	53	39	27	8	11	19
12480	65	26	15	50.5	46.5	39.5	11	41	22
12481	60	29	23	89	44.5	35	24.5	46	29
12482	70	41	26	98	292	56	17	24	42
12483	52.5	38	33	140	66.5	46.5	17	32	43
12484	51	37	42	117	61	45	13	26	36
12485	50	32	24	74	78	39	23	16	30
12486	48	38	26	91	64	46.5	19	23	44.5
12487	112.5	83.5	58	175	552.5	70	74.5	126	75.5
12488	145	63	75	197	162.5	67	105	88.5	95
12489	151.5	66	67	222.5	127.5	83	212	85	97
12490	77	46	43	149	384	54	59	82.5	108.5
12491	38	33	18	61	48	41	13	17	28
12492	60	36	25	102	66	42	22	26.5	65
12493	53.5	37	43	127	55	51	19	107	77
12494	195	53	22	113	49	163	18	17	136
12495	32	29	17	52	43	34	11	13	26
12496	39	34	17	57	50	45	12	11	29
12497	76	90	50	123	74	70.5	35	62	100
12498	63	67	37	124	87	62	27	44	66
12499	98	65	35	107	67	70.5	23	43	61
12500	69	57	40	121	62	56	23	44	88

12501	67	79	38	115	59	64	27	47	92
12502	57	53	30	119.5	58	55	19	41	58.5
12503	67	70	40	138	58	56	26	52	84
12504	68	93	42	130	72	75	35	58	89
12505	84.5	65	48	109	59	60.5	27	47	93
12506	61.5	57	34.5	116	56	56	21	43.5	61
12507	61	55	35	121	54	61.5	23	50	61.5
12508	71	77	41.5	154	58.5	67	33	61	91.5
12509	71	82	44	162	65	69	30	62	79
12510	52	44	31	101.5	47.5	49	16	29	63
12511	85	71	37	104	55	61	26	51	76.5
12512	59	70	37	126	53	59.5	24	54	84
12513	59.5	66	60	114	59	59	22	42	71
12514	79	80	40.5	122	56.5	62	28	57	83.5
12515	73	72.5	66	142	67	66	27	64	152
12516	66	86	42	134	71	67	31	60	88
12517	92	54	27	104	50	76	33	30	50
12518	63.5	67	35	142	57	58	25.5	47	70
12519	72.5	97	51	148	64	70.5	39	77	101
12520	65.5	69	39	122	59	61	30	58	93
12521	67	50	30	108	53	56	19	39	59
12522	81	74	38	126	56	60.5	29	53	110
12523	56	53	32	124	67	49	23	37	62
12524	69	88	58	131	61	66	30	61	96
12525	129.5	61	39	127	61	55.5	27	54.5	83
12526	61	47	37	141	54	53	20	39.5	51
12527	52	45	33	95	51	44	16	33	73
12528	61	70.5	38.5	121	60	56	28	53	70
12529	65.5	47.5	60	125	115	47	70.5	39.5	52
12530	71	50	31	109	56.5	49	26	37	64
12531	51	34.5	25	92	62.5	35	16	27	47
12532	54	58	28	112	50	54	20	47.5	58
12533	56	74	34	116	57	58	26	54	77
12534	64	54	32	124	52	56	33	34	55
12535	70.5	45	27	107	46	63.5	15	35	50
12536	54	54	31	101	58.5	48	24	41	59
12537	52	51	29	110	44	49	22	38	61.5
12538	57	48	38	97.5	56	46	106	41	55
12539	69	51	29	98.5	44	62	20	34	73
12540	62	63	41.5	116.5	57	51.5	26	48	121
12541	43	30	22	80.5	41	39.5	12	21	48.5
12542	60	50	36.5	104	57	46	25	43	106
12543	49	57	34	108	58	51	24	41	66
12544	49	47	30	91	46	44.5	20	39	68.5
12545	77	51	31	124	54	64.5	23	39	61
12546	104.5	48	30	114	48.5	43	37	48	69
12547	99.5	87	50	129.5	60	76.5	34	62	84

12548	163	100	42	135	67	68	35	93	105
12549	60	93	48	124.5	53	60	30.5	74	92
12550	36	24	12.5	63.5	36	32	11	12	27
12551	32	25	19	51	33	32.5	10	13	23
12552	56	29	21	141	43.5	37	13	25	41
12553	60.5	32	16.5	58	44.5	59.5	10	19	32
12554	103	44	37	93	58	66	31	47	45
12555	41	29	18	66	42.5	33	11	22	30
12556	38.5	25	15	58	37	32.5	10	14	28
12557	42	27	13	64	39	35	13	30	23
12558	43.5	27	26	105	42	31.5	13	21	33.5
12559	42	23.5	14	53	39.5	30.5	13	14	25
12560	31.5	25	13	45	37	30	9	10	20
12561	45	23	16	58	40	34	10	16	22
12562	54	25	18	67	47.5	35	15	24	27
12563	42	25	14	61	36	36	9	13	20
12564	54	29	23	139.5	51	39	12	46	36
12565	33	22.5	15	72	37	32	11	13	22
12566	38	27	14	76	41	34	12	27	23
12567	33	24	14	51	39.5	33	9.5	15	23
12568	41	25	18	64.5	39	36	14	76	23
12569	71	36	19	76	51	50	18	138	54
12570	76.5	31	19	95.5	60.5	39	16	56	25
12571	46.5	25	17	56	64	33	38	34	26
12572	76.5	40	47.5	66	49.5	46	51	100	37
12573	42	30	26	109	91	38.5	16.5	31	94
12574	217.5	29.5	26	105	68	39	20	107	39.5
12575	42	25.5	20	83	52.5	35	16	29	64
12576	31	23	12	39	31	30.5	9	12	19
12577	34	23	10	41	32	34	9	11	20
12578	36	25	16	71	35	34	11	16	22
12579	34.5	23	11	37	27	31	9	13	19
12580	33	25	15	85	36	37	7.5	16.5	23
12581	30	22	10	45	33	30	9	11	19
12582	32	20	13	85	34	29	10	17	18.5
12583	37	24	15	86.5	38	31.5	11	20	22
12584	55	25	15	61	37	35.5	13	14	25.5
12585	33	25	13	58	38	32	9	12	20.5
12586	45	26	27	87	42	31	14	25	27
12587	57	27	17	80	42	36	16.5	34	28
12588	640.5	130	33.5	69	49	645.5	15	38	170
12589	45	26	21	76	46	30	15.5	63	65
12590	103	22	16	103	43	31	11	19	23
12591	65.5	24	19	91	57.5	34	14	29	32
12592	32	24	12	50.5	34	29	8	12	22
12593	47	22	13	63	37	30	10	16	22
12594	39	25	14	71.5	37.5	31	11	50.5	30

12595	32.5	22	16	84	34	31	10	14	19
12596	57.5	87.5	64	176	47.5	65.5	19	360	24
12597	31	23	12	58	41	29.5	14	13	18
12598	43	25	17	90	54.5	37	14.5	26.5	32
12599	39	26	17	59	51	30	14	63	24
12600	55	25	35	49.5	59	36	28	22	26
12601	33	25	14	61.5	34	31.5	10	13	23
12602	66	30	25	71	54	41	37	27	30
12603	41	24	17.5	48	48	30	12.5	17	21
12604	43	25	20	115	38.5	36	10.5	20	33
12605	39	26	15	66	47	35	11	19	82
12606	47	28	20	68	59	32	14.5	106.5	89
12607	46	30	22.5	184	118.5	37.5	14	44	96
12608	60.5	25	20	77	117	35	12	31	22.5
12609	49	32	26	140	36	39	14	75	26
12610	58	38	22	95	38.5	53	16	26	41
12611	60	54	29	107	52.5	54	20.5	46	60.5
12612	85	68	34	122	52	54	26	57	86
12613	40	35	20	81.5	43	35	15	27	45
12614	50	58	32.5	105	54	47	26.5	51	64
12615	52	58	29	117	55	44	25	44.5	58
12616	69.5	74	41	118	47	57	30	59	82
12617	63	92.5	40.5	143	59	65	31	73	103
12618	61	83.5	44	124.5	53	63.5	31	62	100
12619	43.5	56.5	26	111	46	44	22	44	62
12620	54	65.5	34	102	56	52	28	53.5	82
12621	51	56	33	123	47	43.5	22	44	58
12622	60	63	30	91	49	49	24	49	75
12623	44	45.5	26	101	42	40.5	18	35	51
12624	60	55	31	97	55	47	23.5	56	71.5
12625	42	40	28	100	46	37	15	28	37.5
12626	81	54	30	119	57	44.5	31	46.5	55
12627	56	54	31	107	48	49	22	41	60
12628	48	41	24	101	45.5	42.5	17	30	46
12629	39	38	22	84	39	36	14	25	45
12630	47	49	26	89	44	45	18	36	65
12631	53	54	31.5	137	51	48	22	60	74
12632	54	63.5	38.5	109	48.5	48.5	23	44	67
12633	45	39	24.5	106	42	38	18.5	29	57
12634	60.5	53	31	98	43	46	22	45	58
12635	50	46	23	97	44	44	19	34	46
12636	42.5	35	20	92	43	34	14	28	52
12637	49	46	24	96	42	41	18	35	45
12638	137	68.5	34	118	46	60.5	24	51	60
12639	59.5	52	24	95	47.5	59.5	20	34	52
12640	271.5	100.5	43	136	51	122	28	60.5	86
12641	52.5	56	31	103	47.5	46	22	44	73

12642	62.5	55	33	125	58.5	50	26	54.5	59
12643	69	143	43	114.5	73.5	68	28.5	54	84
12644	49	41	33	139	47	37.5	19	30	57
12645	68	72.5	37	122	59	68.5	23	47	73
12646	65	61	36	135	55	58	20	44	78
12647	87.5	110	69	158	67	91.5	40	76	121
12648	87	88	42.5	185	69.5	90	31	58.5	94
12649	63	70	38	138.5	54	62	26	46.5	72
12650	71	71	60	161.5	55	66.5	25	43	76.5
12651	65.5	76.5	39	150.5	48	71	29	57.5	94
12652	80	91	49	145	67	78	33	62	103
12653	56	58	34	202	45	62	19.5	39	58
12654	66.5	66	33	151	53	62	30	42	61
12655	72.5	104.5	64	156	73	84	41	77	119
12656	56	47	28	169	55	55	16	35	53
12657	56.5	45	31	206	53	51	17	30.5	50
12658	67.5	82	40	127	56.5	61	30	58	74
12659	70	97.5	46	158.5	60	80	37	62.5	97
12660	49	40	22	108	40	46.5	14	19	43
12661	78	54	40	155.5	52.5	57	24	39	71
12662	58	67	34	177	57	61	22	45	65
12663	41	33	23	101	40	44	11	14.5	42
12664	70	69.5	40	160	57	66	25.5	52	76
12665	57	68	36.5	123.5	48.5	66.5	24	40	81
12666	57	35	18.5	105.5	51	47	22	17	33
12667	244	137	26	96	48.5	239.5	13	15	96
12668	40	32.5	19	75	49	45	10	14	25
12669	44	39	20	109	46	45	17.5	13	29
12670	53	41	20	74	45	46.5	13	21	31
12671	43	32	17	70	37	40.5	10	16	28
12672	234.5	105	60	227.5	87	97	80.5	294	153.5
12673	76	40	20	101	46	53.5	17	32	35
12674	44	28	19	97	52	35	11	21	27
12675	56	40.5	25	88	44	57	15	18	37
12676	42	32	21	87	47	46	11	19	38
12677	57	34	19	111	38	47	10	16	31
12678	43	34	23	70	42	45	12	13	28
12679	51	33	20	95.5	45	41	14	19	27
12680	34	30	17	49	42	38	9.5	12	26
12681	40	31	15	69	44	42.5	10	13	27
12682	61	37	21	124	44	53	38.5	30	32
12683	42.5	34	19	70	43	43	13	16	29
12684	76	35	31	72	59	47	18	53	36
12685	51	24	16	69	41	32	22	92	26
12686	411.5	67.5	22	122	64	371	23.5	22.5	115
12687	61	47	27	97	40	48	19	35	39
12688	46	32	25	142	62	42	15	26	30

12689	41	30.5	20	108.5	46	43.5	11	18	33
12690	50	31	23	120	39	42	14	19	31
12691	61	32	26	154	39	46	12	33	40
12692	52.5	34	26	168	49	51	17	46	57
12693	54.5	31	29	111	44	44	13	27	66
12694	50	29	23	131	40	43	37	23	41
12695	49	32	24	110	38	44	12	21	70
12696	51	27	27	144	48	36	14	27.5	36
12697	45	33	33	120	44.5	45	12	24	54
12698	40	32	18	70	37	40	9	15	43
12699	61	61	34	97	53.5	60	22	44	70
12700	36.5	33	22	100	39	40	11	14	43.5
12701	57	35	20	90	45	42	13	21	41
12702	39	31	18	85	37	40	10	16	29
12703	40	32	19	116.5	48	42	10	16	41
12704	47	33.5	26	156	45	42	13	25	40
12705	41	32	31.5	131	53	40	12	29.5	40
12706	47.5	32	24	120	42	43	43	26	43
12707	61	33	31	90	59	46	19	20	54
12708	43.5	34.5	19.5	123	44	42	18	38.5	35
12709	50.5	39	22	131	48	51	12	46	57
12710	55	36	44	192.5	85.5	47	49	32	29
12711	39	30	18	86	39	42.5	11	15	24
12712	57	43	25	121	44	51	13	18.5	32
12713	70.5	63.5	34	96.5	50	68	23	36	60
12714	67	77.5	37.5	114	63	71	26	48	85
12715	73.5	65.5	42	107	57	67	27	50	67
12716	61.5	60	42	110.5	58.5	55	24	44	94
12717	78	103	69	152	79.5	87	43	78	107
12718	75	83	47	119	65	66	33	62	80
12719	67.5	76	45	136	58.5	71	27	53	78
12720	81	79	46.5	108	60.5	79.5	27.5	60	83
12721	63	84	49	118	75	66	31	56	76
12722	84	81	53	135	79	79	30	54.5	83
12723	62.5	61	36	121	57	62	23	43	77
12724	104	55	30.5	112	51	108	18	24.5	65
12725	47	45	26	84	46.5	48.5	14	25.5	57.5
12726	67	84	47	130	67	70	31.5	68	82.5
12727	51	42	26	89	48.5	46	17.5	27	61
12728	72	82	43.5	121	59	73	33	60	80
12729	59	53	34.5	105	53	54.5	31	40.5	53.5
12730	497	53	38	120.5	70.5	51	23	47	65.5
12731	60	63	40	133	53	53.5	29.5	53	98.5
12732	77.5	90	41	138	54	69	29	57	93.5
12733	70	67	39	118	63	61	31	48	74
12734	76	77.5	56	132	54	81	28	52	79
12735	52	58	32.5	109	51	49	23	43	67.5

12736	64.5	65	33	98	49	64	27	48	75
12737	49	31	25.5	92	46	39	12	26	61
12738	56	49	29	94	44	48.5	18	38	50
12739	63	67	34	118	55	55	24	50	63
12740	57.5	45	27	103	49	45	20	36	46
12741	61	42	26	93	51	44	17	26	66
12742	56.5	70	37	122	55	52	25	50	85.5
12743	55	59	33	115	45	53	24	44	133.5
12744	73	63	30	111	47	54	22	45	63
12745	67	82	38	127	56.5	57.5	27	59	103
12746	65.5	73	53	114.5	59	61	29	58	86
12747	72.5	82	40	124	57	63.5	28	63	97
12748	56	52	32	108.5	50.5	52	19	38	51
12749	85	67	41	135	53	51	27	56.5	78
12750	52	67	37	112	51	51	29	65	73
12751	73	94	47	142	60	63	37	71	101
12752	49	29	20	84	39.5	35	10	19	32
12753	50.5	45	27	110	65	45.5	18	33	49
12754	45	26	20	79	42	33	10	12	22
12755	51	26	22	102	38	31.5	11	19	39
12756	34	22	19	67	31	30	9	11	20
12757	39	32	22	80	45	37	11	19	56
12758	56	27	24	114	66.5	36	28	20	31
12759	38	28	17	87	42.5	30	13	13	24
12760	36	21.5	18	60.5	379.5	30	11	20.5	25
12761	62.5	37	36	127.5	56	44.5	24	62	56.5
12762	31	24	20	57	42	33	8	24	42
12763	38	23	13	66	35	29.5	8	12.5	21
12764	38.5	25	26	114	45.5	33	14	17	33
12765	88	29.5	22	86	44.5	34	32	17	46
12766	34	24	14.5	43	35	32.5	8	11	28
12767	46.5	24	12.5	45	40	29	8	9	22
12768	38	23	21	49	57.5	31.5	10	13	23
12769	32	24	14	43	38	31	8	9.5	21
12770	35.5	26	16	53	45	33.5	10	13	26
12771	38	26.5	13	59	37	35	9	13	23
12772	40.5	28	18	56	46	33	15	16	108
12773	63.5	31	24	69	339	53	17	19	33
12774	35.5	24	16	52	50	34	10	11	23
12775	53	26	28	75	42	35	15	49	26
12776	51	45	32	102	62	47.5	24	32	88
12777	41	24	12	37	36	32	14	12	20
12778	42	27	20	82.5	42	34	10	14	26
12779	50	29	21	91	48	41	25	23	39
12780	40	25.5	14.5	48	40	32	9	26	23
12781	40	24	15	66	36	29	13	15	22
12782	60.5	26	25	109	47	33	14	20	30

12783	38.5	27	30	79	42	34	28.5	14	47
12784	44	28.5	25	109	56.5	39	12	19	44.5
12785	38	22	21	104	39	33	9	15	23
12786	59	26	19	98	47	38	28	28	38
12787	45	33	25	76	40.5	36	14	21	64.5
12788	51	25	23	52.5	45.5	33	15	37	49
12789	64	27	165	99.5	48.5	39	14	32.5	40
12790	50	34	24	109	71	34.5	15	31	39
12791	54.5	30	36	131	55	36	17	35	181
12792	42	22	18	108	107.5	28	35	25	33
12793	73	41	50	101	45.5	41.5	24	34.5	115.5
12794	33	24	19	72	38	32	11	16	34
12795	35	22.5	13	69	35.5	28	8	18	19
12796	39	22	15	60.5	45	27	11.5	24	31
12797	45	23	20	69	62	30	27	26.5	25
12798	41	26	17	61	41	33	10	32	24
12799	32	22	13	58	38	30	12	17	22
12800	50	80	46.5	83	47	50	17	258	31
12801	55	25	21	63.5	40	33	13	46	29
12802	46	25	16	61	37	39	8.5	23	25
12803	41	23	15.5	65	57.5	28	14	81	32
12804	46	22	16	54	38.5	30	11	24	28
12805	118	77	66	93	85.5	81	99	343	89.5
12806	47	23	18	51.5	38	30	11	36	32
12807	41	25	19	65	49	31.5	12	27	25
12808	36	22	24	108	39	29	11	23	35
12809	37	22	19	75	36	26	11	15	38
12810	39	23	15	99	35	34	21	16	30
12811	53	20	16	80	36	27.5	10	14.5	22
12812	45	24	29	138	39	32.5	20	24	26
12813	44	23	26	115	35	29	17	35	26
12814	37	23	20	54	50.5	32	29	13	24
12815	66.5	25	21	97.5	595.5	34	12	28	74
12816	66	29	22	91	41	48.5	14	25	40
12817	39	24	23	89	46.5	30	11	20	30
12818	79	39	34	148	56	39.5	22	62	56
12819	114	88	88	397	98.5	75	53	400	74
12820	152	33	46	237	50	43	30	100	51
12821	34	22	32	74	35	28	10	21	26
12822	80.5	32	28	112	66.5	32	28	50	59
12823	264	29	20	83.5	76	36	57	28	37
12824	33	25	15	65.5	36	32	9	19	20
12825	43.5	34	18	54	49	42	11	14.5	33
12826	36	25	19	74	78	28	10	18.5	40
12827	53	65	35	104	50	48	25	54	60
12828	51	60	31	91	44	46.5	23	44	59
12829	44	41	24	84	40	38	17	30.5	49

12830	57.5	58	36	121	51.5	47	47.5	75.5	66
12831	47	55.5	30	87	69	43	21.5	45	60
12832	59.5	71	36	114.5	64	49	32	59	89
12833	66	61.5	38	150	49	47	24	48	69
12834	51	64	39	103	51	46.5	23	47.5	65
12835	46	50	28	82.5	38.5	41	20	46	58
12836	62.5	75	45	108	50	56.5	34	63	81.5
12837	113	36	20	90	39	44	15	25	36.5
12838	74	74	33	119	49.5	49	28	56	78
12839	37	33	22	65	38	30	14	23	36
12840	44	60	35	107	44	42	24	48	87
12841	46	24	17.5	78	36.5	28	9	15	35
12842	59	79.5	39.5	105.5	55	57	28	64	108.5
12843	54	53	29	106	39	43	20	41	55
12844	54.5	73	38	108.5	52.5	52	35	55	92
12845	44.5	40	20	68	39	33	12	28	36
12846	52	51	25.5	125	39.5	36	18	41	50
12847	62	44	22	88	40	40	18.5	37	46
12848	36	21	15	62	33	29	11	16	48
12849	41	22	19	87	42	28	10	18	24
12850	53	66	31.5	109.5	44	44	25	56	70
12851	39.5	36	22	84.5	33.5	35	14	27	70
12852	56	73	36	140	48.5	50.5	26.5	56	71
12853	59.5	62	32.5	110	47.5	45	25	46	71
12854	48	58	27	85	42	43	21	45.5	127
12855	34	29	21	77.5	36	33	13	22	27
12856	53	54	29	81.5	41	45	22	63	52
12857	41	39	20	83	59.5	33	15	29	51
12858	56	51	27	114	40	37	21	39	51
12859	42	41	23	77.5	36	33.5	17	36	54
12860	58	80	36	104	49	50	26	63	75
12861	59	68.5	31.5	90.5	46	49	29	56	76
12862	39	34	22	76	34.5	32.5	12.5	23	36
12863	46	42	24	84	32.5	37	25	36	67
12864	47	53.5	30	92.5	42	41	20	40	54
12865	51	52	30	89	45	41	22	42	51.5
12866	47	64	32	92	42.5	47.5	24	46.5	66.5
12867	44	45.5	25	79	42	35	17	33	77
12868	60.5	40	27	89	41.5	34	16	36	51
12869	44	54	28	85	42	43	21	39	72.5
12870	68	24	33	105	34	25	12	31	38
12871	64	35	24.5	125	53.5	52	28	24	41
12872	38	35	24	76	51	39.5	11	18	29
12873	45	31	27	98	52	41	12	20.5	30
12874	40	32	20	72	52	42	14	15.5	31
12875	34.5	36	18	52	45	38	8	12	26
12876	41	31	20	84	46	36	31	15	28

12877	58	34	28	129	52	43	18	28	35
12878	53	34	24	110	61	41.5	13	22	38
12879	36	32	19	80	57	37	10	14	34
12880	44.5	30	24	77	45	42	15	15	27
12881	154.5	49	30	83.5	56	57	18	32	40
12882	33	29	19	84	67.5	35	10	14	29
12883	37	28	17	66	44	37.5	10	13	27
12884	40.5	28	19	71	41	37	11	16	28
12885	34	27.5	17	58	40	38	36	13	24
12886	47	32	21	64	44	37	17	17	30
12887	41	27	25	59	46	38	10	11	36.5
12888	67	77	51	119	51	57	25	55	71
12889	55	61	39	127.5	56	47	25	67	107
12890	51	55	32	121.5	57	51	23	52	72
12891	69	59	43	120	62	53.5	28	53	64
12892	48	71	38	123	59	50	27	56	81
12893	53.5	49	29	97	51	56.5	17	33	117
12894	67.5	88	41.5	141	61	61	28.5	76	102
12895	77	77	43	124	65	59	30	67.5	93
12896	54	65.5	37	110	52	53	26	55	70
12897	77	47	27	114	50	45.5	17	36	51
12898	46.5	56.5	36	104	51	45	22	38	57
12899	56	36	23	108	61	40	16	40	43
12900	46	53	26	98	48	43	19	37	55
12901	72.5	75	40	134	62	57.5	28	59	72
12902	41.5	41	23	100	43	35	15	30.5	42
12903	61	52	29	98	43.5	44	21	37	76
12904	43	54	27	94	41	47	20	37.5	62
12905	67	73	37	141	61	50	34	59	77
12906	152	58	31	93	44	67	21.5	42	60.5
12907	38	40	28	99	52.5	39	15.5	29	43
12908	36.5	27	21	123.5	42	35	30	21	25
12909	23	22	12	41	31.5	29	8	9	18
12910	30	26	21	74.5	106	34	9.5	13	25
12911	28	24	15	39	32.5	27	10	10	20
12912	30	23.5	15	117	31.5	30	8	14	19
12913	29.5	23	13	44	53	29	8	11	18
12914	33	24	15	93	37	30	9	13	21.5
12915	26	23	10	38	35	31	7	9.5	18
12916	28	24	14	70.5	32	29	8	10	20
12917	32	25	18	66	42	32	12	14	22
12918	31	23	19	74	39.5	28	11	14	20
12919	44	30	23	80	53	33	18	22	25
12920	23	21	9	34.5	30	26	7	9.5	17
12921	25	23	10	37	31.5	27	10	9	18
12922	43	25	21	103	38	29	9	19	23
12923	37.5	26	17.5	74	43	29	21	22	28

12924	37	27.5	20	49	59	37.5	9	14	26
12925	35	23	16	70	38	28	10	42	20
12926	91	42.5	25	102	64	81.5	16	24	35
12927	39	24	16.5	99	41	30	14	20	24
12928	26	23	14	61	37	26	8	14.5	16.5
12929	35.5	31	17	61	42	43	9.5	11	31
12930	95	127	39	143	38	166	18	73	119
12931	77	110	40	182	41	141.5	17	80	99
12932	79	106	36	134	39	126.5	18	66	88
12933	71.5	99	36	116.5	55	128.5	15	64	70
12934	73	91.5	30	105	39.5	140	18	61.5	70
12935	94	131.5	40	151	50.5	156.5	26	79	118
12936	158	119.5	43	166	46	149	25.5	74	124
12937	75	108	36.5	145	36	131	31	71	113
12938	82	90	35	137	28	129	14	62	106
12939	70	101	33	139	33	136	17	69	83
12940	78.5	119	37.5	145	39	138	19	83	90
12941	62.5	91	34	109.5	32	124	12.5	58	71
12942	68	91	33	126	34	131.5	14	59.5	79
12943	72.5	100	38	131	42.5	132	19	64	89
12944	82	108.5	37	143	50	127	21	67	95
12945	73.5	100	30	158	32	114.5	52	64	115
12946	90	107	34	140	34	178	23	87	128.5
12947	77	93	36	134.5	27	117	18	64	81
12948	59.5	85	33	116	37	110	16	56.5	74
12949	95	88	32	163	29	137	11	58.5	76.5
12950	61	87	30.5	119	29	121.5	14	58	103
12951	65	99.5	36	138.5	33	125	22.5	67	79
12952	71	88.5	31.5	108	28	165.5	11	78	74
12953	71	93	31.5	142	34	115	19	60	92
12954	64.5	89	30	145.5	33.5	118	15	67	83
12955	70.5	101	37	118	42.5	131	17	64	110.5
12956	72	83	35	131	45	99	20.5	63	87
12957	69.5	103	32	147	33	124	20	65	74.5
12958	60	85	34	129.5	36.5	117.5	15	60	74
12959	65.5	83	27	102.5	28	109	15	52	66
12960	64	92	31	128	34.5	131.5	15	63	69.5
12961	65.5	93	31	132	27	118.5	16	58.5	80
12962	64	95.5	30	131	31	116	19.5	60	75
12963	77	95.5	32	119	37	120	23	61	79.5
12964	60	88.5	30	129	37	115	15.5	64	105
12965	82	85	33	105	25	135.5	14	58.5	80
12966	67	81	31.5	135	29.5	105	16	54	73
12967	75.5	115	34.5	160	41	129.5	24	67	91
12968	53	76.5	30	100.5	32	109	10	61	58.5
12969	53	71	25	105	24	102.5	10	49	59
12970	51	72	28	107	29	114	10	54	61.5

12971	55	72	24	86	100	111.5	11	52.5	81
12972	47	69	21	68	20	106	8	44	46
12973	52	71	29	136	48	102.5	10	54	59
12974	59.5	69	29	129	31	106	20	49	72
12975	207	159	54	131	49	168	40.5	186	167.5
12976	58	76	27	126	25	108.5	12	54.5	80
12977	59	68	31	125.5	28	107.5	11	55	104
12978	240.5	63.5	25.5	115.5	27	100	11	48	71
12979	61	85	27	87	26	117	12	72	78
12980	55	79	24	80.5	65	97	9	52	54
12981	47	68	75	100.5	33	103	9	50	61
12982	47	68	25	96	21	104	9	45	69
12983	44	66	22.5	81	18	98	8	41	48.5
12984	51	72	24	81	25	106	15	63	53
12985	52	73	25	85	20.5	102	8	56	59
12986	56.5	75	25	101	22	98.5	7	50	63
12987	64	79	35	131	27	111.5	13	56.5	134
12988	85	92	51	210	117	127	16	122	138
12989	47	64	27	78	23	95	9	44	51
12990	51	72	22	85	21	107.5	18	49	61
12991	133.5	89	46	267	33	121	435.5	319.5	108
12992	46.5	63.5	27.5	98	31	95	12	49	56
12993	156.5	145	37	129	72	194	26	117	112
12994	55	64	25.5	113	18	88	12	50.5	76.5
12995	43	69.5	22	66	23	94	8	41	53.5
12996	62	69	30	133.5	35	89	13	53.5	122
12997	2265	4276	823	3690	1445	2695	1634	2085	2701
12998	49	68.5	26	75	26	89	9	44.5	53.5
12999	56.5	85	40	167.5	22	112	15	210	51
13000	52	76	27	95.5	211.5	96.5	301	50	54
13001	75	83	30	115	30.5	104	15	61	62
13002	52	83	43	106	80	111	11	54	72.5
13003	49.5	78	24	118	18	103	9	49	64
13004	47	67	24	108	20	87.5	9	42	79.5
13005	51.5	75	27	134	20	105	10	54	77.5
13006	62	91.5	29	149	26	108	15	58	79
13007	51	82	28	165	21	97	12	52.5	85.5
13008	59	76	31	109.5	102	101	16	57	65
13009	135	72	35	116	45.5	101	12	52.5	56
13010	82	79	43	108	24.5	106.5	11	51	119
13011	69	62.5	28	69	22.5	85	14	49	47
13012	52.5	66	24.5	119.5	22	97	11	55	165
13013	43	61	20	55.5	13	92	6	39.5	47
13014	116	69	27	81.5	22	103	14	63	47
13015	66	64	27	111	26	77	10	51.5	56
13016	61	70	28	86	34	104	18	49	95
13017	51	63	27	107	23	91	27	54	44

13018	50	60	21	64	20	92	11	47	44
13019	54	58	23	81	17.5	95	9	45	52.5
13020	48	60	25	78.5	23	95	23	50.5	46
13021	47.5	61	21	85.5	21	94.5	9	43	45
13022	61	59	24	104.5	28	90.5	12	45	48
13023	45	61	24	97	21	90	10	47	51
13024	55.5	59	24	68	29	92	19	40.5	59
13025	49.5	67	23.5	103	22	103	11	42	53
13026	58	80	31.5	133.5	51	98	16	74	77
13027	51	58	32.5	83	19	93.5	15	44	45
13028	48	64	23	80	23	93	30	45	46
13029	222.5	79	29	76	83	111	31	82.5	67
13030	61	64	24	67	19	103.5	13	51	44
13031	349	72	28	81	27	111	10	52	58
13032	82	91.5	27	121.5	28	128.5	16	56	85
13033	55	72	25	119.5	21	98	13	48.5	66
13034	63.5	79	28	124.5	26	118.5	13	51	61
13035	53	76	29	114.5	23	99.5	16	52	55
13036	53	76	25	108.5	24.5	95	18	52	59.5
13037	59	87	28	102	30.5	100.5	18	56	76
13038	58.5	78	24	98	23	115.5	11	47	59.5
13039	68	92.5	26	117	28	123	15	52	95
13040	58	84	29	131	37	105	20	55	68
13041	61	80	23	120	22	102	15	50	68
13042	51	77	26	114	25	103	14	52	62
13043	61.5	75	25.5	110	28	101	15	48	82
13044	65.5	83	26	115	32	105	17	48	99
13045	61.5	78	26	109	25	97	17	51.5	68
13046	51	71.5	47	94	30	98.5	12	47.5	55
13047	53	76	25	115	31	96	14	50	60
13048	64	92	31	146	28	110	22	60.5	73
13049	47.5	71	24	107	23.5	91.5	13	45	72.5
13050	59	86.5	27	125	25	104	17	52	60
13051	59.5	89	31.5	138	32	104	21	60	72
13052	57.5	75	28	101	26	98	18	55	72
13053	125.5	73	24	102	28	102	15	49	67
13054	78.5	83	34	112	29	110	19	51	78
13055	60.5	84	25	130	29	105.5	17	49	65
13056	55	86	25	126	29	104	19	55	77
13057	296	67	25	109.5	22	98	12	43	69
13058	53	78	25	98	23	100	13	50	71.5
13059	45	61	24	91	89	81	9	41	59
13060	57	68	25.5	110	16	106	10	130	70
13061	93	109	35	168	46	123.5	28	85	95
13062	51	74	26	115	23	92	12	48	96
13063	68	94	30	133	30	165	17	80	91.5
13064	57	80	31	128	30	100	15	51.5	65

13065	42.5	59	21	112	16.5	88	9	37	40.5
13066	61	84	29	125.5	39	105	16	59.5	68
13067	51	73	23	102	22.5	93	13	46	71
13068	43	55	17	54.5	15	78	9	38	47
13069	56	83	29	130	26	97.5	16	54	73
13070	57.5	83	44.5	119	27	106	18	70	68
13071	41.5	58	24	89	19	84	10	43	53.5
13072	40	59	19	103	16	87	7	42.5	38.5
13073	39.5	57	22	76	23	88.5	11	39	43.5
13074	80	60.5	20	66.5	28	91	11	59	51
13075	61.5	68.5	24	76	22.5	96	12	55	56.5
13076	65	71	22.5	94	22	117	10	53	58
13077	44	60	23	77	19	101	9	51	45
13078	60	92	32	127	33	116	12	58	82
13079	62	97.5	33	104	30	120	10	67	113.5
13080	95	99	29	102	23	119	10	75	63
13081	56	82	28	100.5	20	125	9	48	87
13082	46.5	75.5	27.5	102	21	102.5	10	49.5	68
13083	47	81	30	89	20	101	8	54	53.5
13084	62	93	31	134	38	120	12	64.5	73
13085	61	87	36	139	32	108	11	65	97
13086	67	88	36.5	133	36	119	12	84	115.5
13087	90.5	91	29	129	30	116.5	11	57	57
13088	87	92	30	124	29	139	13	62	73.5
13089	48.5	78	27.5	96	24	98	12	53	66
13090	78	90	35	128	28	112.5	15	57.5	70
13091	60	69	32	117	40	101	11	48	71
13092	55	80.5	29	94	29	109	18	52	92.5
13093	55	85	31.5	126	40	116.5	19	60	63
13094	55	84	30	92	26	117	9	72	64
13095	58	82.5	29	118.5	39	108.5	9	72.5	52
13096	71	83	27	124.5	24.5	114	13	55	57.5
13097	46	77	24	93	22	100	8	50.5	58
13098	49.5	77.5	27	100	20	112	9	48	60
13099	98.5	125	43	154	40.5	189	24.5	79.5	108
13100	89	113	43.5	143.5	36	133	23	78.5	109
13101	150.5	127	49	170.5	46	153	28	82	109
13102	90.5	126	38	148	41	150	22	78	138.5
13103	87.5	111	41	149.5	50.5	134.5	21.5	71.5	99
13104	95	125	39	169	54	142	28	82.5	111
13105	90	103	37	122	29.5	129.5	22	64	76
13106	88	117.5	41	164.5	45	136	85.5	73	98.5
13107	75	102	37	175	34	135	19	67	131.5
13108	82.5	108.5	40	153	39	155	23	96	124
13109	93	110	44	139	47	139	24	77	113
13110	83	108.5	35	152.5	37	131.5	20	67.5	109.5
13111	76	91	34	138	44	145	22	67	101

13112	68.5	97	41	117	53.5	127	19	67.5	84
13113	84	93	37	142.5	52.5	118.5	42	71.5	91
13114	75	114	41	164	41	140.5	26	73	118
13115	79	105	36	164	40	144	16	80	168
13116	84	109	38	140	68	124	22	69	86
13117	77	106	36	155	118	139	22	66.5	123
13118	101	114	44	135	41	136.5	24	70	87.5
13119	70.5	100	35	126	32	130	21	71	104.5
13120	92	96	34	140	37	125.5	18	68	82.5
13121	92.5	116.5	43.5	170	43	130.5	24	72	89.5
13122	94.5	78	31	123.5	64.5	92	18	55	65
13123	72	99	35	148.5	37	122	22	70	82
13124	81	117	37	187	48	132	26	69	118
13125	85	93	32	132	29	125	19	58	74.5
13126	56	79	29	102	31	120	13	52	98
13127	75	106.5	35	179.5	45	130	21	68	81.5
13128	81	100	34	164	46	116	23	71	103
13129	81	106	43	206	35	131	23	68.5	143.5
13130	69	85	35	139	32	109	19	58	70
13131	78	113	42	165.5	50	121	36	70	112
13132	70.5	104	35	149	42.5	128	24	62	86
13133	70	86	33	132	33	114	19	62	80
13134	93	91	35	147	37	123	15	72	119
13135	74	81	36	123	27.5	109	14	64	80
13136	67.5	71	26.5	90	127	108	10	47	58
13137	57	72	24	106	27	113	9	50.5	50
13138	50	63	26.5	92	22	115	9	51	52
13139	55.5	62.5	24	94	19	94	10	45.5	65
13140	60	68	30	72	38	106	10	91	50
13141	116.5	66	29	78.5	34	107	17	57.5	51
13142	59	72	29	86	39	106	12	51	56
13143	53	63	25	123	29	98.5	12	45	51
13144	50	65	26	109	26	108.5	10	55.5	51
13145	54.5	72	27	107	20	109	8	48	58
13146	52	63.5	29	82	23	110	8	46	52
13147	83.5	73	25	97.5	26	103	12.5	49	61
13148	60	93	128.5	111	61	119	10	92	65
13149	58	72.5	27	74	135	119.5	11	49	53
13150	54	70	22	74	28	115	9	49.5	51.5
13151	59	77	29	129	32	110.5	12	60	67
13152	54.5	71	23	84	29.5	111.5	12	48	55.5
13153	56	72	28.5	112	21.5	115.5	11	48	71
13154	48	71	22	90	19	112	8	48	51.5
13155	85	70.5	33	142	38	109	22	58	83
13156	55.5	65	26	104.5	22	92	10	42	68.5
13157	64.5	72	30	155	43	107	20	58	56
13158	56	71.5	32.5	99	30	101.5	40	59	68

13159	56	71	28	73.5	21	103	9	45	68
13160	70	72	28	103	31	105	31	50	118.5
13161	86	76	30.5	87	23	116.5	19	60	62
13162	73	74	28	78	51	110.5	13	63	67.5
13163	68	68.5	28	110.5	26	103	14	52	60
13164	113	66.5	24	83.5	28	100	16	48	50.5
13165	225	91	28.5	71	32	162	14	79.5	70
13166	53	66	26	120	25	102	12	54	47
13167	77	116.5	40	190.5	36	145	27	76	115
13168	47	56	24	92	18	91.5	8	46	41
13169	53	74	27.5	162	23	105	9	53	47
13170	61.5	82.5	28	126	24.5	120	13	123	54.5
13171	52	72	27	91.5	19	111	9	51	52
13172	52.5	66	24	102	19	102	11	46	47
13173	49	55.5	27	135	23	96	9	50	48
13174	59.5	70	29.5	199	24	111.5	12	62.5	64.5
13175	40	44	20	55.5	15.5	61	8	30	27
13176	53	70	27	131	20	102	12	55	48
13177	44.5	68	24	115	17	101	9	45	43
13178	56.5	77	27	159	21	117	11	57.5	58
13179	64	47	26	123	19	70	9	37	37
13180	52	68	33	99	26.5	107	9.5	45	49
13181	50	60	23	94.5	21	96	21	45	78
13182	78	78	34	134	27	121	16	71	76
13183	44	64	23	79.5	17	104	8	46	47
13184	51	61	26	132	22	100	11	58	58
13185	50	64	26	120	18	99.5	10	49	43
13186	61	60	22	88	19	103	7	43	48
13187	54	66	33	121	19	108.5	11	45.5	87
13188	57	61	20	74.5	23	92	9	39	47
13189	142	78	42	103	55	112	117.5	107	63
13190	97.5	127	31	216	34	115.5	18	64	98
13191	132	61	21	66	33	93	10	45	48
13192	61.5	73	27	155	22	106	10	56.5	64
13193	49	64	23	90	40	106	8	48	44
13194	62	61.5	27	66.5	21	89	16	66	47
13195	93	61.5	24	70.5	52	91	11	57	47
13196	72	66	27	76	20.5	115	12	53.5	56
13197	463	115.5	27	77.5	37	564	17	83	165
13198	53	52	27	118	95	76.5	12	44.5	51
13199	44	55.5	24	102.5	21	89	9	44	51
13200	52.5	59	28	120	25	97	9	47	65
13201	76	74	37	188	35.5	111	16	73.5	90.5
13202	70	134	61	152	21	130	16	629	85
13203	72	66.5	32	163.5	22	100.5	11	69.5	70.5
13204	54	61.5	23	61.5	20	101	10	49	39
13205	77	95	28	173.5	29	132	18	64	97.5

13206	59	86	30	131.5	31	105	21	54	72
13207	67.5	81	26	96	24	117	12	50	69
13208	65.5	84	28	114.5	25	109	16	55	75.5
13209	64	88	32	252	27	114	19	63	78
13210	57	69	55	123	26	82	15	48	63.5
13211	59	70.5	29	121	29	96	14	51	74
13212	69	109	32.5	140.5	34	117	26	59.5	101
13213	92	106	41	168	41	119	29	85	134
13214	74	102	34	141.5	59	124	28	66	94
13215	64.5	90	34	147.5	31	105.5	17	55	74
13216	71.5	115	41	96	52	140.5	25	69.5	87.5
13217	90.5	100	30	116	32	267	15	108	77
13218	58	72.5	30.5	131	28	88	16	49	76
13219	80	84	32	145	37	107	19	53.5	77
13220	62	76	29	127	29.5	95	18	50	69
13221	60	68.5	28	109	21	89	12	51	97
13222	82.5	93	37	140	31	108	24	57	95
13223	62	86	36	131	29	110	19	58	66
13224	64	87	34	155	76	106	20	58	82
13225	55.5	70	27	123	30	90	20	47	82
13226	77	101	34	176	36	105	25	60	117
13227	72	98	33	151	52	113	22	66.5	175
13228	59	74	28.5	123.5	27	90.5	21	66.5	84
13229	84	109	39	193	39	115	35	69	124
13230	65.5	82	32	139	29	95	19	52	73
13231	65	85	30	136	34	115	19	59	77
13232	70	110	35.5	151	36	110	25	66	85
13233	70.5	100	36	157.5	39	111	28	60	80
13234	89	104	40.5	148.5	38.5	116	31.5	83.5	95
13235	70.5	90	32	131	30.5	106	19	57	89
13236	54	69	26	111	20.5	99	12	47	75
13237	56.5	67	29.5	152	26	89.5	13	47	68
13238	68	87.5	29	129	28	116	19	53	73
13239	63.5	79	32	108	28	100	19.5	52	73.5
13240	53	66	35	100	23	95	13	40	64
13241	57	80	30	127.5	29	99	16	53	67
13242	68.5	109	31	140	33	130	24	60	92
13243	65.5	97	38	123.5	32	113.5	26	60.5	83.5
13244	60.5	71	31	126	30	101.5	17	50	70.5
13245	52	66	24	94	18	101	9	44	118
13246	49	67	24.5	112	24.5	106	9	45	73.5
13247	45	60	21	87	15.5	91	9	39	42
13248	48	59	21	92	19	89.5	8	38	94
13249	40	49.5	19	59.5	16	83	7	35	36
13250	73	60	21	66	20	97	11	42	53
13251	62	60	24	155	18.5	100	19	48	59
13252	52.5	61	23.5	154	21	99	24	48	50

13253	59	58.5	24	79	28	93	9	51	45
13254	50	63	24	120.5	44	102	18.5	43	52
13255	224.5	63.5	21	76	18	108	10	47.5	47.5
13256	262	72	24	79	20	100	8	56.5	45
13257	64	75	28	70	24	109	35	56	57
13258	248	67	27	89	25	124	9	42	55.5
13259	59	57.5	26	73	22	98	10	43	60
13260	104	89.5	26.5	102	48	158	14	66.5	63
13261	45	61.5	20	65	14	90	6.5	41.5	46.5
13262	935	303.5	29	99	36	1606	13	179	396
13263	60	96.5	27	88	36	133	9	65	78.5
13264	195	113	32	89	26	320	12	77.5	100
13265	70.5	89	29	104	25	131	16.5	73	87
13266	71	92	25	104	24.5	134.5	9	69	65.5
13267	98	100.5	27	101	36.5	141	9	70	72
13268	48.5	86.5	25	93	22.5	113.5	12	57.5	59
13269	61	93	30	132.5	32	118.5	25	76	79
13270	49	85.5	21	109	18	119	8	53	58.5
13271	49	84	22.5	156	19	110	8	61.5	57
13272	66	77.5	28.5	152	30	110	75	83	69
13273	69.5	80.5	48	168.5	39	125	29	75	177
13274	65	93.5	29	94	59	133	12	74	58.5
13275	61	81	22	117	22	115	10	58	54
13276	73	81	24	121	25	115	22	55	59
13277	88	78	22	103	19	111	7	47	53
13278	61.5	82.5	27	96	21	109.5	10	54.5	161
13279	58	75	24	90	90	100	8	45	55
13280	61	76.5	26	91.5	19	108.5	10	56	57
13281	54.5	78	26	101	470	107	11	54	113
13282	63	73	27	111.5	73	104	17	52	812.5
13283	52	75.5	23	108	28	94	15	53.5	47
13284	50.5	68	23	98	30	101.5	14	50	47
13285	43	72	20	66.5	49	101	7	51	50
13286	53.5	80	22	79	198	101.5	10	48	56
13287	53.5	76	23	70	19	108	21	59	55
13288	63.5	81	31	157.5	120	109	13	58	65
13289	49	74.5	22	65	23	107	8	53	47
13290	42	72.5	21.5	83	19	101.5	8	47	49.5
13291	48	78	20	97	17	116.5	7	53	54.5
13292	58	79.5	23	76	24	111.5	8	63	50
13293	46	76	23	108	33	107.5	10	49	66
13294	35	18	15	54	22	19	7	14	34
13295	42	33	22	126	22	28	14	51.5	43
13296	40	24	24	65	25	19	18	17	39.5
13297	47	43	31	117	26	37	16.5	32.5	66
13298	43	37	23	97	113.5	30	12	24	59.5
13299	49.5	30	20	92	35.5	29	22	22	30.5

13300	54.5	53	30	107	29	44	18	31	60
13301	38	25	16	77	32	19	12	20	33
13302	25	19	12	57	37	12	7	25	17
13303	39	24.5	15	71	25	18	11	17	40
13304	34	19	17	69	17	20	9	19	31
13305	52	25	23	82	18	23	10	27.5	30
13306	45	31	29	80	23	24	14	20.5	26
13307	40	16	15	41	22	18.5	6	14	26
13308	32	18	14.5	49	16	17	8	15	35
13309	39	28	14	43	23.5	22	12	18	31
13310	71	75	44	173.5	44	88	23	53	102.5
13311	68.5	62	43	160	54	58	30	44	64
13312	50	35	27.5	114	29	33	19.5	29	47
13313	68.5	97	42	192	33	112	20	71	86.5
13314	30	21	19	62	16	22	14	19	23.5
13315	54.5	41	22	112	31	45	13	28	40
13316	29	12	13	49	22	16	6	12	19
13317	29.5	24.5	19	63.5	19	21	9.5	14	31
13318	49	39	22	101	35	29	15	30	43
13319	57	65	43	158.5	33	78	19	46	78
13320	62	56.5	32	146	31.5	58	21	47.5	82
13321	54	37.5	21	84	31	42	14	23	39
13322	43	27.5	28	121.5	29	31.5	13	26	45
13323	35	23	18	68	29	31	8	17	35.5
13324	24	15.5	11	41	20	14.5	7	15	27
13325	45	35	24	105	24	37	11	27.5	42
13326	75.5	74.5	30	158	44	100	21	55	81.5
13327	64	53	27	139	44	53	19.5	31	62
13328	77	74	37	195	60	60	26.5	52	76
13329	77.5	58	35	124	33	76.5	16	43	67
13330	64	62	31	133	29	75.5	15	47.5	64
13331	62	53.5	32	134.5	38	50	14	31.5	56
13332	40.5	35	17	99	22	39	12	26	32
13333	24.5	18	14	37	17	20	9	13.5	19
13334	77	61	32	156	44	51	25	43	118.5
13335	56	41	23	105.5	20.5	25	9	23	40
13336	44	30	20	109	71	29	13	26	39
13337	50	43.5	23	114.5	23	44	14.5	30	52
13338	68	82	33	172	27	107	13	59	64
13339	90	80	31.5	170	28.5	115	17	51.5	93
13340	58	40	33	147	101	46.5	13	32	51
13341	42	27	20	121	21	31	11	21	51.5
13342	16	12	9	32	8.5	9	10.5	9	11
13343	26	8.5	9	34	7	10	16	8.5	8
13344	18.5	8	9	38	9	9	5	10	15
13345	45	31	28	88	21	39	13	25	44
13346	118.5	64	29	128	29	101	20	63.5	62

13347	61.5	57	24	165	23	82	29	44	45
13348	49	66	23.5	104.5	37	53.5	11	34	29.5
13349	52	16	20	82	17	23	9	24	23
13350	61	14	14.5	103	20	17.5	7	19	15
13351	17.5	8	9	36	19	10.5	5	11	11.5
13352	66	95	31	147	45	135	14	64	65
13353	50	15	15	34	983	50.5	10	23	21.5
13354	109.5	116	41	116	2238.5	210	13	71.5	86.5
13355	167	18	22	78	44	26	19	28	33.5
13356	189	89	33	130	62	115	22	78	73
13357	150	72	81.5	160	30	114	32	63.5	66
13358	150	62	40	111.5	31	86.5	67	55	51
13359	754	76	68.5	89	65.5	208	23	55	74
13360	54	57.5	22.5	141	25	78	11	43.5	57
13361	52	48	24.5	97	35	71	9.5	40	43
13362	35.5	28	18	54	18	34	10	23	26
13363	60	57.5	32.5	170	25	71	16	38.5	85
13364	46	31	26	96.5	24.5	40.5	14	32.5	50.5
13365	87	95	40	247	39	122	14.5	82	115
13366	92	61	24	92	28.5	142.5	11	40.5	67
13367	233	54	25.5	108	25	75	11	40.5	53
13368	83.5	58	31	94	37	95	11	48	52
13369	62	62	28	114	21	94.5	15	44	79
13370	176.5	110	34.5	184	22	267	15	75	115.5
13371	66.5	70	34	191.5	27.5	91	10	59	98
13372	259	73	28	167	118	393	16	67	100
13373	50	66	23	75	22	105.5	10	43	50
13374	80	39	22.5	66.5	32	64	10	32	42
13375	374	53	25	133.5	81	194	30	37	74
13376	49	47	25	110	56	69	9	37.5	60
13377	76	80	40	305.5	31	112	34	69	99
13378	123	71.5	48	317.5	86.5	99.5	94	141	144
13379	65	54	37	177	30	73.5	19	42	109.5
13380	68	28	27	153	26	34.5	13.5	53	85
13381	86	46	29	141	44.5	66	13	47.5	52
13382	35	28	18	76	14	40	7	32.5	27
13383	30	20	19	68	11	26.5	5	20	41
13384	40.5	46	24.5	115	17	70	8	33	60
13385	26.5	10	13	75	11	13	4	10	25.5
13386	83	111.5	31.5	195	27	147	12	70	116
13387	20	10	9	36	9	11	4.5	10	15
13388	78	135	33	147	27	177	14	81.5	100
13389	17	7	8.5	24	8	10	3	8	14
13390	69.5	108.5	31	92.5	24	144.5	11	67	81.5
13391	29	21	17	65	16	31	6	20	49.5
13392	73	99.5	34	137.5	29	148.5	13	71.5	105
13393	11.5	6	6	19	4	6	2	6	9

13394	78.5	134	35	128	26	179	12	79	93.5
13395	34	25	16	74	14	34	5	25.5	52
13396	32	22	19.5	89	21	32	6	26	130
13397	75	36	19	63	17	83.5	18	28.5	65.5
13398	81	11	13	40	34	13.5	5	12	16
13399	128	88	34	96	61.5	131	14	57	71
13400	28	11	11	65	19	21	11	15	13
13401	81	97	34	183	27	135	23	72	68
13402	37	8	9	29	13	8	5	9	7
13403	111.5	89	31	106	37	127.5	15	63	64.5
13404	37	29	15.5	65	27	41.5	11.5	25	28.5
13405	72	10	6	24	55	32	5	9	13
13406	272.5	111	31	108	188	217	20	70	89
13407	47	23	18	58	16	33	6	18.5	44
13408	28.5	23	17	75.5	16	21	8	18	42
13409	28	49	25	20	27	30	38	29	23
13410	81	122	38	194	50	142	24	73	97
13411	75	51	30.5	128.5	21	63	14	38	52.5
13412	38	30	22	107	16	30	7	24	28
13413	44	63	53	50	45	56	65	64	67
13414	29	22	14	72	20	19	8	14	24.5
13415	94	132	40	193	44	168	24	81	103.5
13416	22	19	16.5	53	13	15	7	13.5	23
13417	80	99.5	48.5	196	47	117	25	67.5	93.5
13418	25	17	12	76.5	13	15	6	13	37
13419	87.5	126	36	191.5	37.5	167	21	83	142
13420	22	14	12	52.5	17	13	6	20	24
13421	72	113.5	35	166	43	134	20	71	99.5
13422	90.5	33	20	116	19	30	13	24	41
13423	46	65	70	53	57	74	89	61	73
13424	138.5	102	41	244.5	35	100	25	67	84.5
13425	54	50.5	25	146	62	52	17	34	58
13426	60	61	43	155.5	40.5	59	22	47.5	65.5
13427	48	46	23	118	23	48	12	28	89
13428	64	20	15	67	15	18	8	19	32
13429	156	117	41	197.5	36	135	24	77	112
13430	37	27	19	90.5	19	26	10	21	30
13431	82.5	133	39	190	36	151	22	74	91
13432	39	34.5	19	84.5	16	42	8	29	46
13433	46	41	20.5	121.5	26	43	13	27	48
13434	41	31	18	99	18	34	12	22	48
13435	41	75	51	49	45	57	63	62	59
13436	68	90	33	178	32	115	21.5	62	98.5
13437	48	55	26.5	144	26	57.5	17	39	54
13438	62	76.5	31	145	39	64	24	43	96.5
13439	43	37	20	118.5	27.5	38	12	26	51
13440	79	105	35	167	36	132	19	71.5	97

13441	42	40	25	139	28	38.5	13	33.5	49
13442	45	42	24	129	37	50	11	31	49
13443	62	86	29	178	48	69	28	46.5	96
13444	32	26	20	83	22	21	10	21	27.5
13445	43	43	31	98.5	42	44	13.5	29	43.5
13446	33	20	13.5	83	35	19.5	7	28	34.5
13447	91.5	122.5	37.5	217.5	62	159	20	90	120
13448	32	22	17	74	124.5	18	9	20	40
13449	78	106	36	178	155.5	120.5	26	69	104.5
13450	20.5	17	12	59	14	15	7	15	35
13451	90	128	42	202.5	45	148	29	78	144
13452	42	48	26	125	29	48	16.5	34	73
13453	85	78	32	161	28	106	16	56.5	77
13454	50	47	24	145	25.5	55	15	42	56
13455	44	40	24.5	98	24	39.5	12	28	51.5
13456	32.5	27	15	86.5	33	25	11	18	42
13457	13	8	6	28.5	21	7	3	8	8
13458	72.5	118	35	175	77	135	20	73	87
13459	22.5	16	11	38	13	13.5	7.5	13	24.5
13460	16	10	9	36	12	8	5	9	16
13461	81	130	37	197	42.5	156	25	84	110.5
13462	43	35	26	125	40	39	10	26.5	54
13463	69.5	76	35	151.5	87	82.5	27	54	99.5
13464	39	49	21	114	16	56	11	43.5	41
13465	44.5	46	24	127	19	61	11	36	81
13466	33	20	16	68	13	14	7	43	27
13467	45	28	13	65.5	16	22	10	22.5	27
13468	40	30	16	78	17	26	9	17	48.5
13469	25	15	16	47	10	18	5	11.5	41
13470	117	41	29	144	90.5	55	11	33	79
13471	56	70	30.5	142	39	69	20	49	64
13472	48	62	31	135.5	29	61	15	39	86
13473	45.5	60	26	141	25	60.5	14	34	49
13474	43	34	19	104.5	19	32	10	35	46
13475	52	50	23	131	23	62.5	12	36.5	42
13476	42.5	42	23	110.5	67.5	30	16	30.5	45
13477	49	49	30	146	31	52	16	44	59
13478	50	58.5	30	125	23	65	17	39	62
13479	54	65	27	153	35	71	15.5	45	66
13480	71	91	34	195	88	93	27	57	90.5
13481	69	63	29	157	42	66	16	44	70.5
13482	37.5	43	20	105	21	56	16	33	82
13483	55	53	28.5	134	30	80	11	69	86.5
13484	35.5	39	19	65.5	43.5	58	8	31	276
13485	36.5	41	19	67	14	66	7	28	32
13486	54	56	33	188.5	26	78	23	76	87.5
13487	50	46	25	151	39.5	71.5	12	52	44

13488	49	38	24	134.5	19	57	19	43	155
13489	60	36.5	22	81.5	15	53	9	34	47
13490	42	48	22	96	19	69	11.5	35	50
13491	59	55	25	117	21	79	11	57	45
13492	47.5	48	28	99.5	26	67	9	37.5	46
13493	47	42.5	24	95	19	65	9	30	33
13494	46.5	37	17	57	15	48	7	28	27
13495	68.5	97	33	151	29.5	147	15	69	109.5
13496	76.5	138	38	183	35.5	149	27.5	81	134
13497	90	124	35	182.5	33	158	20	75	94
13498	76.5	110	35	207.5	33.5	144	19	75	103
13499	61.5	96	32	148	26	140.5	15	66	111
13500	93.5	139	40	184	38	139.5	31	86	157
13501	85	112	35	185	31	146	20	75	90
13502	70	100	34	175	34	138	18	68	90
13503	73.5	100	42	169	28	134.5	19	66	85
13504	70	100	32.5	150	32	145	19	77	80
13505	71	110	36.5	162.5	34	135.5	24	66	89.5
13506	75	118.5	36	206.5	32.5	145.5	23	76.5	96.5
13507	70	103	41	177.5	42	135	16	68	95
13508	73.5	102	36	205.5	184	145	20	69	91
13509	81	83	34	143.5	37	141	13	64	94
13510	77	107	35.5	170	33.5	146	21	68	77
13511	79	112	32	159	46	146.5	20	75	91.5
13512	72.5	118	35	178.5	34	144.5	21	68.5	108
13513	134.5	88	28	196.5	63.5	144	12	64	88
13514	81	103	39	236	37	146	17	73	116
13515	71.5	97	29	159	28	121.5	19	60	78
13516	72	98	30	141	35	124	19	66	88
13517	57	75	28	154.5	26	108	14	52.5	91
13518	83	141	37	204	40	155	29	79	110
13519	58	89	30	164	26	125	15	69	97
13520	69	105	35	150.5	28	125	21	66	75
13521	61	72	27	135	28	102	14	52	69
13522	75	93	31	190	44	103.5	24	60	109
13523	64.5	90	32	150	29	131	16	61	108
13524	67	101	34	142	30	133	18.5	68	106
13525	72	111	67	163.5	36	147	19	74	88
13526	64.5	91	52	165	32.5	115.5	16	65	90
13527	61	90	33.5	174	36	119.5	16	65	105
13528	76	117	42	192	41.5	131	24	67	169
13529	88	121	40	175	33	231	25	117	140
13530	63	85	30	152	27	126	24	57	77
13531	70	96.5	34	144.5	27	131	20	66	75
13532	62	96	35	149	24	137.5	12	65	80
13533	53.5	78	33	128	23	116.5	12	57	90
13534	60	79	28	125.5	25	113.5	11	56	80

13535	64.5	91	28	170.5	23	115	14	56	82
13536	81	122	32	154	36	138.5	28	70	141.5
13537	65	111	31.5	173	31	126	21	67	120
13538	53.5	82	28	129	21	115	14	56.5	67.5
13539	70	101	28	131	24	139	15	62.5	101
13540	61	97	32	141	26	134	18	68	123
13541	60	98	30	164.5	28	126	17	63	84
13542	73.5	94	34	157.5	29.5	145	16	64	86
13543	72.5	109	38	179.5	32	116	27	67	126
13544	66.5	102	35.5	181	35	121	23	64	99.5
13545	69	72	33	144	25	98	16.5	47	81
13546	51	69	27	166	19	105.5	11	70.5	62.5
13547	48	74	20	83.5	14	115	8	48	45
13548	54	83.5	27	109	18.5	126	9	56	59
13549	46	71	21	121	16	109	13.5	48	51
13550	45	72	20	108	15	96	7	43.5	48
13551	48	61	23	97	17	94.5	9	44	43
13552	49.5	65	25	135	17	109	9	51	54
13553	58.5	77	27	163	20	114	9	64	52
13554	46	74	21	82	16	117.5	8	53	49.5
13555	63	78	25	126	18	119	10	66.5	55
13556	74	68	26	167	20	95	17	55	49
13557	45.5	66	20.5	127	16	110	12	45	45
13558	54	70	28.5	194	20	109	12	53	53
13559	48	63	23	109	18	99.5	10	55	41
13560	49	68	30	122	18	98	12	52	47
13561	52	61.5	27	165	22	106	9	55	135
13562	50.5	66.5	30	148	17	107	12	55	54
13563	51.5	84.5	23	67.5	15	129	9	59	56
13564	50	77	22	79	17	128	7	51.5	55
13565	66	95	33	156	30.5	131.5	18	72	66.5
13566	54	80	25	146.5	32	131	10	60	59
13567	56.5	73	27	141.5	20	110	12.5	57	69
13568	56.5	77	24	92	22	118	10	59	56
13569	54	85.5	24	92	17	133.5	8	56	59
13570	49	67	23	82	28	120.5	11	51	49
13571	52	53	21	77	17	70	10	47.5	50
13572	42	67.5	22.5	62	12	100.5	10	50	50
13573	46	59	21	84	17	86	13	40	42
13574	56	83	32	162.5	23	129	10.5	72	205
13575	67	85	64	198	52.5	117	14	70	73.5
13576	71	65.5	29	98.5	26	126	25	56	112
13577	37	64	18	61	13	92.5	10	41	47
13578	49.5	67.5	24	64	15	93	9	49	120
13579	40	58	19	89	18	101	8	44	45
13580	43.5	55	19	74	11	92	6	42	39
13581	39	61	16	66.5	13	88	7	35.5	36

13582	54	71	23	134	16.5	117	10	53	48
13583	63	85	30	154	19	134	16	64.5	60
13584	82	103	50	151	30	114	25	102	172
13585	38	64	23	54.5	28	89	9	45	55
13586	54	59	26	59	14	85	12	38	32
13587	157	64.5	20	71	17	90	7.5	43	56.5
13588	53	71	24	133	64.5	100	18	61.5	51
13589	53.5	62	21	65	49.5	92.5	11	42.5	41.5
13590	52	67	27	96.5	45	98	10	47.5	60
13591	86.5	73	25	142	21	107	11	60	93.5
13592	38	56	22	55	15	89	7	37	34.5
13593	46	70	20	68	12	94.5	7	41	49
13594	51	68	20	84.5	13	99	7	48	42
13595	57	69	26	128	23	106	11	56.5	51
13596	60.5	65	20	93.5	14	103	7	46	44
13597	43	61	19	72.5	13	104	8	42	49
13598	45	63	21	109	15	99	9	44	43.5
13599	47	63	21	94.5	13	108	10	49	49
13600	46	69	20	65	16	119	8	44	44
13601	44	58	19	68	10	84	7	41	35
13602	47.5	58	20	99	15	101	8	43	42.5
13603	44	55.5	21	98	13	82	8	55.5	47
13604	44	59.5	19	95	12	90	9	41	43.5
13605	50	67	21	138	20	101	8	44	65
13606	46.5	75	22.5	86	15	118.5	7	52.5	55.5
13607	47	75	21	84	18	102	10	50	43
13608	42	61.5	21	102	16.5	95	8	38.5	57.5
13609	63	62	20	54	14	90	8	44	38
13610	46	72.5	20	92.5	15.5	108	7	45	61
13611	43.5	60.5	21	96.5	31	85	10	45	147.5
13612	38	55	49	63	17	90	7	57	47
13613	56.5	66	23	136.5	14	97	8	52	56
13614	59	81	21	116.5	16	131	9	50.5	55
13615	52.5	82	20	75	390	122	8	56	51
13616	47	67	23	97	15	93.5	10	59	60
13617	50	77.5	26.5	120	30	91	13	59.5	71.5
13618	49	66	22	101	23	84.5	10	44	59
13619	58	82.5	30	148	41	108	16	54.5	97
13620	49	73	25	101.5	22	97	13	51	68
13621	55	87	27	145.5	30	109	18	57	79
13622	62	81	27.5	137.5	23	113	12	56	94
13623	59	79.5	30	136	23	100.5	14.5	57	106
13624	61	73	25	127	31	103	12	57	64
13625	61	99	30	126	28	114.5	18.5	60.5	103
13626	54.5	73	29	126	24	104	12	58	114.5
13627	61	84	27	137	35	110	17	53	143
13628	41	57	21	57	12	82	8	39.5	65

13629	61	88.5	26.5	130	31	109	20	56	74
13630	57	78.5	27	149.5	26	114	12	51.5	141
13631	40	65	21	91	13	87	9	39	51
13632	63	83	28	148.5	19	113	26	61	107
13633	94	82	25	120	25	104	15	50	67
13634	50	61	25	111	25	93.5	10	47	69
13635	62	87	26	117	147	117	16	61.5	76.5
13636	56	81.5	23	111	18	121	11	48.5	71.5
13637	63.5	81	27	168	35	111.5	20	56	73
13638	68	90	35	166	27	118	25	71	87
13639	44	67.5	21	119	16	84	11	44	47.5
13640	52	70	23	125.5	16.5	111.5	10	47	74
13641	62	80	26	116	26	98	17	54	92
13642	50.5	74	23	104	20.5	104.5	11	51	80
13643	63.5	91	24	102	22	121	14	59	70
13644	57	83	28	125	21	114	19	54.5	109.5
13645	58	76	24	138.5	375	102	13	49	57
13646	57	83	27.5	155.5	25	109	17	57	89.5
13647	61	101.5	30	178	28	117	18	58	90
13648	55	89	20	101	18	103	12	49	83
13649	62.5	78	31	134	16	126	11	49.5	57
13650	60	94	27.5	94.5	21	107	17	62	66.5
13651	50	72	23	144	19	112	13	51	108
13652	58	80	37	140	25	111	13	56	67
13653	74.5	89	37	149.5	40	140.5	16	67	91
13654	88.5	122	47	228.5	51.5	151	25.5	82	106
13655	93	130.5	43	188	45.5	157	26	79	156
13656	92.5	130	44	195	46	161	26	80	157
13657	113	136.5	42	187.5	50	160.5	25	77.5	136.5
13658	80	115	41	167.5	41	146	20	82	105
13659	148	142	45	196	47	156	31	82	112.5
13660	103	124	46	253	48	154.5	31	81	129
13661	100.5	123	48	253	49.5	143.5	28	82.5	116
13662	93.5	139	52	190.5	83	154	35	106	114.5
13663	345	170	41	201.5	46	271	31	91.5	209
13664	71	95	30	122	31.5	135	13	67	65.5
13665	74.5	93	36	106.5	31	122.5	16	56	69
13666	72	97	53	146	38	133.5	16	61.5	73
13667	120.5	90	26	91	32	132	114	59	65
13668	59	87	33	90	87	133	182	53	61
13669	72	86	38.5	125	70	136	13	58	99
13670	80	80	35	145	35	125	16	59	139.5
13671	72	82	31	127	23	131	10	55	95.5
13672	92	100	58	518	246	128	27	88.5	124
13673	75	86	33	118.5	92	130	12	57.5	70
13674	61	79	44.5	191.5	34	127	19.5	66	74.5
13675	72	86	27	93	24	125.5	11	52	74

13676	72	84	54	122	26	130	18	58	109.5
13677	108	89	673.5	111.5	36	141	19	60.5	75
13678	62	77	35	143	39	111.5	15	59	62
13679	66	81	31	125.5	34	131	11	76	68.5
13680	57	81.5	30	122	27	122.5	9	57	61
13681	74	109.5	38	177	35.5	135	24	66	103
13682	99	119.5	39	161	37	136.5	20	73	103
13683	74	98	35	139	32	125	17.5	62.5	87
13684	278	133	45	173	66	139	28	78	98
13685	77	105	35.5	158.5	37	133.5	23	64	111
13686	89.5	116	41	173.5	67	140.5	19	67	104.5
13687	64	94	33	98	39	125	9.5	56	99
13688	73	105	35	145	35	134	19	63	82
13689	96	95	37	158	35	131	19	71	93
13690	77.5	101	33	178	33	130	17	63.5	80
13691	80	86	33	137	28.5	129	15.5	63	100.5
13692	73	109	38	134.5	39	137	21	61	85
13693	85.5	113	35.5	182	41	135	23	66.5	103
13694	77	100	36	144.5	40	140	18	64	116
13695	73	128.5	44.5	168.5	38	152.5	26.5	71	164.5
13696	114	101.5	43	134.5	47	149	16	69	81.5
13697	69.5	102	33	150	37	125	19.5	59	110
13698	68	102	33	144	35	127	19	60	95.5
13699	76	82	35	140	30	122	15	71	81
13700	67.5	80	34	215.5	26.5	127	15	62.5	100.5
13701	58	81	30	88.5	21.5	118	10	53.5	62.5
13702	73	79	32	164	31	131	23	57.5	64
13703	50	77	27	86.5	23	114.5	9	46	56
13704	55.5	82	26	103	84	110	10	54	58
13705	63	78	28	161	20.5	112.5	11	45	90
13706	56	74	27	98.5	22	115	8	54	56
13707	98	94	42	197.5	36	141	14	66	131
13708	69	88.5	34	178	33	127	13	60	119
13709	79	86	28	87	65	138	26	52	66
13710	91	83	31.5	185.5	49	129	17	69	127
13711	104.5	78	32	75.5	33	107.5	17	74	162
13712	143.5	72	31	82	31	113	19	52	78
13713	106.5	83	75.5	103.5	67	162.5	12	57	93
13714	72	78	31	135	51	119	29	55	75
13715	606	473	187.5	335	117.5	906	78.5	457	487
13716	57	78	25	73.5	39	118	9	49.5	59
13717	69.5	87	32	96	28	122	13	66	66
13718	116	104	40	90	43	173	11	65	87
13719	63	96	32	87	28	155	11	64	68
13720	69.5	92	32	119	26	144	10	69	72
13721	112	205	47	196	59	220	37	112	144
13722	92.5	144	39	173	102.5	162	20	86	130.5

13723	96	156	37	167	54	175	26	88	120
13724	94	156.5	39	162	55.5	175	30	85.5	105
13725	103.5	158.5	49	197.5	46	178.5	25	100	114.5
13726	100	143	40	159.5	41	187.5	24	84	102
13727	82	141.5	38	200	54.5	181	22	86	124.5
13728	88	136	37	166	36	157	22	120	133
13729	78	139	38.5	160	31	164	16	80	93
13730	89.5	147	37	196.5	41	166	20	84	116.5
13731	108	172	45	190.5	59	221	32	104	136
13732	97	152	38	190	67	164.5	23	80	104
13733	88	130	36.5	166	39	163	16	88	98
13734	89	149	38	167	39	169	25	86	106
13735	87	135	40	170	39	161	23	85	103
13736	85	141	40	208.5	38	167	24.5	87	122
13737	89.5	149.5	37	186	468	165	22	85	105
13738	88	130	36	170.5	43	175	23	85.5	101
13739	83	131.5	38.5	156.5	49.5	148.5	22	75.5	111
13740	100	149	39	175	49	176	26	79	114
13741	91.5	132	37	169	51	166	23	77.5	99.5
13742	118.5	122	32	171	41	153.5	18	75	86
13743	100	125	39	213.5	58	159.5	20	83	121
13744	69.5	85.5	33	149	35.5	103	28	64.5	112.5
13745	74	131.5	37	159	34	153	25	77	130.5
13746	88	121	40	190	46	146	18	78.5	94.5
13747	68	107	30	198	24	148	16	77	76
13748	65	99	29	176	36	145	11	69	111
13749	61	121	27	139	26	152	15	68	82
13750	233	111	31	162.5	37	145	11	71	93.5
13751	87.5	125	41	309	32.5	169	18	141	142
13752	82	121	30	157	35	158	16	79	83
13753	204	105.5	31	115	25	149	11	73	90
13754	85	85	35	274	27.5	112.5	32	122	66
13755	56	79	33	171	30	112	13	97	57
13756	59	110	25	110	42	146.5	9	82.5	85
13757	58	102.5	24	80	496	139.5	10	66.5	72
13758	74.5	91	29	120.5	25	134	10	56	87
13759	95	110	31	185.5	53.5	169	13	79	125
13760	169	146	64	185	38.5	151.5	38	104.5	113
13761	69	93	32	175.5	24.5	139	11	75	86
13762	55	87	26	89.5	26	120	9	49	56
13763	62	100	23	114.5	21	139	10	67.5	60
13764	57	101	24	86.5	20	154	9	66	66
13765	57.5	102.5	24	98	23	151	9	61.5	71
13766	103.5	97	25.5	104.5	47.5	141	11	81	59.5
13767	77	111	30.5	131	431	161	13	68	128
13768	132	99.5	33	105	53	135	16	67.5	77
13769	73	97	27	88	30	137	10	65	69.5

13770	2079	591.5	47.5	142	62	3280	24	626	760
13771	84.5	97	28	103	22	138	11	63	66.5
13772	70	99.5	27	136	47.5	129.5	23	64	61.5
13773	63.5	92	24	88	31	138	14	68	60
13774	127	86	30	96	65.5	130.5	17	70	78
13775	59	91	28	155	23	143.5	11	68	73
13776	66	95	32	210	27	139.5	13	84	69
13777	55.5	94	24	72.5	19	126	9	56	57
13778	51	87	19	70	16	130	8	51	55
13779	52	92.5	21.5	76.5	17	139	8	54.5	61
13780	52	94	26	124	23	143	9	60	60
13781	54	88	21	115	20.5	128	9	58.5	58
13782	52	89.5	24	88	18	128	9	54	58
13783	52	92	24	94	39	131	9	55	60.5
13784	104.5	96	27	122.5	23	143	11	71	66
13785	52.5	102	23	92.5	22	149	10	66	62
13786	67.5	94.5	24.5	99.5	21	136.5	10	63	63
13787	45	79	20	92.5	23	114	7	51	44.5
13788	46	82	51	89	26	110	12	46	53
13789	260	207	88	238.5	59.5	251	60	232	204
13790	176.5	93	32	103	34	144	28	94	78
13791	56	93.5	26	117.5	22	128	16	55.5	60.5
13792	76.5	94	25	78.5	25	119.5	10	54	64
13793	65	84	24	87	29	114	11.5	51	50
13794	66	98	39.5	129.5	32	127	12	69	61
13795	71	89	28	95	33	132	14	54.5	77
13796	59	84	29	87.5	23	128	11	60	66.5
13797	58.5	94	28	112	26	148	11	72.5	64
13798	52.5	93	24	88.5	20	134	8	54	57
13799	56	82	25	106	16	128	9	55	66.5
13800	49	91.5	20	78.5	22	127	8	51	62
13801	56	93	23	99.5	27.5	131.5	9	63	58
13802	60.5	84	40	105	31	125	17.5	57	70
13803	55.5	93	26	101	85	132.5	9.5	56	59
13804	66	96	23	137	20.5	126	10	62	54
13805	80	85	23	93.5	99	117	9	60	56
13806	283	92	32	159	31	146	12	72.5	105
13807	149	91	25	108	21	158	20	63	68
13808	69.5	100.5	30	163	32.5	132.5	16	63	67.5
13809	51	92	21	70	14	131.5	8	57	51
13810	46	89	21	65	14	115.5	8	46	53.5
13811	86	80	29	104	22	119	12	60	66
13812	100	99	26	142	31	125	12	62	85
13813	311	83	23	104	21	120	10	55.5	80
13814	84	135	33	207	33	159	24	80	106.5
13815	81.5	111	33	155	30	138	24	71	89
13816	77.5	119.5	33	202.5	36	142	24	76.5	79.5

13817	74	129	32	173	37	150	23	71.5	87
13818	74	112	39	181.5	49.5	139.5	22	73.5	161
13819	85	133.5	37	180	42	127.5	25	69	109
13820	66	115	30	164	33	138.5	19	68	84
13821	113.5	141	32	190	36.5	148	25	70	108
13822	66	105	29	150	30	133.5	17	58.5	87
13823	75	117	30	141	29.5	155	19	75	86
13824	80	119.5	29	155	40	158	20	68.5	83.5
13825	81.5	129	36	149	39.5	142	31	78	88
13826	70	108	30	154	41	134	19	66	79
13827	79	124	33.5	168	139.5	145	22	72	97
13828	84	123	31	177	34	146.5	22	72	92
13829	83	142	38.5	186	47	144	31	78.5	125
13830	235.5	110.5	34	142	34	127.5	20	73	94
13831	73.5	138	35	181	41	146.5	27	75	130
13832	91	130	34	201	52	149.5	28.5	74	110
13833	63	111	30	150	29	144	16	60.5	100
13834	79	114	32	126	42	146	19	67	88
13835	73	112	33	153	34	131	22	63	166
13836	72	121.5	29	141	35	143.5	21	70	85
13837	94.5	151	39	160	39	169	31.5	85	115
13838	71.5	130	31	160	33	149.5	25	68	101
13839	65	111	29	154	31	129	19	63.5	102
13840	92.5	121	42	120	38	143	27	75	85
13841	99	135	37	180	35	167	41.5	91	135
13842	72	129	31.5	160	31	141	22	66	132
13843	69.5	117	29	123	27.5	136	16	62.5	79.5
13844	67.5	104	34	153	29	125	20	62	108
13845	80	138	32	210	36	151	29	74	113
13846	80	133	33	141	32	156	22	74.5	92
13847	73	114	26	126.5	31	134	23	65	99.5
13848	79	123	30	193	31	141	23	74	85
13849	82.5	119	40	145	34	142.5	21	65	114.5
13850	73.5	109	32	154	31	139	19	66	88.5
13851	71	120	37	140	30	153	18	79	96
13852	60	106.5	34	119.5	26	133	10	69.5	76
13853	83	113	38	185	126	126.5	16.5	150	71
13854	66	97	45	104	25	115	13	59.5	75
13855	140.5	113.5	33	90	29	138	11	75	66
13856	58	112	29.5	121	28	129.5	12	60	67
13857	142	170	48	229	55	207.5	34	153	127.5
13858	82	146	43	174	55	147	28	79	94
13859	74	130	42	126	38	136	22	97	89.5
13860	99.5	165	53	196.5	46	159	31	96	118.5
13861	72.5	120	38	149	36	129	20	77	79
13862	85	129	45	163	36	159	22	79	100
13863	115.5	123	36	162	37.5	132	21	75.5	86

13864	77.5	129	38	156	47	142	28	83	92
13865	79	140	41	162.5	37	155.5	32	90.5	102
13866	80.5	132	37	161	35	130	21	84	97
13867	75.5	131	40	151	39	133.5	27	77.5	98
13868	85	139	41	175	37	137.5	27	77	116
13869	71.5	99	31	155.5	47	120	12	65	74
13870	103	142	42	205	42	148	26	78.5	96
13871	106	117	32.5	151	32	143	22	69	85.5
13872	72	130	41	174	50	145.5	28	80.5	97
13873	61	104	35	126.5	25	127	11.5	60.5	72
13874	62	102	35.5	141	30	129.5	12	67	101.5
13875	63	97.5	38	156	26.5	121	10	67	81.5
13876	65	102	34	176	31	131	14	74.5	126
13877	74	98.5	36	180	31	121.5	11	72	105
13878	57	101.5	32	150	656.5	132	11	68	100.5
13879	69.5	111	31	179.5	32	141.5	14.5	84.5	99
13880	60	103	34	149.5	51	123	12	64.5	82
13881	78.5	103	35	203	33	121	12	96.5	101
13882	61	93	34	179.5	33	137	10	71	75
13883	59	93	34	178	36	115	14	79	87
13884	67	98	33	154.5	31	125.5	11	77	73
13885	71	101	31	186	29	137	12	88.5	77
13886	59	87	33	145	777	106	10	61	81
13887	60	102	33	170	35	135	11	66.5	109.5
13888	58.5	89	35	183.5	29	118	11	57	72.5
13889	60	100	31	156	30	120.5	11	66	91
13890	63	90	35	184	26	118	11.5	85.5	129.5
13891	77	86.5	33	167	31.5	111	12	113	102.5
13892	83	95	33	173	37	109	12	79	73.5
13893	68	90.5	34	178.5	38	121	13	72.5	101.5
13894	55.5	81	32	150	199	112	12	69	83.5
13895	84.5	111	43	185	40	205	13	124	103.5
13896	54.5	89	31.5	188	38.5	117.5	12	60	78
13897	61.5	92	33	160	32	115	11	75	68
13898	54	85	27	103.5	25	112	9	55	85.5
13899	63.5	88	26	100	21	108	11	55	66
13900	75.5	96	33	102	23	121.5	10	59	85.5
13901	67.5	89.5	29	101.5	23.5	114	11.5	53	103
13902	170.5	139	27	121	20	243.5	11	76	229
13903	67	94.5	36	170	26	122	15	75.5	103
13904	74	95	30	142	34	134	12	82	111
13905	75	98	32.5	150	84	124	29	67	77
13906	133.5	117	44	168.5	79	139.5	31	179	135
13907	60	86	26	113	21	122	9	54	57
13908	432.5	101	61.5	321.5	39	124.5	99	439	121
13909	160.5	88	37	160	30	118	15	69	79
13910	54.5	87	26	114	38	115	10	61	62

13911	46.5	84	25	116	18	118	8	51.5	91
13912	144.5	155	51	320	43	207.5	21	103	146
13913	129.5	147	59.5	197	64	218	32	119	129
13914	92	134	49	171	42	191	18	90	107
13915	101	151.5	49	214.5	43	209	22	105	163
13916	105	169	51	224	49	207	31	101	134
13917	98	146	45	212	37.5	204	20	89.5	126
13918	251.5	128	55	186	51	178	21	95	114
13919	163	159.5	49	190	47	243	28	101.5	132
13920	138	174	62	229	50	206.5	26	104	128
13921	103.5	146.5	46	187	44	183	23	101	116
13922	106	163.5	47.5	208.5	44	193	24	92	153
13923	105	162	50.5	229	70.5	205	37	105.5	175
13924	108	149.5	49.5	208.5	52	201	35.5	99	129
13925	211.5	162	49	213	52	201	36.5	118	179.5
13926	88	145	42	203	41	182	21	84.5	129
13927	95	145	45.5	202	42	178	25	86	137
13928	97.5	144	45	184	45	176	22	88.5	111
13929	163	149	52	206	42	204	25.5	87.5	132
13930	99	149	44	218	39.5	195	23	91	129
13931	117	152.5	48	206	51	198.5	30	104	132
13932	86.5	119	56	152	33.5	170	16	77	110
13933	87	139	42.5	176	40	187	23	90	131.5
13934	85.5	115	38	182	40.5	175	13	75	98
13935	85	117.5	40	155.5	31	163	15	78.5	93
13936	92	124	41.5	189	34.5	170	23.5	98	94
13937	281.5	236	44	207	75	444.5	26	119	203
13938	81	100	39	249	26.5	153.5	16	88.5	82
13939	72.5	94	34	153	25	147.5	23	71.5	66
13940	73	111	37	176	30	171	12	73	105
13941	102.5	109	48	216.5	41	169.5	18.5	91	90
13942	118.5	108	41	257.5	27	168	23	107	82
13943	71.5	105	38	134.5	24	161	13	74	90
13944	86.5	110	43.5	176	32	170.5	70	77	146
13945	124	119	48	257	61	182	22	124	133
13946	84	100.5	40	221.5	43	187	15	84	300
13947	83.5	102	43	159	40	157	14.5	76	141
13948	103	127	41	207	36.5	202	18	127	156
13949	98	97	37	186.5	3325	161.5	46	86	93
13950	642	1718	197	1676.5	384	869	423	595	906
13951	136	192	53	329.5	66	200	60	108	174
13952	69	90	42	175	56	156.5	21	72	103
13953	119	131	62	304	80	183	109	240	113
13954	78.5	104	35	122	24	156	14	65.5	91
13955	75	100	32	162	34	165	12	69	80
13956	99	123	34	187	41	167	17	74	129.5
13957	74.5	109	52	111	26	165.5	13	73	80

13958	73.5	104	32.5	108	25	150.5	23	72	80
13959	115	108	42.5	119.5	37	156	15	81	84
13960	80.5	105	33	115.5	26	163	12	81.5	80.5
13961	103.5	107	40.5	134	48	163	14	78.5	83
13962	118	96	45	120.5	32	196	22	86	77
13963	61.5	94	26	76	19	147	9	61	65
13964	63	100	31	94.5	32.5	154.5	11	63	88.5
13965	78	89.5	33	104	29	137	11	66.5	67
13966	67	90	31.5	107	23	151	12	67	72
13967	92	110	38	161.5	30	157	14	71	178
13968	67	102	34	172	26	155	16	71	73.5
13969	95.5	107	44	228.5	42	165.5	22	93	89.5
13970	81	100	31.5	123.5	23	157	11	70.5	133
13971	74	97	45	396	43	153	26	77	75
13972	99.5	104	41	207	1705	178	14	83	655.5
13973	83	102	39	179.5	31	150	13	69	90.5
13974	96	109	69	433.5	65	158	231	103	103
13975	121.5	109.5	39	163.5	32	173.5	17	94	141
13976	354	90	36	194.5	128	139	11	68	157
13977	89	96	59	174	39	141	32	68.5	124.5
13978	68.5	99	35	116	54	160	25	70.5	262
13979	61.5	95	29	95	31	158	11	67	84
13980	61	102	27	100	18.5	157.5	9	65	72.5
13981	56	89	23	100	155	132	10	55	64
13982	55.5	95	27	87	19	151	9	64	70.5
13983	62	92	25.5	99	20.5	148	9	64	71.5
13984	64	91	29	123	23	138	10	58	78
13985	64	86.5	26	97	45	131.5	10	60	64
13986	63	94	28.5	90	65	140	13	60.5	76
13987	76	101	41	185	83.5	130	13	63.5	89.5
13988	60	90	29	154.5	38.5	143	13	61	74.5
13989	59.5	85	26	85	21	130	9	60	55
13990	59	98	31.5	171	43	149	11	58	90
13991	63	94	32	137.5	32	146	14	67.5	73
13992	167	108	40	109	113	218	18	79	113.5
13993	85	93	32	151	27	139.5	13	70	144
13994	208	91	31	107.5	27	158	10	105	71
13995	59	78	26.5	93.5	28	134.5	11	55	57.5
13996	67.5	69	32	104	19	124	10	56	62
13997	93.5	93.5	29	125.5	28	137	14	60	67
13998	67	80.5	27	101	30	121	11.5	56	58
13999	61	95	26	103.5	24	165	9	69	69
14000	131	102	31	98.5	66	154	14	65	100.5
14001	79	95	59	248.5	31	144	43	90	85.5
14002	185	89.5	32	104	26	142	12	70	69
14003	80	115.5	36	111	50	158	47	128	102
14004	91.5	85	34	197	38	135	15	60	65

14005	93.5	97.5	59	167	29.5	150	18.5	72	107
14006	243	94	31	120	43	138	19	55	67
14007	152	88	33	109.5	69.5	146	14	62.5	78.5
14008	82	114.5	35.5	154	36	143	25	72	106.5
14009	75.5	127	39.5	179	36	157	25	81	150
14010	78.5	114	31.5	155	26	135	16.5	64	91
14011	73	113	32	132	26	158	16	71	85
14012	74	111	36	169	35	150	19	68	89
14013	181	123	42.5	207	46	158.5	23	80	91
14014	78.5	100	35	148.5	28	143	19	67	77
14015	88.5	192	34	158	115	155	19	73.5	98
14016	147	179	43	194.5	39.5	438	27	175.5	140
14017	97	118	33	162	31.5	158	21	68	94
14018	76	111	33	139	30.5	148.5	20	70.5	93.5
14019	94.5	114.5	41	181	30	144	19	68	97
14020	145	171	62.5	200.5	53.5	404	24	164	139.5
14021	91.5	120	36	181	46	165	25	76	103
14022	75.5	118	32	151.5	25	163	13.5	76.5	112.5
14023	79	129.5	39	216	34	159	23	76	94
14024	82	123	36	189	51	156.5	23.5	74	127.5
14025	76.5	118	33	177	72	151	17	69.5	88
14026	80.5	120	38	151	35	158	24	78	116.5
14027	75	119	33	157	42	145	21	64	84.5
14028	92.5	147	43	186	44.5	175.5	31	85	125
14029	71	96	33	138	29	142	15	63	84
14030	80	134	35	174	30	165	22	79	94
14031	83.5	126	34	194.5	43	149	23	75	118
14032	107	128	34	191.5	33.5	152.5	21	73	153
14033	77	120	32	166	30	143.5	20	70	85
14034	88	119	35	180	39	158.5	21	74	96
14035	89	137.5	37	167	38	160	23	75.5	102
14036	83.5	137	40	168	45	173	25	77	98.5
14037	82.5	116	49	180.5	41	153.5	19	68	163
14038	59	91.5	31	126	23	141	13	63	82
14039	89	135	42	178.5	50.5	164	31.5	81	128
14040	96.5	138	35	260	50.5	172	21	89	97
14041	74	103	36	141	27	131.5	18	65	115
14042	76	121	39.5	165.5	38	165	24	77	198.5
14043	75	116	35	159	36	142	24	65.5	101
14044	89.5	126	32	163.5	34	157	18	76	95
14045	69.5	102	33.5	139	27	143	14	66.5	85
14046	91	112	35	144	32	143	22	72	105
14047	85	137	39	207	41	164	30	81.5	108
14048	75	114	31	154	36	146	18	67	114
14049	68	100	36	173	32	149.5	18	66.5	90
14050	74	125	34	162	30	154.5	22	72	129
14051	104	185	46	182.5	40	274	16	113.5	162

14052	141	250	61	256	64.5	301	46	140	213
14053	99	196	57	200	63	259	28	116	148
14054	188	399	87	389	150	380	90	217	305
14055	94.5	186.5	45	187	30	268	15	129	124
14056	89.5	189	39.5	133	28	263	12	115.5	123.5
14057	332	187	63	140.5	45.5	261	26	131	154.5
14058	102	190	46	162	41	269	24	133	131
14059	98.5	196	44	146	40	262	15	136	121
14060	98	202	42	151	33	279	26	115.5	114
14061	97	174	41	158	28.5	252.5	12	108	115.5
14062	85	179	49	186	41.5	264	14	111	127.5
14063	87	186.5	37	115.5	32	264.5	15	114	114
14064	93	170.5	40	177	26	252.5	11	107	120
14065	89	159.5	37	133.5	29	252	12	108.5	106
14066	87	175	45	170.5	27	252	12	112	116.5
14067	103	180	39	175.5	27	263.5	14	109	118
14068	88.5	163	37	124	25	227	13	101	102
14069	99	183	40	166.5	200	257.5	14	103.5	107
14070	101	153	44.5	116	40	223	15	102	103
14071	81	181	40	145	26	256	14	108	105
14072	78.5	168	38	129.5	25.5	234	11	98	106
14073	112	175	44	146	40	271.5	22	112	113.5
14074	131.5	183	64	311	51	256	24.5	159	168.5
14075	114	170.5	44.5	144	60	261	28	117.5	109
14076	105	172	48	186.5	29	234	15	116	118
14077	108.5	169	38	194	34	243	13	114	140
14078	116	177	48	247	33	253	27	178	136
14079	94.5	189.5	40	166	28	264	13	114	121
14080	93	174	41	117	31	254	17	105	105.5
14081	80.5	165	41	128	32	239.5	12	102	109
14082	83	183	36	143	28	253	13	106	106
14083	84	172	41	179	50.5	258	14	103	110
14084	86	172	39	141	26	243	21	113.5	113
14085	85	155	42	207.5	40.5	235	13	106	123
14086	71	151	32.5	101	24	232.5	11	89	99
14087	71	155	32	98.5	25	233	9	93	97
14088	82.5	161.5	36.5	128.5	40	241.5	13.5	119	131
14089	65	125	34	130.5	25	206.5	12	85.5	83
14090	127	187.5	52	190.5	47	271	15	146	151
14091	91	157	43	223	37	239	14	102	140
14092	147	155.5	38.5	149.5	68.5	257	19	113.5	119
14093	130	133	38	160	47	219	16	98.5	101
14094	92	155	40	141	64	241.5	13	98.5	217.5
14095	99	155	46.5	220	66.5	229	20	120	221.5
14096	102.5	59	30	110.5	54.5	61	32	43	59
14097	85.5	172.5	47	194	60	233	19	124.5	229
14098	119	165.5	47	205	36	249	28	123	118

14099	110	145	36	105	25	233	12	91	120
14100	78	148.5	33	112.5	50	225	10	90	99.5
14101	72.5	146	28.5	90	21	215	11	88	91
14102	69	128	28	84	19	209.5	9	80.5	82.5
14103	63	133	28	93.5	18.5	192	10	72	80
14104	75	153	30	93	24	214.5	9	84	90.5
14105	109.5	158	39	104	455	251	22	96	129
14106	188	149	34.5	135	34	232	14	96	104
14107	61.5	129	31	91.5	18	197	10	81	78.5
14108	61	148	32	101.5	18	201	9	83	92
14109	77	154	38	136.5	29	231	13	108	108
14110	105.5	140	44	132	31	211.5	17	89	95
14111	88	149.5	48	137.5	24.5	211.5	85.5	117.5	109.5
14112	75	141.5	38	144	83	205.5	45	98	95
14113	94	153.5	46	190	44	224	17	114	115
14114	80	143.5	31	125	30	222.5	11	103	91
14115	89	154	50	197	51	230.5	13	104	111
14116	73	68	33	107	55	77	20	34.5	73
14117	56	47.5	28	113	48	59	14.5	20	48
14118	160	72	36	112	64.5	128.5	20	30.5	61.5
14119	127.5	83	41	107	66	89.5	26	48	77
14120	136	82	38	105	60	131	28	40	96
14121	94	92	39	126	62	117.5	27	44	89
14122	69.5	46.5	27	113.5	53.5	63	16	22	53
14123	67	66	36	102.5	58	71.5	22	41	72
14124	63	46.5	23	96	52	61	15	21	84
14125	69	69.5	41	126	62	73.5	32	58	70.5
14126	75	70	36	106.5	58	81	24	48	68
14127	69	66	36	104	63	77	23	34.5	70
14128	73	65.5	48	127.5	65	75	24	44	87.5
14129	84.5	82	45	131	68	82	26.5	57	110
14130	86	82	42	116.5	65	78.5	33	56	82
14131	80	88.5	40.5	112.5	57	74	27	50	82
14132	69	67.5	42	136	55	69	23	52	85
14133	68.5	59	31	100	56	63	22	40	76
14134	104	56.5	27	107	53	76	18	28	57
14135	67	72.5	40	111	58	76	23	36	77
14136	70	67	34	110	53	69	23	45	77
14137	56	67	31	100.5	52	60.5	22	40	80
14138	75.5	103	47	139	69	80.5	36	74	128.5
14139	70	90	47	117	61	77	31	57	89
14140	63	68	32	92	47.5	65	21	40	62.5
14141	64	79	39	100	64.5	66	30	48	75
14142	66.5	75	34	95	54	63	25	47.5	72.5
14143	59	66	35	92	49	66	23	41	75.5
14144	68	80	40	130	60.5	76	30	56	93
14145	62.5	56	89	99.5	59	60	21	37	62

14146	264	68.5	33.5	110.5	91	76.5	22	53	73
14147	127	89	42.5	138	60	80	29	67	95
14148	62	58.5	36	112	51.5	66	19	37	109.5
14149	68.5	88	44	109.5	74	84	35	58	104
14150	66	66	40.5	132	57	62	25.5	41	60
14151	67	75	37	99.5	49	66	27	50.5	80
14152	57	44	77	117	51	54	14	23	46
14153	63	81	35	100	65	75	25.5	48	94
14154	87	57	26	106	59	61.5	18	35.5	58
14155	75.5	92	50	115.5	61	77.5	37	64	110
14156	92	64	42	111.5	72	63	35	43	78
14157	70	36	20	68	42.5	47	11	16	39
14158	53	38	16	76	37	48	10	19	30
14159	48	37	19	68	42	50	14	13	32
14160	51	38	18	66	43.5	49	11	12	31
14161	53	39	20	96	44	53	11	20	46
14162	43	32	14	49	45	45.5	9.5	11	27
14163	51	35	31	50	46.5	52	13	39	43.5
14164	67	36	23	61	49	54	13	14.5	32
14165	72	36	18	51	44	50	12	15	29
14166	67.5	38	24	73	61	54.5	22	53	38
14167	47	36	19	76	50	51	10	70	32
14168	63	33	22	77	58	46	22	24	50
14169	53.5	37	19	49	46	109	36	18	31
14170	84	35	25	83	72	53	14	53	37
14171	74	34.5	20	69.5	71	44	15	20	36.5
14172	80	39	77	61	49	50.5	17	21	46
14173	44	34	17	64	37	50	9	16	77.5
14174	47	35.5	17	72	36	53	9	16	42
14175	42.5	33	17	71	32.5	52	10	14	34
14176	45	36	17	76	49	51.5	9	18	31
14177	44	33.5	19	66	50	49	12	15.5	40
14178	46	33	17	63	41	47	9	15	30
14179	38	29	12	49	44	47	5.5	16.5	30
14180	33.5	33	20	70	23.5	55	8	10	33
14181	47	46	26	80.5	56.5	56	13	15	64
14182	48	40	19	64	42	56	10	16	36
14183	57.5	34	19.5	150	43	46	9	16	32
14184	35.5	34	14	44.5	40	51	14	17	26
14185	44.5	36	19	72.5	101.5	47	11	23.5	69.5
14186	56	32	25	100.5	53	46	26	22	47.5
14187	47	35	26	109	47.5	46	30	22	42
14188	60	40	22	55	45	54	15	18	38
14189	42.5	31	21.5	68	44	43	11	16	48
14190	57	32	22	98	44	45	18	17	28
14191	60.5	36	25	136	48.5	46.5	25	23	35
14192	48	35	24	136.5	50	48	16	21	36

14193	52	32	21	125	54	46	44	27	34
14194	42	36	18	76	38	41	10	15	29
14195	70	42	49	119.5	72	51	20.5	39	80
14196	95	33	17	57	215	47	12	21	34
14197	65	35	29	123	52	50.5	38	33	46
14198	47	32.5	21	73	46	52	17	32	31
14199	57.5	29	15	65	132	44	18	15	49
14200	243	33.5	16.5	57	39	46	13	78	30
14201	41	30	31	82	38.5	42	15	18	160
14202	57	35	22	104	49	50	33	84	35
14203	42	29.5	26.5	54	59	45	14	27	29
14204	46.5	34	178	66	44	45	11	20	38
14205	42.5	31	19	53	64	38.5	11	24	26
14206	43	32	37	66	33	42	8	13	30
14207	49	32	21	57	45.5	41	14	21	26
14208	45.5	36	19	88.5	37	50	15	18	33
14209	42	32	21	86	41	43	12	18	72
14210	39	34	18	93	36	43	10	17	48
14211	48	44	23	78.5	39	44	14	27	73.5
14212	59	51	23	85	39	50	17	31	54
14213	50	48	24	80.5	40	53	15	40	86
14214	55	33	21	113	49	43	15	19.5	26.5
14215	41	31	15	73	39	41	9	14	31
14216	38	29	16	62.5	34	37	10	14	31.5
14217	42	35	17	74	35.5	51	12	21	35
14218	68	73	34	121	52.5	66	23	42	68
14219	54.5	58.5	36	109	67	59	19	32	58
14220	72	99	44.5	131	63	77.5	33	65	111
14221	75.5	74	38	132	63	72	25	49	68
14222	68	101.5	50	159	67	79	36	64	88
14223	60	90	41	114.5	56.5	66.5	30	51	98
14224	79	91	48	152.5	62	79	32	67	100
14225	65.5	86	45	133.5	57	70.5	23	58	85
14226	77	98.5	55	132	127	82	38	71.5	112
14227	60	69	34	119.5	59	66	23	45	62
14228	67	97	46	131	65	81	32	55.5	103.5
14229	75	86	44.5	130	65	70	27.5	55	81
14230	74	85	46	139	61.5	79	27	85	90.5
14231	79	63	36.5	118	60	60	22	39	97
14232	65	79	43.5	157	63	66.5	29	57	78
14233	76.5	88	40.5	134.5	65	67	35	58	84
14234	59.5	74	46	152	66	59	27.5	51	71
14235	54	72	38	120	59	67	28	48	77
14236	65	66	37.5	148	52	66	26	44	70.5
14237	62	82	43	134.5	73.5	64.5	37	59	89
14238	80	68	35.5	145	58	79	23	45	69
14239	63.5	82	40	122	64	67	27	54.5	71

14240	107.5	111	55	153	71	82.5	39	77	129.5
14241	55.5	56	33	119	52	55	21	52	62
14242	71.5	88	47	160	65	73.5	34	72	117
14243	63	71	35	130	60	63	25	50	70
14244	60	74	36	118	55	67	21	51	88
14245	86	97	41	123	58	77.5	31	61	94.5
14246	76	94.5	43	162.5	57	71	35	68.5	108.5
14247	70	99	55	165	66	83.5	38	67	92.5
14248	46	44.5	27	108.5	44	43	17	28	75
14249	70	79	37	111	57	70	31	60.5	72
14250	60.5	86	46	134.5	54	64	31	60	89
14251	55	74	36	120	51.5	66	23	41	63.5
14252	66.5	82	38	141	55.5	64	29	53	89
14253	55	62	27	109	48	56	21	43	78
14254	61	63	39	138	58	61	32	52	70
14255	55	61	41	105	51	58.5	21	37	53
14256	110	86	133	133	63	72	30	56	112
14257	75	59.5	30	102	48	60.5	20	35	59
14258	73	70	35	102.5	57	63	25	44	102
14259	60	63.5	35	129	51	62	26	42	70
14260	213	95	48	115	60	97	30.5	62	101
14261	59	79	41	135	53	60	28	56	84
14262	64	77.5	38	123	54	67	34	54	79
14263	62	92.5	48	133	67	71	34	62	101.5
14264	59	67.5	35	147	56.5	62	24	46	64
14265	56	81	41.5	135	57.5	64	32	56	76
14266	51	50	36	124	47	50	21	37	57
14267	72.5	87	45	131	53	68.5	35	76	79.5
14268	62	54	32.5	122.5	83	42	20	41	62
14269	51.5	57	30	128	51	49	17	39	55.5
14270	69	101	44	139	62	74.5	38	72.5	97
14271	325	80	42	118.5	60	109.5	30	54	86.5
14272	63.5	76	42	109.5	55.5	67	24	47	79
14273	59	82	44	129.5	57	67.5	33	67	80
14274	52	66	34	123	49	58	22	47	121
14275	64	77.5	41	135	49.5	65	32	61	79
14276	59	67	63	131.5	54.5	52	25	49	83.5
14277	65	65	31.5	125	53	58	23.5	43.5	112.5
14278	53	58	33	104.5	48	53.5	23	44	53
14279	66	62	36	120.5	51	65	20	47	62
14280	52	37	22	65.5	262.5	46	18	54	39
14281	78	33	34	111	63	42	36	46	74
14282	54	43	15	62	39	62	11	23	50
14283	129	71	23	95.5	51	126	16	27	93
14284	41	35	21	103	38	44.5	11	23	26
14285	75	32	15	62	39	39	11	17	24
14286	111	32	25	88	59	42	13	30	33

14287	33.5	33	16	58.5	36	40.5	11	12	23
14288	34	31	16	59	35	39.5	8	13	23
14289	37	30	17	61	38	41	8	14	24
14290	39	31	12	56	36.5	37	9	13	31
14291	50	34	16	63	41	42	11	57	30
14292	44.5	22	16	74	50	26	22	15	24
14293	55	51	22	84	50	59	13	20	37
14294	54	48.5	23.5	87	55	60	12	21	39
14295	50.5	48	23	76	56	56.5	14	18	39
14296	347.5	81	51	129.5	101.5	99	56	79	75.5
14297	47.5	43	19	67	46	51	20.5	14	32
14298	88	87	28.5	103	30	120.5	18	48	68
14299	54	66	28.5	108	38	52	21	36	72
14300	72	81	32	111	41	76.5	25	48	67
14301	67.5	71.5	32	123	42	78	26	50	74
14302	60	65	31.5	109.5	32	68	19	42	175
14303	69.5	85	34	109	34.5	73.5	21	44	104
14304	59.5	76	28	112	44	73	18	43	63
14305	52	70	28	117	35.5	77	19	43.5	98.5
14306	66	69	31	104	40	73	21	39.5	67
14307	68	71.5	32	115	32	79	23	48	80
14308	67	84	28	99	42.5	76	14	41	77
14309	65	90	32.5	137.5	38	81	25	44	88
14310	55	76	27	103.5	40	68	21	42	58
14311	57.5	70	32	118	43	62	22	42	81
14312	86	71	27	128	31.5	79.5	20	45	83
14313	48.5	53	26	103	26	59.5	14.5	35	47
14314	85	73	29	97	37	68.5	20	43	85
14315	57	47	30	96	29	60	14	34	51
14316	59	81	33	119	43	77	25	47	68.5
14317	50.5	58	26	86	32	65	20	41	67
14318	47	54	26	87	39	61	19	37	59
14319	56	60.5	25.5	93.5	26.5	62	37.5	36	49
14320	43	44	21	79	34	55	11	32	40
14321	45.5	53	23	94.5	32.5	68	15	36	51
14322	57	62	28	104.5	29	80	16.5	45.5	70
14323	51	52	34	85	30	62	15	34.5	52.5
14324	65.5	77	29	101	102	68.5	26	47	68
14325	53	69	24	101	28	63	19	39	56.5
14326	76	102	32.5	129.5	48	85.5	50	53	92.5
14327	37	35	21	80	20.5	51	8	26	42
14328	62	63	25	98	32	70	17	37	67
14329	58	69	31	115	35.5	67	24	46	56
14330	50	48.5	25	84	30	59	13	33	44
14331	51	59	30.5	95	27	62.5	16	38	56
14332	53	73	23.5	109	28	72	20	43	59
14333	74	71	27	112.5	30.5	64	19	46	61

14334	70	72	31	126	26	94	19	42	65.5
14335	49	62	22	96	24	67	12	37	71
14336	56.5	59	24	97	31	59	14	36	55
14337	52	58	30	91.5	24.5	67	17	35	64
14338	49	57	21.5	94	26	58	15	36	49
14339	59	62.5	26.5	102	29	64	19	38	54
14340	37.5	45	20	76	28	49	11	29	43
14341	48	67	30	101.5	31	60	18	37.5	57
14342	53	76.5	27	121	65.5	69.5	28	42	109
14343	33	35	15	56	15	46	5	25	27.5
14344	42	35	19	60	27	52	7	26	33
14345	31	33	15	40.5	14	49	5	22	34
14346	35.5	35	19	85	15	47.5	6	25	26
14347	33	36	18	45.5	14	51.5	7	23	30
14348	38.5	36	20	54	15	59.5	7	24	31
14349	27	30	16	56.5	17	45.5	5	20.5	25
14350	28	34	15.5	42	12	45.5	6	22	26
14351	36	33	17	66	12	47	5.5	25	29
14352	42	31	16	59.5	13	45	6	24	25
14353	34	29.5	17	59.5	12	43	6	24.5	28.5
14354	30	39	22	53	26	51	8	28	30
14355	30	31	19	55.5	18	47	6	26	28
14356	35	32	16	67	13	52	6	24	29
14357	35	28	16	57	14	45	10	23	29
14358	33	31	19	74.5	13	45	6	24	27
14359	29.5	34	14	63.5	20	51	6	28.5	28
14360	37	34	18	54	15.5	47.5	7	26	28
14361	36	34	18.5	67	15	48	6	24.5	34.5
14362	59	32	16	52	18	45	7	31	26
14363	57.5	42	19	80	20	52	8	26	90
14364	33.5	33	18	65.5	15	42	6	23	37
14365	41	29	19	90.5	28	42	12	24	27
14366	41	33	20	114	36	50	24	31	41
14367	113.5	31	16	49.5	26	47.5	10	23	45
14368	63.5	35	19	58.5	54	55.5	8	32	34
14369	86	37	20	52	19	54	13	27	46
14370	59	32	26	56	78	50	7	23	45
14371	51.5	32	19	59	21	47	9	24	37.5
14372	51.5	25	16	57	67	32.5	9	21	26
14373	39	32	19	66.5	27.5	44	12	24	62.5
14374	49.5	32	22	84	19	42	9	25	82.5
14375	38	28	14	53	18	42	10	24	24
14376	30	29.5	18	71.5	19	45	7	26.5	27
14377	47.5	28.5	15.5	62	15	41	17	21.5	27
14378	31	31.5	16	53	13	46	5	21.5	28
14379	37	30	18	76	18	47	7	26	32
14380	37	28	18.5	69	19	39	6	21	43

14381	38.5	26	17	60.5	19	34	9	24	130.5
14382	41.5	28	20	66.5	15	41	8	22	31
14383	40	33	20	89	29	52	14	30	50
14384	38	37	20	71	22	45.5	11	26.5	173
14385	44	28	15	55	18	41	7	21	49
14386	41.5	31	20	67	20	41	16	30	28
14387	29	27	16	49	49.5	42	5.5	21	47
14388	42	27	21	78.5	25	43	7	24	52
14389	41	30	16	60	17	45	7	25	33
14390	38.5	30	17	105	17	45	9	51	34
14391	30.5	27	15	51	13	41	5	21	23
14392	38	31	18	77	58.5	43	8	33	24
14393	37	33	24	77.5	98	46.5	7	25	54.5
14394	32	29	17	73	576	41.5	6	25	26
14395	31	24	14	51	92	34	5	16	24
14396	28	24	16	62	15	35	5	21	23
14397	41.5	29	15	47	15	47	6	22	29
14398	36	33	19	66	31	44	8	30	58
14399	41.5	31	18	67	15	52	10	31	43
14400	41	34	16	67	46.5	43	9	25	32
14401	43	41	23	167	24	52.5	7.5	48	41
14402	70	40	29	97	23.5	59.5	14.5	41	56
14403	23	26.5	14	47.5	12	35	4	21	28
14404	42	30	17	56.5	20	43.5	8	31	35
14405	66	33	21	82.5	29	47	9	28	49
14406	44	34	21	135	19	47	7	31	87.5
14407	41.5	32	17	56.5	58.5	49.5	10	27	36
14408	48.5	44	40	105	28	49	12	90	37
14409	47.5	29	16	50.5	17	40	10	23	28
14410	27	25.5	13	61	11	39	6	18	23
14411	54.5	75	26	102	28	65.5	23	39	66.5
14412	47	52.5	20	88	21	51.5	15	30	57
14413	49	40	19	80.5	22	55	12	29	47
14414	43	41	19	99	24	48	9	26	83
14415	52	78	20	99.5	32	60	22	41.5	111
14416	43	43	19	92	19	45	11	28	44
14417	73.5	74	27	128.5	31	61	18	36	59
14418	43	54	20.5	91.5	23.5	48.5	15	29	48
14419	53	61	25	103	28	57.5	18	38	45.5
14420	33	25	15	61	14	37	6	18.5	29
14421	44	58	21	101.5	22	56	15	31	60
14422	43	53	18	112	36.5	53	14	32	80.5
14423	63	94	27	146.5	32	64.5	27	50	95
14424	62.5	88	27	134	41	65	27	49	75.5
14425	60	71	24	92.5	23	75	19	42	56
14426	47	51	21	91.5	25	55	15	31	45
14427	45	47	22	103	30	49	14	30.5	51.5

14428	37	35	18	79	19	41	9	23	48
14429	45.5	51	20	93.5	25	49	17	31	67
14430	44	56	28	89	23.5	55	20	34.5	47
14431	50.5	59	24.5	100	29	59	16	36	57
14432	53.5	56	22	92.5	23	52	16	33.5	64
14433	68	44	18	79	20.5	47	13	28	44
14434	48.5	41	17	110.5	19	43	11	23	39
14435	46	46.5	23	78	23.5	57	16	33	45
14436	38.5	45	20	75	19.5	45	10	26	41
14437	43.5	50.5	18	84	19	49	12	28	37.5
14438	44.5	61	22	94	29	51	18	34	47
14439	39	44.5	21	79	20	47	12	26	38.5
14440	59	52	19	89	30	49.5	11	28	46.5
14441	44	57	23	84.5	25	49	20	35	50
14442	68	63	20	90	23.5	73	14.5	36	55
14443	44	55	22	93.5	26	53	16	31	47.5
14444	44	43	21	101	19.5	47	14	31.5	53
14445	45	55	19	99	24	52	15	31.5	60
14446	43	44	17	88	19.5	51.5	13	29.5	38
14447	51.5	55	22	83	24	57	15.5	33	68
14448	38.5	46	17	94.5	19	46	12	26	37
14449	44	52	18	118	20	65	12	28	38
14450	41	52	18.5	80	20	50	16	30	57
14451	35	35	18	73	15	43	10	24	35
14452	36	43	17	89	20	46.5	11	29.5	51
14453	47.5	54	23.5	115	25	46.5	41	33	48
14454	40.5	35	19	86	21	49	15	30	35
14455	46	45	20	89	24	53	11	30	44
14456	45	44	21	80	17	43	10	26	44
14457	37	39	15	82	18	43	10	25.5	47
14458	60.5	84	27	138.5	40	67	24	46	70
14459	53	54	17	83	21	72.5	12	32.5	52
14460	41.5	43	22	93	21	51	11	29	42
14461	45	44	23	79	21	54	10	27	35
14462	36	35.5	28	83.5	16	41	9	23	28
14463	354.5	182	26	96	22	546.5	14	82	135
14464	144.5	219	50	201	60	272	36	133	167.5
14465	119	170	43	221	41	277	21	109	135.5
14466	146	167	37	160	40	247.5	19	100	205
14467	243.5	154	42	143	36	232	63	106	135
14468	112.5	174	47	233	40.5	244	20.5	111	153.5
14469	110	168	43.5	172	72	223	21	101	127
14470	103	149	38	134	37	201	20	90	110
14471	183	169	35	100	139	298	11	103	111
14472	83	146	111	124	32.5	217	233	98	99
14473	91	135	36	156	60	220	16	93	107.5
14474	76.5	136	39	113	37	207	15	86	88

14475	95	136.5	35	137	35	195	14	90	108
14476	99	146	43	108	38.5	224.5	22	104	107
14477	281	150	41	135.5	39	216	24.5	93.5	99
14478	63	128	31	86	19	186	9	75.5	91
14479	123	142	40.5	115.5	167	208	20	95.5	107
14480	111	142	40	137	39	199	30	91	107
14481	70	119	30	134	25	194	11	79	89.5
14482	93	144	38	175	65	205	14	99	96.5
14483	214	143.5	39	140.5	35	328	19	108.5	209
14484	96.5	136	36	126	24	203	14	88	137.5
14485	86	141	31.5	109	46	207	13	86	93
14486	95	142.5	38	135.5	50	213.5	11	98	131
14487	148.5	155	46	140	96	248	21	106.5	109
14488	77.5	146	35.5	124	31	212	16	95	106
14489	78.5	130	31	107	24	194	11	79.5	100
14490	122.5	129	46	150	45	196	18	85	84.5
14491	96	130	38	127	29	199	14	84	117
14492	193	406	59	386.5	90	295	89	186.5	277
14493	88	129	41	351.5	41	190	15	94	108
14494	86	131	36.5	107	25	172.5	11	83.5	160.5
14495	258.5	155	52	107	60	290	22	90	115
14496	107.5	129	37	137	57	191	13	90	117.5
14497	89.5	149.5	36	158.5	29	203	16	88.5	116
14498	82	122	36	135.5	24	180.5	13	83	99
14499	72	122	32	108.5	28	197	11	85	103
14500	67	125	29	107.5	24	197	10	84	85
14501	71	117	31	103	21.5	180	10	75.5	86.5
14502	74	123	30.5	96.5	24	184.5	9.5	88.5	82
14503	85	126	31	127	29	181	21	79	115.5
14504	80.5	119	37	149.5	32.5	193	12	92	120
14505	115	120	34	110.5	34	183	12	86	105.5
14506	98.5	119	33	124	24	155	13	72	103
14507	90	113.5	62	120.5	34	174	14	74.5	98
14508	73	127	36	176	46	179	23	83	96
14509	76	120	31	96	62	176	19	82	94
14510	535.5	110	30	104	30	176.5	12	73	79
14511	89	126.5	31	142	46	191	15	74	80
14512	171.5	118	29	127	90.5	195	15	81	89
14513	471	107.5	34	92	37	172	18	82.5	87
14514	79	108	28	102	27.5	176	11	76	77
14515	77	106	33	136.5	24	172	47.5	78	80
14516	70	99	32	146	26	173.5	16	71	98
14517	70	113	28	125	24	166	10	74	85
14518	106	118	35.5	113	26	178	12.5	222	72
14519	71	115.5	30	94	21.5	167	9	71	79
14520	130	155	44	170	64	204	32	115	138
14521	67	85	25	77	25	144	15	60	68.5

14522	463.5	212	33	317	37	693	20.5	155.5	275
14523	106.5	115	33	148.5	26	168	28	194	87.5
14524	71	112	29	152	30	179	199	100	102.5
14525	91	116	31	138	25	179	12	74	84
14526	93	119.5	32	94.5	35	178	20	119.5	90
14527	100	124	30	104	30	194.5	22	78	81.5
14528	77	118	31	79	69.5	179	13	85	84
14529	186	100	33	105.5	32.5	162.5	36	73	76
14530	67	85	29	126	76	137	58	66	75
14531	81	125.5	31	118.5	26	173.5	15	77	140
14532	74	102	29	108.5	25	156.5	14	78	78
14533	101	111.5	28	114	20	172.5	9	84	73
14534	101.5	108	28	98	27	169.5	19	69	71.5
14535	123	218	53	219.5	47	254	28	126.5	141
14536	123	193	56	182	48	263	25	116	152.5
14537	123	231	68	198	48.5	269	27	253	176
14538	118	237	53	225	49	292	30.5	137.5	190
14539	96	247	41	237	-1	321	23.5	126.5	165
14540	158.5	211	60.5	222	65	309	25	137	153
14541	159	221	55	208	45	281.5	27	137	192
14542	139	276	69	237	59	332	42.5	156	199.5
14543	148	271.5	86	308	106	278	74.5	143.5	200
14544	110.5	148	61	203	35	222.5	20	104	152
14545	114	216	52	226	50	265	26	129	143
14546	121	201.5	54	220.5	42	273	22	126.5	150
14547	256	246	60.5	240.5	64	554.5	31	154	190
14548	146	247	63	259	57.5	307.5	39.5	181	175.5
14549	149	254	76.5	262	68	294	41	152.5	187
14550	152	254	67.5	252.5	67	323	49	148	200
14551	204	245	63.5	248.5	63.5	351	36	144	176
14552	178	226.5	56	216	74.5	346.5	46	129	163
14553	142	230	79	235	71	259	43.5	142	187
14554	121	208	58	206.5	55.5	275	27	144	173
14555	134	251	59	234	73	256	37	137	171
14556	133	201.5	58.5	264	54	283	28	133	176
14557	130	218	55	222	42	295	24	137	173
14558	113	202	55	225.5	49	260.5	29	130	239
14559	140	259	57	290	64	270	45	142	178
14560	181	326	67	295	65	414.5	51	236	226
14561	209	226	49	210	45	399	37	133.5	168
14562	115.5	190	55	219	46	253	33	132	146
14563	126	218	62	238	52.5	276	34	136	190
14564	124	204	50	205	56	281	31	131	150
14565	193	249	57	252	54	369.5	38	139	191.5
14566	106	190	54	166	41	249.5	25	111	151
14567	154	243	52	280	59	264	43	134.5	186.5
14568	108.5	172.5	48	211	46.5	217	27	108	150.5

14569	140.5	199	53	210.5	51	256	34	124	144
14570	105	166	49.5	205	47	253	23	103	159
14571	112	216	52.5	243	45	256	31	128	181
14572	104	185.5	48	186.5	40.5	246.5	23	141	187
14573	117	193	47.5	230	56	253.5	31	117	171
14574	127	194	61	219	54	251	45	117	167
14575	124	213	54	188.5	71	264	30	114	181
14576	114.5	195	52	223.5	56.5	243.5	36	121.5	148
14577	106.5	178	43.5	199	71	250	28	111	168
14578	112.5	195	47	189.5	41.5	260	26.5	119	144
14579	102	187.5	45	198	39	246	21	115.5	157
14580	121	145.5	49	175	50	214	24	96	111
14581	104.5	160	44	185.5	38	208	25	98	121
14582	81.5	142	40	146	23	216	12	96.5	112.5
14583	75.5	141	31	144	21	207	11	85.5	88
14584	102	137	60	408	38	206	67	110.5	91.5
14585	92	152	42	169	29	222	17	86	118
14586	96	169	39	177.5	27	236	16	103	130
14587	84	144.5	36	142.5	31	206	14	87.5	93
14588	89.5	129	32	123	32	198	14	90	85
14589	88	117	46	247	38	170	55	125	81
14590	76	130	40	144	57	198	31	84.5	137
14591	83	146	40	241	26	219	63.5	102	175.5
14592	84	107.5	44	221	34	171	15	82.5	116.5
14593	104	152	53	228	40	253	19	121	150
14594	89	175	39	168.5	25	250.5	18	118	181
14595	86	147.5	38	149	36	236	18	98	106
14596	96	127.5	38	187.5	47	193.5	31	106	117.5
14597	94	136	34	132	42	207	15	87	105
14598	128	144	34	108	24	217	14	82.5	88
14599	330	161	35	123	34	636.5	25	110	147
14600	86	100	35	100.5	29	146	15	66	82
14601	96	158	43	248	37	237.5	17	111	139
14602	80.5	137.5	38	131	24	194	12	87	86.5
14603	81.5	149.5	42	138	31	236.5	14	95	99
14604	75	134	35.5	117	32	212	13	91.5	84
14605	94.5	131	29.5	153	22	200	32	82.5	81
14606	73	143	33	131	22	211	11	87	92.5
14607	123.5	139	42	117	40.5	205.5	40.5	89	115.5
14608	337	125	39	144	132.5	200	16	105.5	85
14609	88.5	142	41.5	145	40.5	218	29	97	129
14610	98	138	30	104	25	197.5	10	82	87
14611	85	141	42.5	137.5	31	236.5	14	98	108.5
14612	92	110	37	93.5	27.5	177	15	80	95.5
14613	79	114.5	36	119	63.5	176	16	76.5	74
14614	88	121.5	34	207.5	71	196	319	95	80.5
14615	276.5	154.5	41	155	28.5	378	18.5	95	138

14616	71.5	128	33	103.5	44	176.5	12.5	80	92
14617	143	148	58	150.5	46	230.5	20.5	118	96
14618	82	150.5	37	161	27	230	13	90	126
14619	86	157	35.5	143	27	200.5	17	88	110
14620	71.5	121	33	130.5	23	183	12	78.5	84.5
14621	70.5	122	31	131.5	29	187	12	80	71.5
14622	103	145	37	134	40	211	11	105	105
14623	71	136	31	142	24.5	181	15	76.5	117.5
14624	192	127.5	36	119	33	199	14	91.5	91
14625	72	119	36	111.5	33	194	11	85.5	88
14626	84	128	38.5	118	24	175	14	74.5	100
14627	149.5	112	34	111	32	183.5	15	80	114
14628	83	114	32	107	93	210.5	10	68	97
14629	132.5	128	140	161	68.5	207	31	120	130
14630	119.5	112	34	97.5	237	165	20	82	81
14631	75	105	32	99	24	174	17	92.5	77
14632	80.5	122.5	38	149.5	145	183.5	14	79	100
14633	85	128	39	181	31	200	12	117	95
14634	179	111	33.5	104	40.5	177	208.5	79	79
14635	94	136	45	183.5	45	192	25	88.5	135
14636	81.5	110	37	147	37.5	164	19	80	110
14637	97.5	150	38	171.5	38	203.5	25	93	120
14638	191	197	39	168.5	36	363	19	96.5	135
14639	106	173.5	43.5	209	47.5	219	31	110	133
14640	105	166	40.5	178	38	223	24	95	128
14641	105	163.5	43	177	51	193	27	99	113
14642	89.5	162	43	179	44	181	23	238	131
14643	94	144	37.5	162.5	37	200	24	87	110.5
14644	97	173	46	187	43	233	32	109	135
14645	128	158.5	48	182.5	39	202	25.5	121	155
14646	236	228.5	46	209	40	451	27	124	166
14647	108	205	51	231	60	241	34.5	120	142
14648	111	166	44	191.5	43	210	30	99.5	147
14649	82	127	39	134	33	162	22.5	75.5	123
14650	80	140.5	36	158.5	34	182	20	83	130
14651	108	161.5	45	212.5	42	216.5	31	103	121
14652	85.5	120	50	148	27	166	18	84	111
14653	94.5	178	41.5	175	36	228	24	96	118
14654	83	141	38.5	171	35	194	21	87	150
14655	112.5	153	43	176	40	221.5	27	95	152
14656	95.5	145	51	209	36	190	27	94	188.5
14657	116	127	43	161	36	163.5	26.5	82	113
14658	89	153	43	168.5	46.5	185	26	92	137
14659	100	138	43	194	47.5	180.5	30	89	162
14660	88.5	141	61	164	30	192	18	90.5	164
14661	102	161	43	169	45	217	26	104	126
14662	103.5	159	45.5	184.5	38	228	27	107	150

14663	86	136	36	175.5	42	171	23	81	121
14664	86.5	133	38	204	36.5	170	27	82	119
14665	85	130	34	140	41.5	179	22	85	96
14666	98	153.5	43	193	42	200	28	99	115
14667	86	126	36	161	33	166	23	79	99.5
14668	79.5	129.5	35	162	55	162	21.5	77	99.5
14669	116	205	50	232	49.5	218.5	46	127	171.5
14670	94	178	42.5	174.5	49	205	26	95	124
14671	217	148	46	244	45	200.5	34	99.5	113
14672	82	125	37	148	30	192	21	87	116.5
14673	65	103	32	172.5	26	144	14	72	93.5
14674	90	120.5	46.5	198.5	35.5	150.5	22	81	113.5
14675	104	147.5	73	147	41	179	29	86.5	128
14676	86	158	45.5	187	39.5	199.5	29	102.5	148
14677	89	150	39	182	38	209	26	96	116
14678	88	113	30	103.5	23	179.5	12	84.5	85
14679	115	110	32	124	28	170	12	87	124
14680	79	113	33.5	133.5	53	176	131	83.5	124.5
14681	75	102	29	105	20	164	9.5	69.5	77
14682	82.5	105	29	105	34	167	16	72	75
14683	186	131	53.5	266.5	58	193	44	108	244
14684	74	117.5	36	127.5	24	177	12	80.5	135
14685	74	135	31	150	21	201	13	80	128
14686	75	125	35	119	25	173	14	81	110
14687	64	94	32	120	19.5	146	12	80.5	197
14688	63	102.5	27.5	113	22	159.5	10	67	75
14689	66.5	104	29	99	19.5	150	14	67	81.5
14690	326	111	28	103	63	158.5	14	83	73
14691	73.5	105	35	155.5	26	163	12	76	184
14692	74.5	110	37	128	23	166	11.5	72	128.5
14693	59.5	102.5	34	98	28	133	21	59	124.5
14694	74	108	31	127.5	28	176	14	68	75.5
14695	43	57	22.5	125	24	63	12	41.5	94.5
14696	39.5	42	21	77.5	17	55.5	10	34	54
14697	39.5	42	18	87	17	57	8	33	44
14698	53	44	24	99.5	20	52	8	36	52
14699	43	40.5	21	126	24.5	54	17	41	37.5
14700	53	46	28	139	40	61	26	45	48
14701	48.5	58	23	104	38	65	14	34	57
14702	69	59	34	140.5	37.5	75	23	73	60.5
14703	36	34	17	67	16	44	6	24	32
14704	35.5	35	18	58	16	52	8	28	31
14705	200	42	24	72.5	23	53	14.5	33	40
14706	61	49	32	67.5	31	75.5	10	54.5	42
14707	43	43	20	73	16	56	8	29.5	89.5
14708	62	45	21	99	26	60	12	37.5	105.5
14709	81	54	29	188	136	86	19	81.5	76

14710	45	42	22.5	86	41	54	25	34	48.5
14711	36	36	17	63	18	43	6	27	25.5
14712	47	35	22	69	30	50.5	13	35	32.5
14713	59	46	25	155	56	53	57	80.5	49
14714	33	39	17	53	39	55	8	27	26.5
14715	55.5	39	23	146	58	57	28	58	36.5
14716	63	36	24	153	108	52	82	55	32
14717	47	31	23	51.5	20	40.5	11	25	32
14718	497	257.5	51	115.5	29.5	492.5	21	139	224.5
14719	54	36	26.5	81	329	72	19	29.5	31
14720	41	39	22	107.5	24	54	9	34	50.5
14721	31	34	18	51	18	48	7	30.5	26.5
14722	28	40	14	42.5	13	48	6	22.5	27
14723	173	94	34	216	64	73.5	40	87.5	94
14724	100	62	32	95	56.5	145	13	75	82
14725	33.5	34	19	80.5	18	45	9	29.5	45
14726	31	33	17	45	14	50	6	21	26
14727	65.5	60	36	102	36.5	76	23	46	69
14728	72.5	65	33.5	90.5	49.5	90	18	46	61.5
14729	81	82	41	131.5	36	106	23	53	91
14730	79.5	88	39	122	44	84	24	53	85
14731	69.5	71	35	125	37.5	82	17	44	64
14732	67	73	34	99.5	36	80	22	46	81
14733	87	87	39	110.5	39	104.5	24	49	76.5
14734	173	72	37	117	37	87	24	51	139
14735	63	74	33	93	37	80	19	43	66
14736	68	75	35	131	44	81	25	51	65.5
14737	63	87.5	38	133	58	73	27	52	87.5
14738	83	108	50.5	170	57.5	95	38	69	93
14739	81	97	42	143	55.5	99	32	65	84
14740	88	98	37	172	45	84	30	53.5	133
14741	59.5	61	28.5	103	30	79	14	39	55
14742	68.5	81	37	133	38	86.5	24	61	82.5
14743	55	67	31	107	45	68	19.5	42	84
14744	83.5	70	32	131	43	80	23	48	70
14745	68	77	35	127.5	50.5	77	20	47	71
14746	68	86	41	140	43	84	31	52	89.5
14747	66	74	30	115	31	84	20	47	65.5
14748	64	64	32	115	39	76	19	40	56
14749	52	44	28	83	27	59	14	34	46.5
14750	63.5	55.5	30	108.5	592	64	17	38	70
14751	74	67	34	133	38	71	19	44	67
14752	56.5	65	37	97	37	72	19	42.5	65
14753	58	72	30	123	34	71	19	45	129.5
14754	61	67	32	115	46	73	17	41.5	112.5
14755	67.5	87	39	121	57	89	31	52	111
14756	84	98	38.5	153	69	85	30	56	124

14757	58	70	32	109	40	71	21	42	70
14758	66	82	34.5	121.5	39	85	22.5	52	84
14759	79	90	35	129.5	40	85	27	47	93
14760	72	67	47	143.5	36	79.5	18	47	75
14761	66	72	27	119	33	79	17	52	57.5
14762	56	66	37	112	39	71	23	46	61
14763	58.5	78	49	127	32	78.5	22	47	69.5
14764	54	67	30	140	33.5	74.5	16.5	45	95
14765	82	81	35.5	128.5	40	85	29	49.5	111.5
14766	64	62	29	101	29	78	16	42	72
14767	60.5	59	28	83.5	26	76	14	39	58
14768	56	66	31	103	42	66	17.5	43	81
14769	65.5	60	27	126	32	66	18	42	66
14770	69.5	74	48	160	69	70	31	91	85
14771	54.5	67	38	97.5	33	73	16	42	62.5
14772	67	80	45	137	85.5	73	26	49	75.5
14773	71	66.5	37	115	28	70	16	40	79
14774	77	79.5	36	117	43	84.5	23	49	72.5
14775	63	107	42	142	46	85	31	57	95.5
14776	38	39	25	75	18	53	8	31.5	36.5
14777	40	37	23	62.5	15	51	8	101	32
14778	32	38	20	46	15	54	6	23.5	31
14779	66	41	29	106	27	59.5	19	37	50
14780	32	37	20.5	46	42	53	6	25	30
14781	40	39	25	95	20	57	12	30	38
14782	31	36	20	48	14	54	7	27	29.5
14783	35	38	18.5	48	19	56.5	8	28	31.5
14784	37.5	41.5	22	56	17	53	10	30	34
14785	36	40	23	73	17	56	8	31	33
14786	43	42	24	81	379	57.5	11	31	32.5
14787	39	36	21	88	17	51	9.5	30	35
14788	32	41	22	56	19	49	8	32	28
14789	39	38	38	73	20	48	8	29	30
14790	31	36	17	48	16	47	6	26	26
14791	31	35	19	48.5	17	52	7	26	32
14792	61.5	36	25	80.5	18	50	8	26	30
14793	41	35	21.5	77.5	17	51	8	28	31
14794	46	37	21.5	65.5	16	54	7	28	34
14795	43	35	23	71	143	49	9	27	52.5
14796	38.5	36	24.5	71	20.5	49	9	27	44
14797	37	35	26	65	20	49	13	28	30
14798	39	36	22	58	24	54	9	29	32
14799	41	39	22	55.5	23	58	17	43	31
14800	99.5	39	26	98	24	60.5	24	30.5	45
14801	41	35	28	59	35.5	49	13.5	28	99
14802	42.5	41	27	102.5	19	56	11	46	55
14803	39.5	37	22	78	28	48	10	28.5	49

14804	62	38	22	58.5	23	85	9	29	49
14805	46.5	36	31.5	159	26	63	10	29.5	41
14806	39.5	36	24	64	27	48	8	26	33
14807	53	28	34	73	56	40	11	28	32
14808	46	36	32	53	22	56	8	29	35
14809	34	37	21	56	181	51	7	28	32
14810	33	31.5	21	54	17.5	49.5	8	27	32
14811	33	33	19	56	18	51	7	24	32
14812	42.5	38	20	69	36	54	8	37.5	32
14813	33	33	19.5	48	21	43	7	23	30
14814	42	35	24	89	20	51	9	32	65.5
14815	66	37	22	78	23	55.5	10	28	36
14816	62	49	46.5	125.5	60	58	25	41	85
14817	72	50	31	98	27	62	16	39	112
14818	64.5	34.5	27	130	20	49.5	10	31	100
14819	279	42	24	73	26	51	13	28.5	55.5
14820	34	32	19	49	23	46	9	24	29
14821	37	37.5	24	76	21	55	8	28.5	36
14822	44	39	21	64	17	54	9	27	34
14823	44.5	35	29	214.5	289	50	9	32	39
14824	40	40	23	66	18	88	8	38	33
14825	39	34	23	96.5	19	52	8	34	32
14826	79.5	36	20	54	19	53	12	40	29
14827	247.5	32	20	55	16	46	17	26	34
14828	33	33	16	54	19	45	10	23	28
14829	36	33	19	71.5	19	51	10	26	32
14830	35	35	38	58	59	51.5	7	30	34.5
14831	111	39	22	92	18	57	14	30	57
14832	101	35	28	55	23	50	12	30	32
14833	39	37	30	58	31	51	9	31	32
14834	113	42	25	68.5	24	64	16.5	37	40
14835	32	32	18	48	15.5	39	7	25	30
14836	53	37	22	60	19	53.5	10	26	45
14837	50	36	21	53	26	61	11	29	30
14838	45	38	26	69.5	19	60	8	38	47
14839	46	34	29	109	41	56	9	33	96
14840	22	17	16	45	10.5	21	6	13	18
14841	41	33	19	53	15	45	8	27	28
14842	71	51	24	53	25	68	13	49.5	59
14843	41	49.5	27	78	25	56	14	33	72
14844	62	61	25	90.5	25	59.5	20	35	54
14845	58	62	24	83	24	75	14.5	36	62.5
14846	49	65	27	93	25	66	53	39	84
14847	80	65	27.5	108	27.5	96.5	14	36	64
14848	44	44	23	92.5	25	49	14	34.5	54.5
14849	58	53	27	88	28.5	66	15	34	45.5
14850	91	79	31	127.5	35	77.5	22	53	68.5

14851	70	49	28	90	29	57	12	34	71
14852	56	61	24	93.5	25	67	16.5	35	53
14853	76.5	51	24	90	25	56	14	32	66
14854	38	45	24	83	19	59	10	31	53
14855	51	48.5	47	86	23	54	13	31	51
14856	43	39	23	78	20	48	11	30.5	52
14857	44	52	23	100	25	60.5	15	36	55
14858	680	46	29	78	53	62	12	30	51
14859	43	45	24	81	24.5	50	12	29	39
14860	67	80	28.5	95	29	81	19	40	65
14861	46	41	24.5	78	17	52.5	11	28	42.5
14862	42	49	23	125	20	49	12	33	45
14863	47	52	25	100.5	24.5	56	14	32	53
14864	59	64.5	30	105	27	61	20	40	72
14865	74	54	25	84	23	106.5	16	34	52.5
14866	52	66	23	96	40	61	19	39	66
14867	39	38	22	75	17	48	11	28	40
14868	76.5	64	22	91	25	91	22	36	59
14869	47.5	64.5	29	124.5	26	57	18	36	57
14870	35	35	21	67	18	48	9	26	38
14871	81	69	27.5	98	33	65.5	21	36.5	61
14872	66	59	23.5	84.5	20	91	14	36	54
14873	52.5	64	25	95.5	28	65	18	37	55
14874	43	54	24	84	23	56	13	31.5	51.5
14875	48	63	27	84.5	28	54	17	35.5	56
14876	51.5	51	24	86	20	66	12	35	56
14877	50	48	23	98.5	25	60	12	31	57
14878	50	50.5	25	90	24	59	14	33	52
14879	61.5	70	30	103	27	66.5	19.5	42	63
14880	55.5	62	27	104	25	59	20	39	56
14881	50	61	25	97	40	58	16	35	53
14882	42	51	22	90	30	54	14	30.5	51
14883	57	43	22	69.5	18	65.5	10	29	50
14884	44.5	38	19	81.5	17	49	14	25.5	37
14885	38	37	24	69.5	26	48	9	28	39
14886	46	45	25	90	18	48.5	12	28	43
14887	46	31	24	77	59	40	11	28	47
14888	301	57	26	130.5	24	56	17.5	34.5	65
14889	52.5	54	24	90	28	69.5	12	31	46.5
14890	50.5	53	26	91	28	55	15	35.5	63
14891	42	47	25	98	23	54	15	32	49.5
14892	40	36	20	79	17	40	10	23	34
14893	64.5	98	38	165	45	74	33	52	105
14894	66	61.5	44	113	29	64.5	14.5	46	57
14895	52	65	32	97.5	33	64	19.5	40.5	55
14896	62	75	37	114.5	43	68	20	44	68
14897	46	44	39	165	21	61	12	36	40

14898	36	39	21.5	62.5	60	52	7.5	24.5	33
14899	42	45.5	22	71	52.5	55	15	30	36
14900	41	42	21	70	26	57	29	32	33
14901	38	43	22	66	20	56	11	28	31
14902	41	43	22	83	18	53	10	29	34
14903	68	45.5	25	75	18	55	16.5	51.5	37
14904	41	42.5	21	67.5	18	53.5	12	29	31.5
14905	46	40	21	57	18	51.5	7	27	33
14906	40	40	21	74	19	48	8	28	36
14907	37.5	43	20	66.5	18	46	8	23	31
14908	40	40	20	60	21	50	8	26	33
14909	35	38	21	66	18	53	9	32	32
14910	35	38	20	59.5	19	53	8	36	34
14911	36.5	36	20.5	68	17	46	9	30	32
14912	39	39	20	60	17	55	9	29	30
14913	33	37	19	69	19	51	8	28	29.5
14914	38	37	19	79	18	50	13	28	28
14915	151.5	54	31.5	98	521	94	17	46	58.5
14916	147.5	54	49	136	30	76	101	107	46
14917	40	44	23	68	20	51	16	27	32
14918	51	50	36	104.5	29	66	12	37.5	139
14919	71.5	39	41	100.5	87.5	54	21	67.5	94.5
14920	41	40	26	61	112	59	10	35	36
14921	164	44	22.5	78	24.5	119	10	35	67
14922	40	43	21	69	26	55	11	32	68.5
14923	83	57	40	80.5	50.5	69	33	55	55
14924	44.5	70.5	29.5	101.5	32	54.5	113	38	33
14925	34.5	39	21	74	18.5	48.5	9	28	31
14926	45	38	21	52	18	52	11	24	30
14927	46	37.5	21.5	60	19	52.5	8	31.5	33.5
14928	49	39	20	66	38	53	11	31	35
14929	341	50	25	83	24	54	12	35	53
14930	51.5	75	28	116	39	68.5	20	39	56.5
14931	58	78	31	126	32.5	71	23	48	106
14932	68	93	36	162	82	74	28.5	60	135.5
14933	55.5	82	29	127	36	75	26	48	63.5
14934	57	70	31	119	34	61	18	43	63
14935	52	59	25	116	31	66	18	43.5	73.5
14936	53	54	25	100.5	30.5	56	13	33	61.5
14937	59.5	68	26	112.5	28	65	17	39	51
14938	56.5	73	31	119	38	60	19	46	76.5
14939	43	50.5	24	118	26	47.5	13	39	63
14940	48	65	24	113	35	62	15	38	57
14941	57	76	28	127.5	34	68	26	49	59
14942	52	60	27.5	126	29	59	34.5	44	51
14943	56	69	29	119	36.5	66	20	48.5	59
14944	51	55	22.5	87	24	56	14	32	47

14945	51.5	66.5	27	113	30	60	20	39	56
14946	42.5	49	23	89	22	56	12	30	45
14947	45	69	24	108.5	27	61	16	41	56
14948	51	85.5	30	115	34	70	25	46	77
14949	44	50	30	95	36	53	23	34	49
14950	48	62	25	105.5	26	60	17	39	58.5
14951	46	53	27	100	25	50	14	35	44.5
14952	74.5	87.5	28	128.5	34	73	25	53	69
14953	47	66	27	117.5	38	67	18	46	51
14954	61	58	24	103	48	56	17	38.5	62
14955	57	63	26.5	103	29	58	18	38	57
14956	54	72	31	130	39	64	22	45.5	85
14957	36	48	24.5	90	25	53	11	35	38
14958	39	41	22.5	96	18	50	8	26.5	45
14959	57	74	31	112.5	28	67	19	46	61
14960	47.5	44	24	95.5	24	52	17	34	50
14961	54	56	21	95	28.5	70	15	35	67
14962	74	58	21	108	27	60.5	17	34.5	51
14963	48	61	26	100	30.5	60	16	39	59
14964	60	71	28	123	26	75	17	40	70
14965	46.5	52	23	94	20.5	51.5	13	32	39
14966	44	52.5	24	96	25	58	13	34	57
14967	58	74.5	28	128.5	31	61	18	43	62
14968	40	42	21	75	21	48	9	29	40
14969	37	41	22	97	25	50.5	10	40	41
14970	39	38	21	107.5	23	55	10	40	43
14971	42	41	23	95	26	49	8.5	35	34.5
14972	36.5	38	24.5	83.5	23	53.5	10	37	61.5
14973	40	40	23	92	36	47	9	37	40
14974	41	42	20	90	23	54.5	7	34	35
14975	38	40.5	24	123	30	51	10	35	54
14976	65.5	77	32	122	34	77	19	64	112
14977	56	61	27	99	25	78	10	44	95.5
14978	54	54	29	92.5	24	65	12	36	54
14979	91	61.5	29	88	27	78	14	48	74
14980	49.5	62	27	101	24	70	12	42	83
14981	65.5	54.5	30	127.5	77	65	11	47	63
14982	51	51	28	105.5	32.5	64	10	38	40
14983	48	49	31	105.5	45	57	11	49.5	48
14984	79.5	61	31	109	38	88	13	55	49
14985	87	57	28	160	20	94.5	10	37	54
14986	52	51	25	67	27.5	69	10	37	46
14987	45	45	26	65.5	20	57	9	45.5	40
14988	83	70	31	102	39	69.5	25	44	64
14989	49	57.5	27	103	29.5	61	14	35	54
14990	73.5	91	30	113	43	83.5	27	50	89
14991	45	49	25.5	91	29	56.5	14	34	45

14992	171	62	23	95	51.5	77	18	41	54
14993	49	61	25	88.5	38	55	17	37	111.5
14994	93	98.5	33	128	34	216	21	93.5	95.5
14995	45	56	25	101	34	59	16	35	67
14996	59	80	27	115	43	73	22	42	78
14997	69	69	28	124	36	63	17	37	60.5
14998	56.5	51	23	103	52	42	16	30	44
14999	54	60	22	91	33	85	14	48	56
15000	65	68	32	125	46.5	64	21	49	119
15001	42	60	23	111	32.5	59.5	18	34	56
15002	43	54	23	99.5	36	53	13.5	34	47
15003	46	55	27	96	25	59	16	34	61.5
15004	46	64	23.5	110	32	59.5	18	39.5	67
15005	61	80.5	29	118	34	65	21	46	56
15006	58	58	24	87.5	35	60.5	18	42.5	46.5
15007	42	57	27	101	34	55	16	39	75
15008	47	60	23	103	34	63	22	39	50
15009	70.5	76	30.5	142.5	56	68	21	51.5	107
15010	44	41	23.5	101.5	26	50	10	33	39
15011	48	58.5	22.5	104	34.5	55	14	37.5	58
15012	40	48	29	95	21	53	12	35	58.5
15013	50	58	29	100.5	185.5	59	16	36.5	73
15014	55	65	26	109	37	66	22	42	73
15015	42	47	22	95	23.5	53	10.5	31.5	37
15016	54	65	25	113	28	57	16	39	53
15017	61	55	22	106	203	52.5	15	52	49
15018	47	53	24	116.5	38	46	16	37	87
15019	44	62	20	92	26.5	53	11	30	46
15020	45	61	24	100	28	56	16	36	55
15021	56	71.5	26	124	52	64	21.5	41	61
15022	51	74	28	135.5	37	68	19.5	41	73
15023	56	69.5	33.5	127	35	66	18	45	95.5
15024	51	65	30	96.5	32	66	19	42	65
15025	100.5	68.5	29	133.5	60	64	20	41	132
15026	72	88.5	31	142.5	71	66	24	45	93
15027	54.5	55	31	116	41	59.5	17	37	63
15028	81.5	78	28	125	37	87	19	47	96
15029	40	46	24	91.5	30	53	13	40	65
15030	56.5	61	25	103	31.5	55	19	35	55
15031	57	53	22	103	27.5	65.5	14	33	54
15032	52	37	25	97.5	173.5	51	26	29	29
15033	34.5	31	15	51.5	27	46.5	12	24.5	27
15034	148	31	17	55.5	20	43	5	24	25
15035	37	34	16	58	41	48.5	5	25	38
15036	31.5	33	16	67	26.5	45	6	22	27
15037	35.5	36	21	191	15	50	7	37	43
15038	32	35	17	58	13	46	6	23	25

15039	29.5	30	24	53.5	15	42	5	21	26
15040	54.5	38	26	156	80	50	23	87.5	40.5
15041	31	33	14	66.5	17	39.5	6	21	23
15042	50	31	15.5	53	22	44.5	7	23	32
15043	38	28	20.5	74	54	36	12.5	31	42
15044	28	30.5	13	41	12	42	5	18	26
15045	40	33	19	58	23	45	8	35	26
15046	32	28.5	16	59	15	41	7	32	25
15047	49	35	23	65	21	54	17	29	33
15048	89	61.5	18	51	14	243	6	96.5	52
15049	27	24.5	14	62.5	17	37.5	5	20	27
15050	53	35.5	22.5	82	33	43	9	45	37
15051	32.5	31	20	58	26	45	8	27	30
15052	62	39	18	70	1507	78	16	28	51.5
15053	116	35.5	19.5	63	115	46.5	11	24.5	30
15054	117.5	32	24	61	26.5	47	11	28	56.5
15055	48.5	32	19	59.5	17.5	45	10	23	43.5
15056	34	33	21	57.5	65	44	7	80	27
15057	210	42	33	283	154	54	20	52	42.5
15058	27	28	14	49	15	37	8	21	22
15059	52.5	32	18	55	15	44	6	27.5	25
15060	27	32	14	53	13	41	5	21	24
15061	33	31	17	71	16	43	6	24	26
15062	36.5	31	26	57.5	17	39.5	6	28	52.5
15063	31	32	18	55	15	40	5	22	26
15064	110	44	22	92	192.5	135	8	31	44
15065	47.5	28	15	48	23	43	29	19	25
15066	49.5	33	18	51	19	43	21	25.5	72
15067	44.5	30	14	52	81	43.5	6	24	24
15068	38	46	23	60.5	16	52	7	111.5	27
15069	42.5	43	27	105	26	59	19	31	36
15070	41	45.5	23	71	27	57	8	32	36
15071	43	42	20	54	20	57	7	28	57
15072	48.5	46	23	144.5	22	58.5	14	36	36
15073	38	38	23	82	28.5	55	9	32	32
15074	43.5	44	26.5	104	45	59	9	44	52
15075	47	45	22	57	22	58	16	34	36
15076	101	68	35.5	83	772	74	18	51.5	56
15077	52	42	19	48.5	161	63	7	28	34
15078	56	55	37	77	31	63	18	40	58
15079	102	45	28	112	84.5	64	17	37	43
15080	42	41.5	17	56	37	53	8	28	40
15081	44.5	43	28	64	111.5	64	18	37	38
15082	82	42	19	66	22.5	56	9	28	37
15083	32	36	16.5	44	12	50	5	26	29
15084	42	42	21	87	16	60.5	7	34	39
15085	41	52.5	23	81	45.5	63	15	38	44

15086	41	31.5	18	100	451.5	36.5	7	32	26
15087	44	43	26	99	28	53	7	34	32
15088	40.5	41	19.5	63	16	52	7	31.5	31
15089	45.5	39	24	119	18	52	8.5	34	33
15090	44	37	20	52	16	47.5	8	26	30
15091	85	82	35	143	39	120	19	79	95
15092	74.5	92.5	33.5	169.5	42	73	29	51	83
15093	54	59	33	127.5	41	63	19	46.5	57
15094	59.5	65	32.5	144	32	65	19	47.5	64
15095	86	94	32	187	40	94.5	27.5	58	103
15096	60	60.5	32	138	42	64	19	45	80
15097	63	77.5	32	143.5	44	70.5	26	52.5	96
15098	69	73	30	162	39	64.5	23	42.5	80
15099	79	65	31	233	34.5	61	18	47.5	89
15100	71	89	34	154	42	77.5	27	56	67
15101	69	74	28	134	35	87	17	42	59.5
15102	62	65.5	28	169	44	64	18	41.5	53
15103	53	62.5	27.5	152.5	28	58	17	41	61
15104	47	53	26	125	26	55.5	14	35	49
15105	54	64	26	137	35.5	63.5	19	42	64.5
15106	59	48	25.5	123	45.5	53	11	38	39
15107	60	62	28	133	44	65.5	17	42	75
15108	70.5	83.5	31.5	152	49	75	28	52	76
15109	71	83	34	153	48.5	79	28.5	50.5	89
15110	58	69	28	138.5	41	69	19	43.5	81
15111	60.5	56	26	125	32	62	14	37.5	66
15112	64	78	38	169	94	63	28	59	82.5
15113	61	78	33	161	41	70	25	46	69
15114	61.5	73	32	155	32.5	61	22	56	72
15115	60	82	30	146.5	44	72	22	45.5	78
15116	47	44	27	122	21	54	13	32.5	65
15117	48	57	27	117	31	58	15	42.5	66
15118	50	55	28	121	35	57	17	35	66
15119	62	85	34	169	41.5	72	24	52	71
15120	52	69	31	144.5	42	61	24	39	67.5
15121	47.5	54.5	21	107	29	55	11	33	39
15122	58	75	30	162	38	63	24	45	67
15123	50	52.5	23	118	28	55	15	37	66
15124	52	66	29	121	490	67	17	41	69
15125	61.5	66	28	132.5	52	62	19	41	65
15126	53	73	24	148.5	41	66	19	42	59
15127	48.5	51	24	119	31	50	14	32.5	76
15128	66	92	31	184	46.5	74	27	51	70
15129	48	58	34	116.5	27	55	16	34.5	56
15130	61.5	76	28	132	36	71	22	43.5	66
15131	50	51	23	121	27	49	14	40	45
15132	47	54	25	150	26	53	13	38.5	52

15133	53	55	27	134	33	53	19.5	41	59
15134	123.5	62	27	130	29	53	17	39	60
15135	144.5	80	31.5	144.5	36.5	64	25	45	71
15136	59	54	23	113	34	63	17	36	68
15137	39	34	20	116	15	44	7	27	28
15138	47.5	35	20	92	18	45	10.5	24	35
15139	34	34	18	87	28	43	6	25	31
15140	34	33	18	57	18	42	8	22	29
15141	50	36	18	98	22	42	7	28	29
15142	42	44	26	80	26.5	62	13	32	37
15143	43	36	24	136	487	44.5	8	45	75
15144	39	32	20	61	25	43	8	23	29
15145	70	48	27	94.5	31	73	9.5	37.5	49
15146	35.5	31	16	62	19	40	8	23.5	27
15147	38	34	17.5	74	16.5	41.5	12	24	27
15148	37	36	23	80	23	45	8	31	59
15149	36	34	21	83	29	46.5	18	37	42
15150	52	40	23	135	16	46	11	43.5	42
15151	39.5	37	20	99	25.5	44	8	28	47.5
15152	63.5	32	21	81	20	42	9	27	33.5
15153	33	31	18	68	15	43	8	39	27.5
15154	57	65.5	28	111	35	65.5	14	39.5	57.5
15155	69	60	32	100	30	61	19	43	64
15156	56	67	29.5	96	41	61	22	43	68.5
15157	62.5	60	32	109	33	68.5	18	40	54
15158	77.5	92	37	154	68	113	19.5	64.5	93
15159	83	76	33	121	243	97	15	50	72.5
15160	50	47	29	80	42	57	18	37	55
15161	53	57	26	91.5	32	52.5	14	33	71
15162	48	59.5	26	108.5	29	60	15	40	90
15163	61	68	28.5	113	33.5	62	16	41	60
15164	58	68	32	113	39	66.5	31	45	84
15165	56	71	37.5	125	38	69	18	40	57
15166	52	77	27	89.5	32	70	14	42	58.5
15167	55.5	64	30	113	36	72	17	44	79
15168	58	80.5	29	133	42	75	24	47	66
15169	56.5	55	27	104.5	40	59	16	38	60
15170	53	64	27	137	33	64	16	36	65
15171	64	69	35	117	37	64	19	38	60
15172	64	84	33	117	47	74.5	29	47	70.5
15173	57	67	27.5	107	40	60	15	33.5	68
15174	52	60.5	31	116	35	62	16	39	59
15175	70.5	89	50	134	49.5	80	23	54.5	106
15176	50	70	29	124	35	63	17.5	42.5	71
15177	57	65	27	101	47	64	15	39	64
15178	51.5	57	24	122	35	63	15	40.5	49
15179	71.5	68	31.5	121	35	71.5	19	44	75

15180	65	84	28	128	45	70	23	47	67
15181	64	88	27	129	43	70.5	23	42	97.5
15182	57	74	29	120	34	70	20	43	75
15183	65	83	32	149	45	73	22	57	102
15184	61	82	30	157	40	69.5	22	58.5	80.5
15185	52	60	27	101	29	63	14	36	51
15186	65.5	71.5	32	138	50	67	21	45.5	63
15187	65	82	31	131.5	43	72	25	48	74
15188	90	88	31	122	71	87	23	43	74
15189	59	79	38	138	38.5	72	25	43	70
15190	63	70.5	36	114	63	66.5	19	43	66
15191	82	96.5	35	151.5	49	82	28	55.5	99
15192	55	66	26	117	34	63	15	39	63
15193	52.5	59	29	108	41	64	16	38	60
15194	55.5	76	32	102	33	58	22	42	64
15195	56	76	31.5	120	46	71	24	44.5	74
15196	52	62	27	108.5	32	60	16	36	66
15197	52	69.5	28	113.5	33	58	22	39	70
15198	79	92	33	140	51	79	30.5	56	93.5
15199	48	64	27	113	35	64	15	38	61
15200	54	64	28	118	32	69	15	37	68
15201	52	48	25	106	27	59	10	31.5	55
15202	53	71	28.5	110	28	62	18	40	60.5
15203	59	69	29	166	35	71	18	40	59
15204	56	57	27	105	34	59	15	34.5	54
15205	75	48	24	97.5	33	50	13	33	63
15206	62.5	84	30	149	40	67	21	49	88
15207	86	117	35	180	44.5	81	31	57	85
15208	57	58	26	99	30	62	14	37	80
15209	53.5	64	24	98	26	66	14	35	59
15210	56.5	70	28	101	32	63.5	19	40	55
15211	67	77	31	126	45.5	77	20	46	73.5
15212	68	59	27	110	29	62	16.5	39	60
15213	66	87	29	148	38	71	23.5	47	74
15214	71	69	30	132	36	77	19.5	51	74.5
15215	62	54	26	134	35	68	13	37	60
15216	51	59	25	109	29	69	15	35	57
15217	54	61	25	110	31	60.5	21	37.5	55.5
15218	80	104	33	154	49	81.5	35	65	102
15219	56.5	74	30.5	113	37	67	21	42	61
15220	50.5	62	24.5	113.5	27	64	16	36	68.5
15221	58.5	70	27	108	34	66	16	42	76
15222	64.5	72	28	128	40.5	64	19	43	61
15223	59.5	60	24	112	25	67	14	40	72
15224	54.5	80	28	137	33	71	24	48	77
15225	56	70	28.5	111.5	51.5	72	18	42	70
15226	68	70	27	116	30	82	18	47	88.5

15227	31	34.5	18	55	18.5	47	8	21	32
15228	38	40	19	72.5	23	54	8	26	47.5
15229	34	40.5	18	62	19	53	7	23	36
15230	38	40	20	62	18	56.5	8	30	38
15231	32	19	20	54	16	29	7	28	23
15232	31	34	16	55	16	43	6	21	31
15233	53	33	15	53	23	45	16	24	25
15234	39	41	20	83	26	49	11	36	45
15235	37	36.5	20	67.5	58	46.5	8	26	28
15236	68.5	34	18	73	27	43.5	8	24	56
15237	31	31	15	50.5	17	39.5	6	22.5	25
15238	45	39	19	55.5	66	48	11	28.5	31.5
15239	54	34	20.5	47	27	46	10	31	32.5
15240	51	32	21	58	31.5	41.5	10	22	28.5
15241	46	37	24	63	28.5	51	16	28	35
15242	36	37	28.5	129	17	52.5	15	29	36
15243	45	43	21	103.5	30.5	50	8	31	66
15244	40	35	21	102	27	50	11	39	43
15245	33.5	36	27	74	25	43	8	27	27
15246	38	44	18	95.5	18	50	8.5	25	34
15247	33	34	18.5	59	18	42.5	8.5	24	26
15248	52	37	24	108.5	29	49	11	32	43
15249	69	36	22	92	34	47	10	30	43
15250	49	37	21	99.5	547	55	56	29	127.5
15251	43	41	20	65	19	55	9	29	55.5
15252	38	39	22	89	39	48	9	32	81
15253	51.5	42	22	102	25	58.5	9	32	39.5
15254	42	33	17	74.5	15	54	7	24	36
15255	39	32	18	71	17	41	7	23	31
15256	36.5	31	20	66	68	48	9	29	36
15257	40	33	17	59	15.5	42	5	24	27.5
15258	34	33	16	61	17	43	6	23	30
15259	33	36	21	64	20	44	9	26	36.5
15260	57	39	22	93.5	24	49	13	30	35
15261	37	36	21	69	24	50	7	26	30
15262	55	36	20	52	26.5	67	8	31	32
15263	35	29.5	16.5	50.5	29	39.5	7	21	27
15264	36	33	17	66	15	45	10	26	95
15265	39	35	16	53	18	50	6	23	30
15266	48	36	20	61	26.5	57	7	29	32
15267	34	33	17	51	28	43	8	20	27
15268	39.5	35	18	70.5	17	42	11	26	70
15269	85.5	35	19	60	25	48.5	15.5	26	45.5
15270	35.5	30	19	59.5	18	47	6	28.5	29
15271	49	34	45	64	33	54	10	28	34
15272	57.5	35	19	58	23	53	8	36	46
15273	54	33	20.5	49	18.5	47	21	26	28

15274	62.5	34	21	66	24	48	8	25.5	31
15275	43	34	20	58	25	47	8	28	29.5
15276	67	36	22	83	297	47	13	28	30
15277	29	30	16	72	20	40	6	22	28
15278	28	28	15	46	29	40	5	20	28
15279	33	30	17	66	16	43	7	22	25.5
15280	47	31	16	64	23.5	42	6	27	26
15281	30	32	17	55	17.5	40	6	22.5	25.5
15282	49	31	18	52	42	42	20	25	33
15283	37	32	16	65.5	18	40	10	23	29.5
15284	41.5	31	23	121	22	41	11	31	37
15285	34.5	32	19	80.5	18	43	27	26	30
15286	48	34	23.5	163	19	45.5	13	106	27
15287	38	33	18	87	23	44	7	26	46
15288	45	30	22	84	21	43	22	29	50
15289	105	41	36	202.5	26	53	16	104	81
15290	57	38	25.5	99	21	49.5	10	40	46.5
15291	50	34.5	25	99.5	29.5	47	12	33	38.5
15292	39	33	24	124	19	47	10.5	37	50
15293	56	52.5	28	116	52	84	28	36	71.5
15294	89	60	23	151	117	107	11.5	37	132.5
15295	41	34	19	99	45	47	9	29	66
15296	40.5	35	23	103	38	49	17	28	92
15297	93	64	36	202	43	83	28	101	135
15298	62	58	28.5	130	40	54	17	40	67.5
15299	61	87	28	127	41	68	25.5	47.5	69.5
15300	57	69	27	107	41	59	20.5	41.5	56
15301	62	76	27	132.5	43	67	20	44	73
15302	63.5	77	34	126	34.5	80	24	52	97
15303	56	64	28	119.5	38	59	17	43	64
15304	51.5	54	21	97	90	50.5	13	33	40
15305	41	46	26	83	30	50	11	30	54
15306	54	65	28	120	37	60	20	42	49
15307	65	77.5	36	142	38	70.5	28	50	72
15308	54	66	27	109	30	59.5	14	37	58
15309	45	55	24.5	117	32	56	15	37	48
15310	60	72	28	122.5	32	67.5	18	41	71
15311	50	68	26	127.5	35.5	60	17	40	71.5
15312	76	94	38	152	50	71.5	29	82	77.5
15313	48	47	24	90.5	43	51.5	12	34	48
15314	62	65	31	113	33	66	17	42	63
15315	57	51.5	25	97	24	112	9	50	49
15316	51	55	28.5	103.5	33	51	17	33	46
15317	100.5	57	24	106.5	95.5	56	16	39	52
15318	51	68	30	121	30	58	19	42	58
15319	49	57	22	95	31	57	14	33	57.5
15320	62.5	93	33	131	45	70	30	54	79

15321	53	79	29	119	39.5	66	26	43.5	68
15322	60	73.5	28	117	39	66	20	42	63.5
15323	48	58	23.5	105.5	33	55.5	15	37	50
15324	72.5	100	33	136	46	134	24	76	76
15325	50	50	27	100	29	49	14	34	57
15326	59	75	31	137.5	35	69.5	23	46	70.5
15327	67	90	30	121	38	67.5	25	49	103
15328	56	95	34	139	49	69	28	56.5	85.5
15329	246	54.5	32	101	33.5	62	21	46	57.5
15330	59	77	31	132	36	58	26	43	65
15331	69.5	59	26	107	36.5	52	16	40	48
15332	72	70	28	110	41	84.5	16	40	60
15333	65	90	35.5	129.5	40.5	69	29	55	108
15334	62	58	28	120	36	54	21	36	51
15335	87.5	106.5	36	159	47	88	33	92.5	97
15336	68	100.5	36	139.5	46	77	32	62	86
15337	51	66	41	105	30	57	15	42	55
15338	54	71	42.5	125	28	63	19	42	58.5
15339	54.5	67	27.5	106	30	55	21	41	55
15340	48	64	27	121	48	63	17	40.5	47
15341	63	92	34	170	102	66	30.5	50	74
15342	47	59	26.5	96	31	52	15	33	44
15343	54	70	23	135.5	34.5	60	17	38	69
15344	68	89	31	140	44	73.5	27	55.5	102
15345	79	96	40.5	159	56	73	34	59	78.5
15346	111	85	26	151	34	65.5	23	41	83
15347	53	83	26	135	36	64	23	46	71
15348	50	55.5	25	124.5	26	51	14	39	54.5
15349	55	71	27.5	122	32	64	19	44	62
15350	52.5	70.5	27	124	43.5	59	20	39	69
15351	62	88	35	149	43	75	22	42	81
15352	60	77	30	132	33	66	20	46	92
15353	50	59	27	101	28	60	17	37.5	58.5
15354	48	55.5	27	128.5	31	56.5	14	39	68
15355	70	105	35	151	171	79	34.5	56	80.5
15356	74.5	80	43	131	55	74.5	32	64	85.5
15357	75.5	85	31	178	37	66	28	51.5	80
15358	75	86.5	30	152	59	85	22	51	75
15359	107	72	28.5	136	32	68	25	44	77
15360	42	34	23	96	19	40	8	28	33
15361	43.5	59.5	25	109	29	51	13.5	34	48
15362	46	58	21.5	105	27	52.5	16	33.5	53
15363	46.5	61	25	143	26	56.5	19	41	73
15364	69	97	31	143.5	42	72.5	28	52	91
15365	74	85	34	134	48	77	28	62	69
15366	57	69	27	117	32	69.5	17	71.5	56
15367	43	48	24	102	25	51.5	12	31	63

15368	49.5	77	26	112	35	64	22	53.5	63
15369	43.5	51	23	110	24	47	15	30	60
15370	42.5	61	20	97	24	50.5	13	30	55
15371	60	93.5	33	134	39	67	29	49.5	76
15372	42	61	25	105	28.5	53	16	32	57.5
15373	52	66	26	113	43	56	16	43	46
15374	65.5	65	24	133	42	57	15	37	56
15375	48	68	24	109	29	54	17	36	62
15376	56	54	25	117	30.5	50	16	41	54
15377	50	63	26	120	37	56	18.5	53	49
15378	93	62	25	117	27	48	20.5	37	54.5
15379	51	64	24	120.5	28.5	49.5	19	37	56
15380	44	46.5	22	87	26	53	11	32	46
15381	66	74	28	106	33	81	17	43	60
15382	78	70	25	102	24	94.5	12	45	65
15383	43.5	56.5	23	106.5	25	50	14	36	48
15384	57	88	33.5	126.5	82	76	47.5	46.5	70
15385	50	44	26	99	24	49	10.5	31	47
15386	46	46	27.5	86.5	26	59.5	8	38	38
15387	58	68	28	105.5	45	69	17	38.5	66
15388	64	88	36	149	49	79	28	48	85
15389	88	105	36	154	40	86	30	57	82
15390	83	82.5	29	126.5	43	77	20	53	61
15391	47	48	31	78.5	20	59	9	43.5	43
15392	45.5	42	28	100.5	24	49	9	34.5	39
15393	45	62	29	86	21	55	17	29	36
15394	47.5	45	29	95	22	57	25	30	43
15395	54	52	36.5	109	26	60	15	37	42.5
15396	46	47	23	74	22	54	27.5	32	41
15397	53.5	68.5	24	78	23	69	13	34	44
15398	181	47	23	76	27	59	11	36	37
15399	45	42	22	63	19	50	12	26	31
15400	49.5	42	25	89	32	49.5	12	29	34
15401	49.5	50	29	101	23	45	26.5	27	31
15402	25	7	6	18	7	8	5	10	51
15403	54	51.5	28	150	23	77	26	56	47

Table S4

Bayes Net

EDI	VAC	GD7	MHV	NEG	MYC	MVM	REO	PVM	SEN	classified
**100**	0	0	0	1	0	0	0	0	0	EDI
1	**68**	0	0	2	0	0	0	0	0	VAC
0	0	**113**	0	1	24	5	0	0	0	GD7
0	0	0	**72**	5	0	0	0	0	0	MHV
0	0	2	4	**296**	1	0	2	0	0	NEG
0	0	0	2	6	**84**	0	0	0	0	MYC
0	0	6	0	0	1	**69**	0	0	0	MVM
0	0	0	0	6	0	0	**107**	0	0	REO
0	0	0	0	0	0	0	0	**102**	0	PVM
0	0	0	0	3	2	0	0	0	**76**	SEN

Random Forest

EDI	VAC	GD7	MHV	NEG	MYC	MVM	REO	PVM	SEN	classified
**101**	0	0	0	0	0	0	0	0	0	EDI
1	**53**	0	0	16	0	1	0	0	0	VAC
0	0	**140**	0	0	3	0	0	0	0	GD7
0	0	0	**76**	1	0	0	0	0	0	MHV
0	0	1	0	**304**	0	0	0	0	0	NEG
0	0	1	0	2	**89**	0	0	0	0	MYC
0	0	2	0	1	0	**72**	0	0	1	MVM
0	0	0	0	0	0	0	**113**	0	0	REO
0	0	0	0	0	0	0	0	**102**	0	PVM
0	0	0	0	15	0	2	0	0	**64**	SEN

The abbreviations of the viruses are as follows: Epizootic diarrhea virus of infant mice (EDIM), Theiler's mouse encephalomyelitis virus/GDVII strain (GD7), Mouse hepatitis virus (MHV), mouse minute virus (MMV), Mycoplasma pulmonis (MYC), Pneumovirus of mouse (PVM), Respiratory enteric orphan virus (Reo-3 virus) (REO), Sendai virus (SEN), Ectromelia virus (ECTRO) with NEG stands for mice that are negative for any infection from these viruses.

	SI Table 5a: Performance of the testing set with various classification algorithms - TE											
		Table 3a: Performance of the testing set with various classification algorithms Test Efficiency (%)										
	Algorithm	NEG	EDI	GD7	MHV	MMV	MYC	PVM	REO	ECTRO	SEN	AVERAGE
1	Meta Class Classifer	90.8	97.3	98.8	99.8	95.1	99.7	95.8	99.9	99.5	99.0	97.6
2	J48	88.4	97.9	98.4	99.8	89.4	99.1	91.1	99.7	99.4	99.9	96.3
3	Simple Logistic	85.7	97.0	94.6	99.2	92.6	99.6	90.5	99.7	99.5	99.8	95.8
4	SMO	86.9	88.0	98.4	99.8	94.4	99.1	91.2	99.6	99.4	99.8	95.7
5	Multilayer Perception	83.1	98.3	98.7	99.8	80.5	99.3	90.5	99.7	99.4	99.8	94.9
6	Lazy ibk	81.3	97.1	98.2	99.9	84.4	99.4	90.7	99.8	99.4	92.0	94.2
7	LMT	78.5	95.3	87.5	93.0	87.2	99.5	90.6	99.7	99.5	99.1	93.0
8	Rules Decision table	78.2	91.3	98.7	99.4	95.6	98.9	90.4	99.6	99.4	99.8	95.1
9	Meta Bagging	76.2	98.2	90.7	99.5	74.4	99.3	89.3	99.8	99.3	99.9	92.6
10	Meta Logi Boost	75.5	98.3	92.1	99.5	73.5	99.9	89.3	99.8	99.1	99.9	92.7
11	Kstar	75.0	98.1	92.1	99.4	73.4	99.6	90.1	99.8	99.3	99.9	92.7
12	Rules PART	75.0	98.4	92.1	99.4	73.6	99.7	90.1	99.1	99.3	99.9	92.7
13	REP Tree	74.9	97.6	91.0	99.5	72.9	99.5	89.1	99.8	99.3	99.9	92.3
14	Meta Random Committee	74.8	97.1	90.5	99.4	73.6	99.5	89.1	99.8	99.3	99.7	92.3
15	Random Forest	74.4	97.4	92.6	99.4	71.8	98.8	89.1	99.8	99.3	99.8	92.2
16	Random forest subspace	72.3	97.6	90.1	96.9	73.5	99.7	88.3	98.5	99.2	99.7	91.6
17	Classification via Regression	71.7	94.9	84.6	99.2	76.2	98.6	88.7	99.4	99.5	99.9	91.3
18	Logistic	68.6	97.2	79.3	99.9	76.4	99.3	88.1	99.7	98.4	92.2	89.9
19	Rules One R	62.8	96.7	79.0	94.3	77.7	98.1	85.0	90.5	99.1	95.7	87.9
20	Rules Zero R	62.8	96.7	79.0	94.3	77.7	98.1	85.0	90.5	99.1	95.7	87.9
21	Bayesnet	57.5	93.5	82.7	95.2	60.0	97.6	86.0	99.4	98.8	99.6	87.0
22	Random tree	53.3	95.7	87.4	99.2	73.4	71.8	85.2	99.7	81.5	99.7	84.7
23	Naives Bayes	42.1	90.7	68.7	98.4	45.9	98.2	81.0	97.1	97.3	99.7	81.9
24	Meta Filtered Classifier	36.5	95.2	92.2	98.8	38.2	92.2	72.0	99.4	85.4	96.7	80.7
25	NaiveBayes Updateable	0.9	25.5	38.2	2.0	28.4	1.7	8.1	51.4	4.2	84.3	24.5
26	Attribute Selection Classifier	0.4	30.7	25.7	0.5	23.6	1.4	4.1	18.5	4.9	72.4	18.2

SI Table 5b: Performance of the testing set with various classification algorithms - MCC

		Correlation Coefficient (%)										
	Algorithm	NEG	EDI	GD7	MHV	MMV	MYC	PVM	REO	ECTRO	SEN	AVERAGE
1	Meta Class Classifier	59.2	58.2	6.7	79.9	2.7	83.4	70.9	79.3	39.9	16.6	49.7
2	J48	16.5	32.0	5.2	77.6	18.8	25.3	11.0	38.4	16.3	40.2	28.1
3	Simple Logistic	37.9	35.1	27.5	62.2	2.9	80.1	8.4	55.8	46.1	45.0	40.1
4	SMO	7.4	25.1	(0.7)	77.6	2.6	13.4	11.1	14.4	11.5		18.0
5	Multilayer Perception	14.7	57.5	(0.1)	83.3	10.1	52.4	14.8	47.8	25.7	19.0	32.5
6	Lazy ibk	27.3	58.0	11.0	88.2	7.9	73.1	24.1	65.4	38.2	6.5	40.0
7	LMT	35.7	42.2	23.0	25.4	0.5	73.6	30.1	49.9	44.6	27.0	35.2
8	Rules Decision table	(10.2)	(2.0)		12.1	2.8	(0.3)		25.0			4.6
9	Meta Bagging	21.0	59.9	29.5	45.3	(2.3)	67.2	4.6	72.5	11.5	73.8	38.3
10	Meta Logi Boost	24.5	65.1	31.0	38.2	(2.1)	96.7	10.4	73.5	47.4	73.8	45.9
11	Kstar	27.5	62.0	35.9	24.2	(2.5)	83.0	29.1	71.1	11.5	83.1	42.5
12	Rules PART	27.6	67.3	35.6	32.0	(2.1)	84.9	29.1	40.3	11.5	85.4	41.2
13	REP Tree	23.3	59.3	29.7	45.3	(2.0)	73.0	2.7	71.3	16.3	73.8	39.3
14	Meta Random Committee	23.2	55.2	29.5	34.2	(2.5)	73.9	2.6	72.5		14.1	33.6
15	Random Forest	23.0	57.7	26.8	40.1	(2.6)	53.9	11.2	72.5		24.6	34.1
16	Random forest subspace	21.8	60.0	29.3	7.5	(2.3)	87.9	(2.6)	28.6	(0.2)	19.4	24.9
17	Classification via Regression	24.3	44.2	24.4	11.3	(1.6)	52.9	5.3	44.1	60.6	76.8	34.2
18	Logistic	20.4	34.8	4.2	89.2	(2.1)	70.3	9.0	62.7	39.4	9.7	33.7
19	Rules One R	0.4		22.2	(2.0)	(3.6)	(0.8)	(5.9)	(1.7)		(1.0)	0.9
20	Rules Zero R	0.4		22.2	(2.0)	(3.6)	(0.8)	(5.9)	(1.7)		(1.0)	0.9
21	Bayesnet	13.6	14.4	22.2	2.1	(5.1)	49.7	(0.1)	33.2	(0.5)	27.7	15.7
22	Random tree	10.1	36.7	28.8	32.0	(3.5)	0.4	4.3	72.5	(3.6)	21.3	19.9
23	Naives Bayes	10.3	8.6	9.5	3.9	(7.9)	48.5	(4.3)	8.7	5.7	48.3	13.1
24	Meta Filtered Classifier	0.5	46.9	25.8	38.1	(6.5)	5.8	(15.4)	61.1	(4.0)	0.6	15.3
25	NaiveBayes Updateable	(51.0)	(50.7)	(33.9)	(60.7)	(55.7)	(71.6)	(23.1)	(32.0)	(11.8)	56.2	(33.4)
26	Attribute Selection Classifier	(78.1)	(11.1)	(22.4)	(70.5)	(50.7)	(76.3)	(47.5)	(59.3)	(51.9)	50.5	(41.7)

Numbers in the parenthesis represent negative values.

No	Classification Scheme	References
1	Meta Class Classifier	[1]
2	J48	[2]
3	Simple Logistic	[3]
4	SMO	[4]
5	Multilayer Perception	[5]
6	Lazy ibk	[6]
7	LMT	[7]
8	Rules Decision table	[8]
9	Meta Bagging	[9]
10	Meta Logi Boost	[9]
11	Kstar	[10]
12	Rules PART	[11]
13	REP Tree	[12]
14	Meta Random Committee	[13]
15	Random Forest	[14]
16	Random forest subspace	[14]
17	Classification via Regression	[15]
18	Logistic	[15]
19	Rules One R	[16]
20	Rules Zero R	[16]
21	Bayesnet	[17,18]
22	Random tree	[19]
23	Naives Bayes	[20-22]
24	Meta Filtered Classifier	[9]
25	NaiveBayes Updateable	[20-22]
26	Attribute Selection Classifier	[23]

1. Bergamo A, Torresani L, Meta-Class Features for Large-Scale Object Categorization on a Budget. Computer Vision and Pattern Recognition (CVPR) (2012) Meta-Class Features for Large-Scale Object Categorization on a Budget. Computer Vision and Pattern Recognition (CVPR), 2012 pp. 1-8.
2. Poropudas J, Virtanen K (2007) Analyzing air combat simulation results with dynamic Bayesian networks: IEEE Press. 1370-1377 p.
3. Landwehr N, Hall M, Frank E (2005) Logistic model trees. Machine Learning. Machine Learning 59: 161-205.
4. Keerthi SS, Shevade SK, Bhattacharyya C, Murthy KRK (2001) Improvements to Platt's SMO algorithm for SVM classifier design. . Neural Computation 13: 637–649.
5. Witten IH, Frank E, Hall MA (2011) Data mining : practical machine learning tools and techniques. Burlington, MA: Morgan Kaufmann. xxxiii, 629 p. p.

6. Beygelzimer A, Kakade S, Langford J. Cover trees for nearest neighbor; 2006. ACM. pp. 97-104.
7. Gama J (2004) Functional trees. Machine Learning 55: 219-250.
8. Kohavi R. A study of cross-validation and bootstrap for accuracy estimation and model selection; 1995. pp. 1137-1145.
9. Bauer E, Kohavi R (1999) An empirical comparison of voting classification algorithms: Bagging, boosting, and variants. Machine learning 36: 105-139.
10. Cleary JG, Trigg LE. K^*: An Instance-based Learner Using an Entropic Distance Measure; 1995. pp. 108-114.
11. Frank E, Witten IH (1998) Generating accurate rule sets without global optimization.
12. Furnkranz J, Widmer G. Incremental reduced error pruning; 1994. pp. 70-77.
13. Domingos P. Metacost: A general method for making classifiers cost-sensitive; 1999. ACM. pp. 155-164.
14. Breiman L (2001) Random forest. Machine Learning 45: 5-32.
15. Breiman L, Friedman J, Stone CJ, Olshen RA (1984) Classification and regression trees: CRC press.
16. Kohavi R (1995) The power of decision tables. Machine Learning: ECML-95: Springer. pp. 174-189.
17. Wu X, Kumar V (2009) The top ten algorithms in data mining. Boca Raton: CRC Press. xiii, 215 p. p.
18. Wu X, Kumar V, Ross Quinlan J, Ghosh J, Yang Q, et al. (2008) Top 10 algorithms in data mining. Knowledge and Information Systems 14: 1-37.
19. Webb GI. Decision tree grafting from the all-tests-but-one partition; 1999. Citeseer. pp. 702-707.
20. Jiang L, Zhang H (2006) Weightily averaged one-dependence estimators. PRICAI 2006: trends in artificial intelligence: Springer. pp. 970-974.
21. Zheng Z, Webb GI (2000) Lazy learning of Bayesian rules. Machine Learning 41: 53-84.
22. Zheng Z, Webb GI, Ting KM. Lazy Bayesian Rules: A lazy semi-naive Bayesian learning technique competitive to boosting decision trees; 1999. Citeseer.
23. Goldberg DE, Holland JH (1988) Genetic algorithms and machine learning. Machine learning 3: 95-99.
